# Supplementary material for: Enantioselective synthesis of chiral amides by carbene insertion into amide N–H bond
Source: Nat Commun. 2024 Jun 5;15:4793. doi: 10.1038/s41467-024-48266-5 (PMC11153641; doi:10.1038/s41467-024-48266-5)
Supplement: Supplementary file 1 — Supplementary Information [file 41467_2024_48266_MOESM1_ESM.pdf]

Supplementary Information for

Enantioselective Synthesis of Chiral Amides by Carbene Insertion into Amide N–H Bond

Xuan-Ge Zhang[a], Zhi-Chun Yang[a], Jia-Bin Pan[a], Xiao-Hua Liu[b], Qi-Lin Zhou[a]*
[a] State Key Laboratory and Institute of Elemento-Organic Chemistry, College of Chemistry, Frontiers Science Center for New Organic Matter, Nankai University, Tianjin 300071, China
[b] Key Laboratory of Green Chemistry & Technology, Ministry of Education, College of Chemistry, Sichuan University, Chengdu 610064, China

Content

1 Supplementary Methods **2**
1.1 General Information **2**
1.2 Reaction Optimization **2**
1.3 Synthesis and Analytical Data **5**
1.4 Determination of the Configuration of 7d **41**
1.5 Computational Study **42**
2 Supplementary Figures **49**
2.1 NMR Spectra **49**
2.2 HPLC Spectra **129**
3 References **197**

1 Supplementary Methods

1.1 General Information

Caution: This study involves the use of diazo compounds. Diazo compounds are known to have thermal stability issues and are explosive hazards; work with diazo compounds should be performed in a well-ventilated fume hood, require the use of personal protective equipment, and careful handling of the reagents.[1,2]

All reactions and manipulations were performed using standard Schlenk techniques. All solvents were purified and dried using standard procedure[3]. Dirhodium (II) carboxylates (Sigma-Aldrich or TCI), chiral organocatalysts (Daicel) and other commercially available reagents were used without further purification. α-aryl-α-diazoketones were prepared according to the reported procedures[4]. NMR spectra were recorded with a Bruker AV 400 and 600 spectrometer at 400 or 600 MHz (^{1}H NMR), 101 or 151 MHz (^{13}C NMR). Chemical shifts (δ values) were reported in ppm down field from internal Me_4Si. Melting points were measured on a RY–I apparatus and uncorrected. High Resolution Mass Spectra (HRMS) were recorded on an IonSpec FT-ICR mass spectrometer with Electron Spray Ionization (ESI) resource. HPLC analyses were performed on a Hewlett Packard Model HP 1100 Series chromatography. Optical rotations were determined by a Perkin Elmer 341 MC polarimeter.

1.2 Reaction Optimization

Supplementary Table 1. Enantioselective N–H insertion of α-diazoketone with amide: optimization of metal catalyst.

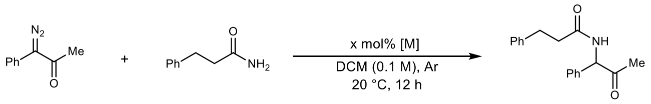

Entry[a]	x	[M]	Yield (%)[b]
1	1	$Rh_2(OAc)_4$	80
2	5	$Cu(MeCN)_4PF_6$	<5
3	5	CuTp*	<5
4	5	$Pd(MeCN)_2Cl_2$	trace
5	5	$FeCl_3$	trace
6	5	$[Ir(COD)Cl]_2$	<5
7	5	$CoBr_2$	<5

[a] Reaction conditions: α-diazoketone (0.2 mmol), 3-phenylpropionamide (0.2 mmol), DCM (2 mL), 20 °C, 12 h. [b] Isolated yield.

Supplementary Table 2. Enantioselective N–H insertion of α-diazoketone with amide: optimization of chiral organocatalyst[a].

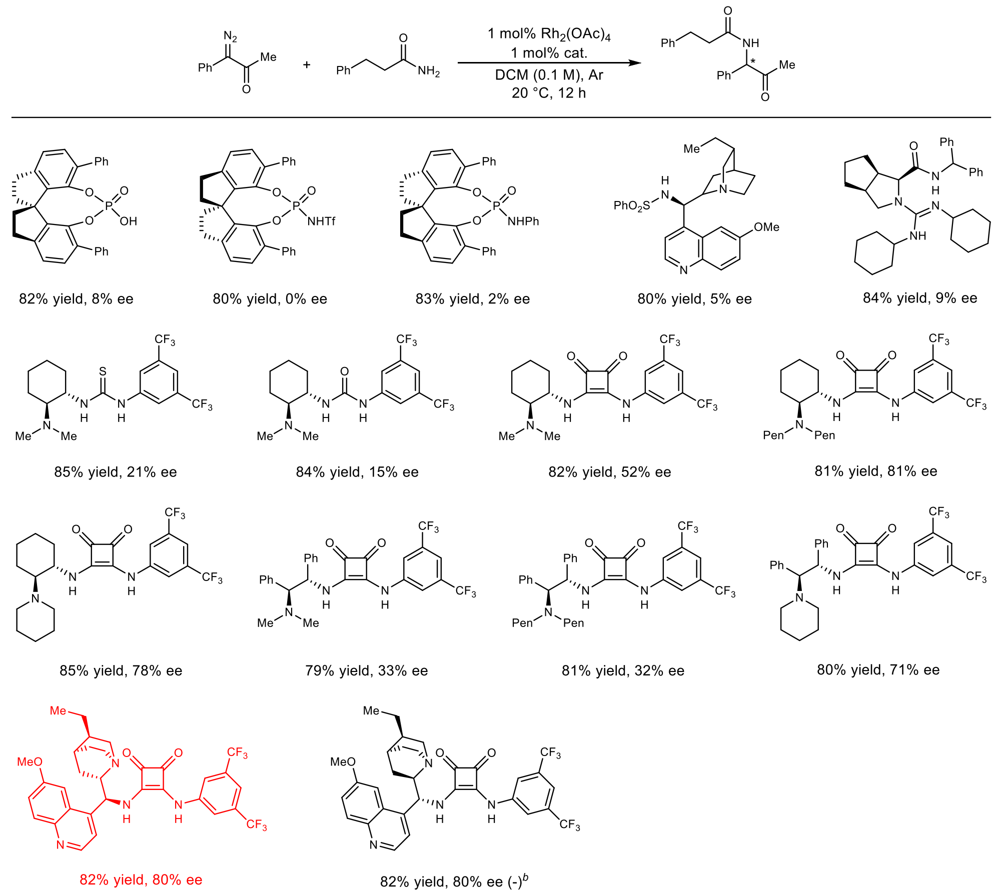

[a] Reaction conditions: α-diazoketone (0.2 mmol), 3-phenylpropionamide (0.2 mmol), DCM (2 mL), 20 °C, 12 h. Isolated yield was given. The ee values were determined by chiral HPLC. [b] The configuration is opposite to those obtained by using other catalysts

Supplementary Table 3. Enantioselective N–H insertion of α-diazoketone with amide: optimization of dirhodium catalyst.

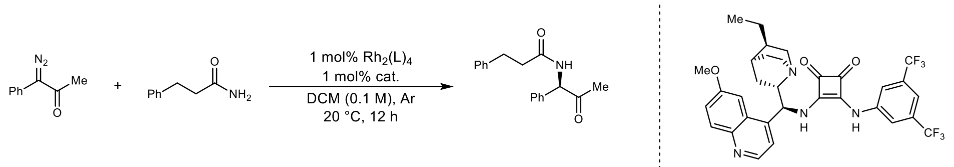

Entry[a]	$Rh_2(L)_4$	Yield (%)[b]	ee (%)[c]
1	$Rh_2(OAc)_4$	83	81
2	$Rh_2(oct)_4$	81	81
3	$Rh_2(TFA)_4$	75	71
4	$Rh_2(piv)_4$	92	76
5	$Rh_2(esp)_2$	86	75
6	$Rh_2(TPA)_4$·DCM	70	88
7[d]	$Rh_2(TPA)_4$·DCM	70	90
8[d, e]	$Rh_2(TPA)_4$·DCM	87	90

[a] Reaction conditions: α-diazoketone (0.2 mmol), 3-Phenylpropionamide (0.2 mmol), DCM (2 mL), 20 °C, 12 h. [b] Isolated yield. [c] The ee values were determined by chiral HPLC. [d] Reaction was ceased within 1 min. [e] Diazo ketone solution was added dropwise.

Supplementary Table 4. Enantioselective N–H insertion of α-diazoketone with amide: optimization of solvent.

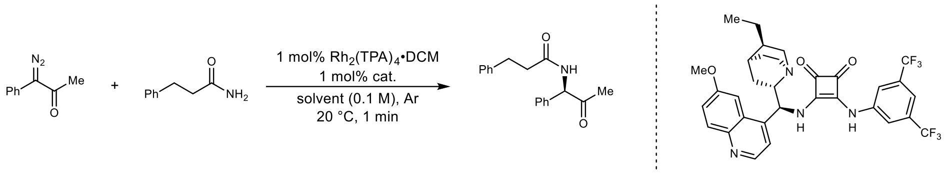

Entry[a]	Solvent	Yield (%)[b]	ee (%)[c]
1	DCM	90	90
2	DCE	85	90
3	$CHCl_3$	83	90
4	THF	76	50
5	toluene	61	87
6	MTBE	80	73
7	dioxane	78	71
8	*n*-hexane	37	86

[a] Reaction conditions: α-diazoketone (0.2 mmol), 3-phenylpropionamide (0.2 mmol), DCM (2 mL), 20 °C, 12 h. [b] Isolated yield was given. [c] The ee values were determined by chiral HPLC.

Supplementary Table 5. Enantioselective N–H insertion of α-diazoketone with amide: optimization of temperature.

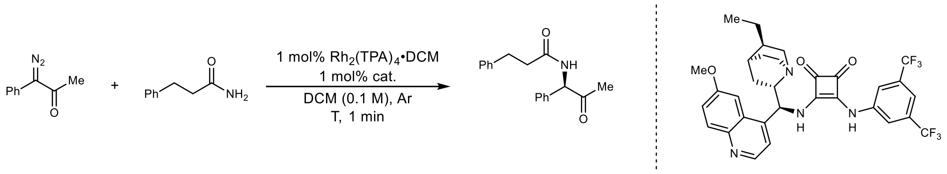

Entry[a]	T	Yield (%)[b]	ee (%)[c]
1	-20°C	94	90
2	0°C	99	92
3	20°C	90	90
4	40°C	70	90

[a] Reaction conditions: α-diazoketone (0.2 mmol), 3-phenylpropionamide (0.2 mmol), DCM (2 mL), 20 °C, 12 h. [b] Isolated yield was given. [c] The ee values were determined by chiral HPLC.

1.3 Synthesis and Analytical Data

Typical Procedure for the Synthesis of α-Diazoketones and Analytical Data

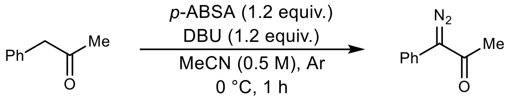

1-Phenylpropan-2-one (1.3 g, 10 mmol), *p*-acetamidobenzenesulfonyl azide (*p*-ABSA, 2.9 g, 12 mmol) and MeCN (20 mL) were introduced into an oven-dried 50 mL Schlenk tube under Ar protection. After cooled by an ice-bath, a solution of 1,8-diazabicyclo[5.4.0]undec-7-ene (DBU, 1.8 g, 12 mmol) in 5 mL MeCN was added dropwisely through a syringe. The mixture was kept stirring at 0 °C for 1 h and then at room temperature until the reaction completed. Then 20 mL 10 w% NaOH (aq.) was carefully added, and the mixture was extracted by MTBE (30 mL×3). The organic layers were combined, concentrated, purified by a flash chromatography on silica gel (PE/EA = 6:1).

1-diazo-1-phenylpropan-2-one (2a)

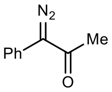

Orange solid, 1.4 g, 85% yield, TLC: R_f = 0.47 (PE/EA = 6:1, v/v).

^{1}H NMR (400 MHz, $CDCl_3$)
δ 7.49 (d, *J* = 7.8 Hz, 2H), 7.40 (t, *J* = 7.8 Hz, 2H), 7.25 (t, *J* = 7.5 Hz, 1H), 2.36 (s, 3H).

^{13}C NMR (101 MHz, $CDCl_3$)
δ 189.9, 129.0, 127.00, 125.8, 125.5, 72.7, 26.9.

1-diazo-1-(p-tolyl)propan-2-one (2b)

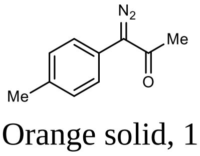

Orange solid, 1.4 g, 80% yield, TLC: R_f = 0.55 (PE/EA = 6:1, v/v).

^{1}H NMR (400 MHz, $CDCl_3$)
δ 7.38 (d, *J* = 7.9 Hz, 2H), 7.23 (d, *J* = 8.4 Hz, 2H), 2.37 (s, 3H), 2.35 (s, 3H).

^{13}C NMR (101 MHz, $CDCl_3$)
δ 190.2, 137.0, 129.8, 126.1, 123.6, 72.5, 26.8, 21.1.

1-diazo-1-(m-tolyl)propan-2-one (2c)

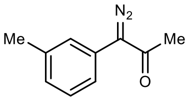

Orange solid, 1.5 g, 86% yield, TLC: R_f = 0.56 (PE/EA = 6:1, v/v).

^{1}H NMR (400 MHz, $CDCl_3$)

δ 7.30 – 7.10 (m, 3H), 7.06 – 7.00 (d, *J* = 7.4 Hz, 1H), 2.33 (s, 3H), 2.31 (s, 3H).

^{13}C NMR (101 MHz, $CDCl_3$)
δ 190.1, 138.8, 128.9, 127.9, 126.5, 125.4, 123.1, 72.7, 26.9, 21.5.

1-diazo-1-(4-(trifluoromethyl)phenyl)propan-2-one (2d)

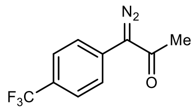

Yellow solid, 2.1 g, 92% yield, TLC: R_f = 0.50 (PE/EA = 6:1, v/v).

^{1}H NMR (600 MHz, $CDCl_3$)
δ 7.64 (m, 4H), 2.42 (s, 3H).

^{13}C NMR (151 MHz, $CDCl_3$)
δ 189.2, 129.9, 128.5 (q, *J* = 32.9 Hz), 125.8, 124.9, 124.0 (q, *J* = 271.9 Hz), 72.7, 27.1.

1-diazo-1-(3-methoxyphenyl)propan-2-one (2e)

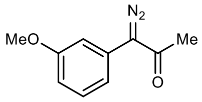

Orange solid, 1.5 g, 78% yield, TLC: R_f = 0.44 (PE/EA = 6:1, v/v).

^{1}H NMR (400 MHz, $CDCl_3$)
δ 7.3 – 7.2 (m, 1H), 7.2 (s, 1H), 7.0 – 6.9 (m, 1H), 6.8 – 6.7 (m, 1H), 3.8 (s, 3H), 2.4 (s, 3H).

^{13}C NMR (101 MHz, $CDCl_3$)
δ 189.8, 160.0, 129.9, 126.9, 117.7, 112.5, 111.5, 72.8, 55.3, 27.1.

1-diazo-1-(2-fluorophenyl)propan-2-one (2f)

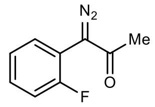

Red oil, 1.3 g, 71% yield, TLC: R_f = 0.52 (PE/EA = 6:1, v/v).

^{1}H NMR (400 MHz, $CDCl_3$)
δ 7.7 – 7.5 (m, 1H), 7.4 – 7.3 (m, 1H), 7.3 – 7.2 (m, 1H), 7.2 – 7.1 (m, 1H), 2.30 (s, 3H).

^{13}C NMR (101 MHz, $CDCl_3$)
δ 190.1, 160.3, 157.9,131.0, 130.0, 124.8 (d, *J* = 3.7 Hz), 116.0 (d, *J* = 21.4 Hz), 68.0, 26.1.

1-diazo-1-(4-fluorophenyl)propan-2-one (2g)

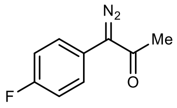

Orange solid, 1.5 g, 84% yield, TLC: R_f = 0.58 (PE/EA = 6:1, v/v).

^{1}H NMR (400 MHz, $CDCl_3$)

δ 7.53 – 7.41 (m, 2H), 7.14 – 7.06 (m, 2H), 2.35 (s, 3H).

^{13}C NMR (101 MHz, $CDCl_3$)

δ 189.8, 161.6 (d, *J* = 248.4 Hz), 127.7, 121.3, 116.1 (d, *J* = 21.9 Hz), 71.9, 26.8.

1-(4-chlorophenyl)-1-diazopropan-2-one (2h)

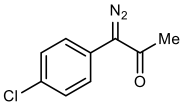

Orange solid, 1.7 g, 86% yield, TLC: R_f = 0.52 (PE/EA = 6:1, v/v).

^{1}H NMR (400 MHz, $CDCl_3$)

δ 7.47 – 7.41 (m, 2H), 7.38 – 7.33 (m, 2H), 2.36 (s, 3H).

^{13}C NMR (101 MHz, $CDCl_3$)

δ 189.5, 132.5, 129.1, 126.6, 124.0, 72.2, 26.9.

1-(4-bromophenyl)-1-diazopropan-2-one (2i)

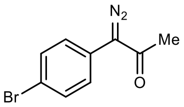

Orange solid, 2.1 g, 89% yield, TLC: R_f = 0.48 (PE/EA = 6:1, v/v).

^{1}H NMR (400 MHz, $CDCl_3$)

δ 7.53 – 7.48 (m, 2H), 7.43 – 7.34 (m, 2H), 2.37 (s, 3H).

^{13}C NMR (101 MHz, $CDCl_3$)

δ 189.5, 132.1, 126.9, 124.6, 120.5, 72.3, 27.0.

1-diazo-1-(4-iodophenyl)propan-2-one (2j)

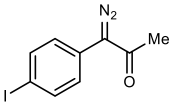

Orange solid, 2.6 g, 90% yield, TLC: R_f = 0.44 (PE/EA = 6:1, v/v).

^{1}H NMR (400 MHz, $CDCl_3$)

δ 7.73 – 7.68 (m, 2H), 7.29 – 7.23 (m, 2H), 2.37 (s, 3H).

^{13}C NMR (101 MHz, $CDCl_3$)

δ 189.4, 138.0, 127.0, 125.3, 91.7, 72.4, 27.0.

1-diazo-1-(naphthalen-2-yl)propan-2-one (2k)

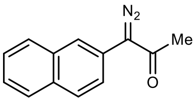

Red solid, 1.6 g, 76% yield, TLC: R_f = 0.40 (PE/EA = 6:1, v/v).

^{1}H NMR (400 MHz, $CDCl_3$)

δ 8.06 (s, 1H), 7.93 – 7.78 (m, 3H), 7.60 – 7.43 (m, 3H), 2.40 (s, 3H).

^{13}C NMR (101 MHz, $CDCl_3$)

δ 190.0, 133.6, 132.0, 128.7, 127.9, 127.7, 126.7, 126.2, 124.7, 123.2, 122.7, 73.1, 27.1.

1-([1,1'-biphenyl]-4-yl)-1-diazopropan-2-one (2l)

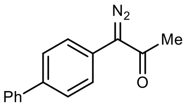

Red solid, 2.1 g, 87% yield, TLC: R_f = 0.51 (PE/EA = 6:1, v/v).

^{1}H NMR (400 MHz, $CDCl_3$)
δ 7.47 – 7.34 (m, 6H), 7.24 (t, J = 7.5 Hz, 2H), 7.15 (t, J = 7.4 Hz, 1H), 2.18 (s, 3H).

^{13}C NMR (101 MHz, $CDCl_3$)
δ 189.9, 140.2, 139.7, 128.9, 127.7, 127.6, 127.0, 126.1, 124.4, 72.7, 27.0.

1-diazo-1-phenylbutan-2-one (2m)

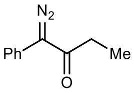

Red oil, 1.1 g, 62% yield, TLC: R_f = 0.66 (PE/EA = 6:1, v/v).

^{1}H NMR (400 MHz, $CDCl_3$)
δ 7.35 (d, J = 7.5 Hz, 2H), 7.24 (t, J = 7.8 Hz, 2H), 7.08 (t, J = 6.9 Hz, 1H), 2.46 (q, J = 7.4 Hz, 2H), 1.04 (t, J = 7.3 Hz, 3H).

^{13}C NMR (101 MHz, $CDCl_3$)
δ 193.5, 129.0, 126.9, 125.8, 125.7, 71.7, 32.4, 8.6.

Typical Procedure for Enantioselective N–H Insertion of α-Diazoketones with Amides and Analytical Data

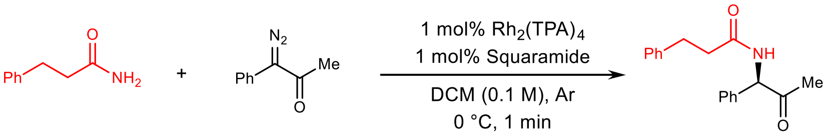

The $Rh_2(TPA)_4$·DCM (2.9 mg, 0.002 mmol, 1 mol%), chiral squaramide catalyst (1.3 mg, 0.002 mmol, 1 mol%) and 3-phenylpropionamide (30.0 mg, 0.2 mmol, 1.0 eq.) were introduced into an oven-dried Schlenk tube containing a stir bar in a argon-filled glove box. Then the Schlenk tube was sealed with a rubber, moved out of the glove box and injected with 1.5 mL DCM. The tube was cooled down to 0 °C and 0.5 mL of DCM solution of 1-diazo-1-phenylpropan-2-on (32.0 mg, 0.2 mmol) was added dropwise within 5 mins via a syringe while stirring . The resulting mixture was allowed to stir for another 1 min at 0 °C. Upon completion, organic volatiles was evaporated in vacuo, and the residue was purified by flash chromatography purification (PE/EA = 3:1, v/v).

(R)-N-(2-oxo-1-phenylpropyl)-3-phenylpropanamide (3a)

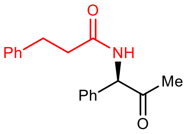

White solid, 56.5 mg, 99% yield, 92% ee, m. p. 90–92 °C, TLC: R_f = 0.50 (PE/EA = 1:1, v/v). HPLC condition: Chiralpak AD-H column (25 cm × 0.46 cm ID), hexane/2-propanol = 90:10, flow rate = 1.0 mL/min, 210 nm UV detector), t_R = 11.52 min (minor) and t_R = 21.05 min (major). $[\alpha]_D^{25}$ –354 (*c* 0.5, $CHCl_3$).

^{1}H NMR (400 MHz, $CDCl_3$)
δ 7.38 – 7.30 (m, 3H), 7.27 – 7.21 (m, 4H), 7.21 – 7.15 (m, 1H), 7.15 – 7.11 (m, 2H), 6.76 (d, *J* = 6.3 Hz, 1H), 5.52 (d, *J* = 6.3 Hz, 1H), 2.92 (t, *J* = 7.7 Hz, 2H), 2.60 – 2.45 (m, 2H), 2.08 (s, 3H).

^{13}C NMR (101 MHz, $CDCl_3$)
δ 203.5, 171.3, 140.7, 136.4, 129.2, 128.6, 128.5, 128.3, 128.0, 126.2, 63.5, 38.0, 31.4, 27.1.

HRMS (ESI)
Calcd for $[C_{18}H_{19}NO_2, M+H]^+$: 282.1489; Found: 282.1488.

(*R*)-N-(2-oxo-1-phenylpropyl)-2-phenylacetamide (3b)

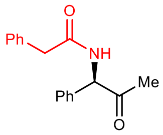

White solid, 53.4 mg, 99% yield, 91% ee, m. p. 94–96 °C, TLC: R_f = 0.60 (PE/EA = 1:1, v/v). HPLC condition: Chiralpak AD-H column (25 cm × 0.46 cm ID), hexane/2-propanol = 90:10, flow rate = 1.0 mL/min, 210 nm UV detector), t_R = 10.92 min (minor) and t_R = 18.00 min (major). $[\alpha]_D^{25}$ –361 (*c* 0.5, $CHCl_3$).

^{1}H NMR (400 MHz, $CDCl_3$)
δ 7.38 – 7.28 (m, 6H), 7.24 (m, 4H), 6.92 – 6.69 (d, *J* = 6.4 Hz, 1H), 5.50 (d, *J* = 6.4 Hz, 1H), 3.57 (s, 2H), 2.06 (s, 3H).

^{13}C NMR (101 MHz, $CDCl_3$)
δ 203.3, 170.2, 136.3, 134.6, 129.3, 129.2, 128.9, 128.6, 127.9, 127.3, 63.6, 43.5, 27.1.

HRMS (ESI)
Calcd for $[C_{17}H_{17}NO_2, M+Na]^+$: 290.1151; Found: 290.1156.

(*R*)-N-(2-oxo-1-phenylpropyl)acetamide (3c)

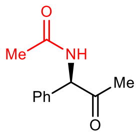

White solid, 38.2 mg, 99% yield, 90% ee, m. p. 100–102 °C, TLC: R_f = 0.30 (PE/EA = 1:1, v/v). HPLC condition: Chiralpak AD-H column (25 cm × 0.46 cm ID), hexane/2-propanol = 90:10, flow rate = 1.0 mL/min, 210 nm UV detector), t_R = 7.08 min (minor) and t_R = 8.57 min (major). $[\alpha]_D^{25}$ –272 (*c* 0.5, $CHCl_3$).

^{1}H NMR (400 MHz, $CDCl_3$)
δ 7.35 (m, 5H), 6.97 – 6.74 (d, J = 6.6 Hz, 1H), 5.56 (d, J = 6.6 Hz, 1H), 2.11 (s, 3H), 2.00 (s, 3H).

^{13}C NMR (101 MHz, $CDCl_3$)

δ 203.6, 169.3, 136. 5, 129.3, 128.6, 128.0, 63.5, 27.2, 23.1, 23.1.

HRMS (ESI)

Calcd for [$C_{11}H_{13}NO_2$, M+Na]$^+$: 214.0838; Found: 214.0839.

(*R*)-N-(2-oxo-1-phenylpropyl)propionamide (3d)

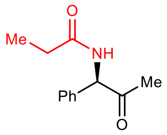

Colorless oil, 41.2 mg, 99% yield, 90% ee, TLC: R_f = 0.49 (PE/EA = 1:1, v/v). HPLC condition: Chiralpak AD-H column (25 cm × 0.46 cm ID), hexane/2-propanol = 90:10, flow rate = 1.0 mL/min, 230 nm UV detector), t_R = 6.91 min (minor) and t_R = 8.45 min (major). $[\alpha]_D^{25}$ –138 (*c* 0.5, $CHCl_3$).

^{1}H NMR (400 MHz, $CDCl_3$)

δ 7.40 – 7.30 (m, 5H), 6.80 (d, *J* = 6.4 Hz, 1H), 5.56 (d, *J* = 6.4 Hz, 1H), 2.25 (qd, *J* = 7.6, 4.4 Hz, 2H), 2.11 (s, 3H), 1.12 (t, *J* = 7.6 Hz, 3H).

^{13}C NMR (101 MHz, $CDCl_3$)

δ 203.7, 172.9, 136.6, 129.3, 128.6, 128.0, 63.4, 29.4, 27.2, 9.5.

HRMS (ESI)

Calcd for [$C_{12}H_{15}NO_2$, M+Na]$^+$: 228.0995; Found: 228.0996.

(*R*)-N-(2-oxo-1-phenylpropyl)butyramide (3e)

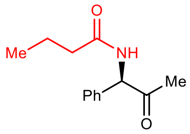

Colorless oil, 42.0 mg, 96% yield, 93% ee, TLC: R_f = 0.52 (PE/EA = 1:1, v/v). HPLC condition: Chiralpak AD-H column (25 cm × 0.46 cm ID), hexane/2-propanol = 90:10, flow rate = 1.0 mL/min, 210 nm UV detector), t_R = 6.95 min (minor) and t_R = 8.87 min (major). $[\alpha]_D^{25}$ –130 (*c* 0.5, $CHCl_3$).

^{1}H NMR (400 MHz, $CDCl_3$)

δ 7.41 – 7.31 (m, 5H), 6.84 (d, *J* = 6.4 Hz, 1H), 5.58 (d, *J* = 6.4 Hz, 1H), 2.21 (td, *J* = 7.3, 2.0 Hz, 2H), 2.12 (s, 3H), 1.64 (h, *J* = 7.4 Hz, 2H), 0.92 (t, *J* = 7.4 Hz, 3H).

^{13}C NMR (101 MHz, $CDCl_3$)

δ 203.7, 172.2, 136.6, 77.4, 77.1, 76.8, 63.4, 38.2, 27.2, 18.9, 13.7.

HRMS (ESI)

Calcd for [$C_{13}H_{17}NO_2$, M+Na]$^+$: 242.1151; Found: 242.1153.

(*R*)-N-(2-oxo-1-phenylpropyl)isobutyramide (3f)

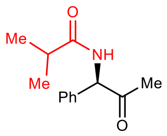

White solid, 38.1 mg, 87% yield, 91% ee, m. p. 108–110 °C, TLC: R_f = 0.58 (PE/EA = 1:1, v/v). HPLC condition: Chiralpak AD-H column (25 cm × 0.46 cm ID), hexane/2-propanol = 90:10, flow rate = 1.0 mL/min, 210 nm UV detector), t_R = 5.84 min (minor) and t_R = 8.27 min (major). $[\alpha]_D^{25}$ –288 (*c* 0.5, $CHCl_3$).

^{1}H NMR (400 MHz, $CDCl_3$)

δ 7.39 – 7.29 (m, 5H), 6.88 – 6.72 (d, *J* = 6.3 Hz, 1H), 5.53 (d, *J* = 6.3 Hz, 1H), 2.41 (h, *J* = 6.9 Hz, 1H), 2.11 (s, 3H), 1.15 (d, *J* = 6.9 Hz, 3H), 1.11 (d, *J* = 6.9 Hz, 3H).

^{13}C NMR (101 MHz, $CDCl_3$)

δ 203.7, 176.1, 136.6, 129.2, 128.5, 127.9, 63.4, 35.3, 27.2, 19.4.

HRMS (ESI)

Calcd for $[C_{13}H_{17}NO_2, M+Na]^+$: 242.1151; Found: 242.1151.

(*R*)-N-(2-oxo-1-phenylpropyl)pivalamide (3g)

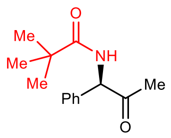

Colorless oil, 30.8 mg, 66% yield, 96% ee, TLC: R_f = 0.62 (PE/EA = 1:1, v/v). HPLC condition: Chiralpak AD-H column (25 cm × 0.46 cm ID), hexane/2-propanol = 90:10, flow rate = 1.0 mL/min, 210 nm UV detector), t_R = 5.16 min (minor) and t_R = 8.77 min (major). $[\alpha]_D^{25}$ –163 (*c* 0.5, $CHCl_3$).

^{1}H NMR (400 MHz, $CDCl_3$)

δ 7.40 – 7.29 (m, 5H), 7.03 (d, *J* = 6.0 Hz, 1H), 5.48 (d, *J* = 6.0 Hz, 1H), 2.11 (s, 3H), 1.20 (s, 9H).

^{13}C NMR (101 MHz, $CDCl_3$)

δ 203.8, 177.7, 136.7, 129.2, 128.5, 127.8, 63.6, 38.7, 29.7, 27.4.

HRMS (ESI)

Calcd for $[C_{14}H_{19}NO_2, M+Na]^+$: 256.1308; Found: 256.1308.

Methyl (*R*)-3-((2-oxo-1-phenylpropyl)carbamoyl)bicyclo[1.1.1]pentane-1-carboxylate (3h)

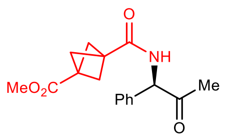

White solid, 60.1 mg, 99% yield, 96% ee, m. p. 120–122 °C, TLC: R_f = 0.52 (PE/EA = 1:1, v/v). HPLC condition: Chiralpak AD-H column (25 cm × 0.46 cm ID), hexane/2-propanol = 90:10, flow rate = 1.0 mL/min, 210 nm UV detector), t_R = 9.36 min (minor) and t_R = 12.04 min (major). $[\alpha]_D^{25}$ –251 (*c* 0.5, $CHCl_3$).

^{1}H NMR (600 MHz, $CDCl_3$)

δ 7.36 (m, 3H), 7.30 (m, 2H), 6.89 (d, *J* = 6.4 Hz, 1H), 5.50 (d, *J* = 6.4 Hz, 1H), 3.68 (s, 3H), 2.28 (s, 6H), 2.11 (s, 3H).

^{13}C NMR (151 MHz, $CDCl_3$)

δ 203.2, 169.7, 168.3, 136.1, 129.3, 128.8, 128.0, 63.1, 52.3, 51.8, 39.1, 36.8, 27.2.

HRMS (ESI)

Calcd for [$C_{17}H_{19}NO_4$, M+Na]$^+$: 324.1206; Found: 324.1211.

(*R*)-N-(2-oxo-1-phenylpropyl)cyclopropanecarboxamide (3i)

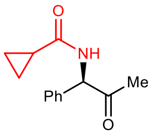

White solid, 36.9 mg, 85% yield, 90% ee, m. p. 102–104 °C, TLC: R_f = 0.55 (PE/EA = 1:1, v/v). HPLC condition: Chiralpak AD-H column (25 cm × 0.46 cm ID), hexane/2-propanol = 90:10, flow rate = 1.0 mL/min, 210 nm UV detector), t_R = 8.30 min (minor) and t_R = 9.89 min (major). $[\alpha]_D^{25}$ –135 (*c* 0.5, $CHCl_3$).

^{1}H NMR (400 MHz, $CDCl_3$)

δ 7.40 – 7.29 (m, 5H), 7.03 (d, *J* = 6.1 Hz, 1H), 5.57 (d, *J* = 6.3 Hz, 1H), 2.10 (s, 3H), 1.45 (m, 1H), 0.98 – 0.84 (m, 2H), 0.79 – 0.65 (m, 2H).

^{13}C NMR (101 MHz, $CDCl_3$)

δ 203.8, 172.9, 136.6, 129.2, 128.6, 128.0, 63.7, 27.2, 14.5, 7.5, 7.4.

HRMS (ESI)

Calcd for [$C_{13}H_{15}NO_2$, M+Na]$^+$: 240.0995; Found: 240.0995.

(*R*)-N-(2-oxo-1-phenylpropyl)cyclobutanecarboxamide (3j)

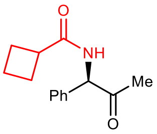

White solid, 46.3 mg, 99% yield, 94% ee, m. p. 116–118 °C, TLC: R_f = 0.60 (PE/EA = 1:1, v/v). HPLC condition: Chiralpak AD-H column (25 cm × 0.46 cm ID), hexane/2-propanol = 90:10, flow rate = 1.0 mL/min, 210 nm UV detector), t_R = 6.84 min (minor) and t_R = 9.92 min (major). $[\alpha]_D^{25}$ –423 (*c* 0.5, $CHCl_3$).

^{1}H NMR (400 MHz, $CDCl_3$)

δ 7.40 – 7.29 (m, 5H), 6.70 (d, *J* = 6.1 Hz, 1H), 5.54 (d, *J* = 6.4 Hz, 1H), 3.05 (pd, *J* = 8.5, 1.1 Hz, 1H), 2.33 – 2.06 (m, 7H), 1.95 (dq, *J* = 11.1, 8.8 Hz, 1H), 1.89 – 1.78 (m, 1H).

^{13}C NMR (101 MHz, $CDCl_3$)

δ 203.8, 174.2, 136.6, 129.2, 128.6, 127.9, 63.4, 39.6, 27.2, 25.2, 25.1, 18.2.

HRMS (ESI)

Calcd for [$C_{14}H_{17}NO_2$, M+Na]$^+$: 254.1151; Found: 254.1156.

(*R*)-N-(2-oxo-1-phenylpropyl)cyclohexanecarboxamide (3k)

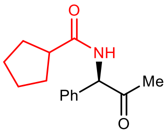

White solid, 49.6 mg, 99% yield, 91% ee, m. p. 115–117 °C, TLC: R_f = 0.68 (PE/EA = 1:1, v/v). HPLC condition: Chiralpak OJ-3 column (25 cm × 0.46 cm ID), hexane/2-propanol = 90:10, flow rate = 1.0 mL/min, 210 nm UV detector), t_R = 8.08 min (minor) and t_R = 9.19 min (major). $[\alpha]_D^{25}$ –200 (*c* 0.5, $CHCl_3$).

^{1}H NMR (400 MHz, $CDCl_3$)
δ 7.34 (m, 5H), 6.79 (d, *J* = 6.3 Hz, 1H), 5.54 (d, *J* = 6.3 Hz, 1H), 2.66 – 2.55 (m, 1H), 2.11 (s, 3H), 1.92 – 1.62 (m, 6H), 1.62 – 1.48 (m, 2H).

^{13}C NMR (101 MHz, $CDCl_3$)
δ 203.8, 175.5, 136.6, 129.2, 128.5, 127.9, 63.5, 45.4, 30.3, 27.2, 25.9, 25.9.

HRMS (ESI)
Calcd for [$C_{15}H_{19}NO_2$, M+Na]$^+$: 268.1308; Found: 268.1308.

(*R*)-N-(2-oxo-1-phenylpropyl)cyclohexanecarboxamide (3l)

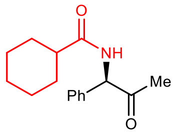

White solid, 52.7 mg, 99% yield, 96% ee, m. p. 160–162 °C, TLC: R_f = 0.60 (PE/EA = 1:1, v/v). HPLC condition: Chiralpak AD-H column (25 cm × 0.46 cm ID), hexane/2-propanol = 90:10, flow rate = 1.0 mL/min, 210 nm UV detector), t_R = 6.69 min (minor) and t_R = 10.10 min (major). $[\alpha]_D^{25}$ –181 (*c* 0.5, $CHCl_3$).

^{1}H NMR (400 MHz, $CDCl_3$)
δ 7.39 – 7.28 (m, 5H), 6.79 (d, *J* = 6.4 Hz, 1H), 5.53 (d, *J* = 6.3 Hz, 1H), 2.21 – 2.05 (m, 4H), 1.92 – 1.72 (m, 4H), 1.69 – 1.56 (m, 1H), 1.48 – 1.33 (m, 2H), 1.32 – 1.12 (m, 3H).

^{13}C NMR (101 MHz, $CDCl_3$)
δ 203.8, 175.2, 136.7, 129.2, 128.5, 127.9, 63.3, 45.1, 29.5, 29.4, 27.2, 25.7, 25.7, 25.6.

HRMS (ESI)
Calcd for [$C_{16}H_{21}NO_2$, M+H]$^+$: 260.1645; Found: 260.1645.

***tert*-butyl (*R*)-4-((2-oxo-1-phenylpropyl)carbamoyl)piperidine-1-carboxylate (3m)**

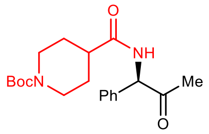

White solid, 69.1 mg, 96% yield, 88% ee, m. p. 123–125 °C, TLC: R_f = 0.35 (PE/EA = 1:1, v/v). HPLC condition: Chiralpak AD-H column (25 cm × 0.46 cm ID), hexane/2-propanol = 90:10, flow rate = 1.0 mL/min, 210 nm UV detector), t_R = 8.85 min (minor) and t_R = 9.60 min (major). $[\alpha]_D^{25}$ –329 (*c* 0.5, $CHCl_3$).

^{1}H NMR (400 MHz, $CDCl_3$)
δ 7.40 – 7.28 (m, 5H), 6.92 (d, *J* = 6.3 Hz, 1H), 5.53 (d, *J* = 6.3 Hz, 1H), 4.10 (s, 2H), 2.73 (s, 2H), 2.36 – 2.26 (m, 1H), 2.11 (s, 3H), 1.85 – 1.72 (m, 2H), 1.67 – 1.53 (m, 2H), 1.44 (s, 9H).

^{13}C NMR (101 MHz, $CDCl_3$)

δ 203.6, 173.6, 154.6, 136.4, 129.3, 128.7, 127.9, 79.6, 63.4, 42.9, 28.5, 28.4, 27.1.

HRMS (ESI)

Calcd for [$C_{20}H_{28}N_2O_4$, M+Na]$^+$: 383.1941; Found: 383.1940.

(*R*)-N-(2-oxo-1-phenylpropyl)tetrahydro-2H-pyran-4-carboxamide (3n)

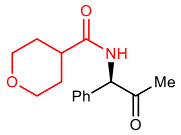

White solid, 52.5 mg, 99% yield, 94% ee, m. p. 128–130 °C, TLC: R_f = 0.20 (PE/EA = 1:1, v/v). HPLC condition: Chiralpak AD-H column (25 cm × 0.46 cm ID), hexane/2-propanol = 90:10, flow rate = 1.0 mL/min, 210 nm UV detector), t_R = 9.58 min (minor) and t_R = 11.72 min (major). $[\alpha]_D^{25}$ –4.8 (*c* 0.5, $CHCl_3$).

^{1}H NMR (400 MHz, $CDCl_3$)

δ 7.43 – 7.28 (m, 5H), 6.86 (d, *J* = 6.0 Hz, 1H), 5.53 (d, *J* = 6.3 Hz, 1H), 3.99 (ddt, *J* = 11.3, 7.3, 3.5 Hz, 2H), 3.47 – 3.32 (m, 2H), 2.40 (tt, *J* = 10.5, 5.4 Hz, 1H), 2.11 (s, 3H), 1.86 – 1.67 (m, 4H).

^{13}C NMR (101 MHz, $CDCl_3$)

δ 203.6, 173.4, 136.4, 129.3, 128.7, 127.9, 127.9, 67.2, 63.4, 41.8, 29.1, 27.2.

HRMS (ESI)

Calcd for [$C_{15}H_{19}NO_3$, M+Na]$^+$: 284.1257; Found: 284.1257.

(*R*)-N-(2-oxo-1-phenylpropyl)cycloheptanecarboxamide (3o)

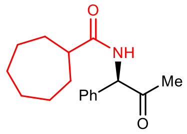

White solid, 55.0 mg, 99% yield, 96% ee, m. p. 143–145 °C, TLC: R_f = 0.50 (PE/EA = 1:1, v/v). HPLC condition: Chiralpak AD-H column (25 cm × 0.46 cm ID), hexane/2-propanol = 90:10, flow rate = 1.0 mL/min, 210 nm UV detector), t_R = 6.35 min (minor) and t_R = 9.49 min (major). $[\alpha]_D^{25}$ –187 (*c* 0.5, $CHCl_3$).

^{1}H NMR (400 MHz, $CDCl_3$)

δ 7.33 – 7.21 (m, 5H), 6.67 (d, *J* = 6.3 Hz, 1H), 5.45 (d, *J* = 6.1 Hz, 1H), 2.22 (m, 1H), 2.03 (s, 3H), 1.86 – 1.58 (m, 5H), 1.57 – 1.31 (m, 7H).

^{13}C NMR (101 MHz, $CDCl_3$)

δ 203.8, 176.4, 136.3, 129.2, 128.5, 127.9, 63.4, 47.0, 31.5, 31.4, 28.2, 28.1, 27.2, 26.6, 26.5.

HRMS (ESI)

Calcd for [$C_{17}H_{23}NO_2$, M+Na]$^+$: 296.1621; Found: 296.1621.

(*R*)-N-(2-oxo-1-phenylpropyl)benzamide (3p)

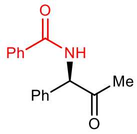

White solid, 47.2 mg, 93% yield, 92% ee, m. p. 100–102 °C, TLC: R_f = 0.61 (PE/EA = 3:1, v/v). HPLC condition: Chiralpak AD-H column (25 cm × 0.46 cm ID), hexane/2-propanol = 90:10, flow rate = 1.0 mL/min, 230 nm UV detector), t_R = 14.53 min (minor) and t_R = 31.41 min (major). $[\alpha]_D^{25}$ –115 (*c* 0.5, $CHCl_3$).

^{1}H NMR (400 MHz, $CDCl_3$)
δ 7.88 – 7.81 (m, 2H), 7.62 (d, *J* = 6.2 Hz, 1H), 7.56 – 7.50 (m, 1H), 7.49 – 7.33 (m, 7H), 5.76 (d, *J* = 6.1 Hz, 1H), 2.20 (s, 3H).

^{13}C NMR (101 MHz, $CDCl_3$)
δ 203.6, 166.3, 136.5, 133.8, 131.8, 129.3, 128.7, 128.6, 128.1, 127.2, 63.9, 27.2.

HRMS (ESI)
Calcd for [$C_{16}H_{15}NO_2$, M+H]$^+$: 254.1176; Found: 254.1176.

(*R*)-4-methoxy-N-(2-oxo-1-phenylpropyl)benzamide (3q)

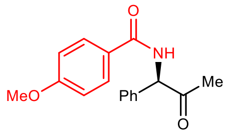

White solid, 57.0 mg, 99% yield, 94% ee, m. p. 95–97 °C, TLC: R_f = 0.55 (PE/EA = 1:1, v/v). HPLC condition: Chiralpak AD-H column (25 cm × 0.46 cm ID), hexane/2-propanol = 90:10, flow rate = 1.0 mL/min, 254 nm UV detector), t_R = 39.10 min (minor) and t_R = 95.04 min (major). $[\alpha]_D^{25}$ –188 (*c* 0.5, $CHCl_3$).

^{1}H NMR (400 MHz, $CDCl_3$)
δ 7.82 – 7.76 (m, 2H), 7.50 (d, *J* = 6.1 Hz, 1H), 7.43 – 7.30 (m, 5H), 6.94 – 6.88 (m, 2H), 5.71 (d, *J* = 6.1 Hz, 1H), 3.83 (s, 3H), 2.16 (s, 3H).

^{13}C NMR (101 MHz, $CDCl_3$)
δ 203.8, 165.9, 162.4, 136.7, 129.3, 129.0, 128.6, 128.1, 126.0, 113.7, 63.9, 55.4, 27.2.

HRMS (ESI)
Calcd for [$C_{17}H_{17}NO_3$, M+Na]$^+$: 306.1101; Found: 306.1106.

(*R*)-N-(2-oxo-1-phenylpropyl)-4-(trifluoromethyl)benzamide (3r)

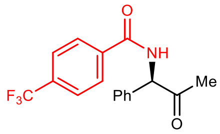

White solid, 64.3 mg, 99% yield, 95% ee, m. p. 110–112 °C, TLC: R_f = 0.75 (PE/EA = 1:1, v/v). HPLC condition: Chiralpak AD-H column (25 cm × 0.46 cm ID), hexane/2-propanol = 90:10, flow rate = 1.0 mL/min, 230 nm UV detector), t_R = 15.34 min (minor) and t_R = 24.62 min (major). $[\alpha]_D^{25}$ –165 (*c* 0.5, $CHCl_3$).

^{1}H NMR (400 MHz, $CDCl_3$)

δ 7.91 (d, *J* = 8.1 Hz, 2H), 7.68 (d, *J* = 7.9 Hz, 3H), 7.44 – 7.31 (m, 5H), 5.71 (d, *J* = 6.1 Hz, 1H), 2.18 (s, 3H).

^{13}C NMR (101 MHz, $CDCl_3$)

δ 203.3, 165.1, 137.0, 136.1, 133.4 (q, *J* = 32.5 Hz), 129.4, 128.9, 128.1, 127.6, 125.6 (q, *J* = 3.9 Hz), 123.6 (q, *J* = 272.5 Hz), 64.0, 27.1.

^{19}F NMR (376 MHz, $CDCl_3$)

δ –63.0.

HRMS (ESI)

Calcd for [$C_{17}H_{14}F_3NO_2$, M+Na]$^+$: 344.0869; Found: 344.0874.

(*R*)-4-fluoro-N-(2-oxo-1-phenylpropyl)benzamide (3s)

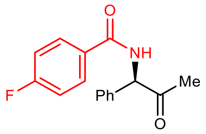

White solid, 54.9 mg, 99% yield, 96% ee, m. p. 83–85 °C, TLC: R_f = 0.60 (PE/EA = 1:1, v/v). HPLC condition: Chiralpak AD-H column (25 cm × 0.46 cm ID), hexane/2-propanol = 90:10, flow rate = 1.0 mL/min, 230 nm UV detector), t_R = 13.56 min (minor) and t_R = 20.75 min (major). $[\alpha]_D^{25}$ –137 (*c* 0.5, $CHCl_3$).

^{1}H NMR (400 MHz, $CDCl_3$)

δ 7.72 – 7.63 (m, 2H), 7.46 (d, *J* = 6.1 Hz, 1H), 7.30 – 7.16 (m, 5H), 6.93 (td, *J* = 8.7, 2.0 Hz, 2H), 5.57 (d, *J* = 6.1 Hz, 1H), 2.01 (s, 3H).

^{13}C NMR (101 MHz, $CDCl_3$)

δ 203.6, 166.1, 165.3, 163.6, 136.4, 130.0 (d, *J* = 3.2 Hz), 129.5 (d, *J* = 8.9 Hz), 129.3, 128.7, 128.1, 115.6 (d, *J* = 21.9 Hz). 64.0, 27.1.

^{19}F NMR (376 MHz, $CDCl_3$)

δ –108.

HRMS (ESI)

Calcd for [$C_{16}H_{14}FNO_2$, M+Na]$^+$: 294.0901; Found: 294.0904.

(*R*)-4-chloro-N-(2-oxo-1-phenylpropyl)benzamide (3t)

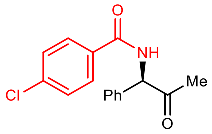

White solid, 57.7 mg, 99% yield, 96% ee, m. p. 108–110 °C, TLC: R_f = 0.63 (PE/EA = 1:1, v/v). HPLC condition: Chiralpak AD-H column (25 cm × 0.46 cm ID), hexane/2-propanol = 90:10, flow rate = 1.0 mL/min, 230 nm UV detector), t_R = 18.13 min (minor) and t_R = 34.02 min (major). $[\alpha]_D^{25}$ –98.2 (*c* 0.5, $CHCl_3$).

^{1}H NMR (600 MHz, $CDCl_3$)

δ 7.74 (d, *J* = 8.2 Hz, 2H), 7.57 (d, *J* = 6.0 Hz, 1H), 7.44 – 7.31 (m, 7H), 5.69 (d, *J* = 6.1 Hz, 1H), 2.16 (s, 3H).

^{13}C NMR (151 MHz, $CDCl_3$)

δ 203.5, 165.3, 138.0, 136.3, 132.1, 129.4, 128.8, 128.8, 128.6, 128.1, 63.97, 27.2.

HRMS (ESI)

Calcd for [$C_{16}H_{14}ClNO_2$, M+Na]$^+$: 310.0605; Found: 310.0605.

(*R*)-4-bromo-N-(2-oxo-1-phenylpropyl)benzamide (3u)

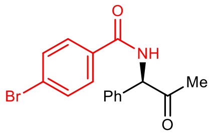

White solid, 63.9 mg, 96% yield, 96% ee, m. p. 113–115 °C, TLC: R_f = 0.85 (PE/EA = 1:1, v/v). HPLC condition: Chiralpak AD-H column (25 cm × 0.46 cm ID), hexane/2-propanol = 90:10, flow rate = 1.0 mL/min, 254 nm UV detector), t_R = 22.56 min (minor) and t_R = 44.80 min (major). $[\alpha]_D^{25}$ –140.0 (*c* 0.5, $CHCl_3$).

^{1}H NMR (600 MHz, $CDCl_3$)

δ 7.68 (d, *J* = 8.2 Hz, 2H), 7.55 (d, *J* = 8.1 Hz, 3H), 7.44 – 7.32 (m, 5H), 5.69 (d, *J* = 6.0 Hz, 1H), 2.16 (s, 3H).

^{13}C NMR (151 MHz, $CDCl_3$)

δ 203.4, 165.4, 136.3, 132.6, 131.8, 129.3, 128.8, 128.8, 128.1, 126.5, 64.0, 27.1.

HRMS (ESI)

Calcd for [$C_{16}H_{14}BrNO_2$, M+H]$^+$: 332.0281; Found: 332.0278.

(*R*)-4-iodo-N-(2-oxo-1-phenylpropyl)benzamide (3v)

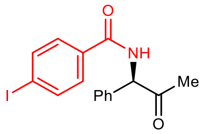

White solid, 75.8 mg, 99% yield, 96% ee, m. p. 130–132 °C, TLC: R_f = 0.86 (PE/EA = 1:1, v/v). HPLC condition: Chiralpak OJ-3 column (25 cm × 0.46 cm ID), hexane/2-propanol = 90:10, flow rate = 1.0 mL/min, 254 nm UV detector), t_R = 12.02 min (major) and t_R = 16.73 min (minor). $[\alpha]_D^{25}$ –90.7 (*c* 0.5, $CHCl_3$).

^{1}H NMR (600 MHz, $CDCl_3$)

δ 7.76 (d, *J* = 8.1 Hz, 2H), 7.57 (d, *J* = 6.1 Hz, 1H), 7.52 (d, *J* = 8.1 Hz, 2H), 7.42 – 7.31 (m, 5H), 5.68 (d, *J* = 6.1 Hz, 1H), 2.16 (s, 3H).

^{13}C NMR (151 MHz, $CDCl_3$)

δ 203.5, 165.6, 137.8, 136.3, 133.2, 129.4, 128.8, 128.8, 128.1, 98.9, 64.0, 27.2.

HRMS (ESI)

Calcd for [$C_{16}H_{14}INO_2$, M+Na]$^+$: 401.9961; Found: 401.9961.

(*R*)-N-(2-oxo-1-phenylpropyl)-2-naphthamide (3w)

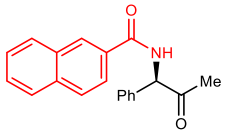

White solid, 54.5 mg, 90% yield, 97% ee, m. p. 134–136 °C, TLC: R_f = 0.62 (PE/EA = 1:1, v/v). HPLC condition: Chiralpak OJ-3 column (25 cm × 0.46 cm ID), hexane/2-propanol = 80:20, flow rate = 1.0 mL/min, 230 nm UV detector), t_R = 18.86 min (major) and t_R = 24.37 min (minor). $[\alpha]_D^{25}$ –230 (*c* 0.5, $CHCl_3$).

^{1}H NMR (600 MHz, $CDCl_3$)
δ 8.33 (s, 1H), 7.91 (d, *J* = 7.9 Hz, 1H), 7.85 (m, 3H), 7.74 (d, *J* = 6.1 Hz, 1H), 7.53 (p, *J* = 7.0 Hz, 2H), 7.45 (d, *J* = 7.6 Hz, 2H), 7.40 (t, *J* = 7.5 Hz, 2H), 7.34 (t, *J* = 7.3 Hz, 1H), 5.78 (d, *J* = 6.1 Hz, 1H), 2.19 (s, 3H).

^{13}C NMR (151 MHz, $CDCl_3$)
δ 203.7, 166.4, 136.6, 134.9, 132.6, 131.0, 129.3, 129.0, 128.7, 128.5, 128.1, 127.8, 127.8, 126.8, 123.7, 64.1, 27.2.

HRMS (ESI)
Calcd for $[C_{20}H_{17}NO_2, M+Na]^+$: 326.1151; Found: 326.1151.

(*R*)-N-(2-oxo-1-phenylpropyl)furan-2-carboxamide (3x)

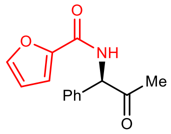

White solid, 49.0 mg, 99% yield, 94% ee, m. p. 58-60 °C, TLC: R_f = 0.65 (PE/EA = 1:1, v/v). HPLC condition: Chiralpak AD-H column (25 cm × 0.46 cm ID), hexane/2-propanol = 90:10, flow rate = 1.0 mL/min, 230 nm UV detector), t_R = 13.00 min (minor) and t_R = 28.61 min (major). $[\alpha]_D^{25}$ -153.3 (*c* 0.5, $CHCl_3$).

^{1}H NMR (400 MHz, $CDCl_3$)
δ 7.67 (d, *J* = 6.5 Hz, 1H), 7.46 (dd, *J* = 1.7, 0.8 Hz, 1H), 7.42 – 7.31 (m, 5H), 7.07 (dd, *J* = 3.5, 0.8 Hz, 1H), 6.47 (dd, *J* = 3.5, 1.8 Hz, 1H), 5.69 (d, *J* = 6.5 Hz, 1H), 2.16 (s, 3H).

^{13}C NMR (101 MHz, $CDCl_3$)
δ 203.1, 157.4, 147.5, 144.2, 136.3, 129.3, 128.7, 128.1, 114.7, 112.1, 63.2, 27.2.

HRMS (ESI)
Calcd for $[C_{14}H_{13}NO_3, M+H]^+$: 244.0968; Found: 244.0973.

(*R*)-N-(2-oxo-1-phenylpropyl)thiophene-2-carboxamide (3y)

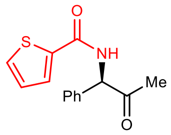

White solid, 52.2 mg, 99% yield, 95% ee, m. p. 96-98 °C, TLC: R_f = 0.70 (PE/EA = 1:1, v/v). HPLC condition: Chiralpak AD-H column (25 cm × 0.46 cm ID), hexane/2-

propanol = 90:10, flow rate = 1.0 mL/min, 230 nm UV detector), t_R = 13.48 min (minor) and t_R = 24.45 min (major). $[\alpha]_D^{25}$ -227.0 (*c* 0.5, $CHCl_3$).

^{1}H NMR (600 MHz, $CDCl_3$)
δ 7.57 (d, *J* = 3.7 Hz, 1H), 7.47 (d, *J* = 4.9 Hz, 1H), 7.42 (d, *J* = 6.0 Hz, 1H), 7.38 (m, 4H), 7.34 (q, *J* = 5.0, 4.4 Hz, 1H), 7.06 (s, 1H), 5.68 (d, *J* = 6.0 Hz, 1H), 2.16 (s, 3H).

^{13}C NMR (151 MHz, $CDCl_3$)
δ 203.4, 161.0, 138.3, 136.3, 130.4, 129.3, 128.8, 128.6, 128.1, 127.7, 63.8, 27.2.

HRMS (ESI)
Calcd for [$C_{14}H_{13}NO_2S$, M+Na]$^+$: 282.0559; Found: 282.0562.

(*R*)-N-(2-oxo-1-phenylpropyl)picolinamide (3z)

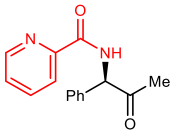

White solid, 45.7 mg, 90% yield, 96% ee, m. p. 86-88 °C, TLC: R_f = 0.55 (PE/EA = 1:1, v/v). HPLC condition: Chiralpak AD-H column (25 cm × 0.46 cm ID), hexane/2-propanol = 90:10, flow rate = 1.0 mL/min, 230 nm UV detector), t_R = 17.10 min (minor) and t_R = 44.51 min (major). $[\alpha]_D^{25}$ -226.0 (*c* 0.5, $CHCl_3$).

^{1}H NMR (400 MHz, $CDCl_3$)
δ 9.18 (d, *J* = 6.9 Hz, 1H), 8.59 (s, 1H), 8.11 (d, *J* = 7.8 Hz, 1H), 7.81 (tt, *J* = 7.8, 2.2 Hz, 1H), 7.48 – 7.31 (m, 6H), 5.73 (d, *J* = 7.1 Hz, 1H), 2.19 (s, 3H).

^{13}C NMR (101 MHz, $CDCl_3$)
δ 203.0, 163.7, 149.5, 148.3, 137.2, 136.3, 129.3, 128.7, 128.1, 126.3, 122.2, 63.7, 27.2.

HRMS (ESI)
Calcd for [$C_{15}H_{14}N_2O_2$, M+Na]$^+$: 277.0947; Found: 277.0951.

(*R*)-N-(2-oxo-1-phenylpropyl)-1H-indole-2-carboxamide (3aa)

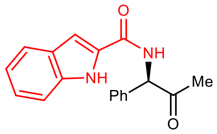

White solid, 47.3 mg, 81% yield, 97% ee, m. p. 115-117 °C, TLC: R_f = 0.72 (PE/EA = 1:1, v/v). HPLC condition: Chiralpak OJ-3 column (25 cm × 0.46 cm ID), hexane/2-propanol = 80:20, flow rate = 1.0 mL/min, 230 nm UV detector), t_R = 17.00 min (major) and t_R = 27.76 min (minor). $[\alpha]_D^{25}$ -171.5 (*c* 0.5, $CHCl_3$).

^{1}H NMR (600 MHz, $CDCl_3$)
δ 9.90 (s, 1H), 7.72 (d, *J* = 5.9 Hz, 1H), 7.63 (d, *J* = 8.0 Hz, 1H), 7.40 (m, 5H), 7.19 (t, *J* = 7.6 Hz, 1H), 7.13 – 7.08 (m, 2H), 7.02 (d, *J* = 2.0 Hz, 1H), 5.72 (d, *J* = 5.8 Hz, 1H), 2.17 (s, 3H).

^{13}C NMR (151 MHz, $CDCl_3$)

δ 203.1, 161.1, 136.6, 136.5, 130.0, 129.4, 128.8, 128.0, 127.5, 124.5, 121.9, 120.5, 112.1, 103.1, 63.8, 27.0.

HRMS (ESI)

Calcd for [$C_{18}H_{16}N_2O_2$, M+Na]$^+$: 315.1104; Found: 315.1110.

(*R*)-N-(2-oxo-1-phenylpropyl)acrylamide (3ab)

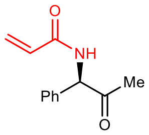

White solid, 34.5 mg, 85% yield, 90% ee, m. p. 100-102 °C, TLC: R_f = 0.52 (PE/EA = 1:1, v/v). HPLC condition: Chiralpak AD-H column (25 cm × 0.46 cm ID), hexane/2-propanol = 90:10, flow rate = 1.0 mL/min, 210 nm UV detector), t_R = 8.19 min (minor) and t_R = 10.84 min (major). $[\alpha]_D^{25}$ -356.5 (*c* 0.5, $CHCl_3$).

^{1}H NMR (400 MHz, $CDCl_3$)

δ7.44 – 7.30 (m, 5H), 7.10 – 6.92 (m, 1H), 6.29 (dd, *J* = 17.0, 1.7 Hz, 1H), 6.18 (dd, *J* = 17.0, 10.0 Hz, 1H), 5.71 – 5.61 (m, 2H), 2.15 (s, 3H).

^{13}C NMR (101 MHz, $CDCl_3$)

δ 203.4, 164.5, 136.3, 130.3, 129.3, 128.7, 128.0, 127.2, 63.5, 27.2.

HRMS (ESI)

Calcd for [$C_{12}H_{13}NO_2$, M+Na]$^+$: 226.0838; Found: 226.0839.

(*R*)-N-(2-oxo-1-phenylpropyl)-3-(trimethylsilyl)propiolamide (3ac)

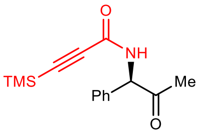

White solid, 55.0 mg, 99% yield, 85% ee, m. p. 96-98 °C, TLC: R_f = 0.80 (PE/EA = 1:1, v/v). HPLC condition: Chiralpak AD-H column (25 cm × 0.46 cm ID), hexane/2-propanol = 90:10, flow rate = 1.0 mL/min, 230 nm UV detector), t_R = 4.72 min (minor) and t_R = 6.06 min (major). $[\alpha]_D^{25}$ -173.5 (*c* 0.5, $CHCl_3$).

^{1}H NMR (400 MHz, $CDCl_3$)

δ 7.33 – 7.20 (m, 5H), 7.09 (d, *J* = 6.5 Hz, 1H), 5.48 (d, *J* = 6.5 Hz, 1H), 2.03 (s, 3H), 0.13 (s, 9H).

^{13}C NMR (101 MHz, $CDCl_3$)

δ 203.2, 152.4, 136.4, 130.0, 129.6, 128.8, 97.9, 93.0, 64.3, 27.8, 0.0.

HRMS (ESI)

Calcd for [$C_{15}H_{19}NO_2Si$, M+Na]$^+$: 296.1077; Found: 296.1081.

(*R*)-N-(2-oxo-1-(*p*-tolyl)propyl)butyramide (4a)

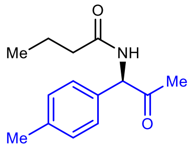

Colorless oil, 46.6 mg, 99% yield, 92% ee, TLC: R_f = 0.69 (PE/EA = 1:1, v/v). HPLC condition: Chiralpak AD-H column (25 cm × 0.46 cm ID), hexane/2-propanol = 90:10, flow rate = 1.0 mL/min, 230 nm UV detector), t_R = 6.64 min (minor) and t_R = 9.25 min (major). $[\alpha]_D^{25}$ -115.8 (*c* 0.5, $CHCl_3$).

^{1}H NMR (600 MHz, $CDCl_3$)

δ 7.18 (q, *J* = 7.8 Hz, 4H), 6.76 (d, *J* = 6.5 Hz, 1H), 5.52 (d, *J* = 6.4 Hz, 1H), 2.33 (s, 3H), 2.18 (td, *J* = 7.4, 4.5 Hz, 2H), 2.10 (s, 3H), 1.63 (h, *J* = 7.4 Hz, 2H), 0.90 (t, *J* = 7.4 Hz, 3H).

^{13}C NMR (151 MHz, $CDCl_3$)

δ 203.9, 172.2, 138.5, 133.6, 129.9, 127.9, 63.1, 38.3, 27.1, 21.2, 18.9, 13.7.

HRMS (ESI)

Calcd for $[C_{14}H_{19}NO_2, M+Na]^+$: 256.1308; Found: 256.1307.

(*R*)-N-(2-oxo-1-(*m*-tolyl)propyl)butyramide (4b)

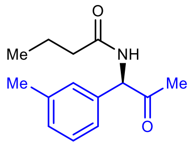

Colorless oil, 47.0 mg, 99% yield, 92% ee, TLC: R_f = 0.70 (PE/EA = 1:1, v/v). HPLC condition: Chiralpak AD-H column (25 cm × 0.46 cm ID), hexane/2-propanol = 90:10, flow rate = 1.0 mL/min, 230 nm UV detector), t_R = 5.94 min (minor) and t_R = 7.28 min (major). $[\alpha]_D^{25}$ -513.7 (*c* 0.5, $CHCl_3$).

^{1}H NMR (600 MHz, $CDCl_3$)

δ 7.24 (t, *J* = 7.5 Hz, 1H), 7.16 – 7.07 (m, 3H), 6.77 (d, *J* = 6.7 Hz, 1H), 5.52 (d, *J* = 6.5 Hz, 1H), 2.34 (s, 3H), 2.19 (td, *J* = 7.4, 3.4 Hz, 2H), 2.11 (s, 3H), 1.67 – 1.59 (m, 2H), 0.91 (t, *J* = 7.4 Hz, 3H).

^{13}C NMR (151 MHz, $CDCl_3$)

δ 203.9, 172.2, 139.0, 136.4, 129.4, 129.1, 128.6, 125.0, 77.3, 77.1, 76.9, 63.4, 38.3, 27.2, 21.4, 18.9, 13.7.

HRMS (ESI)

Calcd for $[C_{14}H_{19}NO_2, M+Na]^+$: 256.1308; Found: 256.1306.

(*R*)-N-(2-oxo-1-(4-(trifluoromethyl)phenyl)propyl)butyramide (4c)

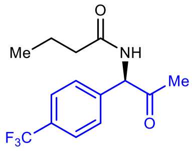

White solid, 57.5 mg, 99% yield, 80% ee, m. p. 106-108 °C, TLC: R_f = 0.71 (PE/EA = 1:1, v/v). HPLC condition: Chiralpak AD-H column (25 cm × 0.46 cm ID), hexane/2-propanol = 90:10, flow rate = 1.0 mL/min, 230 nm UV detector), t_R = 5.55 min (major) and t_R = 13.40 min (minor). $[\alpha]_D^{25}$ -194.4 (*c* 0.5, $CHCl_3$).

^{1}H NMR (600 MHz, $CDCl_3$)
δ 7.64 (d, *J* = 8.0 Hz, 2H), 7.46 (d, *J* = 8.0 Hz, 2H), 6.94 (d, *J* = 5.9 Hz, 1H), 5.59 (d, *J* = 6.0 Hz, 1H), 2.21 (td, *J* = 7.4, 4.7 Hz, 2H), 2.12 (s, 3H), 1.63 (h, *J* = 7.3 Hz, 2H), 0.90 (t, *J* = 7.4 Hz, 3H).

^{13}C NMR (151 MHz, $CDCl_3$)
δ 202.7, 172.3, 140.7, 130.8 (q, *J* = 32.5 Hz), 128.3, 126.2 (q, *J* = 4.0, 3.5 Hz), 123.8 (q, *J* = 272.5 Hz), 63.2, 38.2, 27.2, 18.9, 13.7.

^{19}F NMR (376 MHz, $CDCl_3$)
δ −62.8.

HRMS (ESI)
Calcd for $[C_{14}H_{16}F_3NO_2$, M+Na$]^+$: 310.1025; Found: 310.1026.

(*R*)-N-(1-(3-methoxyphenyl)-2-oxopropyl)butyramide (4d)

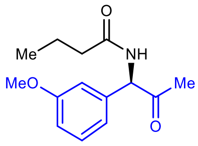

Colorless oil, 50.5 mg, 99% yield, 86% ee, TLC: R_f = 0.60 (PE/EA = 1:1, v/v). HPLC condition: Chiralpak AD-H column (25 cm × 0.46 cm ID), hexane/2-propanol = 90:10, flow rate = 1.0 mL/min, 254 nm UV detector), t_R = 8.77 min (minor) and t_R = 9.72 min (major). $[\alpha]_D^{25}$ -478.2 (*c* 0.5, $CHCl_3$).

^{1}H NMR (400 MHz, $CDCl_3$)
δ 7.30 – 7.25 (m, 1H), 6.92 – 6.84 (m, 3H), 6.80 (d, *J* = 6.4 Hz, 1H), 5.53 (d, *J* = 6.4 Hz, 1H), 3.80 (s, 3H), 2.20 (td, *J* = 7.2, 1.5 Hz, 2H), 2.12 (s, 3H), 1.64 (h, *J* = 7.4 Hz, 2H), 0.91 (t, *J* = 7.4 Hz, 3H).

^{13}C NMR (101 MHz, $CDCl_3$)
δ 203.6, 172.2, 160.1, 138.0, 130.3, 120.2, 114.0, 113.5, 63.3, 55.3, 38.3, 27.1, 19.0, 13.7.

HRMS (ESI)
Calcd for $[C_{14}H_{19}NO_3$, M+H$]^+$: 250.1438; Found: 250.1436.

(*R*)-N-(1-(2-fluorophenyl)-2-oxopropyl)butyramide (4e)

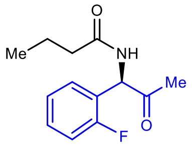

Colorless oil, 43.1 mg, 91% yield, 93% ee, TLC: R_f = 0.55 (PE/EA = 2:1, v/v). HPLC condition: Chiralpak AD-H column (25 cm × 0.46 cm ID), hexane/2-propanol = 90:10, flow rate = 1.0 mL/min, 254 nm UV detector), t_R = 8.62 min (minor) and t_R = 9.34 min (major). $[\alpha]_D^{25}$ -352.3 (*c* 0.5, $CHCl_3$).

^1H NMR (400 MHz, $CDCl_3$)

δ 7.40 – 7.30 (m, 2H), 7.20 – 7.06 (m, 2H), 6.87 (d, *J* = 6.5 Hz, 1H), 5.79 (d, *J* = 6.6 Hz, 1H), 2.23 (td, *J* = 7.3, 2.1 Hz, 2H), 2.14 (s, 3H), 1.65 (h, *J* = 7.4 Hz, 2H), 0.92 (t, *J* = 7.4 Hz, 3H).

^{13}C NMR (101 MHz, $CDCl_3$)

δ 202.47, 172.27, 161.82, 159.36, 130.4 (d, *J* = 8.2 Hz), 130.0 (d, *J* = 3.6 Hz), 129.95, 129.92, 124.86, 124.82, 124.15 (d, *J* = 14.1 Hz), 116.09 (d, *J* = 21.6 Hz). 58.13, 38.19, 26.69, 18.90, 13.65.

HRMS (ESI)

Calcd for $[C_{14}H_{19}NO_3, M+Na]^+$: 260.1057; Found: 260.1057.

(*R*)-N-(1-(4-fluorophenyl)-2-oxopropyl)butyramide (4f)

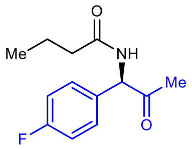

White solid, 47.5 mg, 99% yield, 74% ee, m. p. 52-54 °C, TLC: R_f = 0.65 (PE/EA = 1:1, v/v). HPLC condition: Chiralpak AD-H column (25 cm × 0.46 cm ID), hexane/2-propanol = 90:10, flow rate = 1.0 mL/min, 230 nm UV detector), t_R = 6.57 min (minor) and t_R = 9.50 min (major). $[\alpha]_D^{25}$ -223.4 (*c* 0.5, $CHCl_3$).

^1H NMR (400 MHz, $CDCl_3$)

δ 7.27 – 7.18 (t, *J* = 8.4 Hz, 2H), 6.99 (t, *J* = 8.4 Hz, 2H), 6.86 – 6.70 (d, *J* = 6.2 Hz, 1H), 5.47 (d, *J* = 6.2 Hz, 1H), 2.12 (t, *J* = 7.7 Hz, 2H), 2.04 (s, 3H), 1.56 (h, *J* = 7.3 Hz, 2H), 0.83 (t, *J* = 7.4 Hz, 3H).

^{13}C NMR (101 MHz, $CDCl_3$)

δ 203.5, 172.3, 162.7 (d, *J* = 247.9 Hz), 132.5 (d, *J* = 3.2 Hz), 129.7 (d, *J* = 8.2 Hz), 116.2 (d, *J* = 21.8 Hz), 62.7, 38.2, 27.1, 18.9, 13.7.

^{19}F NMR (376 MHz, $CDCl_3$)

δ −113.0.

HRMS (ESI)

Calcd for $[C_{13}H_{16}FNO_2, M+Na]^+$: 260.1057; Found: 260.1058.

(*R*)-N-(1-(4-chlorophenyl)-2-oxopropyl)butyramide (4g)

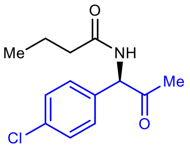

White solid, 50.6 mg, 99% yield, 83% ee, m. p. 74-76 °C, TLC: R_f = 0.66 (PE/EA = 1:1, v/v). HPLC condition: Chiralpak AD-H column (25 cm × 0.46 cm ID), hexane/2-propanol = 90:10, flow rate = 1.0 mL/min, 230 nm UV detector), t_R = 6.76 min (minor) and t_R = 11.79 min (major). $[\alpha]_D^{25}$ -224.2 (*c* 0.5, $CHCl_3$).

^{1}H NMR (600 MHz, $CDCl_3$)

δ 7.34 (d, *J* = 8.1 Hz, 2H), 7.26 (d, *J* = 8.2 Hz, 2H), 6.86 (d, *J* = 6.3 Hz, 1H), 5.51 (d, *J* = 6.2 Hz, 1H), 2.19 (td, *J* = 7.4, 4.6 Hz, 2H), 2.11 (s, 3H), 1.62 (h, *J* = 7.3 Hz, 2H), 0.90 (s, 3H).

^{13}C NMR (151 MHz, $CDCl_3$)

δ 203.1, 172.2, 135.3, 134.6, 129.4, 129.3, 62.8, 38.2, 27.1, 18.9, 13.7.

HRMS (ESI)

Calcd for $[C_{13}H_{16}ClNO_2, M+Na]^+$: 276.0762; Found: 276.0760.

(*R*)-N-(1-(4-bromophenyl)-2-oxopropyl)butyramide (4h)

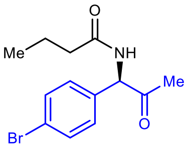

White solid, 59.9 mg, 99% yield, 85% ee, m. p. 75-77 °C, TLC: R_f = 0.66 (PE/EA = 1:1, v/v). HPLC condition: Chiralpak AD-H column (25 cm × 0.46 cm ID), hexane/2-propanol = 90:10, flow rate = 1.0 mL/min, 230 nm UV detector), t_R = 7.05 min (minor) and t_R = 13.62 min (major). $[\alpha]_D^{25}$ -233.9 (*c* 0.5, $CHCl_3$).

^{1}H NMR (600 MHz, $CDCl_3$)

δ 7.50 (d, *J* = 8.0 Hz, 2H), 7.20 (d, *J* = 8.1 Hz, 2H), 6.88 (d, *J* = 6.1 Hz, 1H), 5.50 (d, *J* = 6.1 Hz, 1H), 2.19 (td, *J* = 7.4, 4.7 Hz, 2H), 2.11 (s, 3H), 1.62 (h, *J* = 7.3 Hz, 2H), 0.90 (t, *J* = 7.4 Hz, 3H).

^{13}C NMR (151 MHz, $CDCl_3$)

δ 203.1, 172.3, 135.8, 132.4, 129.6, 122.7, 62.9, 38.2, 27.1, 18.9, 13.7.

HRMS (ESI)

Calcd for $[C_{13}H_{16}BrNO_2, M+H]^+$: 298.0437; Found: 298.0437.

(*R*)-N-(1-(4-iodophenyl)-2-oxopropyl)butyramide (4i)

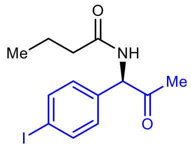

White solid, 69.2 mg, 99% yield, 89% ee, m. p. 70-72 °C, TLC: R_f = 0.67 (PE/EA = 1:1, v/v). HPLC condition: Chiralpak AD-H column (25 cm × 0.46 cm ID), hexane/2-propanol = 90:10, flow rate = 1.0 mL/min, 230 nm UV detector), t_R = 7.55 min (minor) and t_R = 16.77 min (major). $[\alpha]_D^{25}$ -126.4 (*c* 0.5, $CHCl_3$).

^{1}H NMR (600 MHz, $CDCl_3$)

δ 7.70 (d, *J* = 8.1 Hz, 2H), 7.07 (d, *J* = 8.0 Hz, 2H), 6.83 (d, *J* = 6.1 Hz, 1H), 5.47 (d, *J* = 6.1 Hz, 1H), 2.19 (td, *J* = 7.4, 4.4 Hz, 2H), 2.11 (s,3H), 1.62 (h, *J* = 7.3 Hz, 2H), 0.90 (t, *J* = 7.4 Hz, 3H).

^{13}C NMR (151 MHz, $CDCl_3$)

δ 203.0, 172.2, 138.3, 136.4, 129.8, 94.4, 63.0, 38.2, 27.1, 18.9, 13.7.

HRMS (ESI)

Calcd for $[C_{13}H_{16}INO_2$, M+Na$]^+$: 368.0118; Found: 368.0118.

(*R*)-N-(1-(naphthalen-2-yl)-2-oxopropyl)butyramide (4j)

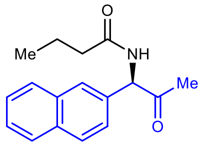

Colorless oil, 54.0 mg, 99% yield, 94% ee, TLC: R_f = 0.68 (PE/EA = 1:1, v/v). HPLC condition: Chiralpak AD-H column (25 cm × 0.46 cm ID), hexane/2-propanol = 80:20, flow rate = 1.0 mL/min, 254 nm UV detector), t_R = 5.22 min (minor) and t_R = 7.47 min (major). $[\alpha]_D^{25}$ -334.2 (*c* 0.5, $CHCl_3$).

^{1}H NMR (600 MHz, $CDCl_3$)

δ 7.83 (m, 4H), 7.53 – 7.46 (m, 2H), 7.36 (dd, *J* = 8.1, 1.8 Hz, 1H), 6.93 (d, *J* = 6.4 Hz, 1H), 5.72 (d, *J* = 6.4 Hz, 1H), 2.21 (dd, *J* = 14.8, 7.3 Hz, 2H), 2.13 (s, 3H), 1.62 (dtt, *J* = 14.5, 7.1, 4.4 Hz, 2H), 0.90 (t, *J* = 7.4 Hz, 3H).

^{13}C NMR (151 MHz, $CDCl_3$)

δ 203.8, 172.3, 133.9, 133.4, 133.2, 129.2, 128.0, 127.8, 127.8, 126.6, 126.6, 124.9, 63.6, 38.3, 27.3, 19.0, 13.7.

HRMS (ESI)

Calcd for $[C_{17}H_{19}NO_2$, M+Na$]^+$: 292.1308; Found: 292.1313.

(*R*)-N-(1-([1,1'-biphenyl]-4-yl)-2-oxopropyl)butyramide (4k)

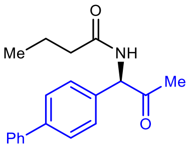

White solid, 58.9 mg, 99% yield, 91% ee, m. p. 117-119 °C, TLC: R_f = 0.68 (PE/EA = 1:1, v/v). HPLC condition: Chiralpak AD-H column (25 cm × 0.46 cm ID), hexane/2-propanol = 80:20, flow rate = 1.0 mL/min, 254 nm UV detector), t_R = 5.09 min (minor) and t_R = 8.94 min (major). $[\alpha]_D^{25}$ -230.8 (*c* 0.5, $CHCl_3$).

^{1}H NMR (600 MHz, $CDCl_3$)

δ 7.57 (dd, *J* = 15.1, 7.7 Hz, 4H), 7.43 (t, *J* = 7.6 Hz, 2H), 7.37 (m, *J* = 16.3, 7.6 Hz, 3H), 6.87 (d, *J* = 6.4 Hz, 1H), 5.60 (d, *J* = 6.3 Hz, 1H), 2.21 (td, *J* = 7.4, 3.7 Hz, 2H), 2.15 (s, 3H), 1.65 (h, *J* = 7.3 Hz, 2H), 0.92 (t, *J* = 7.4 Hz, 3H).

^{13}C NMR (151 MHz, $CDCl_3$)

δ 203.7, 172.3, 141.6, 140.3, 135.5, 128.9, 128.4, 128.0, 127.6, 127.1, 63.2, 38.3, 27.3, 19.0, 13.7.

HRMS (ESI)

Calcd for $[C_{19}H_{21}NO_2, M+Na]^+$: 318.1465; Found: 318.1461.

(*R*)-N-(2-oxo-1-phenylbutyl)butyramide (4l)

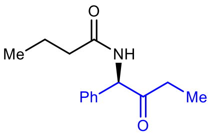

Colorless oil, 47.0 mg, 99% yield, 86% ee, TLC: R_f = 0.80 (PE/EA = 1:1, v/v). HPLC condition: Chiralpak AD-H column (25 cm × 0.46 cm ID), hexane/2-propanol = 90:10, flow rate = 1.0 mL/min, 210 nm UV detector), t_R = 7.05 min (minor) and t_R = 7.58 min (major). $[\alpha]_D^{25}$ -148.4 (*c* 0.5, $CHCl_3$).

^{1}H NMR (600 MHz, $CDCl_3$)

δ 7.35 (m, 2H), 7.31 (m, 3H), 6.82 (d, *J* = 6.6 Hz, 1H), 5.57 (d, *J* = 6.5 Hz, 1H), 2.42 (qd, *J* = 7.3, 4.9 Hz, 2H), 2.19 (td, *J* = 7.4, 4.2 Hz, 2H), 1.63 (h, *J* = 7.3 Hz, 2H), 1.00 (t, *J* = 7.3 Hz, 3H), 0.90 (t, *J* = 7.4 Hz, 3H).

^{13}C NMR (151 MHz, $CDCl_3$)

δ 206.8, 172.2, 136.9, 129.2, 128.5, 128.0, 62.6, 38.3, 33.1, 19.0, 13.7, 7.7.

HRMS (ESI)

Calcd for $[C_{14}H_{19}NO_2, M+H]^+$: 234.1489; Found: 234.1487.

(*R*)-N-(2-oxo-1-(*p*-tolyl)propyl)benzamide (4m)

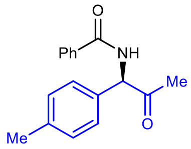

White solid, 53.5 mg, 99% yield, 90% ee, m. p. 68-70 °C, TLC: R_f = 0.70 (PE/EA = 2:1, v/v). HPLC condition: Chiralpak AD-H column (25 cm × 0.46 cm ID), hexane/2-propanol = 80:20, flow rate = 1.0 mL/min, 230 nm UV detector), t_R = 9.28 min (major) and t_R = 16.64 min (minor). $[\alpha]_D^{25}$ -221.0 (*c* 0.5, $CHCl_3$).

^{1}H NMR (600 MHz, $CDCl_3$)
δ 7.81 (d, *J* = 7.7 Hz, 2H), 7.55 (d, *J* = 6.0 Hz, 1H), 7.49 (t, *J* = 7.4 Hz, 1H), 7.41 (t, *J* = 7.6 Hz, 2H), 7.28 (d, *J* = 7.8 Hz, 2H), 7.19 (d, *J* = 7.8 Hz, 2H), 5.69 (d, *J* = 6.1 Hz, 1H), 2.33 (s, 3H), 2.16 (s, 3H).

^{13}C NMR (151 MHz, $CDCl_3$)
δ 203.8, 166.3, 138.6, 133.8, 133.5, 131.7, 130.0, 128.6, 128.0, 127.3, 63.6, 27.1, 21.2.

HRMS (ESI)
Calcd for $[C_{17}H_{17}NO_2, M+H]^+$: 268.1332; Found: 268.1331.

(*R*)-N-(1-(naphthalen-2-yl)-2-oxopropyl)benzamide (4n)

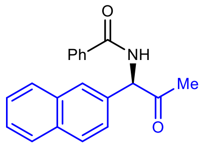

White solid, 61.0 mg, 99% yield, 91% ee, m. p. 106-108 °C, TLC: R_f = 0.68 (PE/EA = 2:1, v/v). HPLC condition: Chiralpak AD-H column (25 cm × 0.46 cm ID), hexane/2-propanol = 80:20, flow rate = 1.0 mL/min, 254 nm UV detector), t_R = 10.24 min (minor) and t_R = 22.97 min (major). $[\alpha]_D^{25}$ -232.8 (*c* 0.5, $CHCl_3$).

^{1}H NMR (600 MHz, $CDCl_3$)
δ 7.93 (s, 1H), 7.85 (m, 5H), 7.68 (d, J = 6.0 Hz, 1H), 7.53 – 7.47 (m, 3H), 7.43 (m, 3H), 5.89 (d, J = 6.0 Hz, 1H), 2.20 (s, 3H).

^{13}C NMR (151 MHz, $CDCl_3$)
δ 203.6, 166.4, 133.8, 133.7, 133.5, 133.3, 131.8, 129.3, 128.6, 128.1, 127.9, 127.75, 127.2, 126.6, 125.0, 64.1, 27.3.

HRMS (ESI)
Calcd for $[C_{20}H_{17}NO_2, M+H]^+$: 304.1332; Found: 304.1329.

Methyl (*R*)-3-((1-(4-fluorophenyl)-2-oxopropyl)carbamoyl)bicyclo[1.1.1]pentane-1-carboxylate (4o)

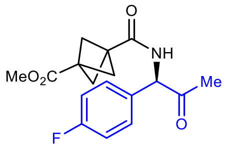

White solid, 64.0 mg, 99% yield, 90% ee, m. p. 120-122 °C, TLC: R_f = 0.67 (PE/EA = 1:1, v/v). HPLC condition: Chiralpak AD-H column (25 cm × 0.46 cm ID), hexane/2-propanol = 90:10, flow rate = 1.0 mL/min, 210 nm UV detector), t_R = 8.17 min (minor) and t_R = 13.44 min (major). $[\alpha]_D^{25}$ -172.8 (*c* 0.5, $CHCl_3$).

^{1}H NMR (600 MHz, $CDCl_3$)
δ 7.31 – 7.26 (m, 2H), 7.07 (t, *J* = 8.4 Hz, 2H), 6.92 (d, *J* = 6.2 Hz, 1H), 5.48 (d, *J* = 6.2 Hz, 1H), 3.69 (s, 3H), 2.28 (s, 6H), 2.11 (s, 3H).

^{13}C NMR (151 MHz, $CDCl_3$)
δ 203.0, 169.7, 168.3, 163.6, 162.0, 132.0, 132.0, 129.82 (d, *J* = 8.3 Hz), 116.34 (d, *J* = 21.9 Hz), 62.4, 52.3, 51.9, 39.0, 36.8, 27.1.

^{19}F NMR (376 MHz, $CDCl_3$)
δ −112.6.

HRMS (ESI)
Calcd for $[C_{17}H_{18}FNO_4, M+H]^+$: 320.1293; Found: 320.1293.

Methyl (*R*)-3-((1-(4-chlorophenyl)-2-oxopropyl)carbamoyl)bicyclo[1.1.1]pentane-1-carboxylate (4p)

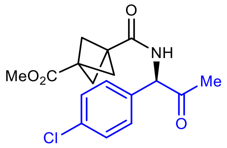

White solid, 67.2 mg, 99% yield, 88% ee, m. p. 122-124 °C, TLC: R_f = 0.68 (PE/EA = 1:1, v/v). HPLC condition: Chiralpak AD-H column (25 cm × 0.46 cm ID), hexane/2-propanol = 90:10, flow rate = 1.0 mL/min, 230 nm UV detector), t_R = 8.58 min (minor) and t_R = 17.51 min (major). $[\alpha]_D^{25}$ -186.4 (*c* 0.5, $CHCl_3$).

^{1}H NMR (600 MHz, $CDCl_3$)
δ 7.35 (d, *J* = 8.1 Hz, 2H), 7.25 (d, *J* = 8.1 Hz, 2H), 6.94 (d, *J* = 6.1 Hz, 1H), 5.46 (d, *J* = 6.1 Hz, 1H), 3.69 (s, 3H), 2.27 (s, 6H), 2.11 (s, 3H).

^{13}C NMR (151 MHz, $CDCl_3$)
δ 202.7, 169.7, 168.3, 134.8, 134.7, 129.5, 129.4, 62.5, 52.3, 51.7, 39.0, 36.8, 27.1.

HRMS (ESI)
Calcd for $[C_{17}H_{18}ClNO_4, M+Na]^+$: 358.0817; Found: 358.0815.

Methyl (R)-3-((1-(4-bromophenyl)-2-oxopropyl)carbamoyl)bicyclo[1.1.1]pentane-1-carboxylate (4q)

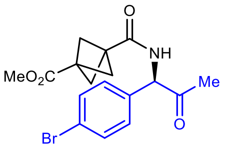

White solid, 76.0 mg, 99% yield, 89% ee, m. p. 132-134 °C, TLC: R_f = 0.68 (PE/EA = 1:1, v/v). HPLC condition: Chiralpak AD-H column (25 cm × 0.46 cm ID), hexane/2-propanol = 90:10, flow rate = 1.0 mL/min, 230 nm UV detector), t_R = 9.11 min (minor) and t_R = 20.67 min (major). $[\alpha]_D^{25}$ -170.6 (c 0.5, $CHCl_3$).

^{1}H NMR (600 MHz, $CDCl_3$)

δ 7.51 (d, J = 8.1 Hz, 2H), 7.19 (d, J = 8.1 Hz, 2H), 6.93 (d, J = 6.1 Hz, 1H), 5.44 (d, J = 6.1 Hz, 1H), 3.69 (s, 3H), 2.27 (s, 6H), 2.11 (s, 3H).

^{13}C NMR (151 MHz, $CDCl_3$)

δ 202.6, 169.7, 168.3, 135.2, 132.5, 129.7, 123.0, 62.6, 52.3, 51.9, 39.0, 36.8, 27.1.

HRMS (ESI)

Calcd for [$C_{17}H_{18}BrNO_4$, M+Na]$^+$: 402.0311; Found: 402.0310.

(R)-2-(1-(4-chlorobenzoyl)-5-methoxy-2-methyl-1H-indol-3-yl)-N-(2-oxo-1-phenylpropyl)acetamide (5a)

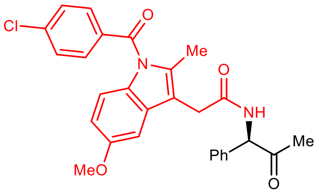

White solid, 80.0 mg, 82% yield, 90% ee, m. p. 128-130 °C, TLC: R_f = 0.50 (PE/EA = 1:1, v/v). HPLC condition: Chiralpak AD-H column (25 cm × 0.46 cm ID), hexane/2-propanol = 90:10, flow rate = 1.0 mL/min, 210 nm UV detector), t_R = 56.76 min (minor) and t_R = 69.67 min (major). $[\alpha]_D^{25}$ -78.4 (c 0.5, $CHCl_3$).

^{1}H NMR (600 MHz, $CDCl_3$)

δ 7.69 (d, J = 8.3 Hz, 2H), 7.49 (d, J = 8.1 Hz, 2H), 7.31 (m, 3H), 7.18 (d, J = 4.9 Hz, 2H), 7.04 (d, J = 6.4 Hz, 1H), 6.97 (d, J = 9.0 Hz, 1H), 6.83 (s, 1H), 6.70 (d, J = 9.2 Hz, 1H), 5.49 (d, J = 6.4 Hz, 1H), 3.75 (s, 3H), 3.67 – 3.56 (m, 2H), 2.34 (s, 3H), 2.05 (s, 3H).

^{13}C NMR (151 MHz, $CDCl_3$)

δ 203.0, 169.2, 168.3, 156.2, 139.4, 136.3, 136.0, 133.8, 131.3, 131.0, 130.2, 129.2, 128.7, 127.9, 115.2, 112.8, 112.6, 100.6, 63.6, 55.7, 32.2, 27.0, 13.4.

HRMS (ESI)

Calcd for [$C_{28}H_{25}ClN_2O_4$, M+Na]$^+$: 511.1395; Found: 511.1391.

(*R*)-1-(2,6-difluorobenzyl)-N-(2-oxo-1-phenylpropyl)-1H-1,2,3-triazole-4-carboxamide (5b)

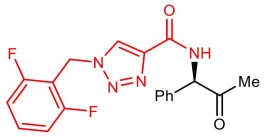

White solid, 68.8 mg, 93% yield, 89% ee, m. p. 152-154 °C, TLC: R_f = 0.50 (PE/EA = 1:1, v/v). HPLC condition: Chiralpak AD-H column (25 cm × 0.46 cm ID), hexane/2-propanol = 90:10, flow rate = 1.0 mL/min, 210 nm UV detector), t_R = 46.30 min (minor) and t_R = 85.78 min (major). $[\alpha]_D^{25}$ -121.8 (*c* 0.5, $CHCl_3$).

^{1}H NMR (600 MHz, $CDCl_3$)
δ 8.29 (d, *J* = 6.8 Hz, 1H), 8.02 (s, 1H), 7.42 – 7.30 (m, 6H), 6.96 (t, *J* = 7.9 Hz, 2H), 5.68 (d, *J* = 6.6 Hz, 1H), 5.64 (s, 2H), 2.15 (s, 3H).

^{13}C NMR (151 MHz, $CDCl_3$)
δ 202.5, 162.1 (d, *J* = 7.0 Hz), 160.5 (d, *J* = 7.0 Hz), 159.2, 143.0, 136.0, 131.8 (t, *J* = 10.4 Hz), 129.3, 128.7, 128.1, 125.5, 111.9 (dd, *J* = 20.9, 4.6 Hz), 110.2 (t, *J* = 18.8 Hz). 63.3, 41.7, 27.1.

^{19}F NMR (376 MHz, $CDCl_3$)
δ −114.1.

HRMS (ESI)
Calcd for [$C_{19}H_{16}F_2N_4O_2$, M+Na]$^+$: 393.1134; Found: 393.1133.

(*R*)-3-(4,5-diphenyloxazol-2-yl)-N-(2-oxo-1-phenylpropyl)propanamide (5c)

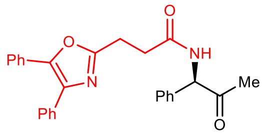

Yellow oil, 85.0 mg, 99% yield, 83% ee, TLC: R_f = 0.50 (PE/EA = 1:1, v/v). HPLC condition: Chiralpak AD-H column (25 cm × 0.46 cm ID), hexane/2-propanol = 90:10, flow rate = 1.0 mL/min, 230 nm UV detector), t_R = 11.72 min (minor) and t_R = 14.66 min (major). $[\alpha]_D^{25}$ -81.2 (*c* 0.5, $CHCl_3$).

^{1}H NMR (400 MHz, $CDCl_3$)
δ 7.56 (d, J = 7.2 Hz, 2H), 7.51 (d, J = 7.2 Hz, 2H), 7.30 (m, 6H), 7.24 (m, 5H), 5.51 (d, J = 6.3 Hz, 1H), 3.13 (t, J = 7.4 Hz, 2H), 2.81 (t, J = 7.4 Hz, 2H), 2.05 (s, 3H).

^{13}C NMR (101 MHz, $CDCl_3$)
δ 203.3, 170.6, 162.2, 145.4, 136.2, 135.0, 132.4, 129.2, 128.9, 128.6, 128.6, 128.5, 128.4, 128.0, 127.9, 126.5, 63.8, 32.7, 27.1, 23.8.

HRMS (ESI)
Calcd for [$C_{27}H_{24}N_2O_3$, M+H]$^+$: 425.1860; Found: 425.1864.

(*S*)-2-(6-methoxynaphthalen-2-yl)-N-((*R*)-2-oxo-1-phenylpropyl)propanamide (5d)

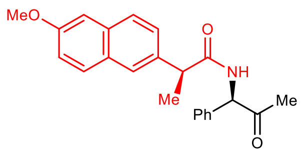

White solid, 72.8 mg, 99% yield, >99:1 dr, m. p. 137-139 °C, TLC: R_f = 0.65 (PE/EA = 1:1, v/v). HPLC condition: Chiralpak AD-H column (25 cm × 0.46 cm ID), hexane/2-propanol = 90:10, flow rate = 1.0 mL/min, 230 nm UV detector), t_R = 19.36 min (minor) and t_R = 33.68 min (major). $[\alpha]_D^{25}$ -52.8 (*c* 0.5, $CHCl_3$).

^{1}H NMR (600 MHz, $CDCl_3$)

δ 7.66 (d, *J* = 8.5 Hz, 1H), 7.62 (d, *J* = 8.9 Hz, 1H), 7.53 (s, 1H), 7.26 (m, 4H), 7.16 – 7.08 (m, 4H), 6.78 (d, *J* = 6.4 Hz, 1H), 5.49 (d, *J* = 6.2 Hz, 1H), 3.90 (s, 3H), 3.76 (q, *J* = 7.1 Hz, 1H), 2.05 (s, 3H), 1.52 (d, *J* = 7.1 Hz, 3H).

^{13}C NMR (151 MHz, $CDCl_3$)

δ 203.3, 173.3, 157.7, 136.4, 136.2, 133.7, 129.3, 129.1, 129.0, 128.5, 127.8, 127.4, 126.3, 126.0, 119.0, 105.6, 63.8, 55.3, 46.6, 27.1, 18.6.

HRMS (ESI)

Calcd for $[C_{23}H_{23}NO_3, M+H]^+$: 362.1751; Found: 362.1751.

(*R*)-N-((*R*)-2-oxo-1-phenylpropyl)-2-(2-oxopyrrolidin-1-yl)butanamide (5e)

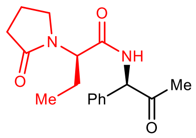

White solid, 60.5 mg, 99% yield, 96:4 dr, m. p. 174-176 °C, TLC: R_f = 0.35 (PE/EA = 1:3, v/v). HPLC condition: Chiralpak AD-H column (25 cm × 0.46 cm ID), hexane/2-propanol = 90:10, flow rate = 1.0 mL/min, 230 nm UV detector), t_R = 9.53 min (minor) and t_R = 11.02 min (major). $[\alpha]_D^{25}$ -173.2 (*c* 0.5, $CHCl_3$).

^{1}H NMR (600 MHz, $CDCl_3$)

δ 7.36 (dd, J = 15.1, 7.0 Hz, 3H), 7.29 (d, J = 7.1 Hz, 2H), 7.24 (d, J = 6.8 Hz, 1H), 5.45 (d, J = 6.7 Hz, 1H), 4.53 (dd, J = 9.0, 6.7 Hz, 1H), 3.43 (td, J = 8.9, 6.2 Hz, 1H), 3.36 (td, J = 8.9, 5.4 Hz, 1H), 2.59 – 2.43 (m, 2H), 2.18 – 2.10 (m, 1H), 2.08 (m, 4H), 1.93 (dp, J = 14.5, 6.9 Hz, 1H), 1.69 – 1.59 (m, 1H), 0.85 (t, J = 7.4 Hz, 3H).

^{13}C NMR (151 MHz, $CDCl_3$)

δ 203.1, 176.6, 169.7, 135.4, 129.4, 128.8, 128.0, 63.7, 56.0, 43.7, 31.1, 27.0, 20.8, 18.3, 10.5.

HRMS (ESI)

Calcd for $[C_{17}H_{22}N_2O_3, M+Na]^+$: 325.1523; Found: 325.1526.

(2*S*,5*R*,6*R*)-3,3-dimethyl-7-oxo-N-((*R*)-2-oxo-1-phenylpropyl)-6-(2-phenylacetamido)-4-thia-1-azabicyclo[3.2.0]heptane-2-carboxamide (5f)

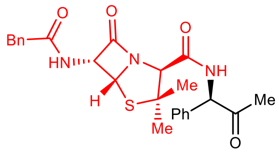

Yellow oil, 93.8 mg, 99% yield, 86:14 dr, TLC: R_f = 0.50 (PE/EA = 1:1, v/v). HPLC condition: Chiralpak AD-H column (25 cm × 0.46 cm ID), hexane/2-propanol = 90:10, flow rate = 1.0 mL/min, 210 nm UV detector), t_R = 33.50 min (minor) and t_R = 50.51 min (major). $[\alpha]_D^{25}$ +19.8 (*c* 0.5, $CHCl_3$).

^{1}H NMR (600 MHz, $CDCl_3$)
δ 7.83 (d, *J* = 6.0 Hz, 1H), 7.35 (m, 4H), 7.29 (m, 4H), 7.24 (m, 2H), 6.26 (d, *J* = 9.2 Hz, 1H), 5.79 (dd, *J* = 9.2, 4.5 Hz, 1H), 5.41 (dd, *J* = 16.7, 5.2 Hz, 2H), 4.10 (s, 1H), 3.60 (s, 2H), 2.09 (s, 3H), 1.54 (s, 3H), 1.17 (s, 3H).

^{13}C NMR (151 MHz, $CDCl_3$)
δ 202.5, 176.3, 170.6, 166.6, 135.4, 133.8, 129.4, 129.2, 129.1, 128.8, 128.0, 127.6, 72.2, 66.5, 64.9, 63.4, 57.5, 43.2, 28.5, 27.0, 26.3.

HRMS (ESI)
Calcd for $[C_{25}H_{27}N_3O_4S, M+Na]^+$: 488.1614; Found: 488.1615.

Methyl N-(*tert*-butoxycarbonyl)-N-((*R*)-2-oxo-1-phenylpropyl)-L-glutaminate (5g)

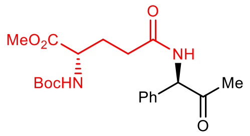

White solid, 79.0 mg, 99% yield, 91:9 dr, m. p. 95-97 °C, TLC: R_f = 0.50 (PE/EA = 1:1, v/v). HPLC condition: Chiralpak AD-H column (25 cm × 0.46 cm ID), hexane/2-propanol = 90:10, flow rate = 1.0 mL/min, 210 nm UV detector), t_R = 20.27 min (minor) and t_R = 27.65 min (major). $[\alpha]_D^{25}$ -203.2 (*c* 0.5, $CHCl_3$).

^{1}H NMR (600 MHz, $CDCl_3$)
δ 7.35 (m, 5H), 5.54 (d, *J* = 6.6 Hz, 1H), 5.33 (d, *J* = 8.2 Hz, 1H), 4.45 (dt, *J* = 9.6, 4.6 Hz, 1H), 3.72 (s, 3H), 2.36 (m, 1H), 2.28 (m, 1H), 2.22 – 2.13 (m, 1H), 2.10 (s, 3H), 1.89 (m, 1H), 1.42 (s, 9H), 1.37 – 1.22 (m, 1H).

^{13}C NMR (151 MHz, $CDCl_3$)
δ 203.5, 172.9, 171.3, 155.8, 136.1, 129.2, 128.6, 128.1, 80.1, 63.8, 52.8, 52.4, 32.1, 28.9, 28.3, 27.1.

HRMS (ESI)
Calcd for $[C_{20}H_{28}N_2O_6, M+H]^+$: 393.2020; Found: 393.2019.

Methyl N-(*tert*-butoxycarbonyl)-N-((*R*)-2-oxo-1-phenylpropyl)-L-asparaginate (5h)

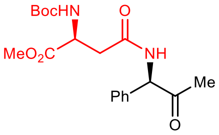

White solid, 76.0 mg, 99% yield, 90:10 dr, m. p. 116-118 °C, TLC: R_f = 0.55 (PE/EA = 1:1, v/v). HPLC condition: Chiralpak AD-H column (25 cm × 0.46 cm ID), hexane/2-propanol = 90:10, flow rate = 1.0 mL/min, 210 nm UV detector), t_R = 12.51 min (minor) and t_R = 18.62 min (major). $[\alpha]_D^{25}$ -123.1 (*c* 0.5, $CHCl_3$).

^{1}H NMR (600 MHz, $CDCl_3$)

δ 7.36 (m, 3H), 7.29 (d, *J* = 7.3 Hz, 2H), 7.06 (d, *J* = 6.3 Hz, 1H), 5.67 (d, *J* = 8.8 Hz, 1H), 5.50 (d, *J* = 6.3 Hz, 1H), 4.54 – 4.44 (m, 1H), 3.72 (s, 3H), 2.99 (dd, *J* = 15.8, 4.6 Hz, 1H), 2.71 (dd, *J* = 15.9, 4.5 Hz, 1H), 2.09 (s, 3H), 1.40 (s, 9H).

^{13}C NMR (151 MHz, $CDCl_3$)

δ 203.1, 171.9, 169.3, 155.6, 136.1, 129.3, 128.7, 127.9, 79.9, 63.5, 52.6, 50.3, 37.6, 28.3, 27.0.

HRMS (ESI)

Calcd for $[C_{19}H_{26}N_2O_6$, M+Na$]^+$: 401.1683; Found: 401.1688.

Typical Procedure for Grignard reaction of 3p and Analytical Data

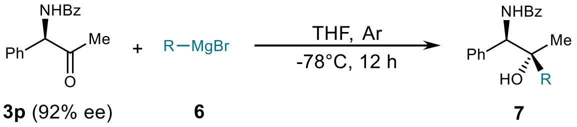

Under Ar, the chiral α-amidoketone **3p** (13 mg, 0.05 mmol) was dissolved in dry THF (2 mL) and was cooled to –78 °C. Then Grignard reagent (1.0 M in THF, 0.6 mL, 0.6 mmol) was added dropwisely and the mixture was stirred at –78 °C until the reaction finished (monitoring by TLC, usually 12 h). The reaction was carefully quenched by water (2 mL) and extracted by ethyl acetate (5 mL ×3). The organic layers were combined, washed with brine, dried over anhydrous $NaSO_4$. After a filtration, the solution was concentrated to afford crude product and the d.r. was determined by ^{1}H NMR. The crude product was further purified by column chromatography (PE/EA = 6:1) to afford **6**.

N-((1*R*, 2*R*)-2-hydroxy-1,2-diphenylpropyl)benzamide (7a)

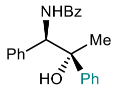

White solid, 15.9 mg, 96% yield, 90% ee, dr >20:1, m. p. 162-164 °C, TLC: R_f = 0.48 (PE/EA = 3:1, v/v). HPLC condition: Chiralpak AD-H column (25 cm × 0.46 cm ID), hexane/2-propanol = 70:30, flow rate = 1.0 mL/min, 230 nm UV detector), t_R = 6.11 min (minor) and t_R = 15.23 min (major). $[\alpha]_D^{25}$ +19.7 (*c* 0.5, $CHCl_3$).

^{1}H NMR (400 MHz, $CDCl_3$)
δ 7.61 – 7.56 (m, 2H), 7.46 – 7.40 (m, 4H), 7.39 – 7.32 (m, 5H), 7.29 (m, 2H), 7.27 – 7.20 (m, 1H), 7.04 (d, *J* = 8.2 Hz, 1H), 5.39 (d, *J* = 8.2 Hz, 1H), 2.38 (s, 1H), 1.39 (s, 3H).

^{13}C NMR (101 MHz, $CDCl_3$)
δ 166.6, 145.2, 138.7, 134.6, 131.3, 128.6, 128.4, 127.9, 127.1, 126.8, 124.8, 76.4, 62.0, 29.4.

HRMS (ESI)
Calcd for $[C_{22}H_{21}NO_2, M+H]^+$: 332.1645; Found: 332.1648.

N-((1*R*, 2*R*)-12-hydroxy-1-phenylpropyl-2-naphthalenyl)benzamide (7b)

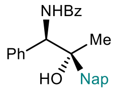

White solid, 14.5 mg, 76% yield, 93% ee, dr >20:1, m. p. 170-172 °C, TLC: R_f = 0.50 (PE/EA = 3:1, v/v). HPLC condition: Chiralpak AD-H column (25 cm × 0.46 cm ID), hexane/2-propanol = 70:30, flow rate = 1.0 mL/min, 230 nm UV detector), t_R = 10.71 min (minor) and t_R = 16.90 min (major). $[\alpha]_D^{25}$ +84.3 (*c* 0.5, $CHCl_3$).

^{1}H NMR (600 MHz, $CDCl_3$)
δ 8.10 (s, 1H), 7.87 – 7.77 (m, 3H), 7.65 (m, 1H), 7.52 – 7.42 (m, 4H), 7.38 (m, 4H), 7.36 – 7.30 (m, 2H), 7.20 (t, *J* = 7.6 Hz, 2H), 7.09 (d, *J* = 8.1 Hz, 1H), 5.54 (d, *J* = 8.1 Hz, 1H), 2.42 (s, 1H), 1.48 (s, 3H).

^{13}C NMR (151 MHz, $CDCl_3$)
δ 166.6, 142.5, 138.7, 134.4, 133.2, 132.5, 131.2, 128.6, 128.5, 128.4, 128.3, 128.2, 128.0, 127.6, 126.8, 126.2, 125.9, 123.8, 123.1, 76.6, 61.8, 29.5.

HRMS (ESI)
Calcd for $[C_{26}H_{23}NO_2, M+Na]^+$: 404.1621; Found: 404.1626.

N-((1*R*, 2*R*)-12-hydroxy-1-phenylpropyl-2-vinyl)benzamide (7c)

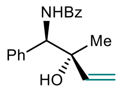

White solid, 13.7 mg, 97% yield, 92% ee, dr >20:1, m. p. 127-129 °C, TLC: R_f = 0.52 (PE/EA = 3:1, v/v). HPLC condition: Chiralpak AD-H column (25 cm × 0.46 cm ID),

hexane/2-propanol = 90:10, flow rate = 1.0 mL/min, 230 nm UV detector), t_R = 10.46 min (minor) and t_R = 17.55 min (major). $[\alpha]_D^{25}$ -34.9 (*c* 0.5, $CHCl_3$).

^1H NMR (400 MHz, $CDCl_3$)

δ 7.66 – 7.53 (m, 2H), 7.35 – 7.29 (m, 1H), 7.28 – 7.09 (m, 7H), 6.98 (d, *J* = 8.3 Hz, 1H), 5.85 (dd, *J* = 17.2, 10.7 Hz, 1H), 5.29 (dd, *J* = 17.1, 1.3 Hz, 1H), 5.03 (dd, *J* = 10.8, 1.3 Hz, 1H), 4.89 (d, *J* = 8.3 Hz, 1H), 1.87 (s, 1H), 1.01 (s, 3H).

^{13}C NMR (101 MHz, $CDCl_3$)

δ 166.7, 142.8, 138.4, 134.6, 131.4, 128.5, 128.4, 128.4, 127.8, 127.0, 113.8, 75.2, 60.4, 26.8.

HRMS (ESI)

Calcd for $[C_{18}H_{19}NO_2, M+H]^+$: 282.1489; Found: 282.1487.

N-((1*R*,2*R*)-2-hydroxy-2-methyl-1-phenylbutyl)benzamide (7d)

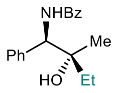

White solid, 13.4 mg, 94% yield, 94% ee, dr >20:1, m. p. 140-142 °C, TLC: R_f = 0.45 (PE/EA = 3:1, v/v). HPLC condition: Chiralpak AD-H column (25 cm × 0.46 cm ID), hexane/2-propanol = 90:10, flow rate = 1.0 mL/min, 210 nm UV detector), t_R = 8.93 min (minor) and t_R = 14.10 min (major). $[\alpha]_D^{25}$ +77.2 (*c* 0.5, $CHCl_3$).

^1H NMR (400 MHz, $CDCl_3$)

δ 7.78 (d, *J* = 7.2 Hz, 2H), 7.48 (m, 1H), 7.44 – 7.36 (m, 4H), 7.35 – 7.24 (m, 4H), 5.05 (d, *J* = 8.7 Hz, 1H), 2.00 (s, 1H), 1.71 (q, *J* = 7.5 Hz, 2H), 1.10 – 0.91 (m, 6H).

^{13}C NMR (101 MHz, $CDCl_3$)

δ 166.8, 139.6, 134.5, 131.5, 128.6, 128.4, 128.3, 127.6, 127.0, 75.1, 59.5, 32.6, 24.5, 8.5.

HRMS (ESI)

Calcd for $[C_{18}H_{21}NO_2, M+H]^+$: 284.1645; Found: 284.1649.

N-((1*R*,2*R*)-2-hydroxy-2-methyl-1-phenyldecyl)benzamide (7e)

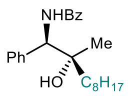

White solid, 13.8 mg, 75% yield, 92% ee, dr >20:1, m. p. 113-115 °C, TLC: R_f = 0.48 (PE/EA = 3:1, v/v). HPLC condition: Chiralpak AD-H column (25 cm × 0.46 cm ID), hexane/2-propanol = 90:10, flow rate = 1.0 mL/min, 230 nm UV detector), t_R = 7.38 min (minor) and t_R = 9.73 min (major). $[\alpha]_D^{25}$ +29.3 (*c* 0.5, $CHCl_3$).

^1H NMR (400 MHz, $CDCl_3$)

δ 7.84 – 7.76 (m, 2H), 7.53 – 7.47 (m, 1H), 7.43 (m, 2H), 7.40 – 7.31 (m, 4H), 7.31 – 7.27 (m, 1H), 7.22 (d, *J* = 8.7 Hz, 1H), 5.05 (d, *J* = 8.7 Hz, 1H),

1.71 – 1.65 (m, 2H), 1.48 – 1.19 (m, 13H), 1.03 (s, 3H), 0.89 – 0.83 (m, 3H).

^{13}C NMR (101 MHz, $CDCl_3$)

δ 166.7, 139.5, 134.6, 131.5, 128.6, 128.4, 128.2, 127.6, 127.0, 100.0, 74.9, 59.9, 40.0, 31.9, 30.1, 29.5, 29.2, 25.2, 24.1, 22.6, 14.1.

HRMS (ESI)

Calcd for [$C_{24}H_{33}NO_2$, M+Na]$^+$: 390.2404; Found: 390.2406.

N-((1*R*,2*R*)-2-hydroxy-2-methyl-1,3-diphenylpropyl)benzamide (7f)

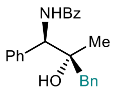

White solid, 13.0 mg, 75% yield, 90% ee, dr >20:1, m. p. 142-144 °C, TLC: R_f = 0.60 (PE/EA = 3:1, v/v). HPLC condition: Chiralpak AD-H column (25 cm × 0.46 cm ID), hexane/2-propanol = 90:10, flow rate = 1.0 mL/min, 230 nm UV detector), t_R = 14.18 min (minor) and t_R = 34.49 min (major). $[\alpha]_D^{25}$ +26.3 (*c* 0.5, $CHCl_3$).

^{1}H NMR (400 MHz, $CDCl_3$)

δ 7.89 – 7.82 (m, 2H), 7.57 – 7.41 (m, 6H), 7.38 – 7.26 (m, 8H), 5.12 (d, *J* = 8.8 Hz, 1H), 3.12 (d, *J* = 13.4 Hz, 1H), 2.97 (d, *J* = 13.4 Hz, 1H), 1.96 (s, 1H), 0.95 (s, 3H).

^{13}C NMR (101 MHz, $CDCl_3$)

δ 166.7, 139.5, 136.7, 134.6, 131.6, 130.7, 128.6, 128.4, 128.3, 127.6, 127.1, 126.8, 74.7, 60.3, 46.0, 25.3.

HRMS (ESI)

Calcd for [$C_{23}H_{23}NO_2$, M+H]$^+$: 346.1802; Found: 346.1806.

N-((1*R*,2*R*)-2-cyclopropyl-2-hydroxy-1-phenylpropyl)benzamide (7g)

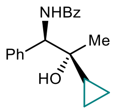

White solid, 12.6 mg, 85% yield, 94% ee, dr >20:1, m. p. 149-151 °C, TLC: R_f = 0.50 (PE/EA = 3:1, v/v). HPLC condition: Chiralpak AD-H column (25 cm × 0.46 cm ID), hexane/2-propanol = 90:10, flow rate = 1.0 mL/min, 254 nm UV detector), t_R = 10.54 min (minor) and t_R = 16.80 min (major). $[\alpha]_D^{25}$ +33.9 (*c* 0.5, $CHCl_3$).

^{1}H NMR (400 MHz, $CDCl_3$)

δ 7.83 – 7.76 (m, 2H), 7.52 – 7.47 (m, 1H), 7.46 – 7.38 (m, 4H), 7.37 – 7.26 (m, 3H), 7.21 (d, *J* = 8.2 Hz, 1H), 5.15 (d, *J* = 8.3 Hz, 1H), 1.90 (s, 1H), 1.02 (m, 4H), 0.65 (dtd, *J* = 9.5, 5.4, 4.0 Hz, 1H), 0.52 – 0.30 (m, 3H).

^{13}C NMR (101 MHz, $CDCl_3$)

δ 165.6, 137.9, 133.1, 130.0, 127.0, 126.9, 126.7, 126.2, 125.5, 71.5, 61.1, 23.4, 18.3, 0.0, -1.2.

HRMS (ESI)

Calcd for [$C_{19}H_{21}NO_2$, M+H]$^+$: 296.1645; Found: 296.1647.

N-((1*R*,2*R*)-2-cyclohexyl-2-hydroxy-1-phenylpropyl)benzamide (7h)

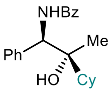

White solid, 12.0 mg, 71% yield, 92% ee, dr >20:1, m. p. 133-135 °C, TLC: R_f = 0.52 (PE/EA = 3:1, v/v). HPLC condition: Chiralpak AD-H column (25 cm × 0.46 cm ID), hexane/2-propanol = 90:10, flow rate = 1.0 mL/min, 230 nm UV detector), t_R = 11.10 min (minor) and t_R = 33.24 min (major). $[\alpha]_D^{25}$ +26.4 (*c* 0.5, $CHCl_3$).

^{1}H NMR (400 MHz, $CDCl_3$)

δ 7.88 – 7.80 (m, 2H), 7.56 – 7.51 (m, 1H), 7.47 (m, 2H), 7.44 – 7.33 (m, 4H), 7.33 – 7.30 (m, 1H), 7.27 (d, *J* = 9.3 Hz, 1H), 5.23 (d, *J* = 8.9 Hz, 1H), 1.95 (m, 2H), 1.78 (m, 4H), 1.59 (s, 1H), 1.30 (m, 2H), 1.22 – 1.03 (m, 3H), 0.92 (s, 3H).

^{13}C NMR (101 MHz, $CDCl_3$)

δ 166.4, 139.8, 134.7, 131.4, 128.6, 128.5, 128.3, 127.5, 127.0, 57.8, 44.7, 28.3, 27.1, 26.9, 26.8, 26.6, 21.5.

HRMS (ESI)

Calcd for $[C_{22}H_{27}NO_2, M+H]^+$: 338.2115; Found: 338.2121.

(*S*)-N-(2-methyl-1-phenylallyl)benzamide (8)[5]

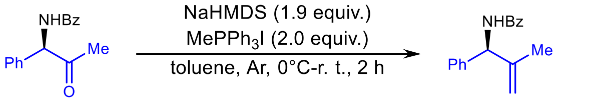

3p (92% ee) **9**, 96% yield, 89% ee

Under Ar, the methylphosphonium iodide (36 mg, 0.1 mmol) was suspended in dry toluene (1 mL) and was cooled to 0 °C. Then NaHMDS (2.0 M in THF, 45 μL, 0.09 mmol) was added dropwisely and the mixture was stirred at 0 °C for 30 min, after which a solution of **3p** (13 mg, 0.05 mmol) in dry toluene (1 mL) was added dropwisely and the mixture was warmed up to room temperature and stired for 2 h. The reaction was quenched by water (1 mL). The emulsion was extracted by diethyl ether (3 mL ×3). The organic layers were combined, washed with brine, dried over anhydrous $NaSO_4$. The solution was concentrated and purified by column chromatography (PE/EA = 6:1) to afford **8** as white solid, 24.1 mg, 96% yield, 89% ee, decomposed at 200 °C, TLC: R_f = 0.78 (PE/EA = 3:1, v/v). HPLC condition: Chiralpak AD-H column (25 cm × 0.46 cm ID), hexane/2-propanol = 90:10, flow rate = 1.0 mL/min, 230 nm UV detector), t_R = 9.76 min (minor) and t_R = 13.93 min (major). $[\alpha]_D^{25}$ -47.4 (*c* 0.5, $CHCl_3$). By comparing the rotational values [4], the absolute configuration of the insertion product is confirmed as *R*.

^{1}H NMR (400 MHz, $CDCl_3$)

δ 7.84 – 7.75 (m, 2H), 7.54 – 7.28 (m, 8H), 6.44 (d, *J* = 8.1 Hz, 1H), 5.68 (d, *J* = 8.1 Hz, 1H), 5.07 (s, 2H), 1.74 (s, 3H).

^{13}C NMR (101 MHz, $CDCl_3$)

δ 166.4, 143.9, 139.8, 134.4, 131.6, 128.9, 128.6, 127.9, 127.5, 127.0, 111.8, 58.9, 20.6.

HRMS (ESI)

Calcd for [$C_{17}H_{17}NO$, M+Na]$^+$: 274.1202; Found: 274.1197.

N-((1*R*,2*S*)-2-hydroxy-1-phenylpropyl)benzamide (9)

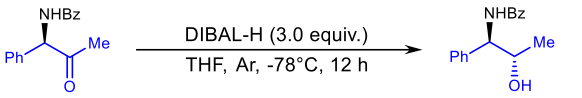

3p (92% ee)

8, 99% yield
92% ee, >20:1 dr

Under Ar, the chiral α-amidoketone **3p** (13 mg, 0.05 mmol) was dissolved in dry THF (1 mL) and was cooled to –78 °C. Then DIBAL-H (1.5 M in THF, 0.1 mL, 0.15 mmol) was added dropwisely and the mixture was stirred at –78 °C for 12 h. The reaction was carefully quenched by water (1 mL). After a filtration, the emulsion was extracted by ethyl acetate (3 mL ×3). The organic layers were combined, washed with brine, dried over anhydrous $NaSO_4$. The solution was concentrated to afford crude product and the d.r. was determined by ^{1}H NMR. The crude product was further purified by column chromatography (PE/EA = 1:1) to afford **9** as white solid, 13.1 mg, 99% yield, 92% ee, dr >20:1, m. p. 123-125 °C, TLC: R_f = 0.50 (PE/EA = 1:1, v/v). HPLC condition: Chiralpak AS-H column (25 cm × 0.46 cm ID), hexane/2-propanol = 90:10, flow rate = 1.0 mL/min, 230 nm UV detector), t_R = 21.33 min (minor) and t_R = 30.07 min (major). $[\alpha]_D^{25}$ +14.4 (*c* 0.5, $CHCl_3$).

^{1}H NMR (400 MHz, $CDCl_3$)

δ 7.90 – 7.77 (m, 2H), 7.55 – 7.49 (m, 1H), 7.48 – 7.42 (m, 2H), 7.41 – 7.29 (m, 5H), 7.16 (d, *J* = 8.0 Hz, 1H), 5.12 (dd, *J* = 8.0, 3.7 Hz, 1H), 4.23 (qd, *J* = 6.2, 3.5 Hz, 1H), 2.22 (s, 1H), 1.15 (d, *J* = 6.4 Hz, 3H).

By comparing the NMR data [6], the relative configuration of the reduction product **7** is confirmed as *anti*.

^{13}C NMR (101 MHz, $CDCl_3$)

δ 167.1, 137.7, 134.4, 131.6, 128.6, 127.9, 127.9, 127.0, 70.1, 59.1, 20.1.

HRMS (ESI)

Calcd for [$C_{16}H_{17}NO_2$, M+Na]$^+$: 278.1151; Found: 278.1156.

(4*R*,5*R*)-5-methyl-2,4-diphenyl-4,5-dihydrooxazole (10)

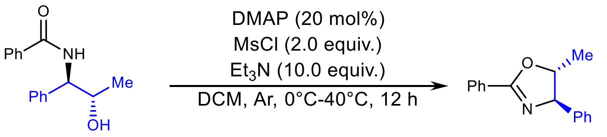

9, 99% yield
92% ee, >20:1 dr

10, 97% yield
90% ee, >20:1 dr

Under Ar, the chiral α-amido alcohol **9** (13 mg, 0.05 mmol) and DMAP (1 mg, 0.01 mmol) was dissolved in dry DCM (4 mL) and was cooled to 0 °C. Then Et_3N (14 μL, 0.1 mmol) and MsCl (8 μL, 0.4 mmol) was added and the mixture was stirred at 0 °C

for 30 min. Then another portion of Et_3N (56 μL, 0.4 mmol) was added and the mixture was heated up to 40 °C and stirred at this temperature for 12 h. The solution was concentrated to afford crude product and the d.r. was determined by ^{1}H NMR. The crude product was further purified by column chromatography (PE/EA = 1:1) to afford **10** as colorless oil, 11.5 mg, 97% yield, 90% ee, dr >20:1, TLC: R_f = 0.63 (PE/EA = 10:1, v/v). HPLC condition: Chiralpak AD-H column (25 cm × 0.46 cm ID), hexane/2-propanol = 90:10, flow rate = 1.0 mL/min, 230 nm UV detector), t_R = 6.25 min (major) and t_R = 10.19 min (minor). $[\alpha]_D^{25}$ -10.0 (*c* 0.5, $CHCl_3$).

^{1}H NMR (400 MHz, $CDCl_3$)
δ 8.05 (d, *J* = 7.1 Hz, 2H), 7.55 – 7.47 (m, 1H), 7.44 (m 6.6 Hz, 2H), 7.39 – 7.32 (m, 2H), 7.29 (m, 3H), 4.85 (d, *J* = 7.5 Hz, 1H), 4.63 – 4.55 (m, 1H), 1.56 (d, *J* = 6.2 Hz, 3H).

^{13}C NMR (101 MHz, $CDCl_3$)
δ 164.0, 142.2, 131.5, 128.8, 128.4, 128.4, 127.9, 127.7, 126.7, 84.2, 20.8.

HRMS (ESI)
Calcd for [$C_{16}H_{15}NO$, M+Na]$^+$: 260.1046; Found: 260.1044.

(*R*)-5-chloro-1-methyl-N-(2-oxo-1-phenylpropyl)-1H-indole-2-carboxamide (11)

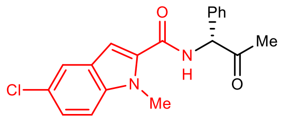

White solid, 68.0 mg, 99% yield, 96% ee, m. p. 100-102 °C, TLC: R_f = 0.74 (PE/EA = 3:1, v/v). HPLC condition: Chiralpak AD-H column (25 cm × 0.46 cm ID), hexane/2-propanol = 90:10, flow rate = 1.0 mL/min, 230 nm UV detector), t_R = 19.73 min (minor) and t_R = 40.19 min (major). $[\alpha]_D^{25}$ -111.1 (*c* 0.5, $CHCl_3$).

^{1}H NMR (600 MHz, $CDCl_3$)
δ 7.58 (m, 2H), 7.44 – 7.38 (m, 4H), 7.35 (m, 1H), 7.24 (m, 2H), 6.91 (s, 1H), 5.67 (d, *J* = 6.0 Hz, 1H), 3.94 (s, 3H), 2.17 (s, 3H).

^{13}C NMR (151 MHz, $CDCl_3$)
δ 203.2, 161.3, 137.4, 136.3, 132.3, 129.4, 128.8, 128.0, 126.8, 126.2, 124.7, 121.1, 111.3, 104.0, 63.8, 31.7, 27.1.

HRMS (ESI)
Calcd for [$C_{19}H_{17}ClN_2O_2$, M+Na]$^+$: 363.0871; Found: 363.0874.

5-chloro-N-((1*R*,2*R*)-2-hydroxy-1-phenylpropyl)-1-methyl-1H-indole-2-carboxamide (12)

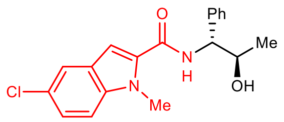

White solid, 34.2 mg, 99% yield, 96% ee, dr >20:1, m. p. 165-167 °C, TLC: R_f = 0.54 (PE/EA = 1:1, v/v). HPLC condition: Chiralpak AS-H column (25 cm × 0.46 cm ID),

hexane/2-propanol = 90:10, flow rate = 1.0 mL/min, 230 nm UV detector), t_R = 19.10 min (minor) and t_R = 26.16 min (major). $[\alpha]_D^{25}$ +393.9 (*c* 0.5, $CHCl_3$).

^{1}H NMR (400 MHz, $CDCl_3$)

δ 7.43 (s, 1H), 7.31 – 7.16 (m, 5H), 7.12 (s, 2H), 7.01 (d, *J* = 8.1 Hz, 1H), 6.71 (s, 1H), 4.93 (dd, *J* = 8.1, 3.8 Hz, 1H), 4.09 (m, 1H), 3.83 (s, 3H), 1.81 (s, 1H), 1.01 (d, *J* = 6.4 Hz, 3H).

^{13}C NMR (101 MHz, $CDCl_3$)

δ 161.7, 137.5, 137.4, 132.9, 128.7, 128.0, 127.9, 126.8, 126.2, 124.6, 120.9, 111.3, 103.4, 70.0, 58.8, 31.7, 20.2.

HRMS (ESI)

Calcd for $[C_{19}H_{19}ClN_2O_2, M+Na]^+$: 365.1027; Found: 365.1026.

1.4 Determination of the Configuration of 7d

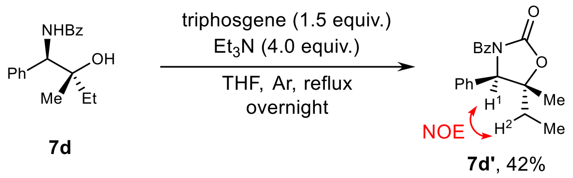

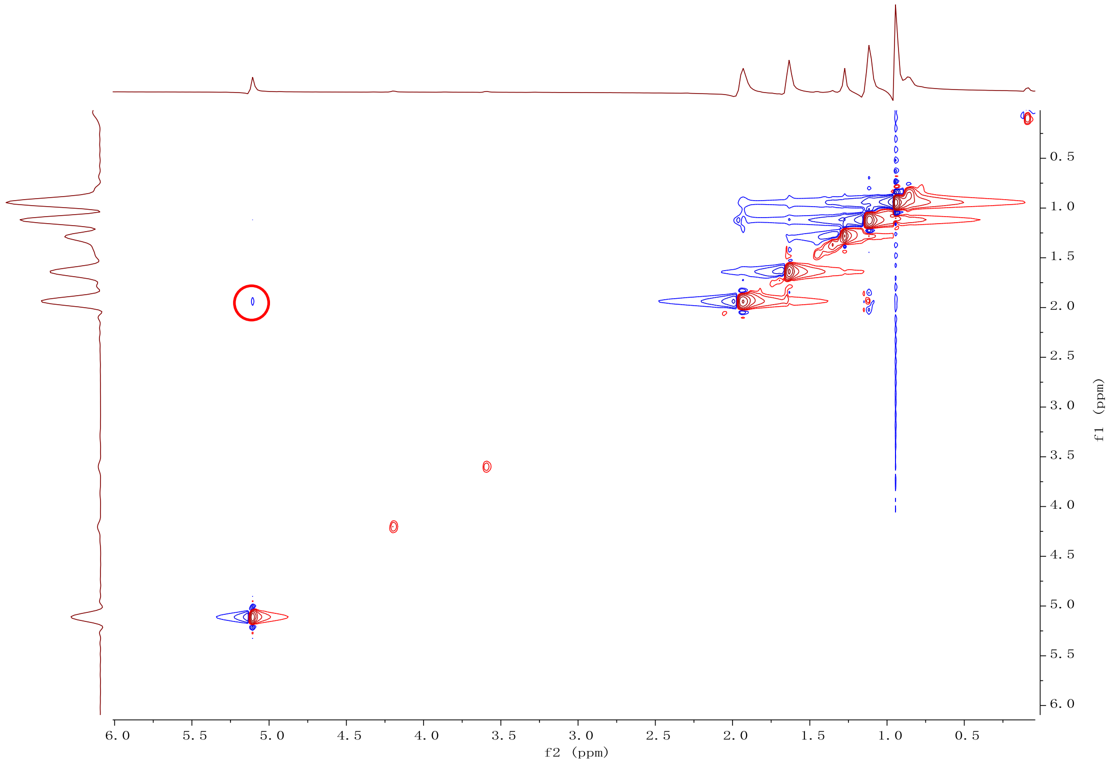

Supplementary Figure 1. ^{1}H-NOE Spectra of 7d'. Signal marked by red circle indicates there is NOE effect between H^1 and H^2.

To determine the relative configuration of **7d**, further transformation to corresponding oxazolidinone **7d'** was performed and ^{1}H-NOE data was collected. The spectrum suggested that the benzylic H and the ethyl group are on the same side of the oxazolidinone, which means the major isomer of **7d** has (1*R*, 2*R*) configuration.

Preparation of **7d'**: **7d** (14 mg, 0.05 mmol, 1.0 equiv) was dissolved with dry THF (1 mL) under argon. Triphosgene (22 mg, 0.075 mmol, 1.5 equiv) and Et_3N (20 mg, 0.2 mmol, 4.0 equiv) were added in one portion at room temperature. The resulting mixture was heated up to reflux and stirred overnight. The mixture was then concentrated and purified by column chromatography to afford the desired oxazolidinone product **7d'**.

1.5 Computational Study

DFT Methods for the enantioselective proton transfer.

DFT calculations were carried out using the Gaussian 16 program package[7]. The B3LYP method[8, 9] and the dispersion energy corrections by D3 version of Grimme's dispersion[10] were used. The def2svp basis set[11, 12] of Ahlrichs and coworkers for all the atoms has been selected for geometry optimizations and calculation of Gibbs energy corrections at 298 K temperature. Final energies were retrieved from single-point calculations at the B3LYP level of theory with def2tzvpp basis set for all atoms. All structures have been optimized considering solvent effects using the SMD model[13] for DCM. Reaction paths were traced by the intrinsic reaction coordinate method for all transition states. All energetics reported throughout the text are in kcal/mol. Structures were generated using CYLview[14].

Supplementary Table 6. Calculated Gibbs energy for transition states of the product.

	Gibbs energy corrections	Single-point energies	Gibbs energy	ΔG
**Zero point: free enol**				
free enol	0.181152	-632.538663	-632.357511	0.0
free ylide	0.180440	-632.533388	-632.352948	2.9
free iminium	0.180622	-632.513694	-632.333072	15.3
(*R*)-3c	0.177199	-632.546574	-632.369375	-7.4
Rh_2OAc_4	0.157444	-1135.831121	-1135.673677	-
Rh_2TPA_4	1.073033	-3909.780089	-3908.707056	-
C8	0.504919	-2282.305784	-2281.800865	-
**Zero point: free enol + C8**				
TSR-I	0.709812	-2914.848126	-2914.138314	12.6
TSR-II	0.713269	-2914.851298	-2914.138029	12.8
TSR-III	0.713814	-2914.849411	-2914.135597	14.3
TSR-IV	0.710728	-2914.845536	-2914.134808	14.8
TSR-V	0.713565	-2914.847494	-2914.133929	15.3
TSR-VI	0.712084	-2914.845842	-2914.133758	15.4
TSR-VII	0.710976	-2914.844566	-2914.133590	15.6
TSS-I	0.708943	-2914.845096	-2914.136153	13.9
TSS-II	0.710528	-2914.845448	-2914.134920	14.7
TSS-III	0.712937	-2914.846837	-2914.133900	15.4
TSS-IV	0.715639	-2914.847455	-2914.131816	16.7
TSS-V	0.710229	-2914.840250	-2914.130021	17.8
**Zero point: free enol + C8 + Rh_2OAc_4**				
RhTSRa-I	0.895831	-4050.708297	-4049.812466	12.3
RhTSRa-II	0.900255	-4050.711284	-4049.811029	13.2
RhTSRa-III	0.896835	-4050.704530	-4049.807695	15.3
RhTSRa-IV	0.900576	-4050.707171	-4049.806595	16.0
RhTSRa-V	0.897052	-4050.702533	-4049.805481	16.7
RhTSRa-VI	0.899592	-4050.704212	-4049.804620	17.2
RhTSSa-I	0.895858	-4050.698767	-4049.802909	18.3
RhTSSa-II	0.896804	-4050.698570	-4049.801766	19.0
RhTSSa-III	0.899381	-4050.697771	-4049.798390	21.1
RhTSSa-IV	0.899616	-4050.694308	-4049.794692	23.4
RhTSRb-I	0.893559	-4050.716066	-4049.822507	6.0
RhTSRb-II	0.889340	-4050.710436	-4049.821096	6.9
RhTSRb-III	0.894231	-4050.713918	-4049.819687	7.8
RhTSRb-IV	0.896692	-4050.712834	-4049.816142	10.0
RhTSSb-I	0.888054	-4050.709811	-4049.821757	6.5

RhTSSb-II	0.891474	-4050.711429	-4049.819955	7.6
RhTSSb-III	0.890562	-4050.709965	-4049.819403	7.9
RhTSSb-III	0.894589	-4050.711899	-4049.817310	9.3
**Zero point: RhTPA-TSR**				
RhTPA-TSR	1.818607	-6824.696157	-6822.877550	0
RhTPA-TSS	1.811275	-6824.685378	-6822.874103	2.2

We firstly calculated the energy of three possible intermiediates generating from the decomposition of rhodium associated ylide intermediate, and the free enol turned out to have the lowest energy. Therefore, the 1, 3-proton transfer of free enol was considered as the enantio-determining step. Among all the twelve squaramide catalyzed proton transfer models, **TSR-I** has the lowest energy, requiring 12.6 kcal/mol free energy based on free enol to complete the proton transfer.

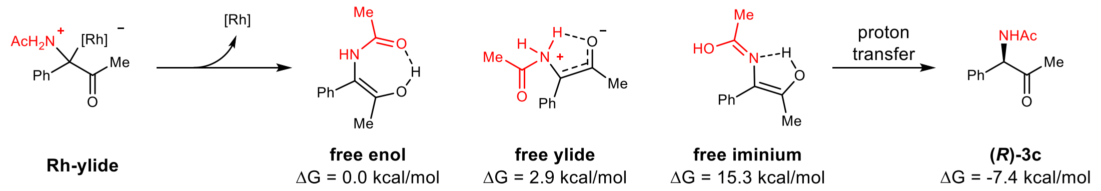

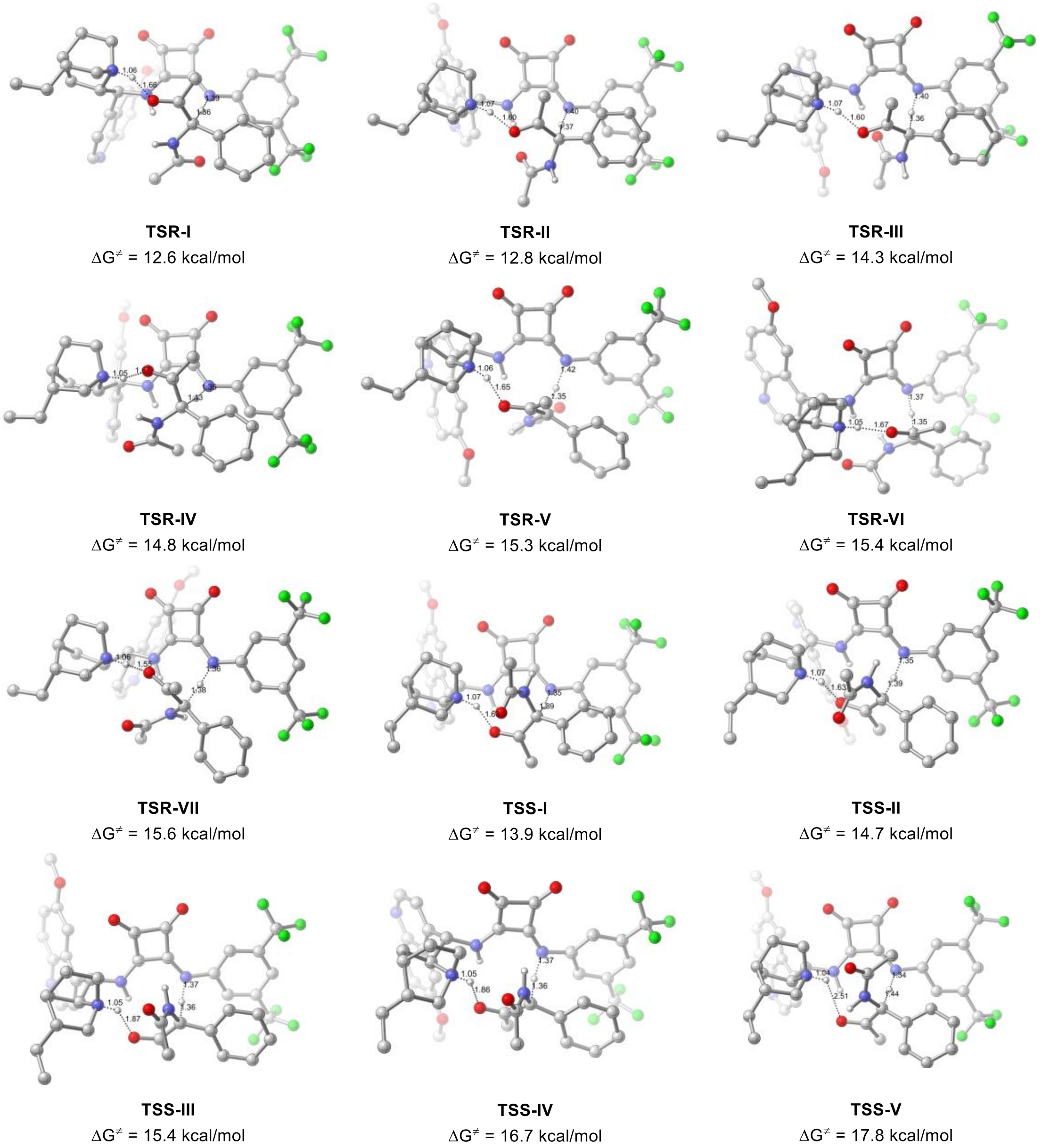

Supplementary Figure 2. Transition state structure without the participation of rhodium complex. Performed at the b3lyp-D3(BJ)/def2tzvpp (SMD-dichloromethane)//b3lyp-D3(BJ)/def2svp (gas) level.

Then, we tried to introduce the rhodium catalyst into the transtion states with lower energies (**TSR-I**, **TSR-II**, **TSR-III** and **TSS-I**, **TSS-II**) through coordination with the squaramide to see if such an interaction can lower the energies. To simplify the calculation, $Rh_2(OAc)_4$ instead of $Rh_2(TPA)_4$ was used firstly. As a result, coordination of rhodium catalyst to oxygen of squaramide doesn't significantly lower the energy barrier, however, bonding of rhodium with the quinoline motif lowered the energy by 6.6 kcal/mol. Taken all the calculations considering the participation of $Rh_2(OAc)_4$ together, **RhTSRb-I** has the has the lowest energy, requiring 6.0 kcal/mol free energy based on free enol to complete the proton transfer.

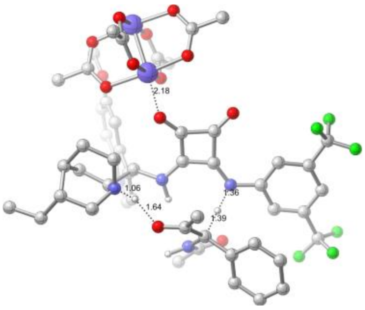

RhTSRa-I
$\Delta G^{\neq}$ = 12.3 kcal/mol

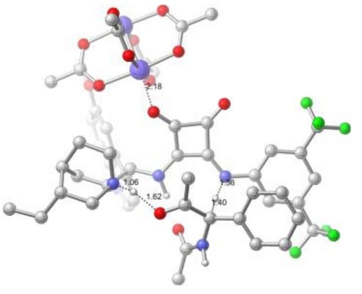

RhTSRa-II
$\Delta G^{\neq}$ = 13.2 kcal/mol

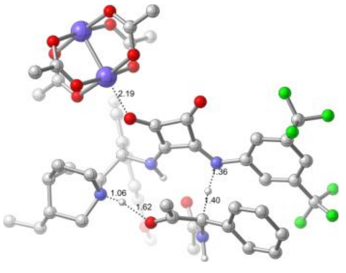

RhTSRa-III
$\Delta G^{\neq}$ = 15.3 kcal/mol

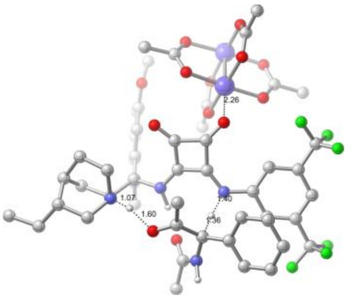

RhTSRa-IV
$\Delta G^{\neq}$ = 16.0 kcal/mol

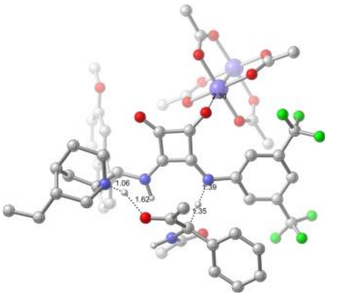

RhTSRa-V
$\Delta G^{\neq}$ = 16.7 kcal/mol

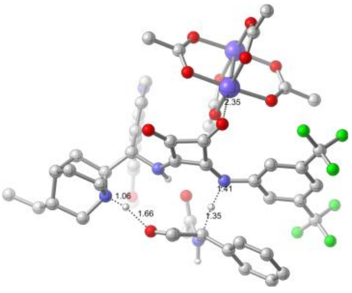

RhTSRa-VI
$\Delta G^{\neq}$ = 17.2 kcal/mol

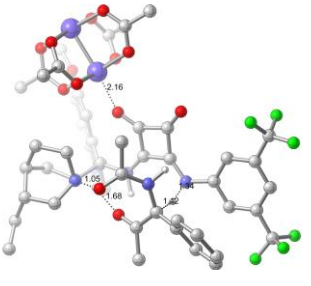

RhTSSa-I
$\Delta G^{\neq}$ = 18.3 kcal/mol

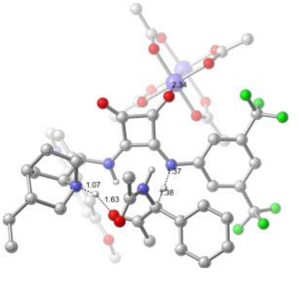

RhTSSa-II
$\Delta G^{\neq}$ = 19.0 kcal/mol

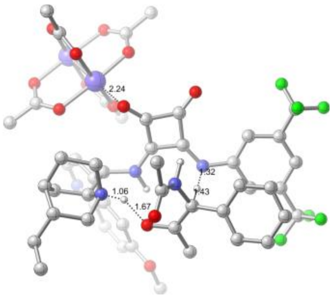

RhTSSa-III
$\Delta G^{\neq}$ = 21.1 kcal/mol

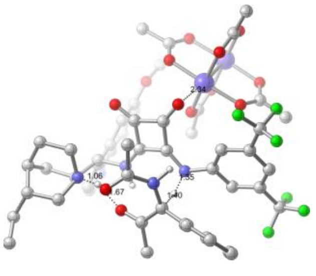

RhTSSa-VI
$\Delta G^{\neq}$ = 23.4 kcal/mol

Supplementary Figures 3. Transition state structure with rhodium acetate dimer coordinated to the oxygen of squaramide. Performed at the b3lyp-D3(BJ)/def2tzvpp (SMD-dichloromethane)//b3lyp-D3(BJ)/def2svp (gas) level.

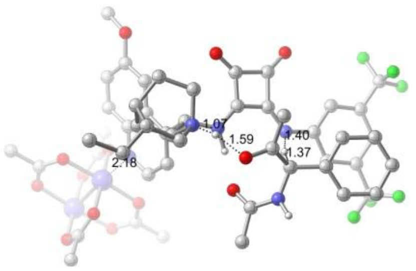

RhTSRb-I
ΔG≠ = 6.0 kcal/mol

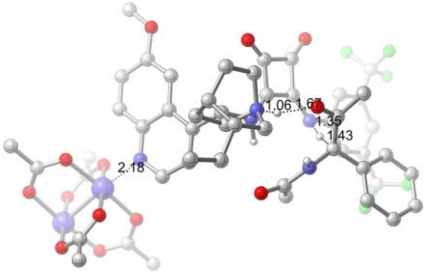

RhTSRb-II
ΔG≠ = 6.9 kcal/mol

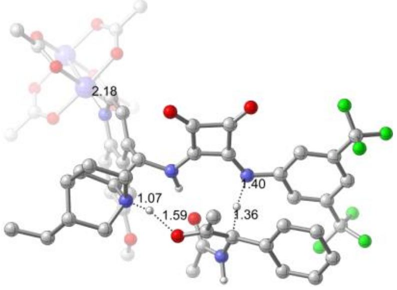

RhTSRb-III
ΔG≠ = 7.8 kcal/mol

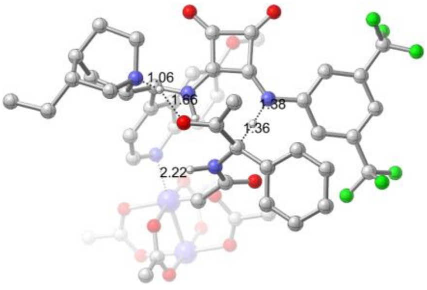

RhTSRb-IV
ΔG≠ = 10.0 kcal/mol

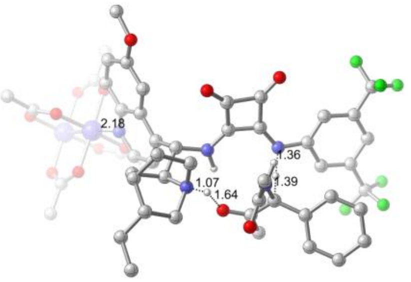

RhTSSb-I
ΔG≠ = 7.6 kcal/mol

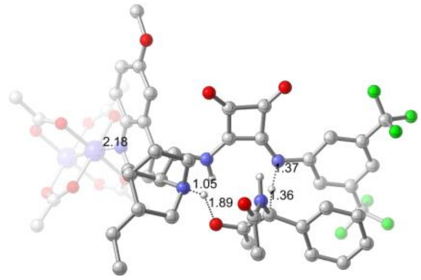

RhTSSb-II
ΔG≠ = 7.6 kcal/mol

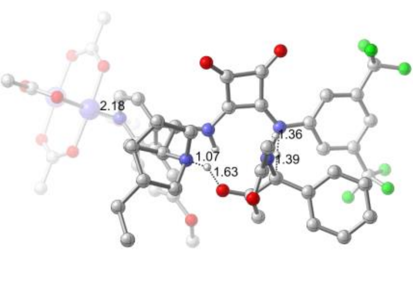

RhTSSb-III
ΔG≠ = 7.9 kcal/mol

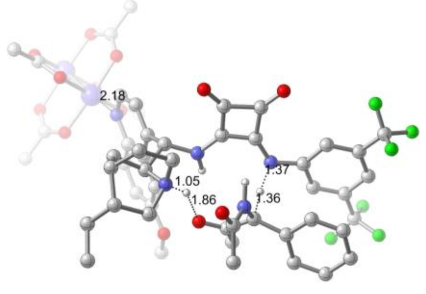

RhTSSb-IV
ΔG≠ = 9.3 kcal/mol

Supplementary Figure 4. Transition state structure with rhodium acetate dimer coordinated to the nitrogen of squaramide. Performed at the b3lyp-D3(BJ)/def2tzvpp (SMD-dichloromethane)//b3lyp-D3(BJ)/def2svp (gas) level.

Finally, we changed the ligand of the dirhodium complex from acetate anion to triphenyl acetate anion (TPA), then reoptimized the structures and recalculated the

energies based on the coordination and proton transfer modes of **RhTSRb-I** and **RhTSSb-I**. Calculation results reveal that **RhTPA-TSR** is mostly likely the proton transfer mode of this squaramide and $Rh_2(TPA)_4$ co-catalyzed N-H insertion reaction, which requires only -7.6 kcal/mol based on the free enol to complete the proton transfer.

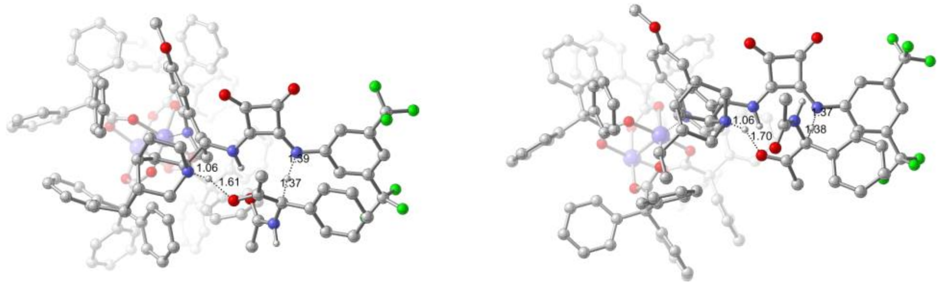

RhTPA-TSR
$\Delta G^{\neq}$ = -7.6 kcal/mol

RhTPA-TSS
$\Delta G^{\neq}$ = -5.4 kcal/mol

Supplementary Figure 5. Transition state structures with rhodium triphenylacetate dimer coordinated to the nitrogen of squaramide. Performed at the b3lyp-D3(BJ)/def2tzvpp (SMD-dichloromethane)//b3lyp-D3(BJ)/def2svp (gas) level.

2 Supplementary Figures

2.1 NMR Spectra

1-diazo-1-phenylpropan-2-one (2a)

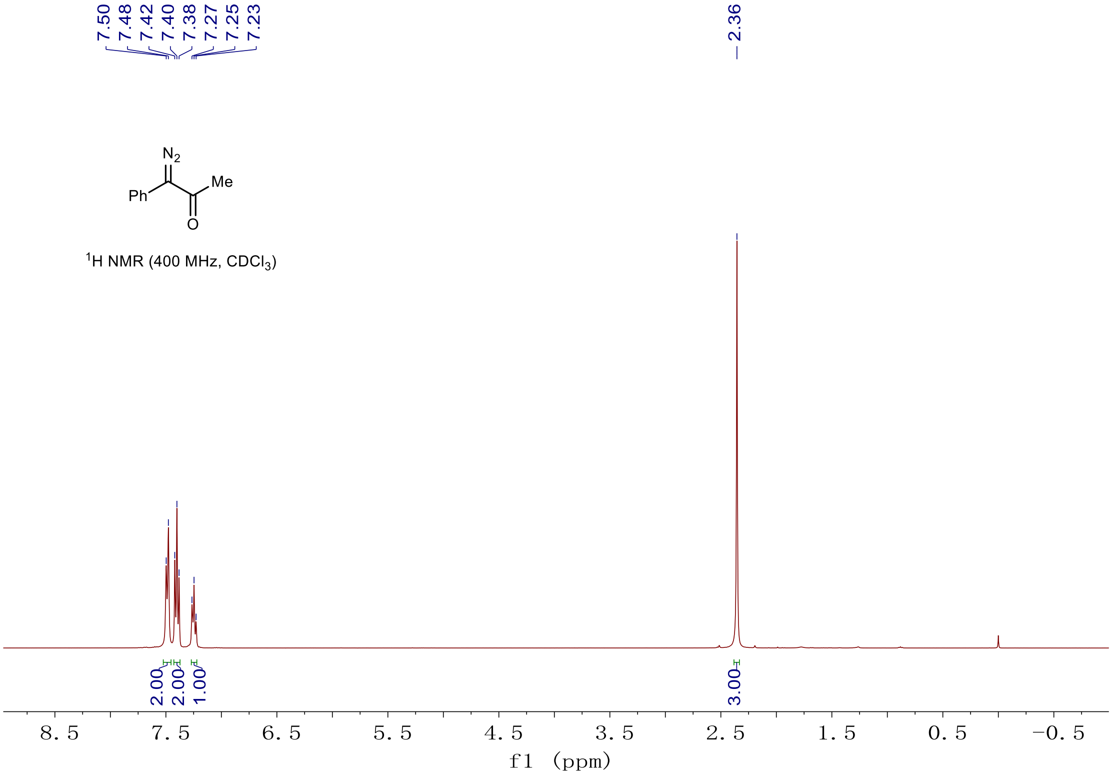

Supplementary Figure 6. ^{1}H NMR spectrum of compound 2a.

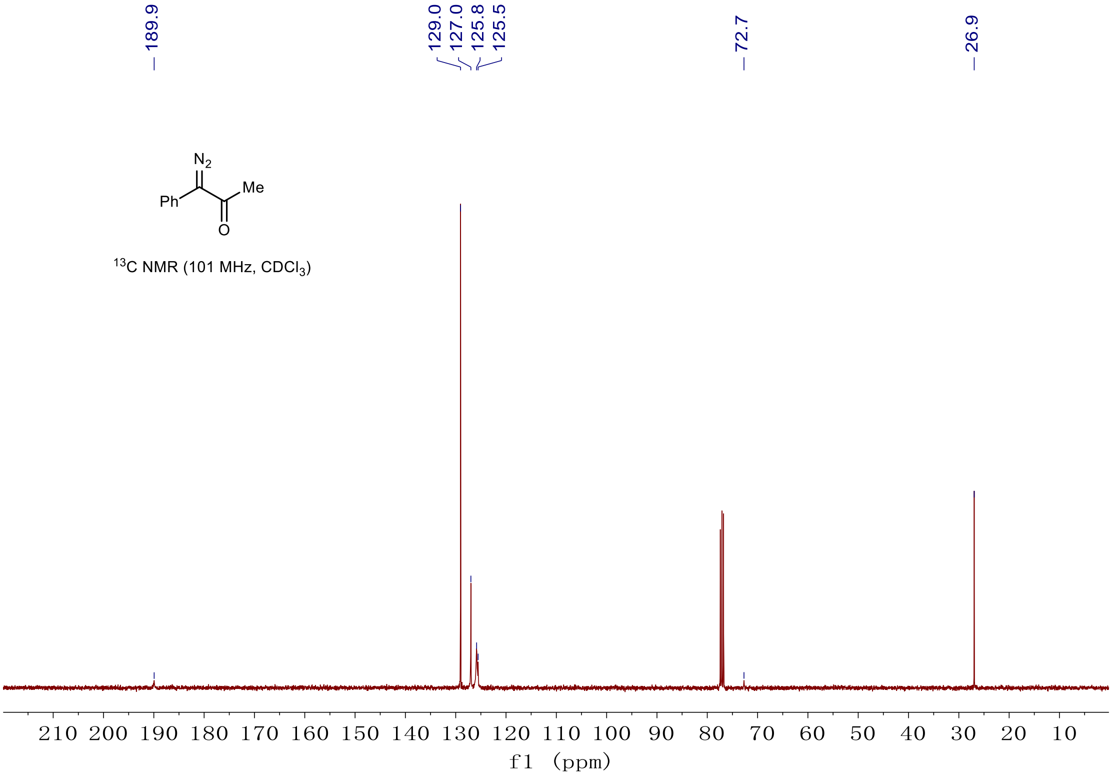

Supplementary Figure 7. ^{13}C NMR spectrum of compound 2a.

1-diazo-1-(p-tolyl)propan-2-one (2b)

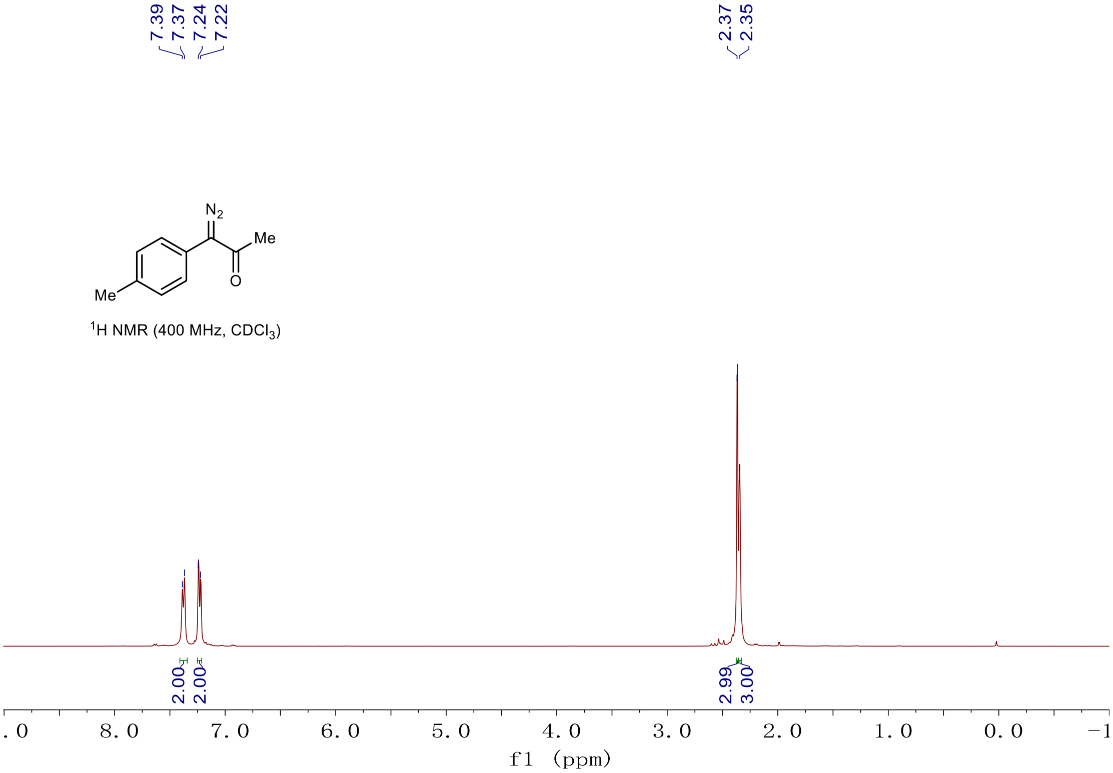

Supplementary Figure 8. ^{1}H NMR spectrum of compound 2b.

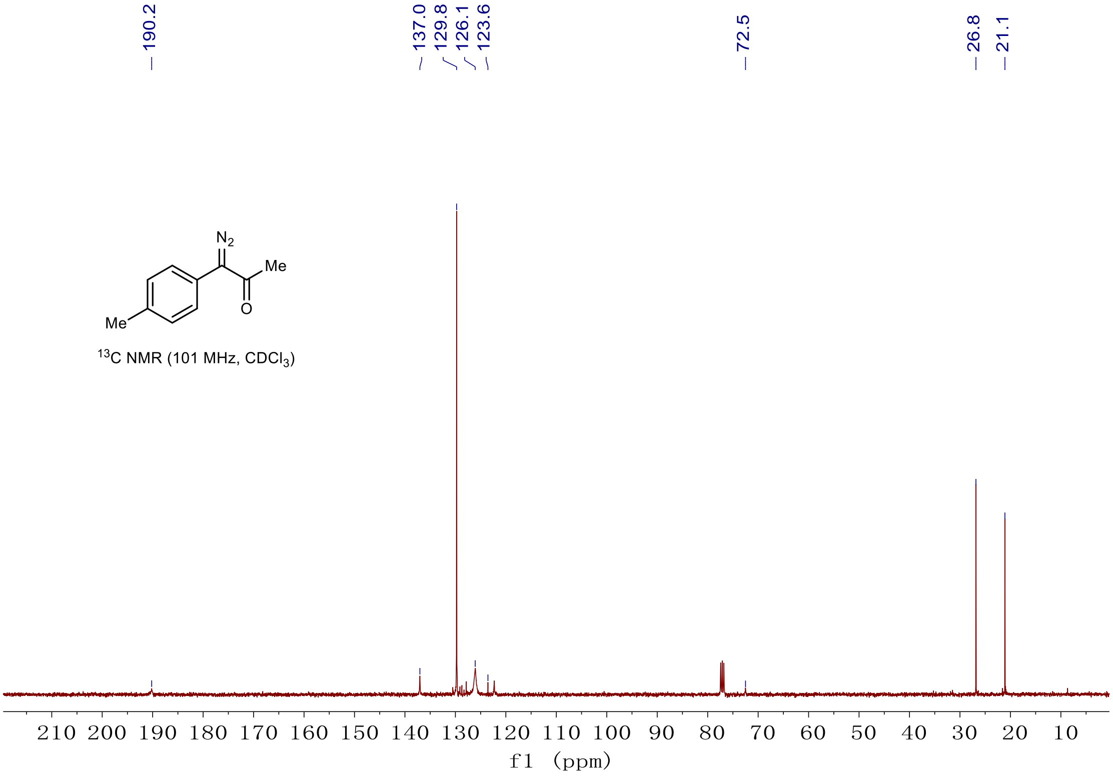

Supplementary Figure 9. ^{13}C NMR spectrum of compound 2b.

1-diazo-1-(m-tolyl)propan-2-one (2c)

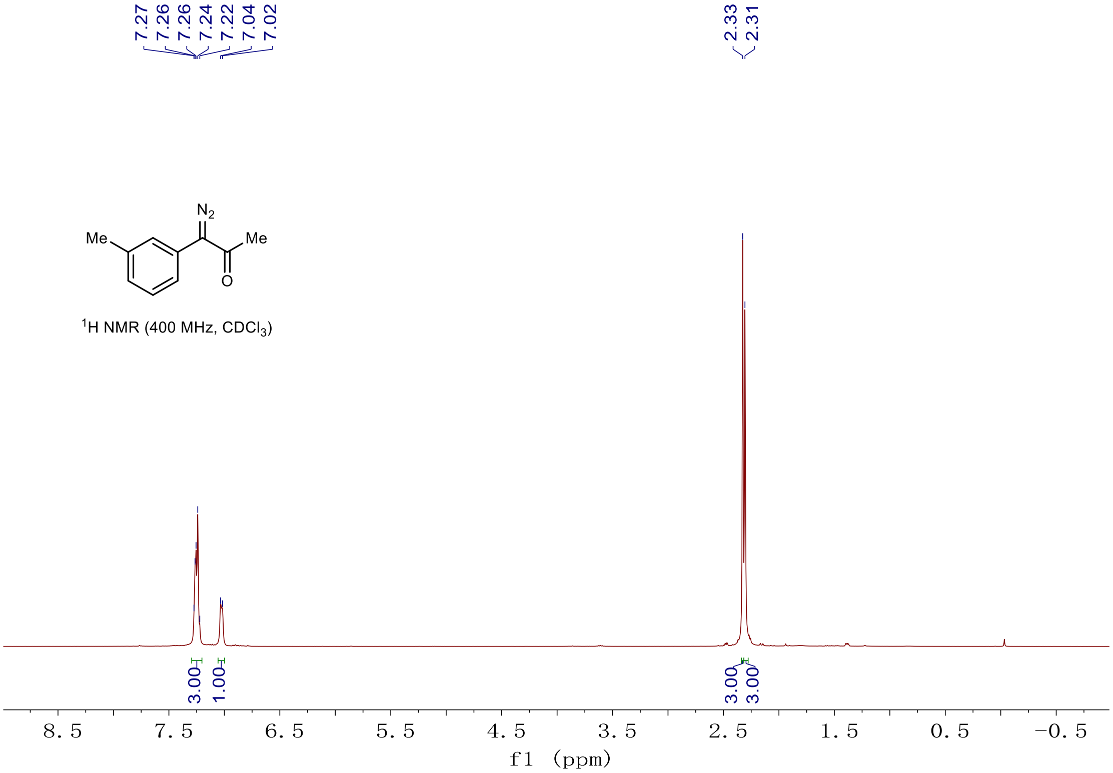

Supplementary Figure 10. ^{1}H NMR spectrum of compound 2c.

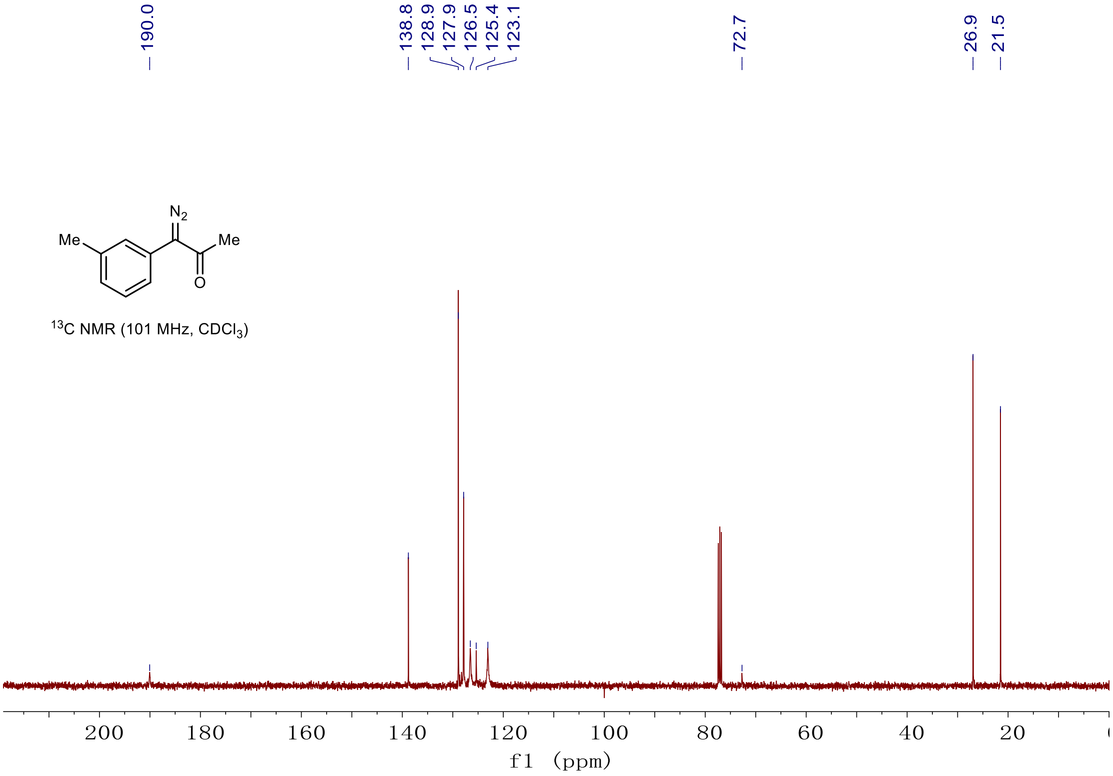

Supplementary Figure 11. ^{13}C NMR spectrum of compound 2c.

1-diazo-1-(4-(trifluoromethyl)phenyl)propan-2-one (2d)

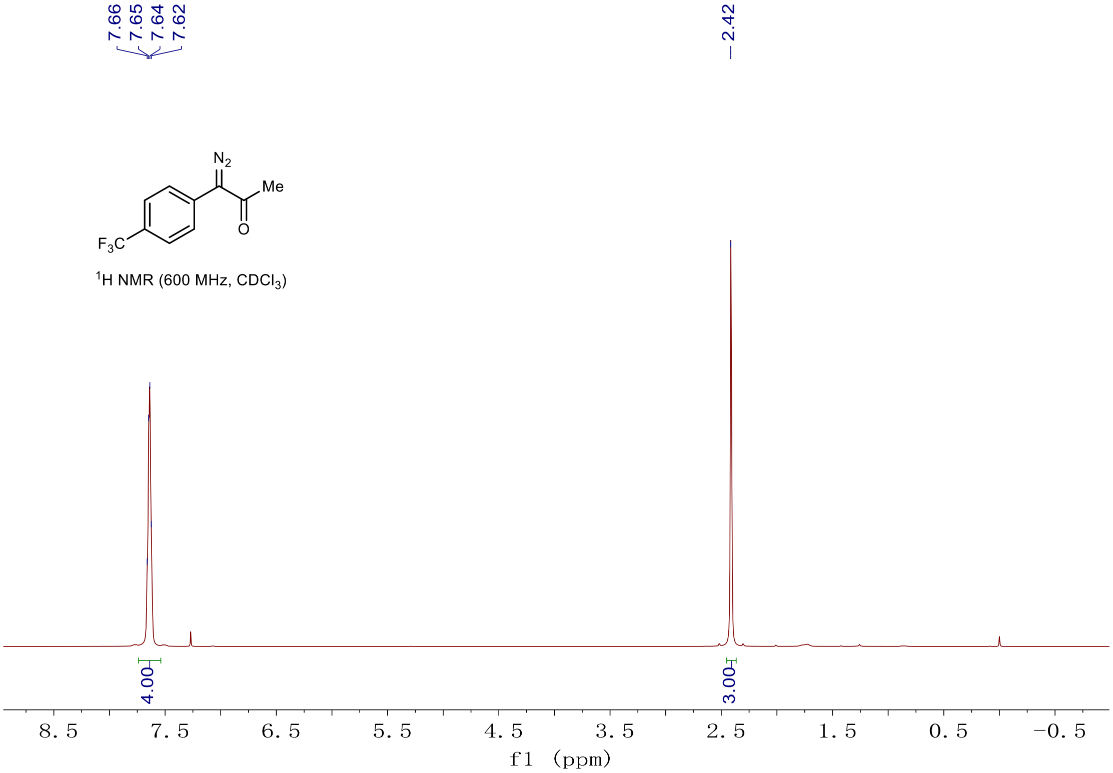

Supplementary Figure 12. ^{1}H NMR spectrum of compound 2d.

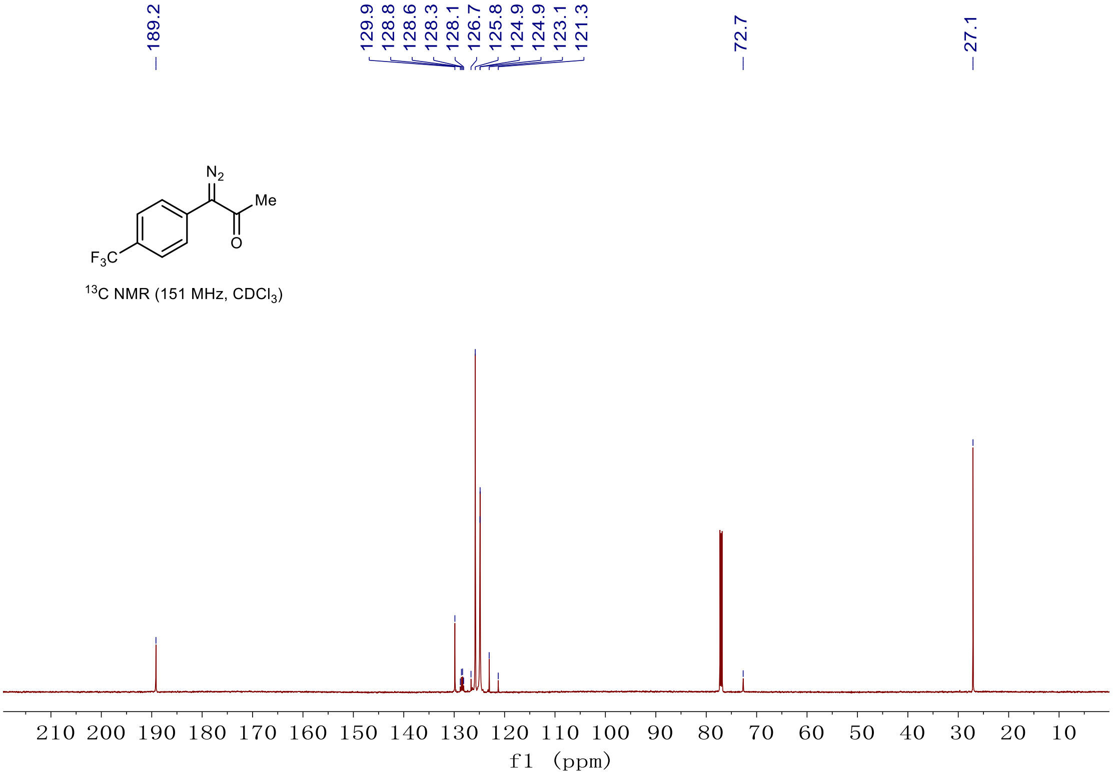

Supplementary Figure 13. ^{13}C NMR spectrum of compound 2d.

1-diazo-1-(3-methoxyphenyl)propan-2-one (2e)

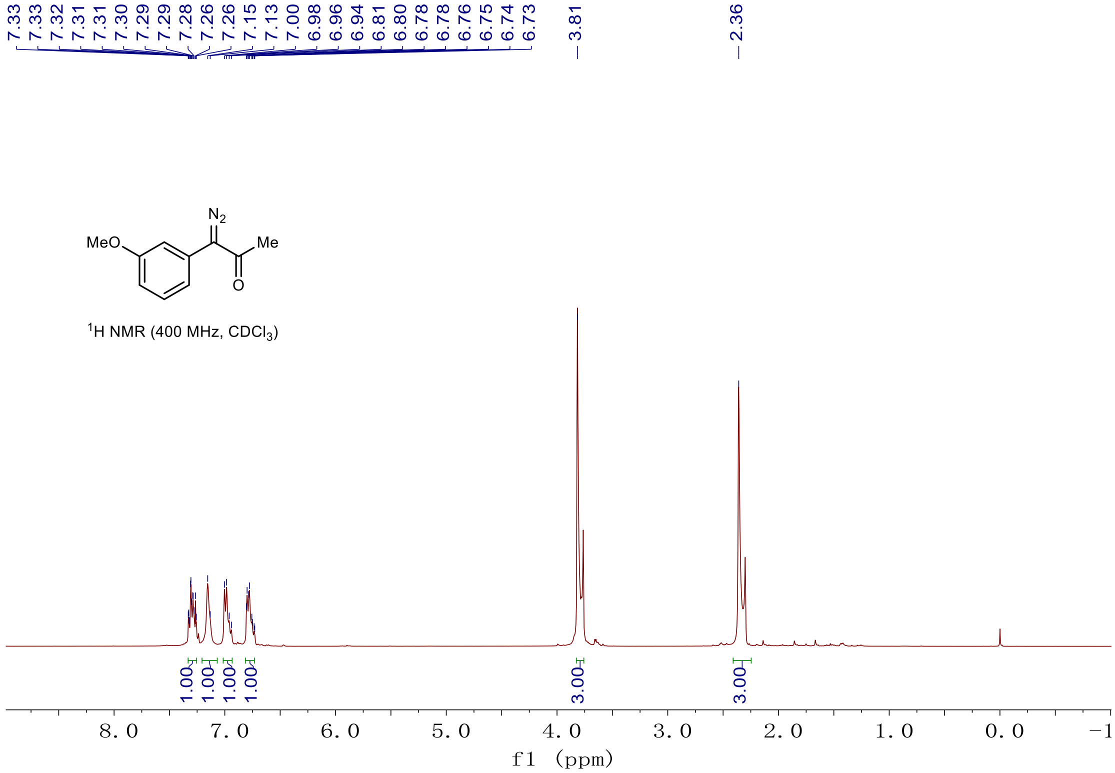

Supplementary Figure 14. ^{1}H NMR spectrum of compound 2e.

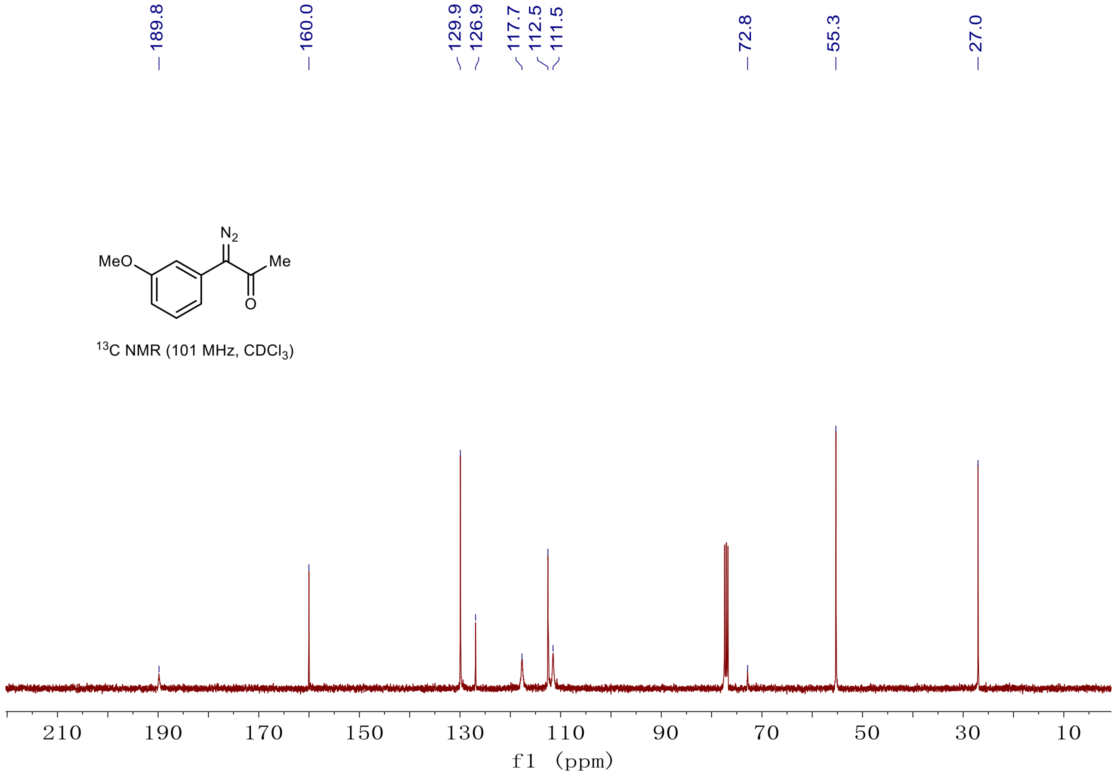

Supplementary Figure 15. ^{13}C NMR spectrum of compound 2e.

1-diazo-1-(2-fluorophenyl)propan-2-one (2f)

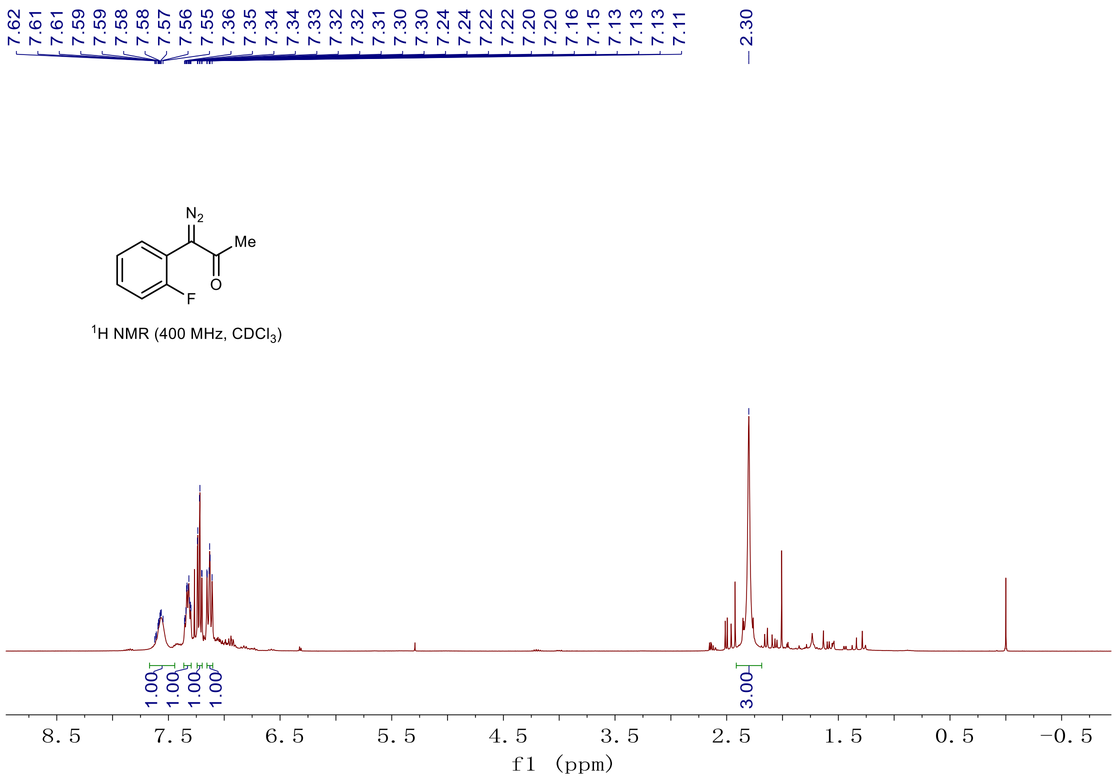

Supplementary Figure 16. ^{1}H NMR spectrum of compound 2f.

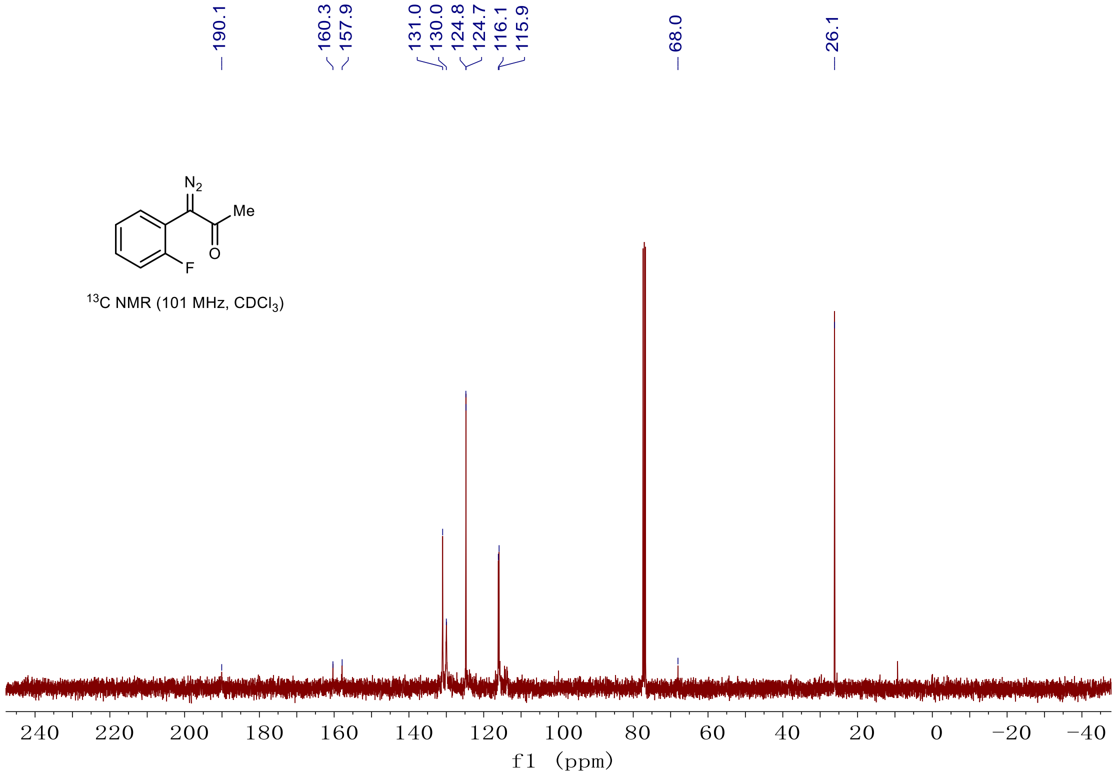

Supplementary Figure 17. ^{13}C NMR spectrum of compound 2f.

1-diazo-1-(4-fluorophenyl)propan-2-one (2g)

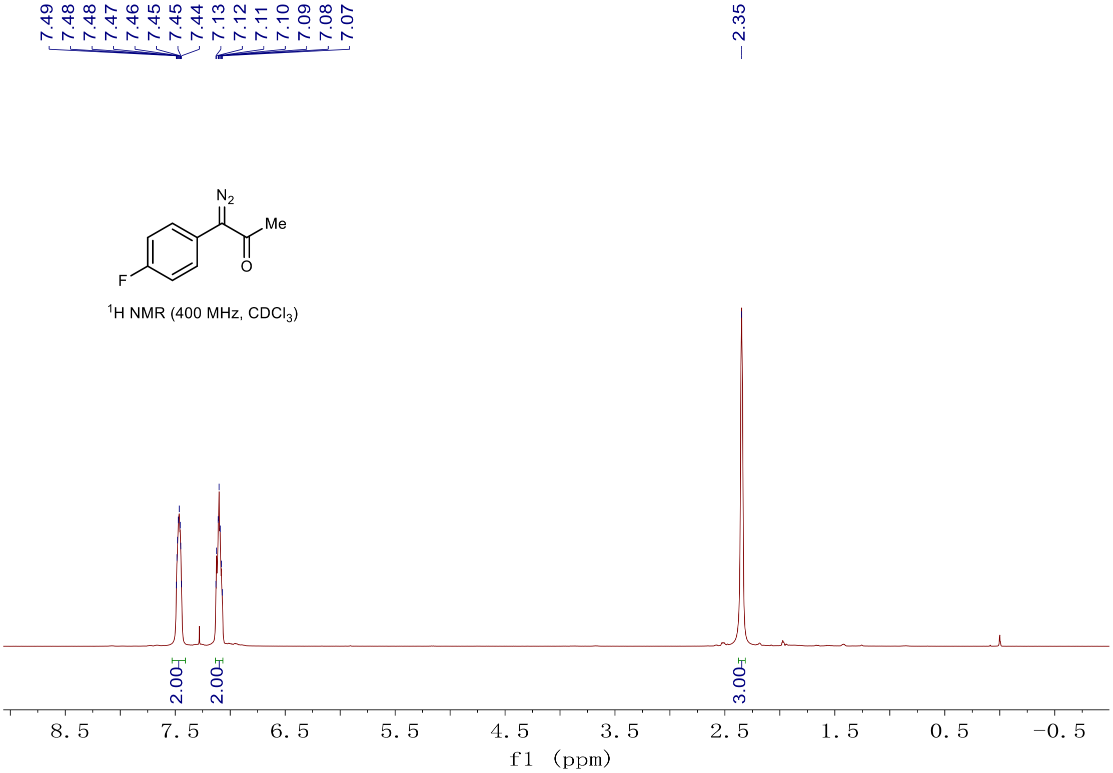

Supplementary Figure 18. ^{1}H NMR spectrum of compound 2g.

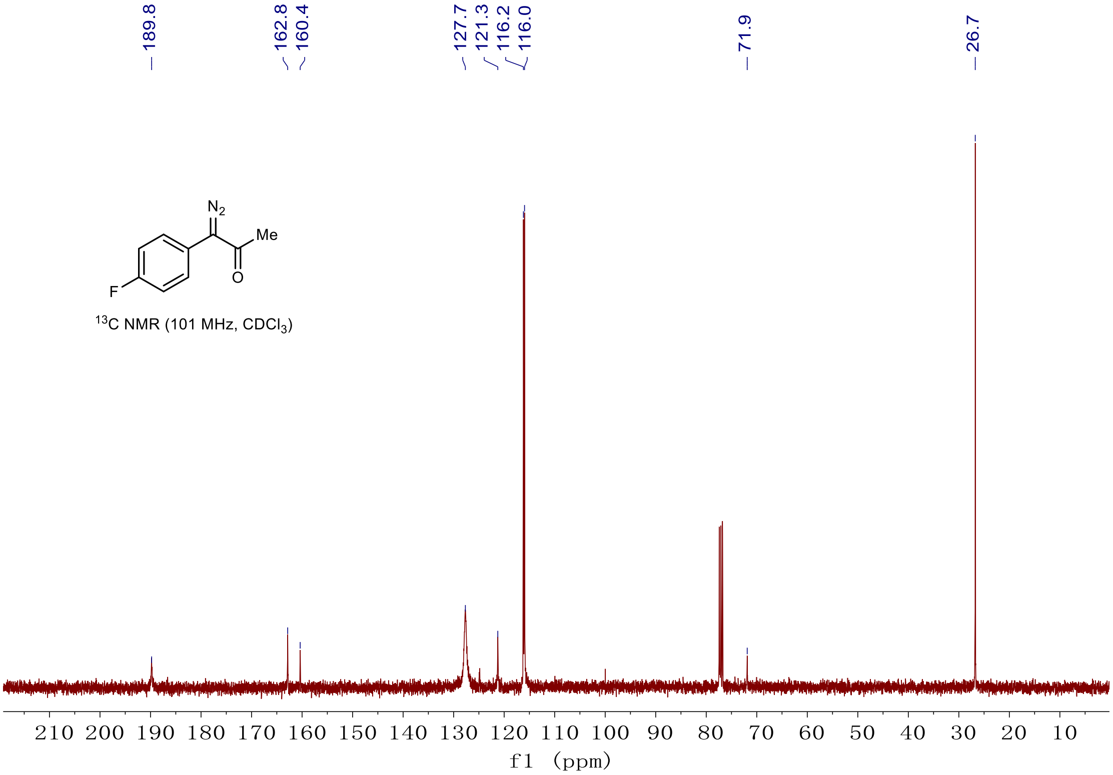

Supplementary Figure 19. ^{13}C NMR spectrum of compound 2g.

1-(4-chlorophenyl)-1-diazopropan-2-one (2h)

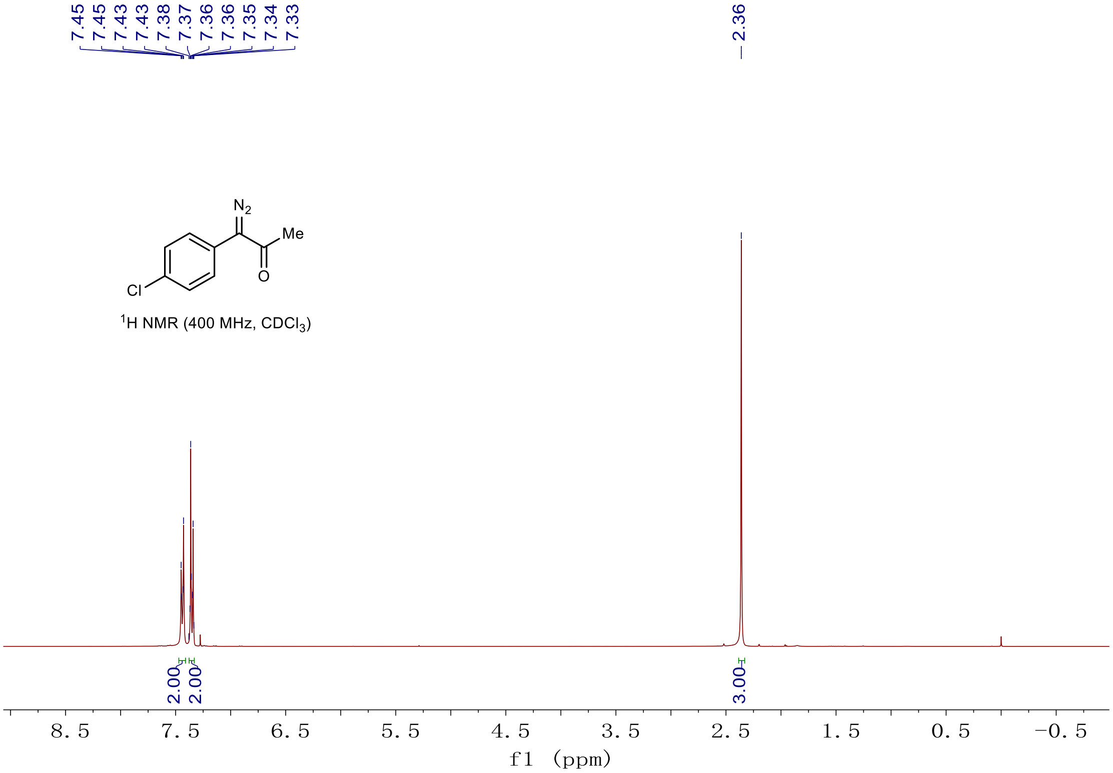

Supplementary Figure 20. ^{1}H NMR spectrum of compound 2h.

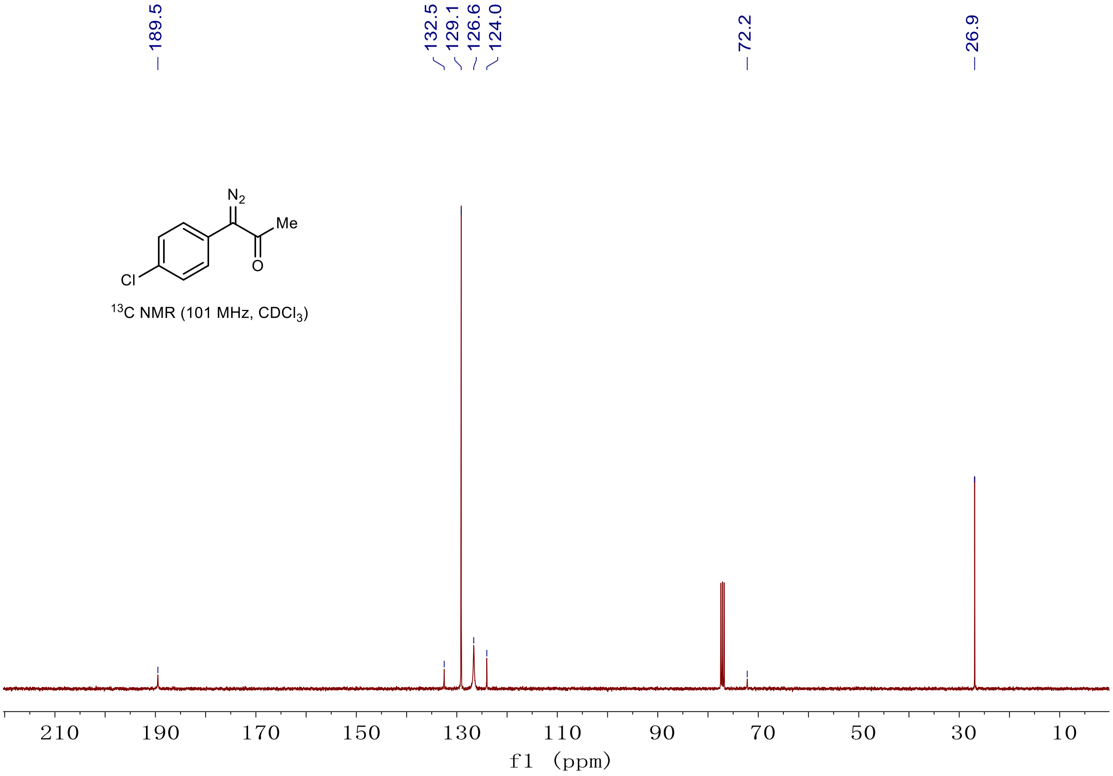

Supplementary Figure 21. ^{13}C NMR spectrum of compound 2h.

1-(4-bromophenyl)-1-diazopropan-2-one (2i)

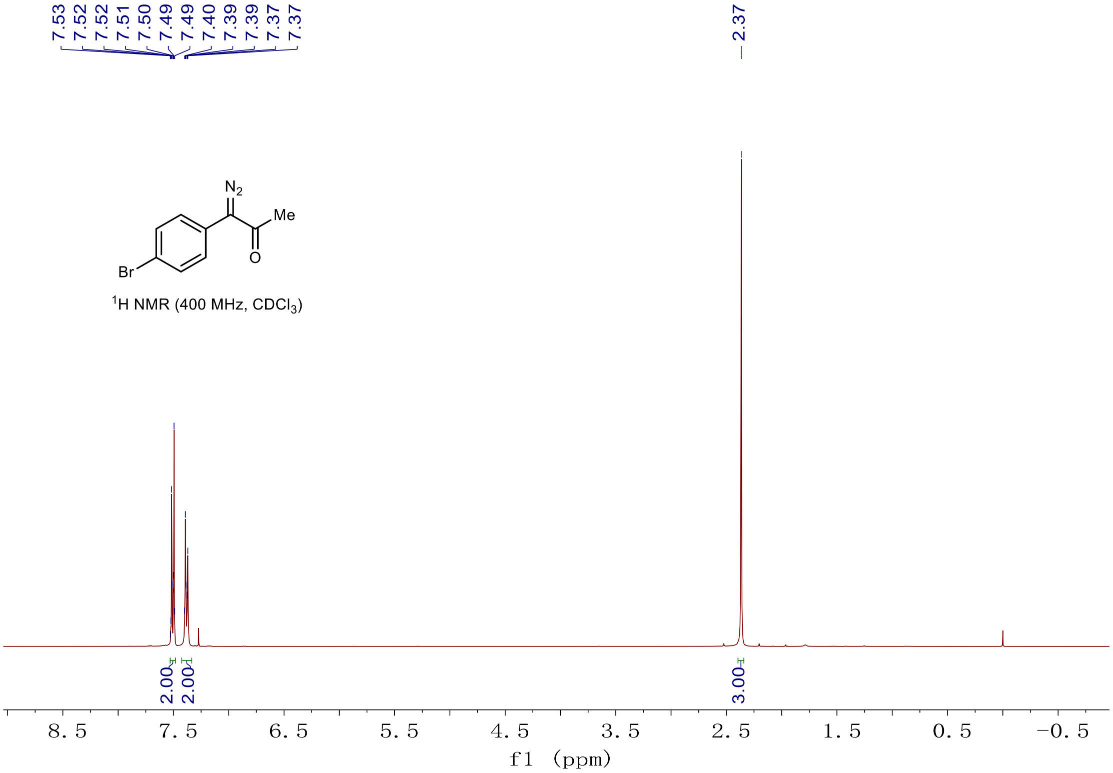

Supplementary Figure 22. ^{1}H NMR spectrum of compound 2i.

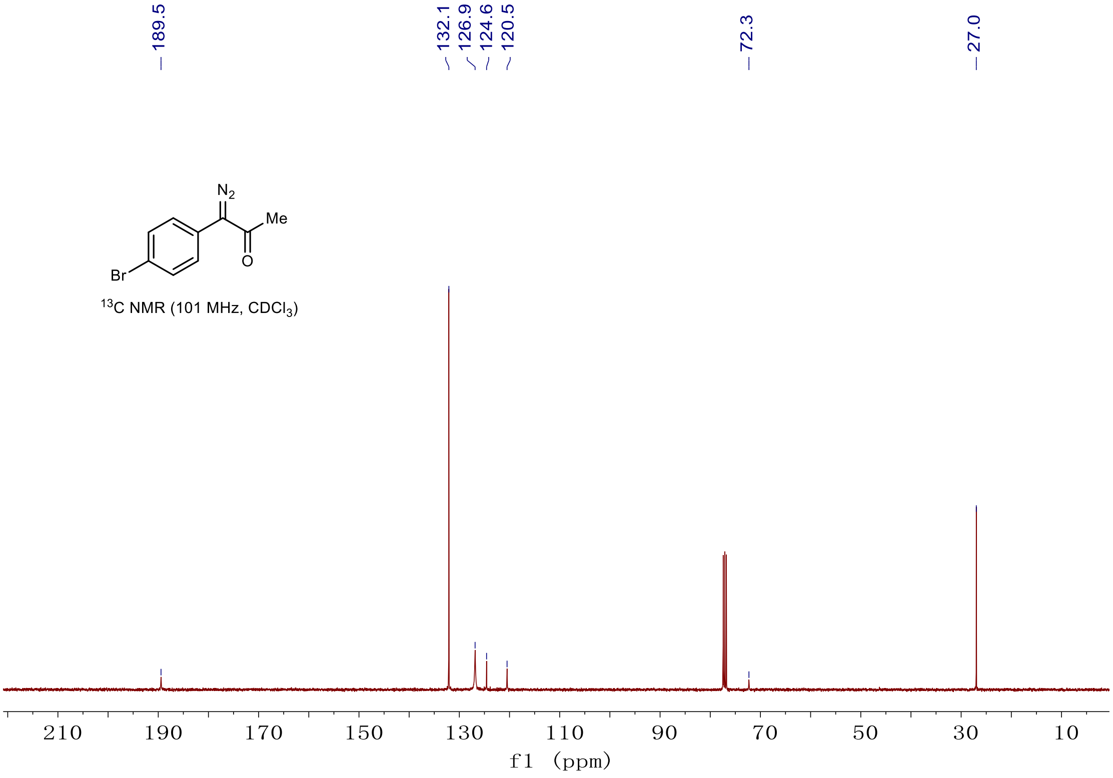

Supplementary Figure 23. ^{13}C NMR spectrum of compound 2i.

1-diazo-1-(4-iodophenyl)propan-2-one (2j)

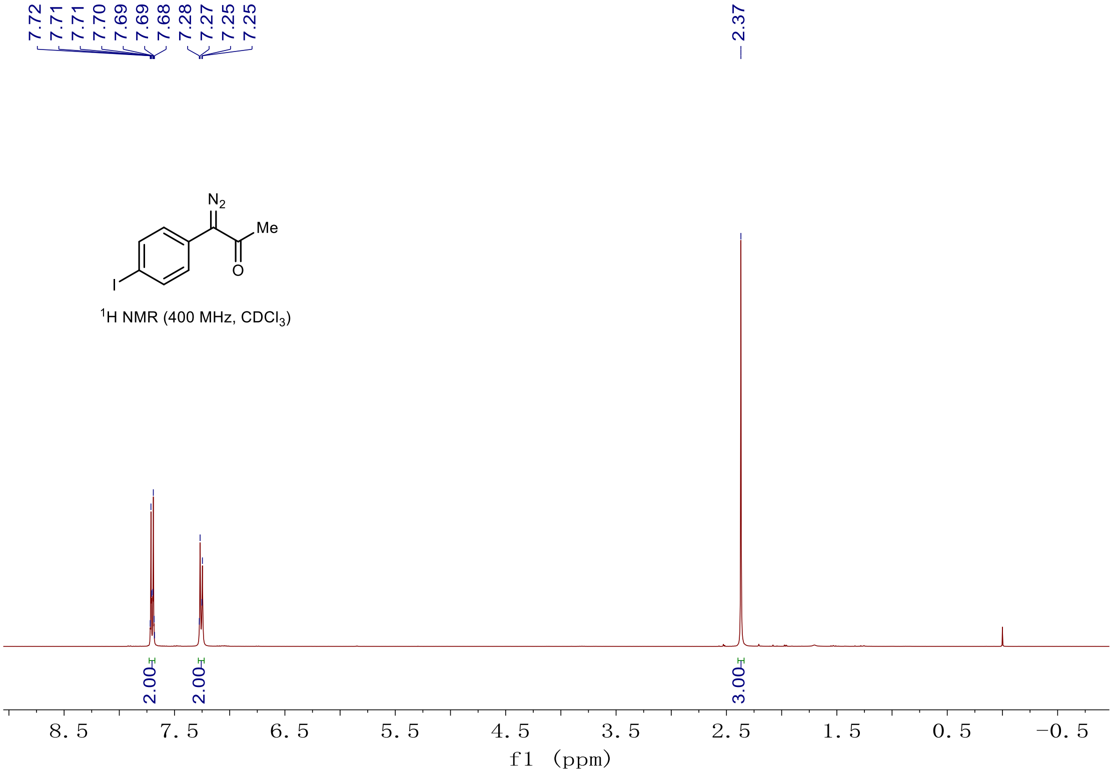

Supplementary Figure 24. ^{1}H NMR spectrum of compound 2j.

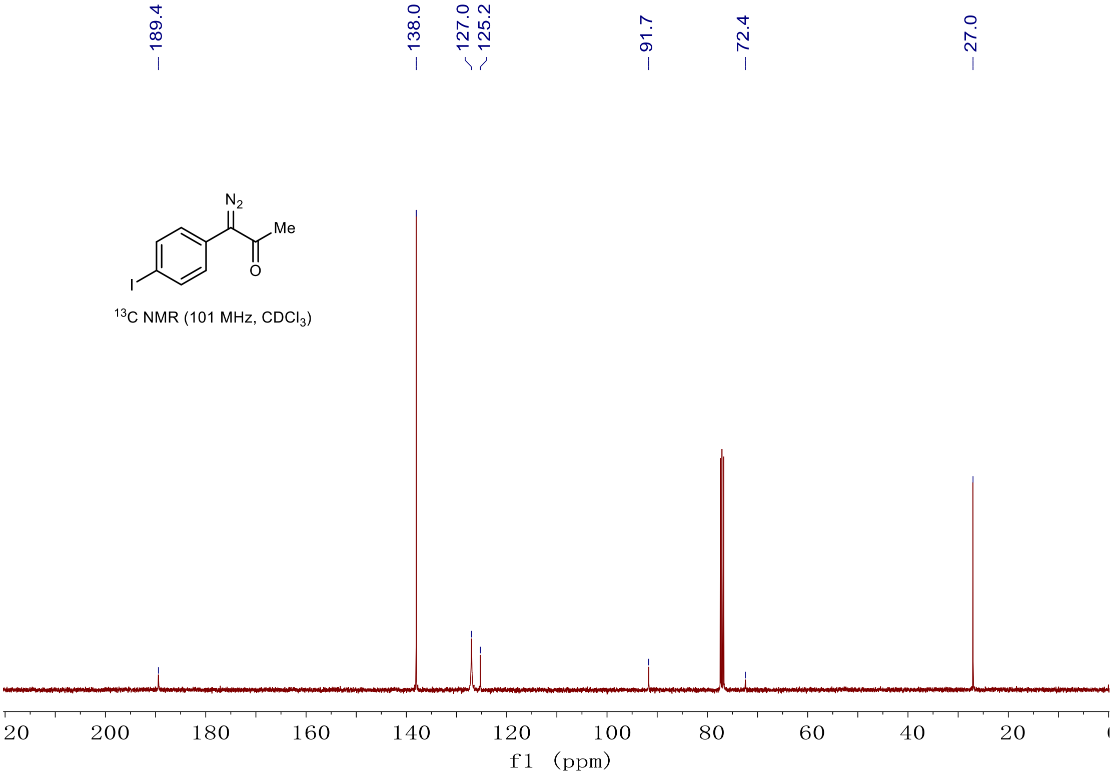

Supplementary Figure 25. ^{13}C NMR spectrum of compound 2j.

1-diazo-1-(naphthalen-2-yl)propan-2-one (2k)

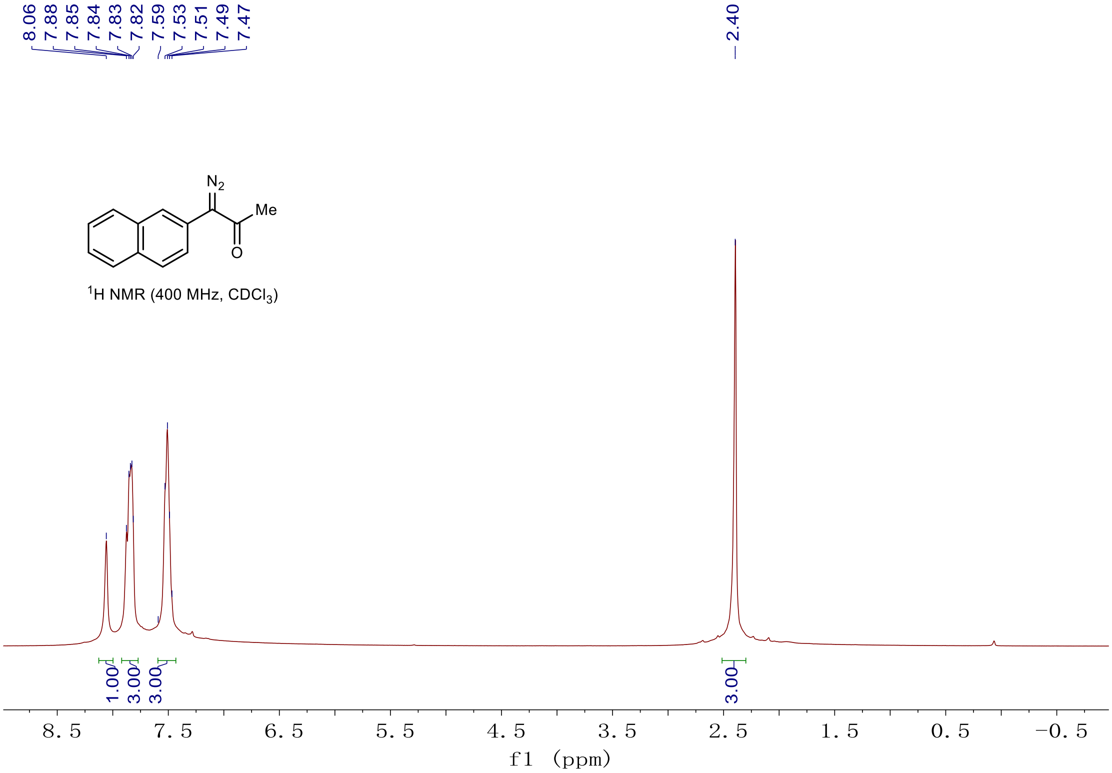

Supplementary Figure 26. ^{1}H NMR spectrum of compound 2k.

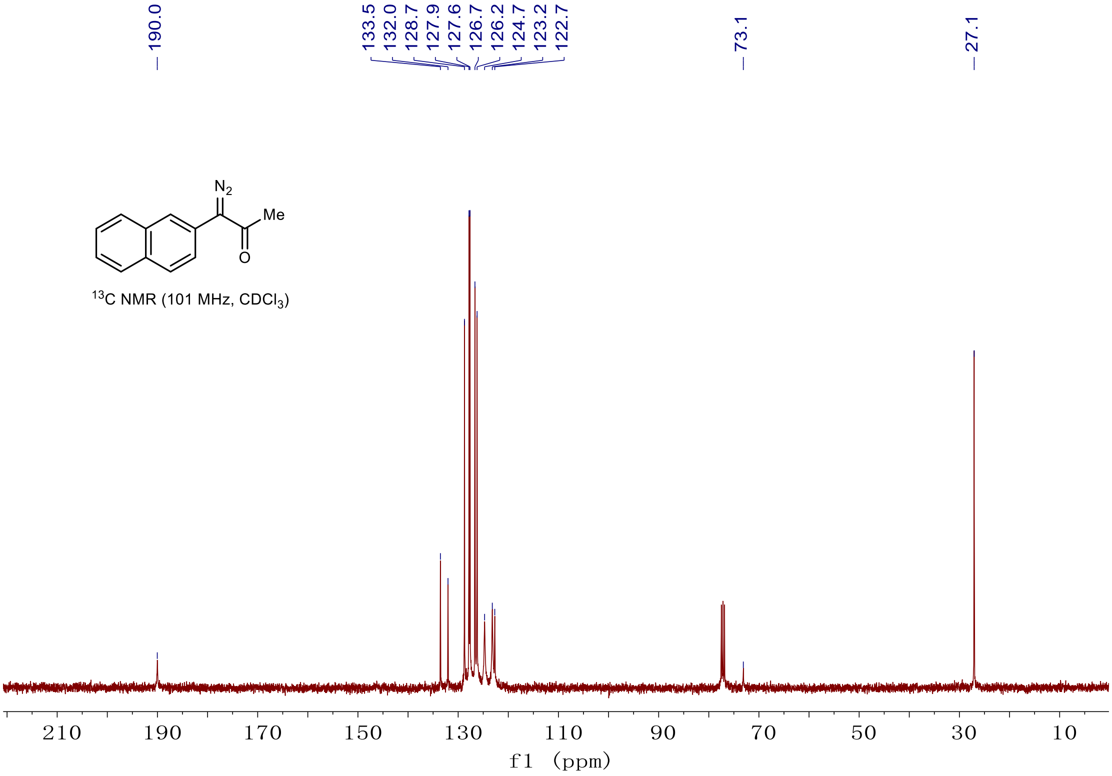

Supplementary Figure 27. ^{13}C NMR spectrum of compound 2k.

1-([1,1'-biphenyl]-4-yl)-1-diazopropan-2-one (2l)

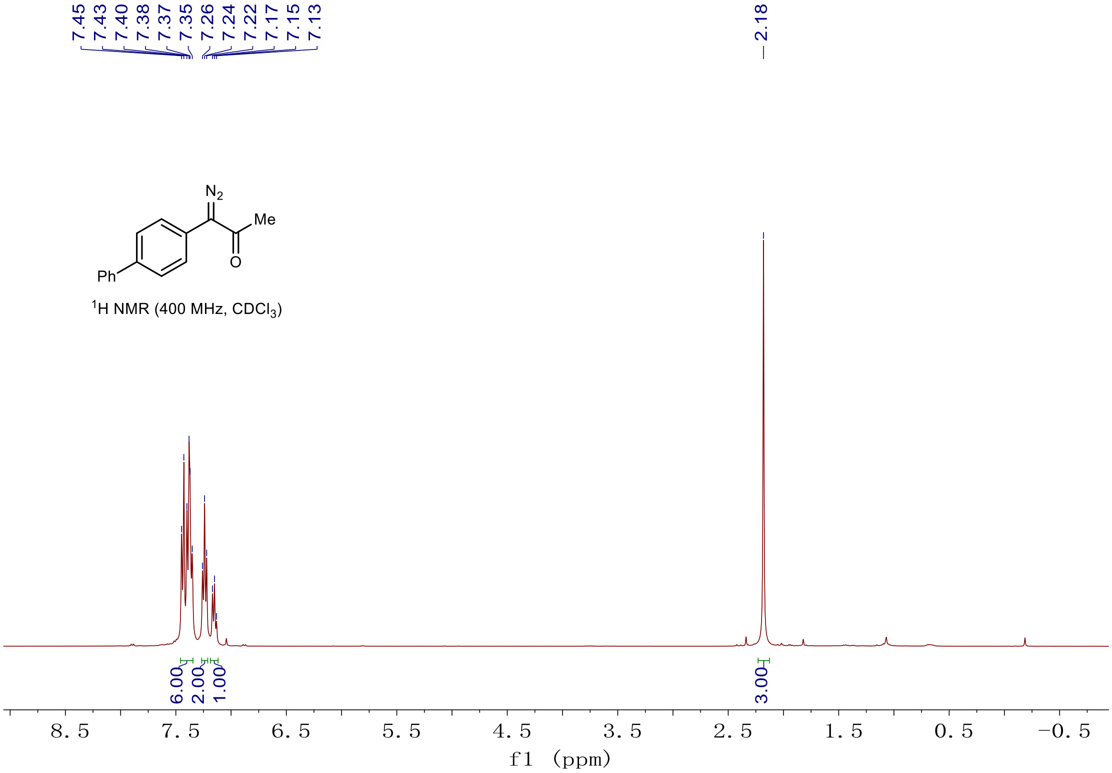

Supplementary Figure 28. ^{1}H NMR spectrum of compound 2l.

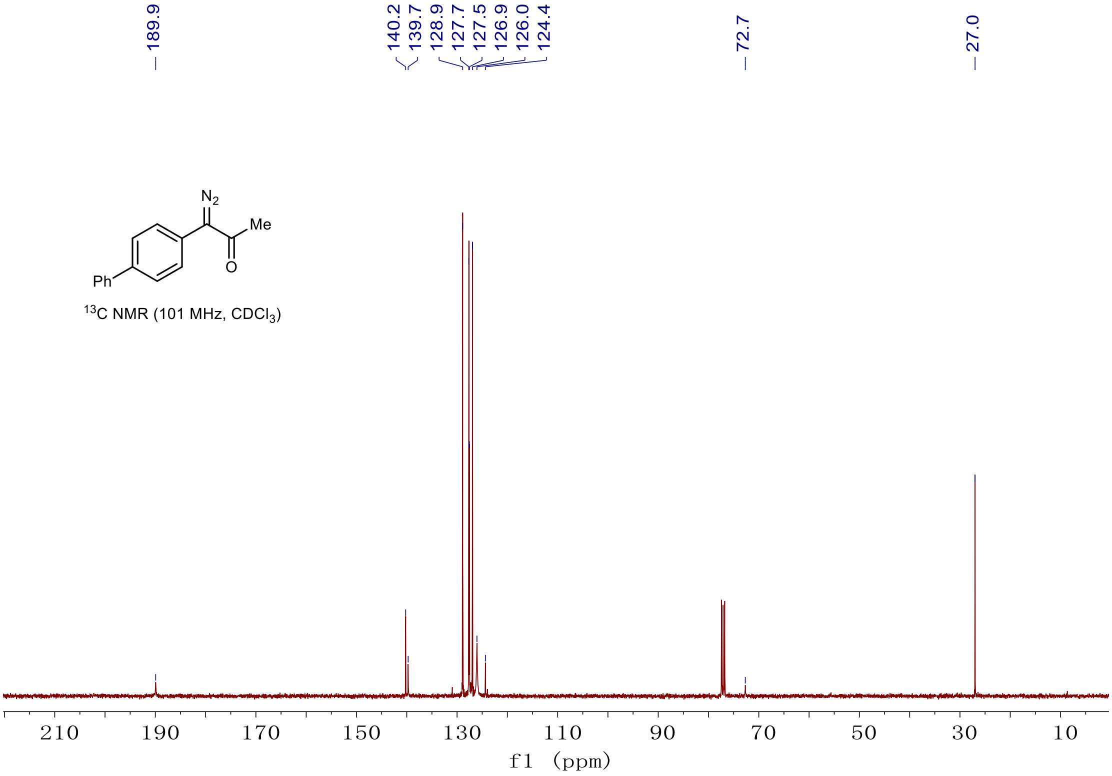

Supplementary Figure 29. ^{13}C NMR spectrum of compound 2l.

1-diazo-1-phenylbutan-2-one (2m)

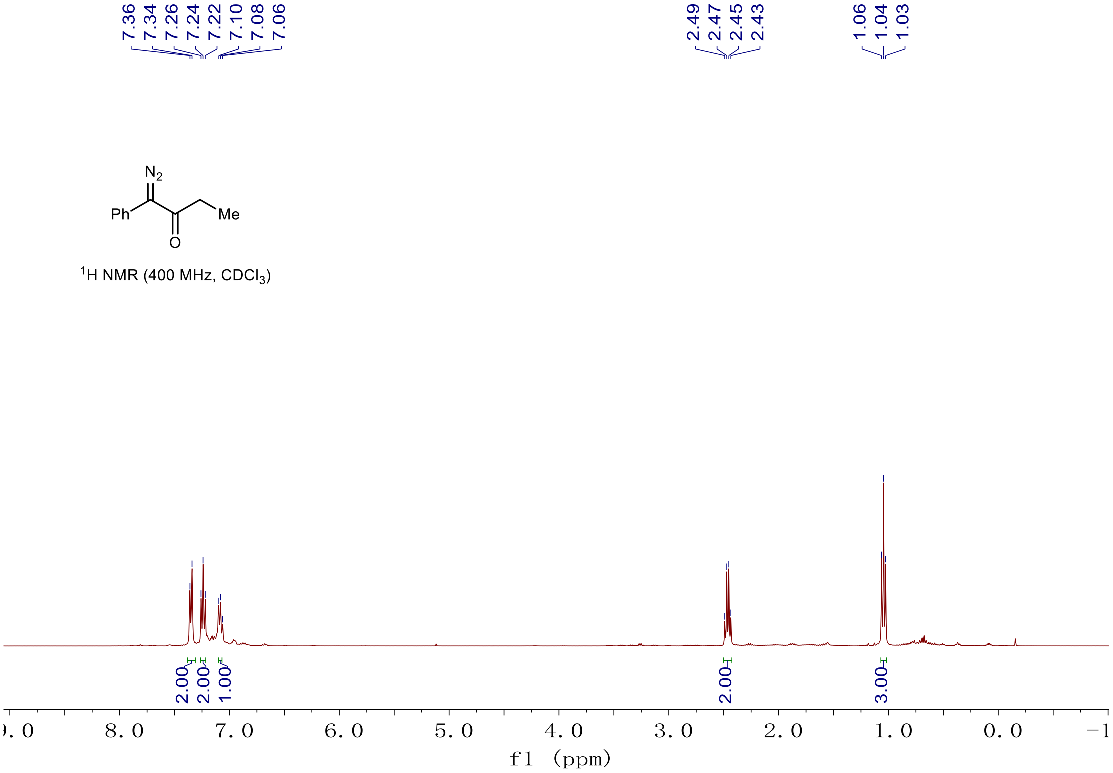

Supplementary Figure 30. ^{1}H NMR spectrum of compound 2m.

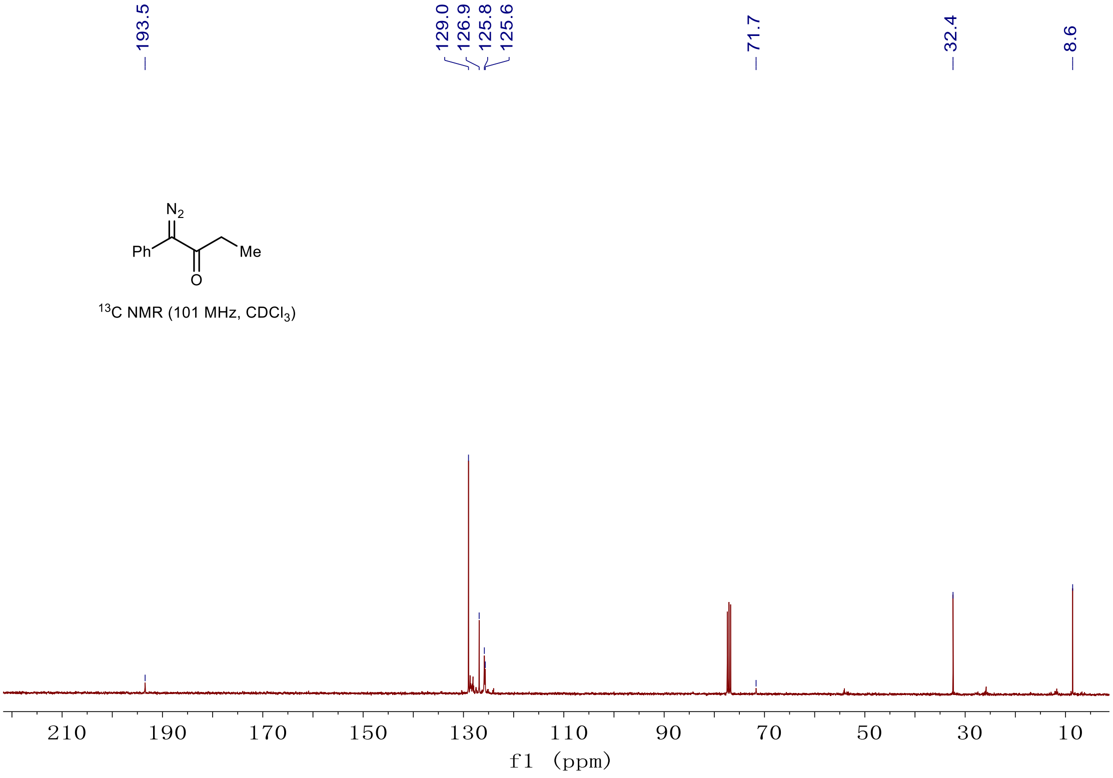

Supplementary Figure 31. ^{13}C NMR spectrum of compound 2m.

(*R*)-N-(2-oxo-1-phenylpropyl)-3-phenylpropanamide (3a)

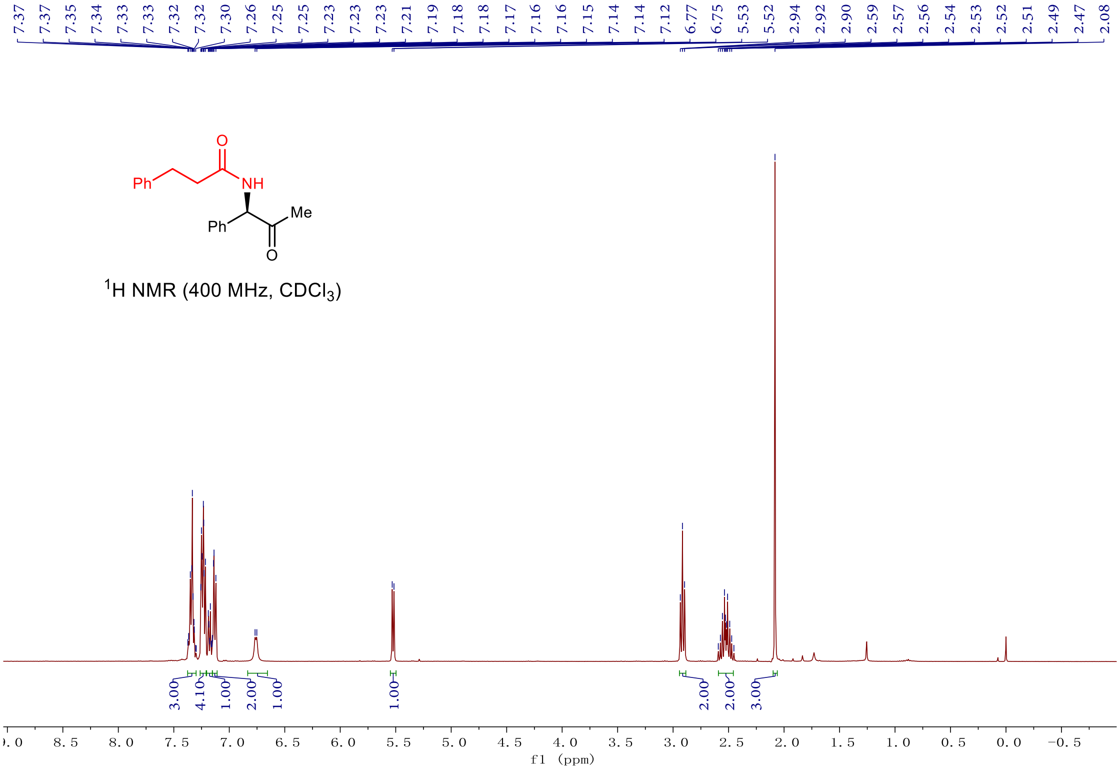

Supplementary Figure 32. ^{1}**H NMR spectrum of compound 3a.**

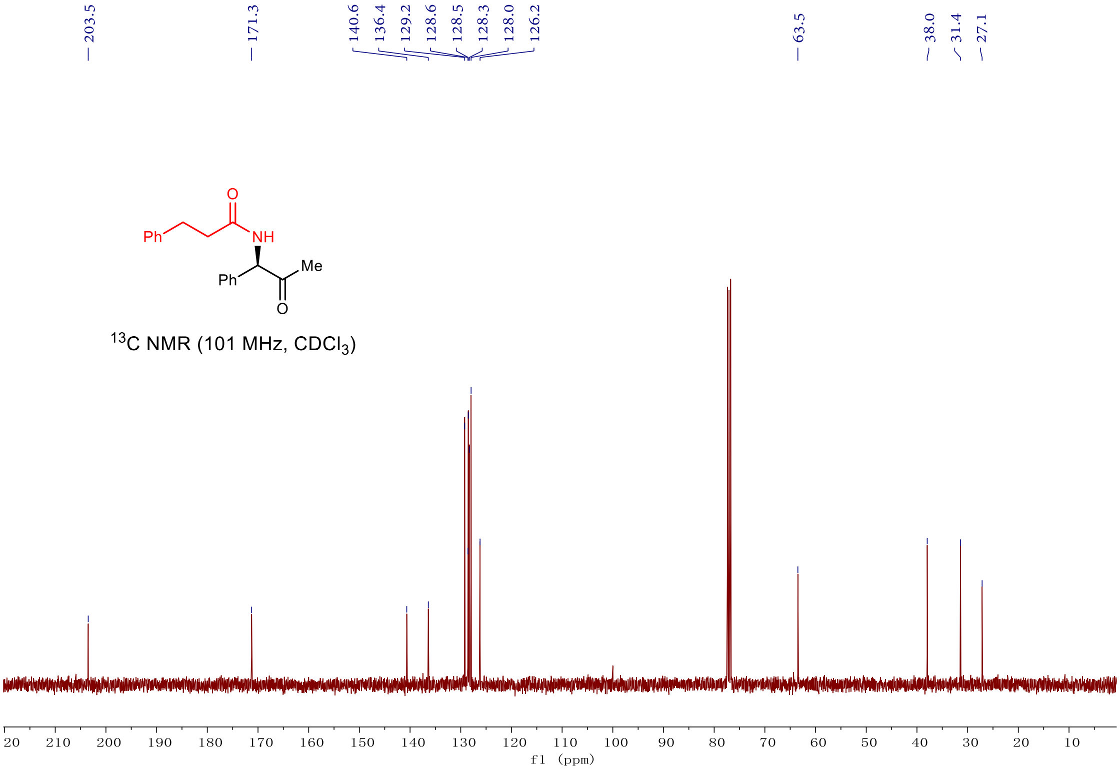

Supplementary Figure 33. ^{13}**C NMR spectrum of compound 3a.**

(*R*)-N-(2-oxo-1-phenylpropyl)-2-phenylacetamide (3b)

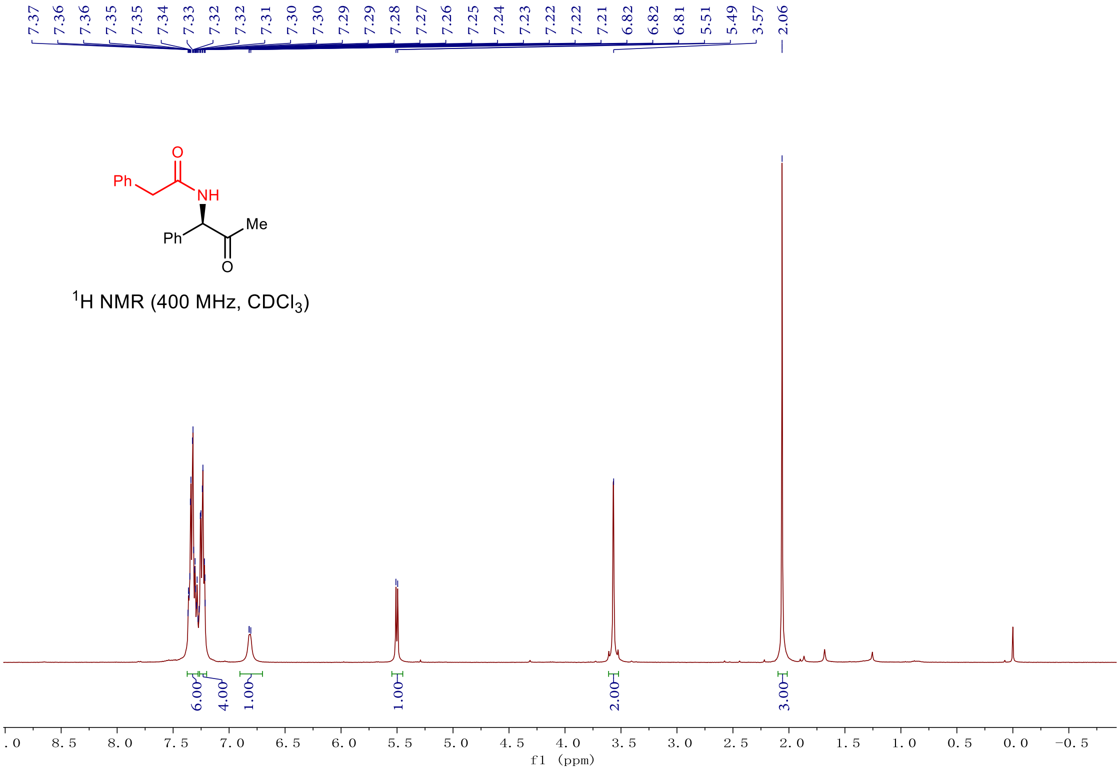

Supplementary Figure 34. ^{1}H NMR spectrum of compound 3b.

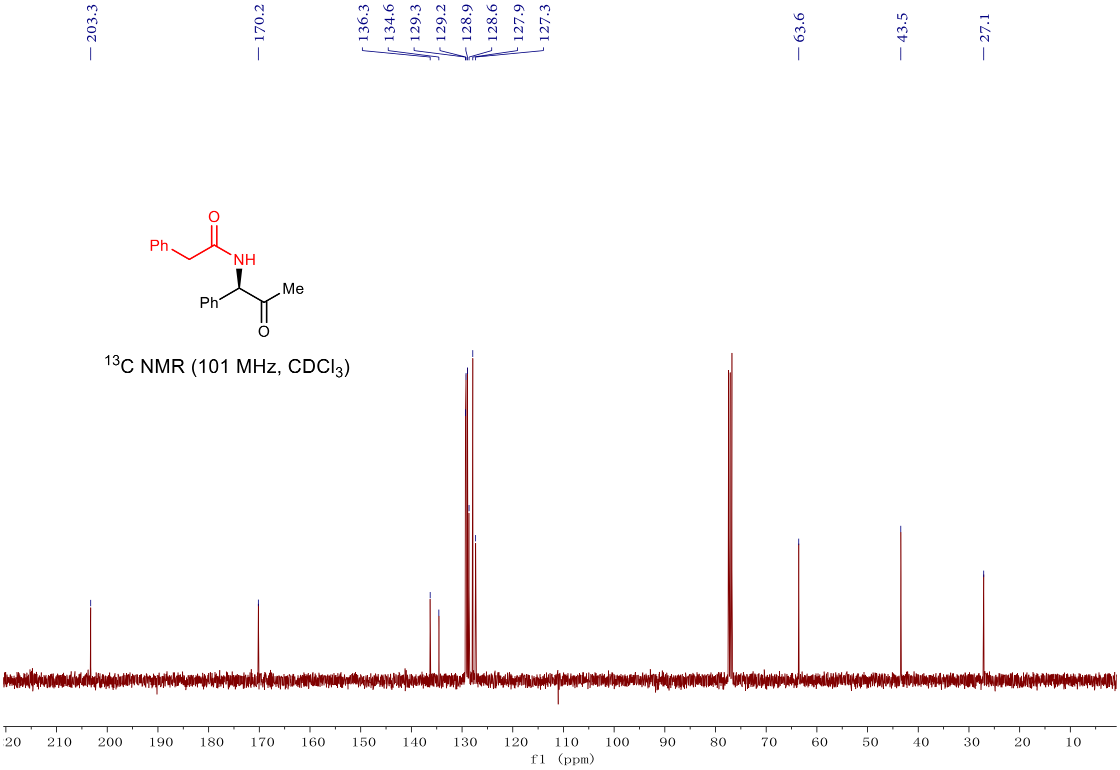

Supplementary Figure 35. ^{13}C NMR spectrum of compound 3b.

(*R*)-N-(2-oxo-1-phenylpropyl)acetamide (3c)

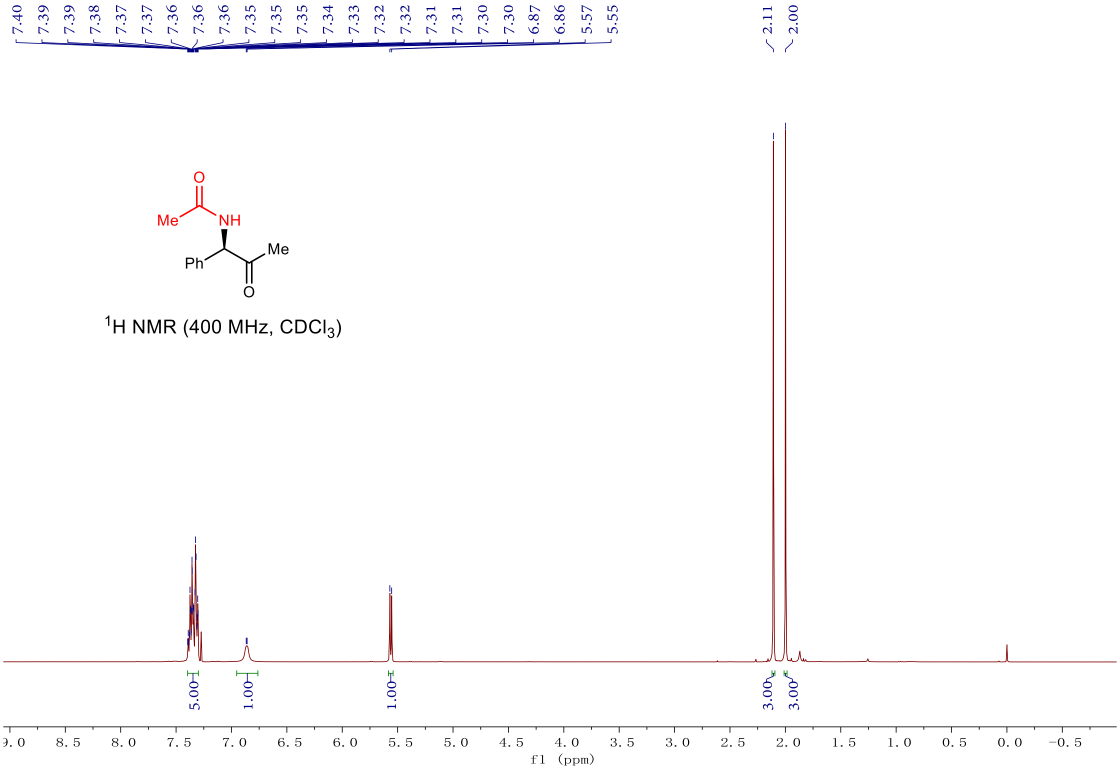

Supplementary Figure 36. ^{1}H NMR spectrum of compound 3c.

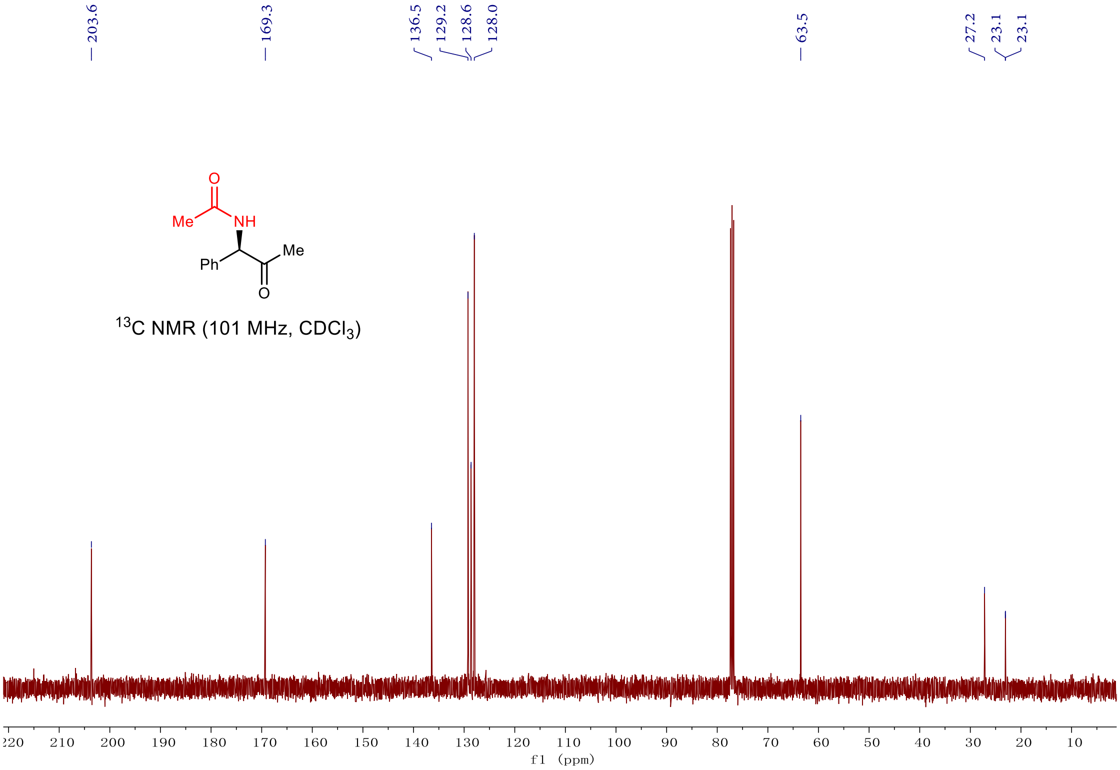

Supplementary Figure 37. ^{13}C NMR spectrum of compound 3c.

(*R*)-N-(2-oxo-1-phenylpropyl)propionamide (3d)

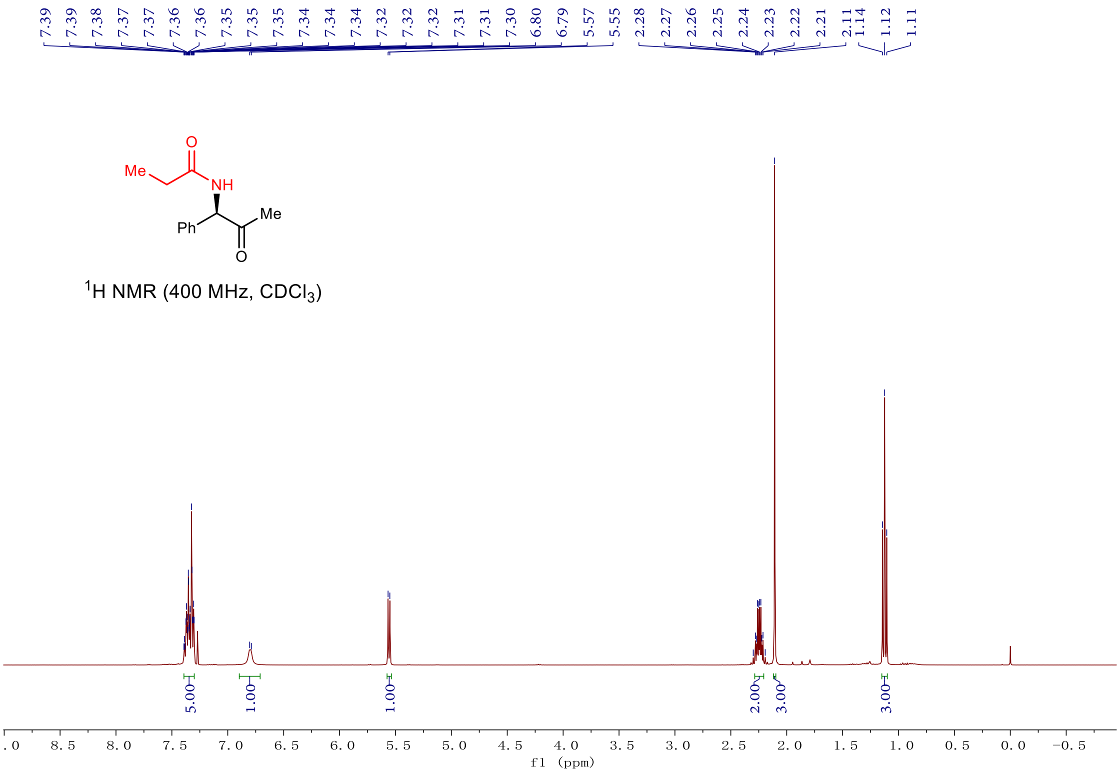

Supplementary Figure 38. ^{1}H NMR spectrum of compound 3d.

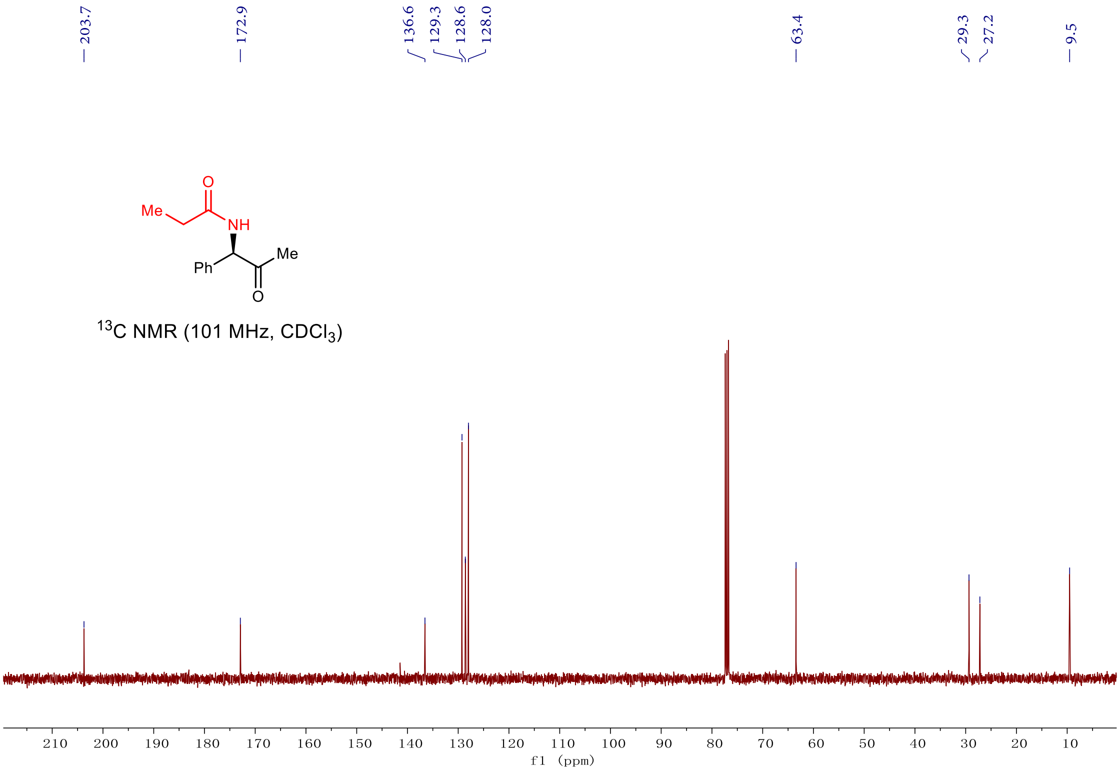

Supplementary Figure 39. ^{13}C NMR spectrum of compound 3d.

(*R*)-N-(2-oxo-1-phenylpropyl)butyramide (3e)

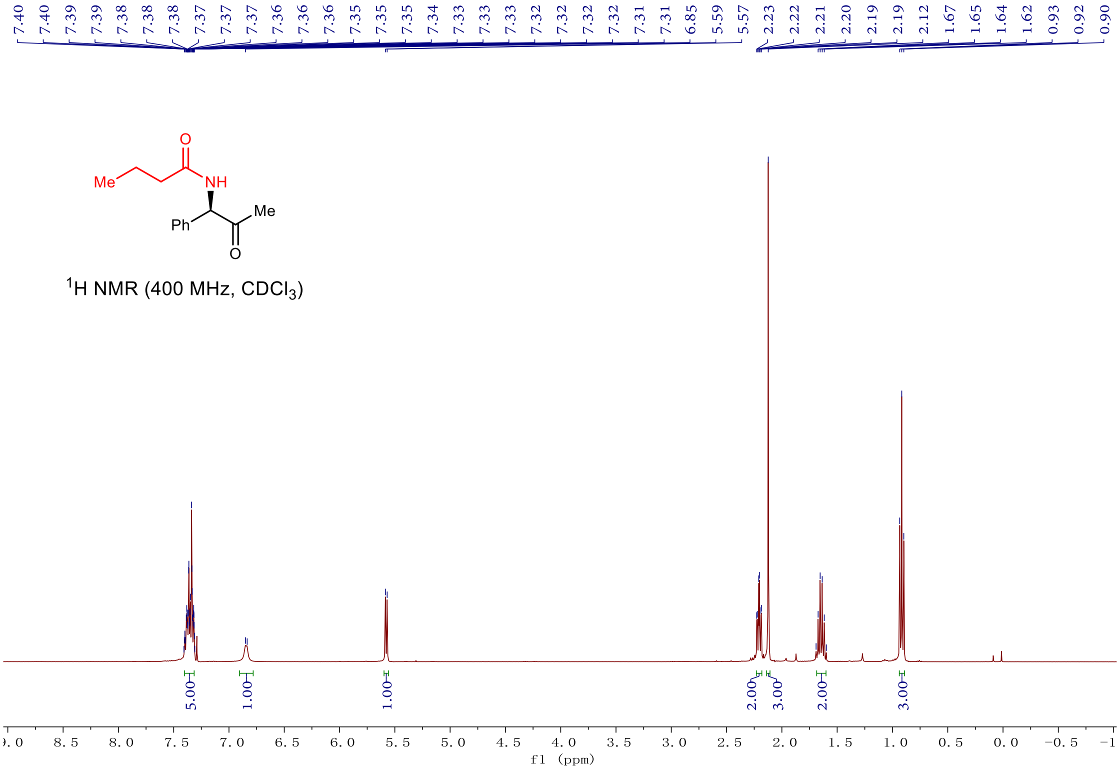

Supplementary Figure 40. ^{1}H NMR spectrum of compound 3e.

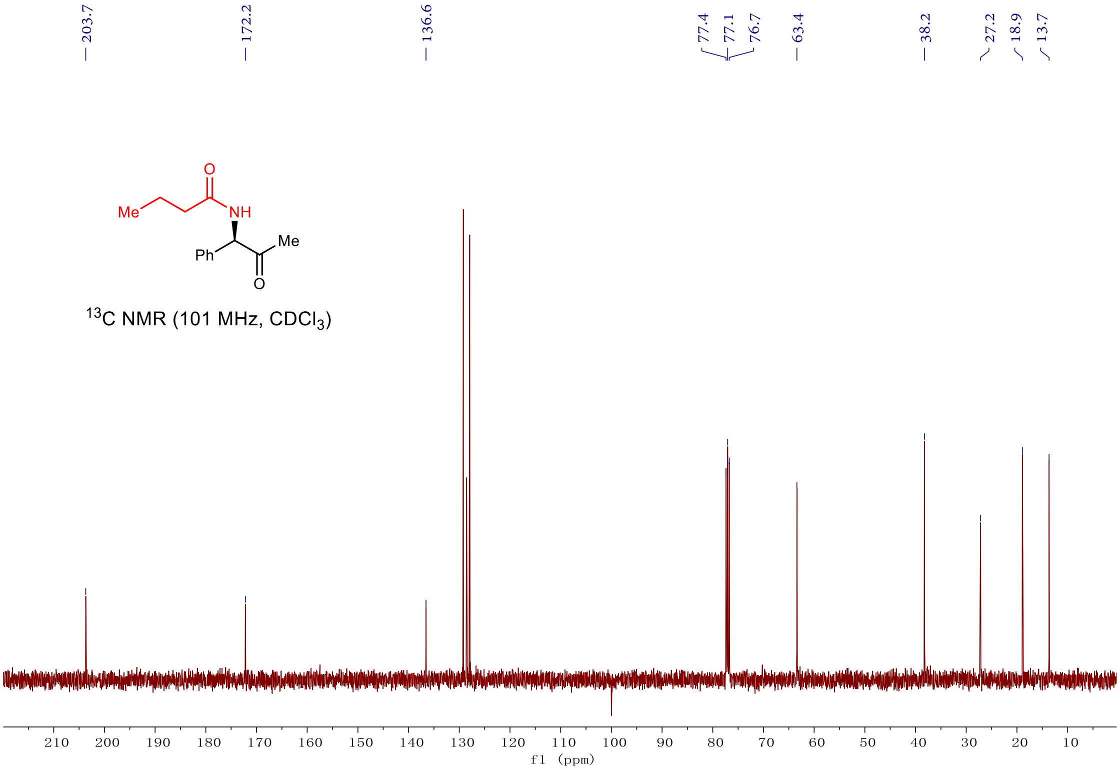

Supplementary Figure 41. ^{13}C NMR spectrum of compound 3e.

(*R*)-N-(2-oxo-1-phenylpropyl)isobutyramide (3f)

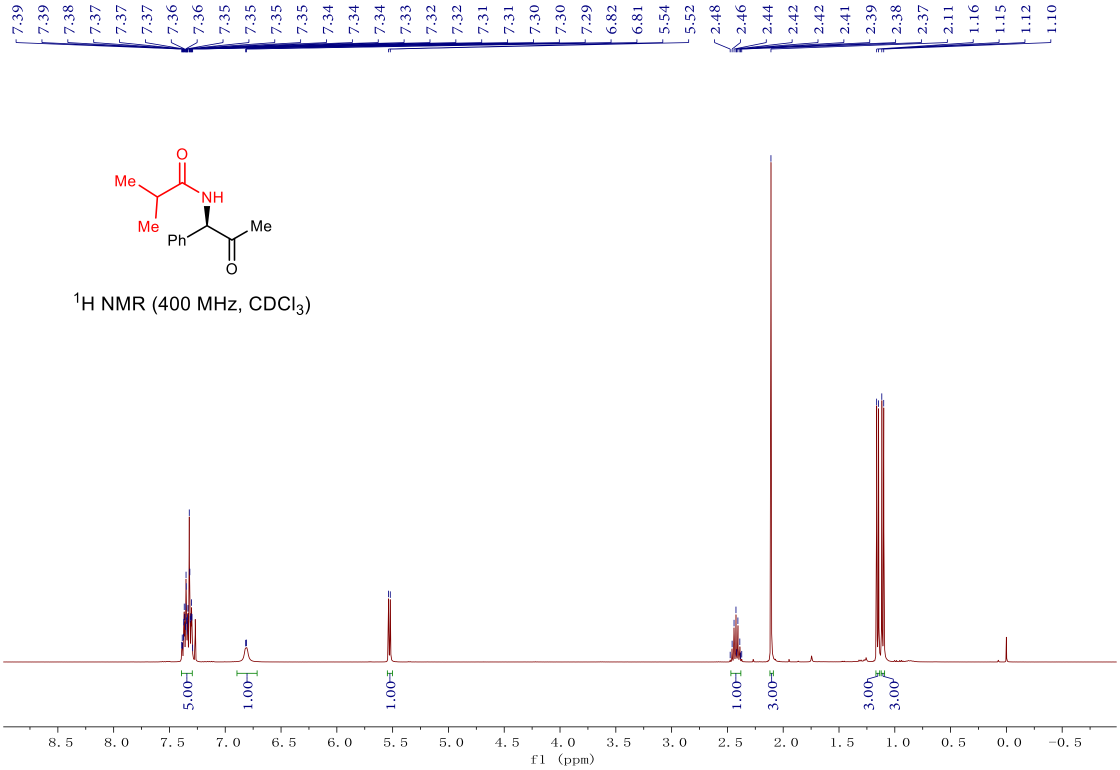

Supplementary Figure 42. ^{1}H NMR spectrum of compound 3f.

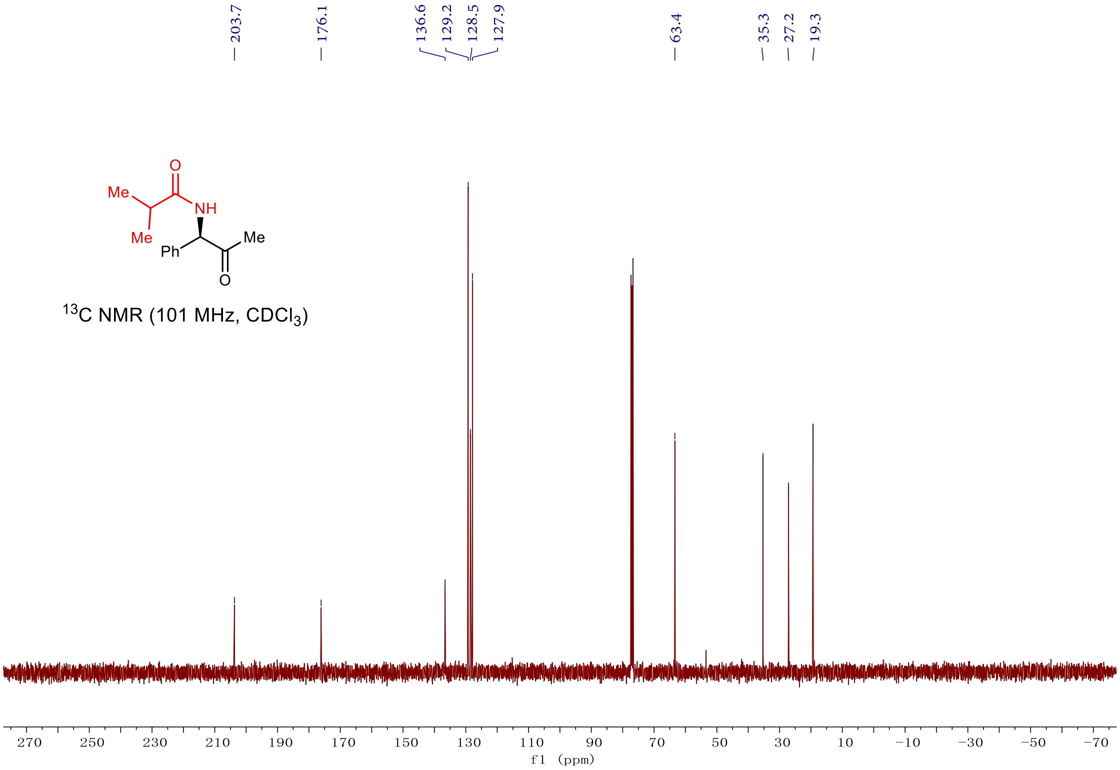

Supplementary Figure 43. ^{13}C NMR spectrum of compound 3f.

(*R*)-N-(2-oxo-1-phenylpropyl)pivalamide (3g)

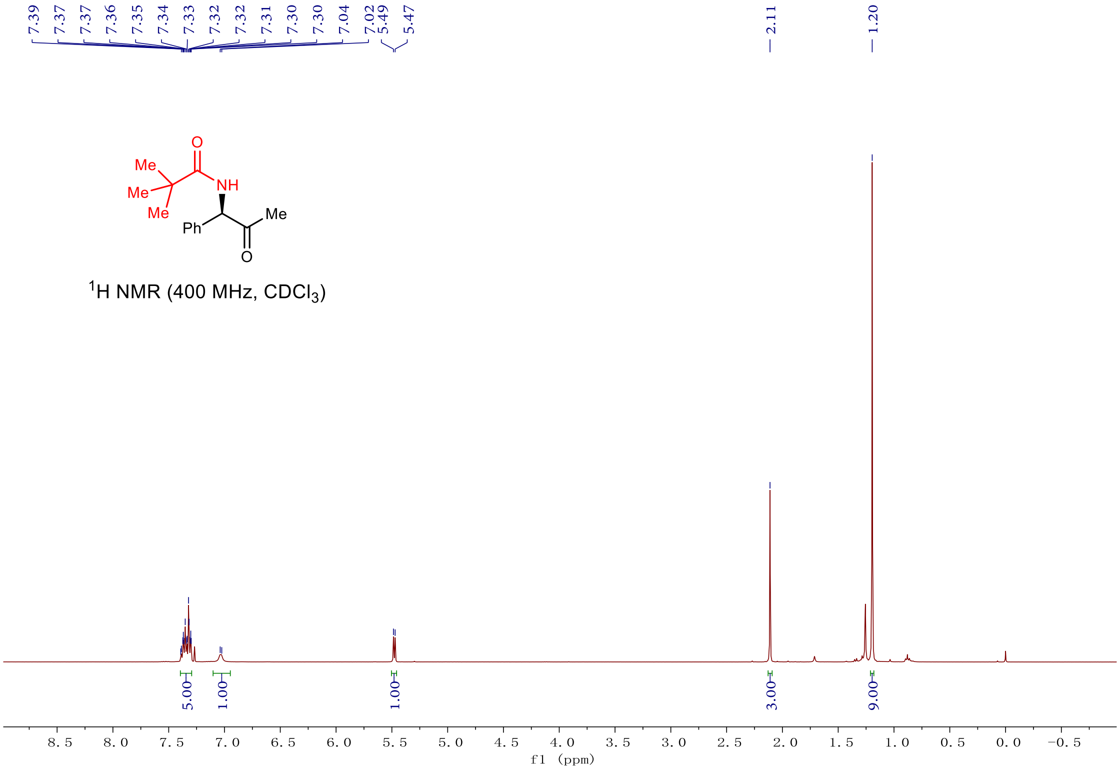

Supplementary Figure 44. ^{1}H NMR spectrum of compound 3g.

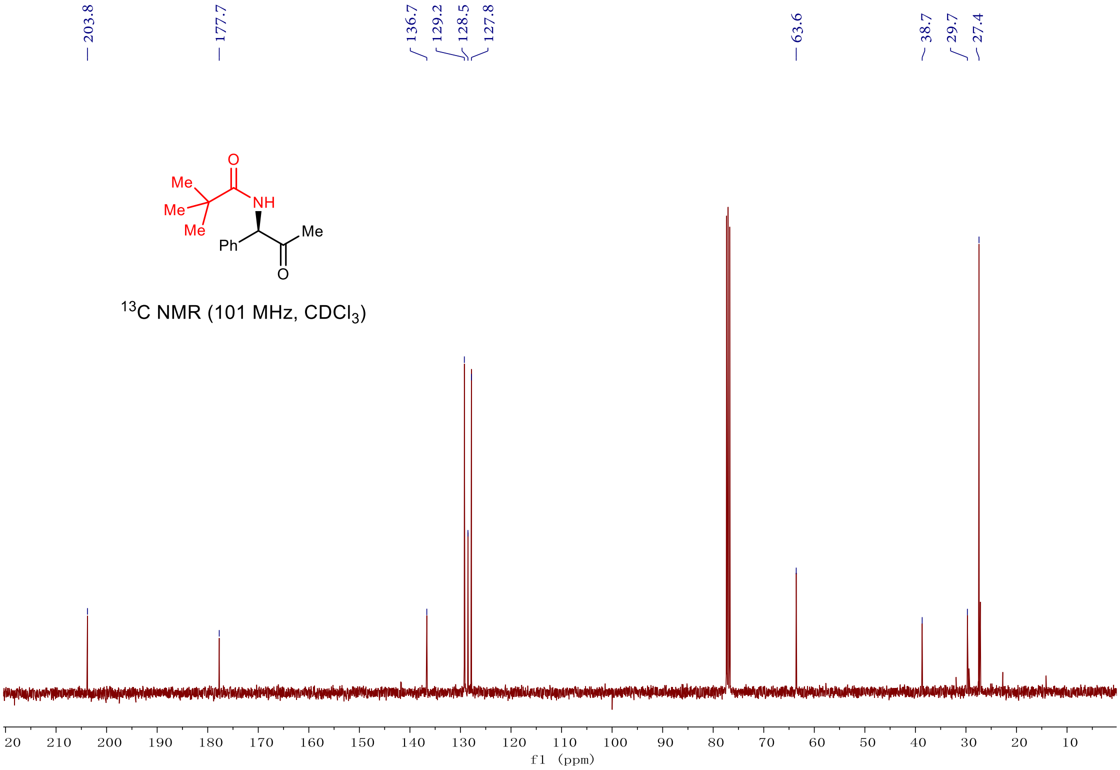

Supplementary Figure 45. ^{13}C NMR spectrum of compound 3g.

Methyl (*R*)-3-((2-oxo-1-phenylpropyl)carbamoyl)bicyclo[1.1.1]pentane-1-carboxylate (3h)

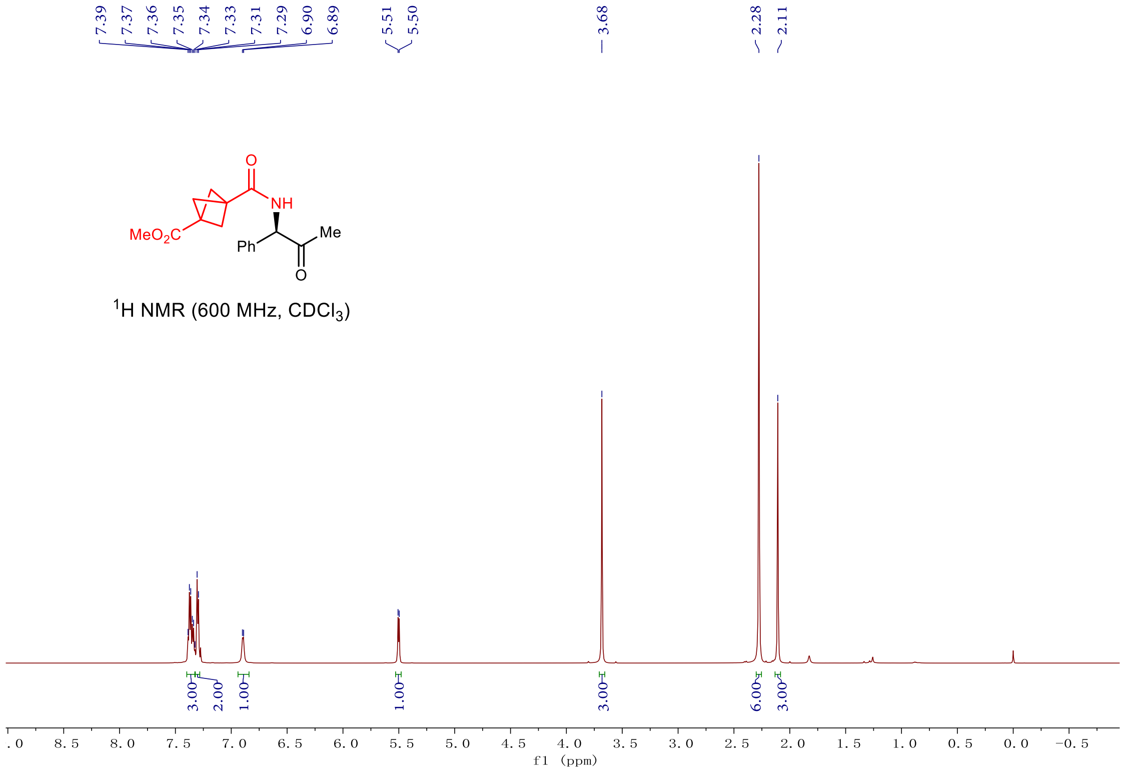

Supplementary Figure 46. ^{1}H NMR spectrum of compound 3h.

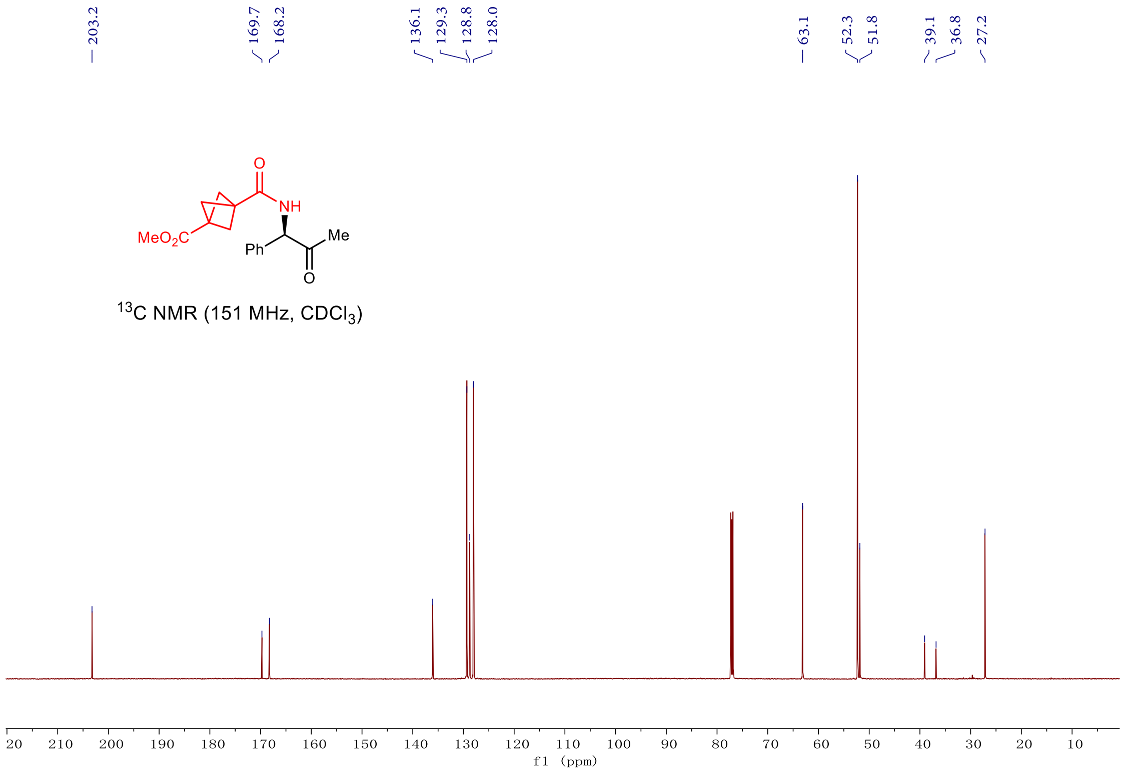

Supplementary Figure 47. ^{13}C NMR spectrum of compound 3h.

(*R*)-N-(2-oxo-1-phenylpropyl)cyclopropanecarboxamide (3i)

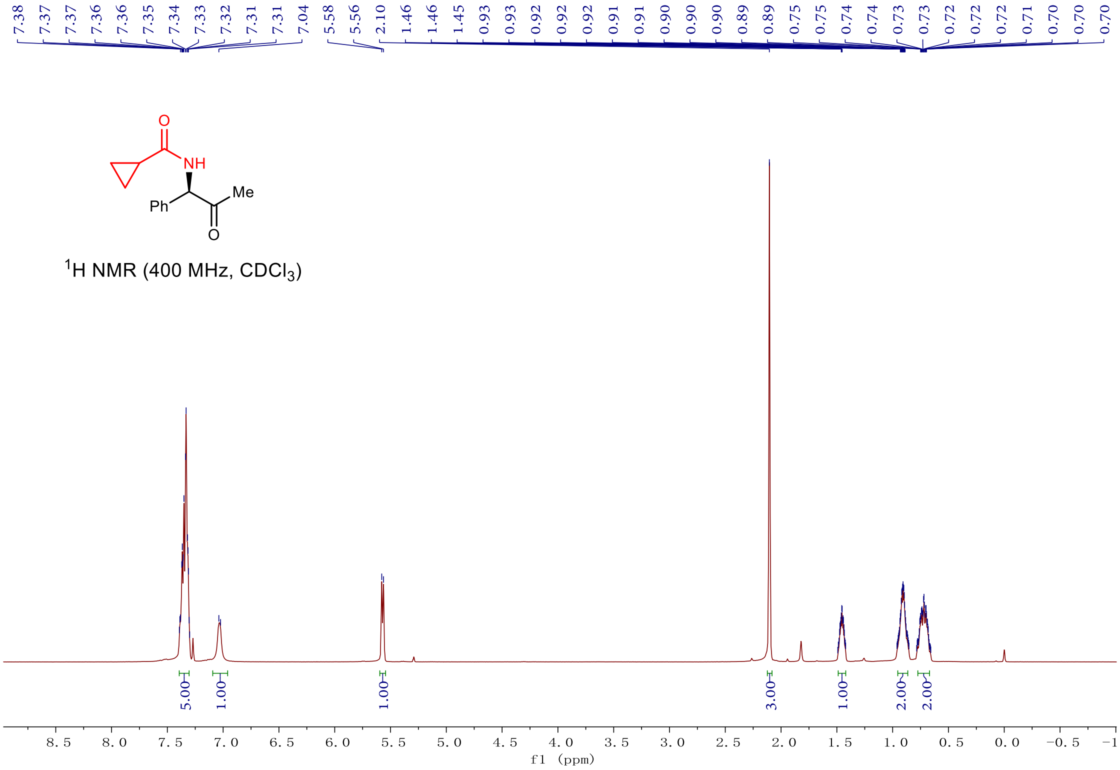

Supplementary Figure 48. ^{1}H NMR spectrum of compound 3i.

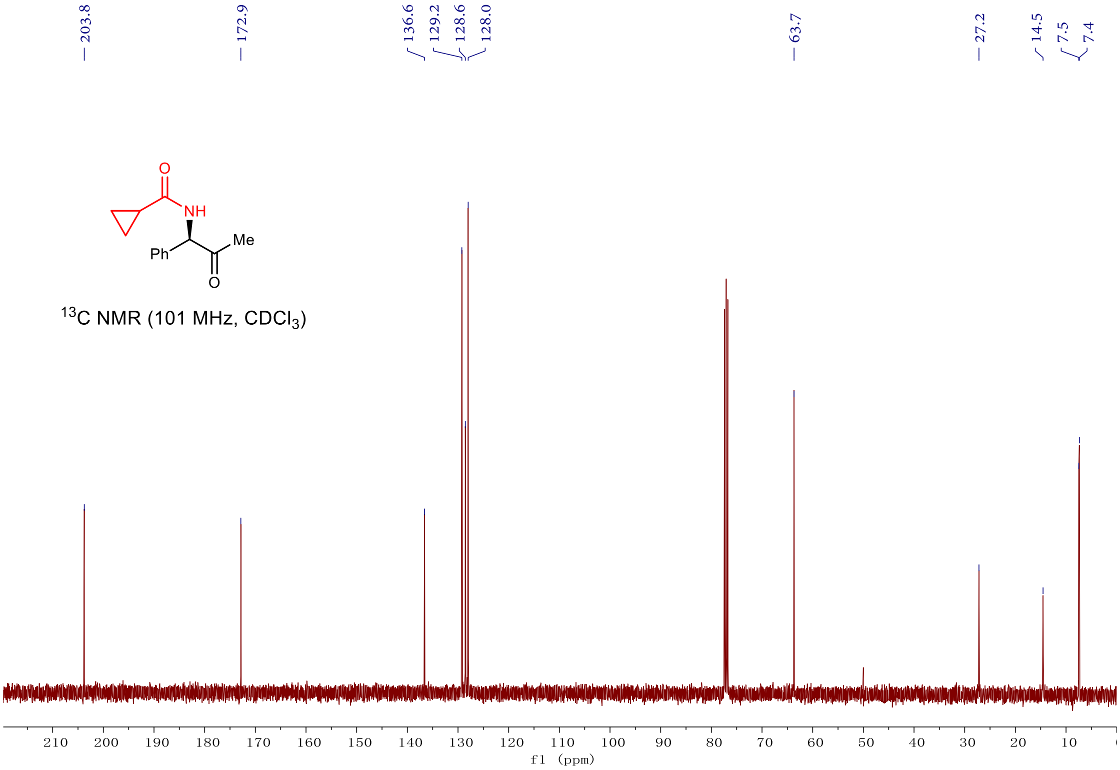

Supplementary Figure 49. ^{13}C NMR spectrum of compound 3i.

(*R*)-N-(2-oxo-1-phenylpropyl)cyclobutanecarboxamide (3j)

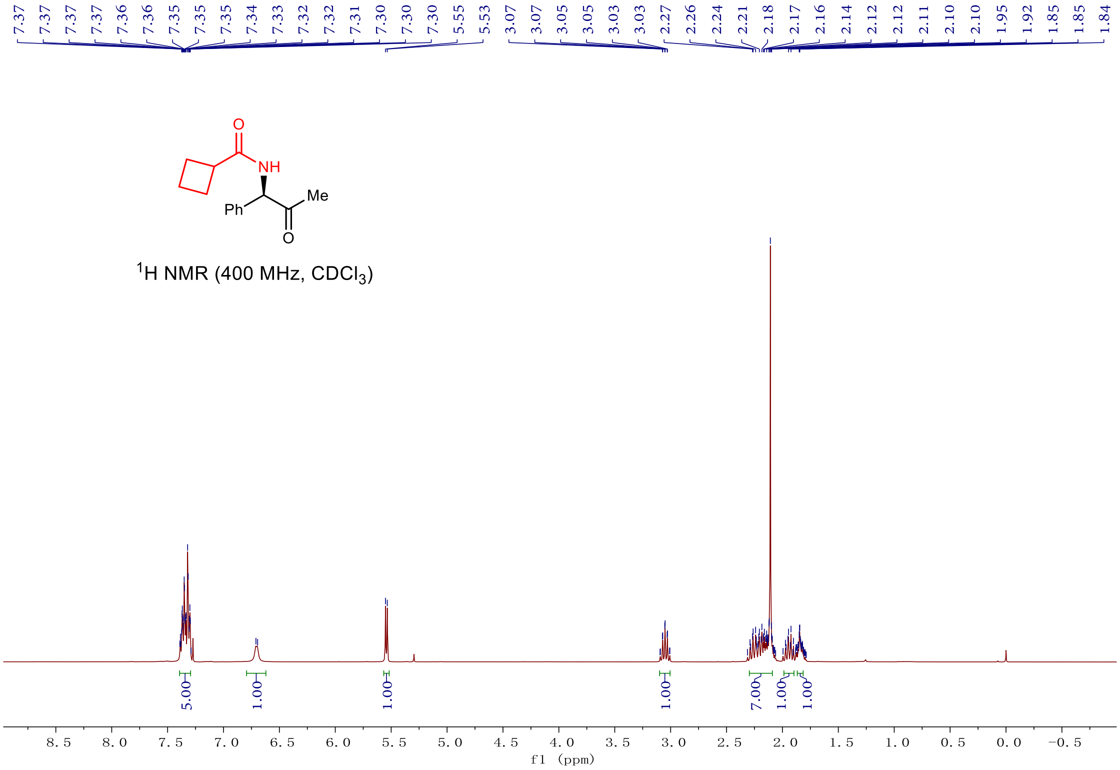

Supplementary Figure 50. ^{1}H NMR spectrum of compound 3j.

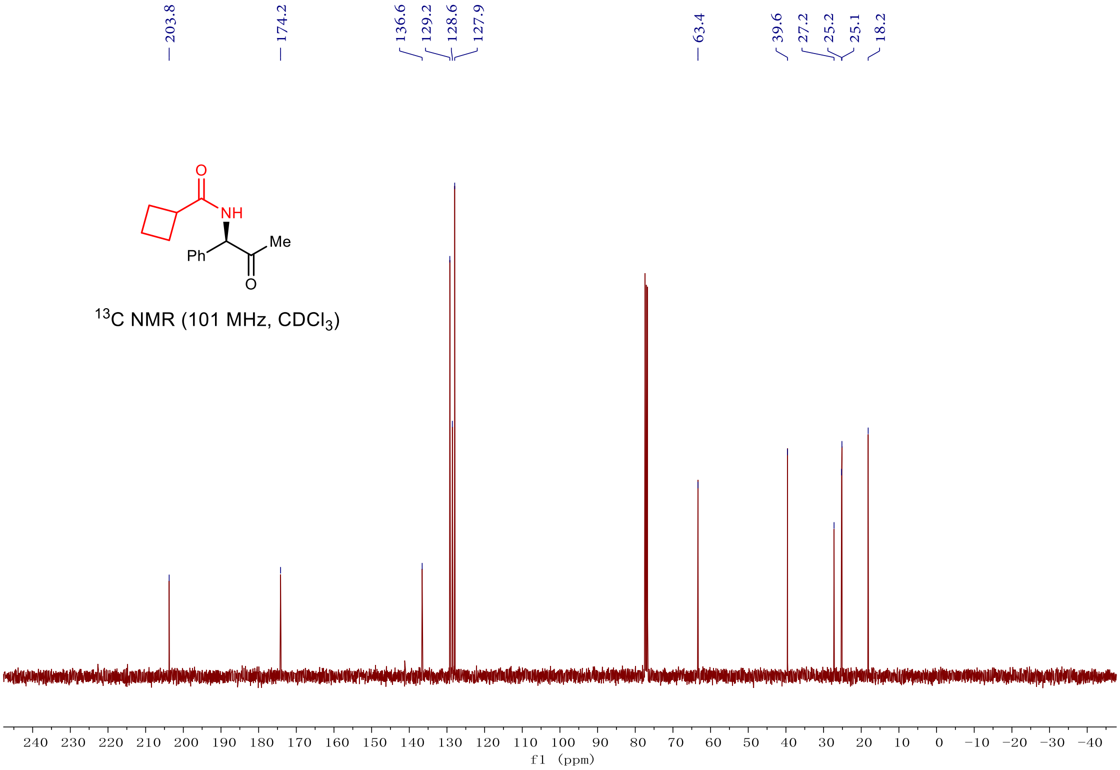

Supplementary Figure 51. ^{13}C NMR spectrum of compound 3j.

(*R*)-N-(2-oxo-1-phenylpropyl)cyclohexanecarboxamide (3k)

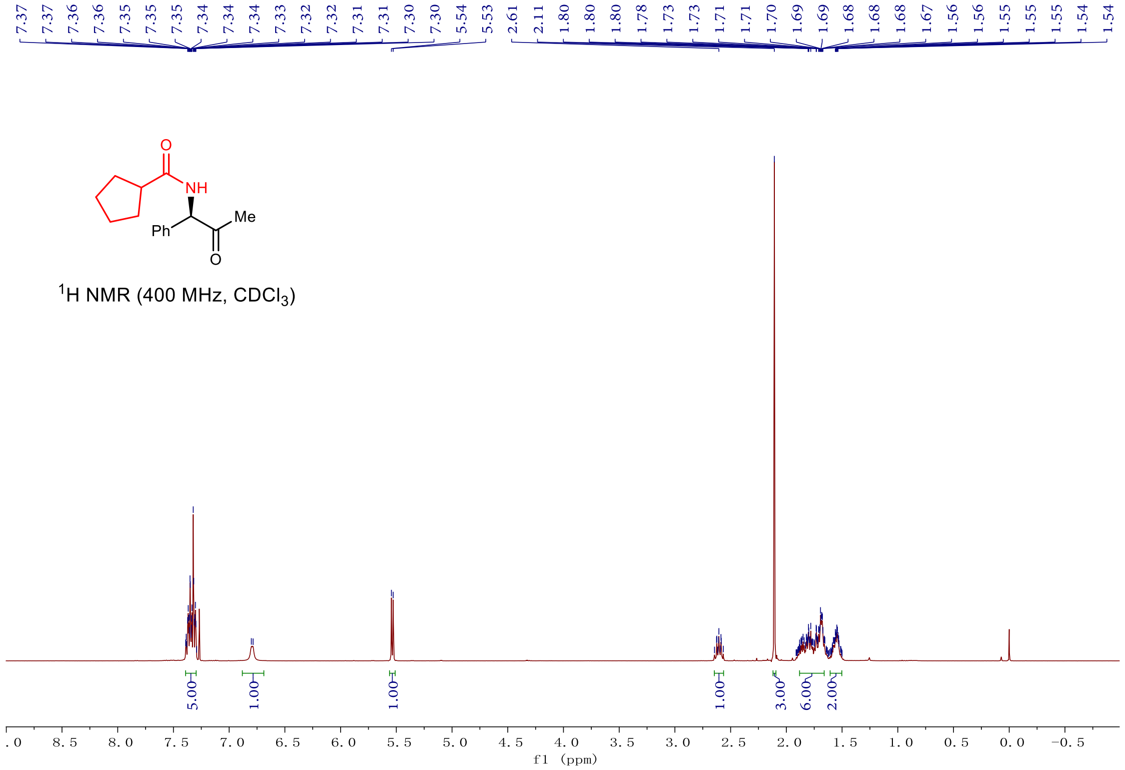

Supplementary Figure 52. ^{1}H NMR spectrum of compound 3k.

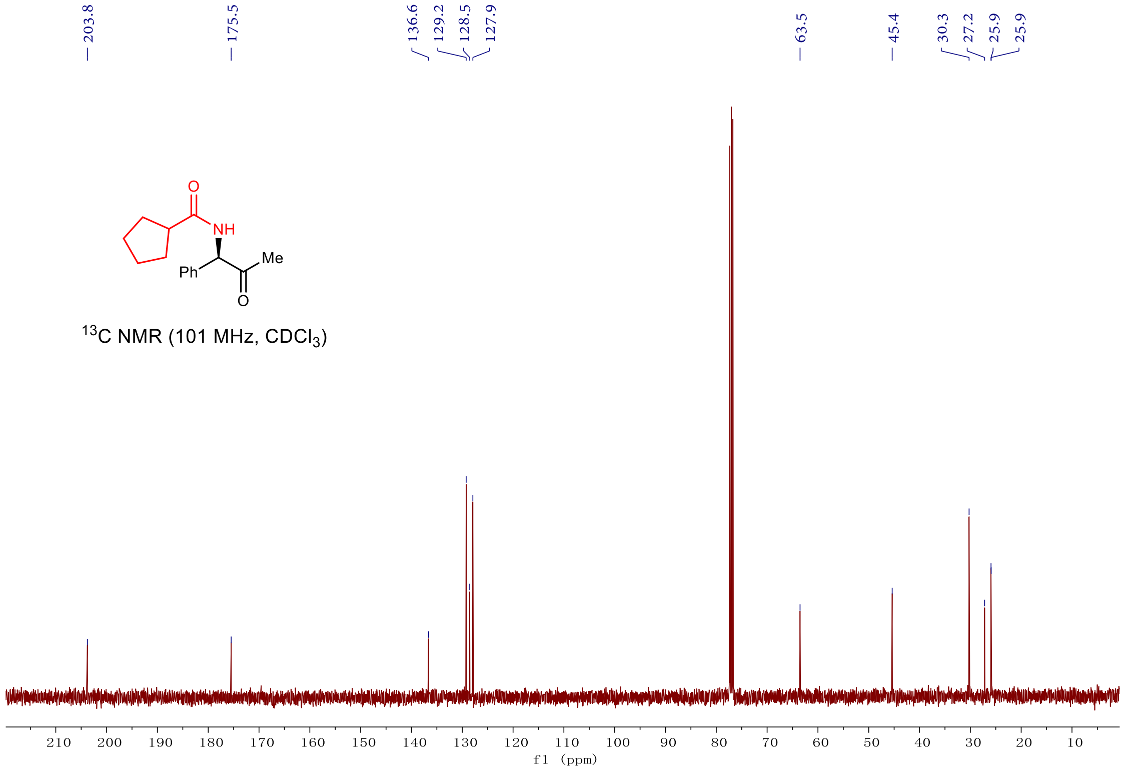

Supplementary Figure 53. ^{13}C NMR spectrum of compound 3k.

(*R*)-N-(2-oxo-1-phenylpropyl)cyclohexanecarboxamide (3l)

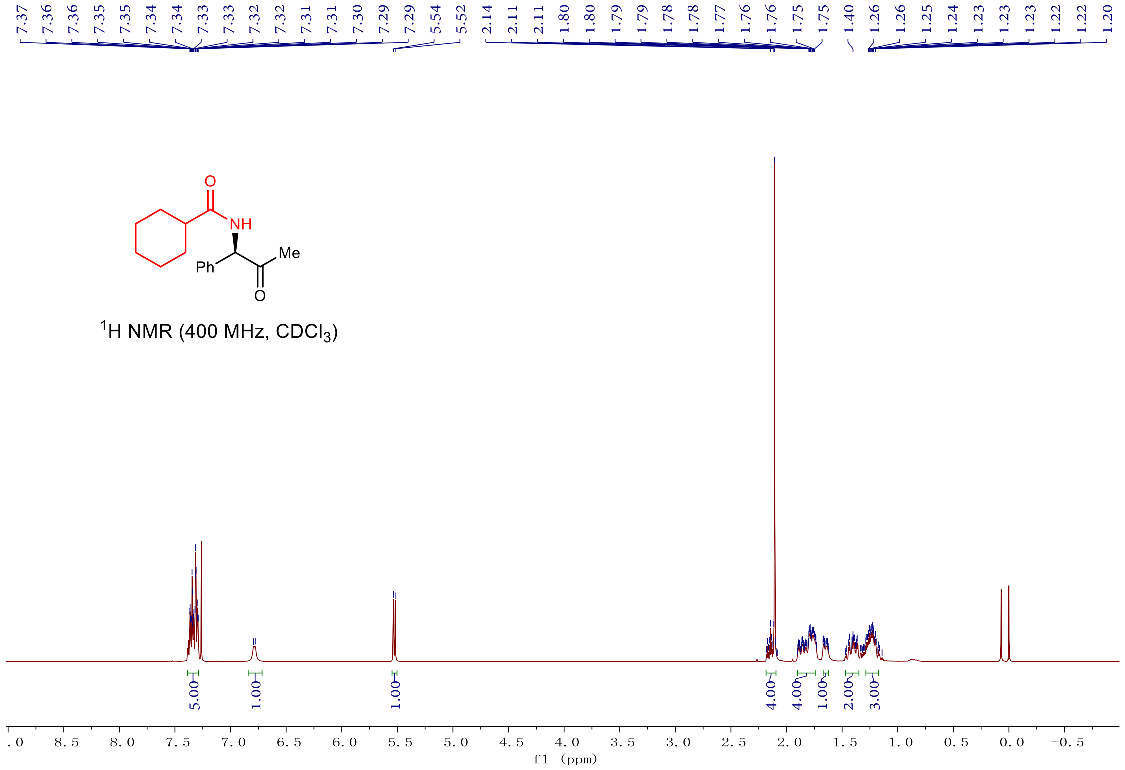

Supplementary Figure 54. ^{1}H NMR spectrum of compound 3l.

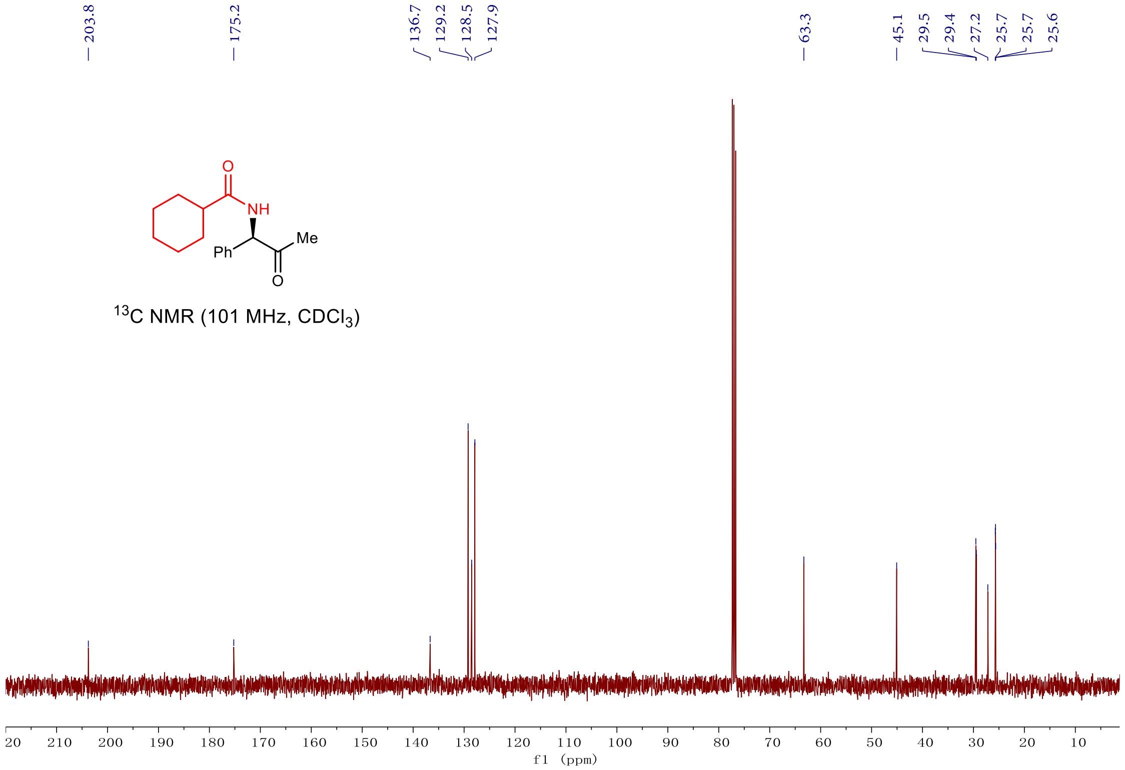

Supplementary Figure 55. ^{13}C NMR spectrum of compound 3l.

tert-butyl (*R*)-4-((2-oxo-1-phenylpropyl)carbamoyl)piperidine-1-carboxylate (3m)

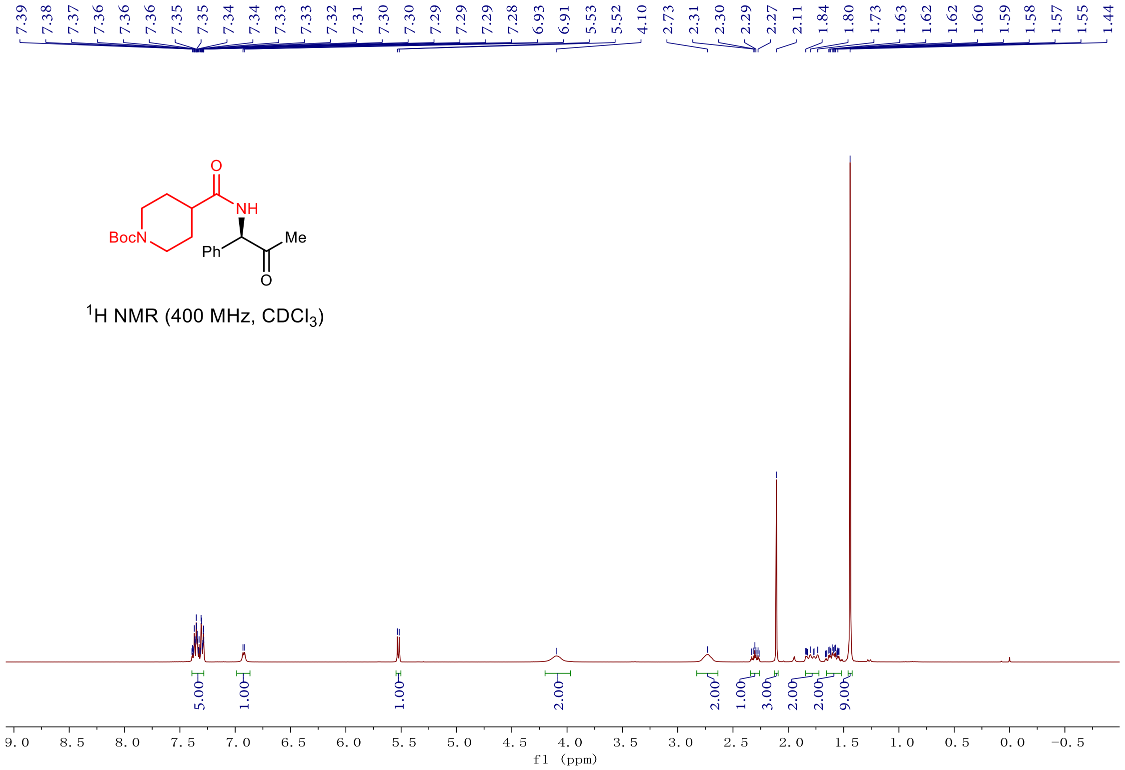

Supplementary Figure 56. **^{1}H NMR spectrum of compound 3m.**

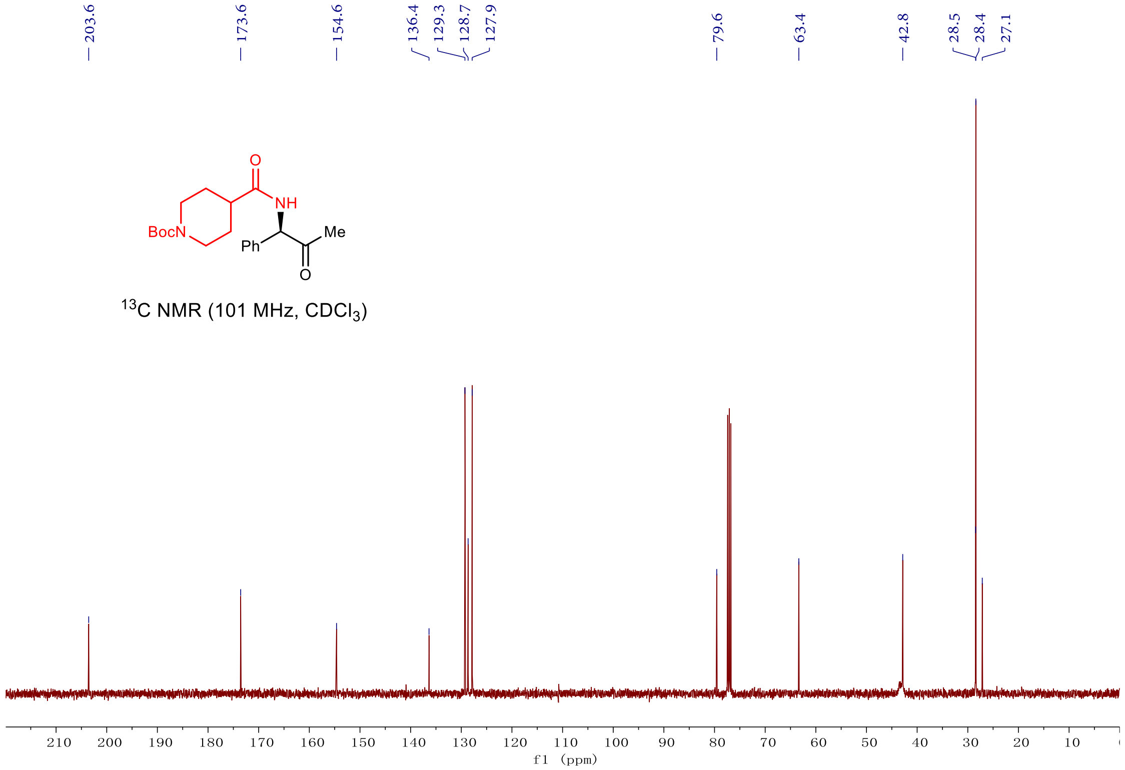

Supplementary Figure 57. **^{13}C NMR spectrum of compound 3m.**

(*R*)-N-(2-oxo-1-phenylpropyl)tetrahydro-2H-pyran-4-carboxamide (3n)

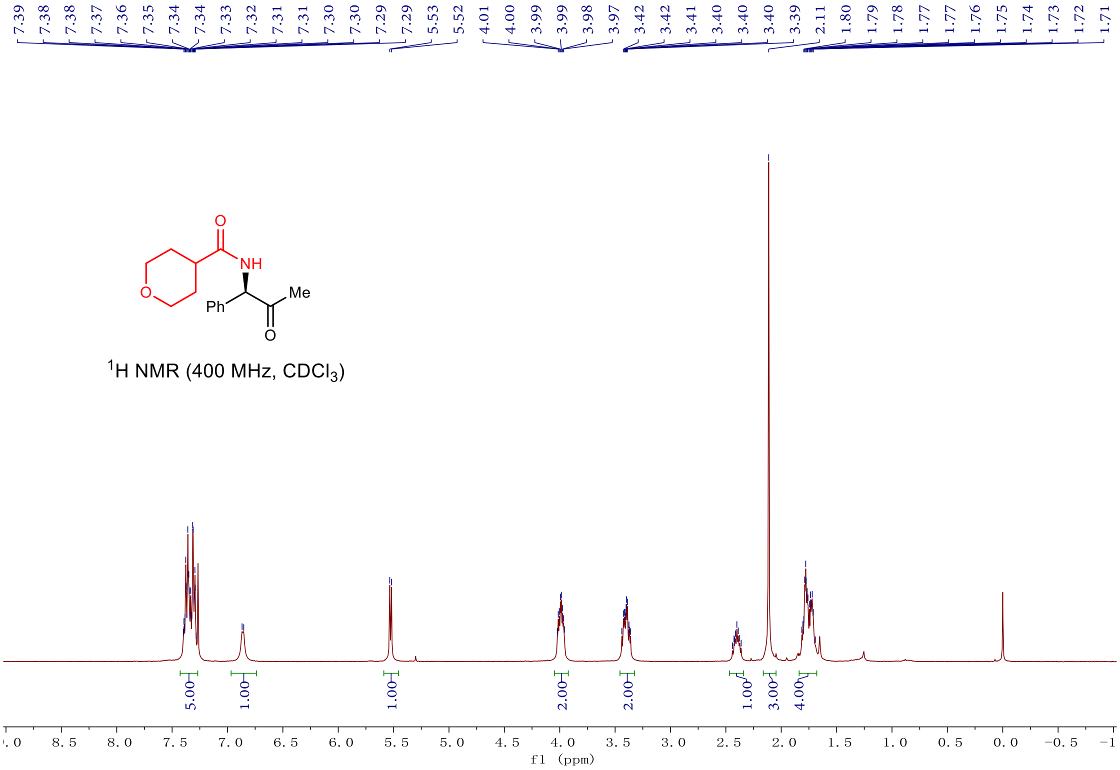

Supplementary Figure 58. ^{1}H NMR spectrum of compound 3n.

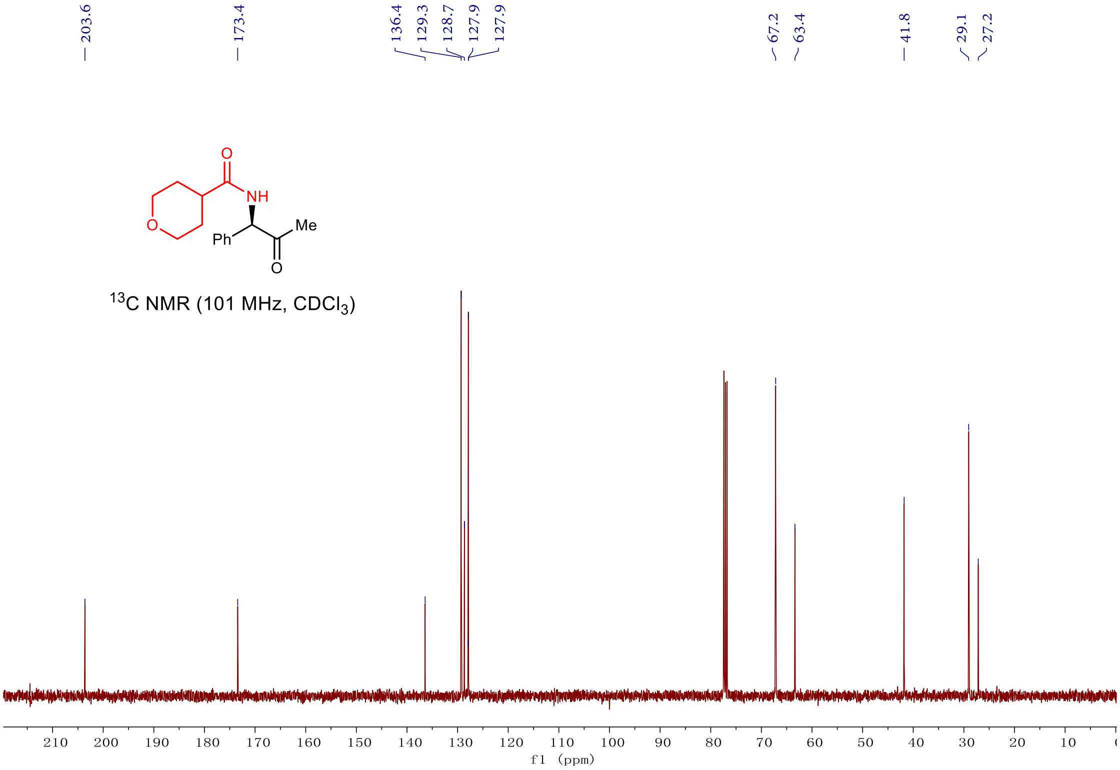

Supplementary Figure 59. ^{13}C NMR spectrum of compound 3n.

(*R*)-N-(2-oxo-1-phenylpropyl)cycloheptanecarboxamide (3o)

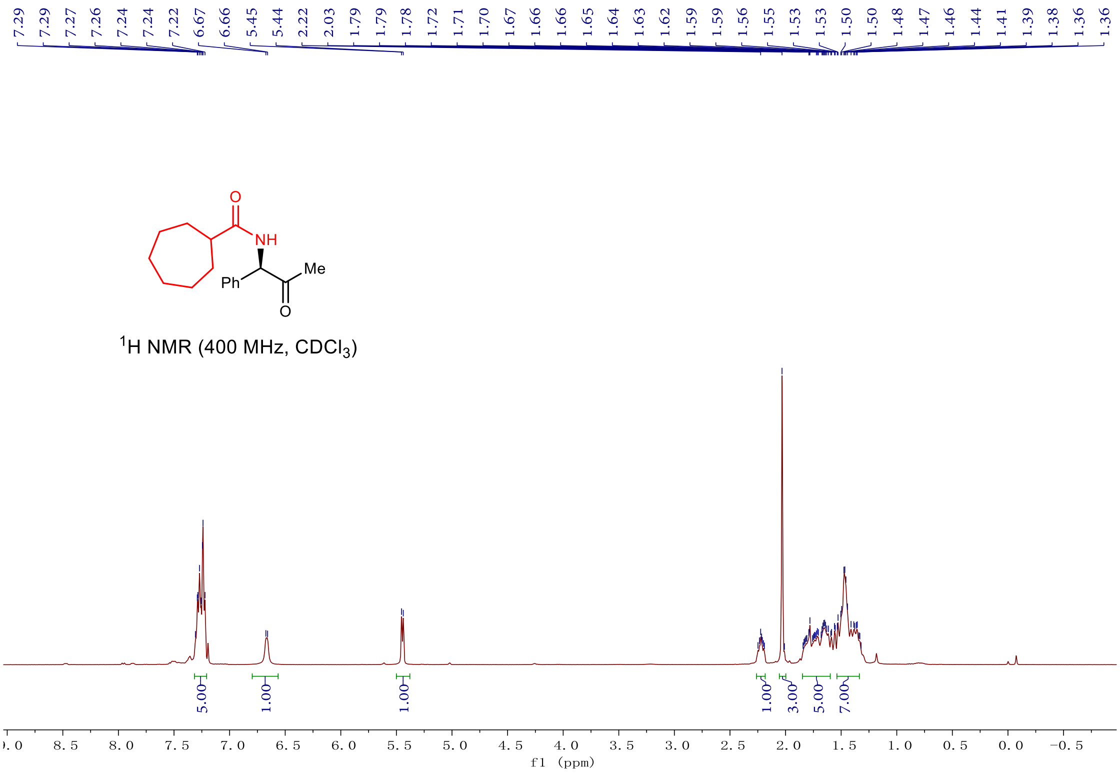

Supplementary Figure 60. ^{1}H NMR spectrum of compound 3o.

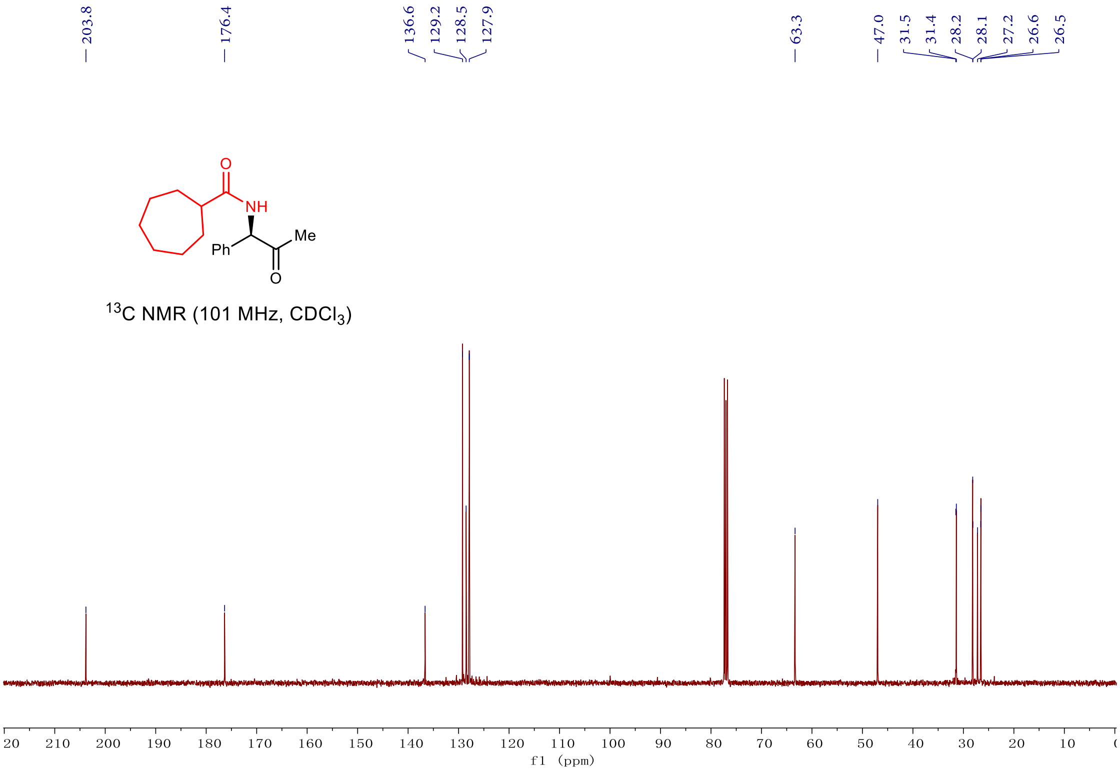

Supplementary Figure 61. ^{13}C NMR spectrum of compound 3o.

(*R*)-N-(2-oxo-1-phenylpropyl)benzamide (3p)

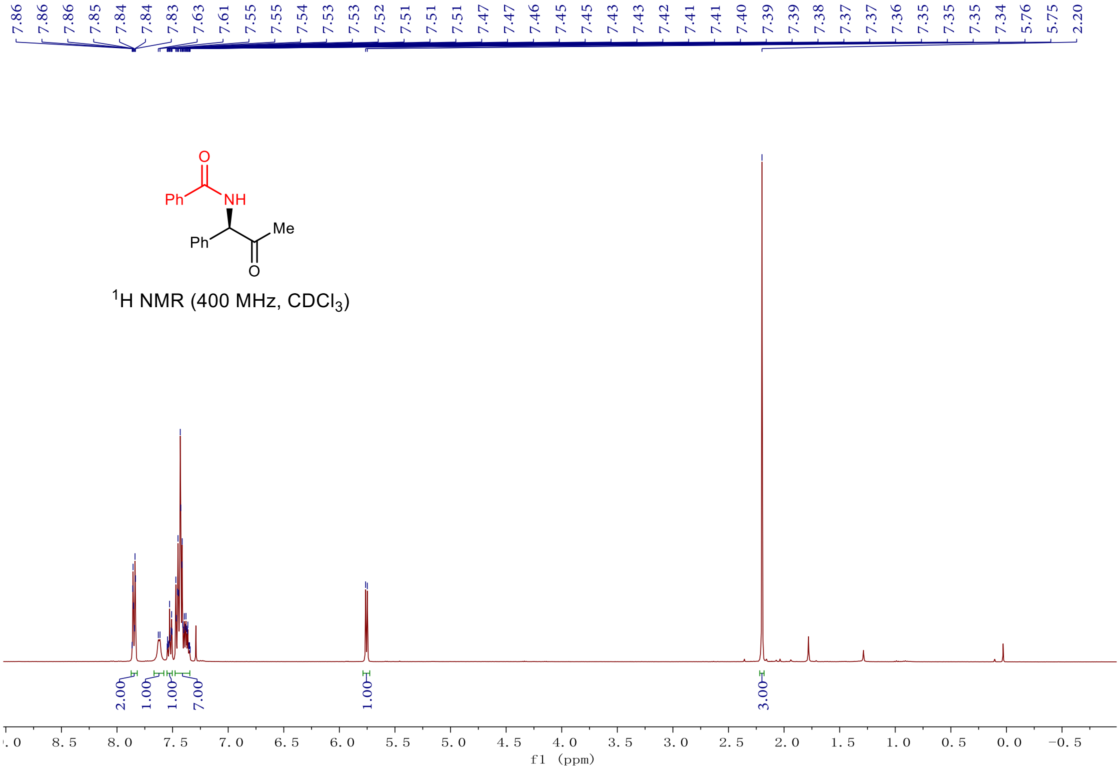

Supplementary Figure 62. ^{1}H NMR spectrum of compound 3p.

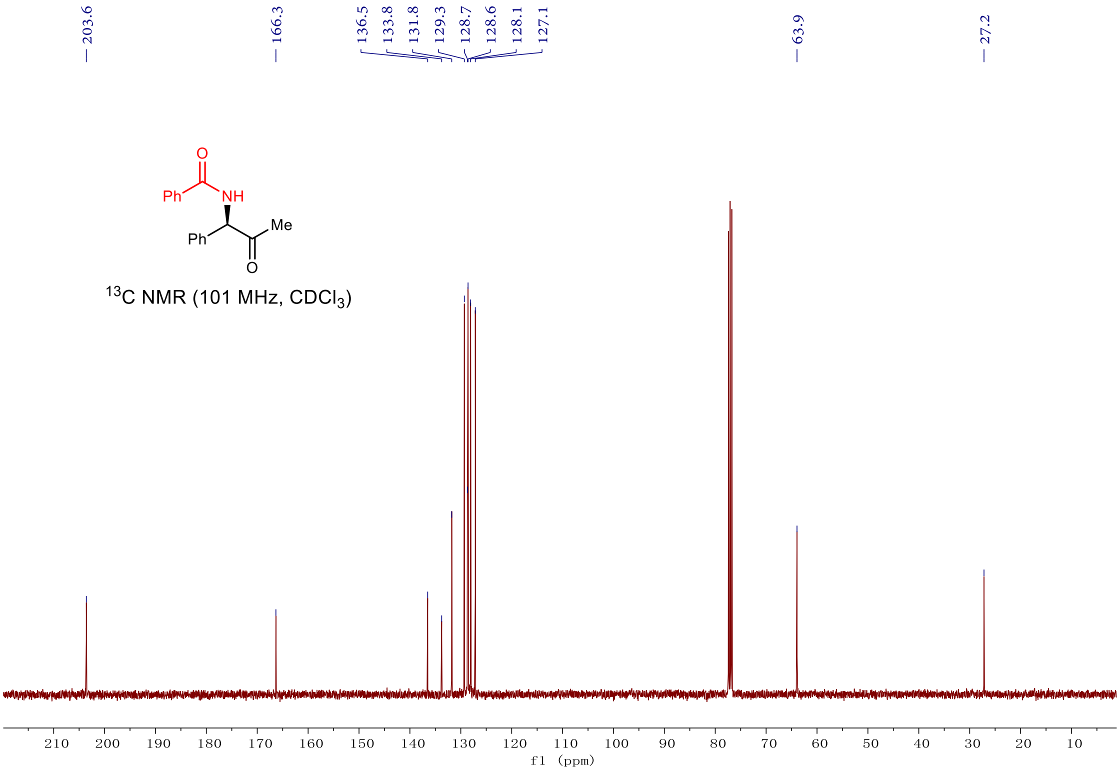

Supplementary Figure 63. ^{13}C NMR spectrum of compound 3p.

(*R*)-4-methoxy-N-(2-oxo-1-phenylpropyl)benzamide (3q)

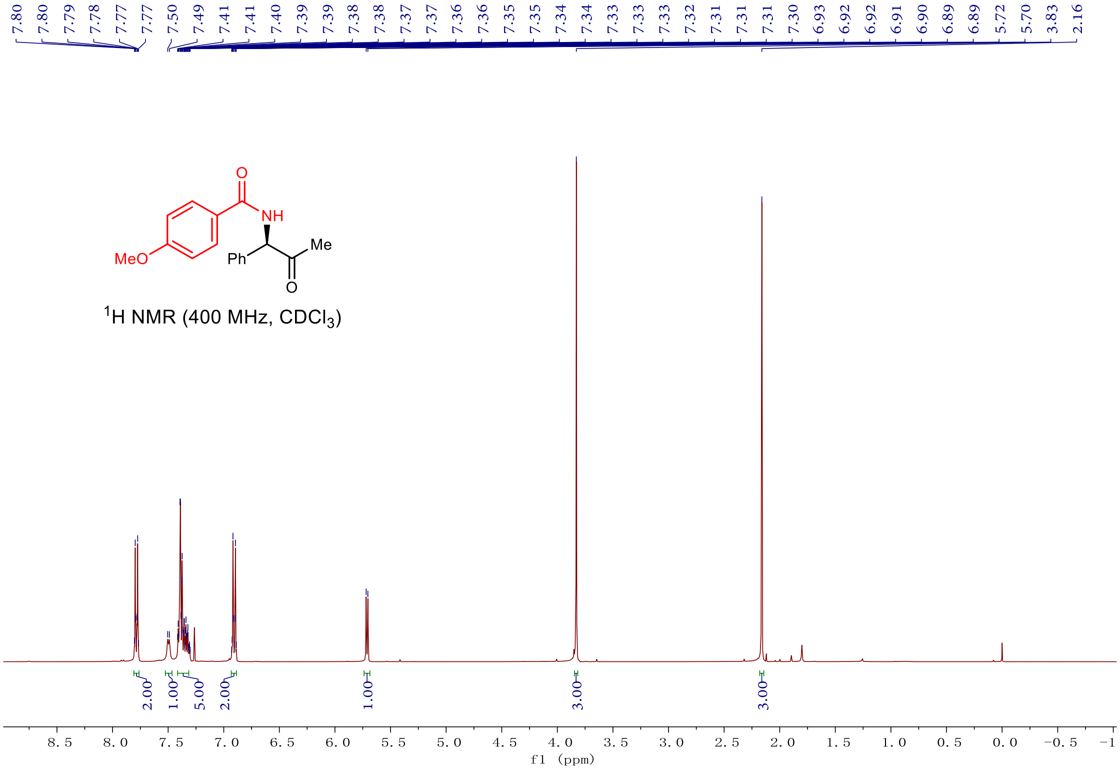

Supplementary Figure 64. ^{1}H NMR spectrum of compound 3q.

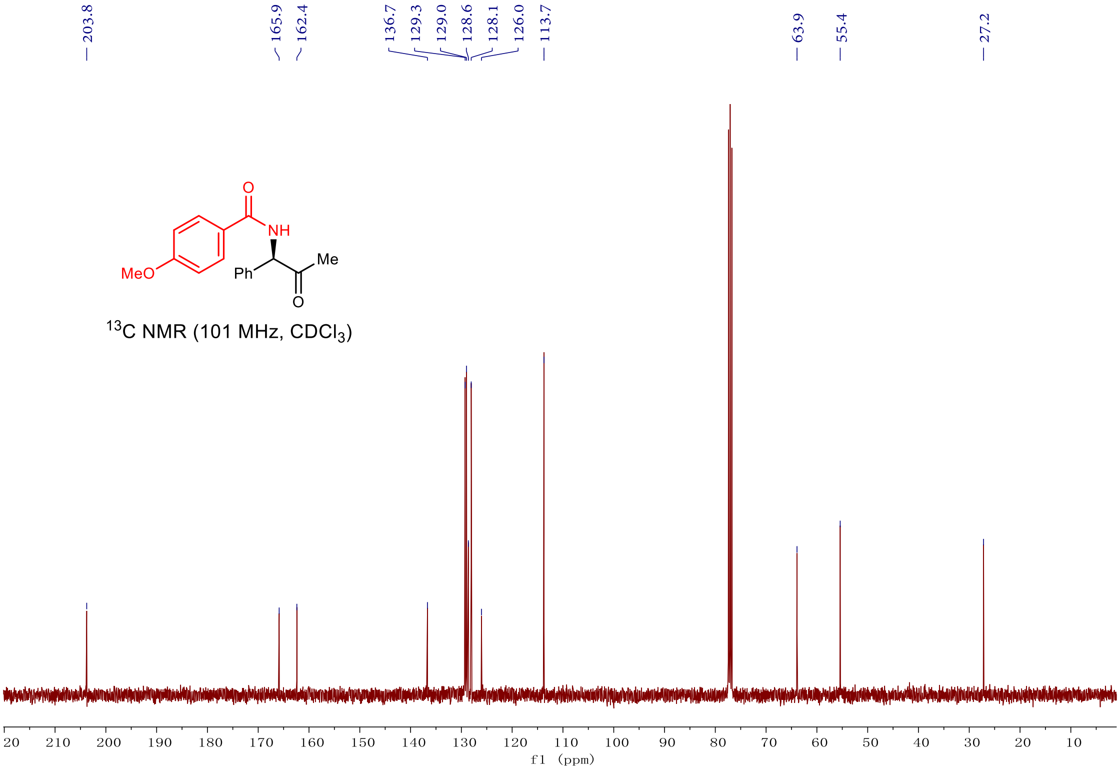

Supplementary Figure 65. ^{13}C NMR spectrum of compound 3q.

(*R*)-N-(2-oxo-1-phenylpropyl)-4-(trifluoromethyl)benzamide (3r)

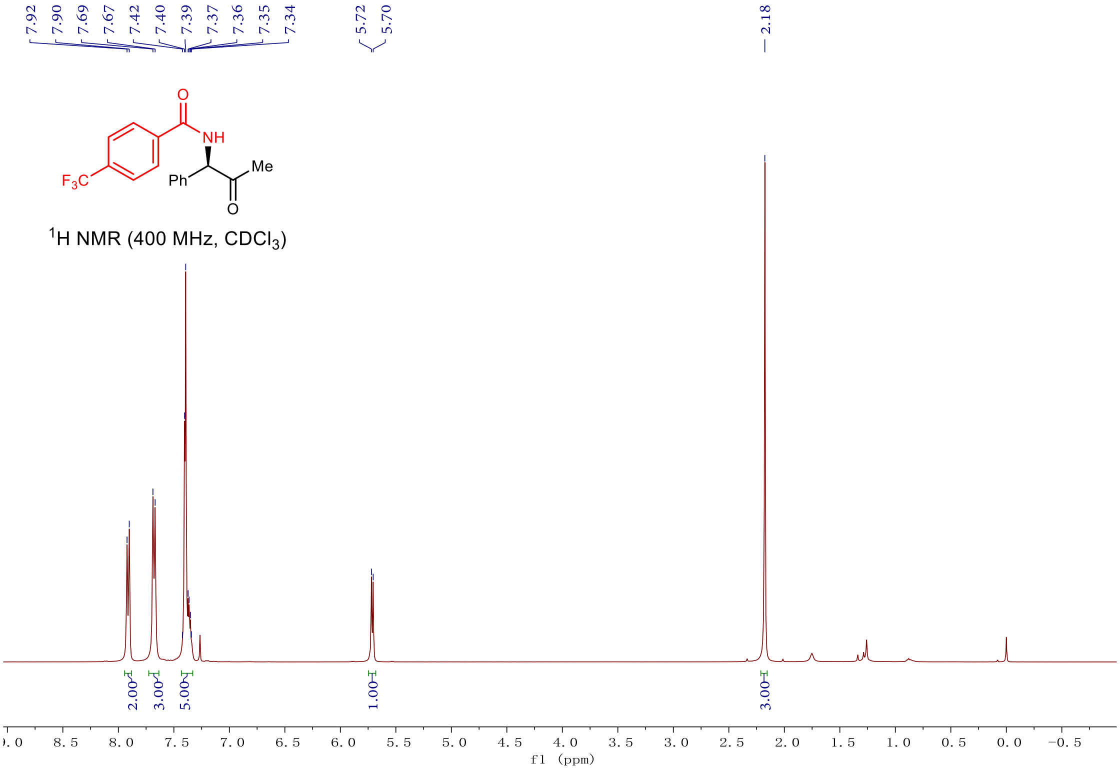

Supplementary Figure 66. ^{1}H NMR spectrum of compound 3r.

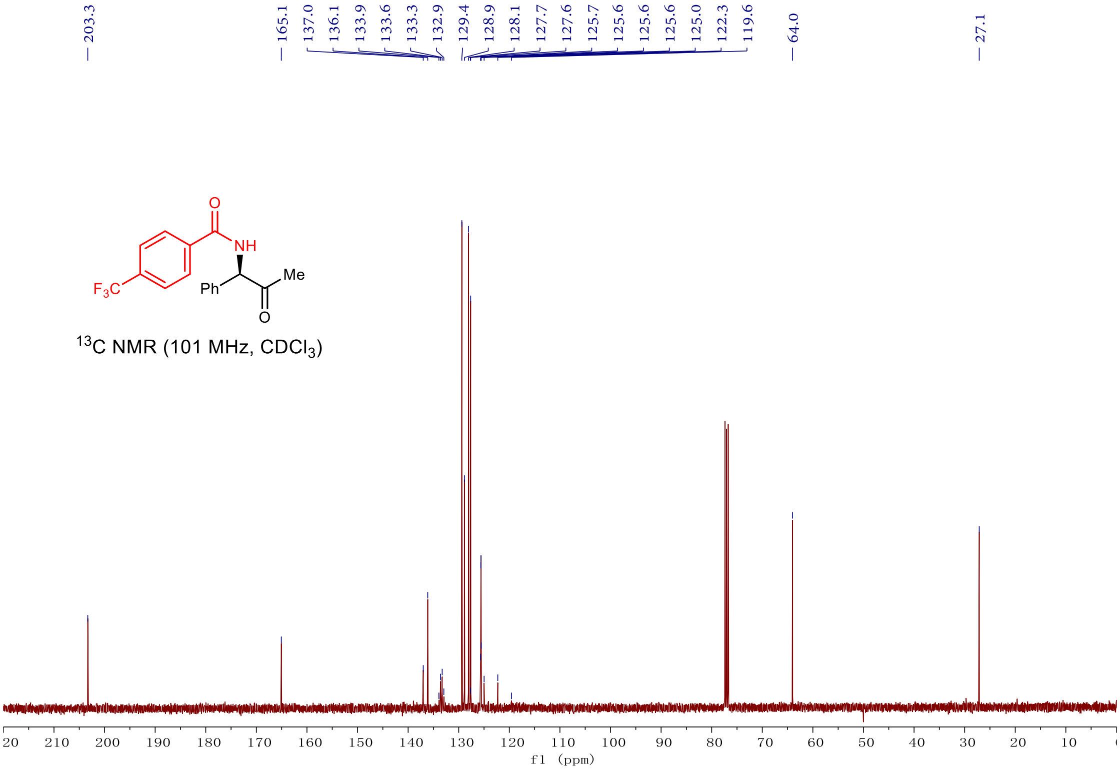

Supplementary Figure 67. ^{13}C NMR spectrum of compound 3r.

(*R*)-4-fluoro-N-(2-oxo-1-phenylpropyl)benzamide (3s)

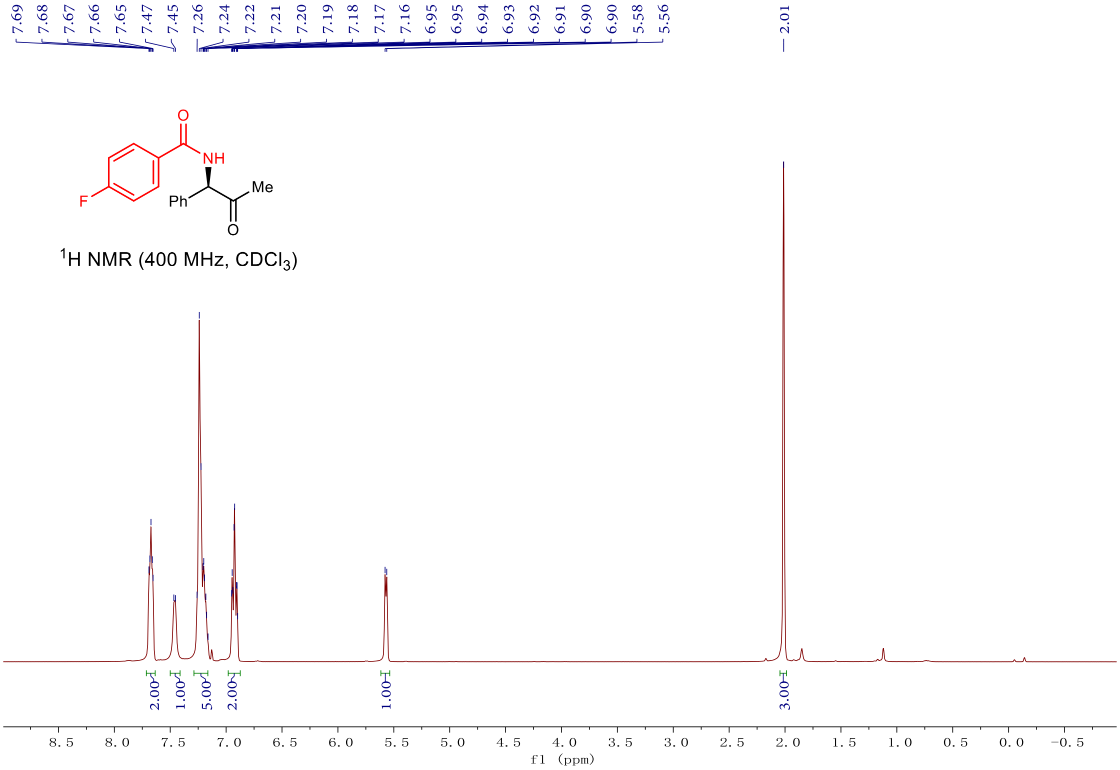

Supplementary Figure 68. ^{1}H NMR spectrum of compound 3s.

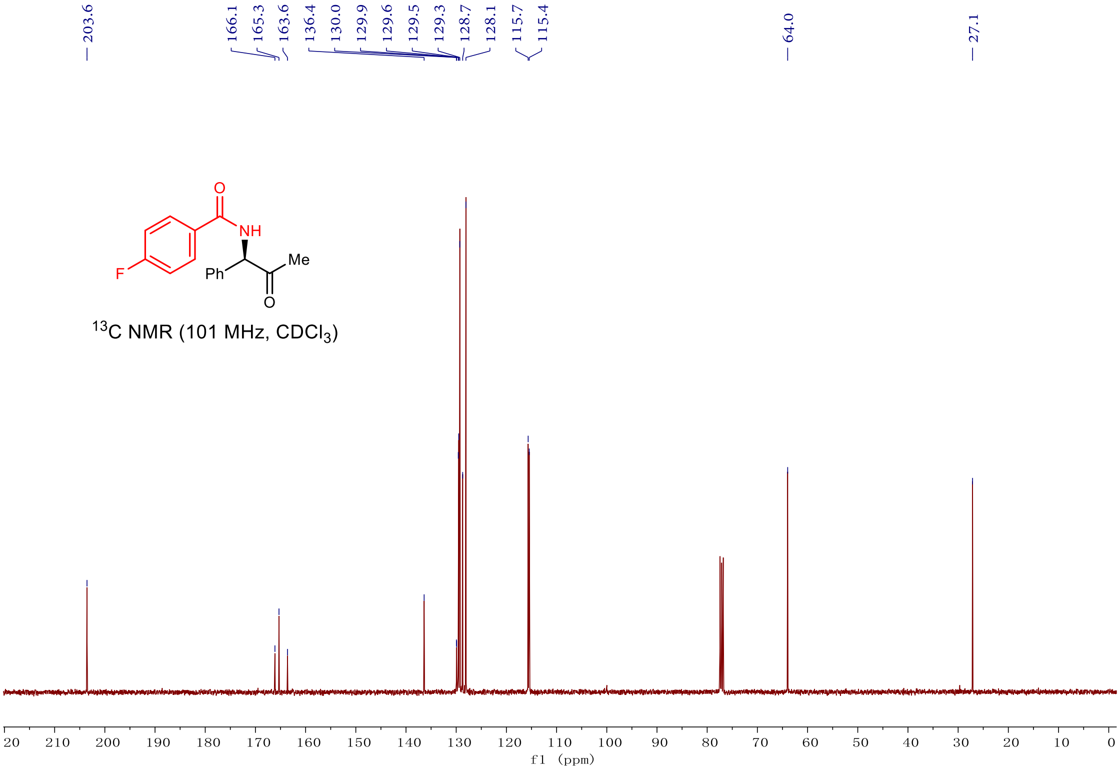

Supplementary Figure 69. ^{13}C NMR spectrum of compound 3s.

(*R*)-4-chloro-N-(2-oxo-1-phenylpropyl)benzamide (3t)

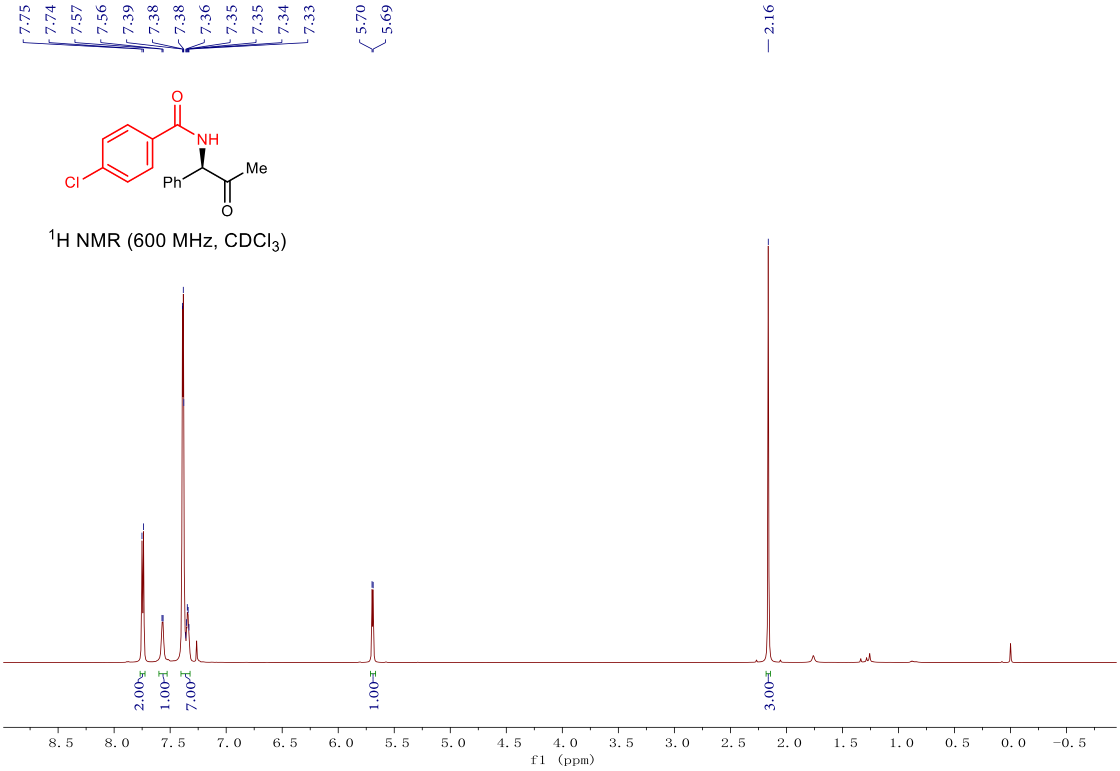

Supplementary Figure 70. ^{1}H NMR spectrum of compound 3t.

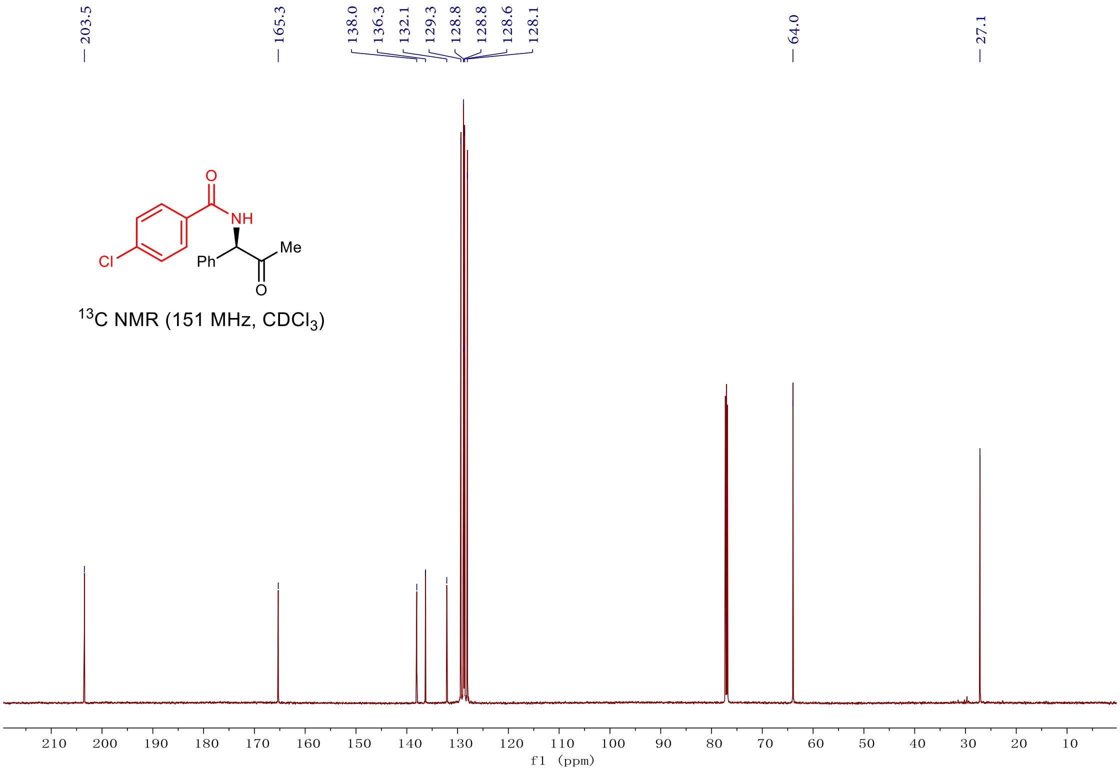

Supplementary Figure 71. ^{13}C NMR spectrum of compound 3t.

(*R*)-4-bromo-N-(2-oxo-1-phenylpropyl)benzamide (3u)

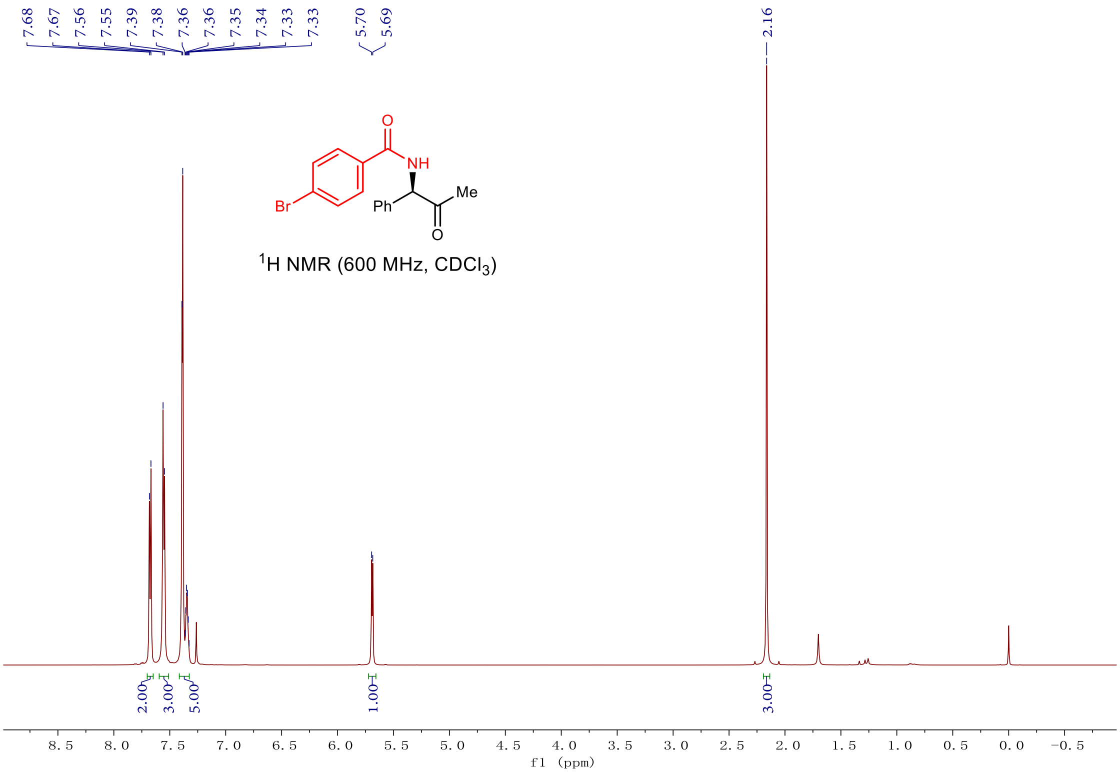

Supplementary Figure 72. ^{1}H NMR spectrum of compound 3u.

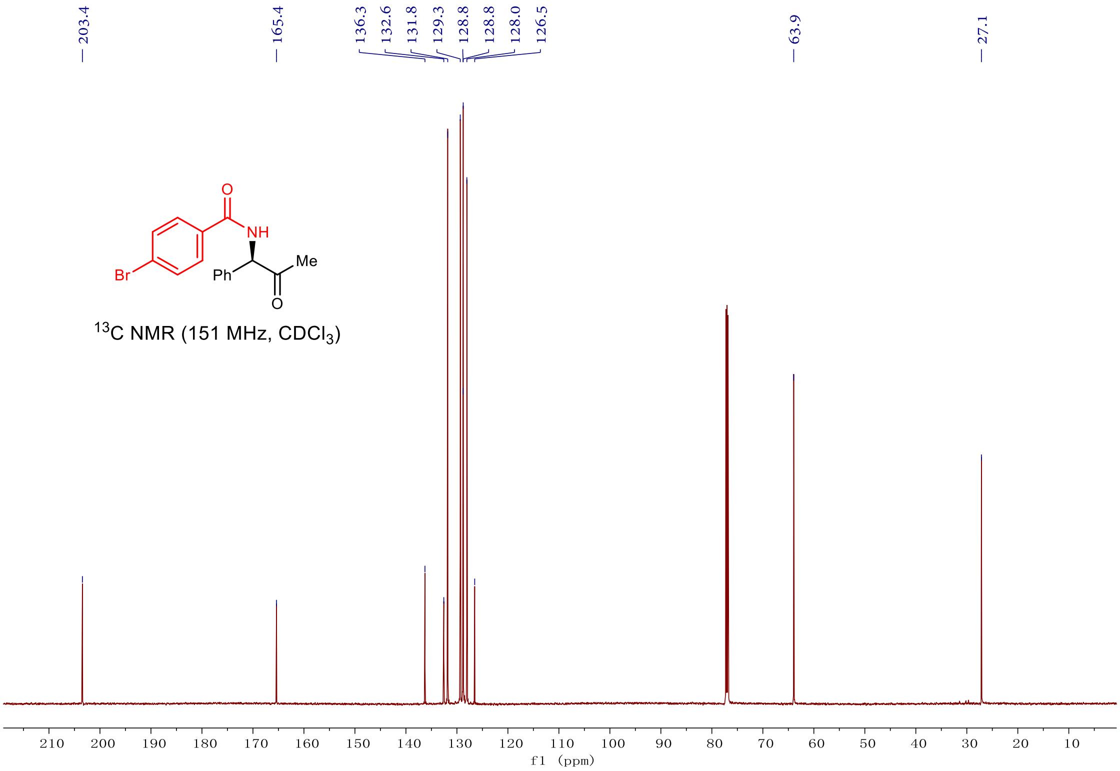

Supplementary Figure 73. ^{13}C NMR spectrum of compound 3u.

(*R*)-4-iodo-N-(2-oxo-1-phenylpropyl)benzamide (3v)

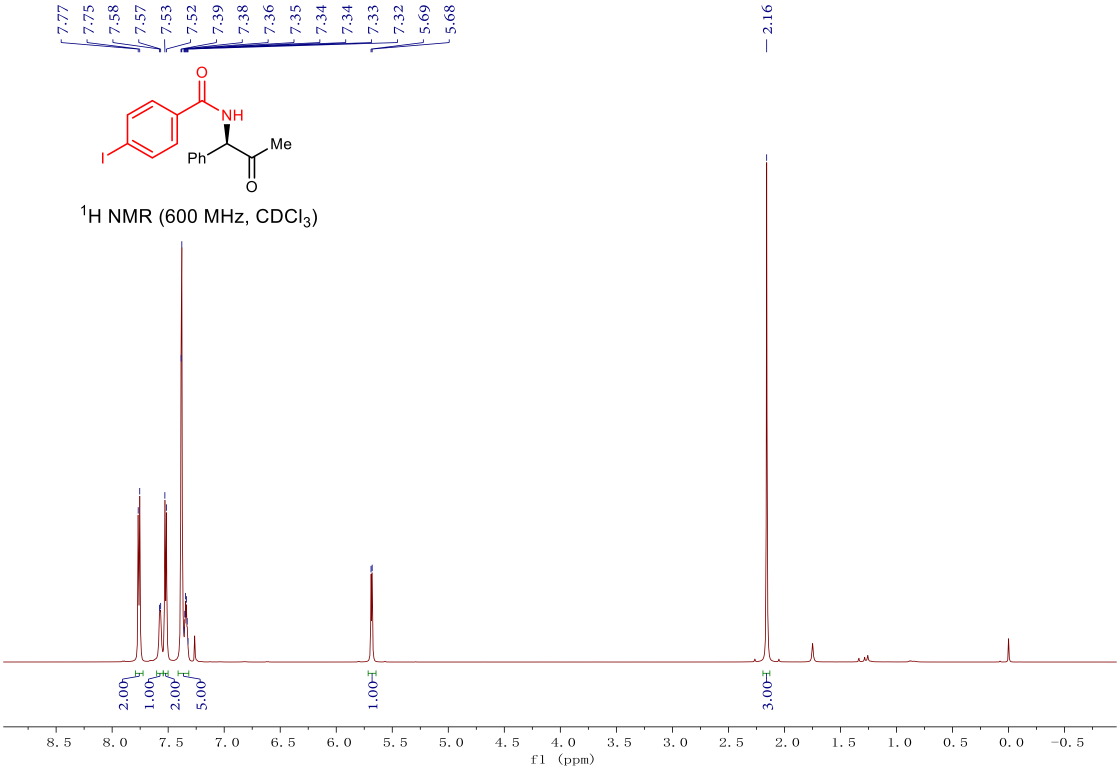

Supplementary Figure 74. ^{1}H NMR spectrum of compound 3v.

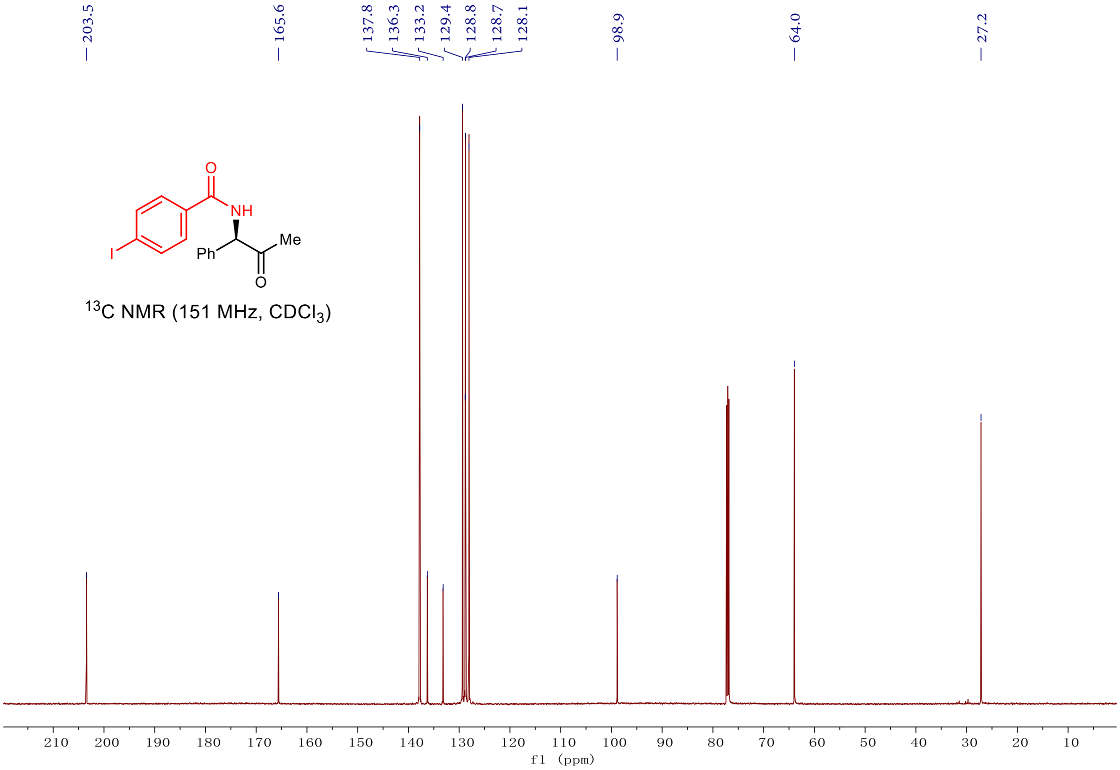

Supplementary Figure 75. ^{13}C NMR spectrum of compound 3v.

(*R*)-N-(2-oxo-1-phenylpropyl)-2-naphthamide (3w)

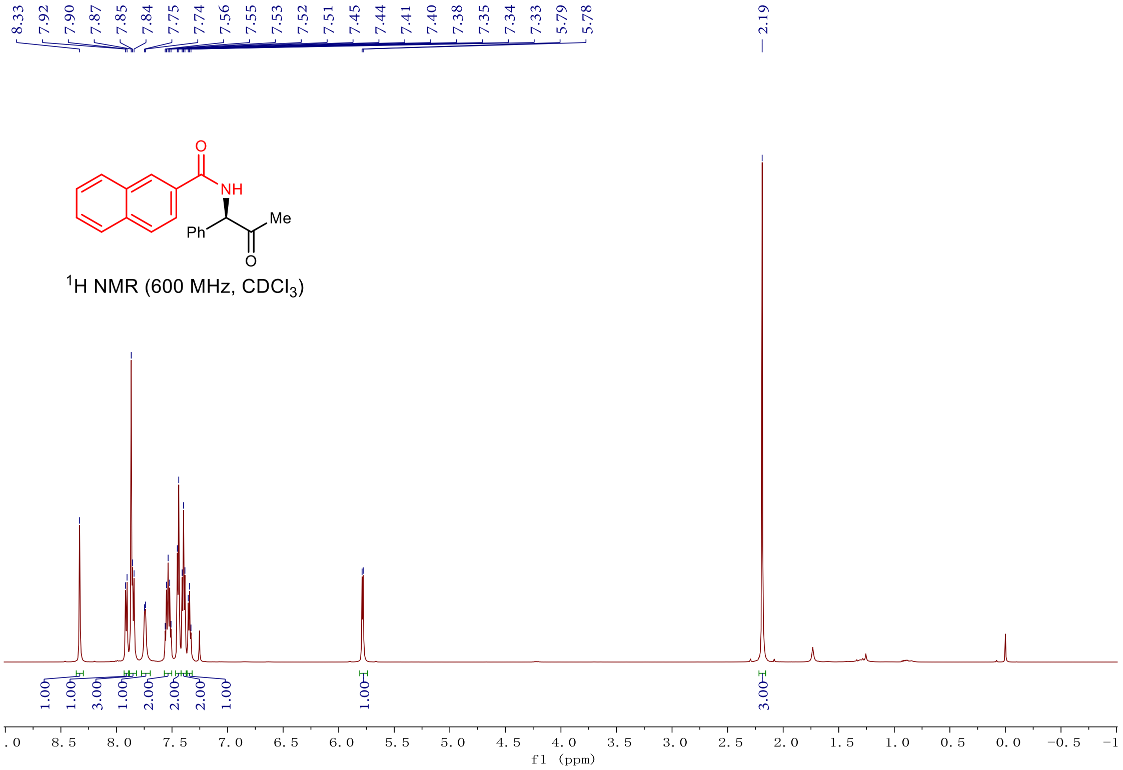

Supplementary Figure 76. ^{1}H NMR spectrum of compound 3w.

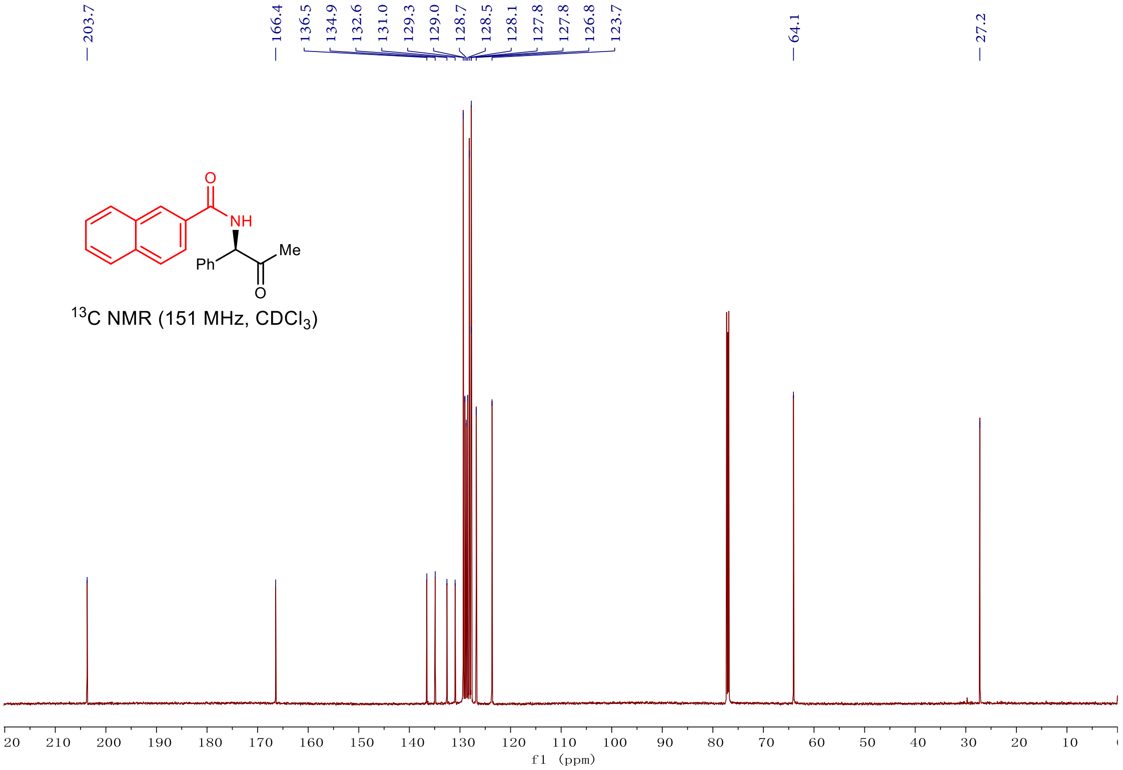

Supplementary Figure 77. ^{13}C NMR spectrum of compound 3w.

(*R*)-N-(2-oxo-1-phenylpropyl)furan-2-carboxamide (3x)

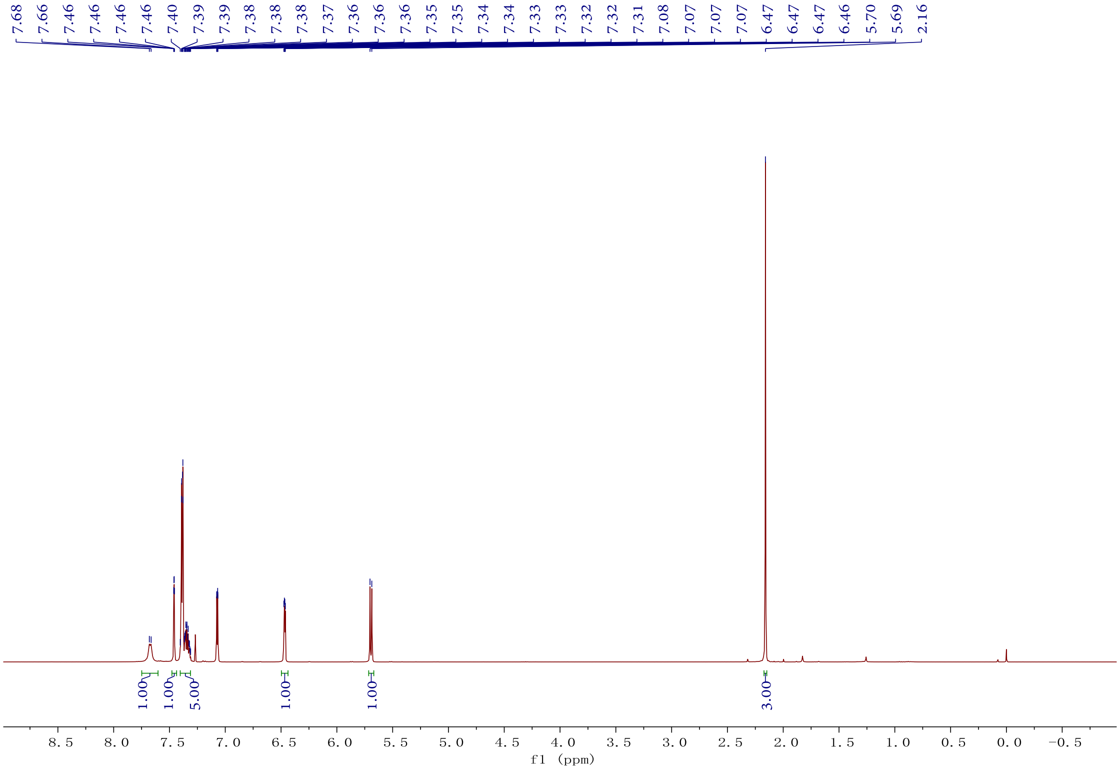

Supplementary Figure 78. ^{1}H NMR spectrum of compound 3x.

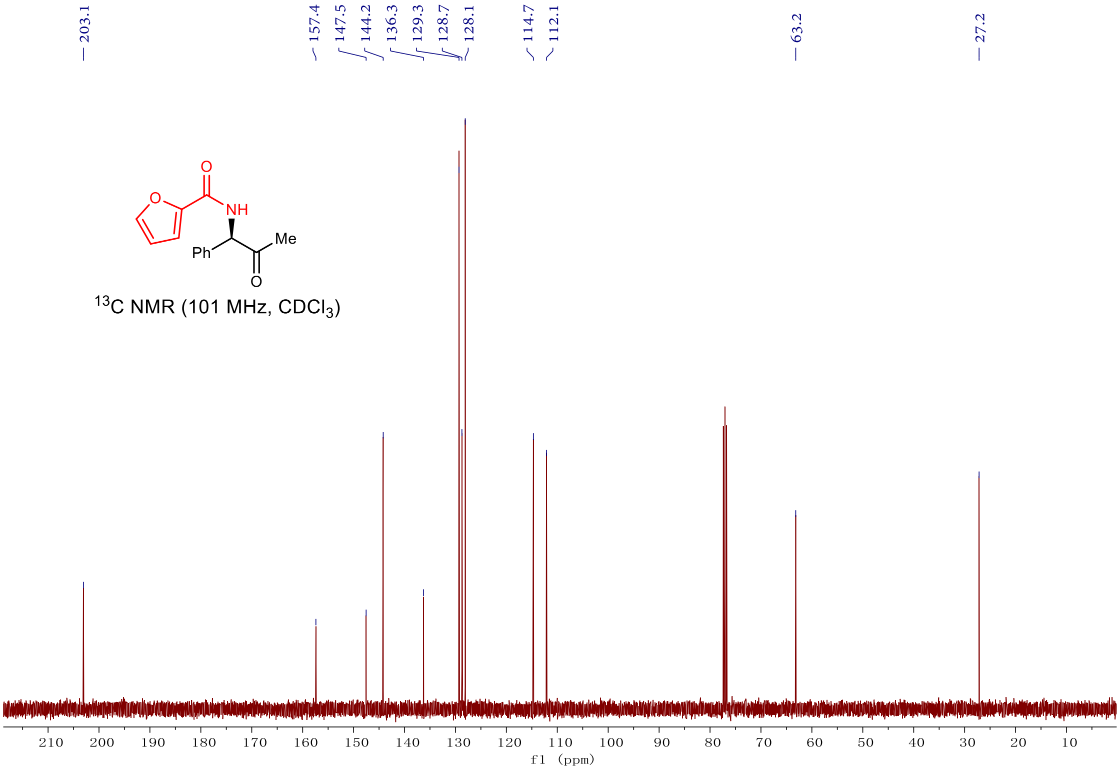

Supplementary Figure 79. ^{13}C NMR spectrum of compound 3x.

(*R*)-N-(2-oxo-1-phenylpropyl)thiophene-2-carboxamide (3y)

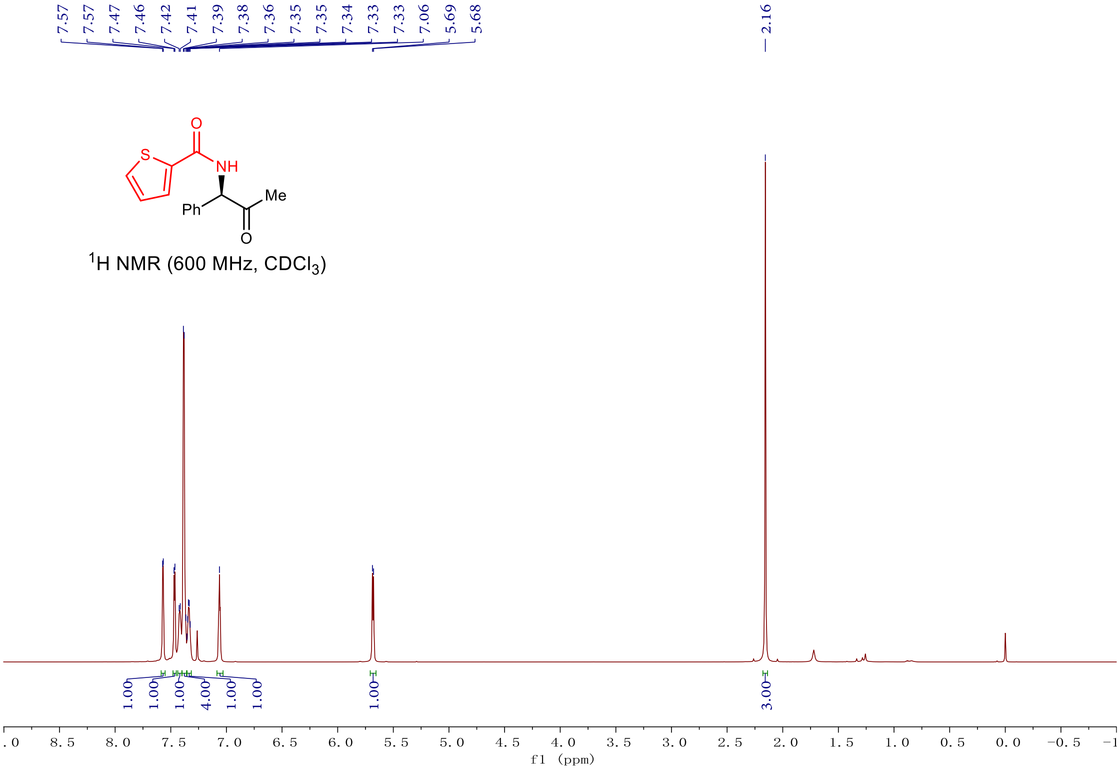

Supplementary Figure 80. ^{1}H NMR spectrum of compound 3y.

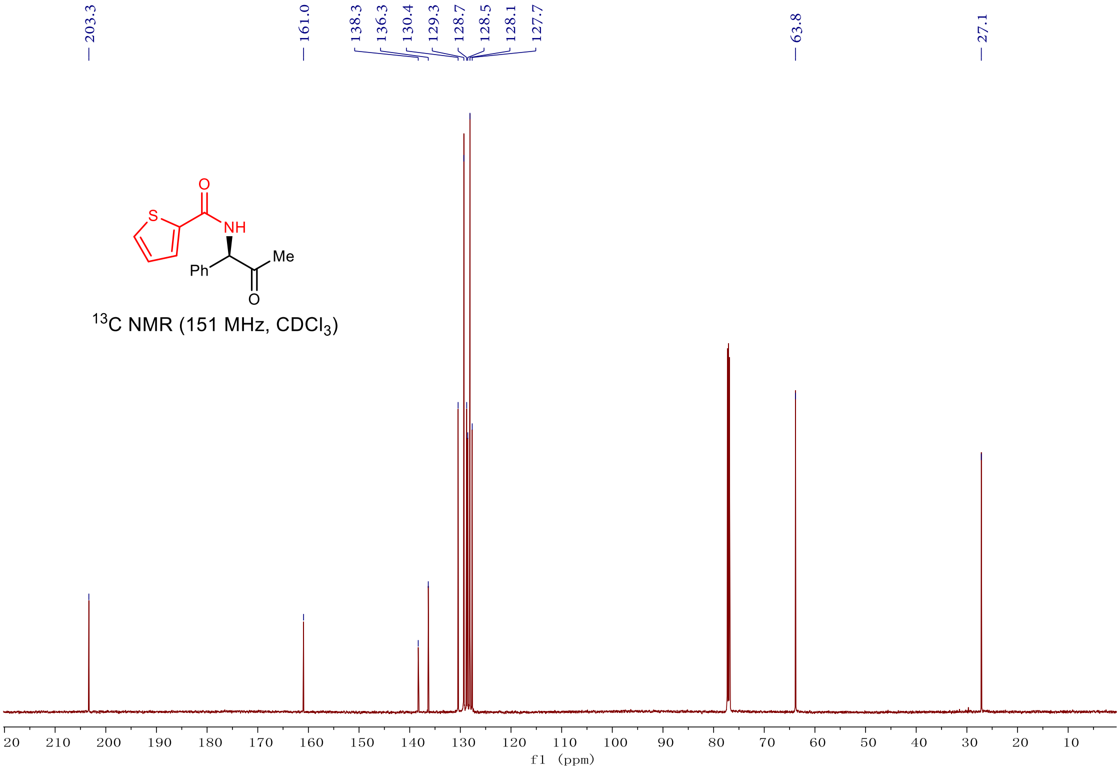

Supplementary Figure 81. ^{13}C NMR spectrum of compound 3y.

(*R*)-N-(2-oxo-1-phenylpropyl)picolinamide (3z)

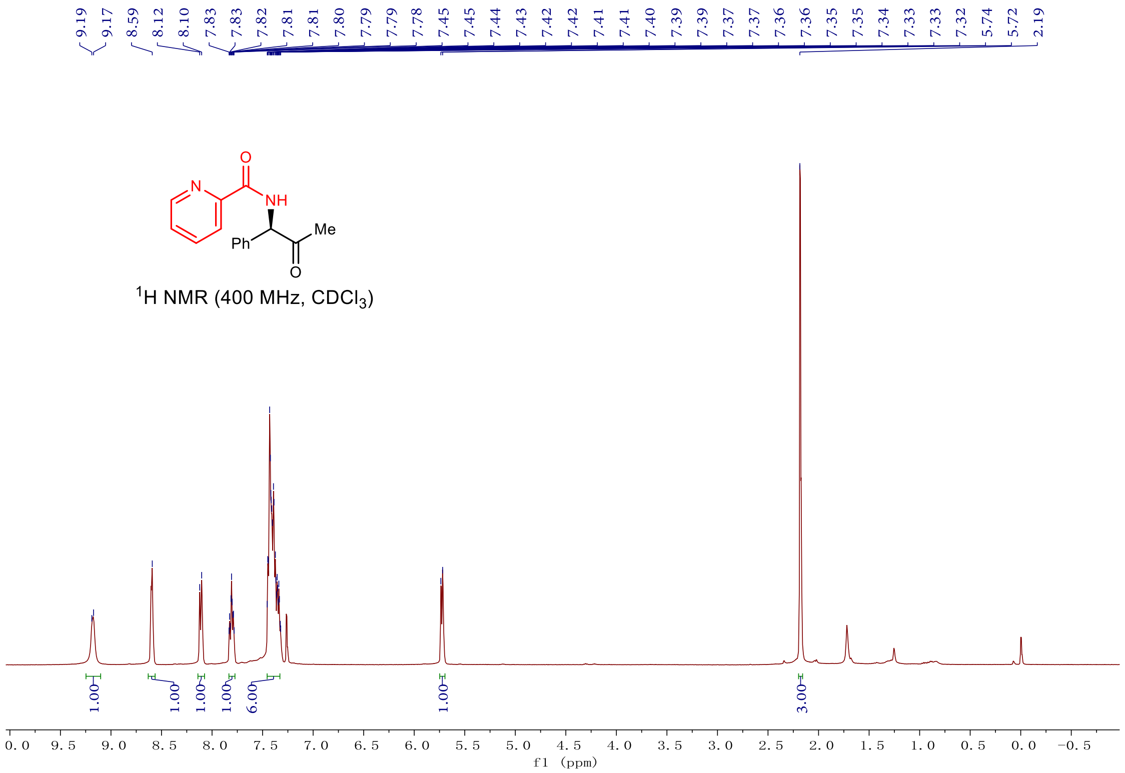

Supplementary Figure 82. ^{1}H NMR spectrum of compound 3z.

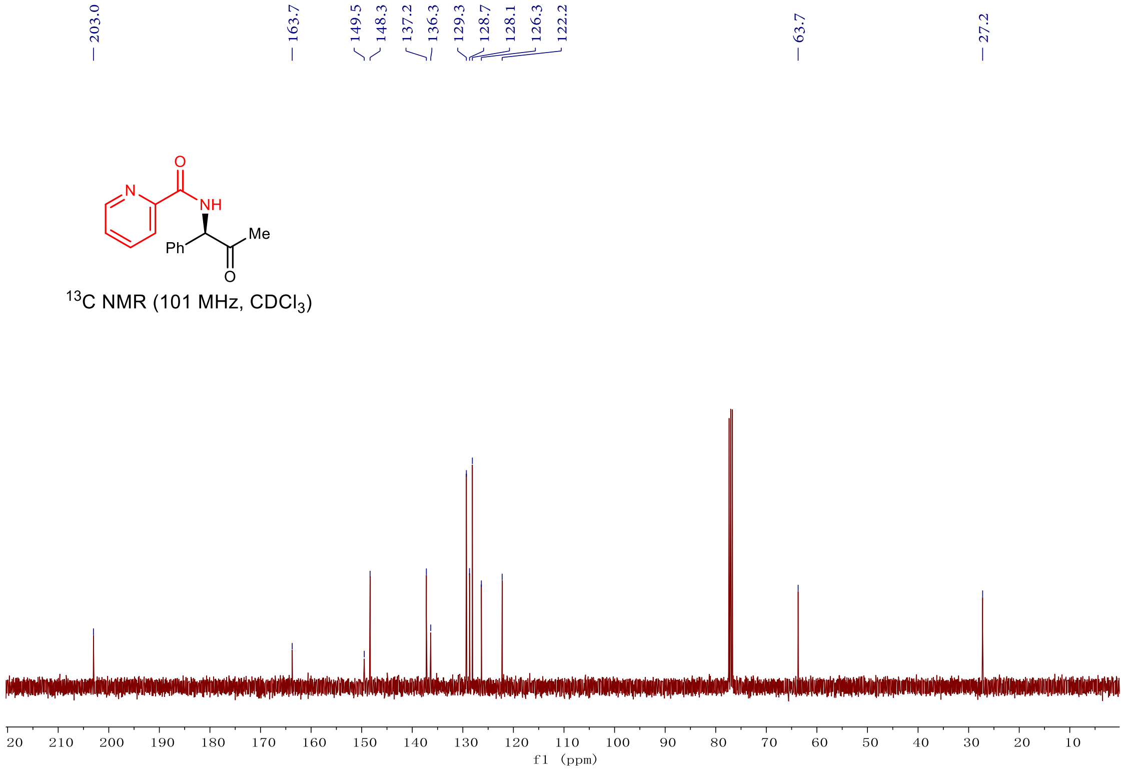

Supplementary Figure 83. ^{13}C NMR spectrum of compound 3z.

(*R*)-N-(2-oxo-1-phenylpropyl)-1H-indole-2-carboxamide (3aa)

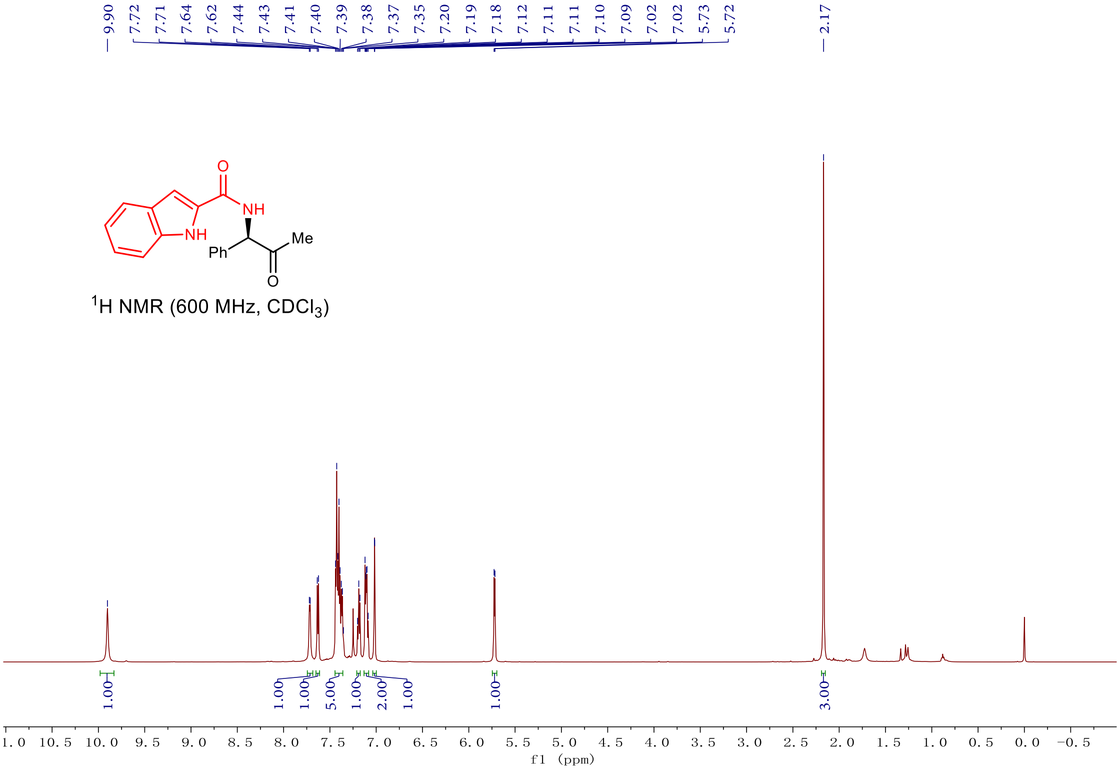

Supplementary Figure 84. ^{1}H NMR spectrum of compound 3aa.

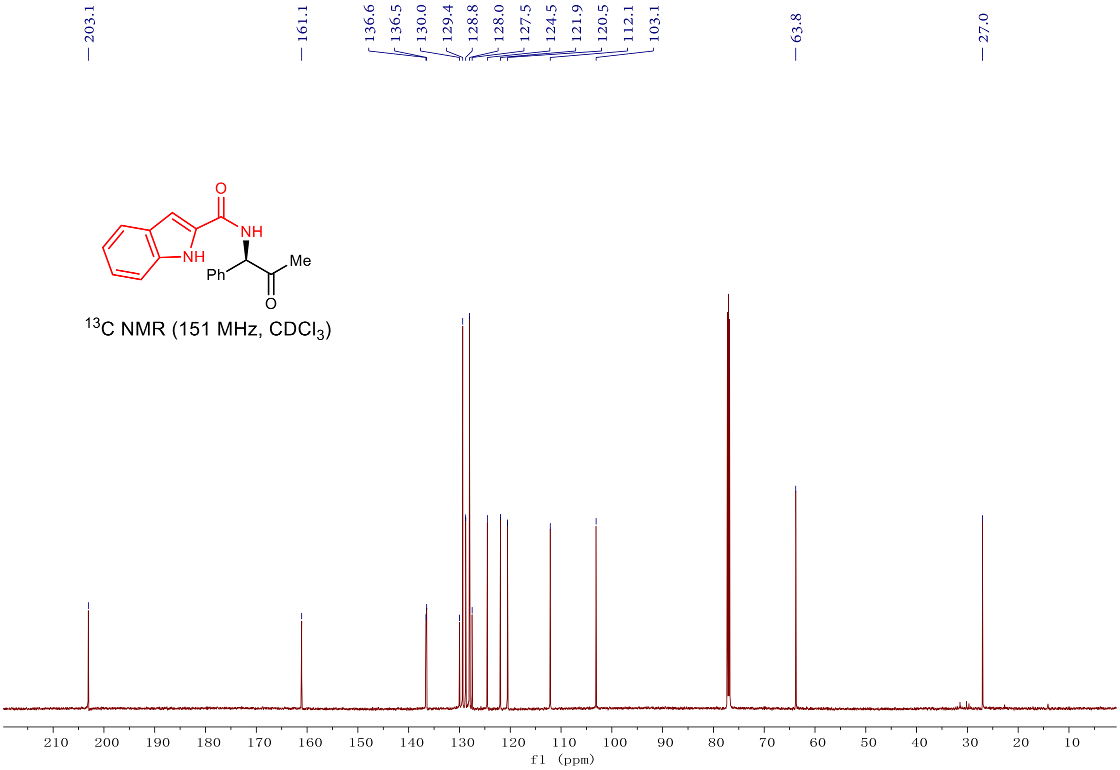

Supplementary Figure 85. ^{13}C NMR spectrum of compound 3aa.

(*R*)-N-(2-oxo-1-phenylpropyl)acrylamide (3ab)

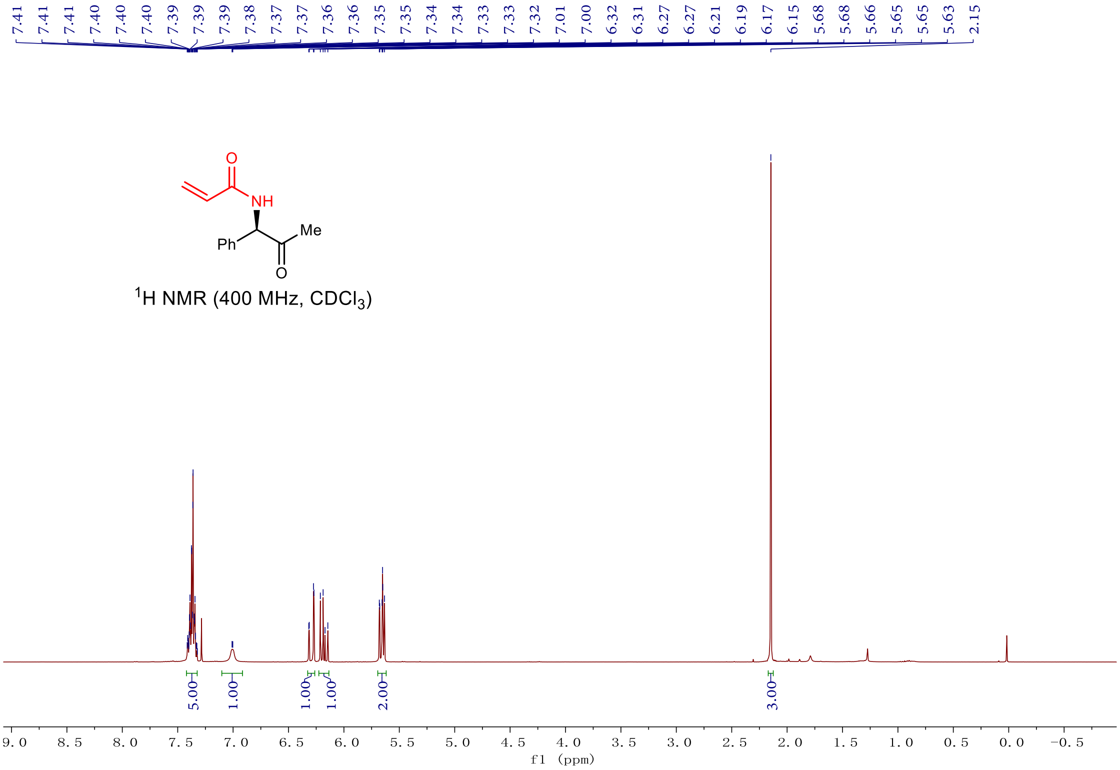

Supplementary Figure 86. ^{1}H NMR spectrum of compound 3ab.

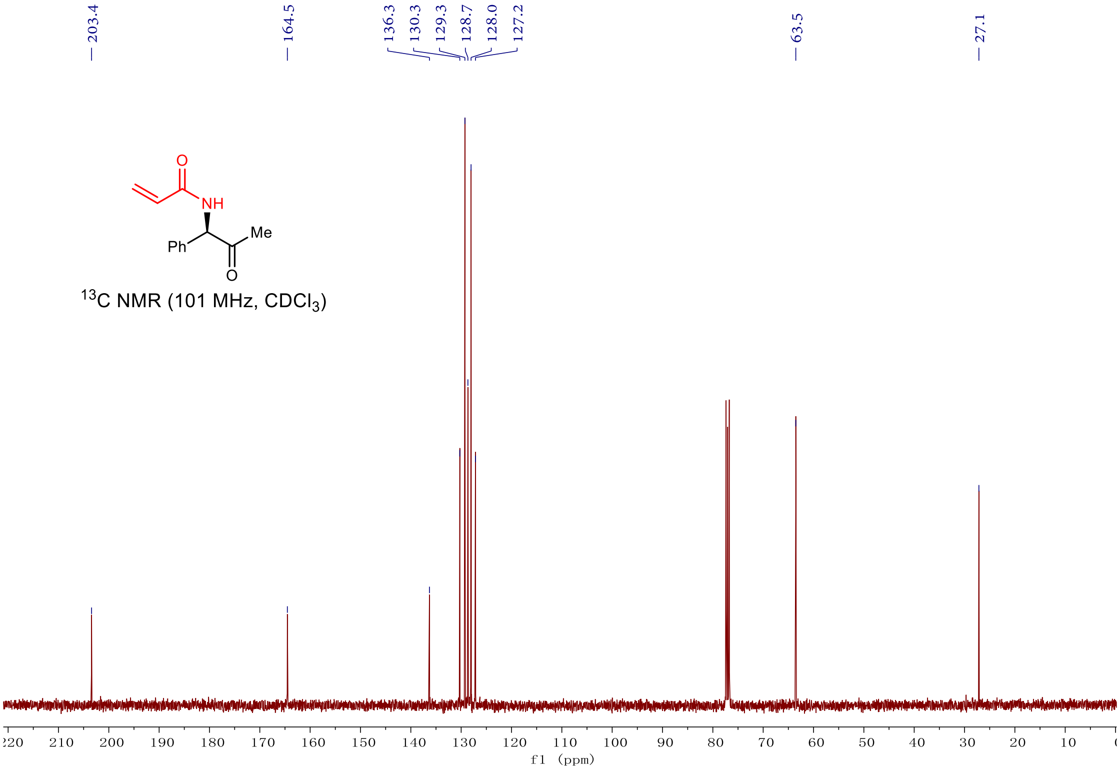

Supplementary Figure 87. ^{13}C NMR spectrum of compound 3ab.

(*R*)-N-(2-oxo-1-phenylpropyl)-3-(trimethylsilyl)propiolamide (3ac)

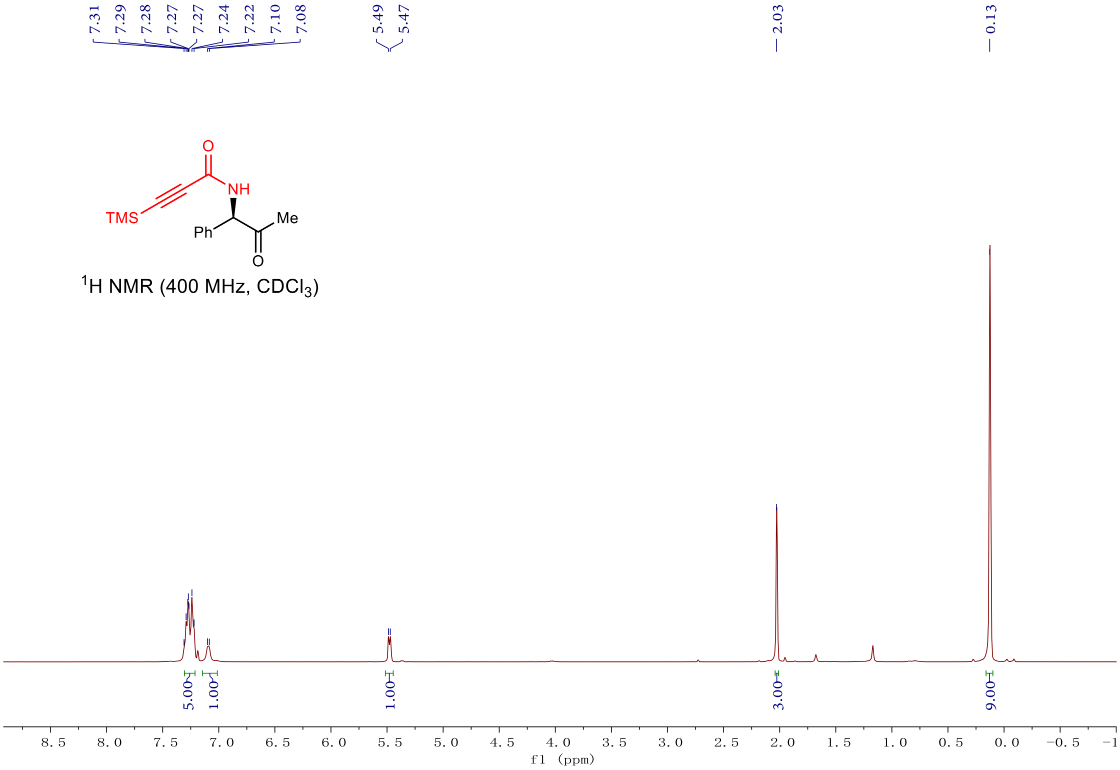

Supplementary Figure 88. ^{1}H NMR spectrum of compound 3ac.

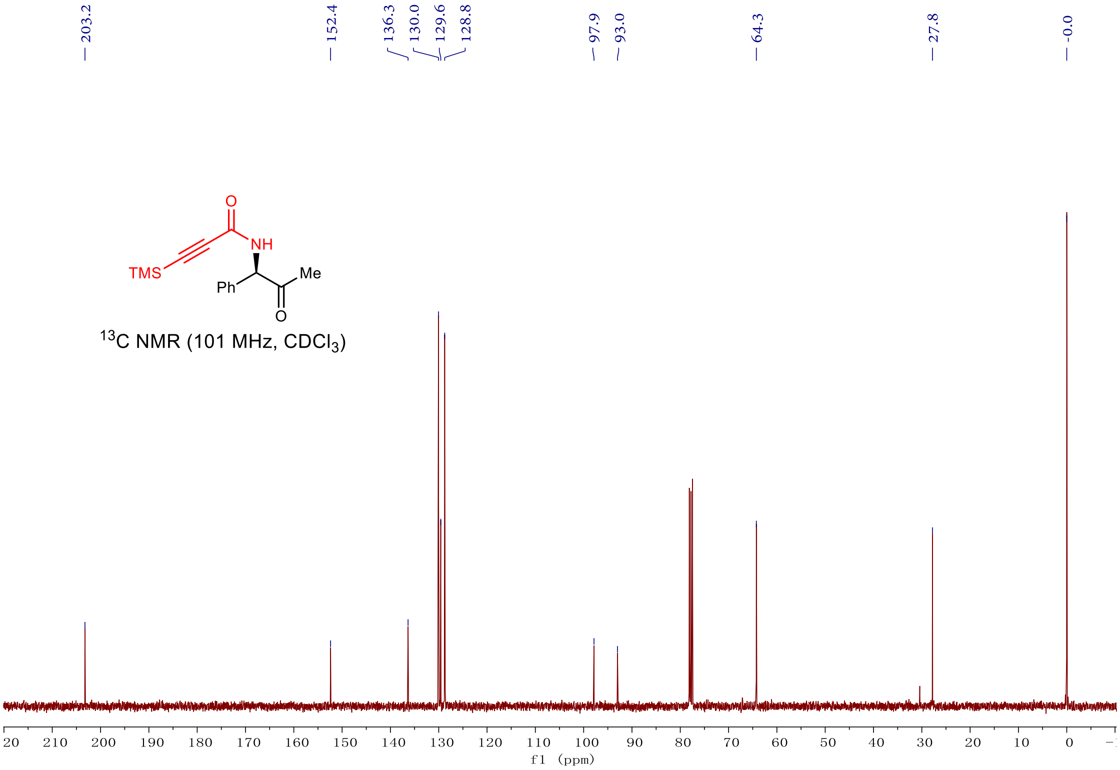

Supplementary Figure 89. ^{13}C NMR spectrum of compound 3ac.

(*R*)-N-(2-oxo-1-(*p*-tolyl)propyl)butyramide (4a)

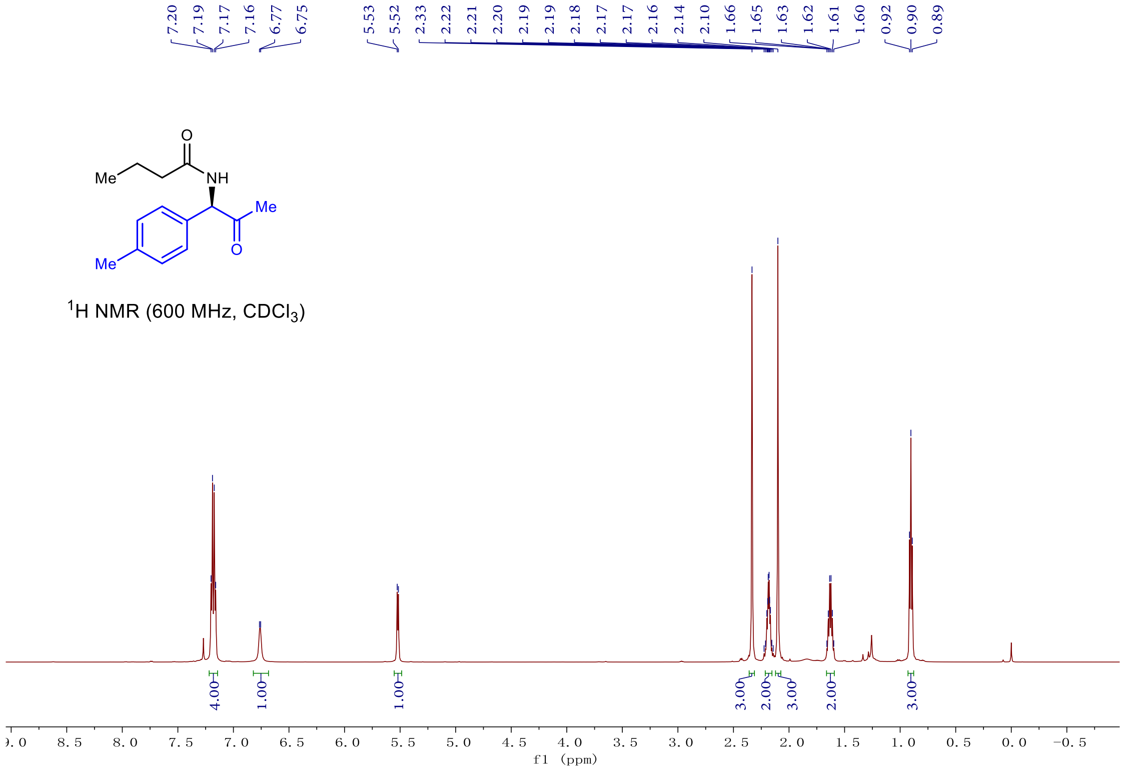

Supplementary Figure 90. ^{1}H NMR spectrum of compound 4a.

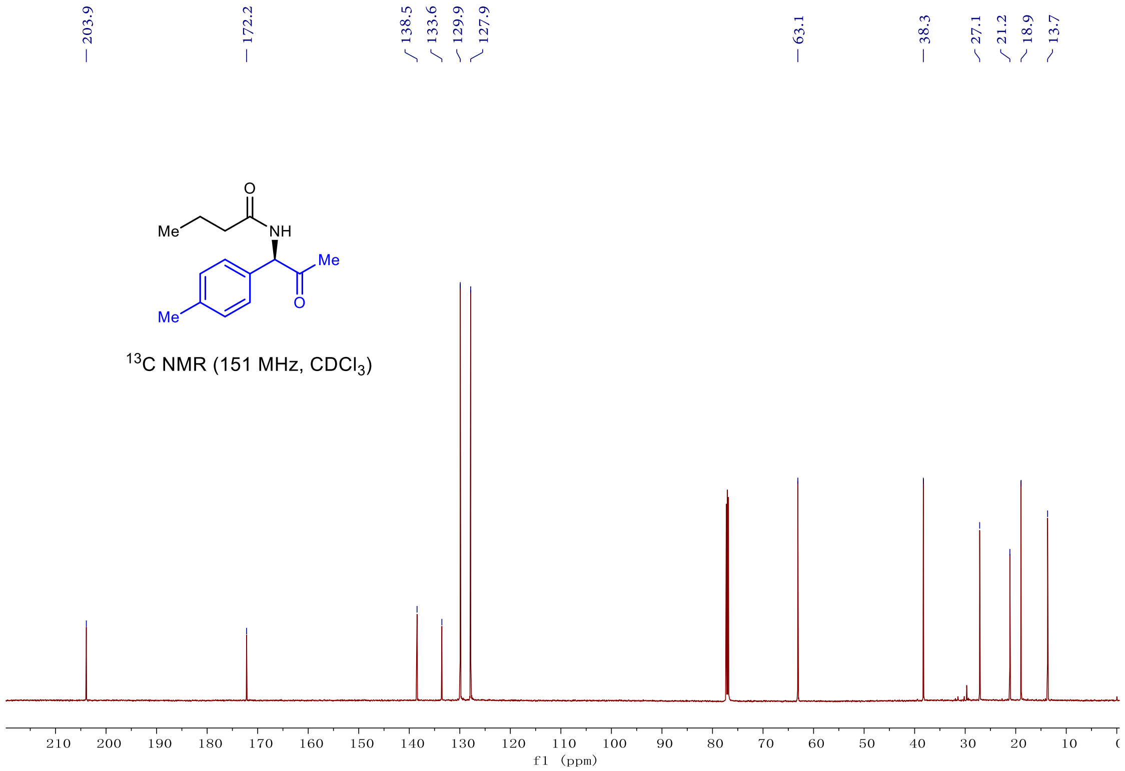

Supplementary Figure 91. ^{13}C NMR spectrum of compound 4a.

(*R*)-N-(2-oxo-1-(*m*-tolyl)propyl)butyramide (4b)

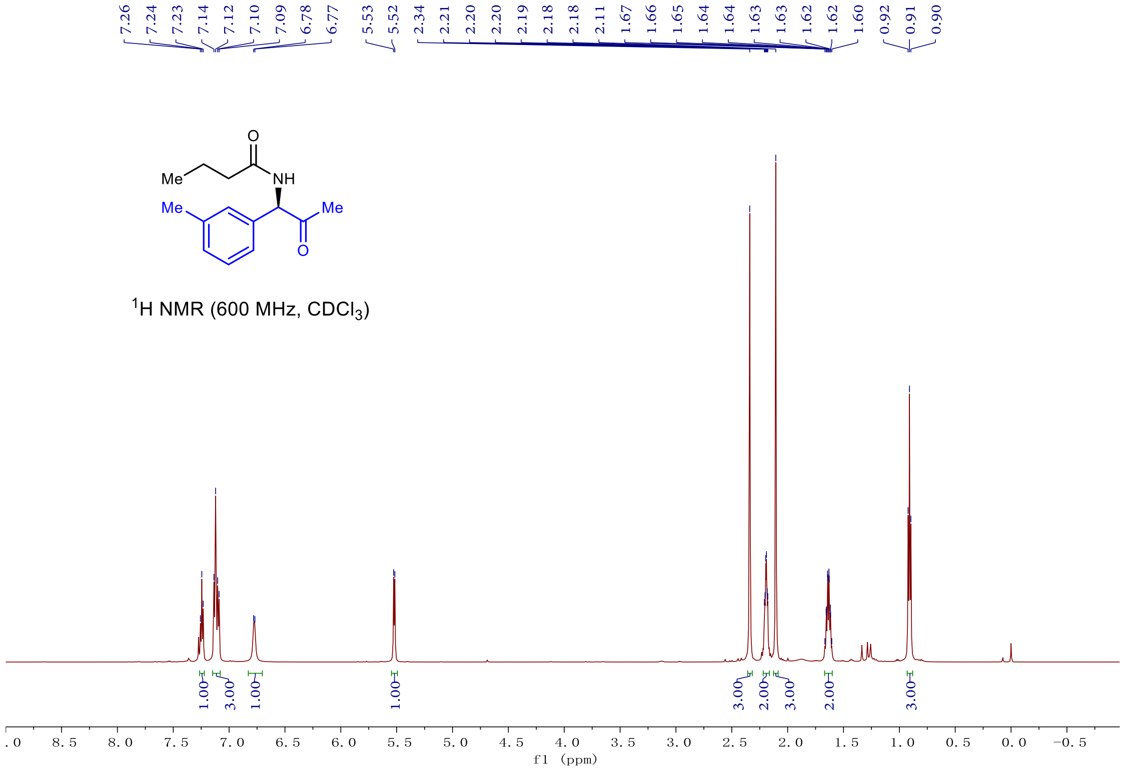

Supplementary Figure 92. ^{1}H NMR spectrum of compound 4b.

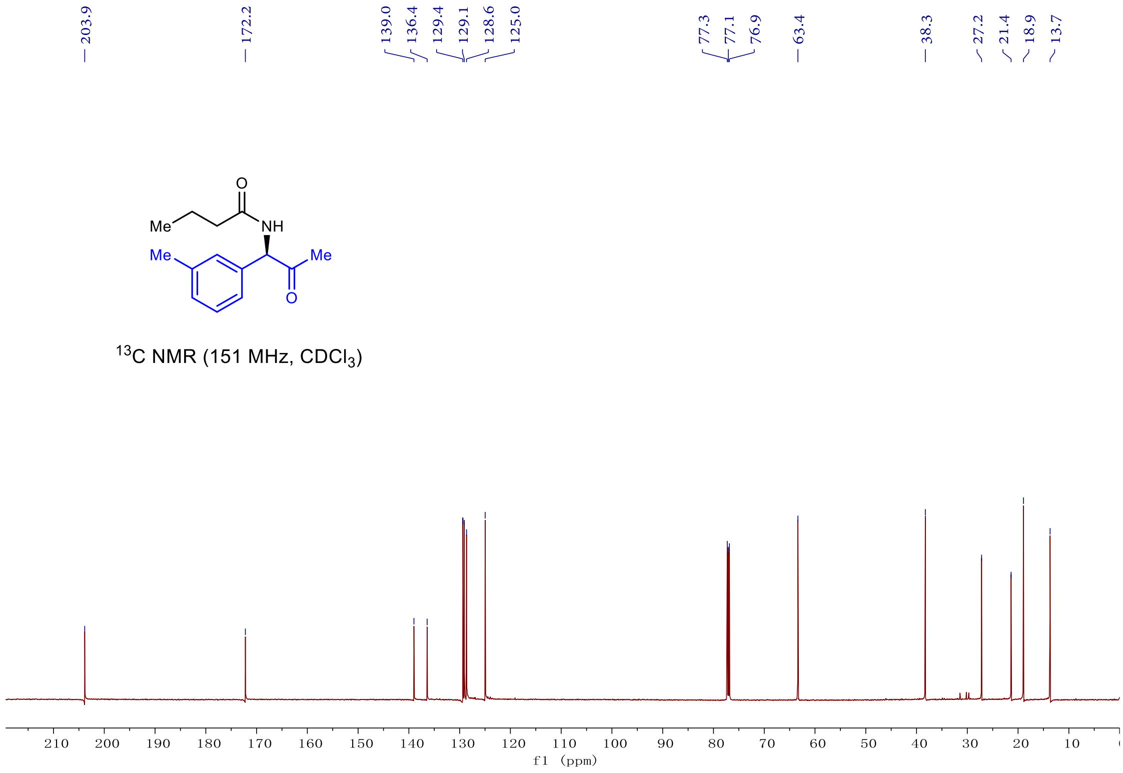

Supplementary Figure 93. ^{13}C NMR spectrum of compound 4b.

(*R*)-N-(1-(3-methoxyphenyl)-2-oxopropyl)butyramide (4c)

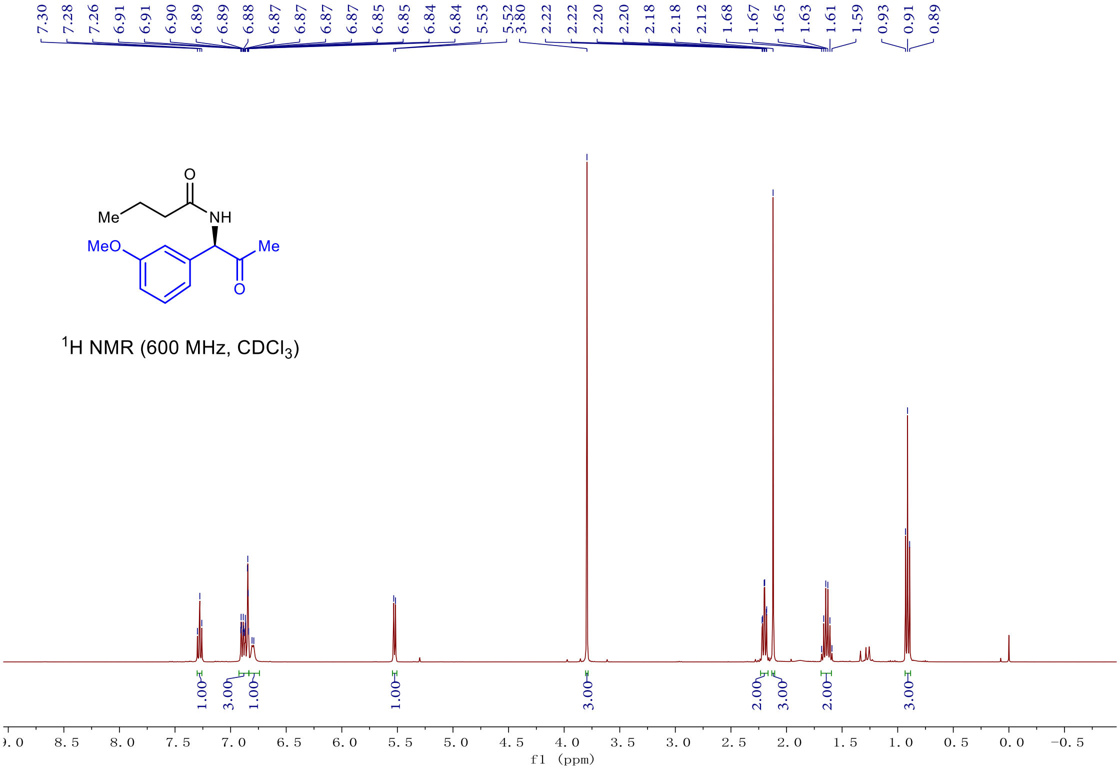

Supplementary Figure 94. ^{1}H NMR spectrum of compound 4c.

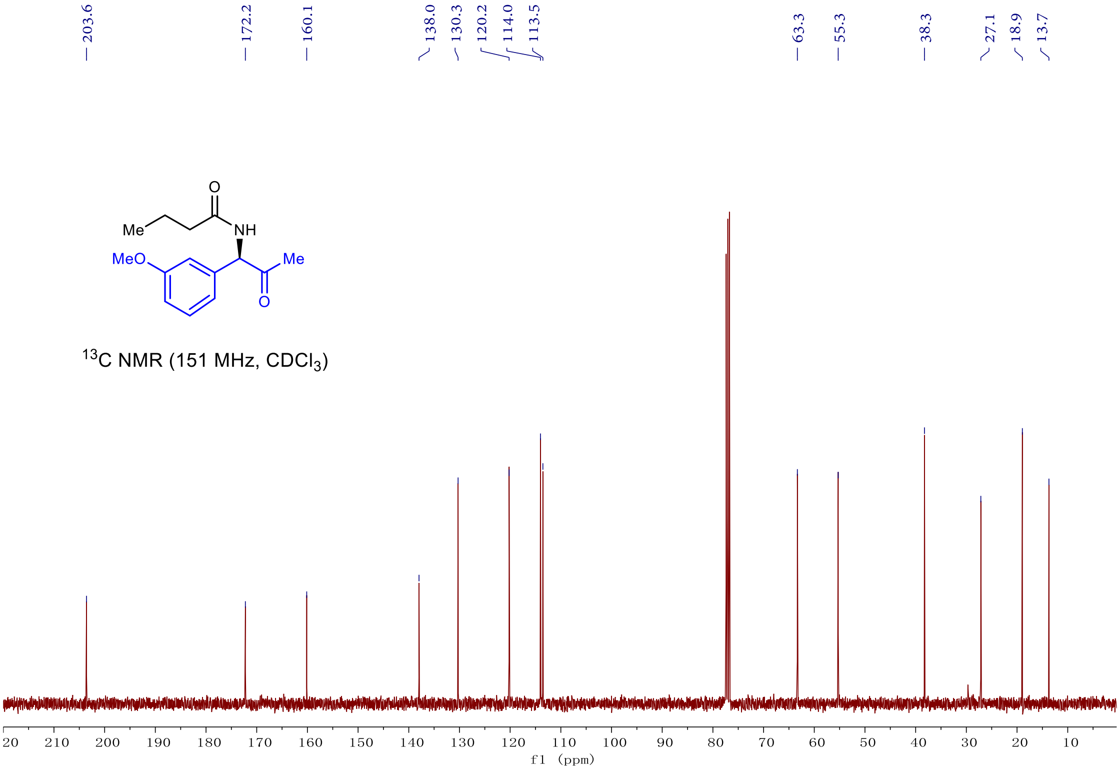

Supplementary Figure 95. ^{13}C NMR spectrum of compound 4c.

(*R*)-N-(2-oxo-1-(4-(trifluoromethyl)phenyl)propyl)butyramide (4d)

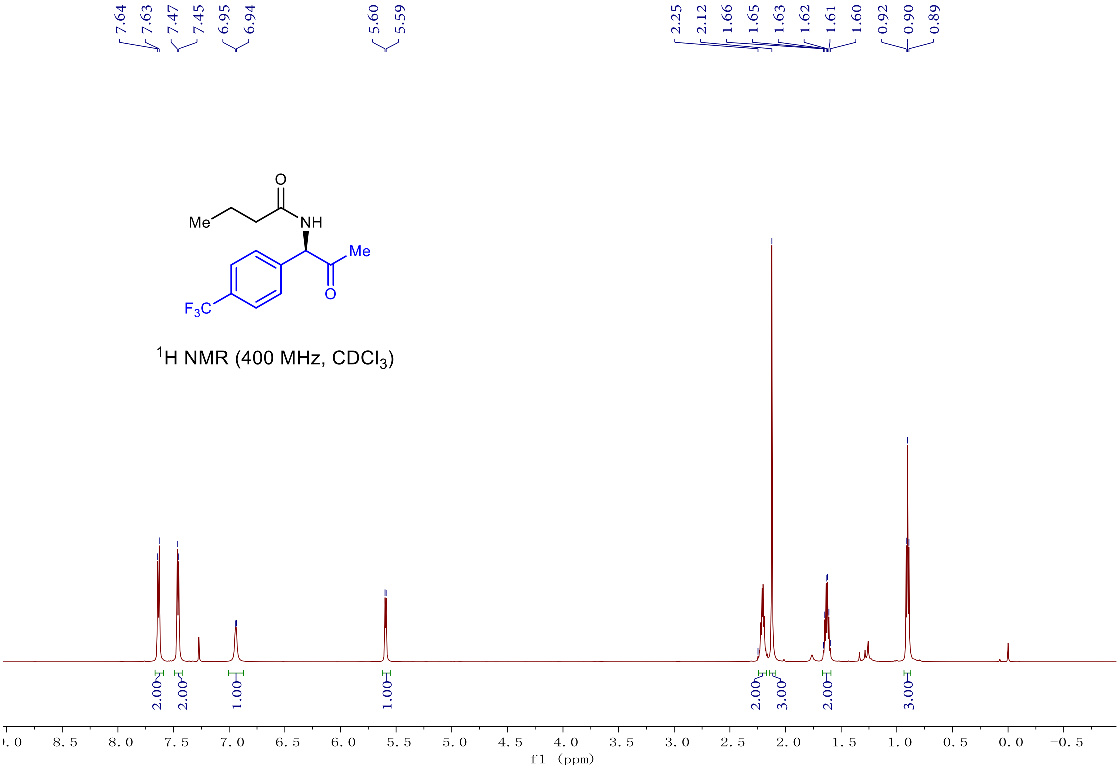

Supplementary Figure 96. ^{1}H NMR spectrum of compound 4d.

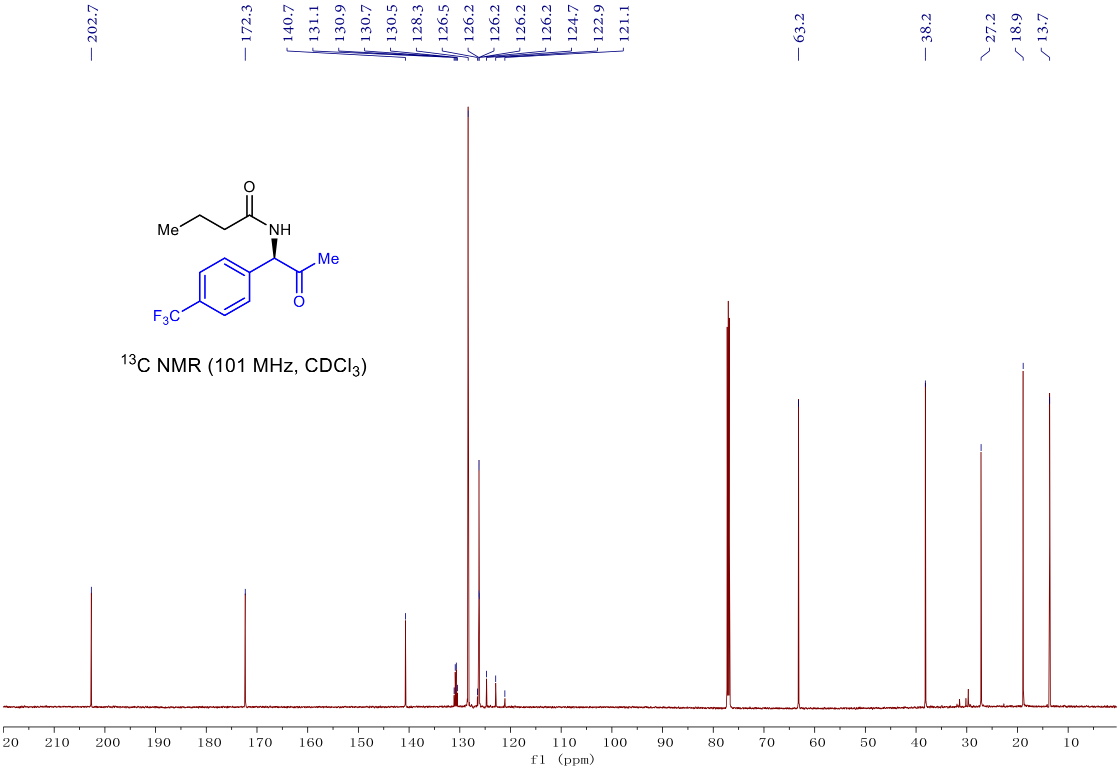

Supplementary Figure 97. ^{13}C NMR spectrum of compound 4d.

(*R*)-N-(1-(2-fluorophenyl)-2-oxopropyl)butyramide (4e)

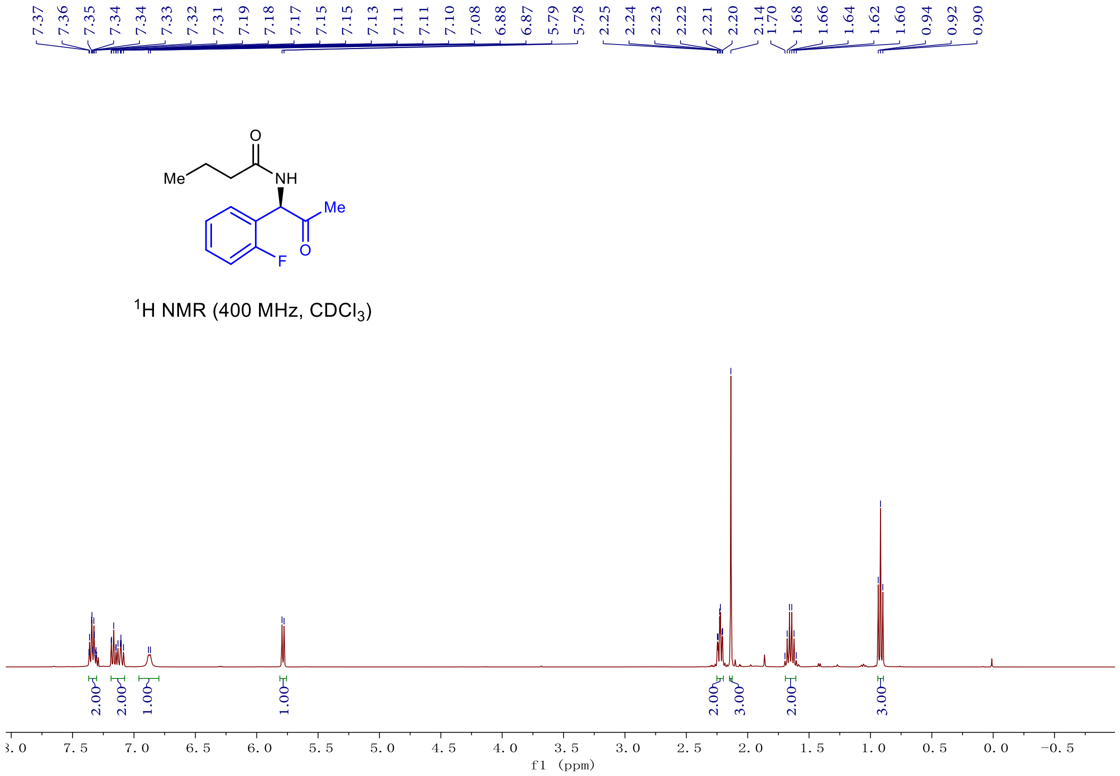

Supplementary Figure 98. ^{1}H NMR spectrum of compound 4e.

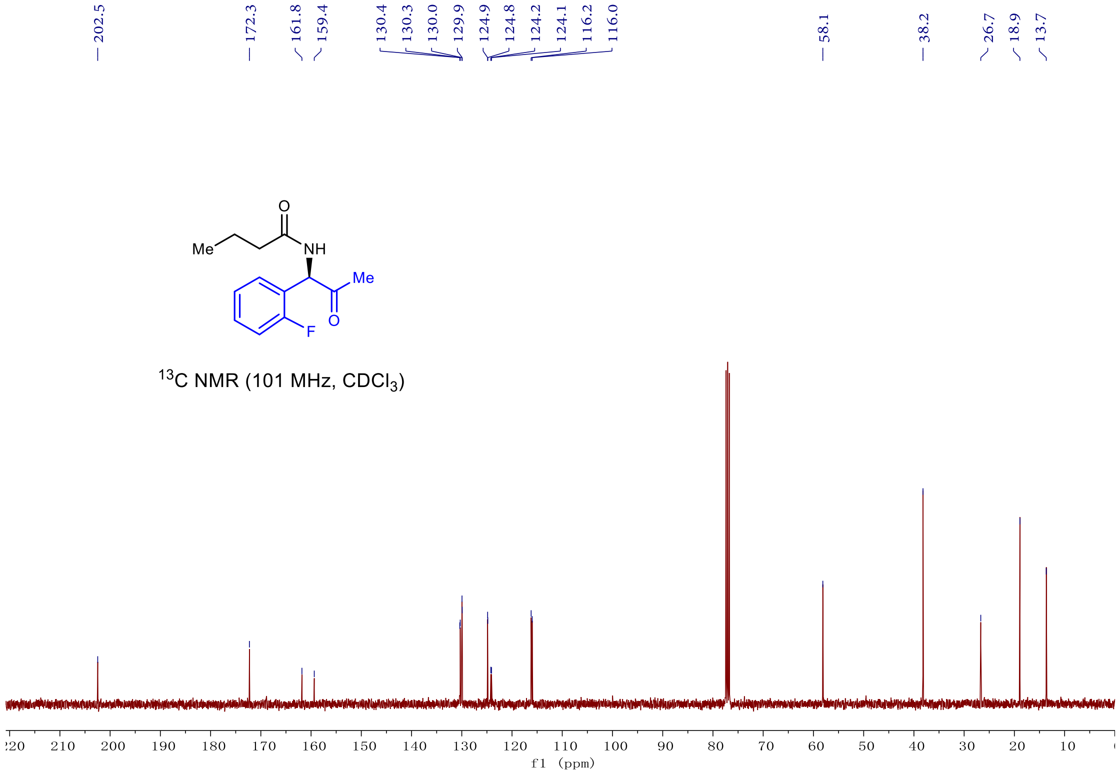

Supplementary Figure 99. ^{13}C NMR spectrum of compound 4e.

(*R*)-N-(1-(4-fluorophenyl)-2-oxopropyl)butyramide (4f)

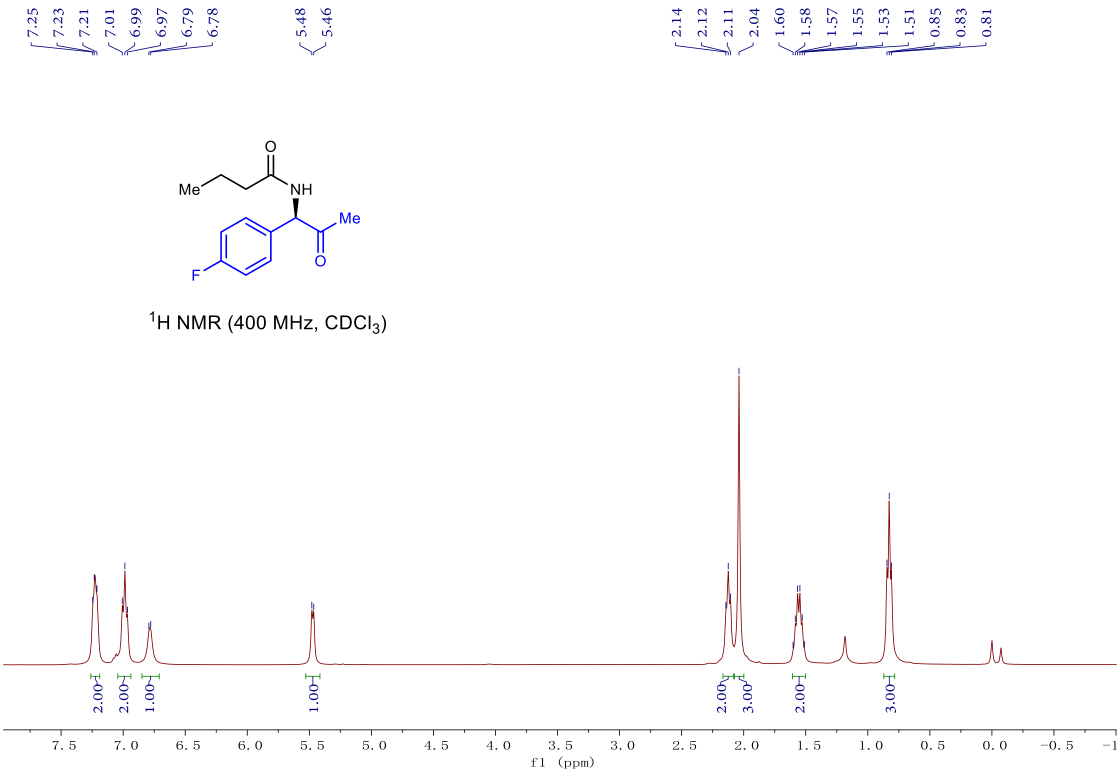

Supplementary Figure 100. ^{1}H NMR spectrum of compound 4f.

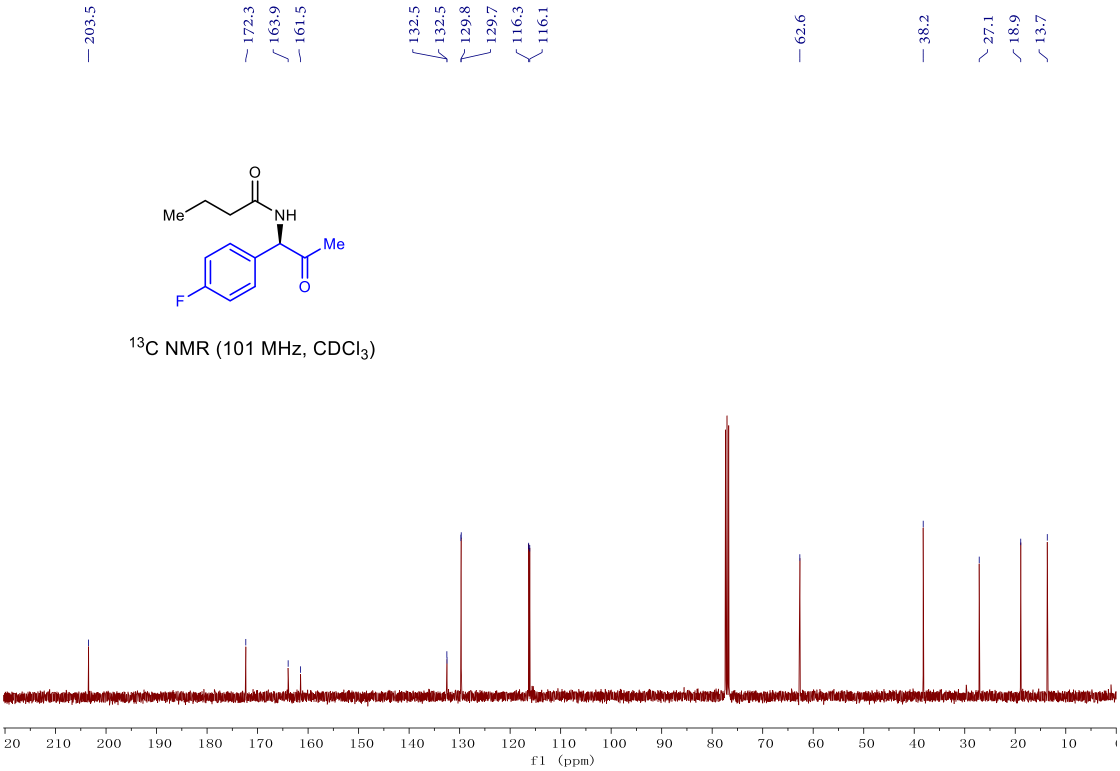

Supplementary Figure 101. ^{13}C NMR spectrum of compound 4f.

(*R*)-N-(1-(4-chlorophenyl)-2-oxopropyl)butyramide (4g)

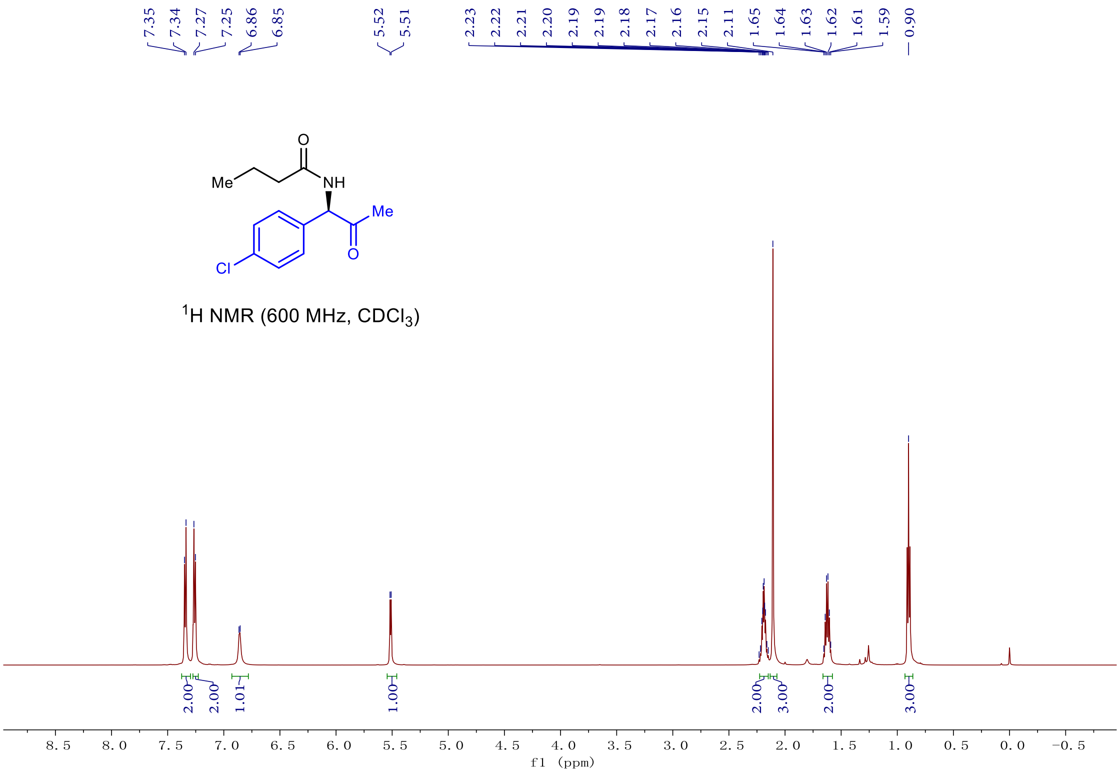

Supplementary Figure 102. ^{1}H NMR spectrum of compound 4g.

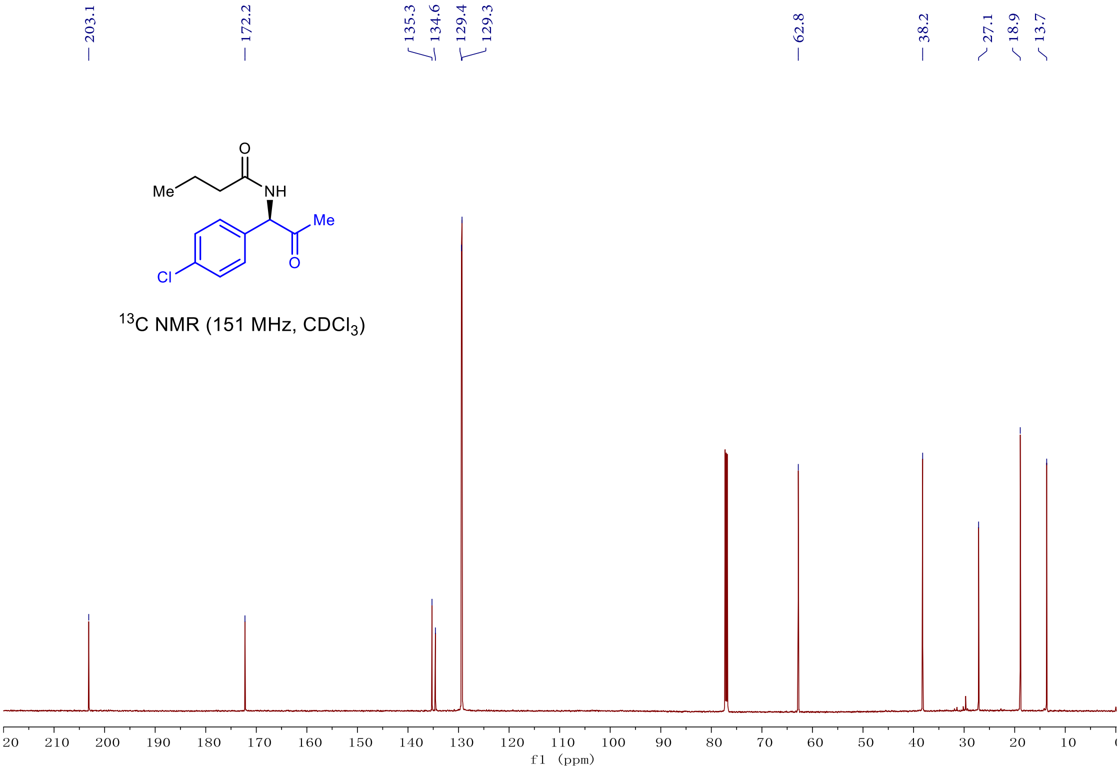

Supplementary Figure 103. ^{13}C NMR spectrum of compound 4g.

(*R*)-N-(1-(4-bromophenyl)-2-oxopropyl)butyramide (4h)

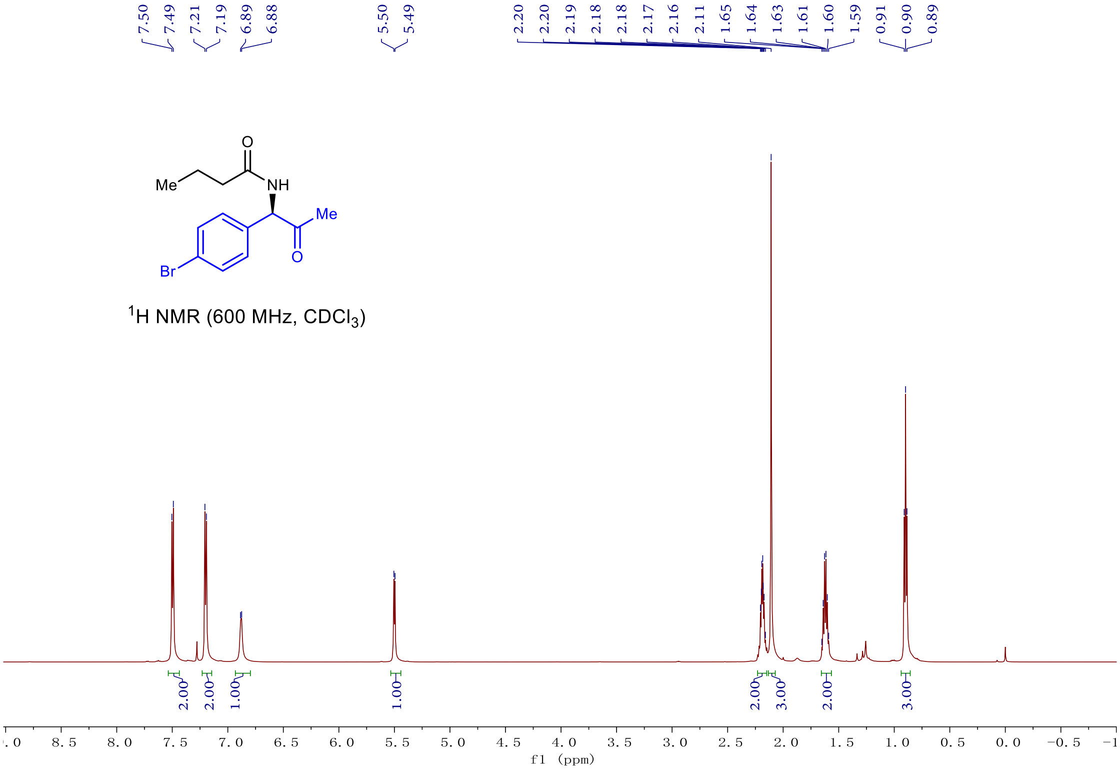

Supplementary Figure 104. ^{1}H NMR spectrum of compound 4h.

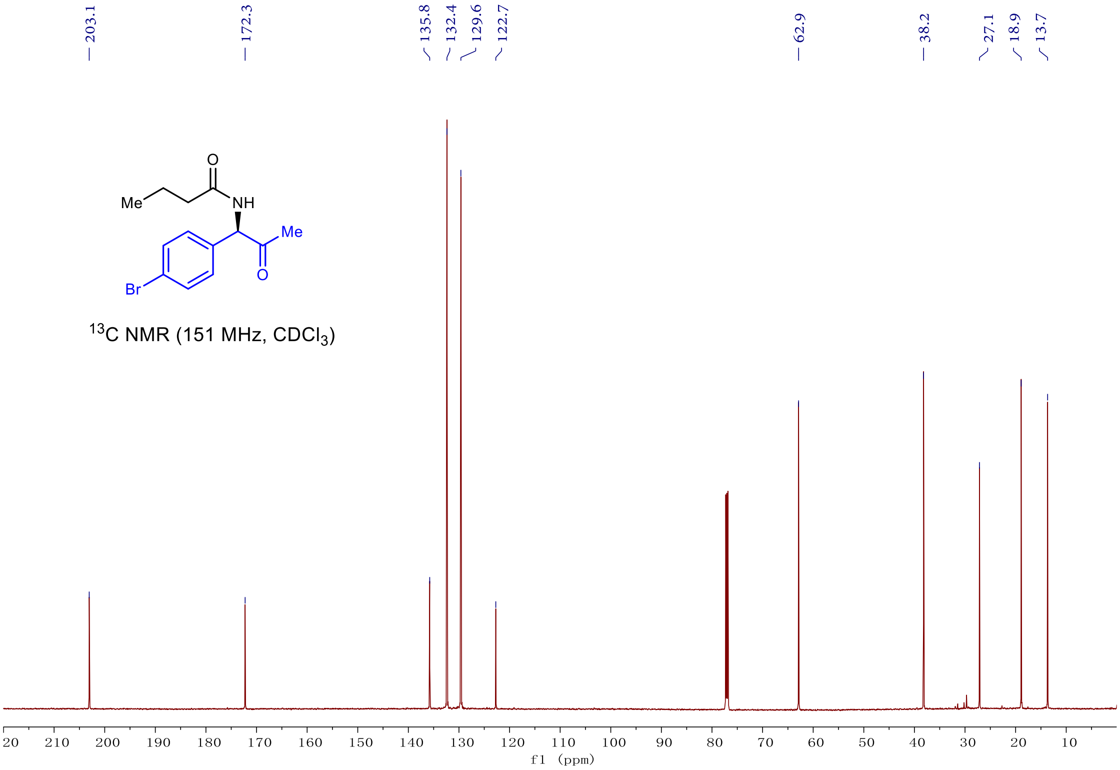

Supplementary Figure 105. ^{13}C NMR spectrum of compound 4h.

(*R*)-N-(1-(4-iodophenyl)-2-oxopropyl)butyramide (4i)

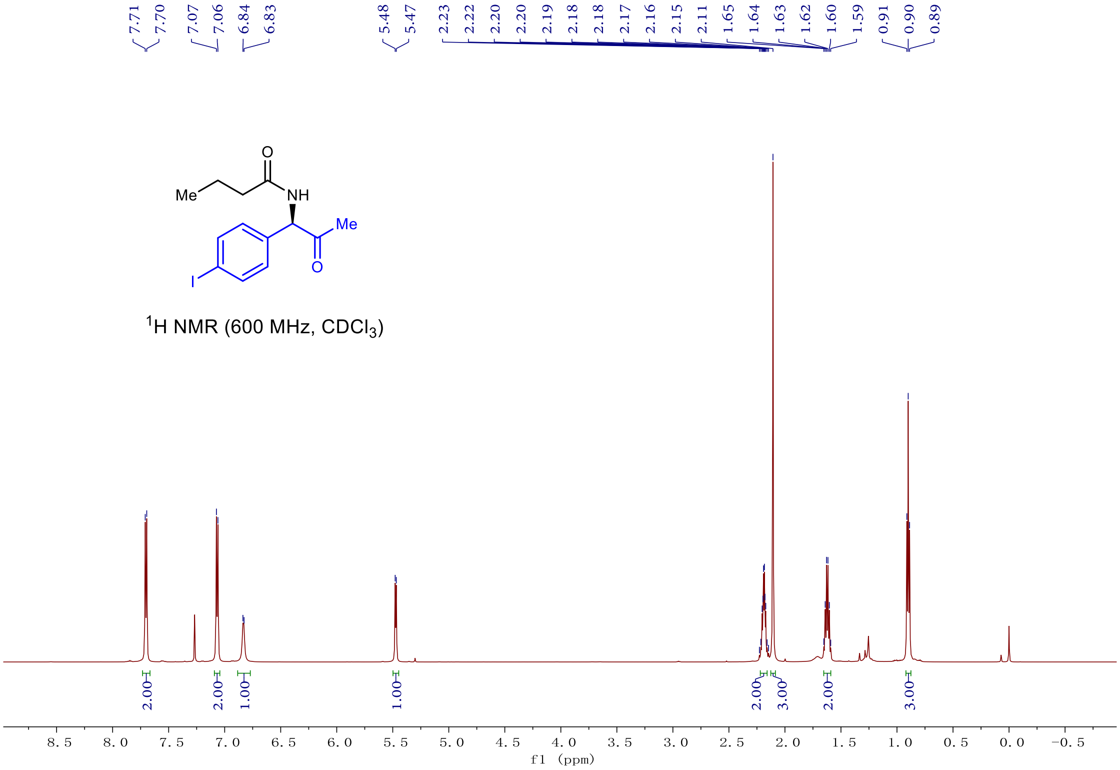

Supplementary Figure 106. ^{1}H NMR spectrum of compound 4i.

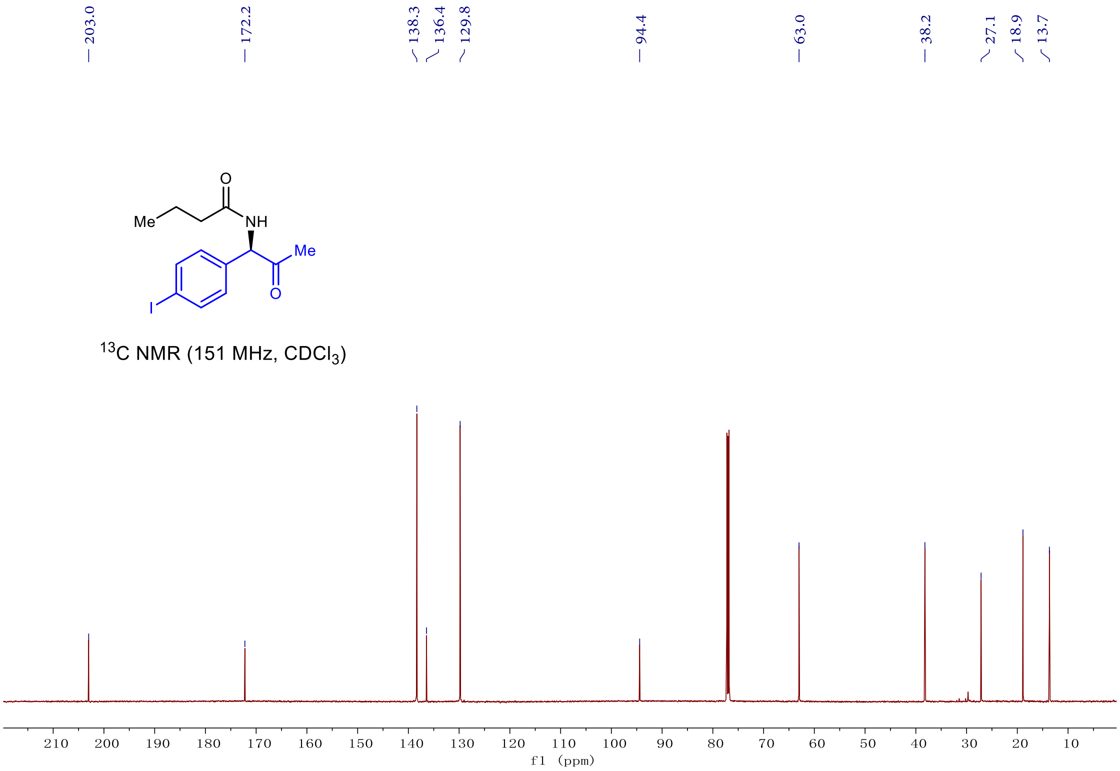

Supplementary Figure 107. ^{13}C NMR spectrum of compound 4i.

(*R*)-N-(1-(naphthalen-2-yl)-2-oxopropyl)butyramide (4j)

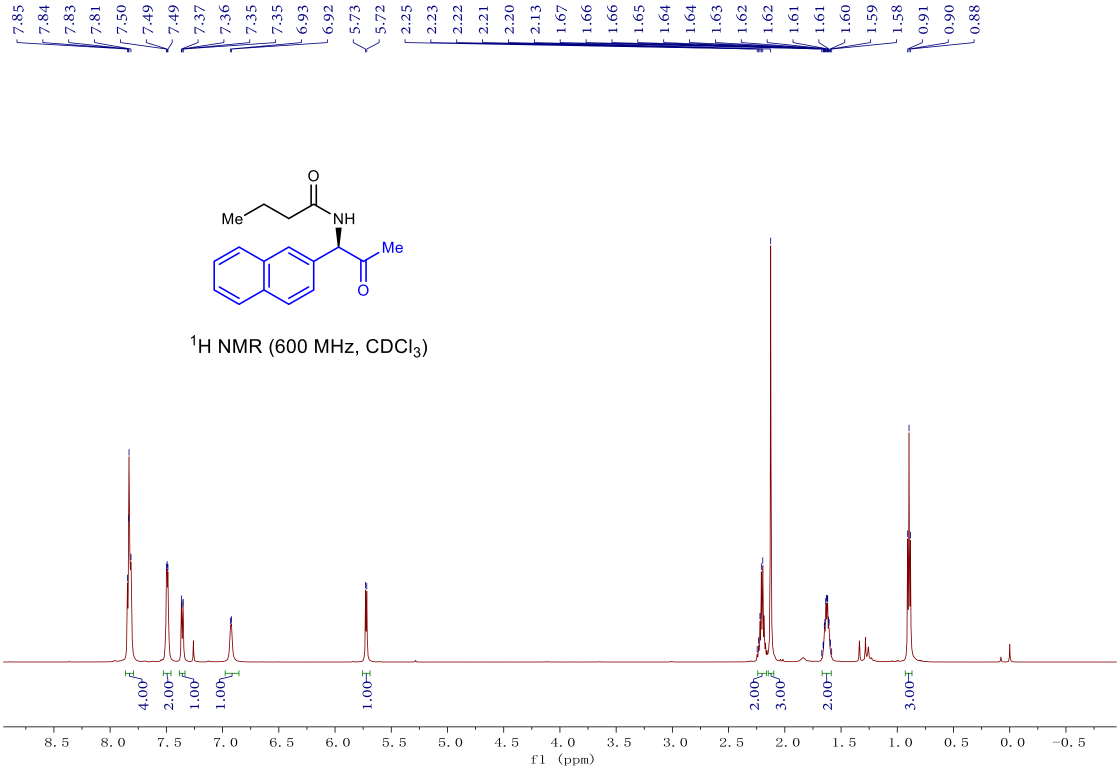

Supplementary Figure 108. ^{1}H NMR spectrum of compound 4j.

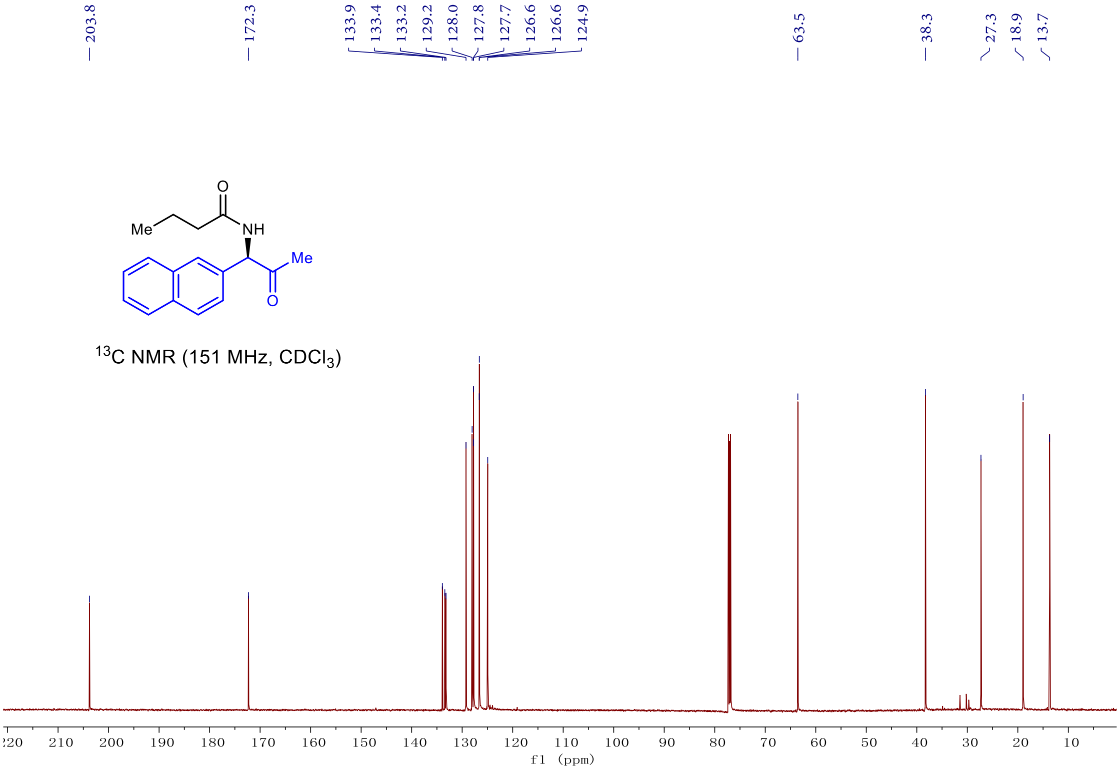

Supplementary Figure 109. ^{13}C NMR spectrum of compound 4j.

(*R*)-N-(1-([1,1'-biphenyl]-4-yl)-2-oxopropyl)butyramide (4k)

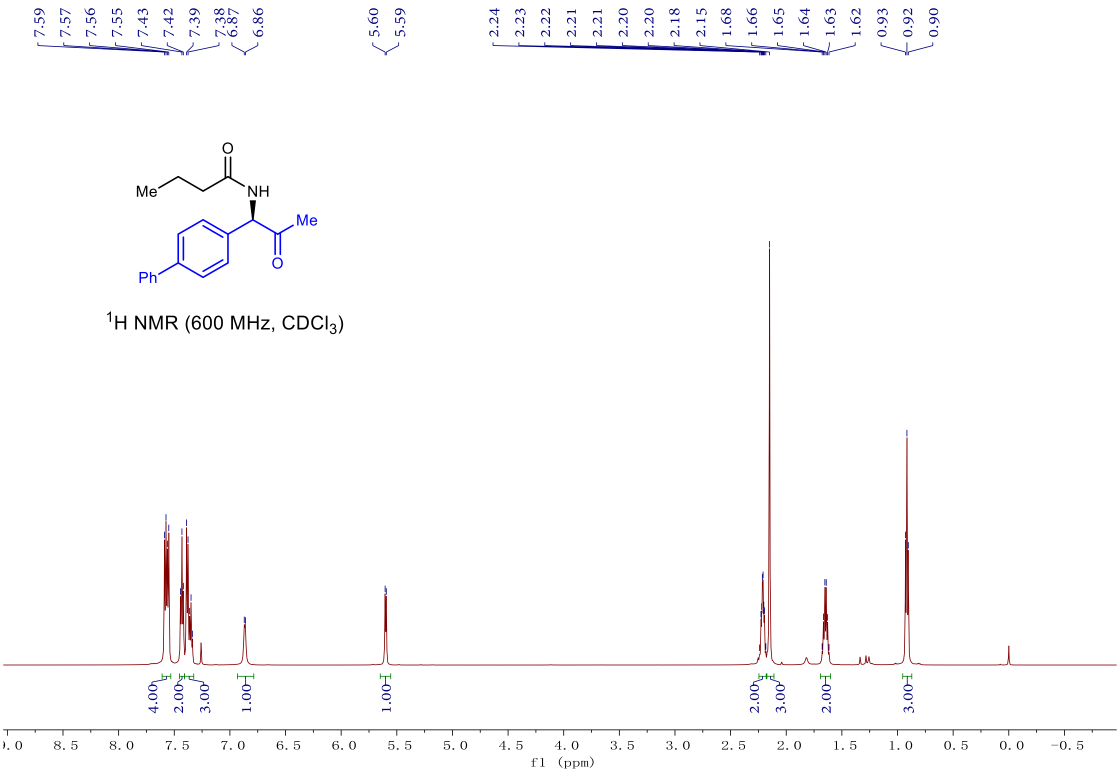

Supplementary Figure 110. ^{1}H NMR spectrum of compound 4k.

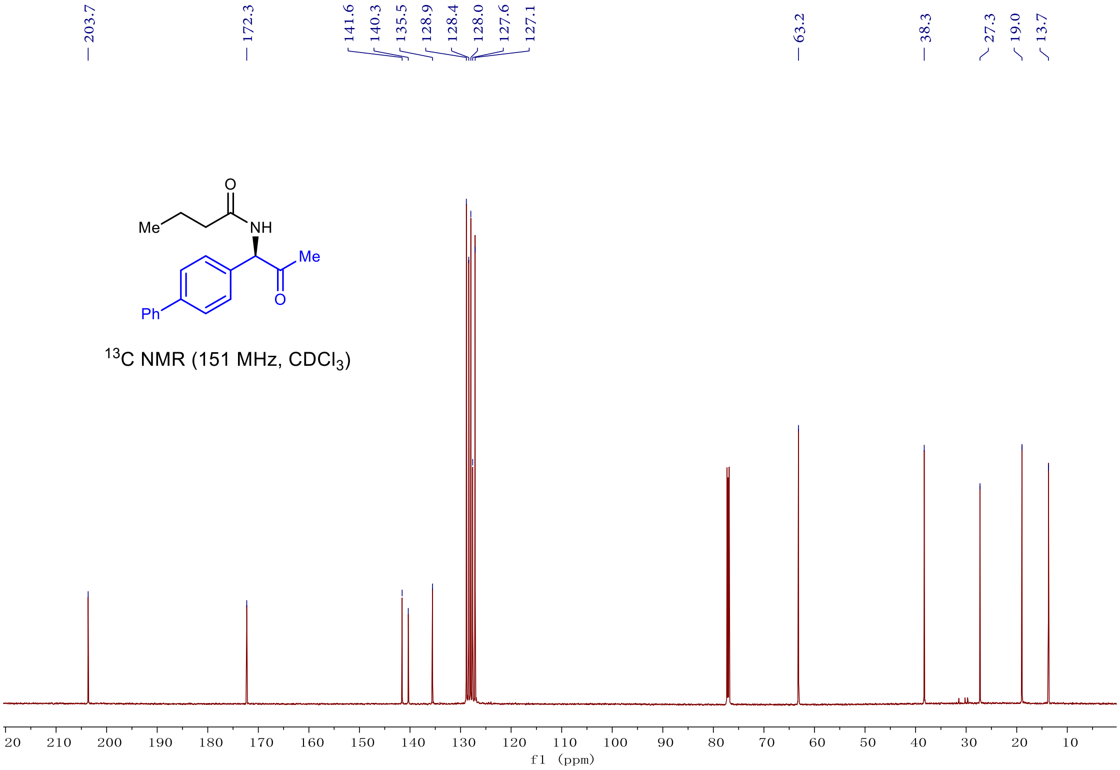

Supplementary Figure 111. ^{13}C NMR spectrum of compound 4k.

(*R*)-N-(2-oxo-1-phenylbutyl)butyramide (4l)

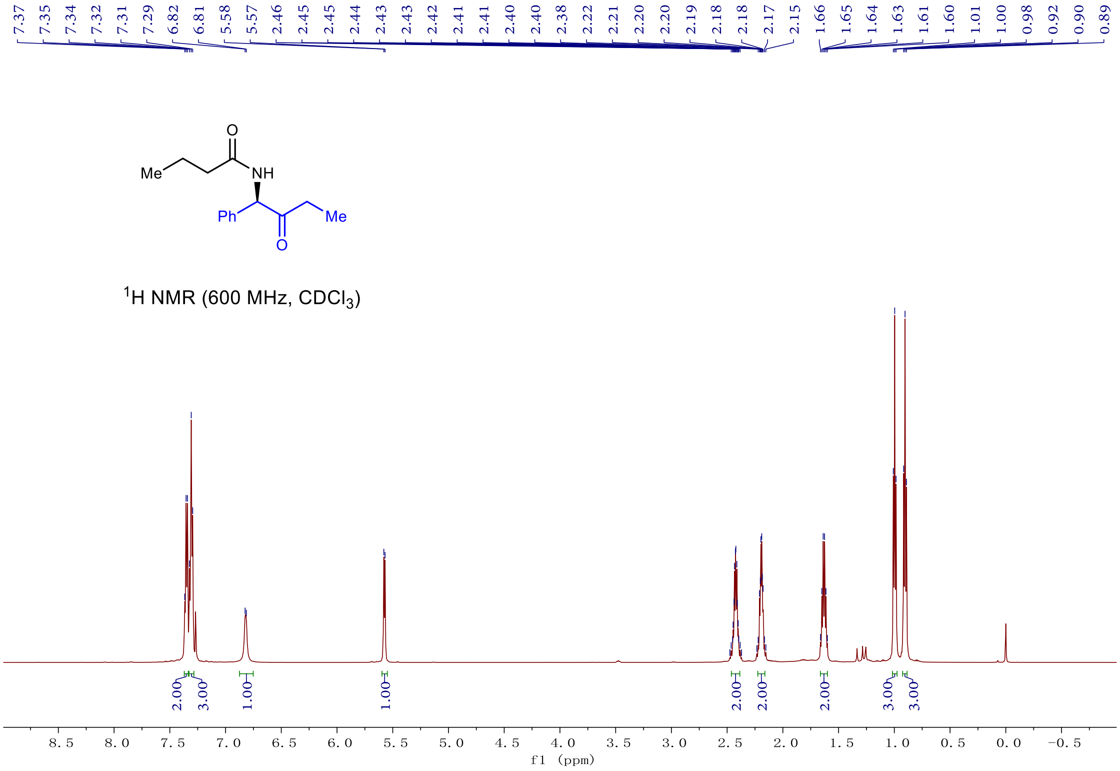

Supplementary Figure 112. ^{1}H NMR spectrum of compound 4l.

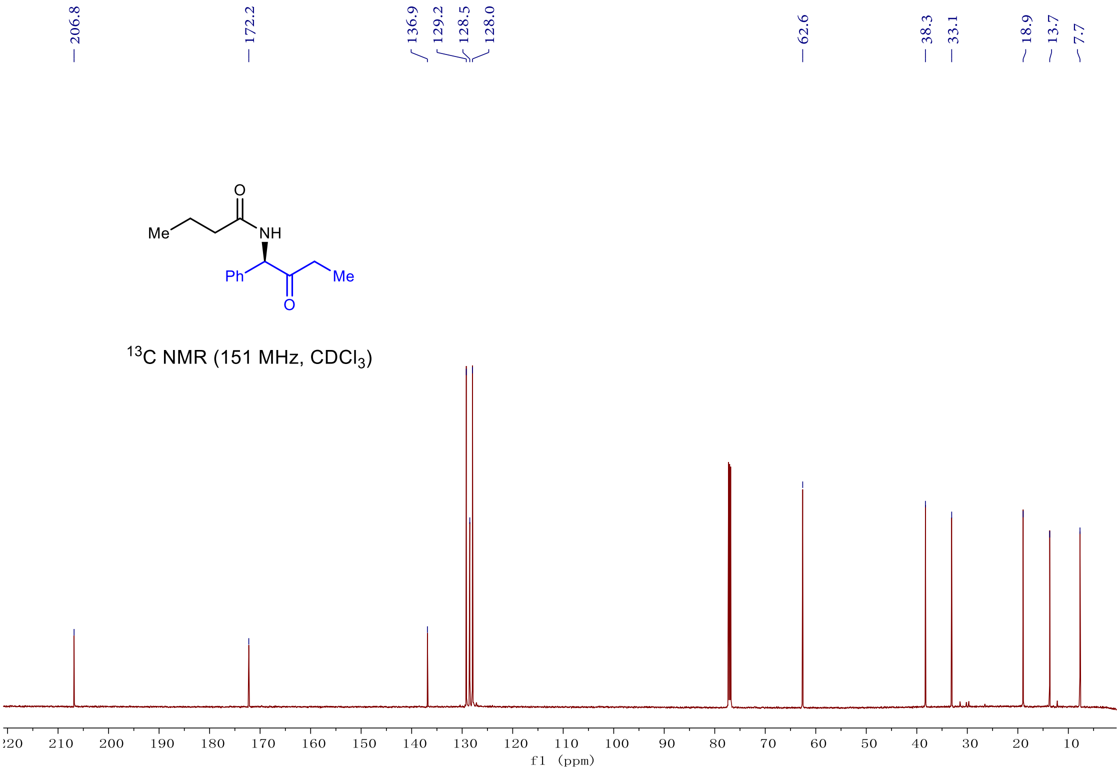

Supplementary Figure 113. ^{13}C NMR spectrum of compound 4l.

(*R*)-N-(2-oxo-1-(p-tolyl)propyl)benzamide (4m)

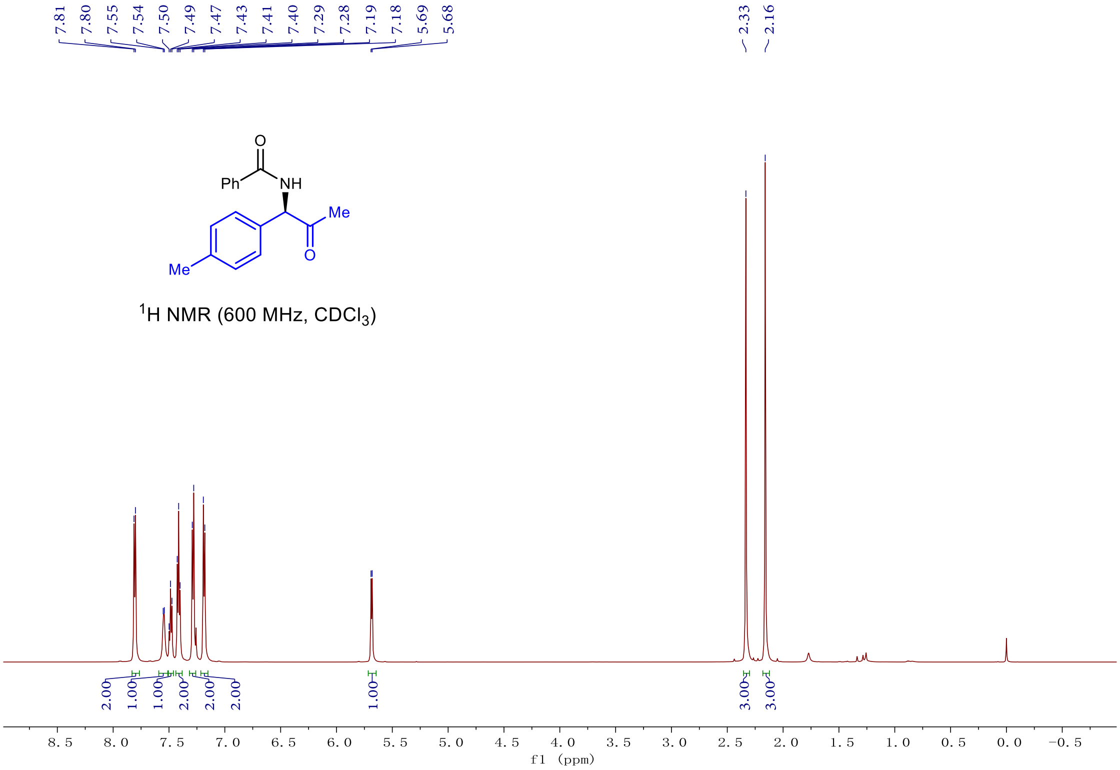

Supplementary Figure 114. ^{1}H NMR spectrum of compound 4m.

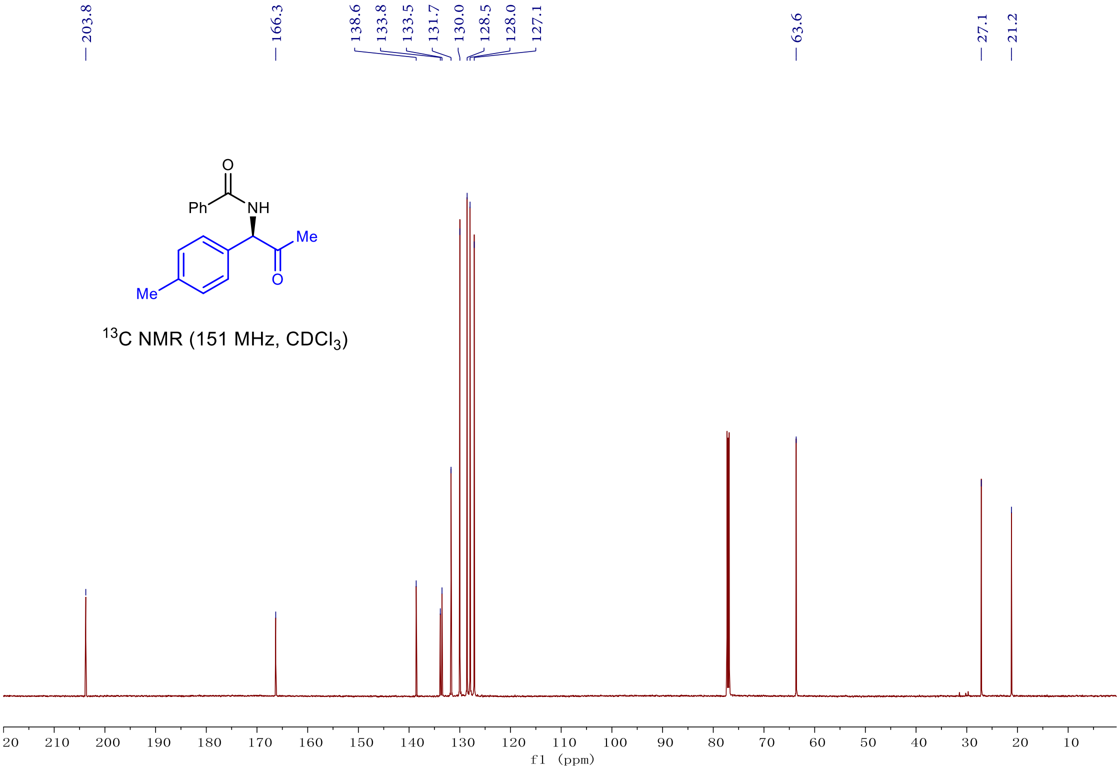

Supplementary Figure 115. ^{13}C NMR spectrum of compound 4m.

(*R*)-N-(1-(naphthalen-2-yl)-2-oxopropyl)benzamide (4n)

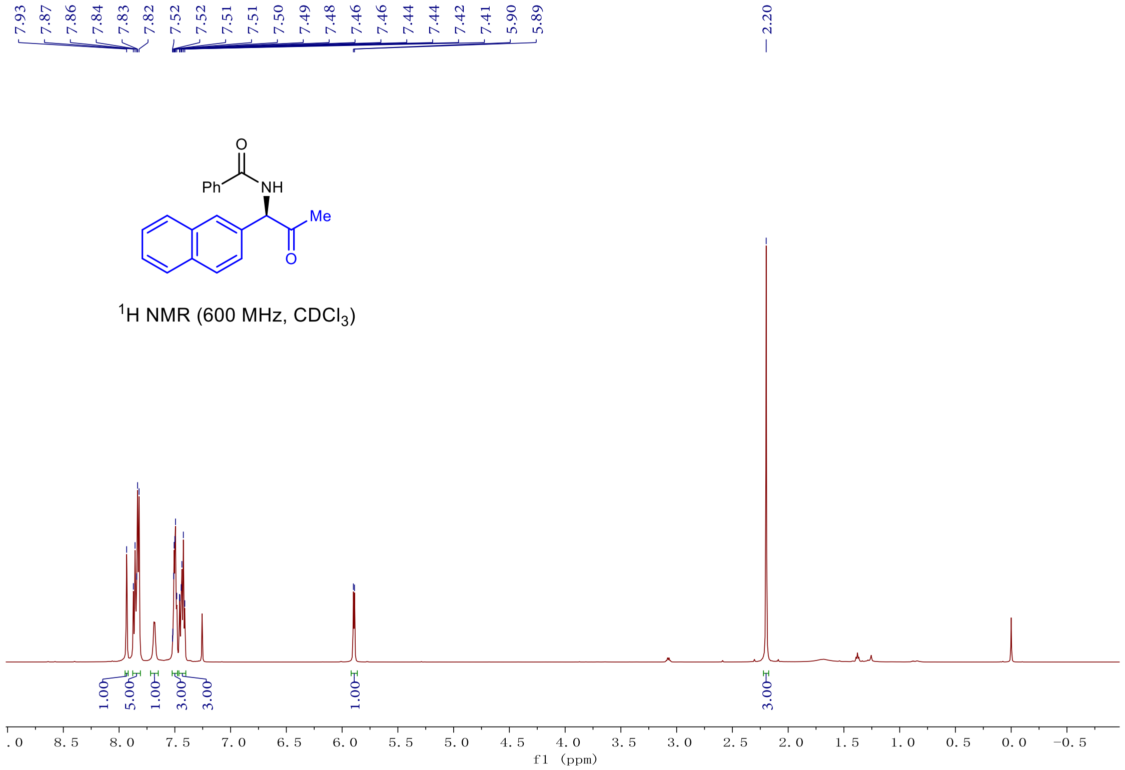

Supplementary Figure 116. ^{1}H NMR spectrum of compound 4n.

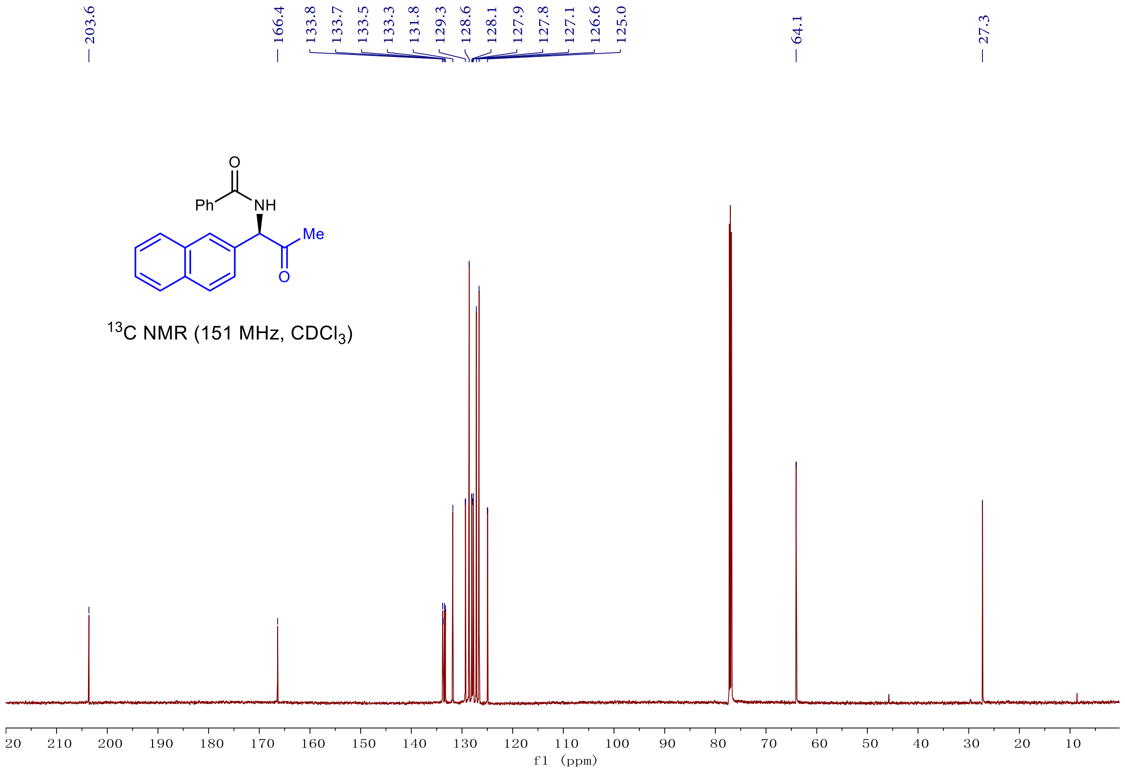

Supplementary Figure 117. ^{13}C NMR spectrum of compound 4n.

Methyl (*R*)-3-((1-(4-fluorophenyl)-2-oxopropyl)carbamoyl)bicyclo[1.1.1]pentane-1-carboxylate (4o)

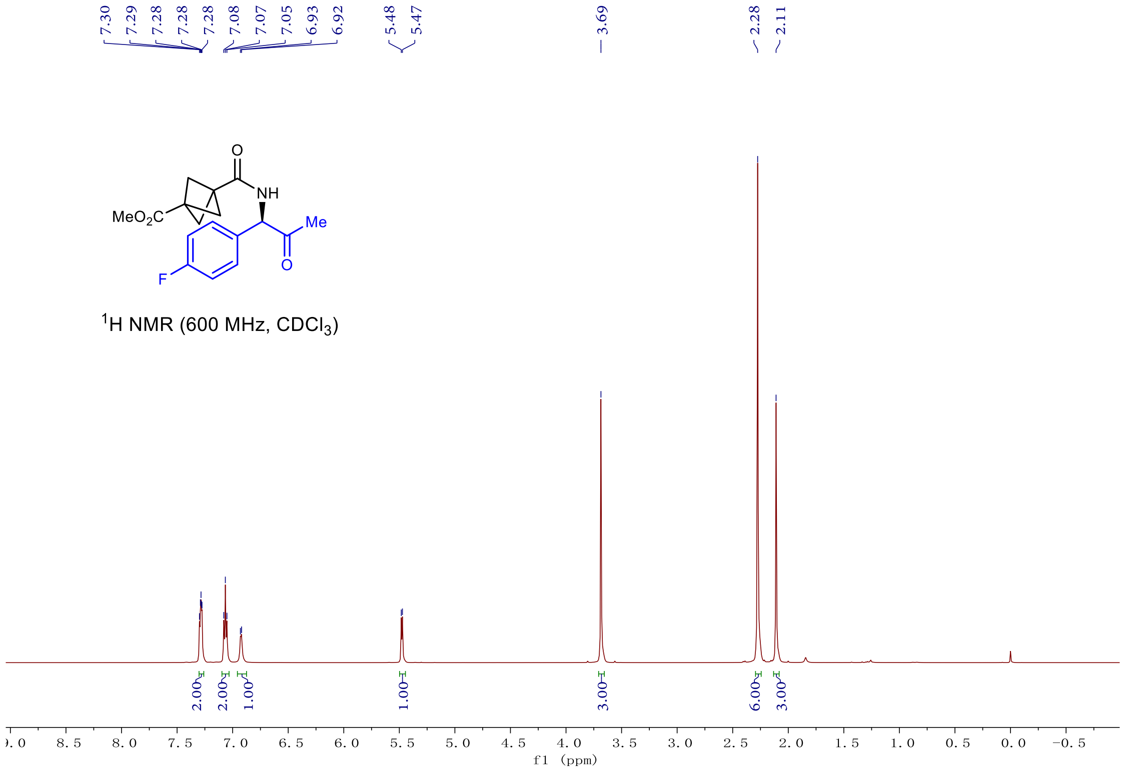

Supplementary Figure 118. ^{1}H NMR spectrum of compound 4o.

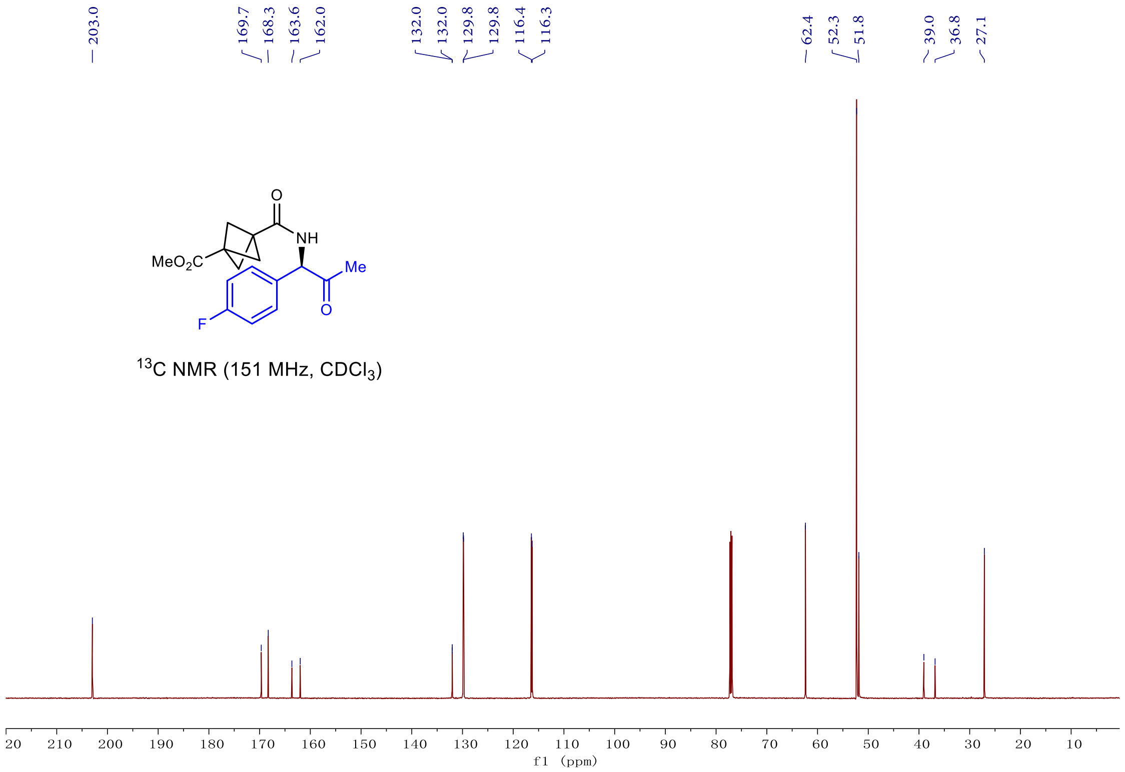

Supplementary Figure 119. ^{13}C NMR spectrum of compound 4o.

Methyl (*R*)-3-((1-(4-chlorophenyl)-2-oxopropyl)carbamoyl)bicyclo[1.1.1]pentane-1-carboxylate (4p)

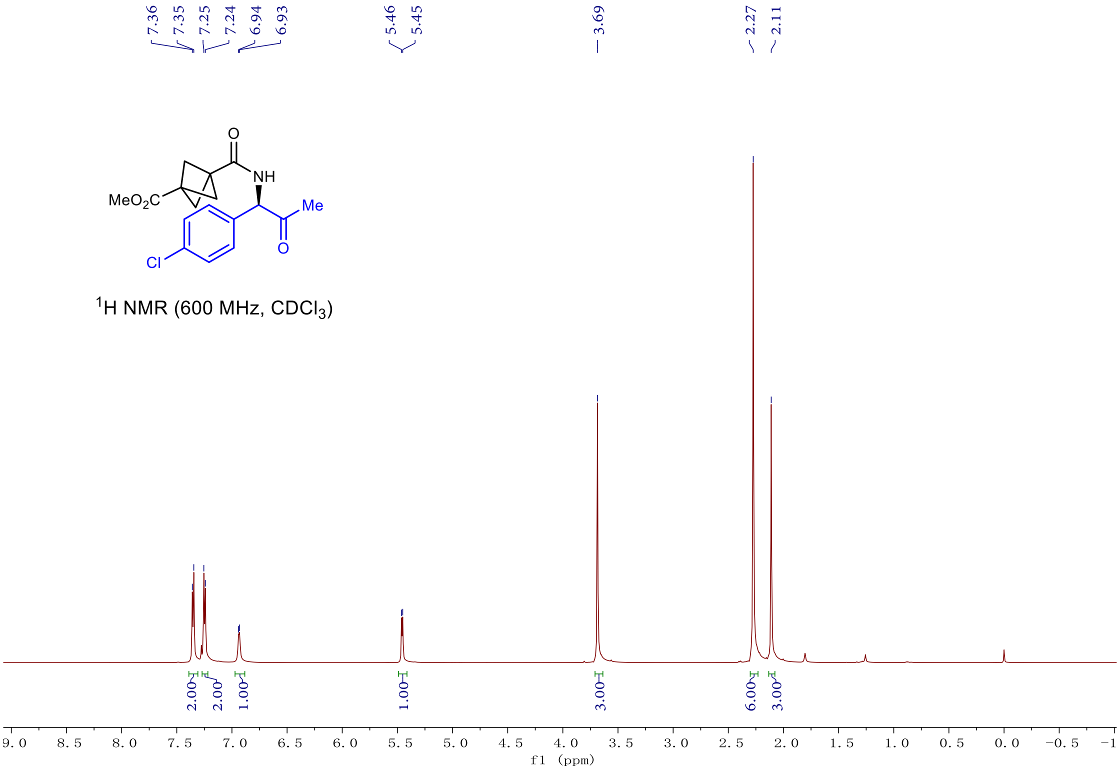

Supplementary Figure 120. ^{1}H NMR spectrum of compound 4p.

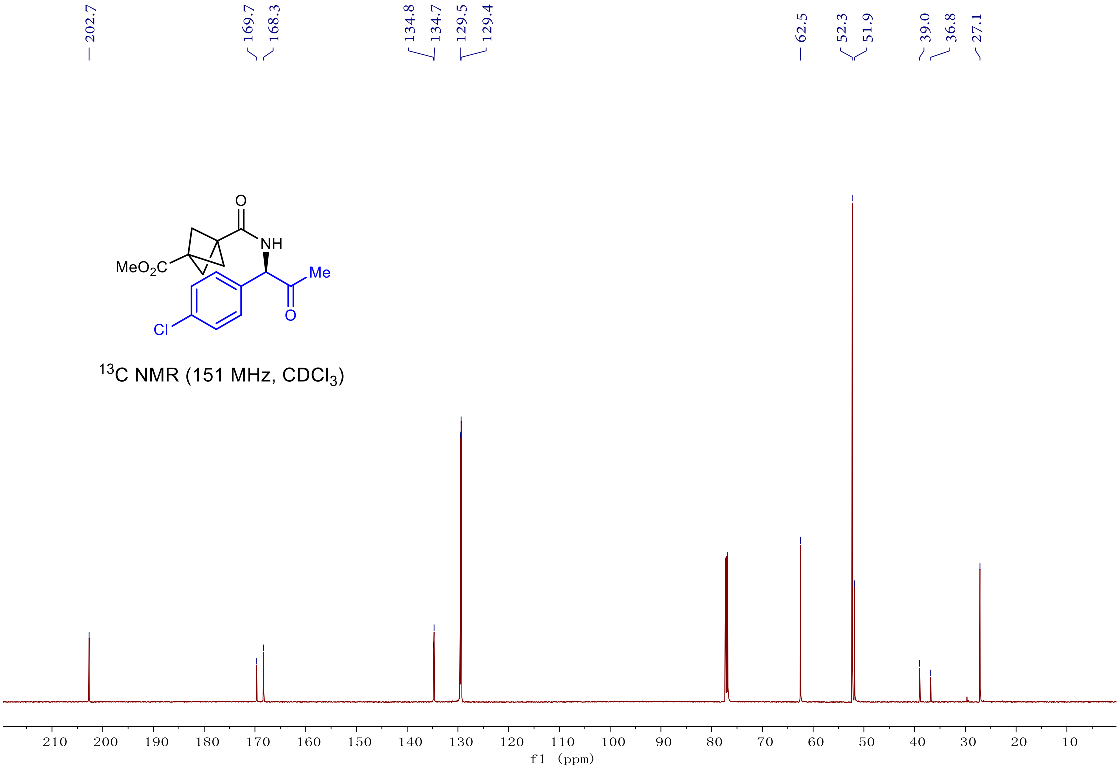

Supplementary Figure 121. ^{13}C NMR spectrum of compound 4p.

Methyl (*R*)-3-((1-(4-bromophenyl)-2-oxopropyl)carbamoyl)bicyclo[1.1.1]pentane-1-carboxylate (4q)

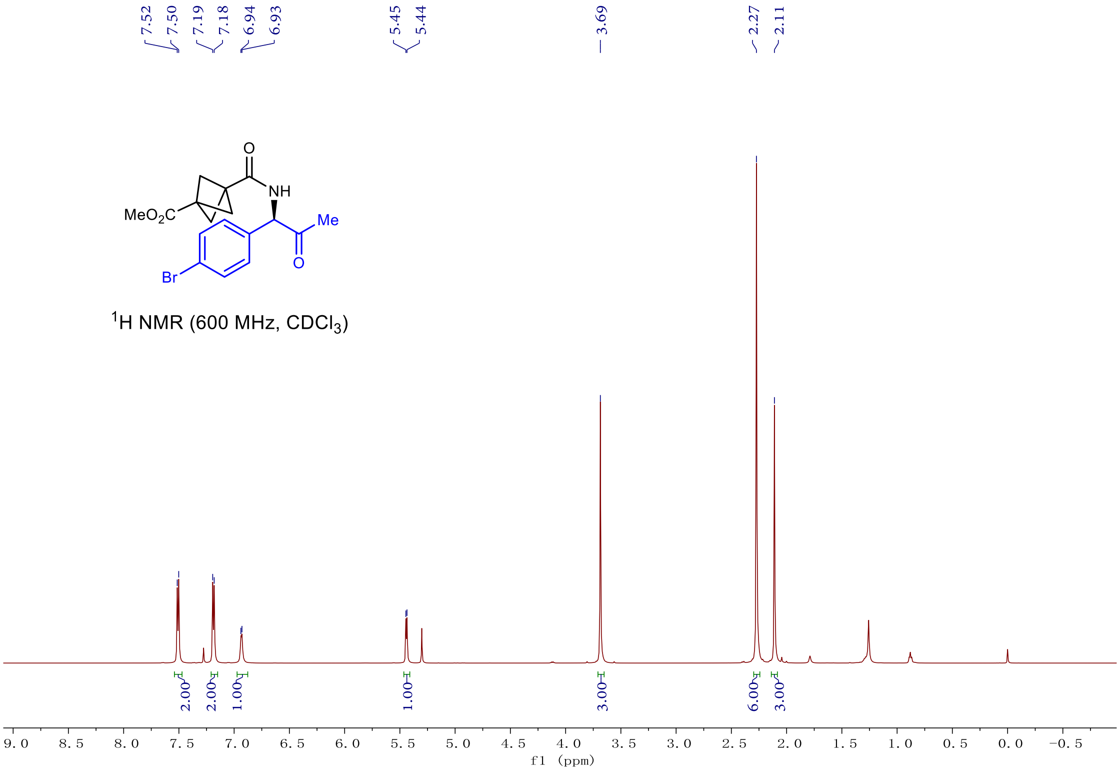

Supplementary Figure 122. ^{1}H NMR spectrum of compound 4q.

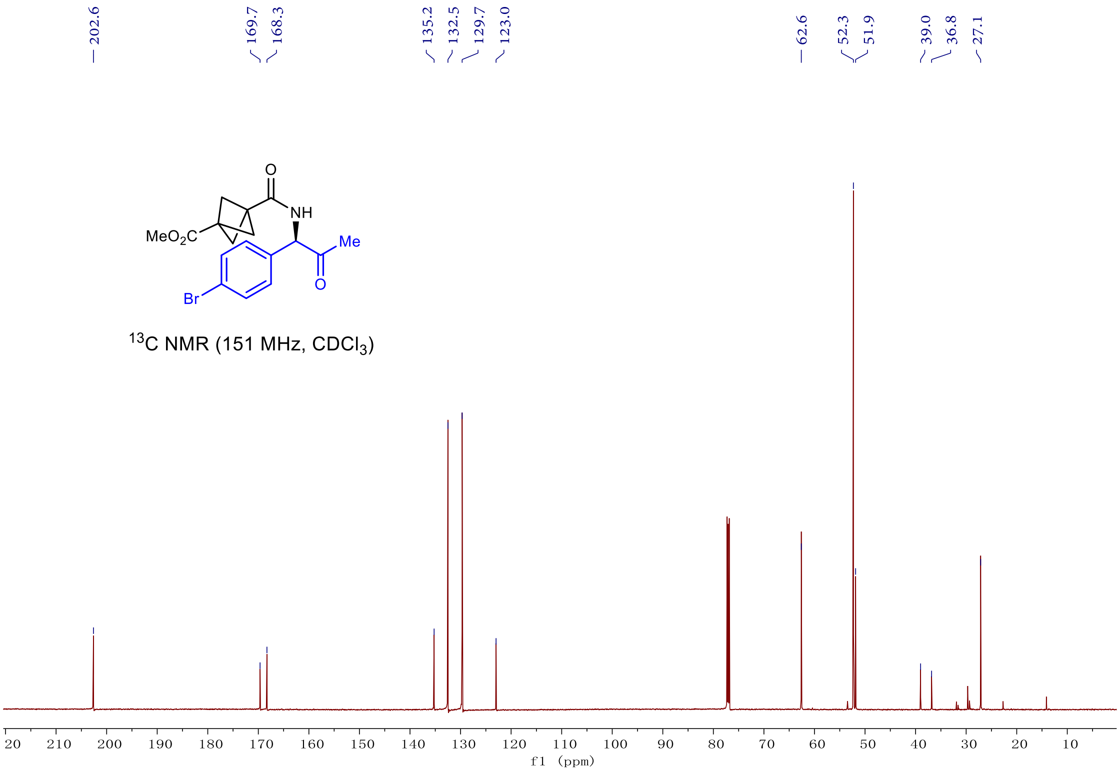

Supplementary Figure 123. ^{13}C NMR spectrum of compound 4q.

(*R*)-2-(1-(4-chlorobenzoyl)-5-methoxy-2-methyl-1H-indol-3-yl)-N-(2-oxo-1-phenylpropyl)acetamide (5a)

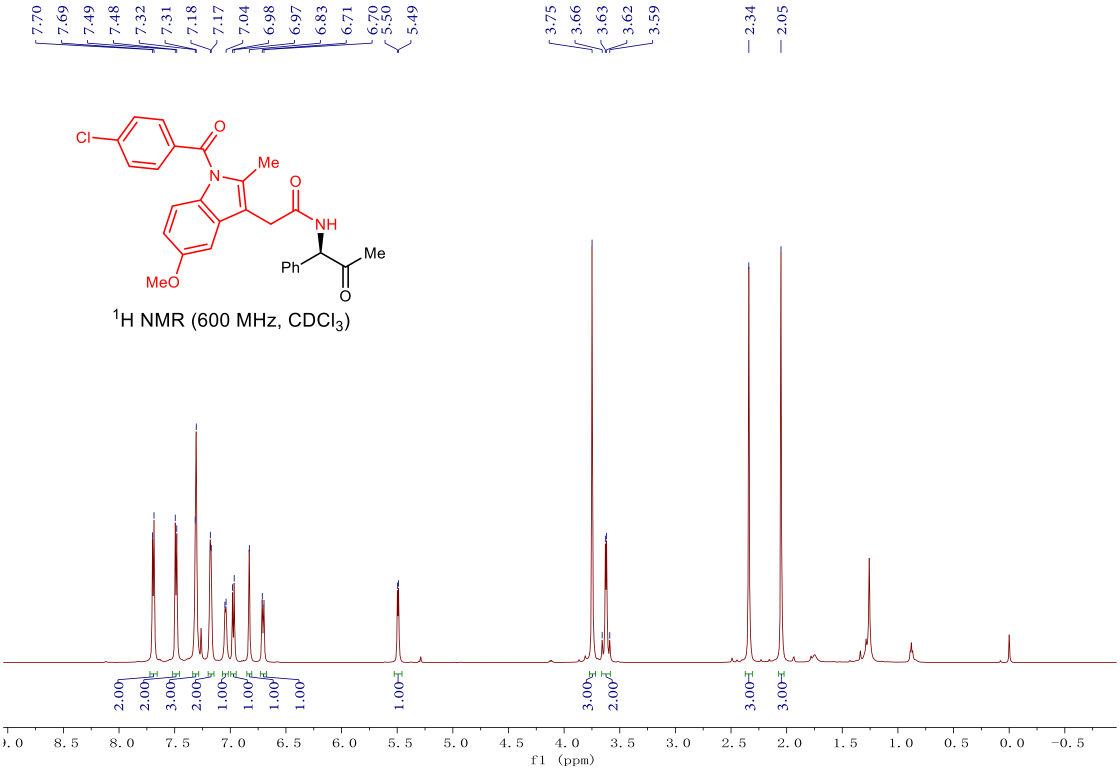

Supplementary Figure 124. ^{1}H NMR spectrum of compound 5a.

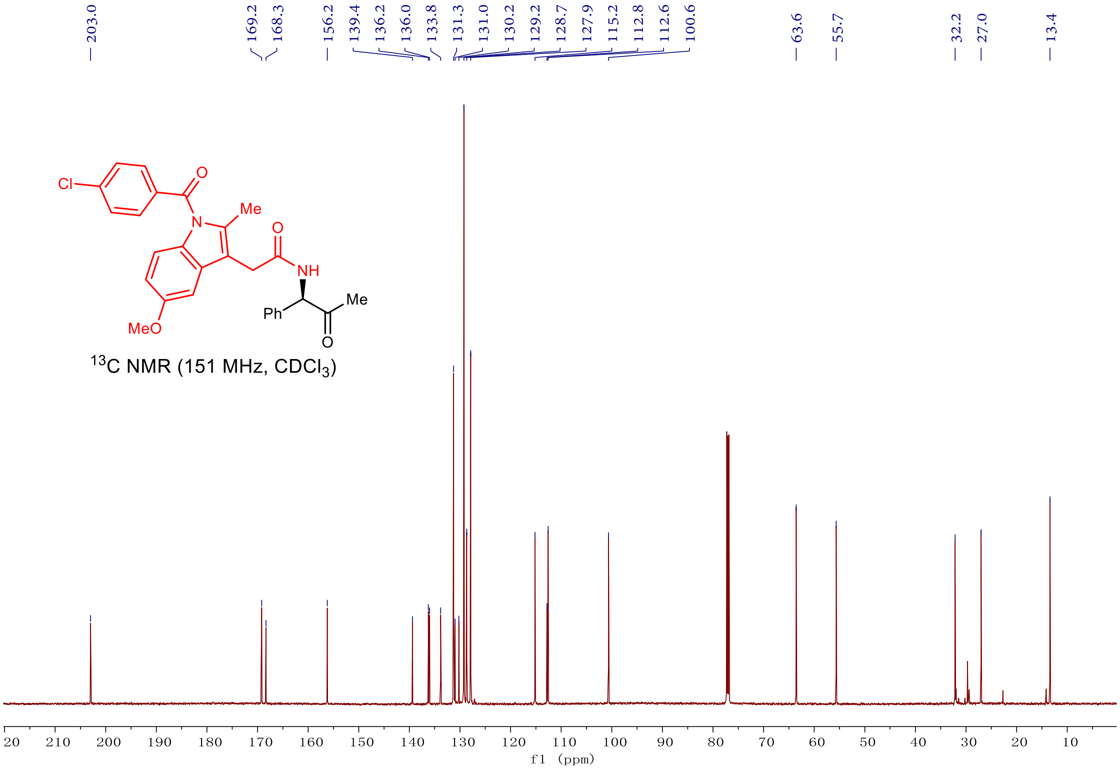

Supplementary Figure 125. ^{13}C NMR spectrum of compound 5a.

(*R*)-1-(2,6-difluorobenzyl)-N-(2-oxo-1-phenylpropyl)-1H-1,2,3-triazole-4-carboxamide (5b)

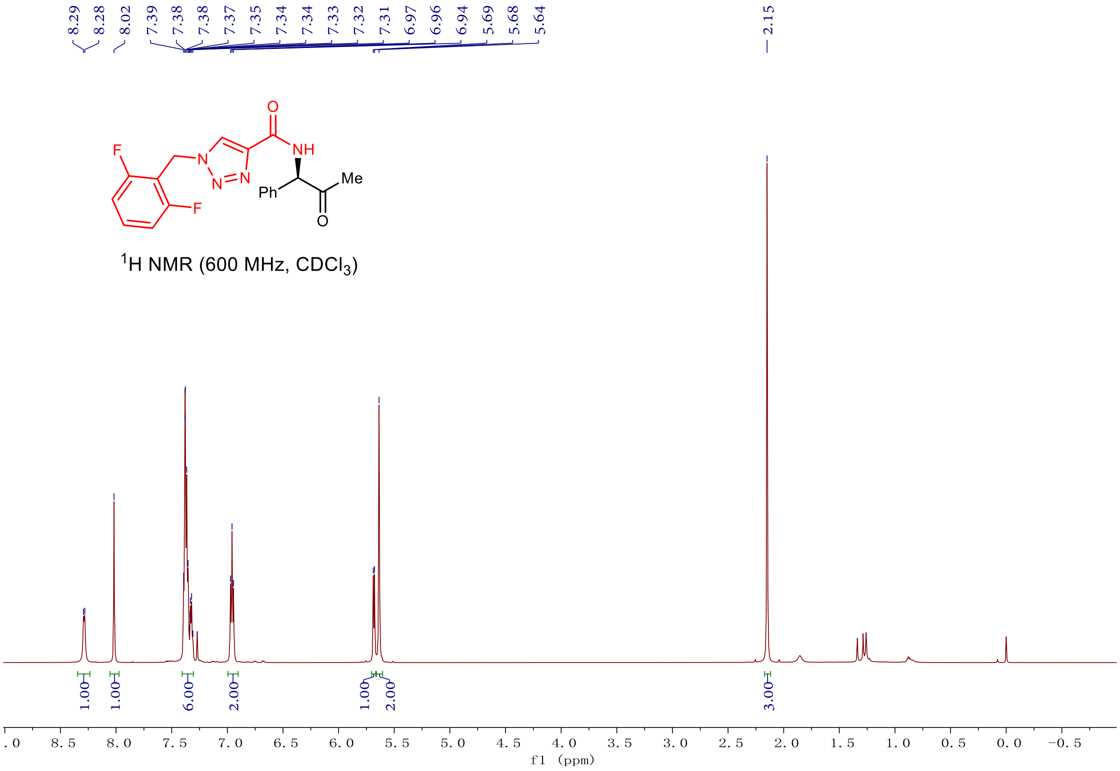

Supplementary Figure 126. ^{1}H NMR spectrum of compound 5b.

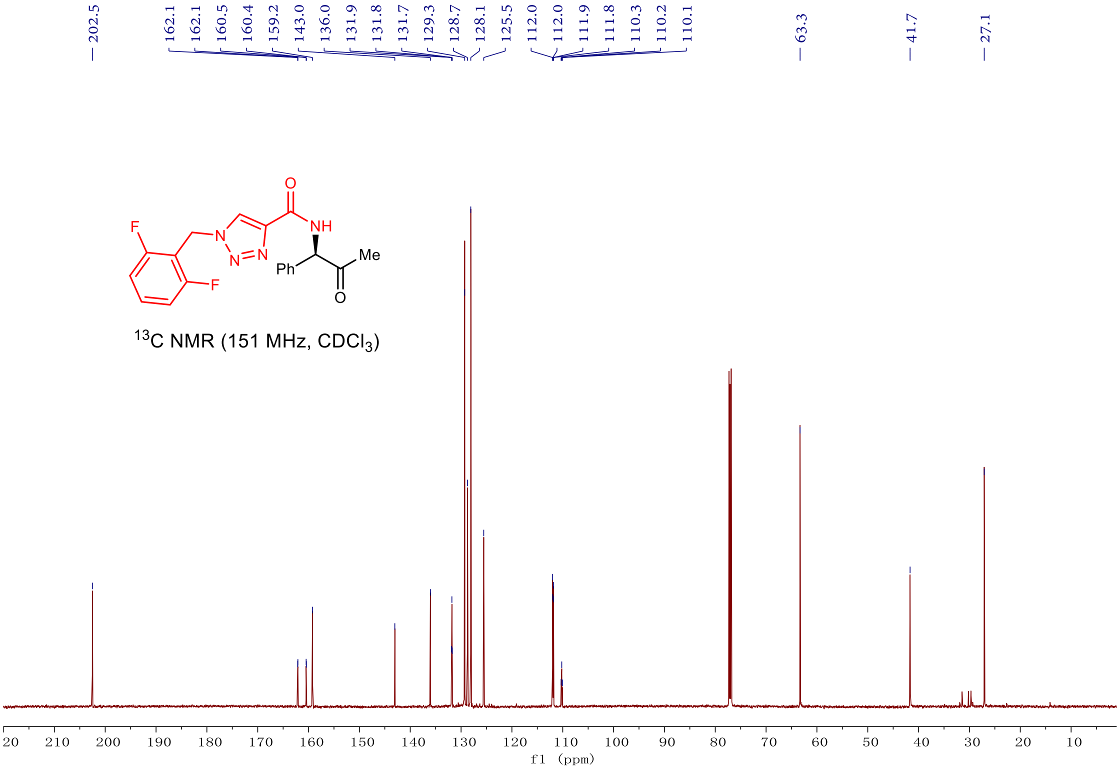

Supplementary Figure 127. ^{13}C NMR spectrum of compound 5b.

(*R*)-3-(4,5-diphenyloxazol-2-yl)-N-(2-oxo-1-phenylpropyl)propanamide (5c)

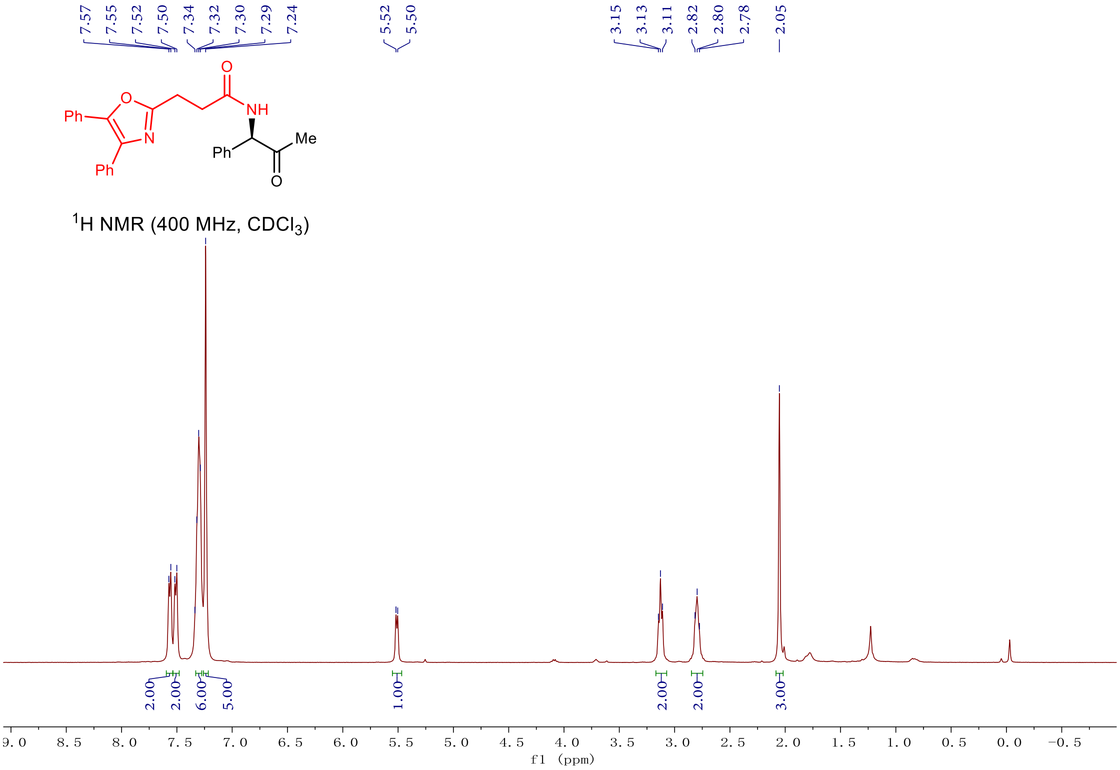

Supplementary Figure 128. ^{1}H NMR spectrum of compound 5c.

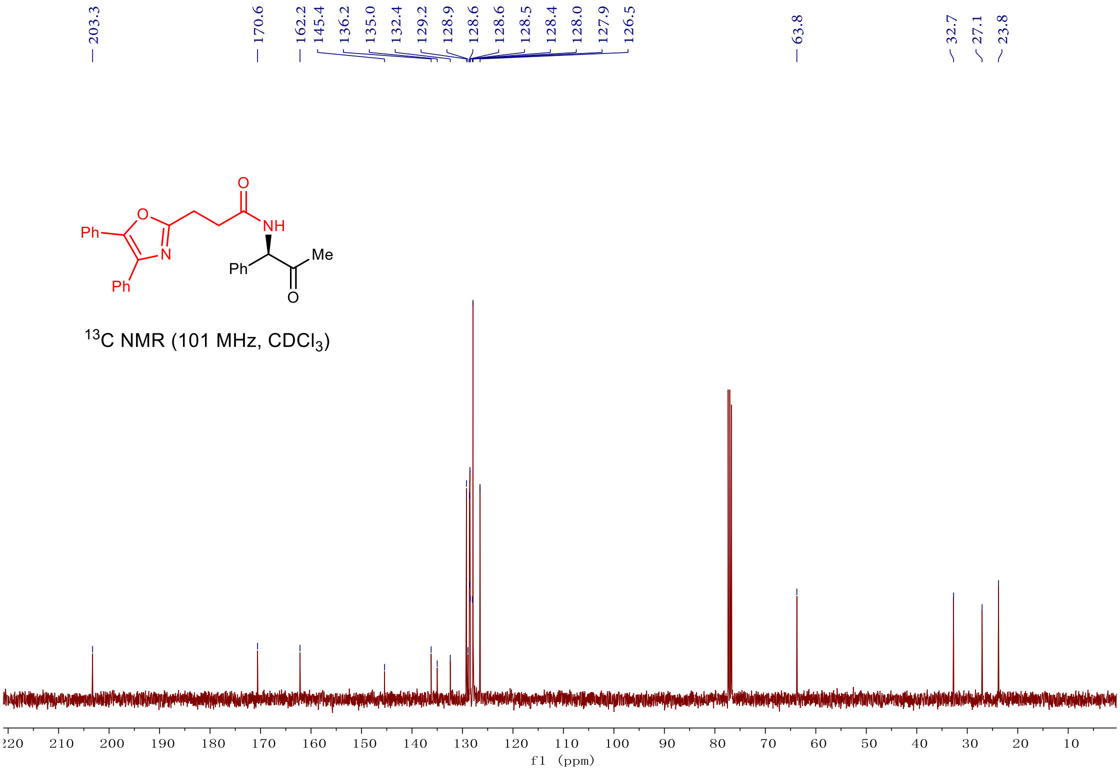

Supplementary Figure 129. ^{13}C NMR spectrum of compound 5c.

(*S*)-2-(6-methoxynaphthalen-2-yl)-N-((*R*)-2-oxo-1-phenylpropyl)propanamide (5d)

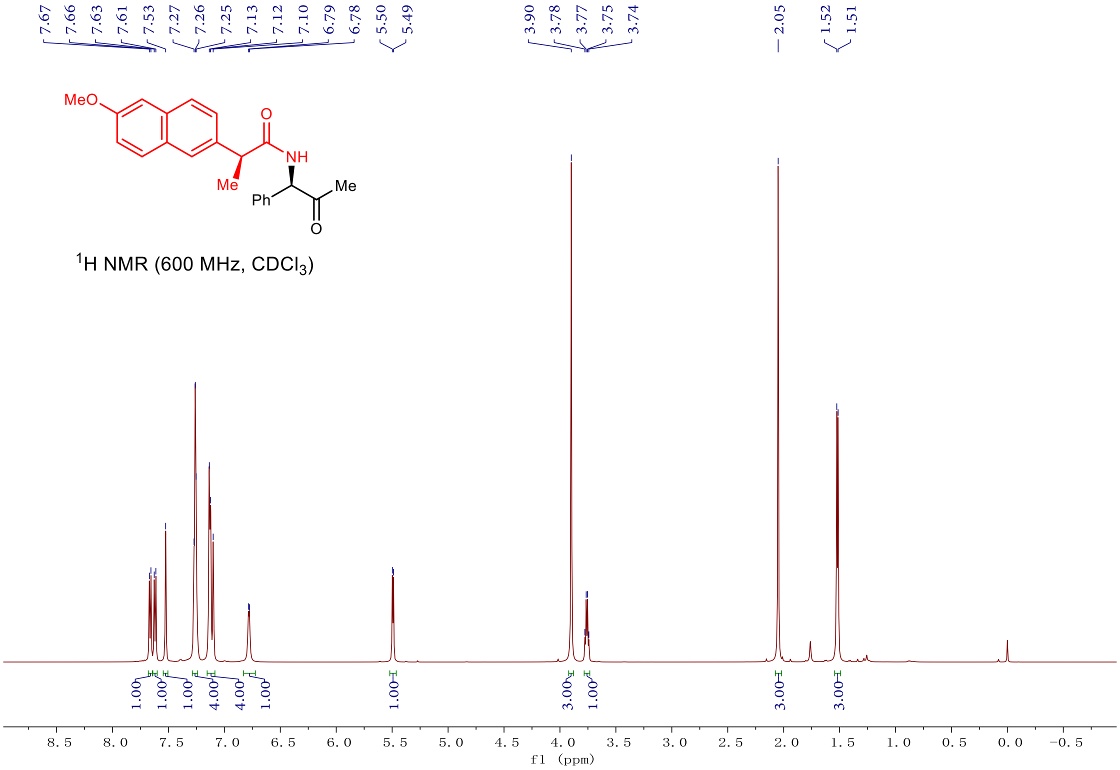

Supplementary Figure 130. ^{1}H NMR spectrum of compound 5d.

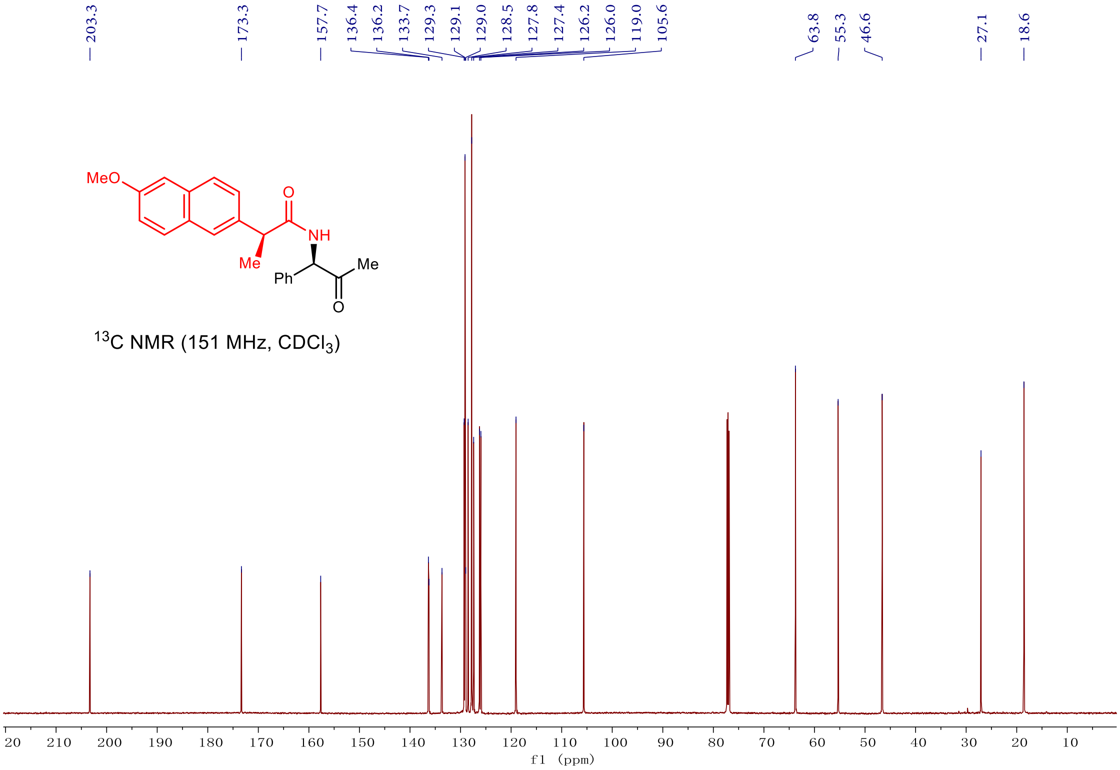

Supplementary Figure 131. ^{13}C NMR spectrum of compound 5d.

(*R*)-N-((*R*)-2-oxo-1-phenylpropyl)-2-(2-oxopyrrolidin-1-yl)butanamide (5e)

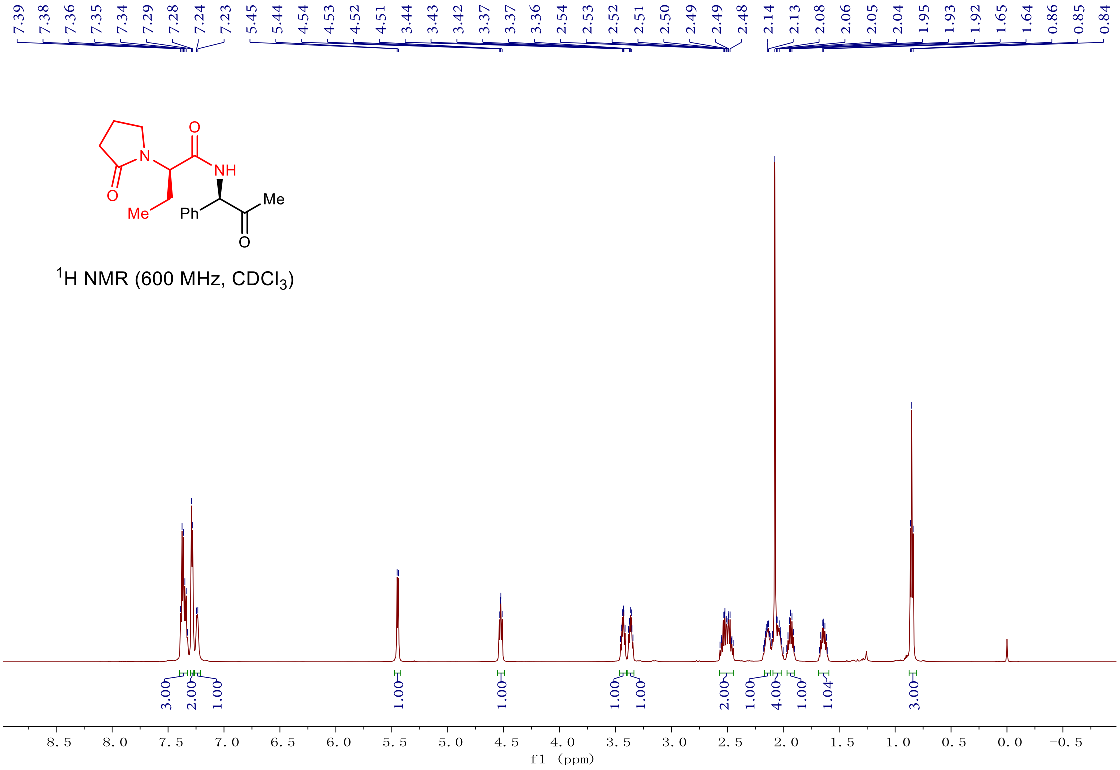

Supplementary Figure 132. ^{1}H NMR spectrum of compound 5e.

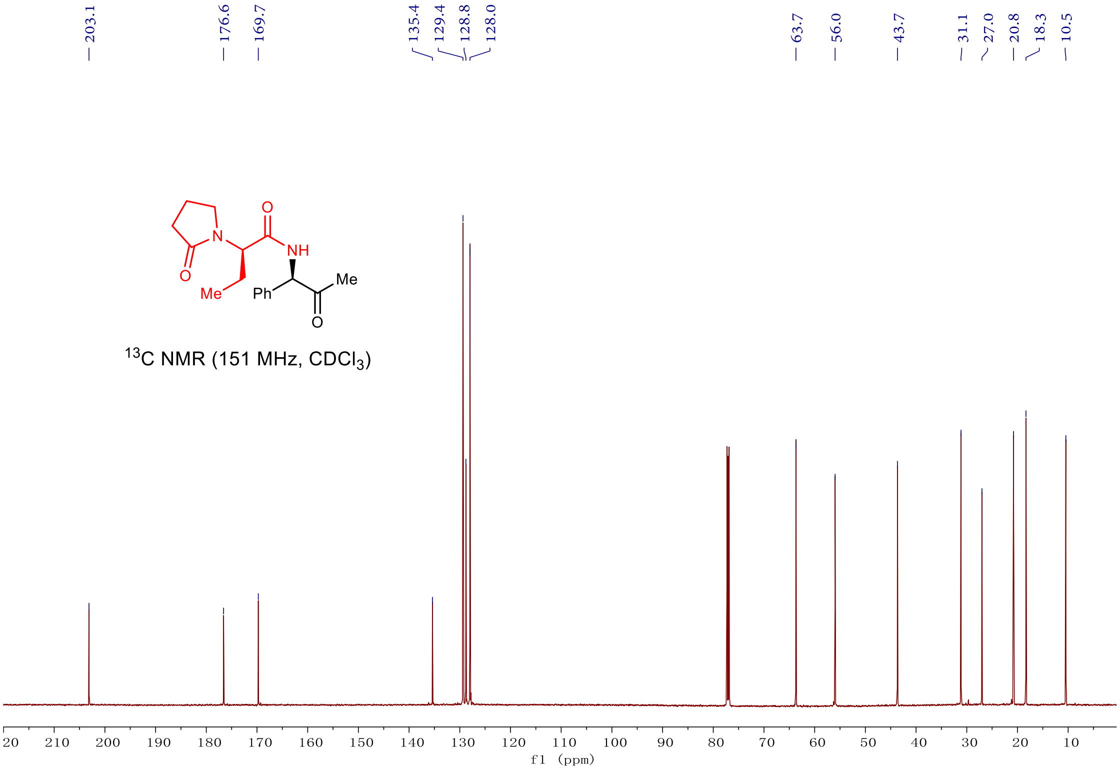

Supplementary Figure 133. ^{13}C NMR spectrum of compound 5e.

(2*S*,5*R*,6*R*)-3,3-dimethyl-7-oxo-N-((*R*)-2-oxo-1-phenylpropyl)-6-(2-phenylacetamido)-4-thia-1-azabicyclo[3.2.0]heptane-2-carboxamide (5f)

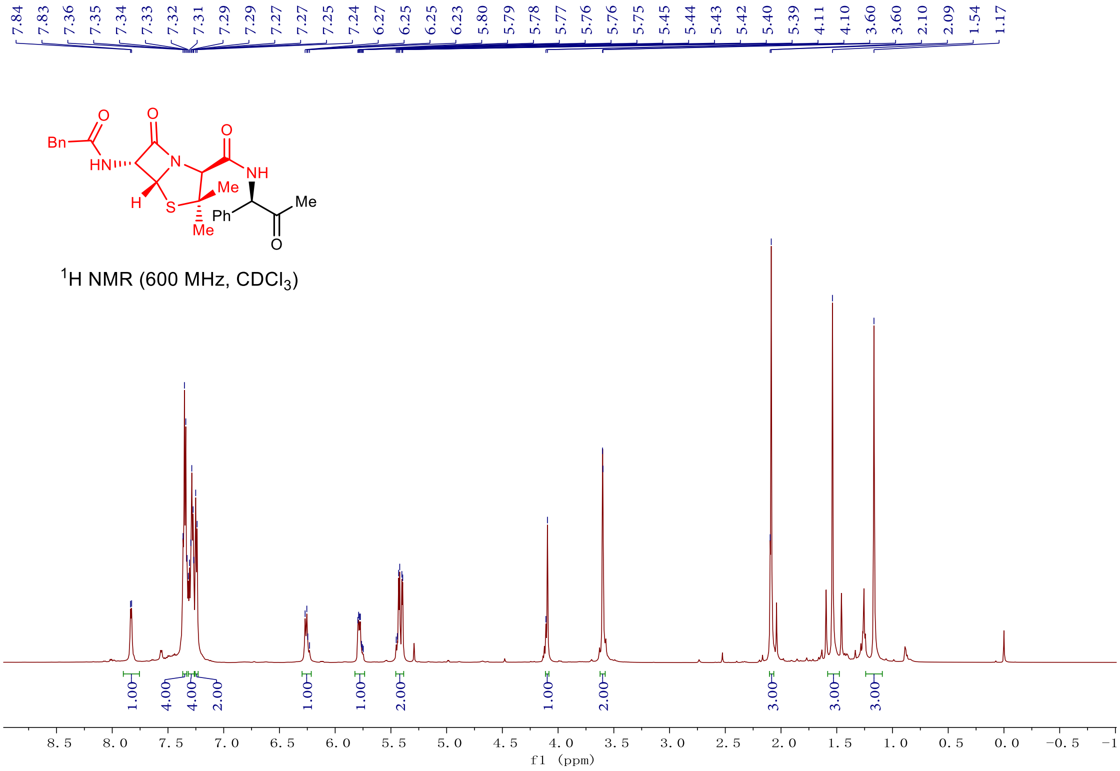

Supplementary Figure 134. ^{1}H NMR spectrum of compound 5f.

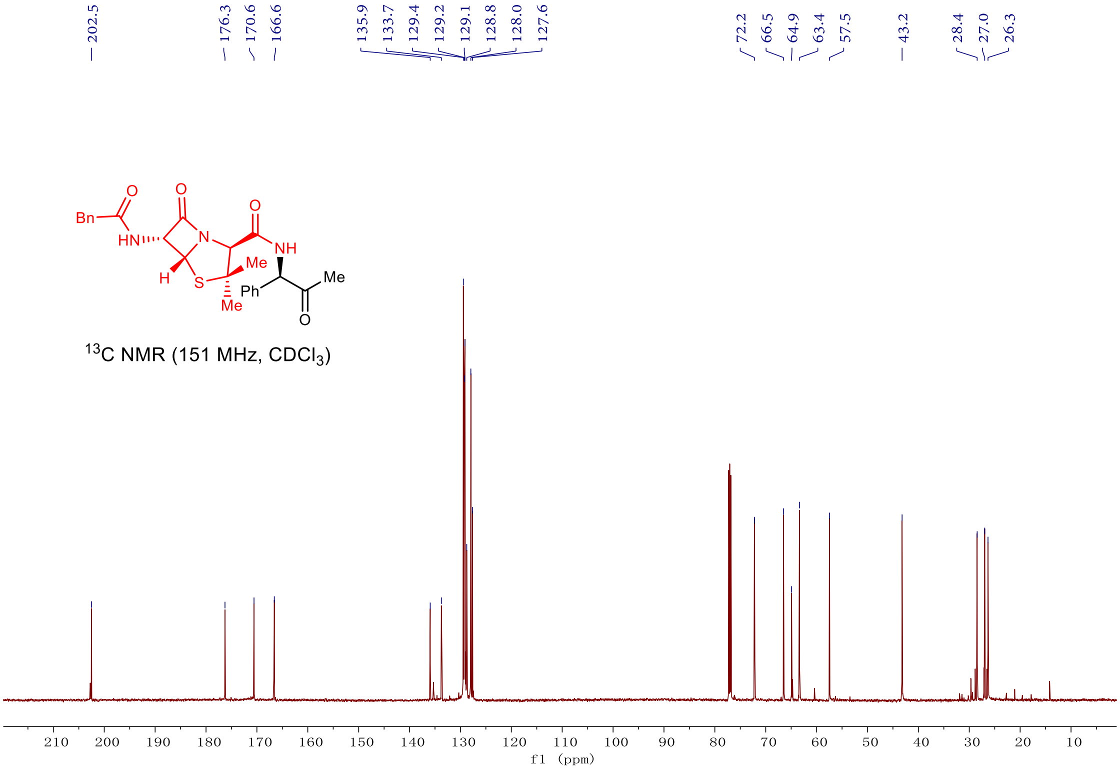

Supplementary Figure 135. ^{13}C NMR spectrum of compound 5f.

Methyl N-(*tert*-butoxycarbonyl)-N-((*R*)-2-oxo-1-phenylpropyl)-L-glutaminate (5g)

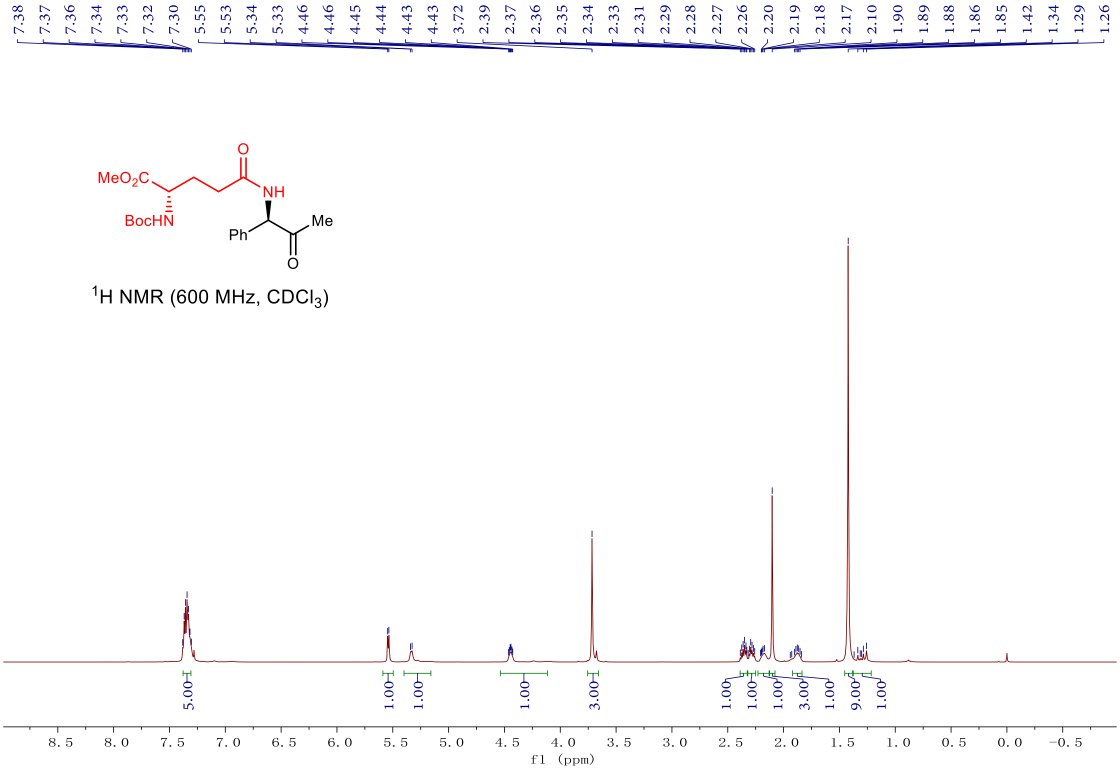

Supplementary Figure 136. ^{1}H NMR spectrum of compound 5g.

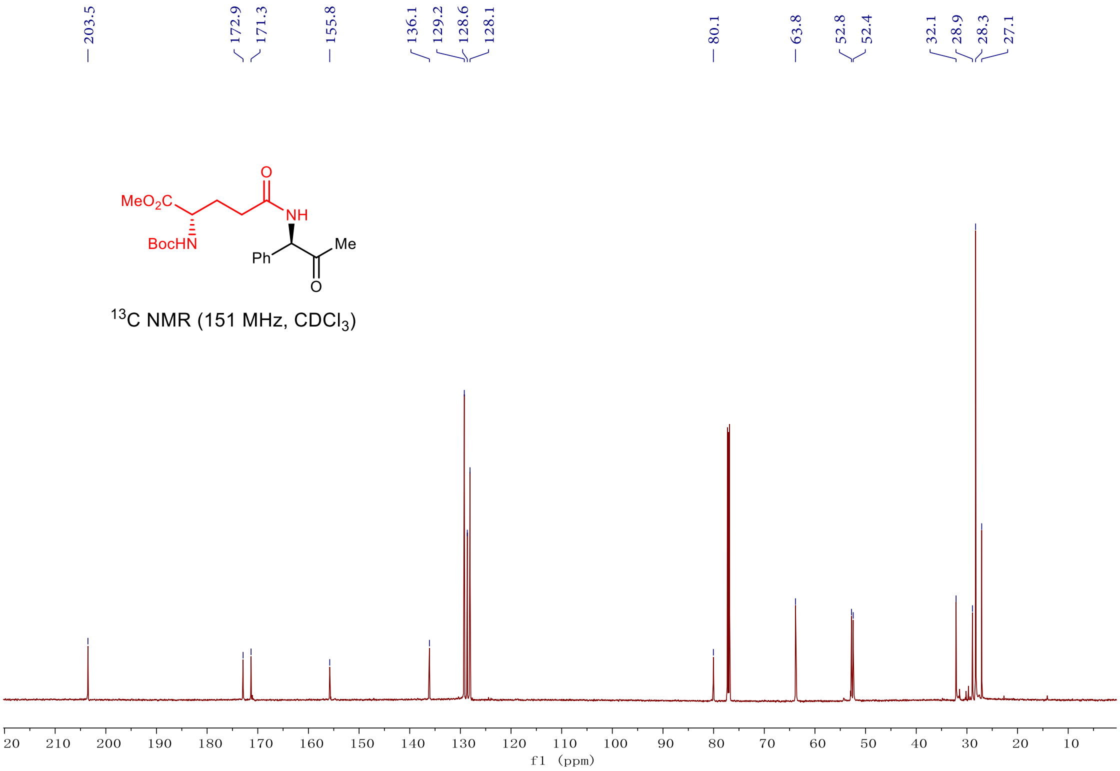

Supplementary Figure 137. ^{13}C NMR spectrum of compound 5g.

Methyl N-(*tert*-butoxycarbonyl)-N-((*R*)-2-oxo-1-phenylpropyl)-L-asparaginate (5h)

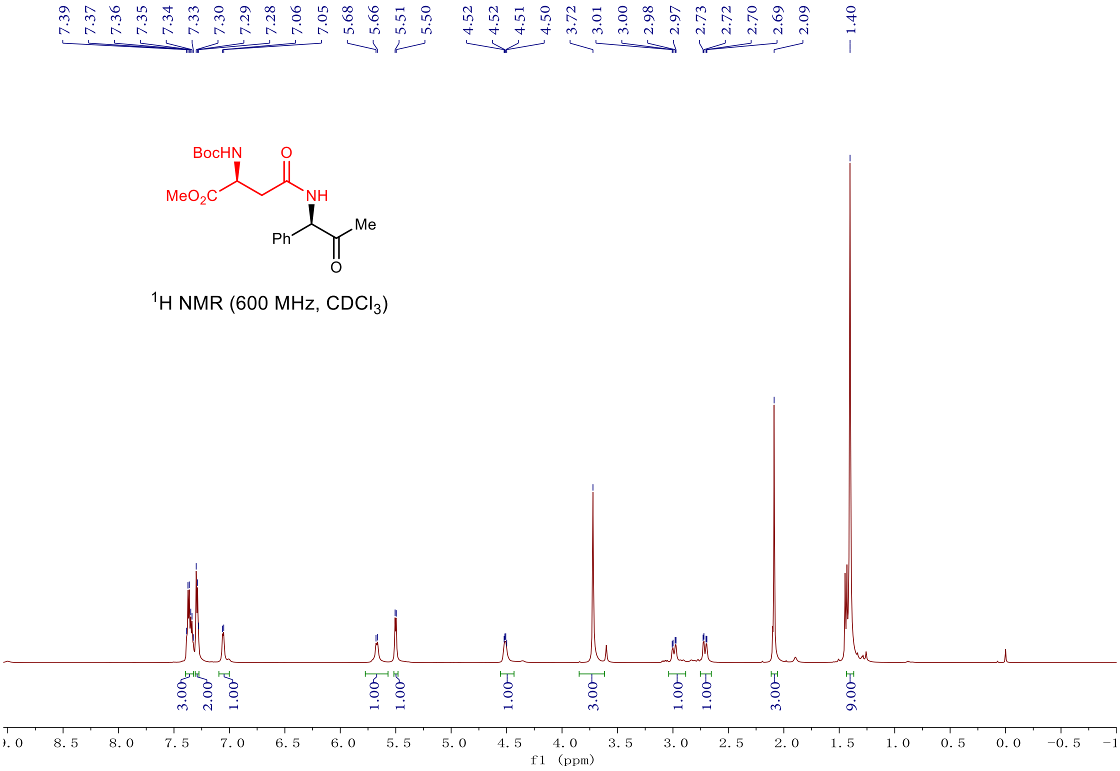

Supplementary Figure 138. ^{1}H NMR spectrum of compound 5h.

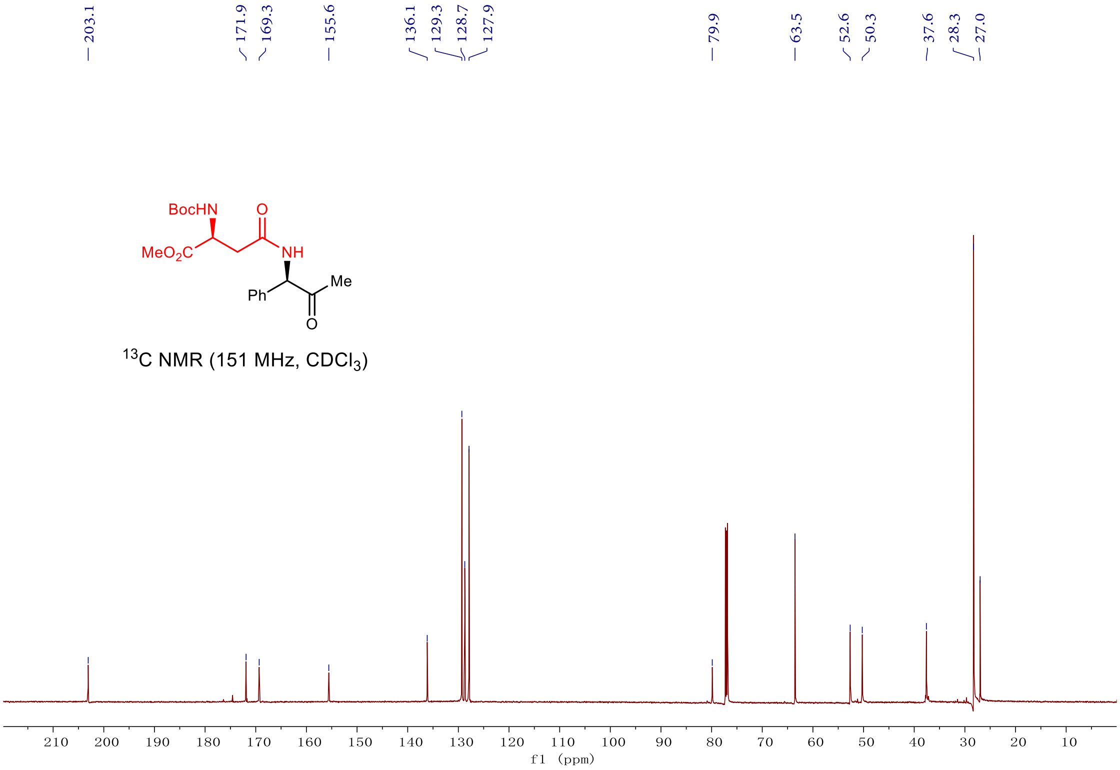

Supplementary Figure 139. ^{13}C NMR spectrum of compound 5h.

N-((1*R*, 2*R*)-2-hydroxy-1,2-diphenylpropyl)benzamide (7a)

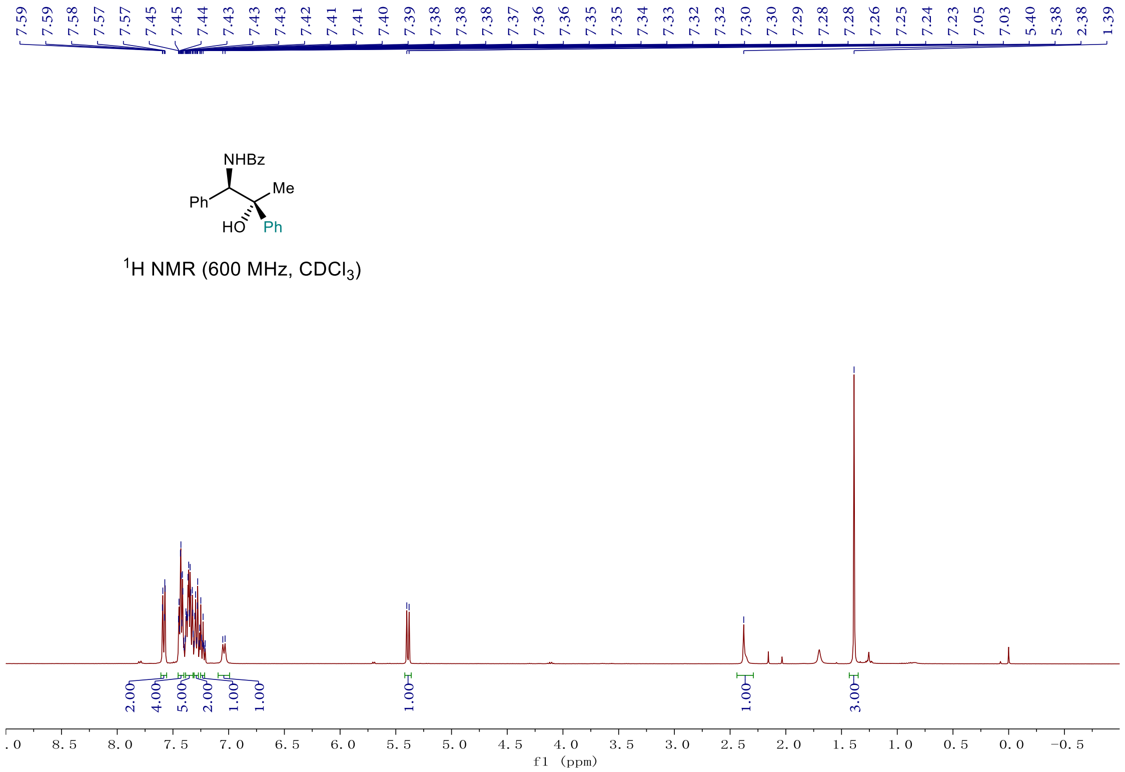

Supplementary Figure 140. ^{1}H NMR spectrum of compound 7a.

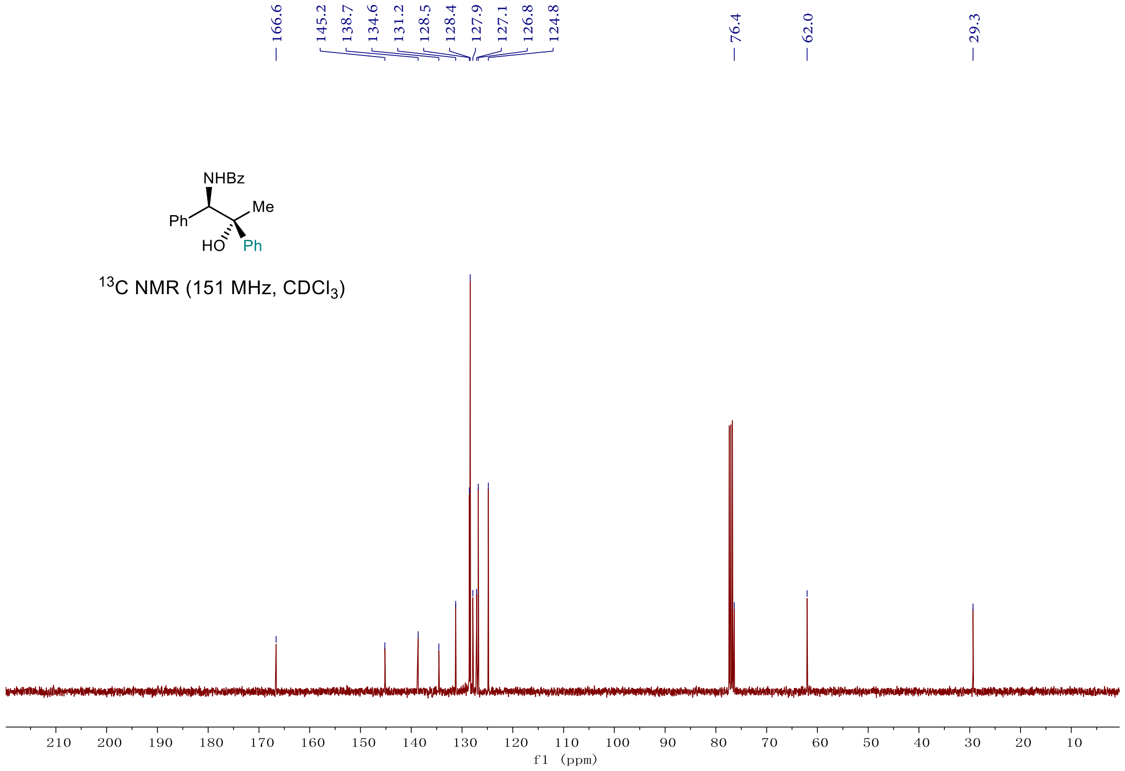

Supplementary Figure 141. ^{13}C NMR spectrum of compound 7a.

N-((1*R*, 2*R*)-12-hydroxy-1-phenylpropyl-2-naphthalenyl)benzamide (7b)

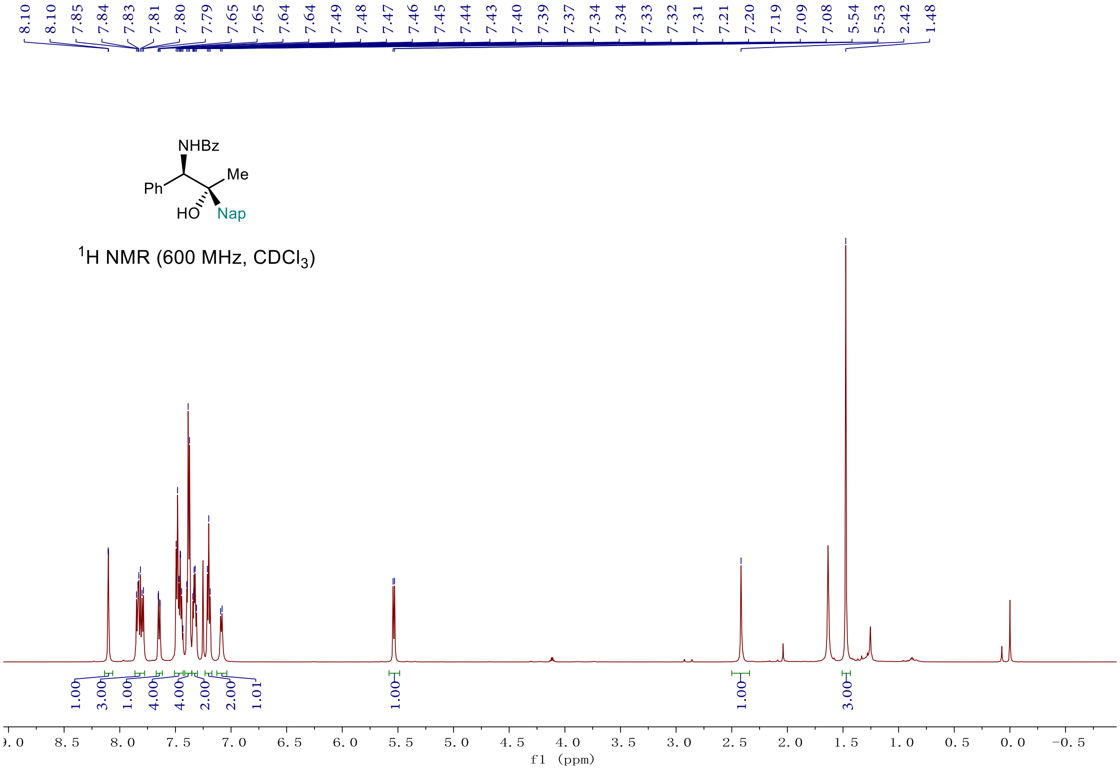

Supplementary Figure 142. ^{1}H NMR spectrum of compound 7b.

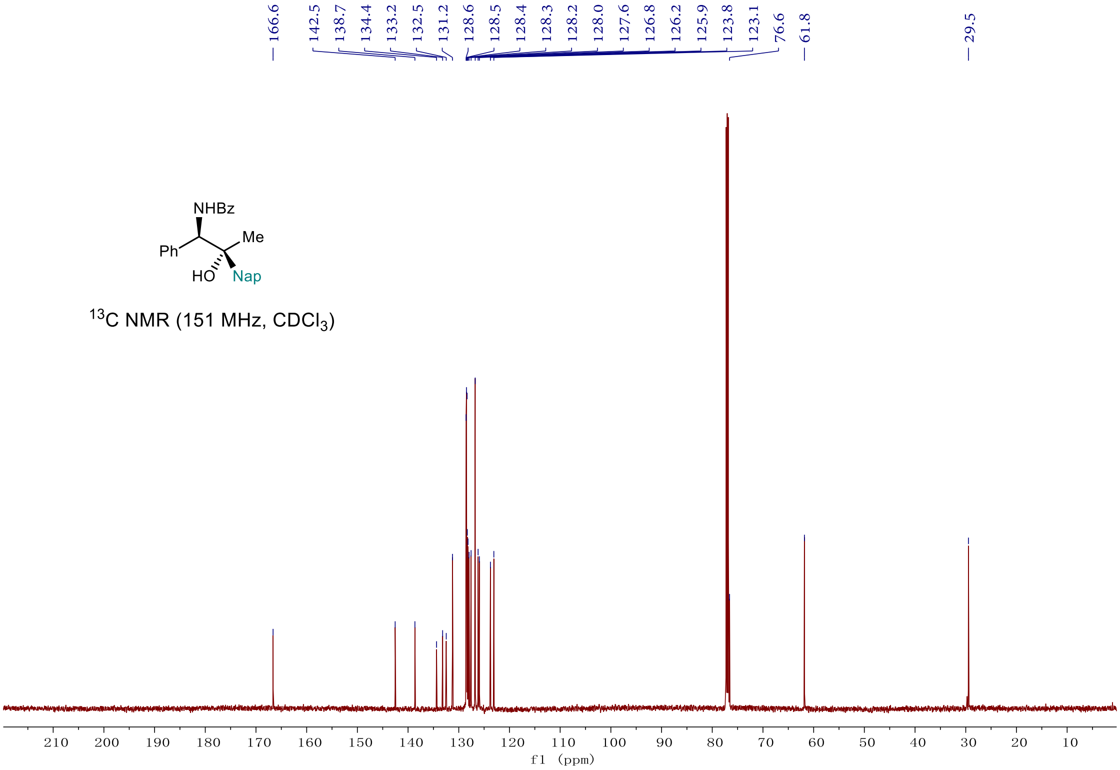

Supplementary Figure 143. ^{13}C NMR spectrum of compound 7b.

N-((1*R*, 2*R*)-12-hydroxy-1-phenylpropyl-2-vinyl)benzamide (7c)

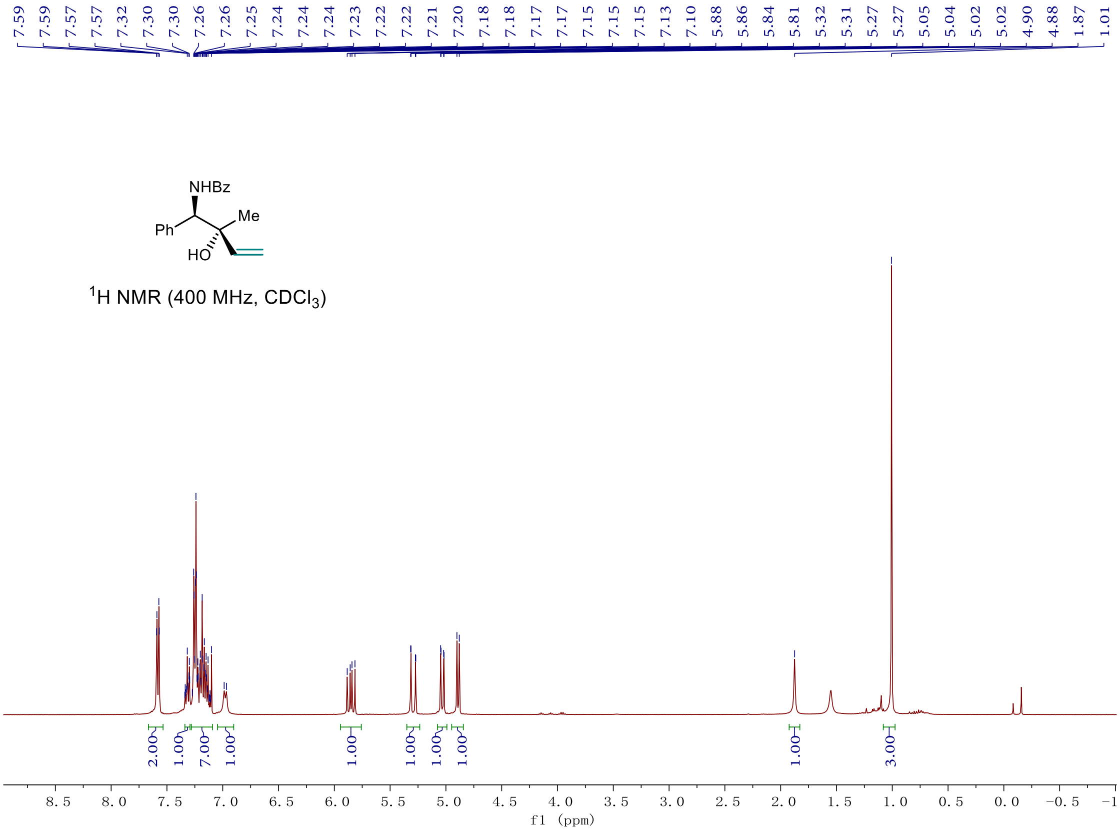

Supplementary Figure 144. ^{1}H NMR spectrum of compound 7c.

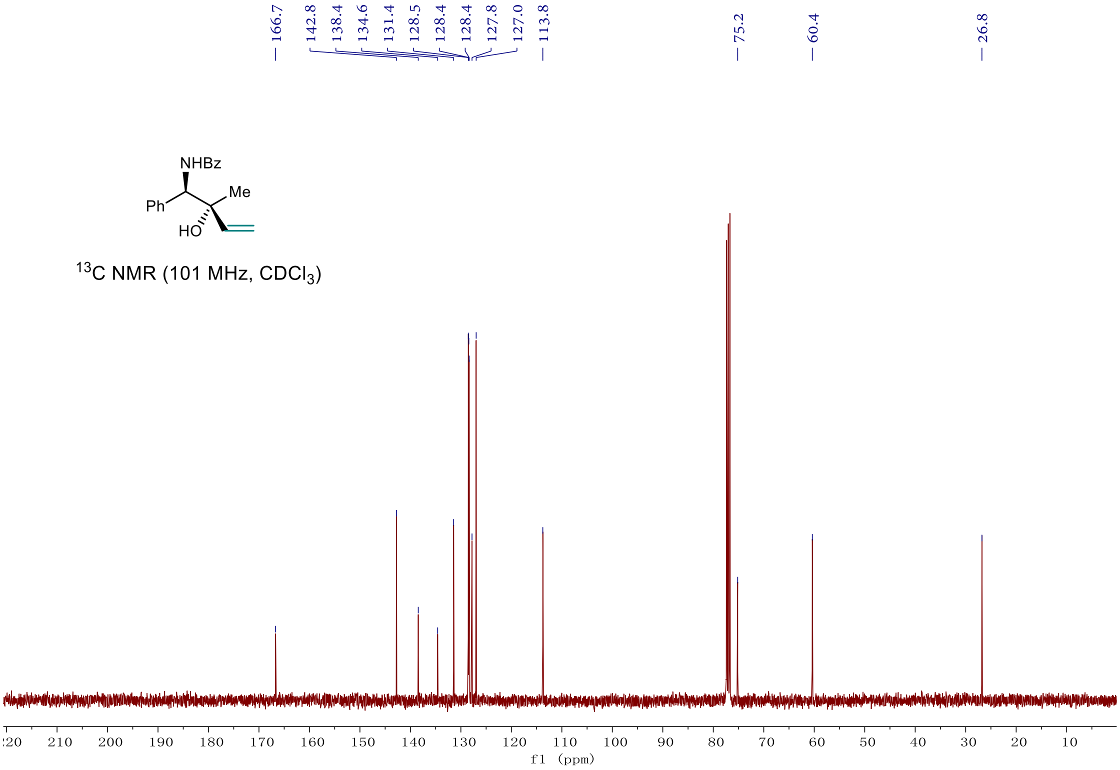

Supplementary Figure 145. ^{13}C NMR spectrum of compound 7c.

N-((1*R*,2*R*)-2-hydroxy-2-methyl-1-phenylbutyl)benzamide (7d)

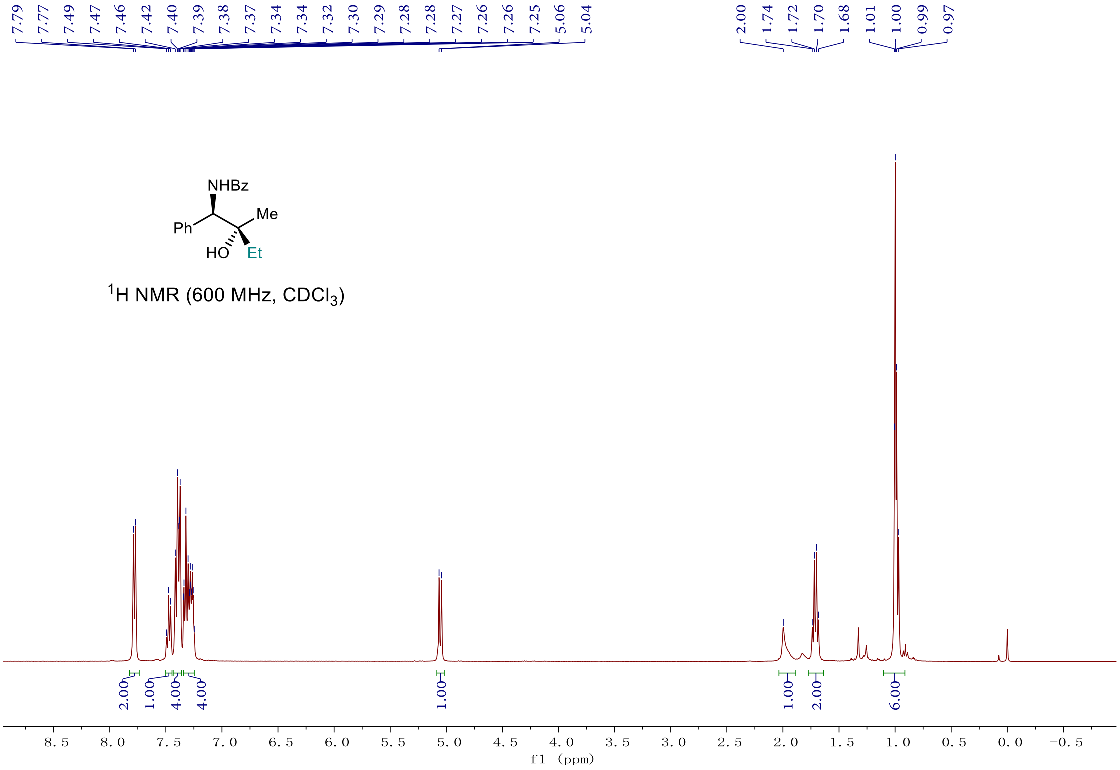

Supplementary Figure 146. ^{1}H NMR spectrum of compound 7d.

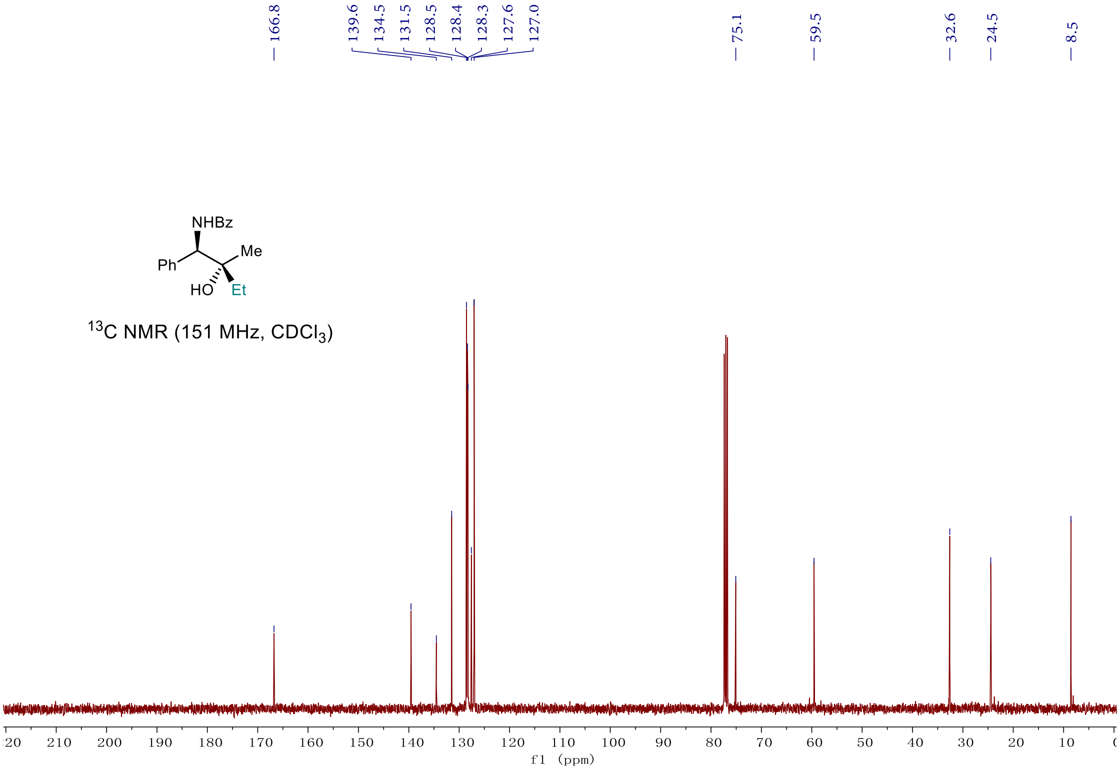

Supplementary Figure 147. ^{13}C NMR spectrum of compound 7d.

N-((1*R*,2*R*)-2-hydroxy-2-methyl-1-phenyldecyl)benzamide (7e)

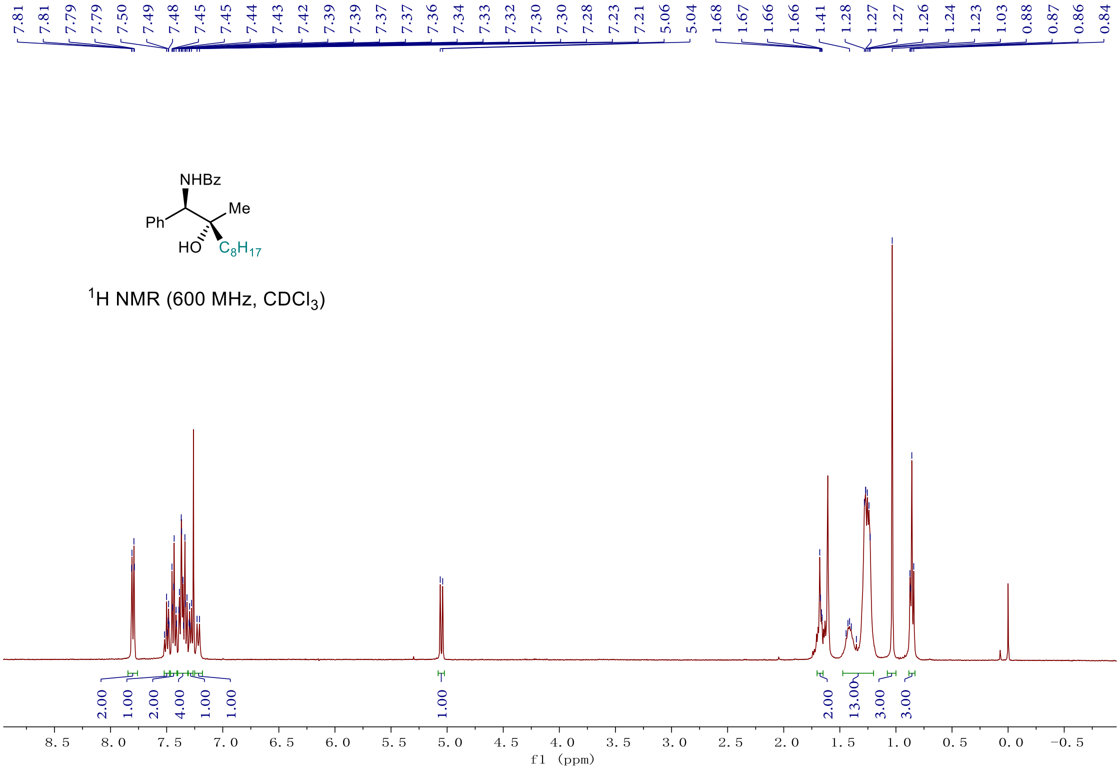

Supplementary Figure 148. ^{1}H NMR spectrum of compound 7e.

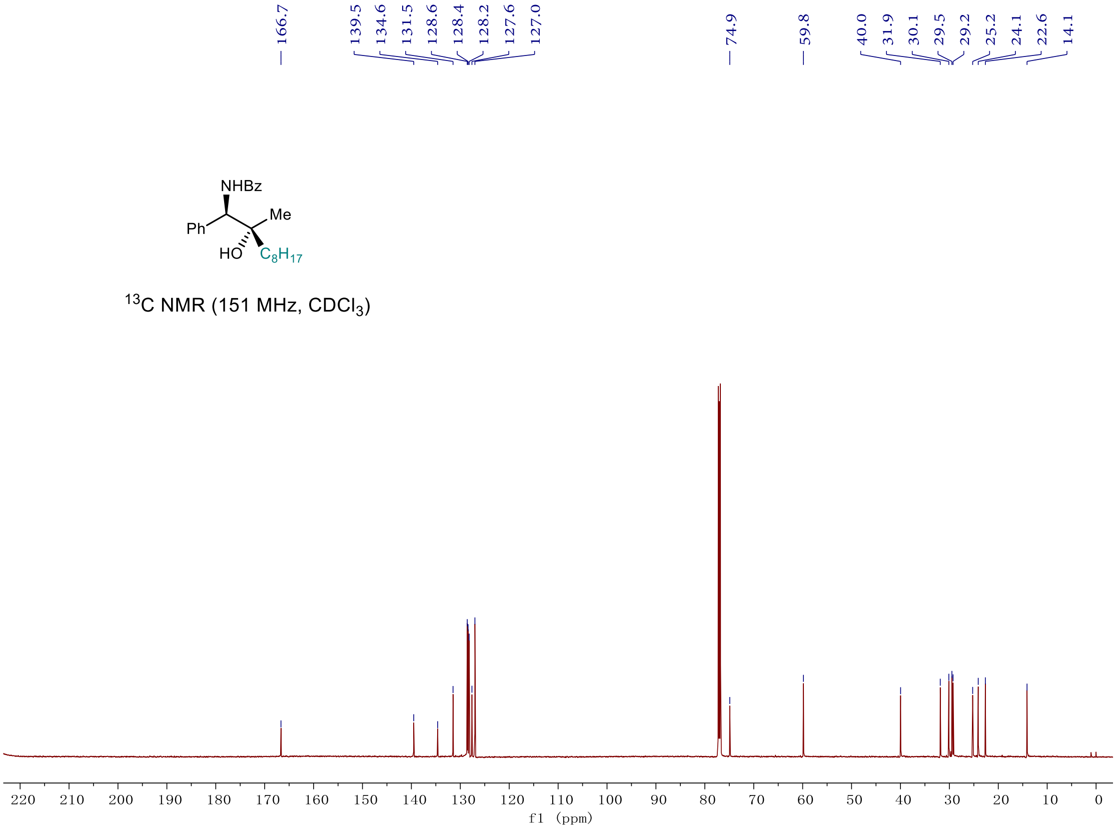

Supplementary Figure 149. ^{13}C NMR spectrum of compound 7e.

N-((1*R*,2*R*)-2-hydroxy-2-methyl-1,3-diphenylpropyl)benzamide (7f)

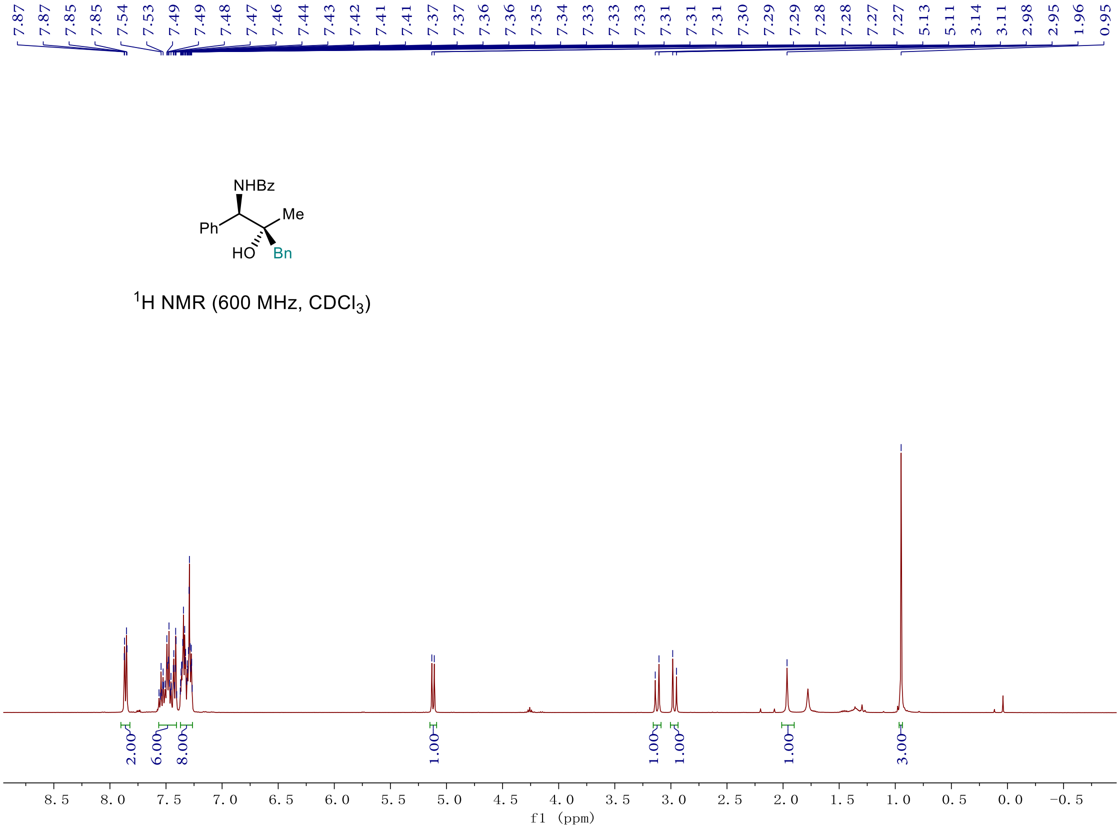

Supplementary Figure 150. ^{1}H NMR spectrum of compound 7f.

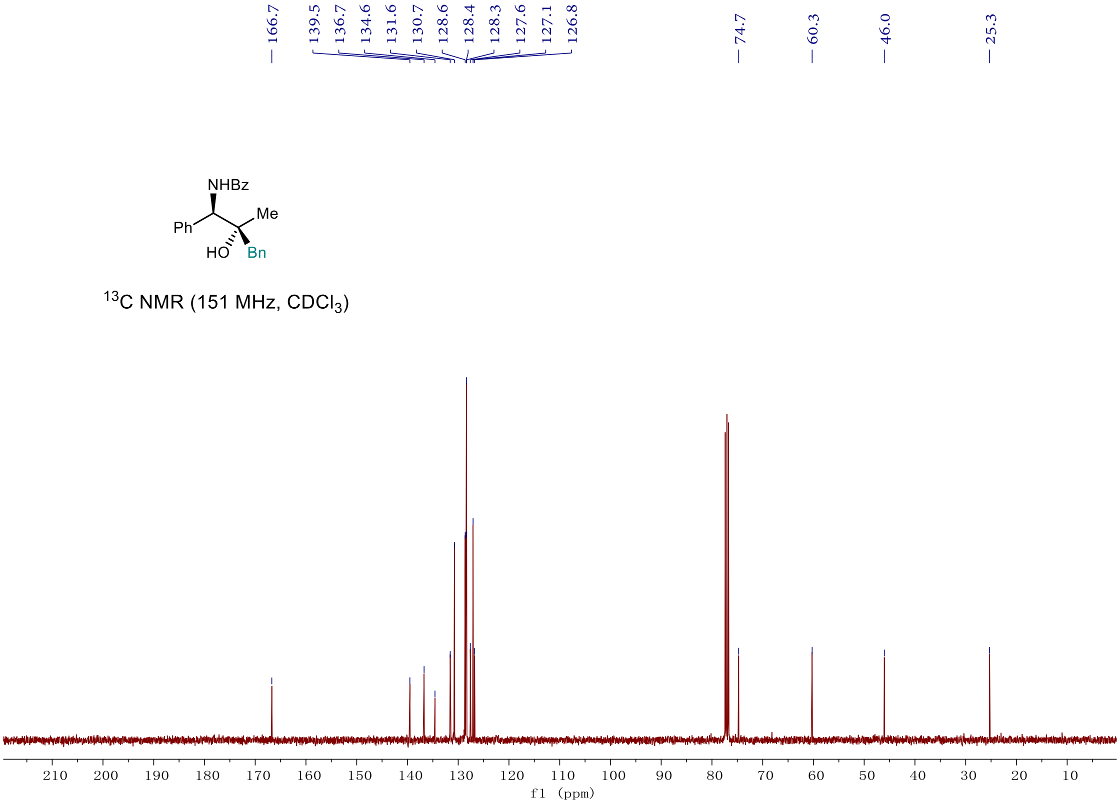

Supplementary Figure 151. ^{13}C NMR spectrum of compound 7f.

N-((1*R*,2*R*)-2-cyclopropyl-2-hydroxy-1-phenylpropyl)benzamide (7g)

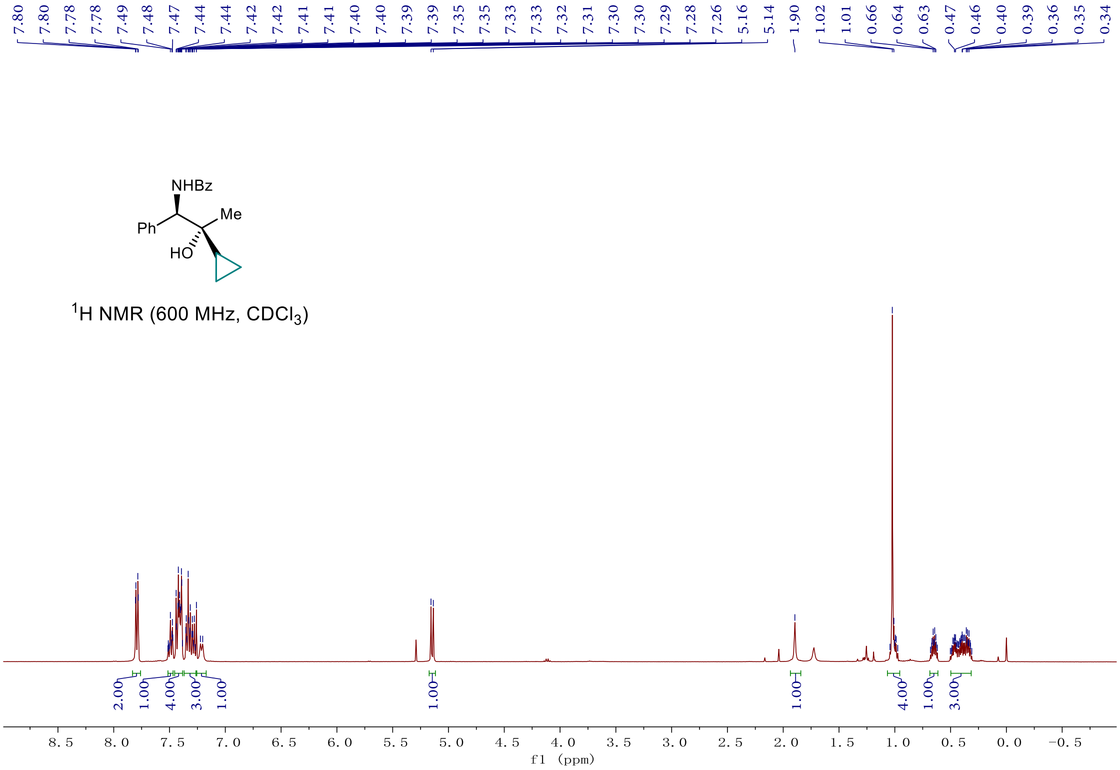

Supplementary Figure 152. ^{1}H NMR spectrum of compound 7g.

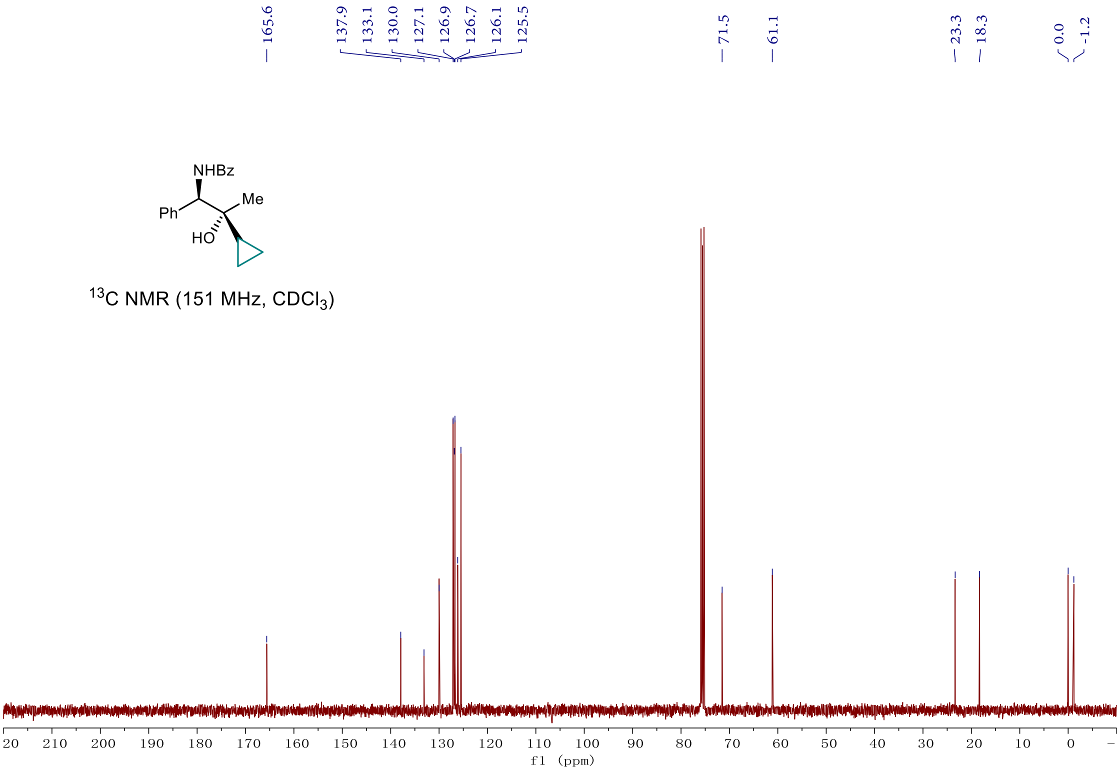

Supplementary Figure 153. ^{13}C NMR spectrum of compound 7g.

N-((1*R*,2*R*)-2-cyclohexyl-2-hydroxy-1-phenylpropyl)benzamide (7h)

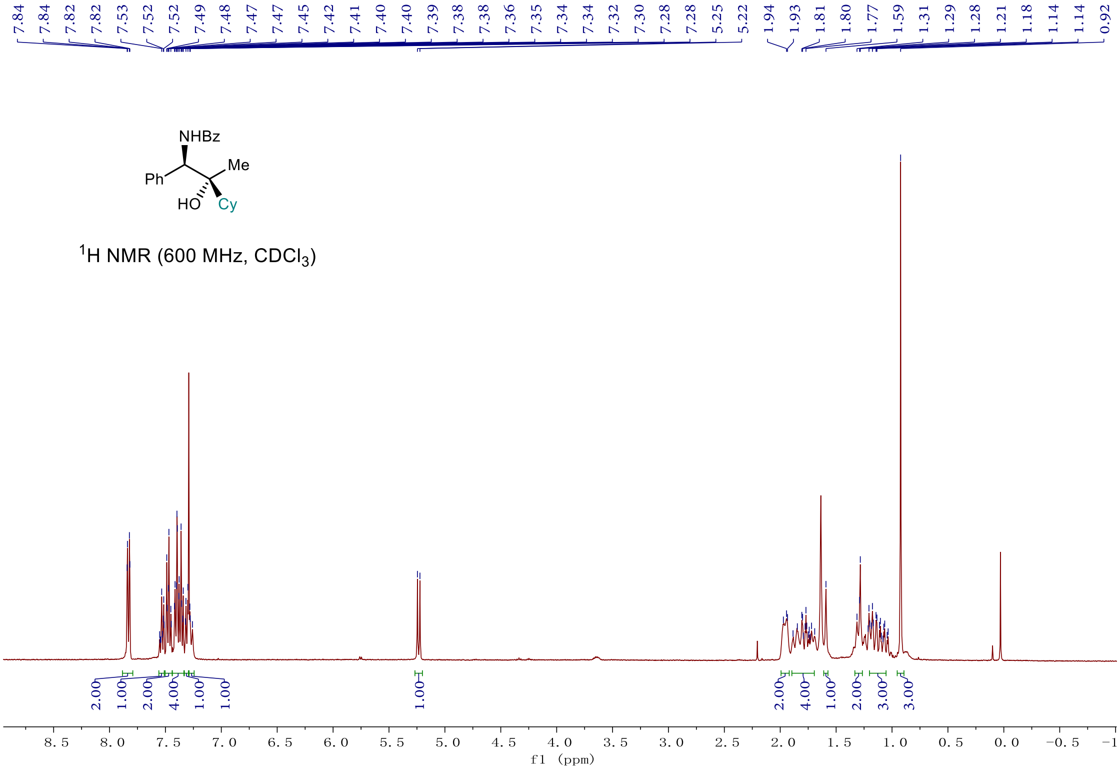

Supplementary Figure 154. ^{1}H NMR spectrum of compound 7h.

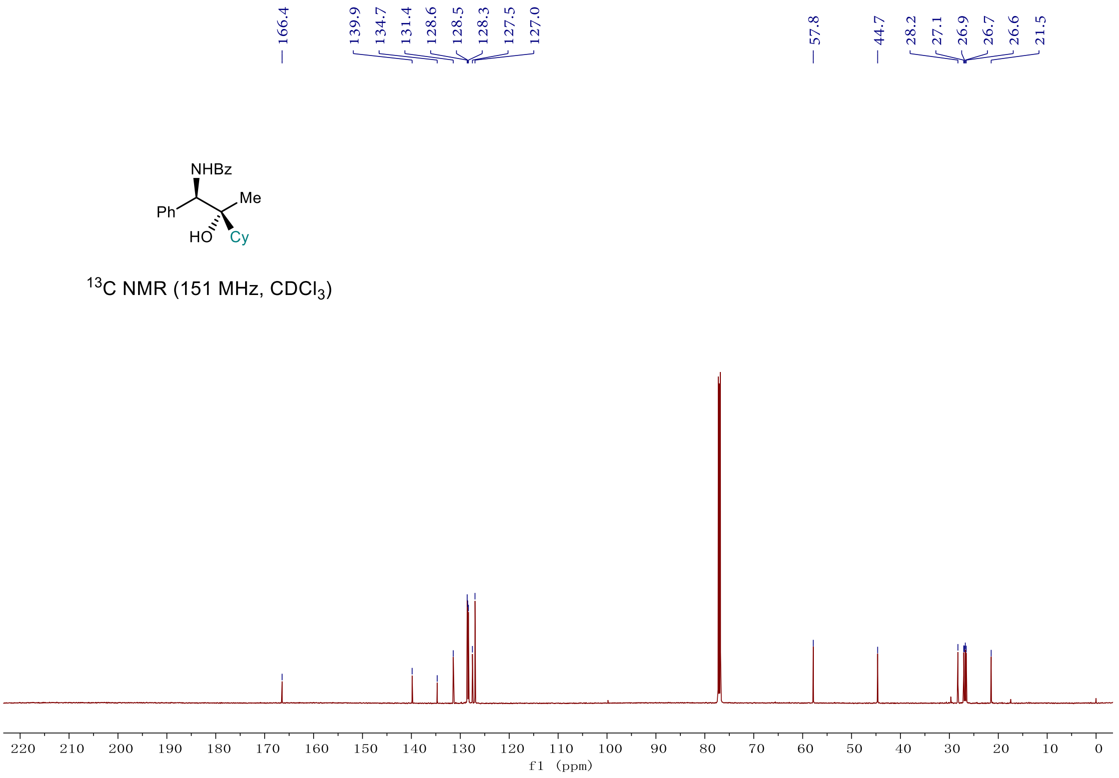

Supplementary Figure 155. ^{13}C NMR spectrum of compound 7h.

(*S*)-N-(2-methyl-1-phenylallyl)benzamide (8)

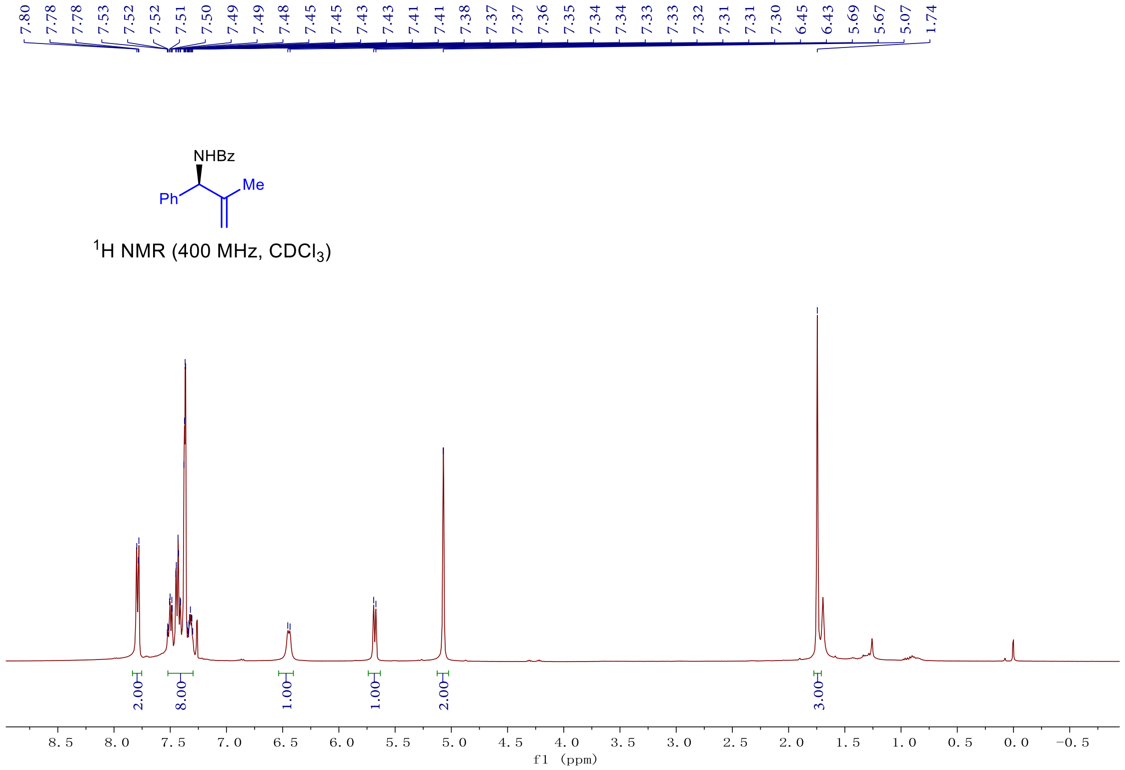

Supplementary Figure 156. ^{1}H NMR spectrum of compound 8.

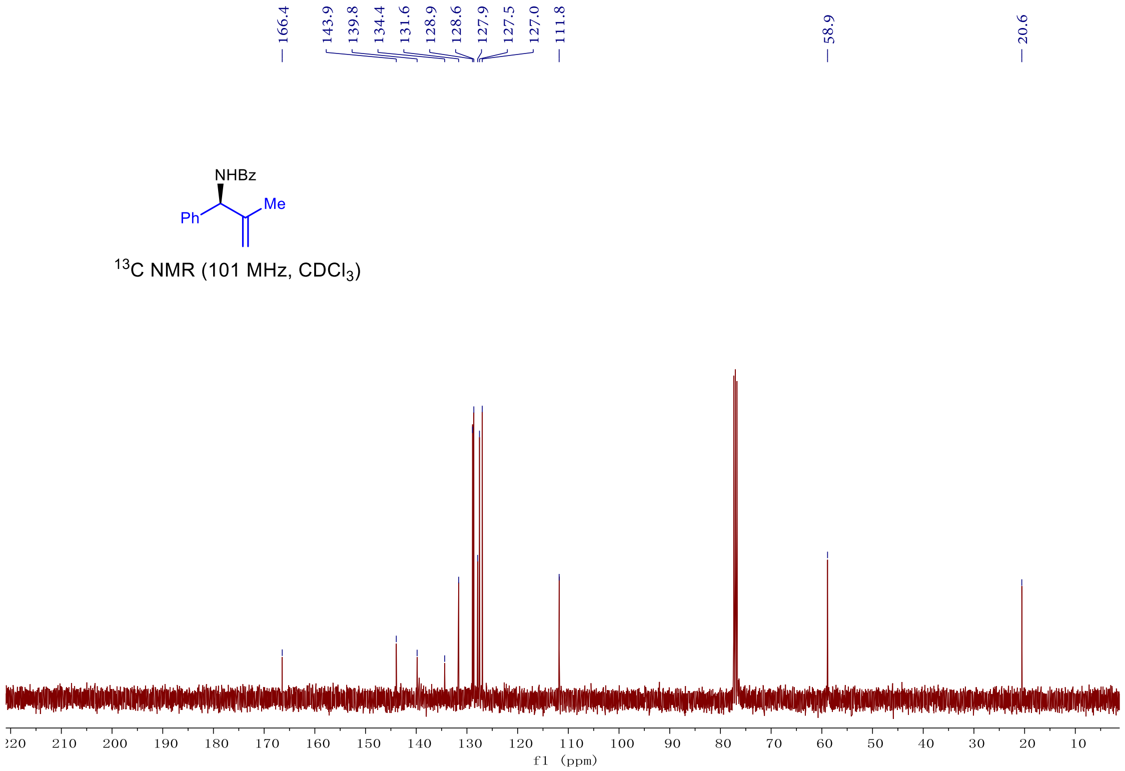

Supplementary Figure 157. ^{13}C NMR spectrum of compound 8.

N-((1*R*,2*S*)-2-hydroxy-1-phenylpropyl)benzamide (9)

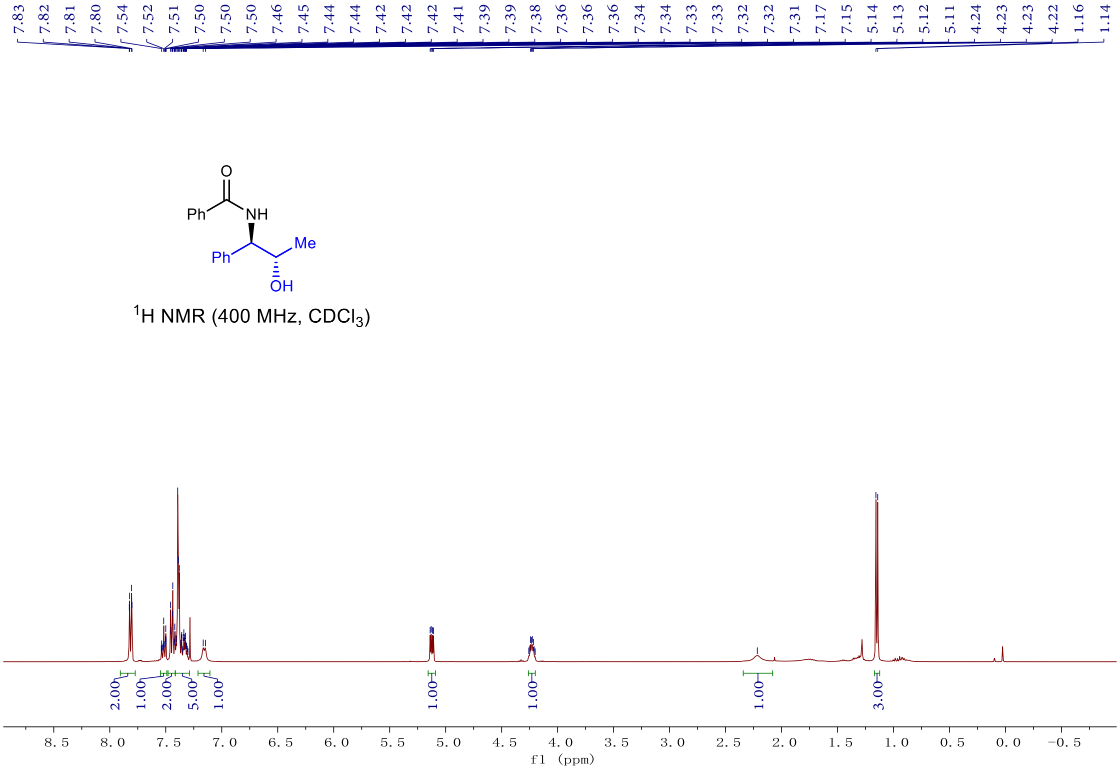

Supplementary Figure 158. ^{1}H NMR spectrum of compound 9.

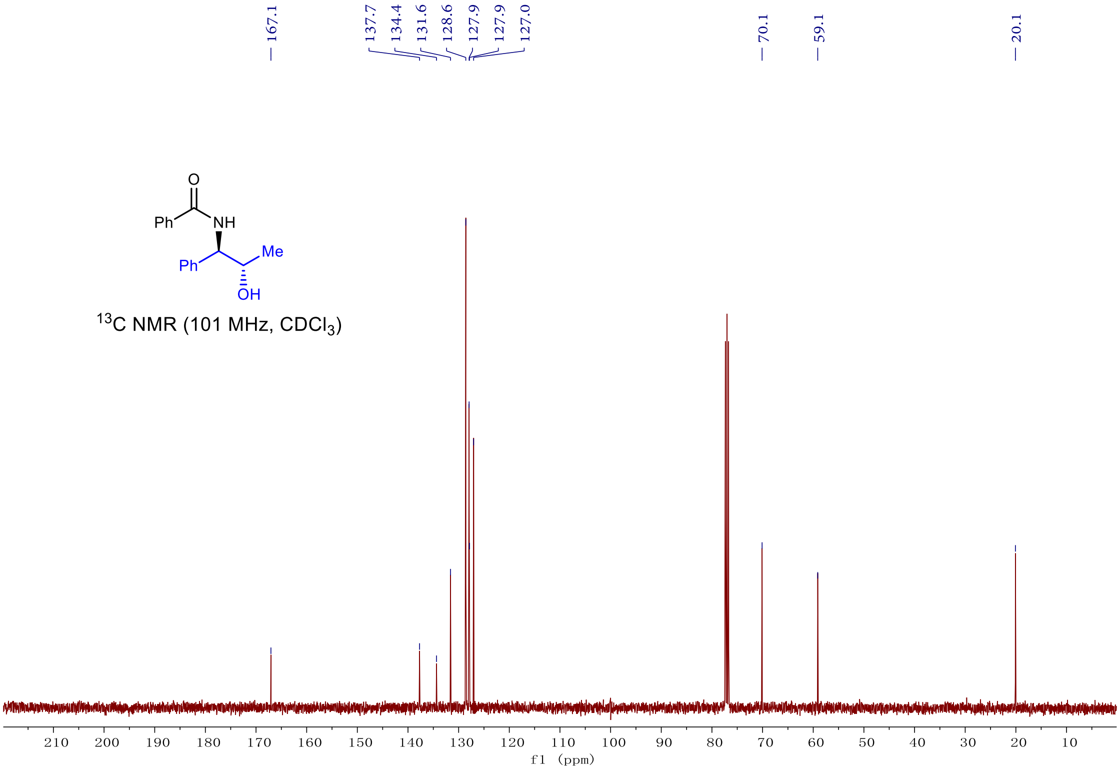

Supplementary Figure 159. ^{13}C NMR spectrum of compound 9.

(4*R*,5*R*)-5-methyl-2,4-diphenyl-4,5-dihydrooxazole (10)

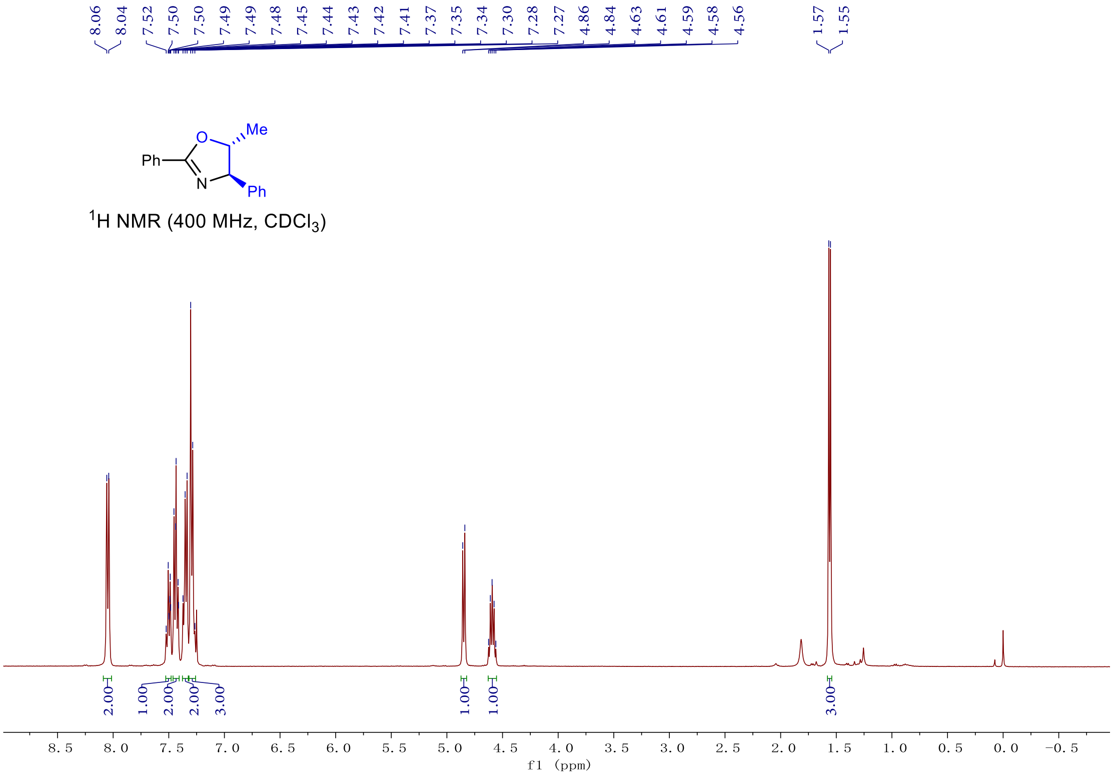

Supplementary Figure 160. ^{1}H NMR spectrum of compound 10.

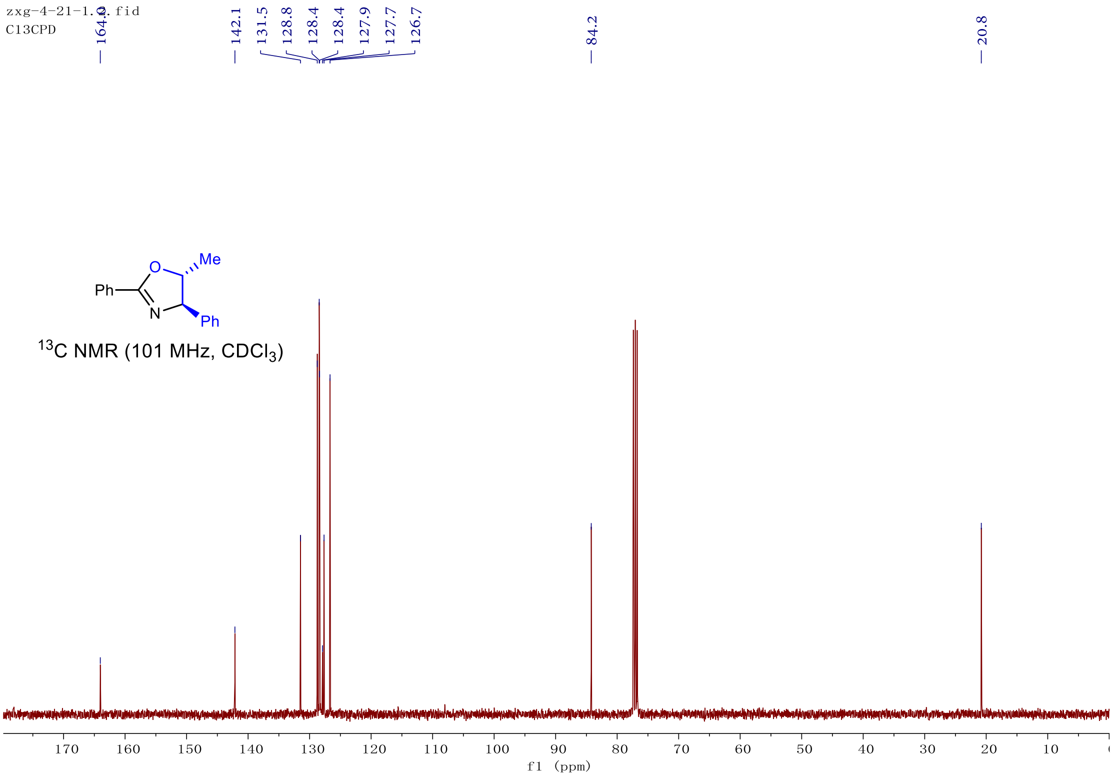

Supplementary Figure 161. ^{13}C NMR spectrum of compound 10.

(*R*)-5-chloro-1-methyl-N-(2-oxo-1-phenylpropyl)-1H-indole-2-carboxamide (11)

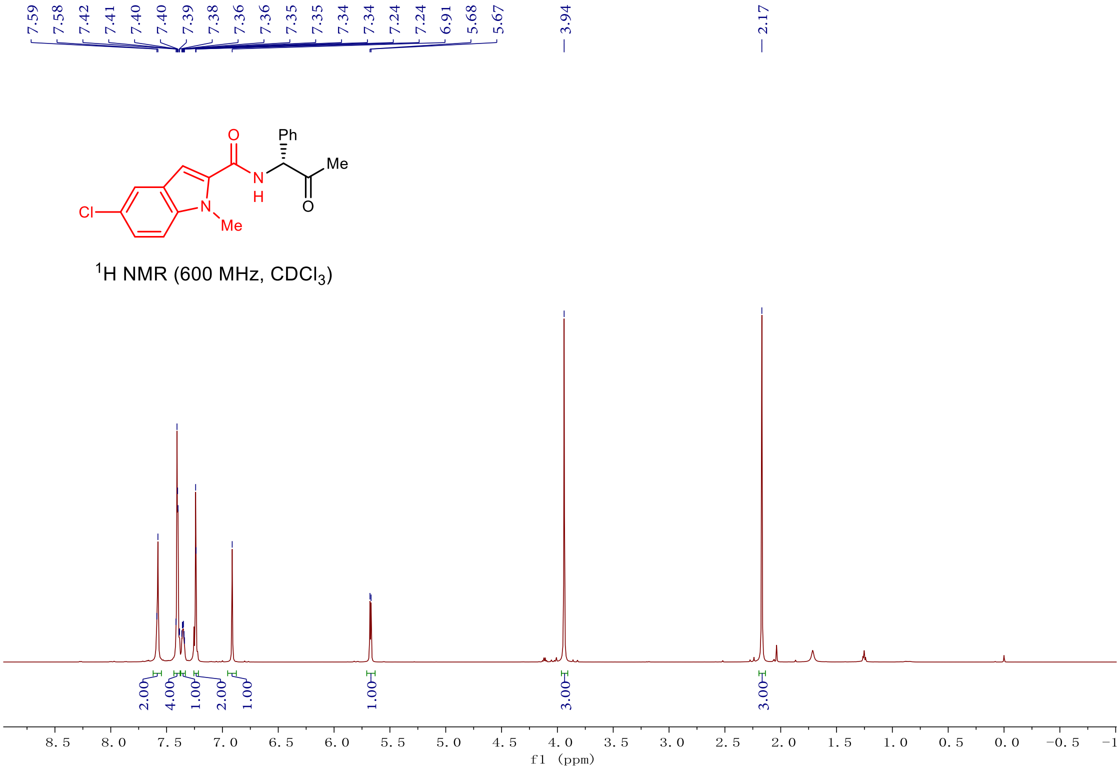

Supplementary Figure 162. ^{1}H NMR spectrum of compound 11.

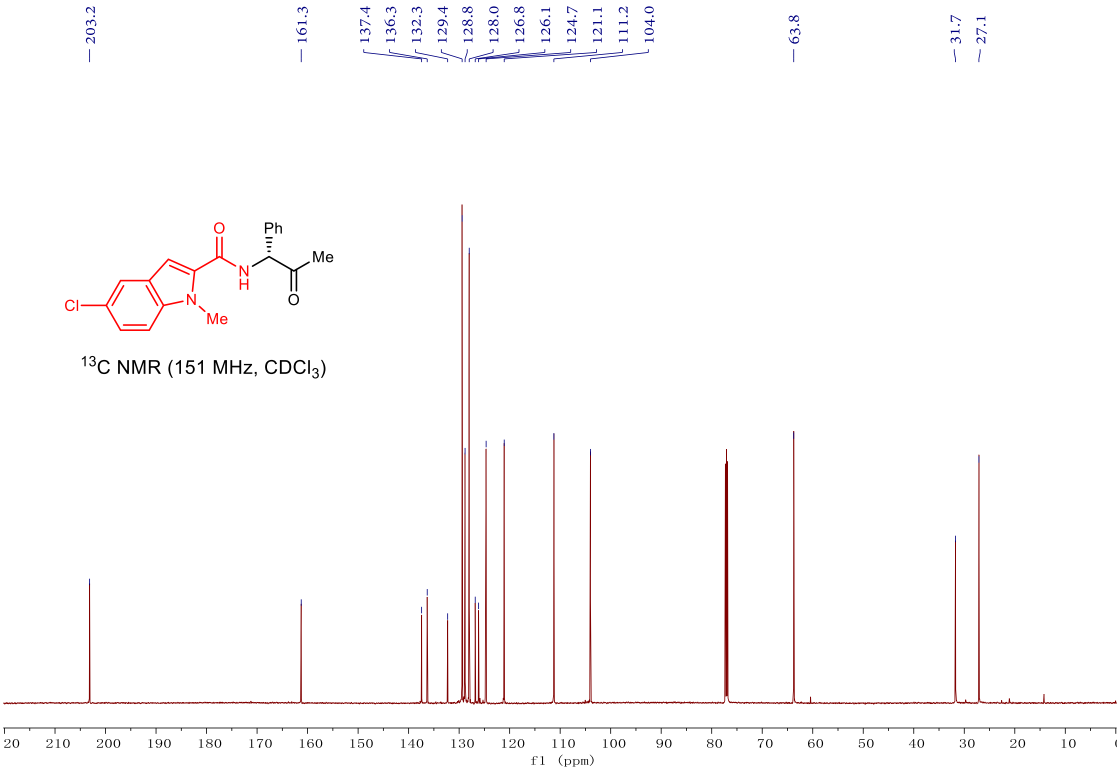

Supplementary Figure 163. ^{13}C NMR spectrum of compound 11.

5-chloro-N-((1*R*,2*R*)-2-hydroxy-1-phenylpropyl)-1-methyl-1H-indole-2-carboxamide (12)

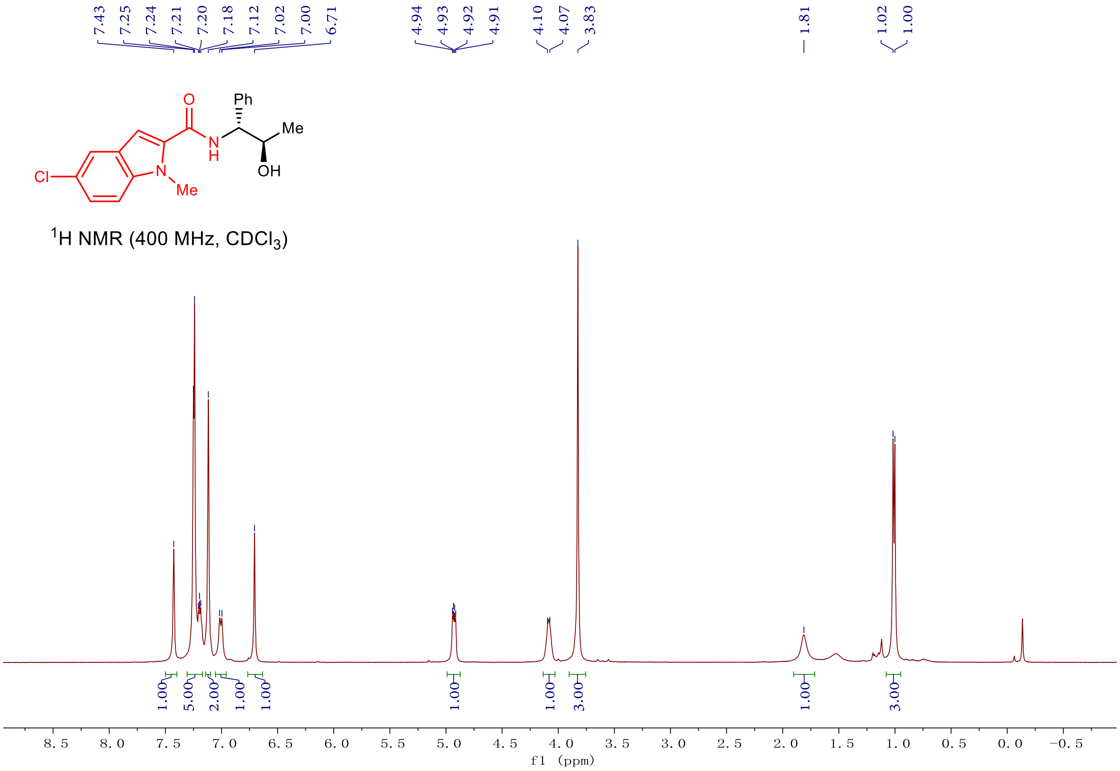

Supplementary Figure 164. ^{1}H NMR spectrum of compound 12.

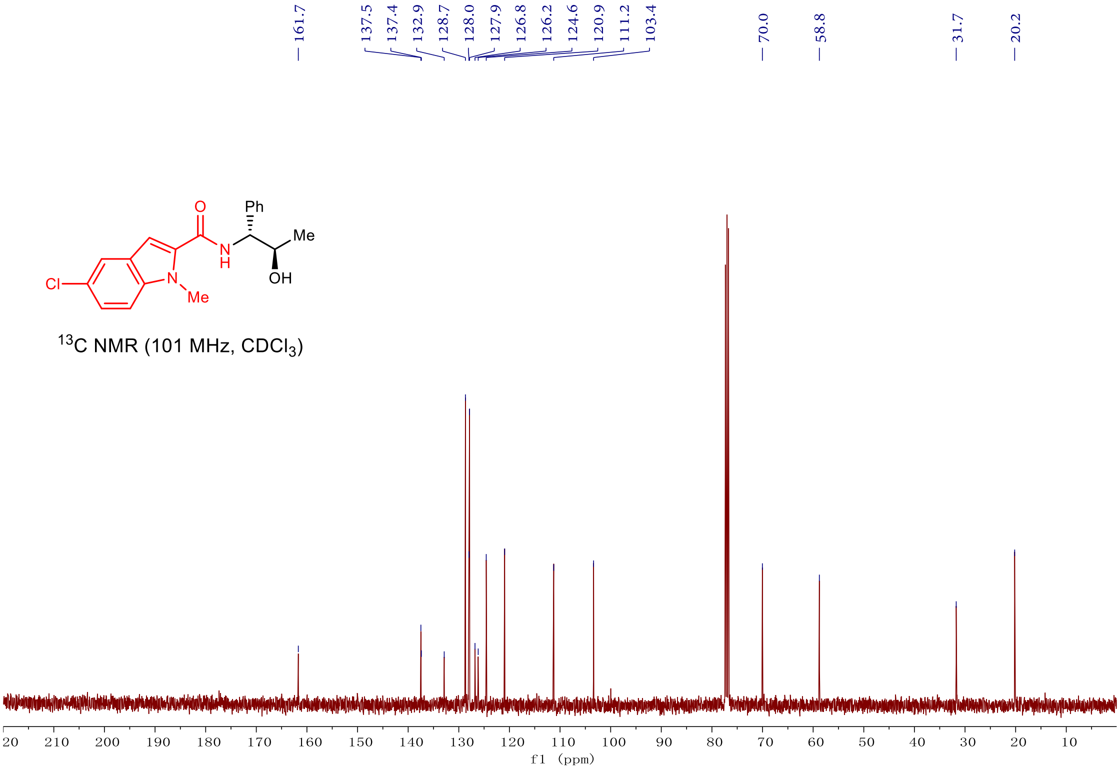

Supplementary Figure 165. ^{13}C NMR spectrum of compound 12.

2.2 HPLC Spectra

Supplementary Figure 166. HPLC spectrum of (*R*)-N-(2-oxo-1-phenylpropyl)-3-phenylpropanamide (3a) and (*S*)-N-(2-oxo-1-phenylpropyl)-3-phenylpropanamide (*ent*-3a)

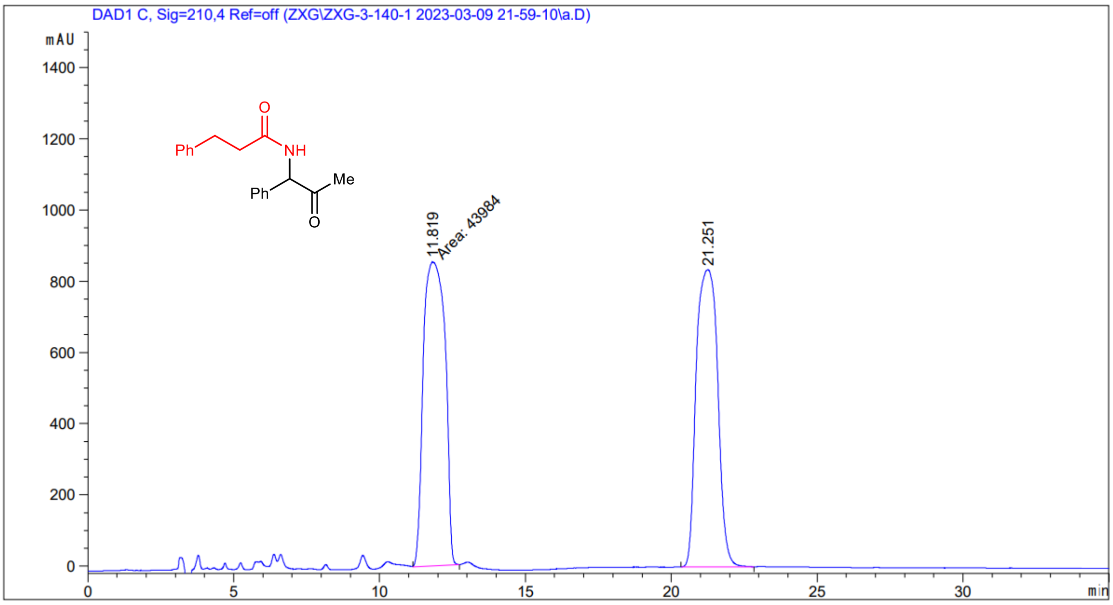

Peak #	RetTime [min]	Type	Width [min]	Area [mAU*s]	Height [mAU]	Area %
1	11.819	FM	0.8587	4.39840e4	853.72015	50.7595
2	21.251	BB	0.6023	4.26678e4	834.79480	49.2405

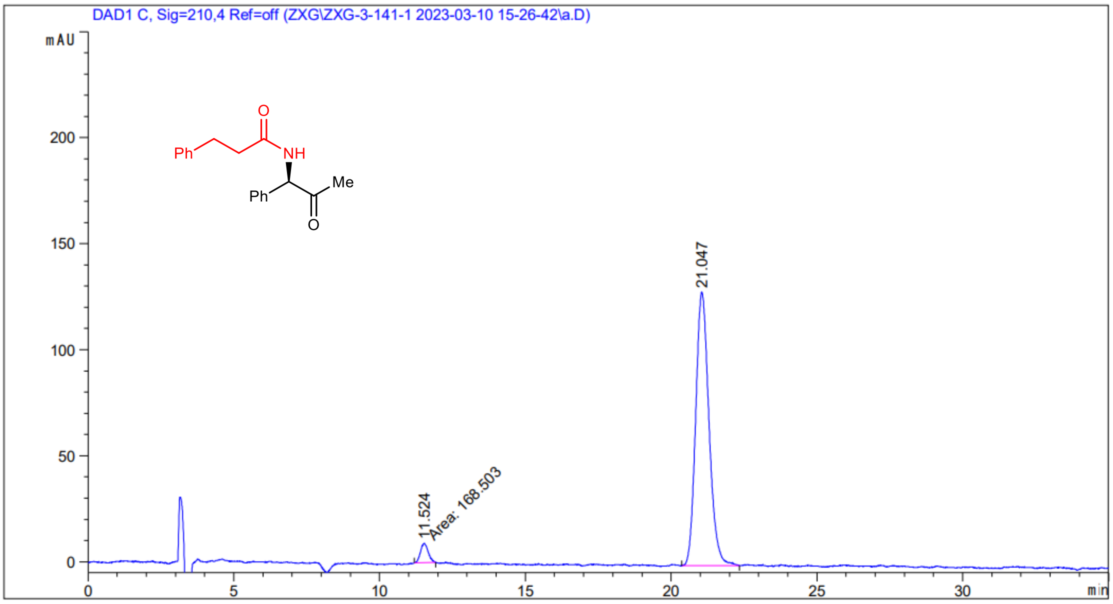

Peak #	RetTime [min]	Type	Width [min]	Area [mAU*s]	Height [mAU]	Area %
1	11.524	MM	0.3074	168.50296	9.13548	3.9564
2	21.047	BV R	0.4779	4090.51294	129.01549	96.0436

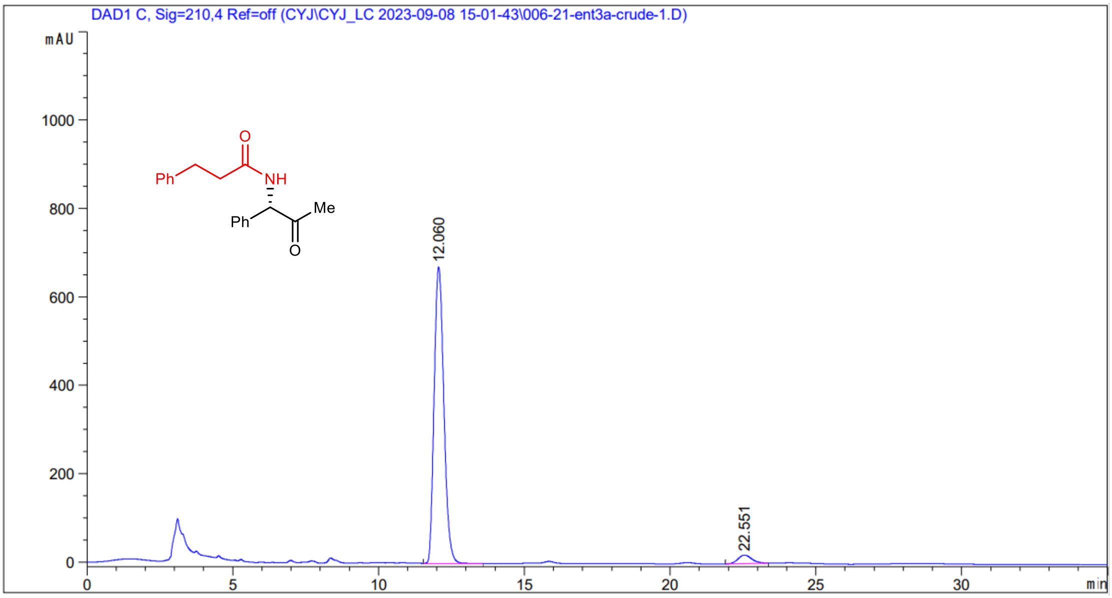

峰 #	保留时间 [min]	类型	峰宽 [min]	峰面积 [mAU*s]	峰高 [mAU]	峰面积 %
1	12.060	BB	0.3654	1.54961e4	670.97510	96.1000
2	22.551	BB	0.4586	628.86737	19.27812	3.9000

Supplementary Figure 167. HPLC spectrum of (*R*)-N-(2-oxo-1-phenylpropyl)-2-phenylacetamide (3b)

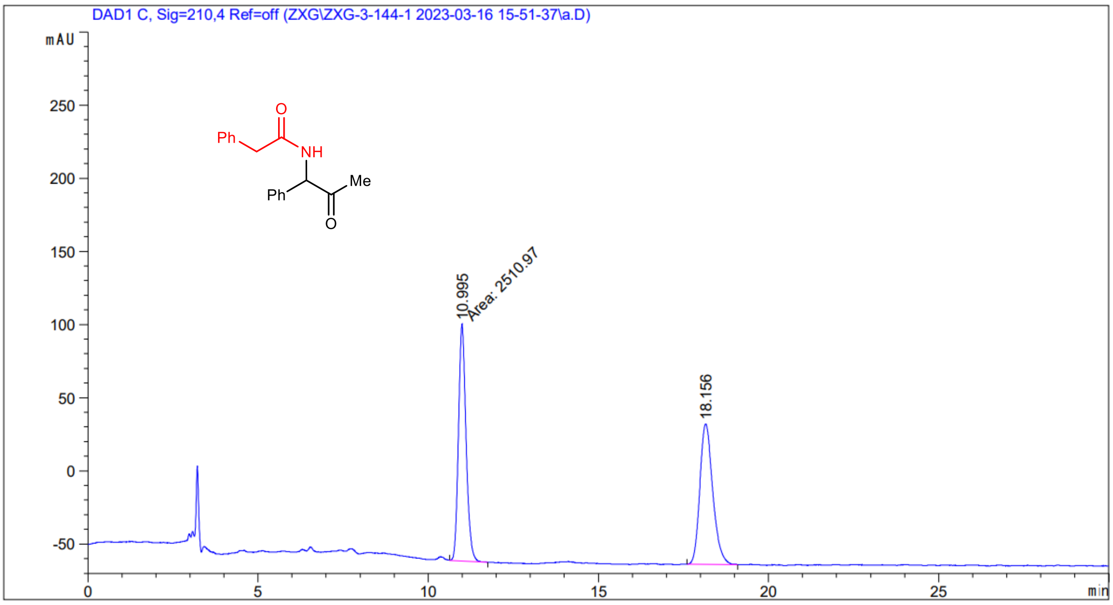

Peak #	RetTime [min]	Type	Width [min]	Area [mAU*s]	Height [mAU]	Area %
1	10.995	FM	0.2582	2510.97437	162.10052	49.8156
2	18.156	BB	0.4073	2529.56006	96.03963	50.1844

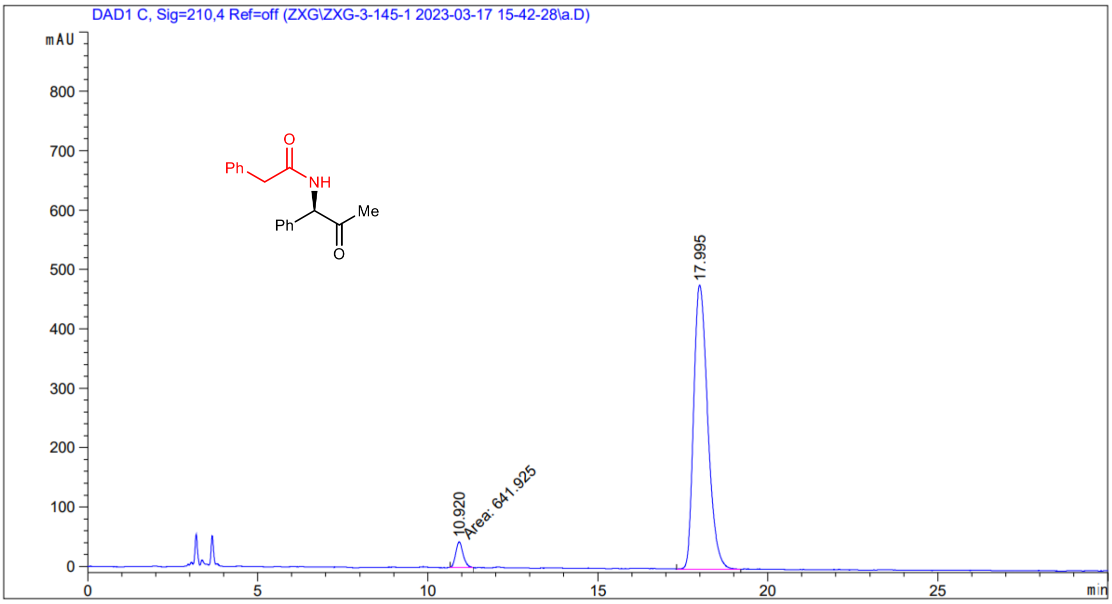

Peak #	RetTime [min]	Type	Width [min]	Area [mAU*s]	Height [mAU]	Area %
1	10.920	MM	0.2502	641.92480	42.75703	4.4606
2	17.995	BB	0.4493	1.37489e4	478.32968	95.5394

Supplementary Figure 168. HPLC spectrum of (*R*)-N-(2-oxo-1-phenylpropyl)acetamide (3c)

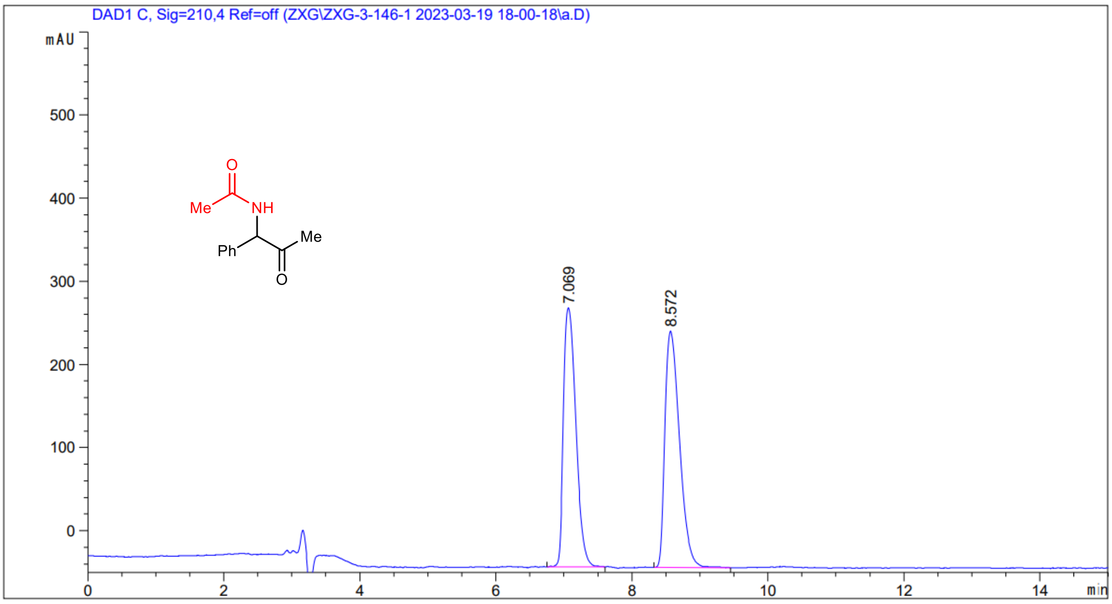

Peak #	RetTime [min]	Type	Width [min]	Area [mAU*s]	Height [mAU]	Area %
1	7.069	VV R	0.2016	3939.76318	311.43127	48.0049
2	8.572	BV R	0.2305	4267.23389	284.61847	51.9951

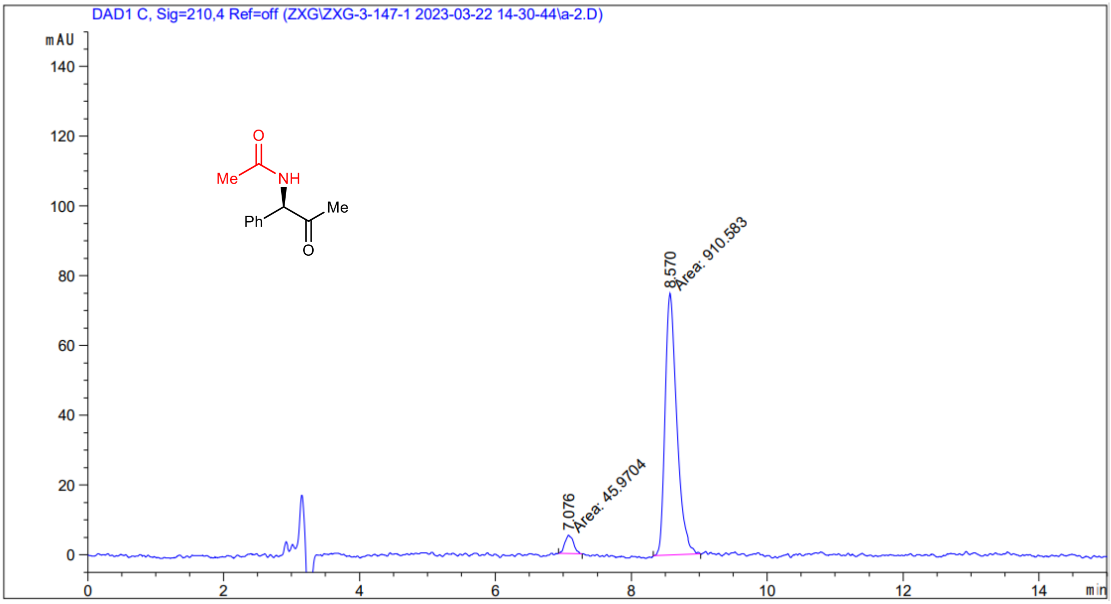

Peak #	RetTime [min]	Type	Width [min]	Area [mAU*s]	Height [mAU]	Area %
1	7.076	MM	0.1453	45.97036	5.27439	4.8058
2	8.570	MM	0.2020	910.58276	75.14705	95.1942

Supplementary Figure 169. HPLC spectrum of (*R*)-N-(2-oxo-1-phenylpropyl)propionamide (3d)

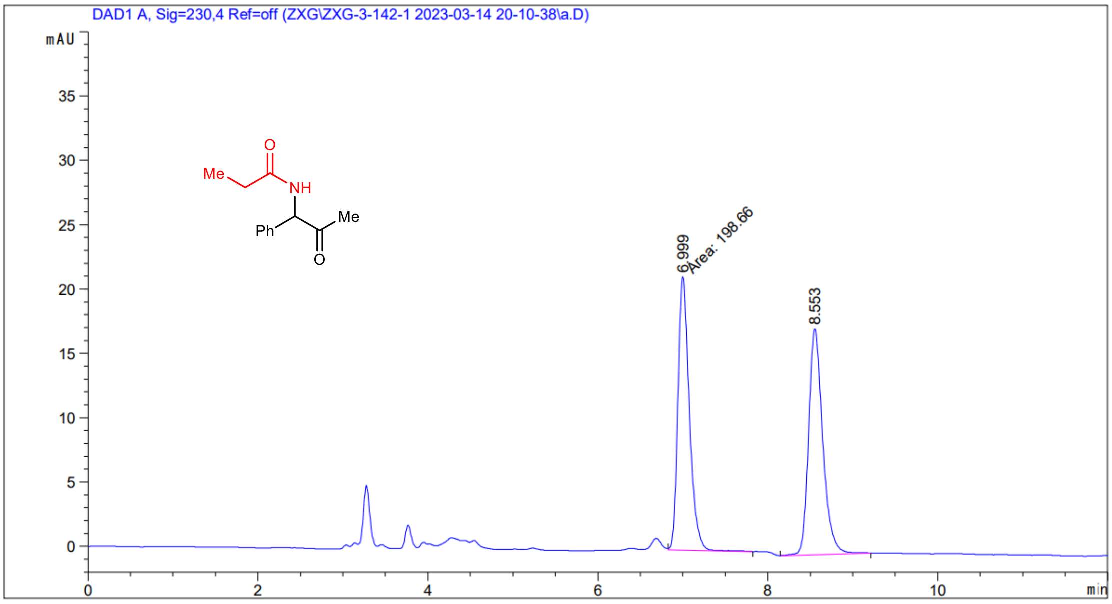

Peak #	RetTime [min]	Type	Width [min]	Area [mAU*s]	Height [mAU]	Area %
1	6.999	FM	0.1556	198.65973	21.27899	49.4475
2	8.553	BB	0.1769	203.09882	17.56004	50.5525

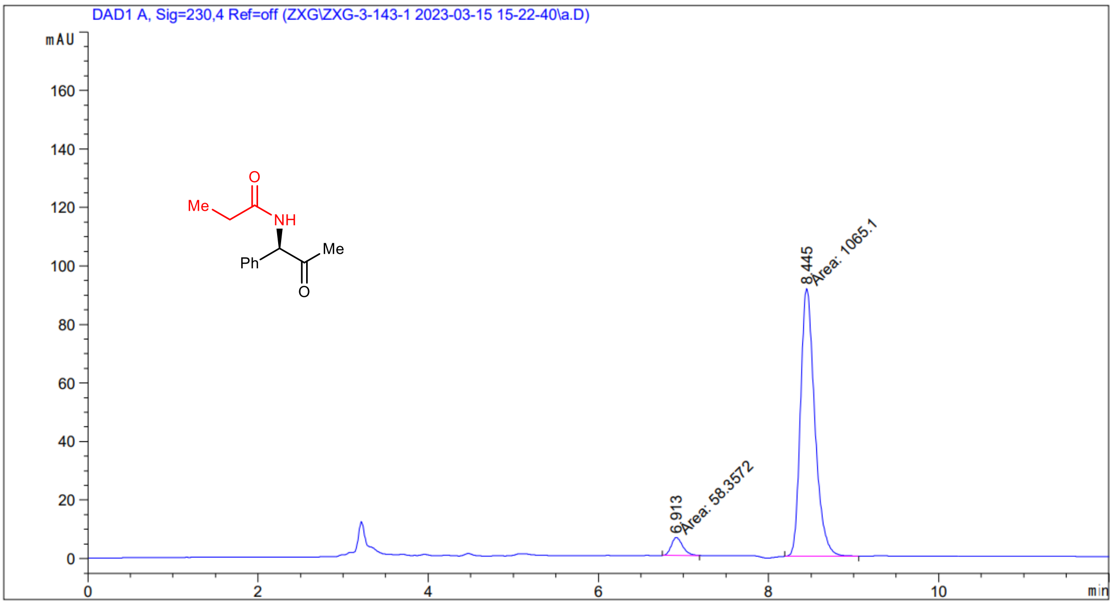

Peak #	RetTime [min]	Type	Width [min]	Area [mAU*s]	Height [mAU]	Area %
1	6.913	MM	0.1567	58.35719	6.20602	5.1944
2	8.445	MM	0.1940	1065.09900	91.52132	94.8056

Supplementary Figure 170. HPLC spectrum of (*R*)-N-(2-oxo-1-phenylpropyl)butyramide (3e)

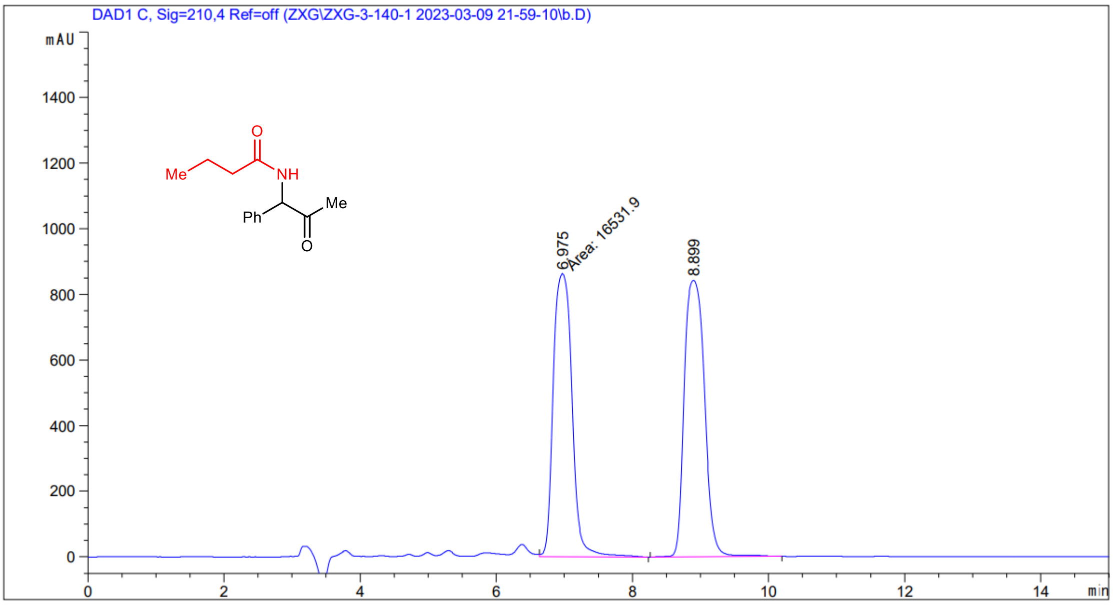

Peak #	RetTime [min]	Type	Width [min]	Area [mAU*s]	Height [mAU]	Area %
1	6.975	FM	0.3192	1.65319e4	863.29266	48.6646
2	8.899	BV R	0.3350	1.74392e4	843.15002	51.3354

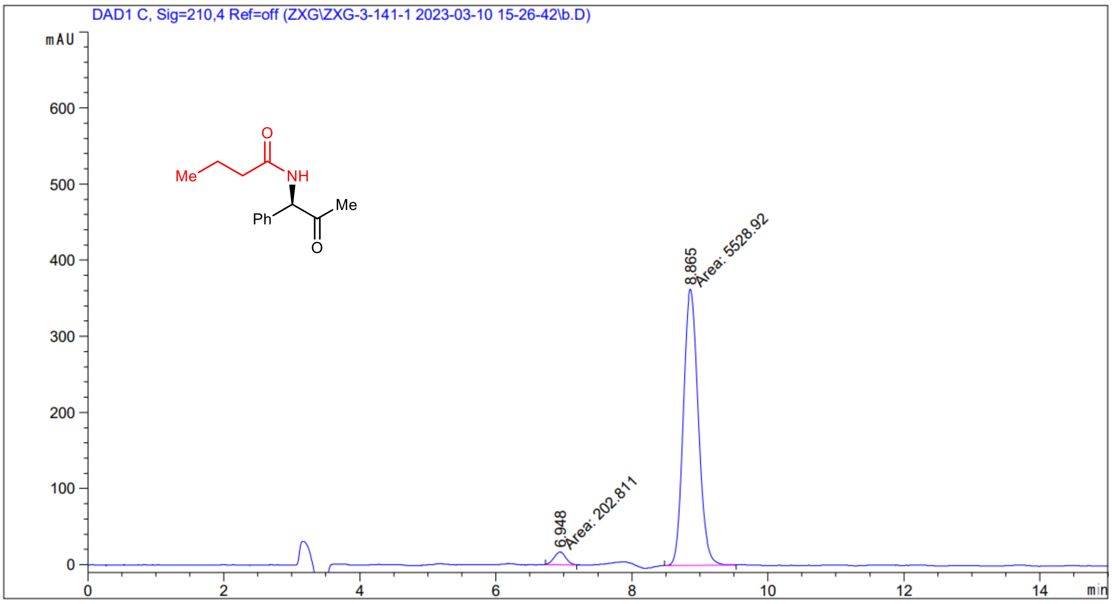

Peak #	RetTime [min]	Type	Width [min]	Area [mAU*s]	Height [mAU]	Area %
1	6.948	MM	0.2009	202.81139	16.82807	3.5384
2	8.865	MM	0.2540	5528.92334	362.74643	96.4616

Supplementary Figure 171. HPLC spectrum of (*R*)-N-(2-oxo-1-phenylpropyl)isobutyramide (3f)

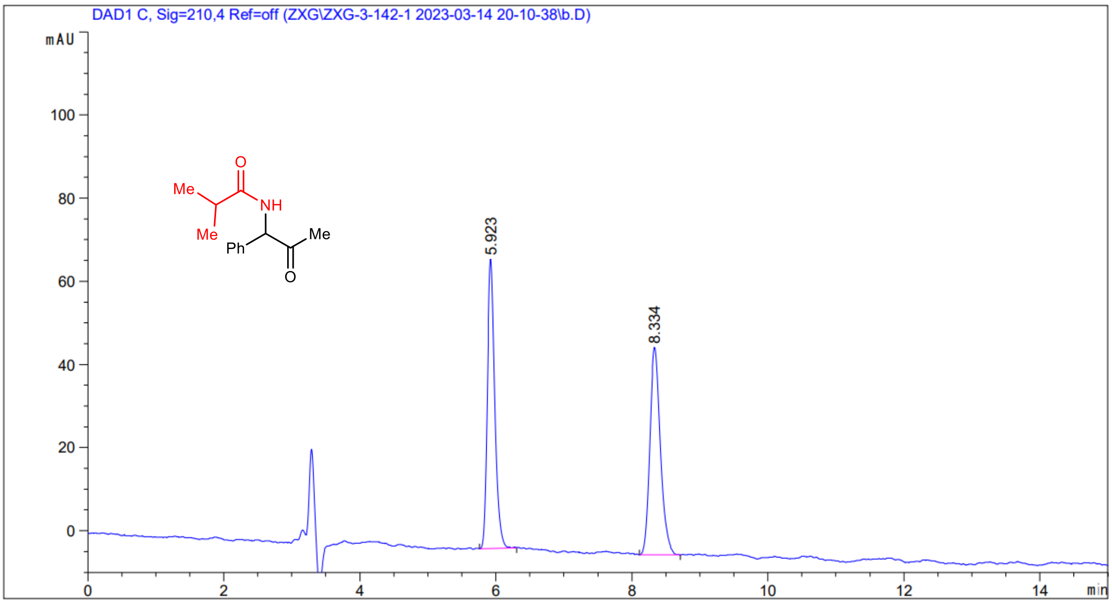

Peak #	RetTime [min]	Type	Width [min]	Area [mAU*s]	Height [mAU]	Area %
1	5.923	BV R	0.1168	530.07495	69.62437	49.5566
2	8.334	BB	0.1662	539.56079	49.87043	50.4434

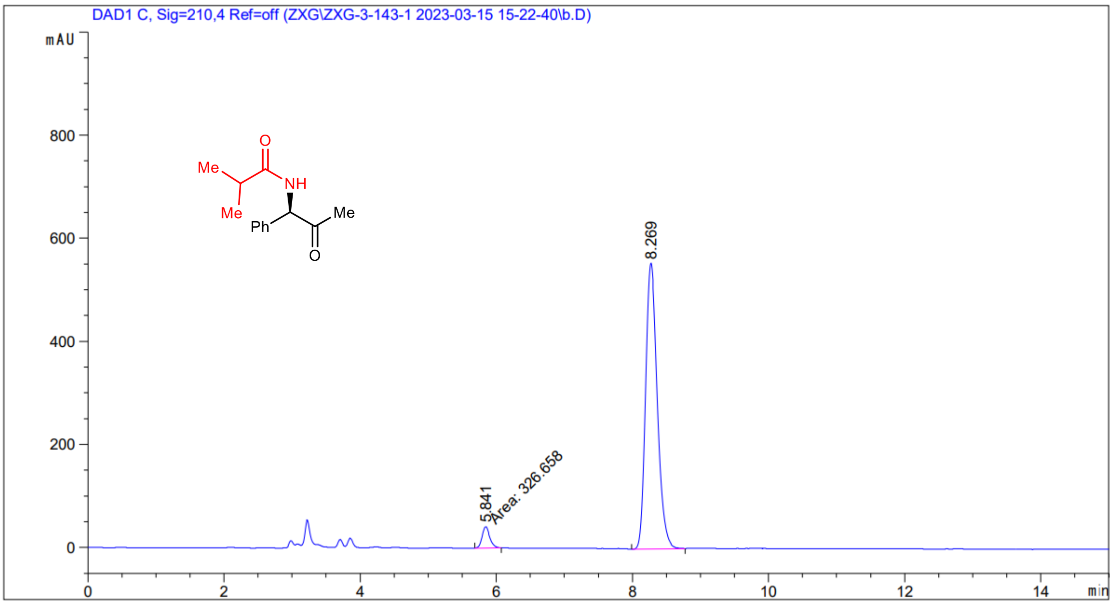

Peak #	RetTime [min]	Type	Width [min]	Area [mAU*s]	Height [mAU]	Area %
1	5.841	MM	0.1314	326.65762	41.42654	4.6988
2	8.269	BB	0.1854	6625.25635	554.50470	95.3012

Supplementary Figure 172. HPLC spectrum of (*R*)-N-(2-oxo-1-phenylpropyl)pivalamide (3g)

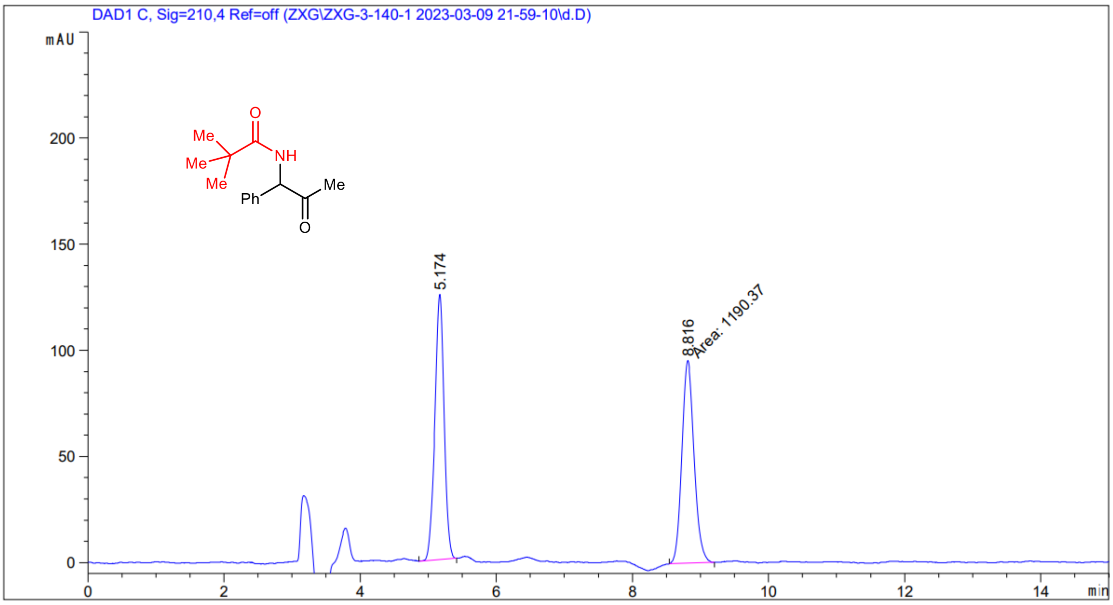

Peak #	RetTime [min]	Type	Width [min]	Area [mAU*s]	Height [mAU]	Area %
1	5.174	BB	0.1418	1175.07031	124.86395	49.6766
2	8.816	MM	0.2078	1190.36829	95.46886	50.3234

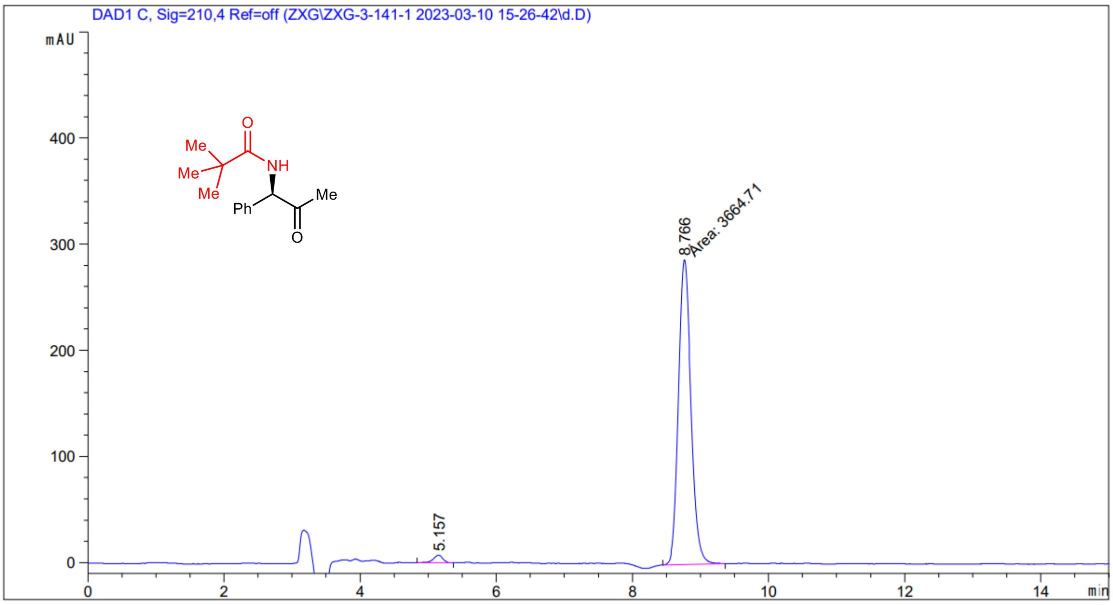

Peak #	RetTime [min]	Type	Width [min]	Area [mAU*s]	Height [mAU]	Area %
1	5.157	VB R	0.1400	69.04695	7.18947	1.8493
2	8.766	MM	0.2126	3664.71484	287.31079	98.1507

Supplementary Figure 173. HPLC spectrum of Methyl (*R*)-3-((2-oxo-1-phenylpropyl)carbamoyl)bicyclo[1.1.1]pentane-1-carboxylate (3h)

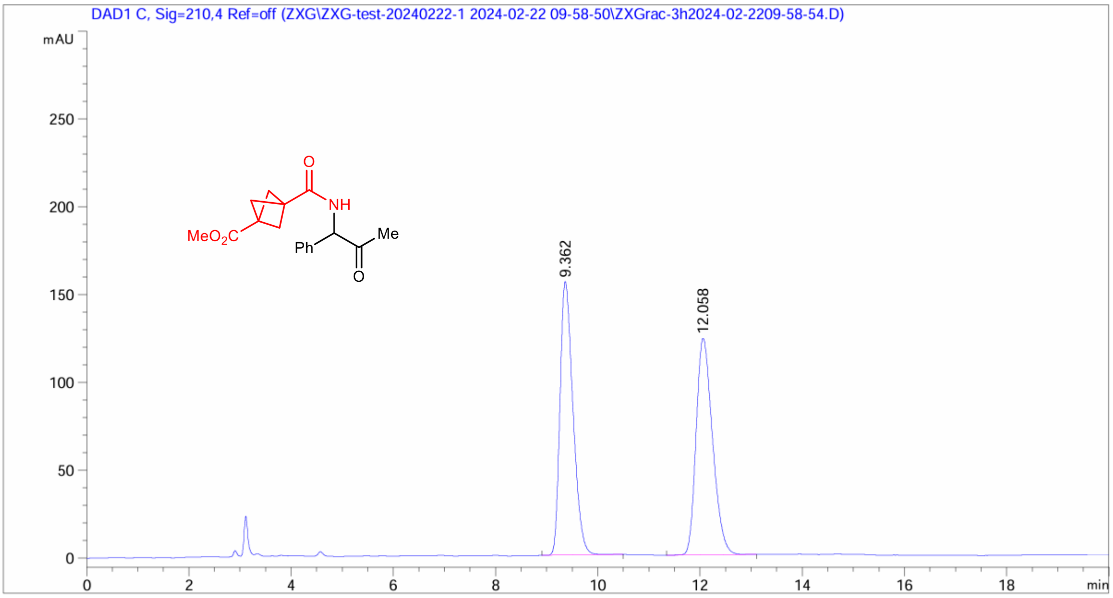

峰 #	保留时间 [min]	类型	峰宽 [min]	峰面积 [mAU*s]	峰高 [mAU]	峰面积 %
1	9.362	BB	0.2702	2751.77368	155.66449	50.0090
2	12.058	BB	0.3443	2750.77856	123.29362	49.9910

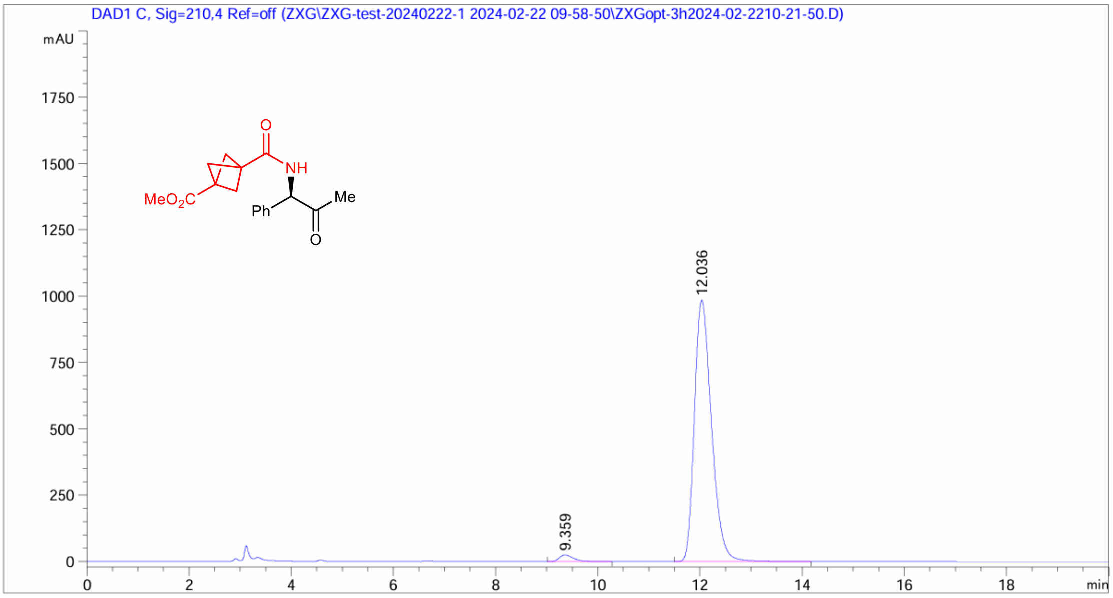

峰 #	保留时间 [min]	类型	峰宽 [min]	峰面积 [mAU*s]	峰高 [mAU]	峰面积 %
1	9.359	BB	0.2854	465.11194	24.72976	1.9896
2	12.036	BB	0.3571	2.29126e4	986.03241	98.0104

Supplementary Figure 174. HPLC spectrum of (*R*)-N-(2-oxo-1-phenylpropyl)cyclopropanecarboxamide (3i)

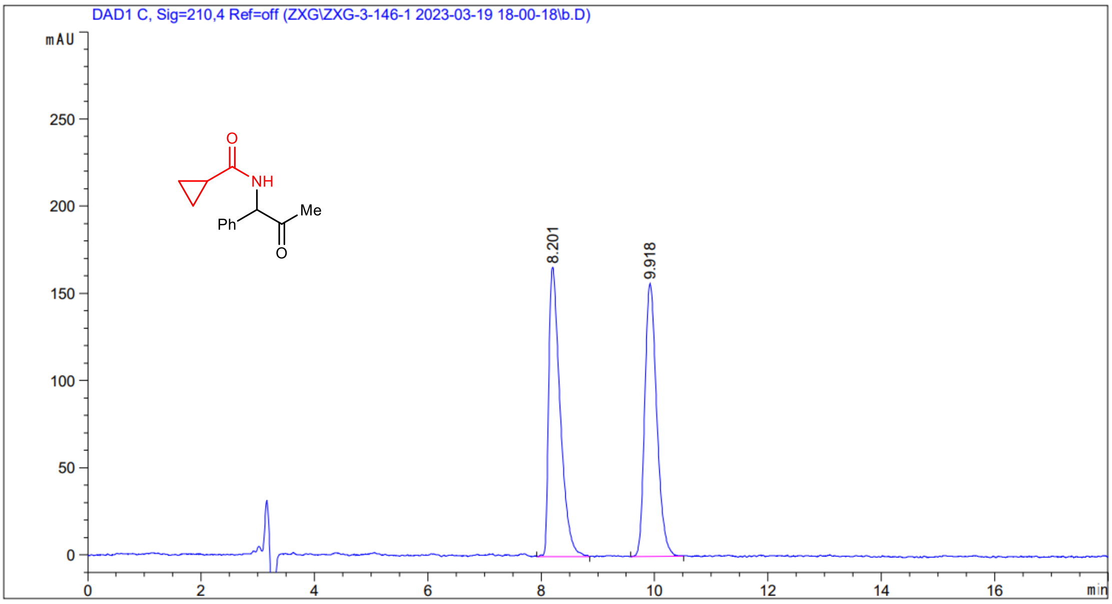

Peak #	RetTime [min]	Type	Width [min]	Area [mAU*s]	Height [mAU]	Area %
1	8.201	VV R	0.2077	2329.53052	166.05791	50.2882
2	9.918	BV R	0.2238	2302.82764	156.40549	49.7118

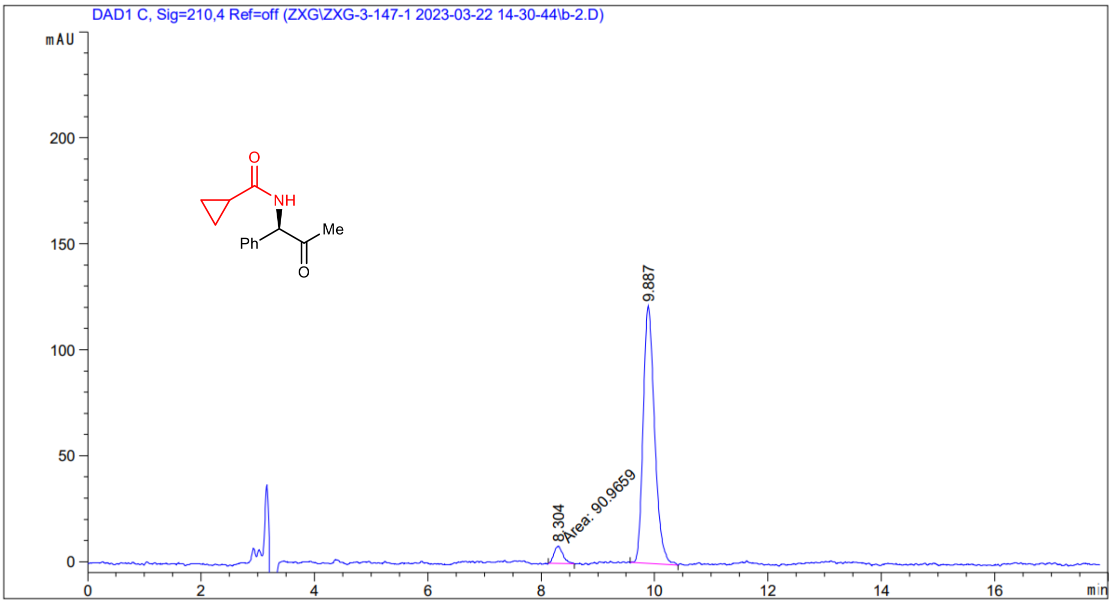

Peak #	RetTime [min]	Type	Width [min]	Area [mAU*s]	Height [mAU]	Area %
1	8.304	MM	0.1842	90.96591	8.22876	5.0788
2	9.887	VV R	0.2153	1700.13855	121.48813	94.9212

Supplementary Figure 175. HPLC spectrum of (*R*)-N-(2-oxo-1-phenylpropyl)cyclobutanecarboxamide (3j)

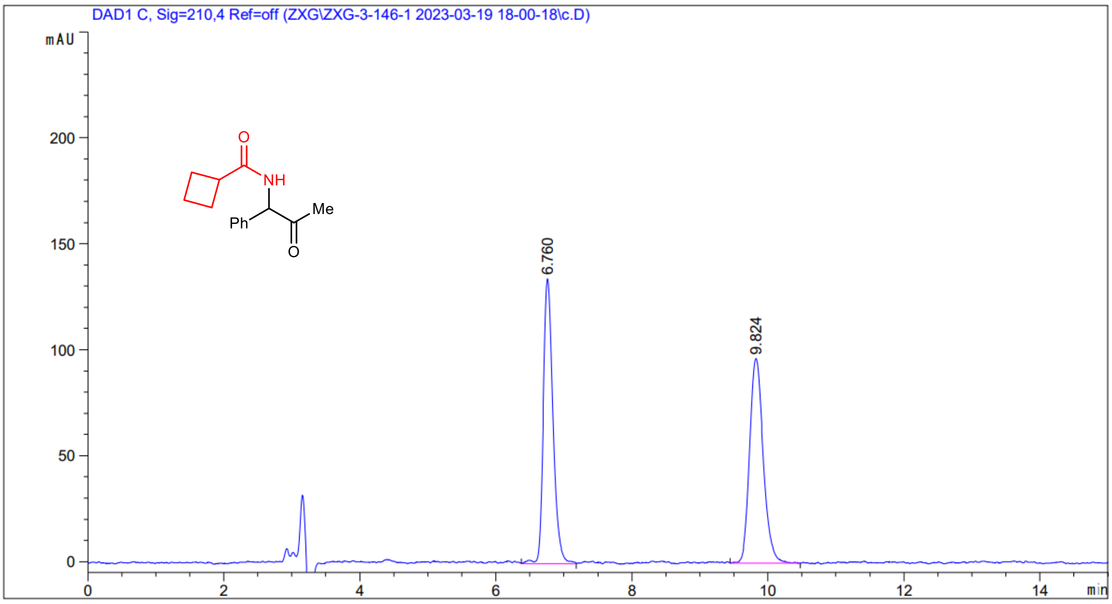

Peak #	RetTime [min]	Type	Width [min]	Area [mAU*s]	Height [mAU]	Area %
1	6.760	VV R	0.1496	1318.28088	134.23711	49.4784
2	9.824	VV R	0.2208	1346.07507	96.47938	50.5216

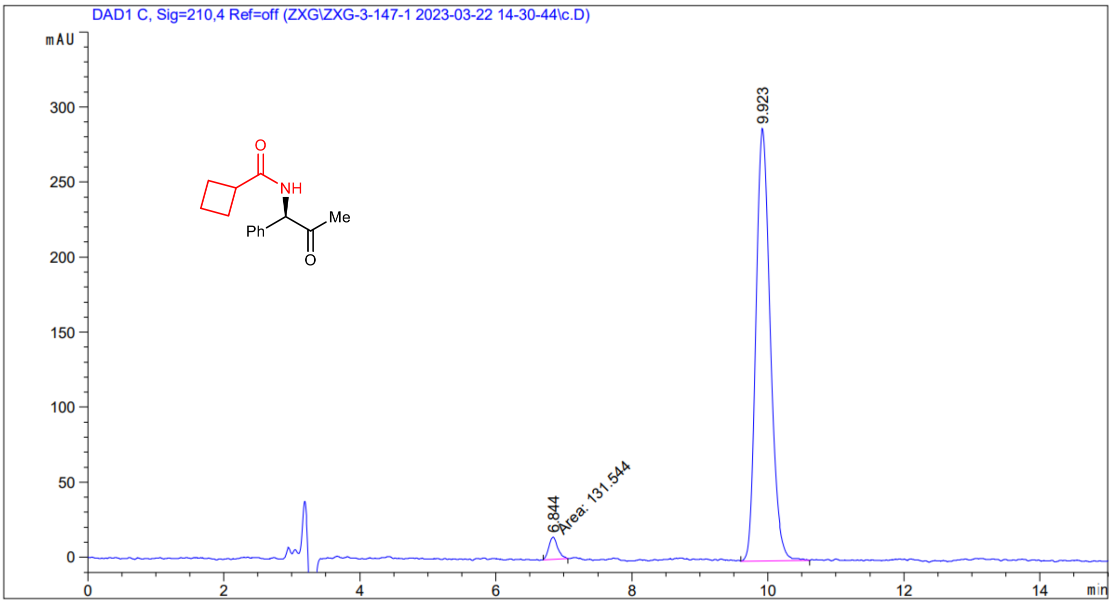

Peak #	RetTime [min]	Type	Width [min]	Area [mAU*s]	Height [mAU]	Area %
1	6.844	MM	0.1485	131.54350	14.76227	3.0114
2	9.923	BV R	0.2316	4236.59668	288.02933	96.9886

Supplementary Figure 176. HPLC spectrum of (*R*)-N-(2-oxo-1-phenylpropyl)cyclohexanecarboxamide (3k)

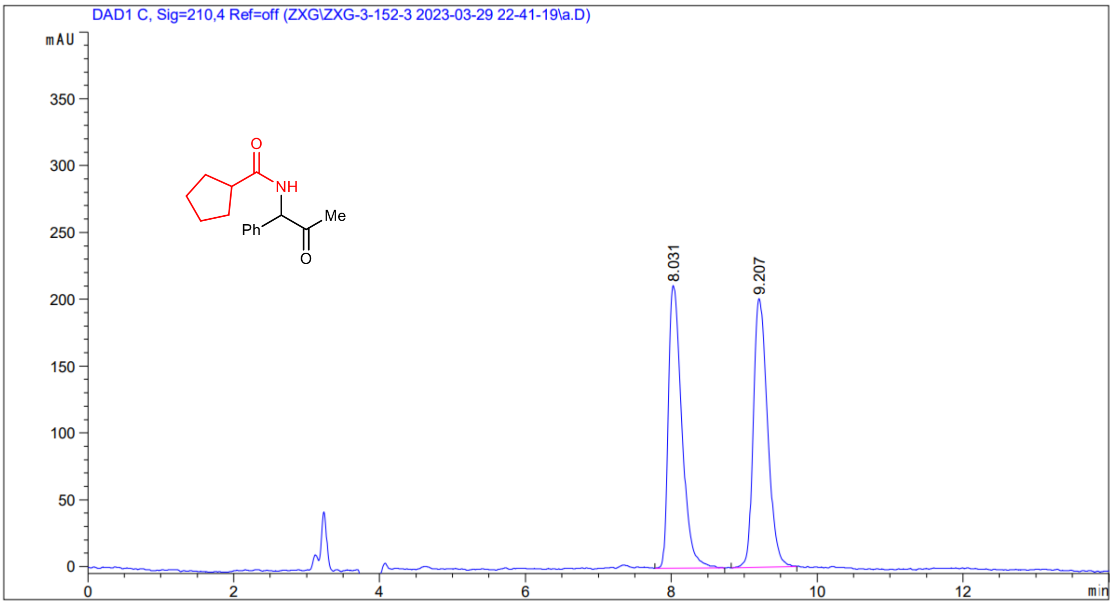

Peak #	RetTime [min]	Type	Width [min]	Area [mAU*s]	Height [mAU]	Area %
1	8.031	BV R	0.1981	2722.35400	211.53304	49.9728
2	9.207	BV R	0.2061	2725.31372	201.11176	50.0272

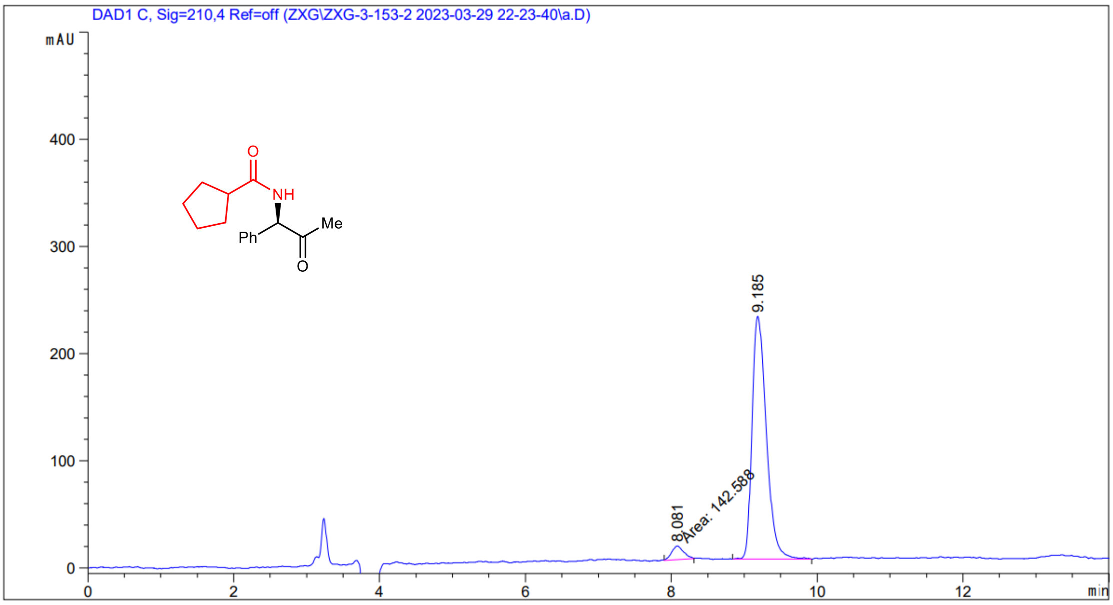

Peak #	RetTime [min]	Type	Width [min]	Area [mAU*s]	Height [mAU]	Area %
1	8.081	MM	0.1873	142.58774	12.68911	4.4098
2	9.185	VV R	0.2091	3090.81128	226.65193	95.5902

Supplementary Figure 177. HPLC spectrum of (*R*)-N-(2-oxo-1-phenylpropyl)cyclohexanecarboxamide (3l)

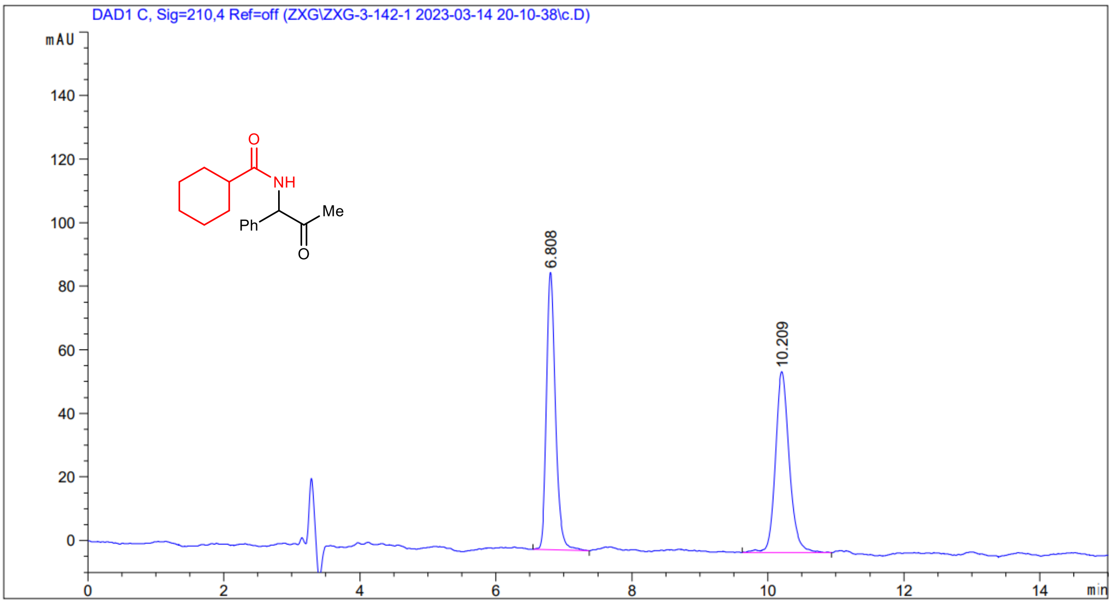

Peak #	RetTime [min]	Type	Width [min]	Area [mAU*s]	Height [mAU]	Area %
1	6.808	BB	0.1388	800.29645	87.43360	49.7197
2	10.209	VB R	0.2140	809.32062	56.88039	50.2803

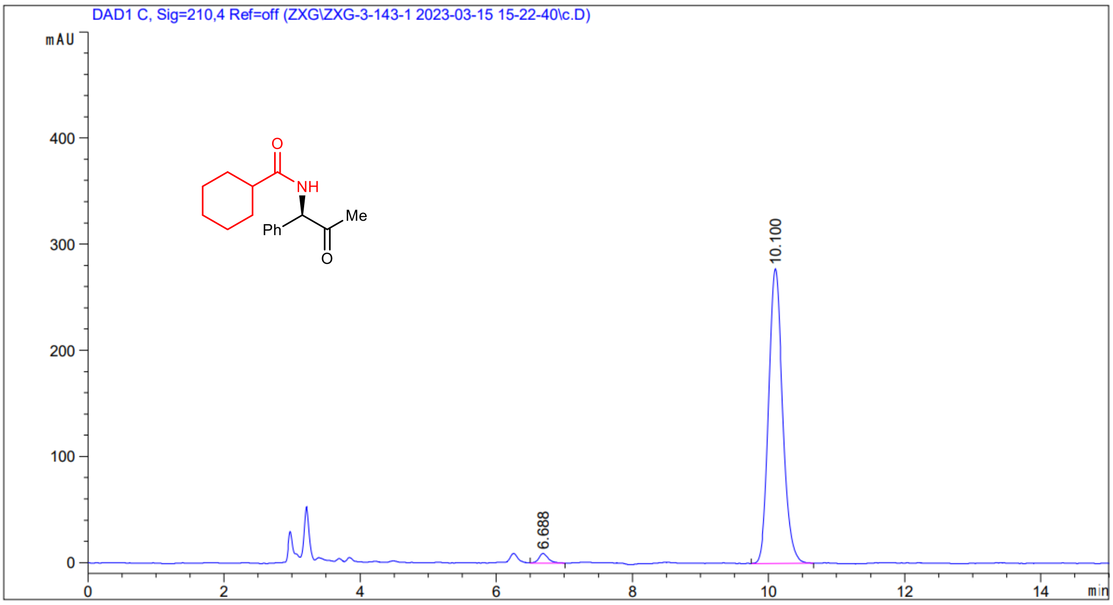

Peak #	RetTime [min]	Type	Width [min]	Area [mAU*s]	Height [mAU]	Area %
1	6.688	VB	0.1472	88.30025	9.10116	2.1867
2	10.100	BB	0.2200	3949.70410	277.56445	97.8133

Supplementary Figure 178. HPLC spectrum of *tert*-butyl (*R*)-4-((2-oxo-1-phenylpropyl)carbamoyl)piperidine-1-carboxylate (3m)

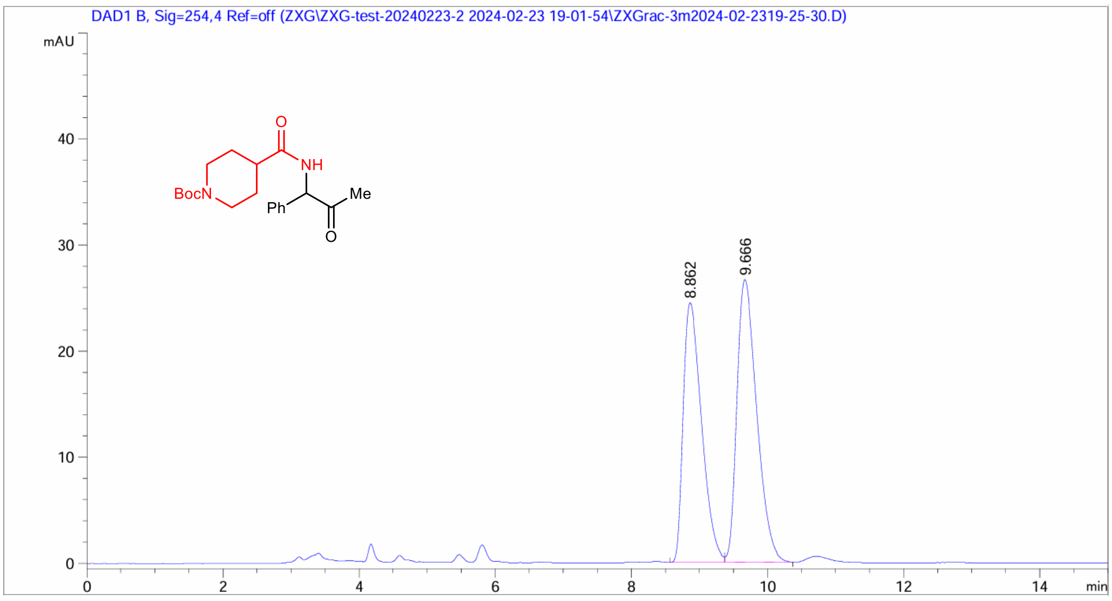

峰 #	保留时间 [min]	类型	峰宽 [min]	峰面积 [mAU*s]	峰高 [mAU]	峰面积 %
1	8.862	BV	0.2957	461.17206	24.48676	46.0124
2	9.666	VB	0.3129	541.10492	26.64327	53.9876

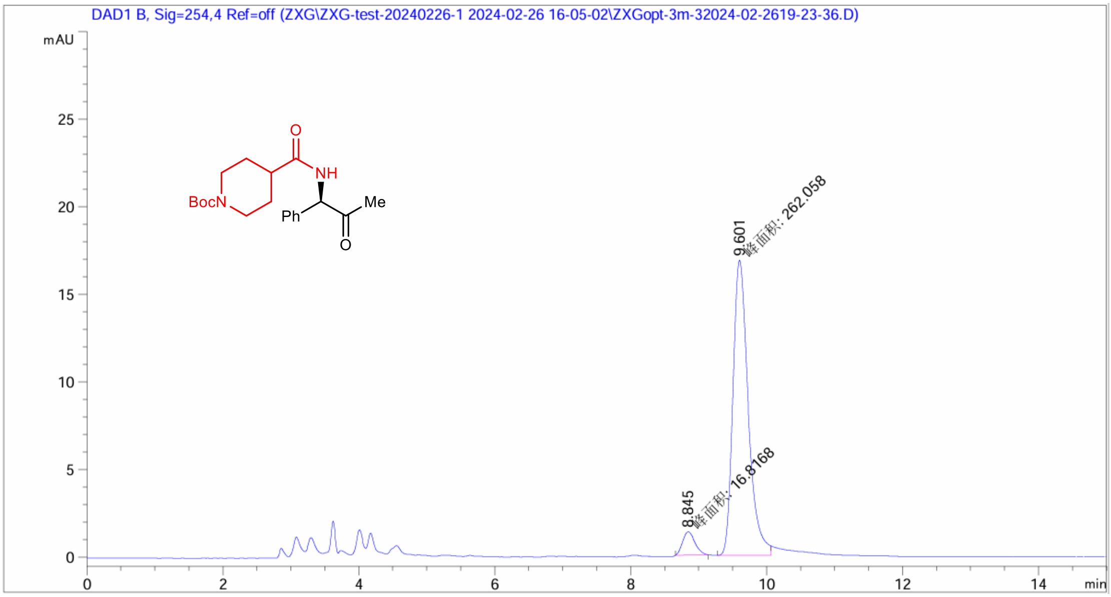

峰 #	保留时间 [min]	类型	峰宽 [min]	峰面积 [mAU*s]	峰高 [mAU]	峰面积 %
1	8.845	MM	0.2098	16.81676	1.33564	6.0302
2	9.601	MF	0.2596	262.05817	16.82139	93.9698

Supplementary Figure 179. HPLC spectrum of (*R*)-N-(2-oxo-1-phenylpropyl)tetrahydro-2H-pyran-4-carboxamide (3n)

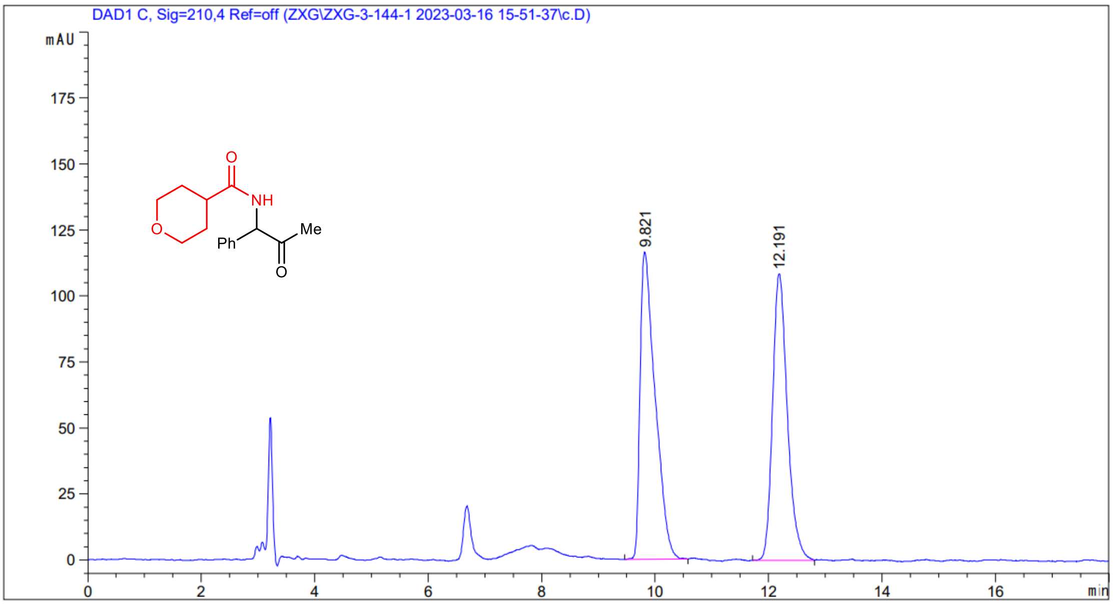

Peak #	RetTime [min]	Type	Width [min]	Area [mAU*s]	Height [mAU]	Area %
1	9.821	BB	0.2689	2145.37134	116.49549	52.2247
2	12.191	BB	0.2750	1962.59521	108.53320	47.7753

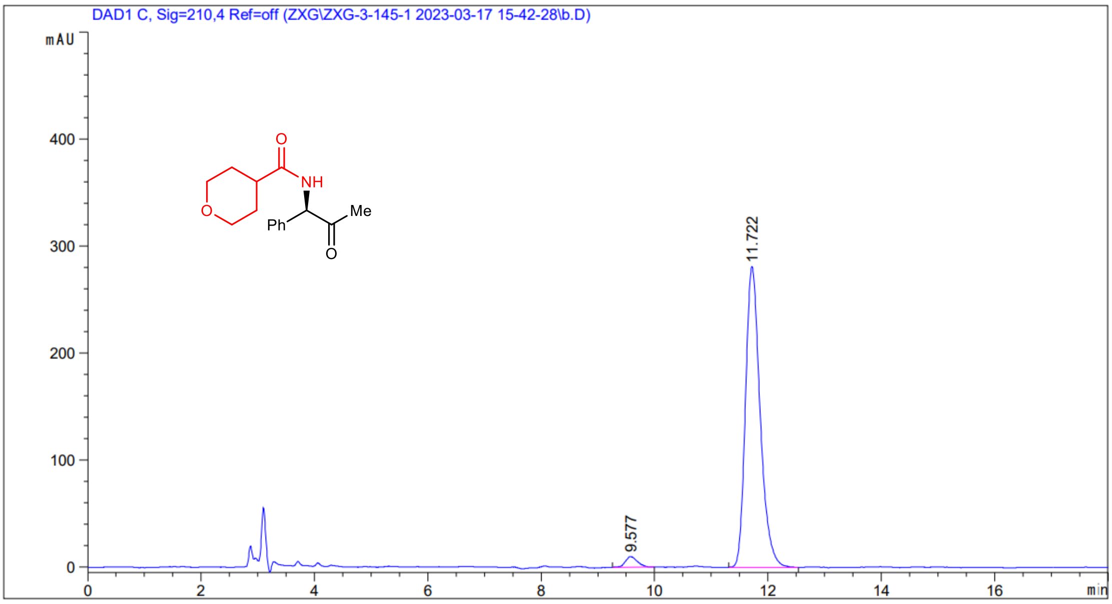

Peak #	RetTime [min]	Type	Width [min]	Area [mAU*s]	Height [mAU]	Area %
1	9.577	BB	0.2242	149.71127	10.02674	2.8766
2	11.722	BB	0.2758	5054.82227	281.13309	97.1234

Supplementary Figure 180. HPLC spectrum of (*R*)-N-(2-oxo-1-phenylpropyl)cycloheptanecarboxamide (3o)

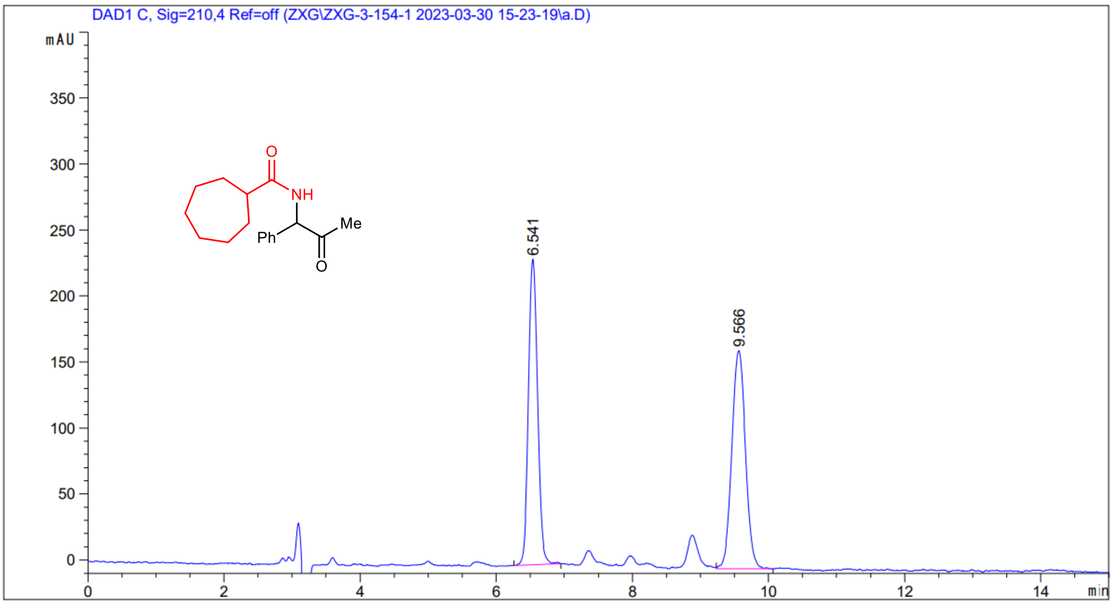

Peak #	RetTime [min]	Type	Width [min]	Area [mAU*s]	Height [mAU]	Area %
1	6.541	BV R	0.1443	2146.67896	231.26904	48.3010
2	9.566	VV R	0.2183	2297.69482	165.20981	51.6990

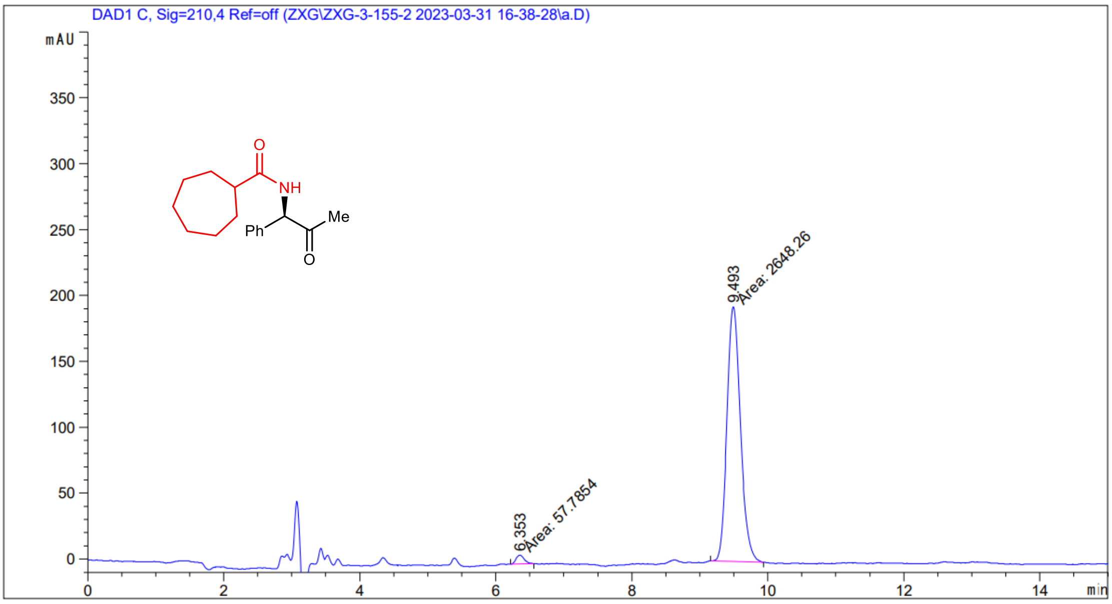

Peak #	RetTime [min]	Type	Width [min]	Area [mAU*s]	Height [mAU]	Area %
1	6.353	MM	0.1442	57.78539	6.68040	2.1354
2	9.493	MM	0.2285	2648.26465	193.16435	97.8646

Supplementary Figure 181. HPLC spectrum of (*R*)-N-(2-oxo-1-phenylpropyl)benzamide (3p)

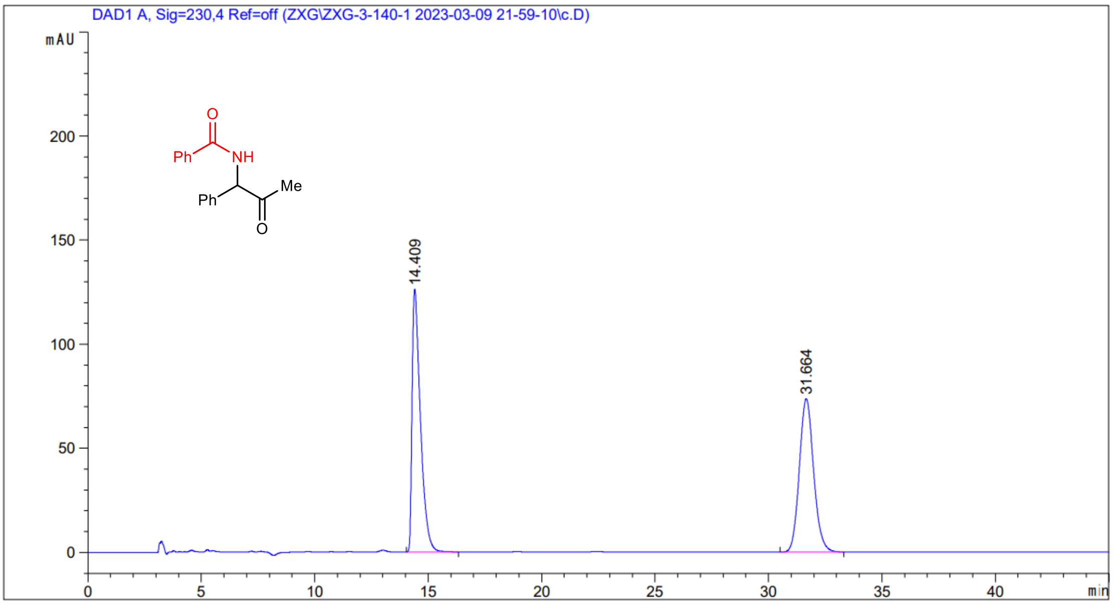

Peak #	RetTime [min]	Type	Width [min]	Area [mAU*s]	Height [mAU]	Area %
1	14.409	BB	0.3816	3220.35400	126.23434	49.9994
2	31.664	BB	0.6739	3220.43433	73.67891	50.0006

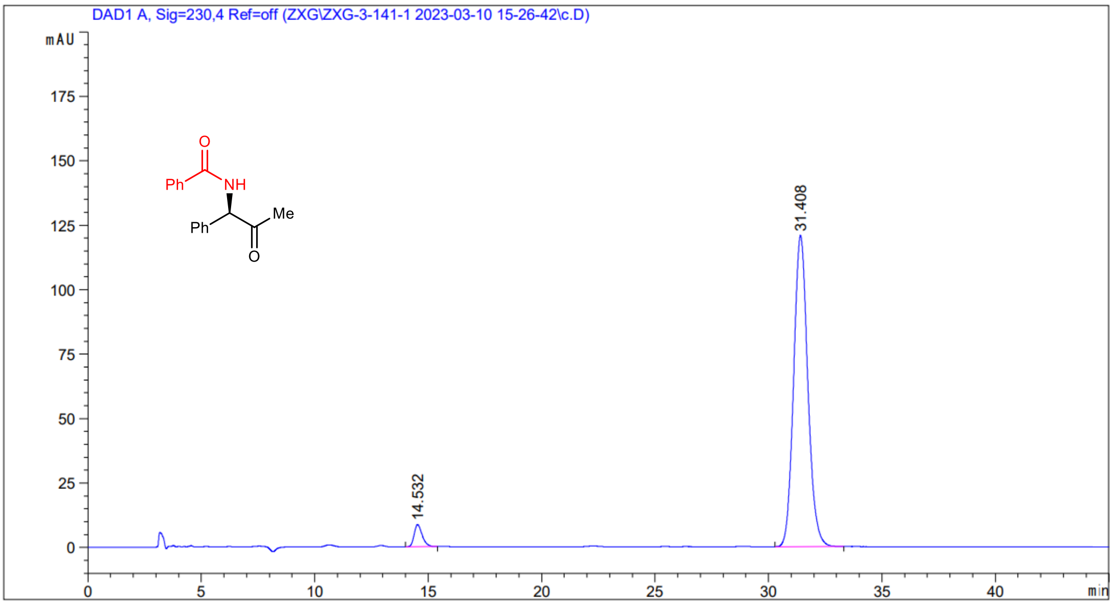

Peak #	RetTime [min]	Type	Width [min]	Area [mAU*s]	Height [mAU]	Area %
1	14.532	BB	0.3773	212.58931	8.57352	3.8986
2	31.408	BB	0.6680	5240.34521	120.82393	96.1014

Supplementary Figure 182. HPLC spectrum of (*R*)-4-methoxy-N-(2-oxo-1-phenylpropyl)benzamide (3q)

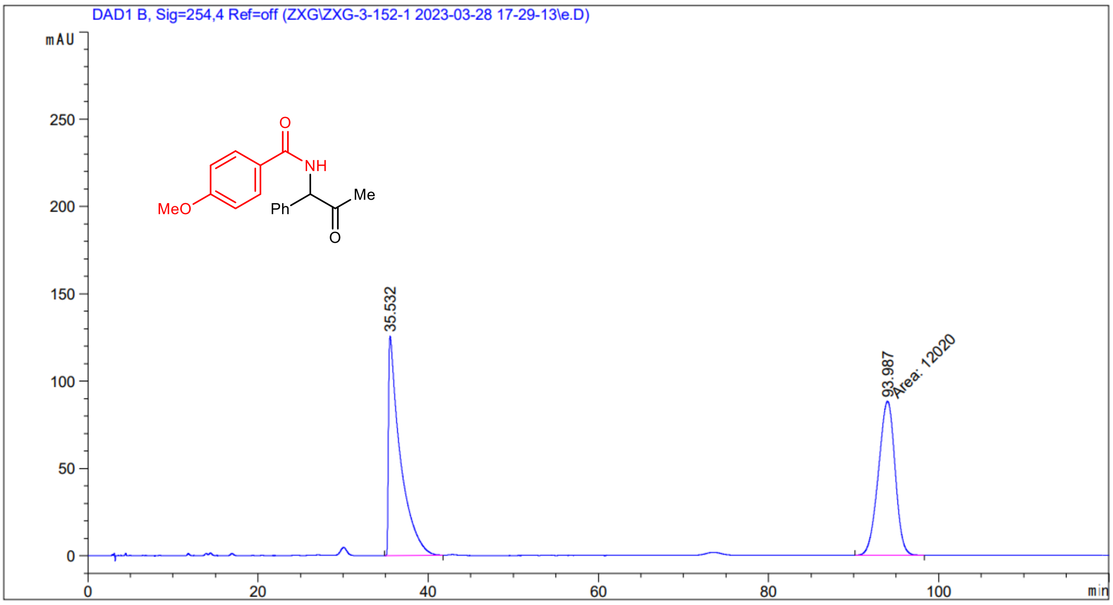

Peak #	RetTime [min]	Type	Width [min]	Area [mAU*s]	Height [mAU]	Area %
1	35.532	BB	1.2642	1.20088e4	125.51913	49.9767
2	93.987	MM	2.2707	1.20200e4	88.22691	50.0233

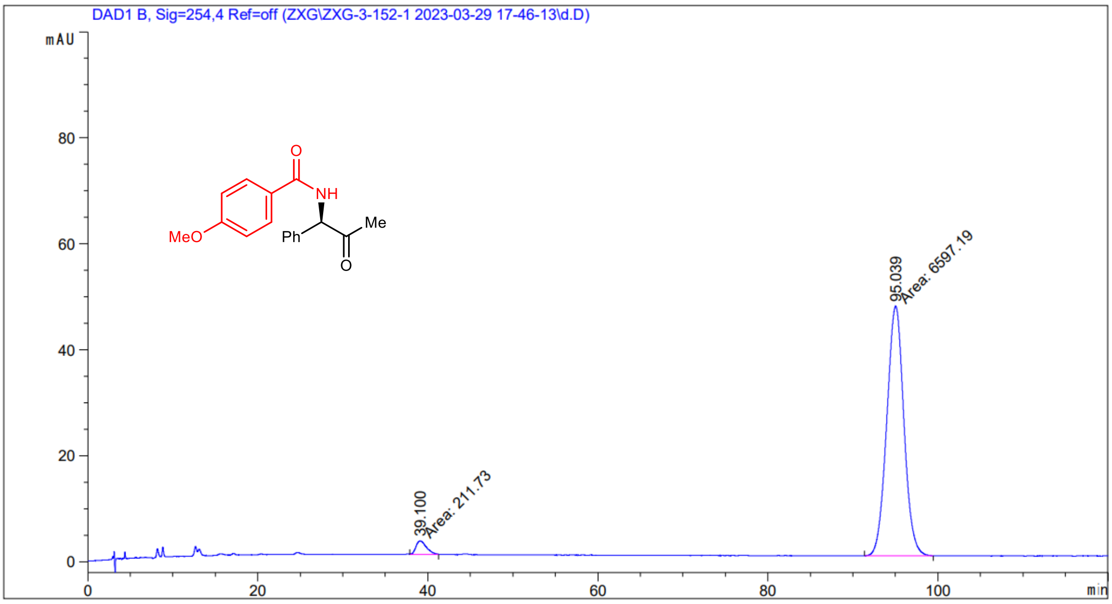

Peak #	RetTime [min]	Type	Width [min]	Area [mAU*s]	Height [mAU]	Area %
1	39.100	MM	1.4049	211.73024	2.51183	3.1096
2	95.039	MM	2.3350	6597.19482	47.08866	96.8904

Supplementary Figure 183. HPLC spectrum of (*R*)-N-(2-oxo-1-phenylpropyl)-4-(trifluoromethyl)benzamide (3r)

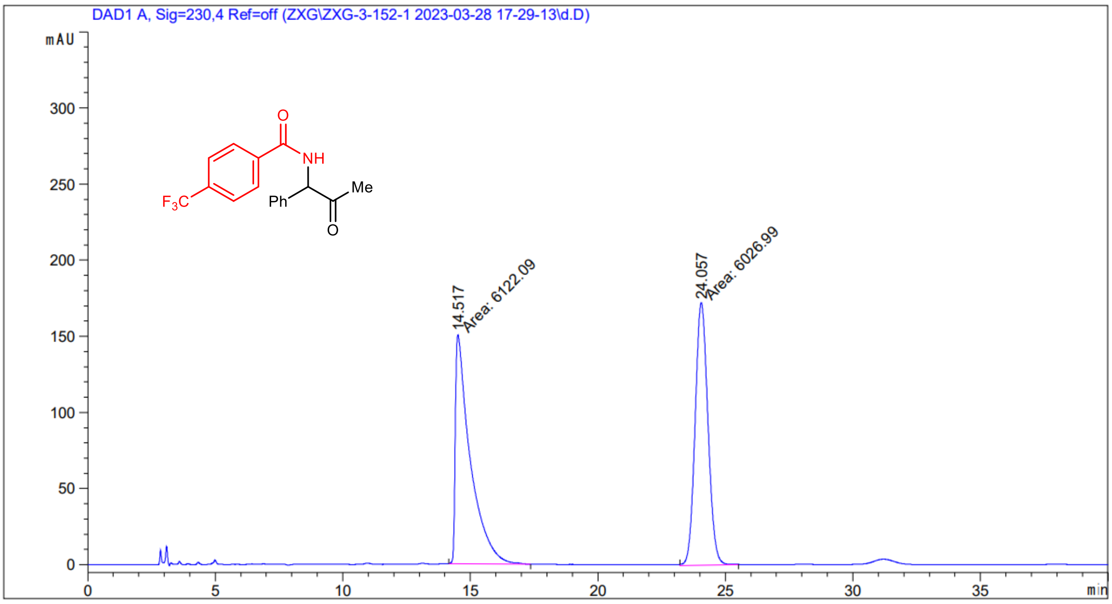

Peak #	RetTime [min]	Type	Width [min]	Area [mAU*s]	Height [mAU]	Area %
1	14.517	MM	0.6786	6122.08643	150.35742	50.3914
2	24.057	MM	0.5824	6026.99121	172.47578	49.6086

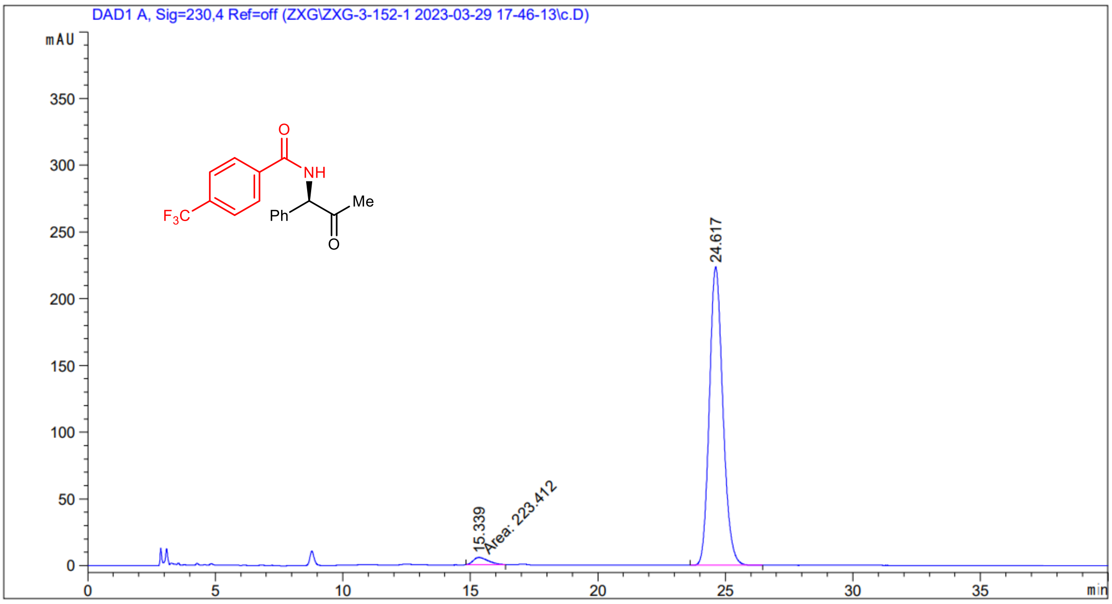

Peak #	RetTime [min]	Type	Width [min]	Area [mAU*s]	Height [mAU]	Area %
1	15.339	MM	0.6774	223.41168	5.49687	2.6390
2	24.617	BB	0.5663	8242.26465	223.45955	97.3610

Supplementary Figure 184. HPLC spectrum of (*R*)-4-fluoro-N-(2-oxo-1-phenylpropyl)benzamide (3s)

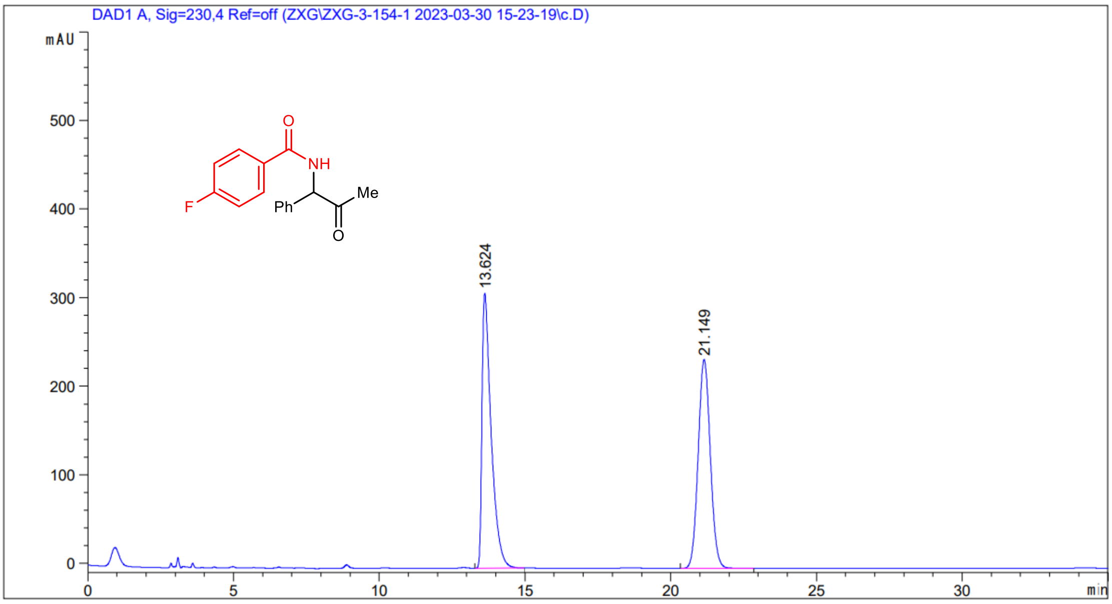

Peak #	RetTime [min]	Type	Width [min]	Area [mAU*s]	Height [mAU]	Area %
1	13.624	BB	0.3218	6805.72607	310.37527	49.9348
2	21.149	BB	0.4498	6823.50293	235.65918	50.0652

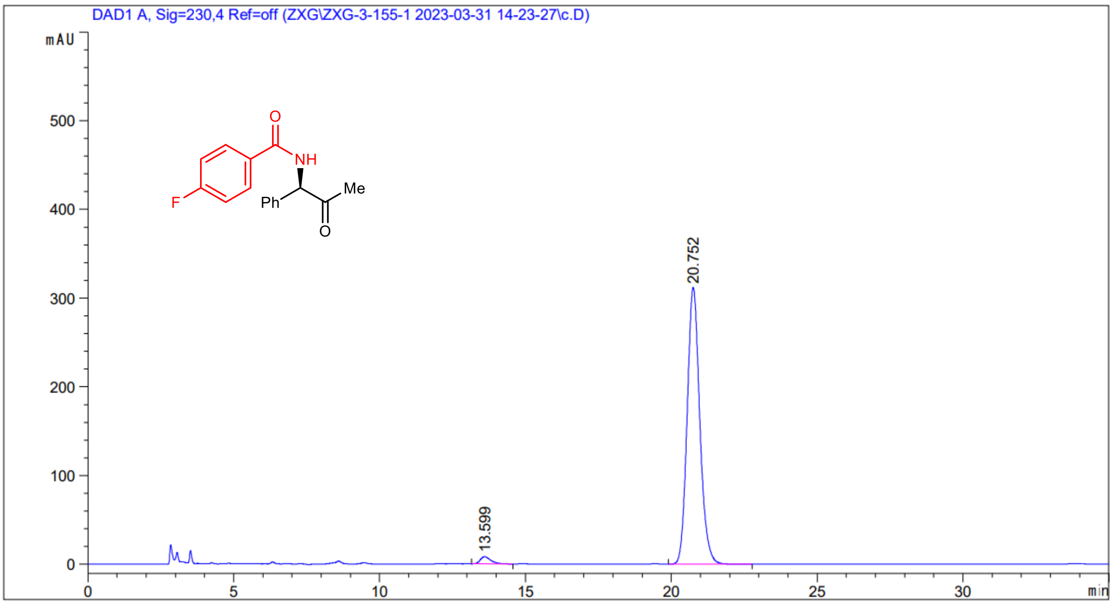

Peak #	RetTime [min]	Type	Width [min]	Area [mAU*s]	Height [mAU]	Area %
1	13.599	BB	0.3740	203.20302	8.11776	2.1271
2	20.752	BB	0.4600	9349.96484	311.65903	97.8729

Supplementary Figure 185. HPLC spectrum of (*R*)-4-chloro-N-(2-oxo-1-phenylpropyl)benzamide (3t)

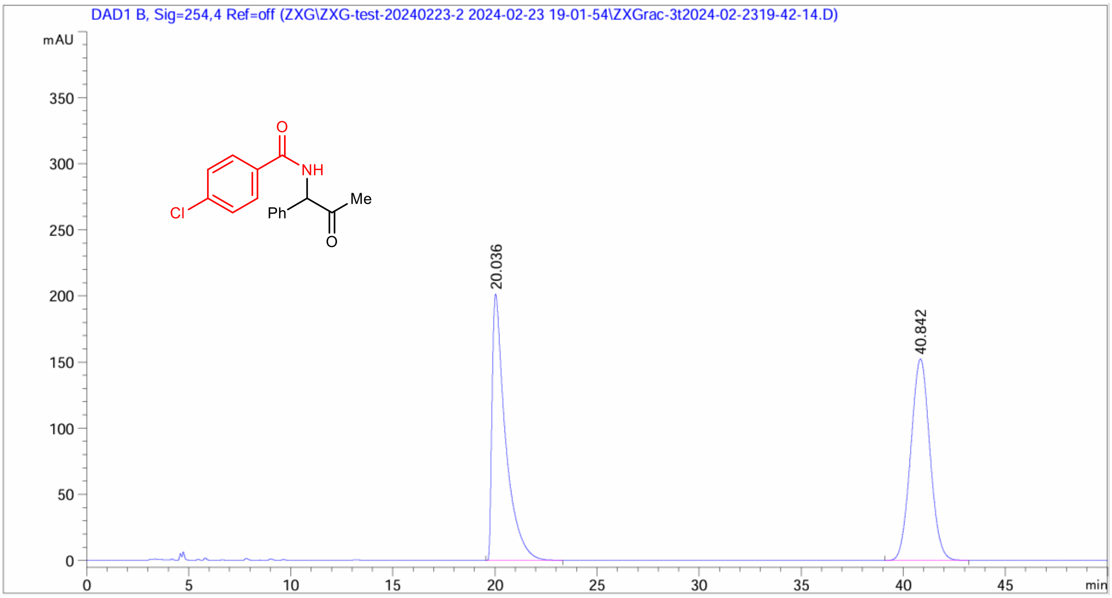

峰 #	保留时间 [min]	类型	峰宽 [min]	峰面积 [mAU*s]	峰高 [mAU]	峰面积 %
1	20.036	BB	0.6552	9170.40430	201.40721	47.9717
2	40.842	BB	1.0075	9945.88477	152.37071	52.0283

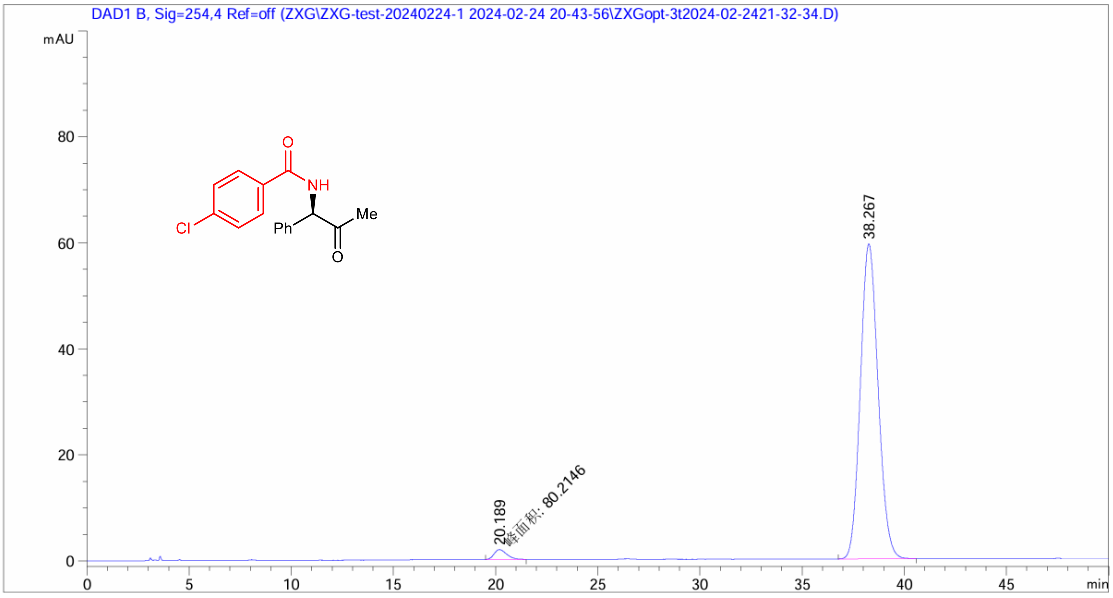

峰 #	保留时间 [min]	类型	峰宽 [min]	峰面积 [mAU*s]	峰高 [mAU]	峰面积 %
1	20.189	MM	0.7164	80.21456	1.86623	2.1885
2	38.267	BB	0.9263	3585.03589	59.38747	97.8115

Supplementary Figure 186. HPLC spectrum of (*R*)-4-bromo-N-(2-oxo-1-phenylpropyl)benzamide (3u)

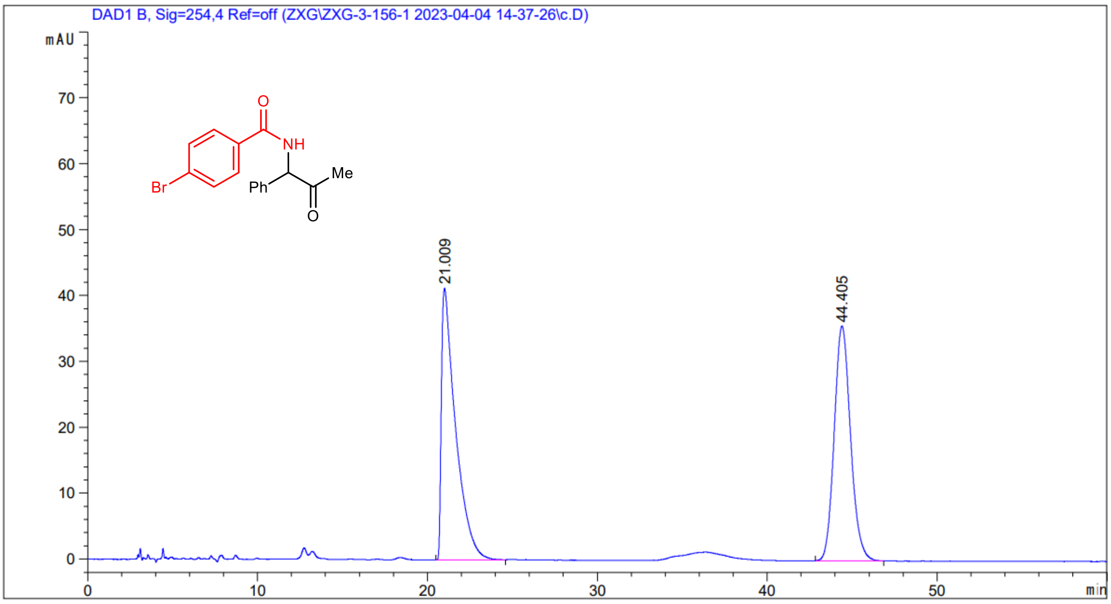

Peak #	RetTime [min]	Type	Width [min]	Area [mAU*s]	Height [mAU]	Area %
1	21.009	BB	0.8039	2386.82227	41.20100	50.0365
2	44.405	BB	1.0207	2383.33936	35.61402	49.9635

V

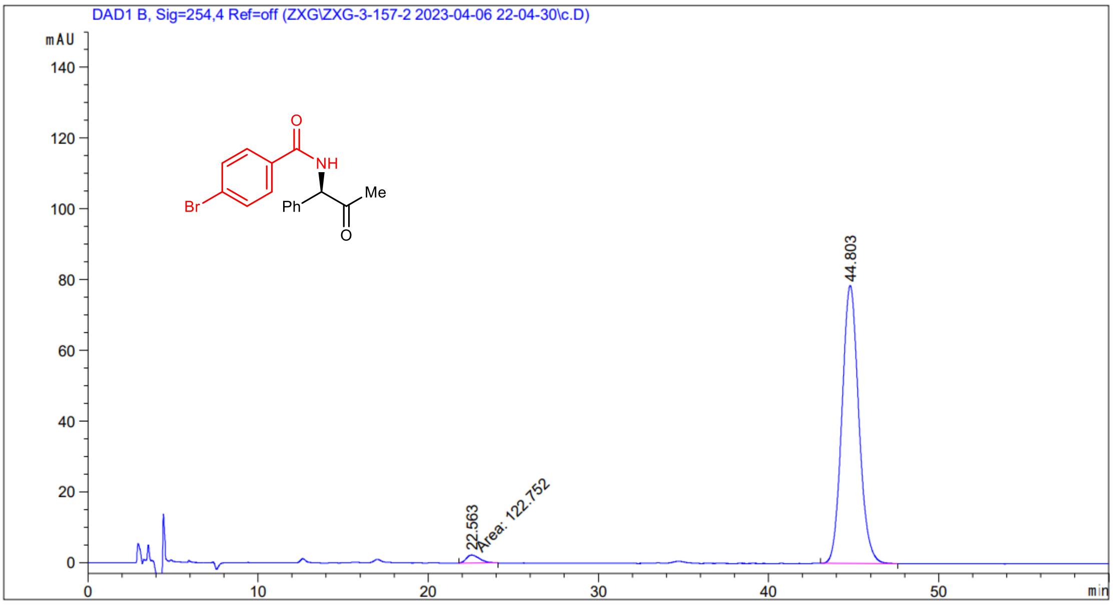

Peak #	RetTime [min]	Type	Width [min]	Area [mAU*s]	Height [mAU]	Area %
1	22.563	MM	0.9061	122.75229	2.25800	2.2221
2	44.803	BB	1.0622	5401.45508	78.55927	97.7779

Supplementary Figure 187. HPLC spectrum of (*R*)-4-iodo-N-(2-oxo-1-phenylpropyl)benzamide (3v)

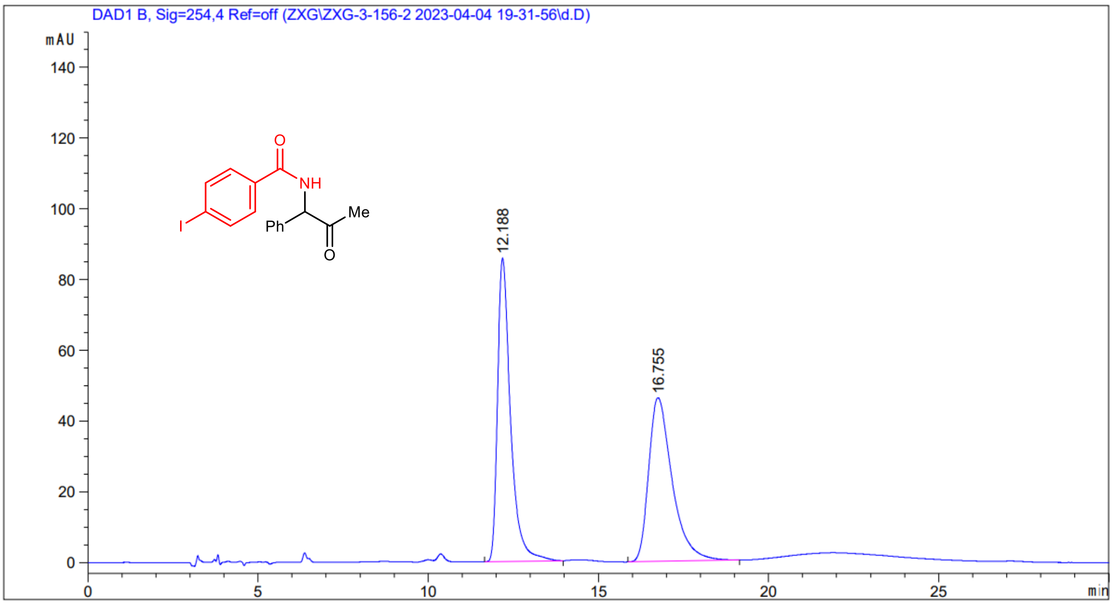

Peak #	RetTime [min]	Type	Width [min]	Area [mAU*s]	Height [mAU]	Area %
1	12.188	BB	0.3992	2272.48047	85.74079	50.5367
2	16.755	BB	0.7265	2224.21143	46.29952	49.4633

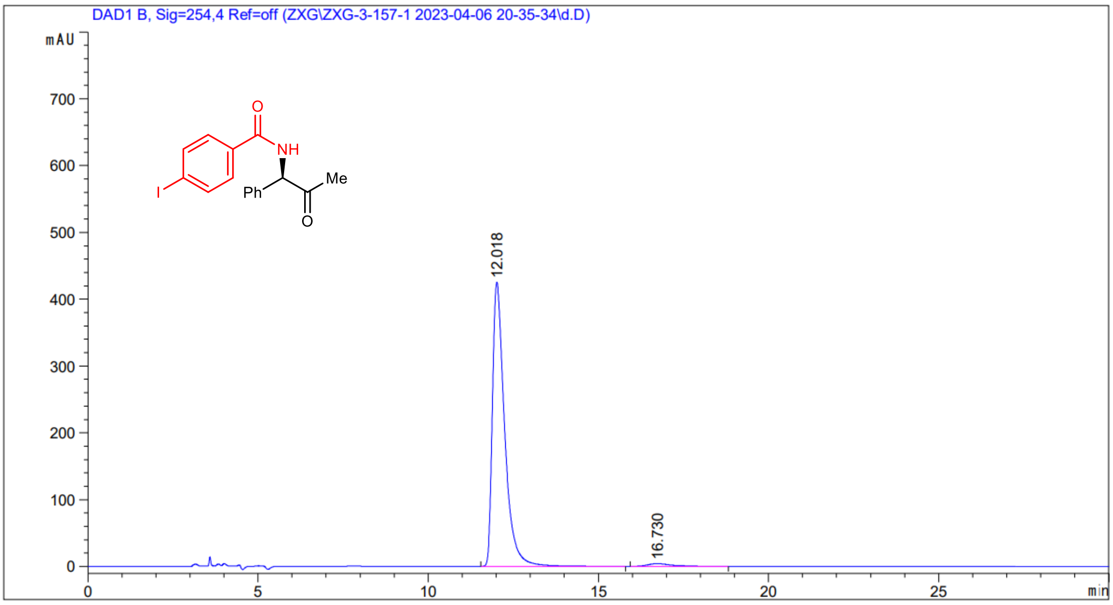

Peak #	RetTime [min]	Type	Width [min]	Area [mAU*s]	Height [mAU]	Area %
1	12.018	BB	0.3760	1.06400e4	425.18222	98.1088
2	16.730	BB	0.6719	205.10744	4.05634	1.8912

Supplementary Figure 188. HPLC spectrum of (*R*)-N-(2-oxo-1-phenylpropyl)-2-naphthamide (3w)

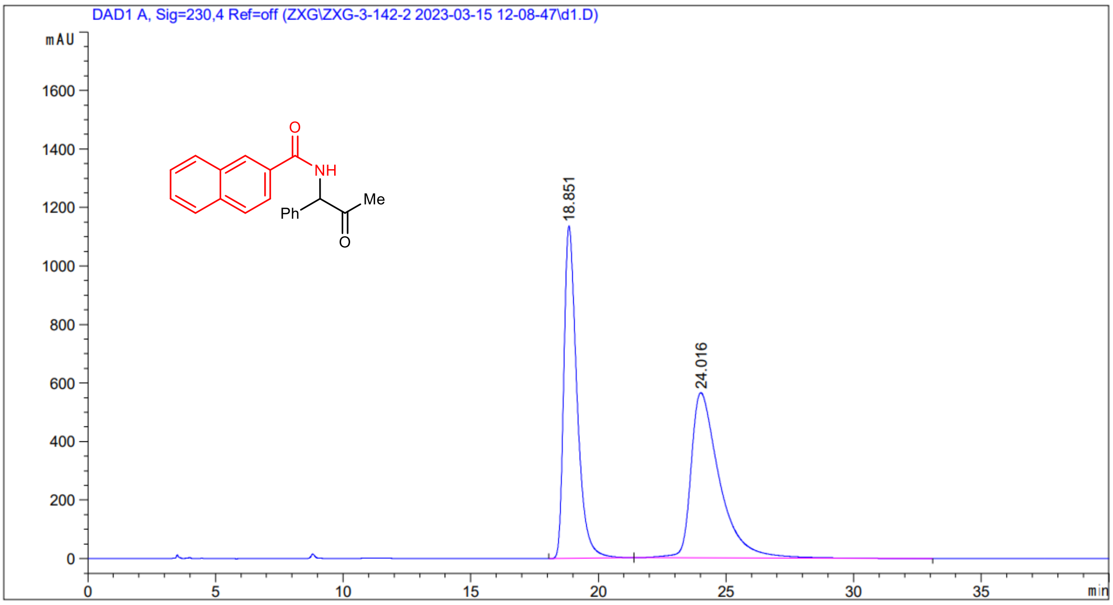

Peak #	RetTime [min]	Type	Width [min]	Area [mAU*s]	Height [mAU]	Area %
1	18.851	BB	0.5416	4.04649e4	1135.84619	48.0061
2	24.016	BB	1.1543	4.38263e4	564.18372	51.9939

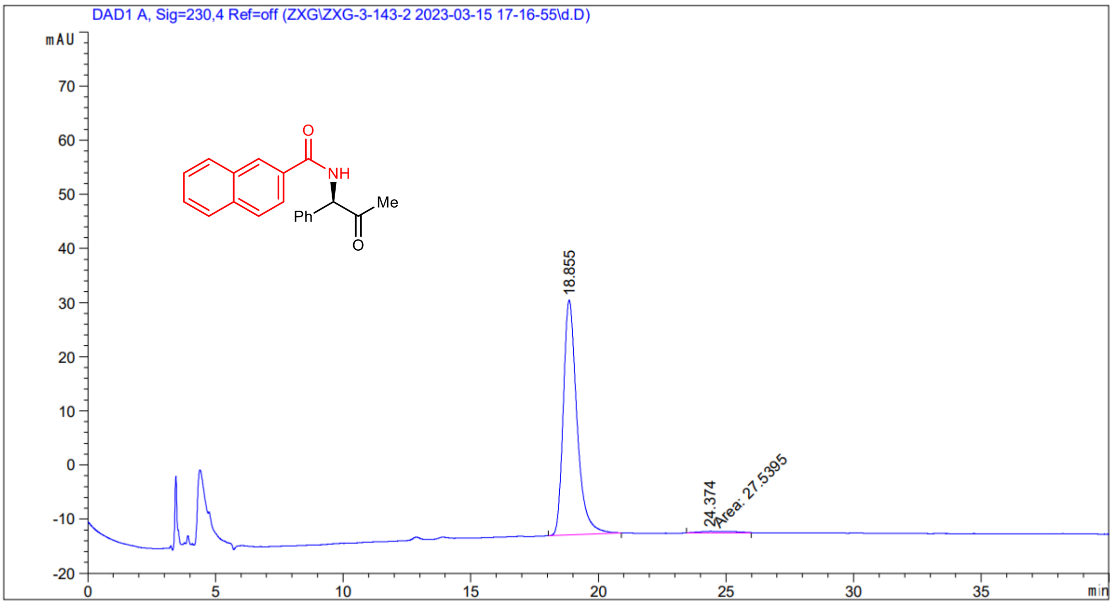

Peak #	RetTime [min]	Type	Width [min]	Area [mAU*s]	Height [mAU]	Area %
1	18.855	BB	0.5594	1612.48669	43.40464	98.3208
2	24.374	MM	1.5279	27.53948	3.00401e-1	1.6792

Supplementary Figure 189. HPLC spectrum of (*R*)-N-(2-oxo-1-phenylpropyl)furan-2-carboxamide (3x)

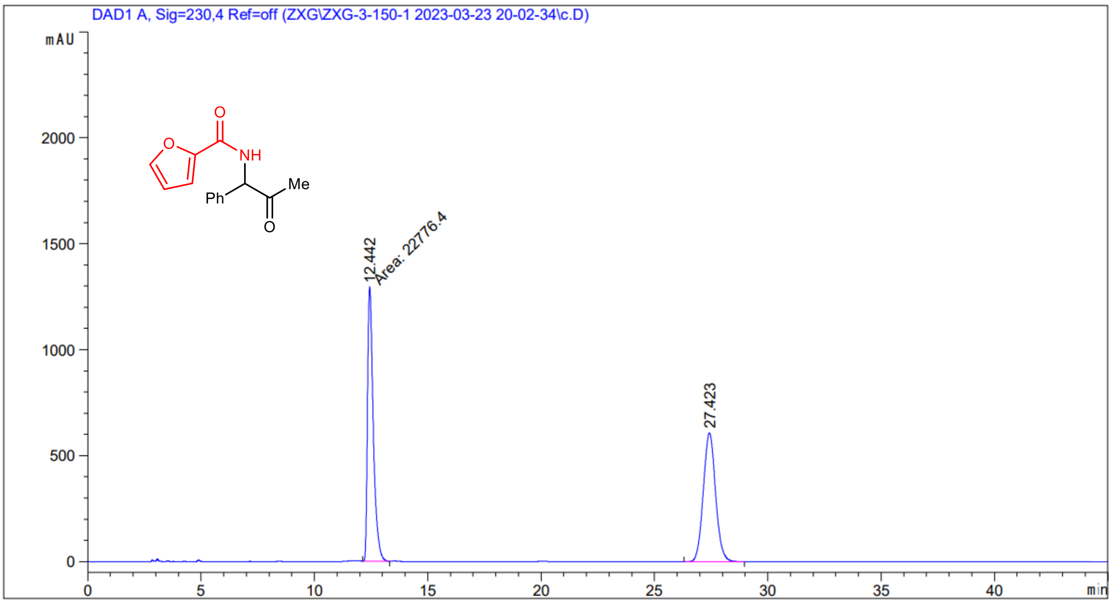

Peak #	RetTime [min]	Type	Width [min]	Area [mAU*s]	Height [mAU]	Area %
1	12.442	FM	0.2934	2.27764e4	1293.78345	49.9001
2	27.423	BB	0.5804	2.28675e4	608.55402	50.0999

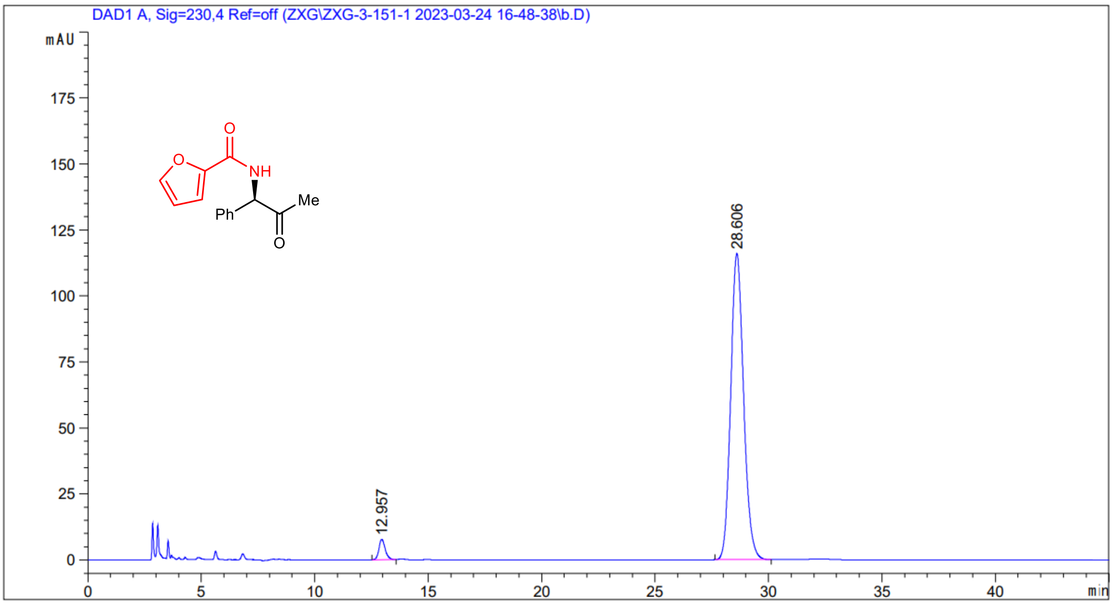

Peak #	RetTime [min]	Type	Width [min]	Area [mAU*s]	Height [mAU]	Area %
1	12.957	BB	0.2979	149.75870	7.73530	3.1671
2	28.606	BB	0.6089	4578.79199	115.90053	96.8329

Supplementary Figure 190. HPLC spectrum of (*R*)-N-(2-oxo-1-phenylpropyl)thiophene-2-carboxamide (3y)

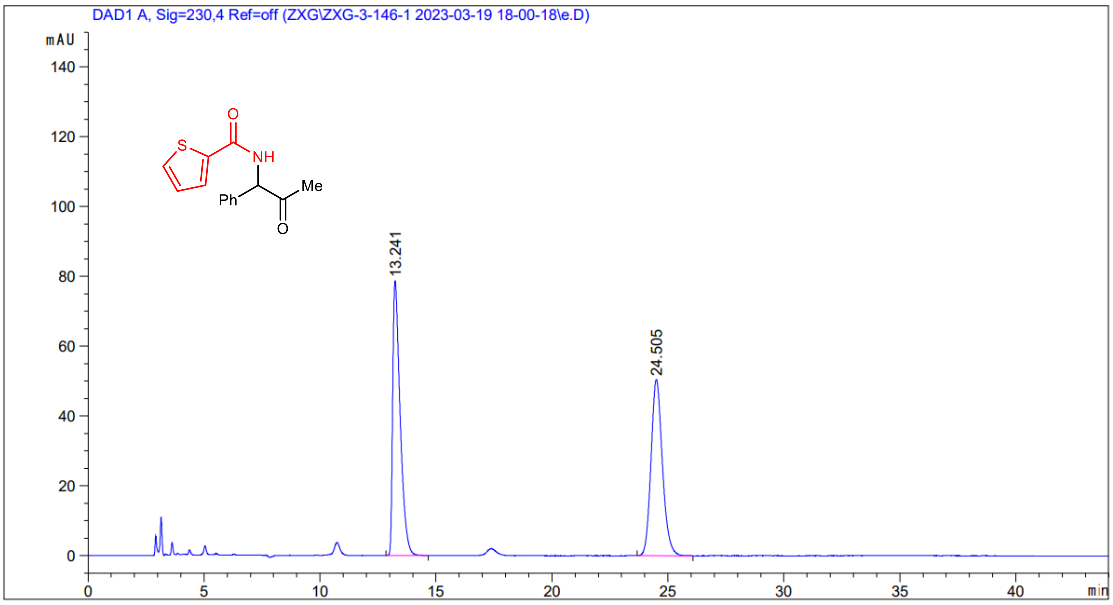

Peak #	RetTime [min]	Type	Width [min]	Area [mAU*s]	Height [mAU]	Area %
1	13.241	BB	0.3325	1742.98071	78.66444	50.0772
2	24.505	BB	0.5253	1737.60779	50.51386	49.9228

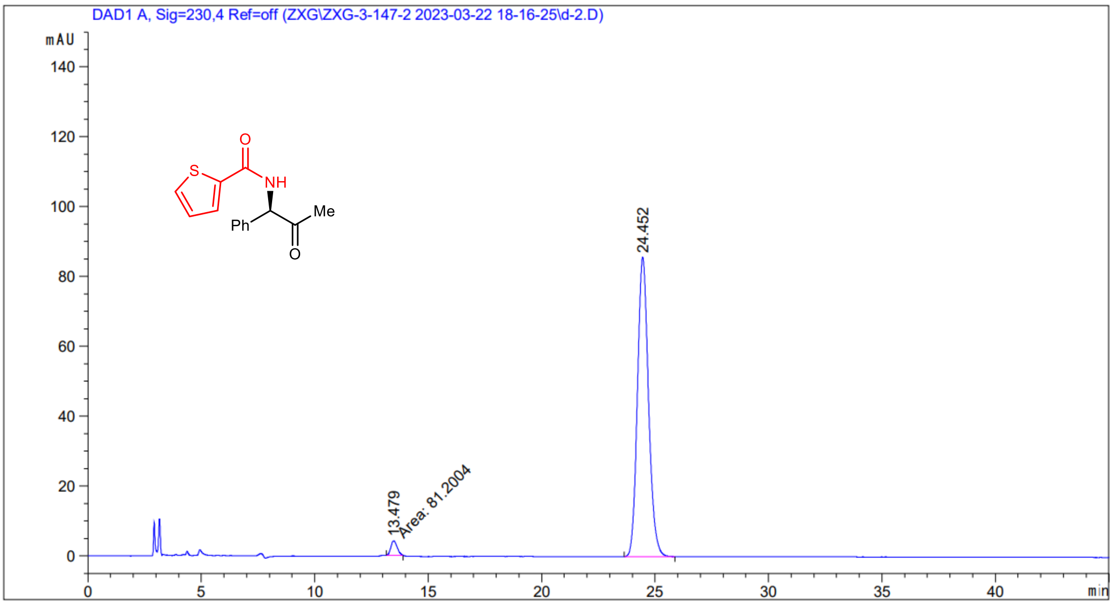

Peak #	RetTime [min]	Type	Width [min]	Area [mAU*s]	Height [mAU]	Area %
1	13.479	MM	0.3218	81.20042	4.20572	2.7119
2	24.452	BB	0.5242	2913.06104	85.77892	97.2881

Supplementary Figure 191. HPLC spectrum of (*R*)-N-(2-oxo-1-phenylpropyl)picolinamide (3z)

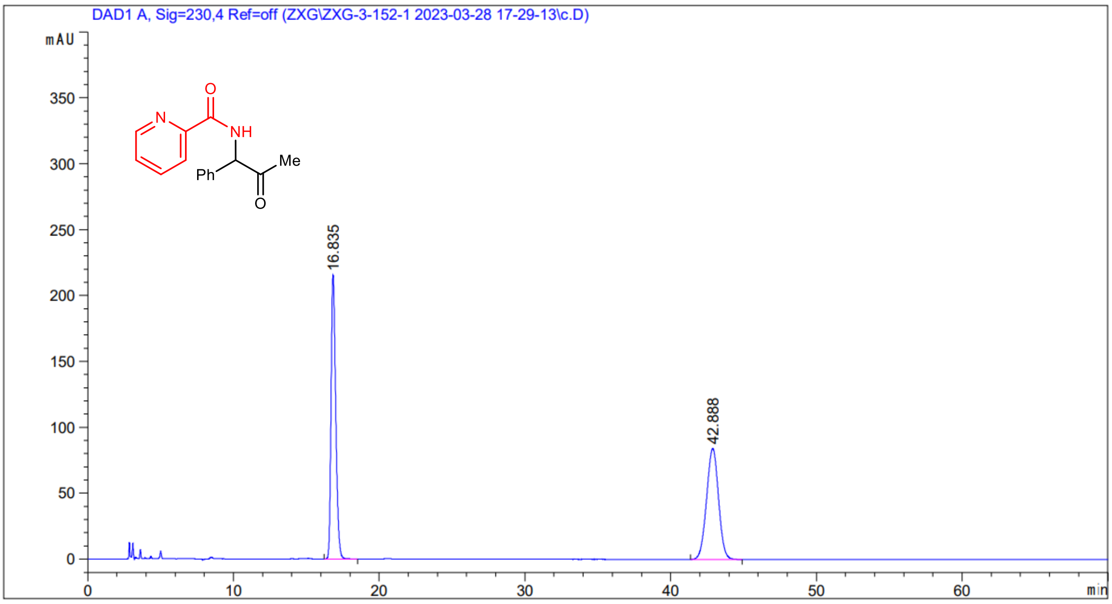

Peak #	RetTime [min]	Type	Width [min]	Area [mAU*s]	Height [mAU]	Area %
1	16.835	BB	0.3418	4797.44629	215.40486	50.0335
2	42.888	BB	0.8829	4791.01904	84.06163	49.9665

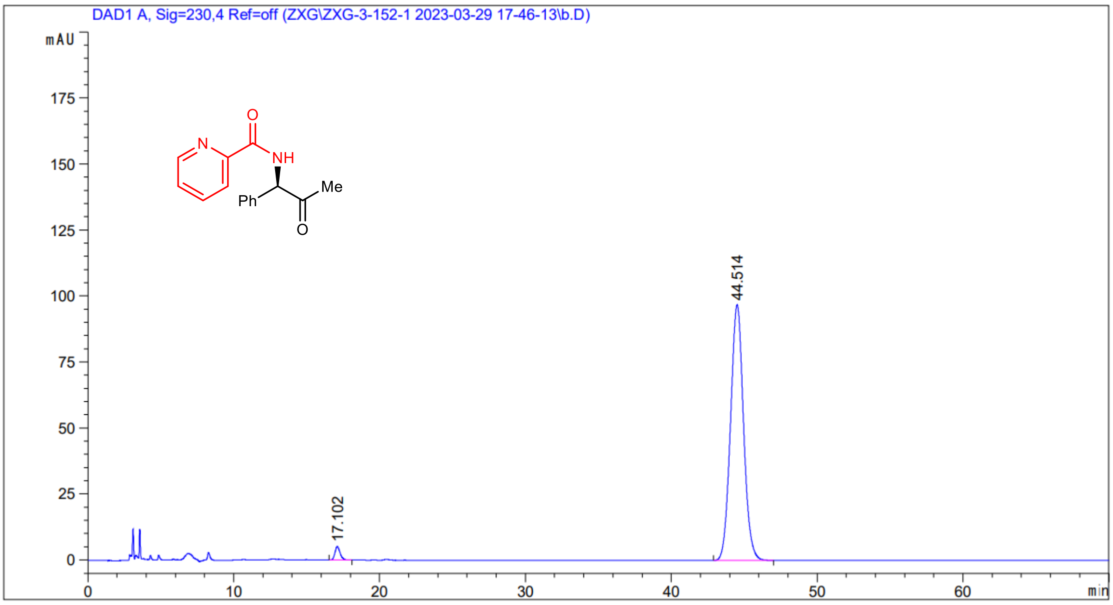

Peak #	RetTime [min]	Type	Width [min]	Area [mAU*s]	Height [mAU]	Area %
1	17.102	BB	0.3779	126.30787	5.11808	2.0624
2	44.514	BB	0.9528	5997.97705	96.80750	97.9376

Supplementary Figure 192. HPLC spectrum of (*R*)-N-(2-oxo-1-phenylpropyl)-1H-indole-2-carboxamide (3aa)

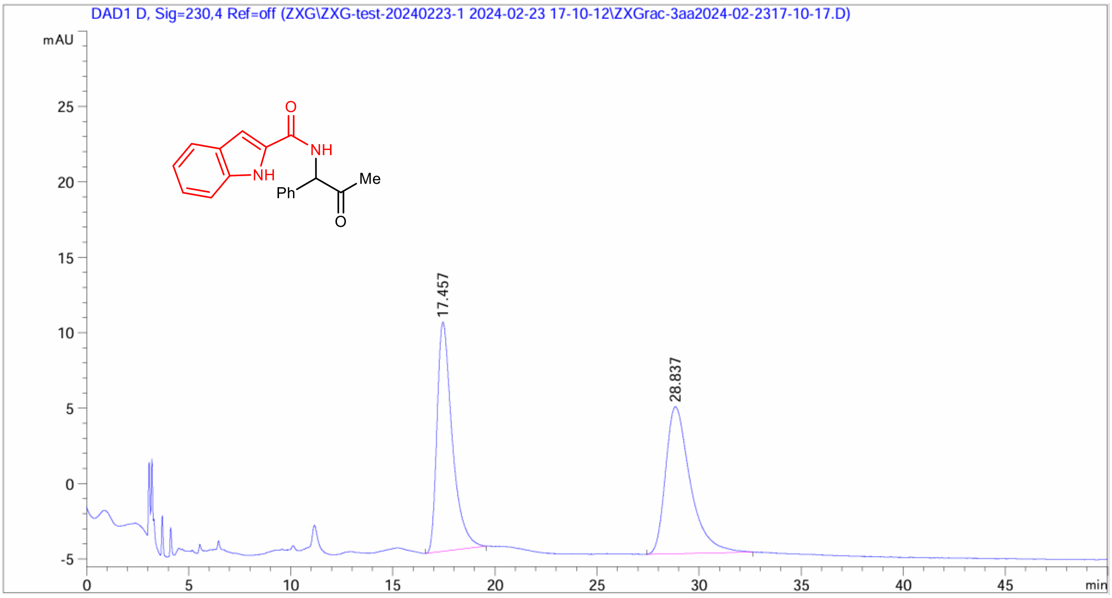

峰 #	保留时间 [min]	类型	峰宽 [min]	峰面积 [mAU*s]	峰高 [mAU]	峰面积 %
1	17.457	BB	0.7378	786.69843	15.20008	48.9927
2	28.837	BB	1.0032	819.04688	9.73851	51.0073

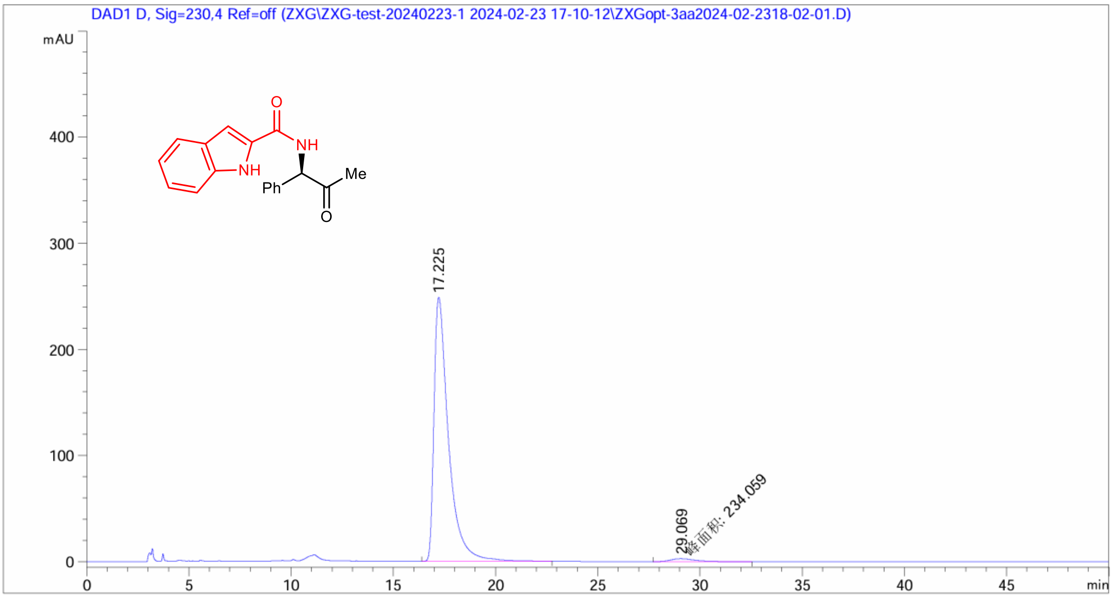

峰 #	保留时间 [min]	类型	峰宽 [min]	峰面积 [mAU*s]	峰高 [mAU]	峰面积 %
1	17.225	BB	0.7245	1.22529e4	248.83485	98.1256
2	29.069	MM	1.3896	234.05856	2.80734	1.8744

Supplementary Figure 193. HPLC spectrum of (*R*)-N-(2-oxo-1-phenylpropyl)acrylamide (3ab)

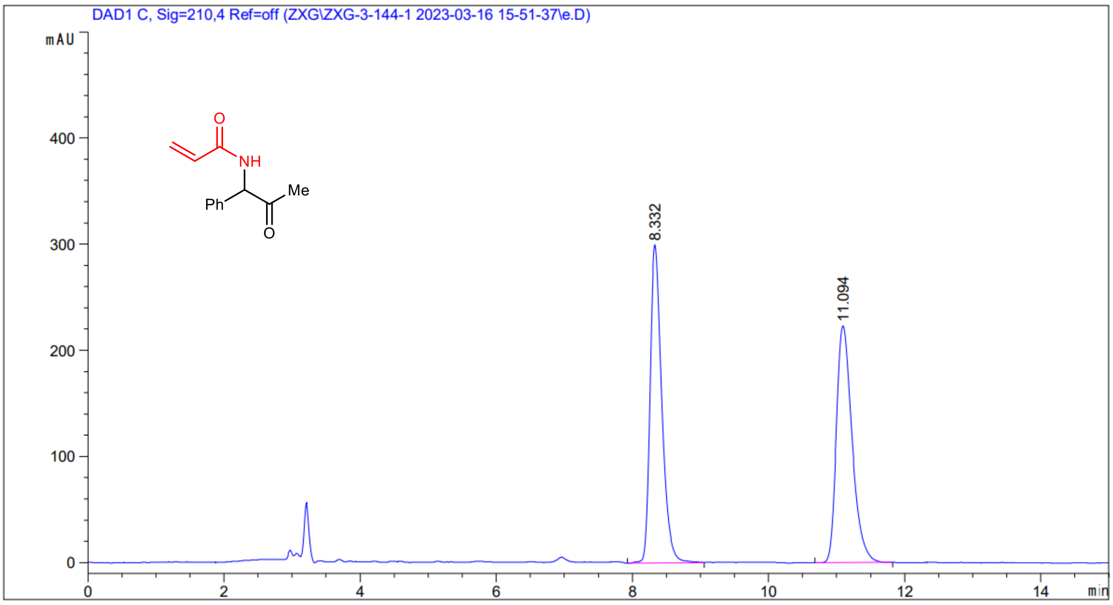

Peak #	RetTime [min]	Type	Width [min]	Area [mAU*s]	Height [mAU]	Area %
1	8.332	BB	0.1841	3596.01025	299.50638	50.2611
2	11.094	BB	0.2460	3558.64478	223.17900	49.7389

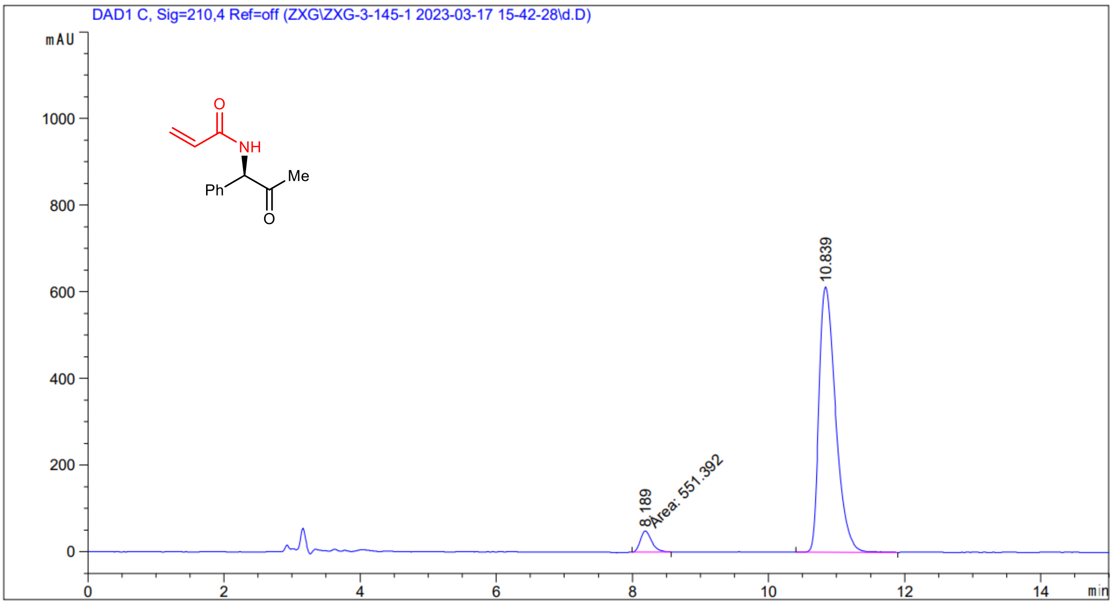

Peak #	RetTime [min]	Type	Width [min]	Area [mAU*s]	Height [mAU]	Area %
1	8.189	MM	0.1900	551.39160	48.37686	5.0498
2	10.839	BB	0.2616	1.03677e4	612.20251	94.9502

Supplementary Figure 194. HPLC spectrum of (*R*)-N-(2-oxo-1-phenylpropyl)-3-(trimethylsilyl)propiolamide (3ac)

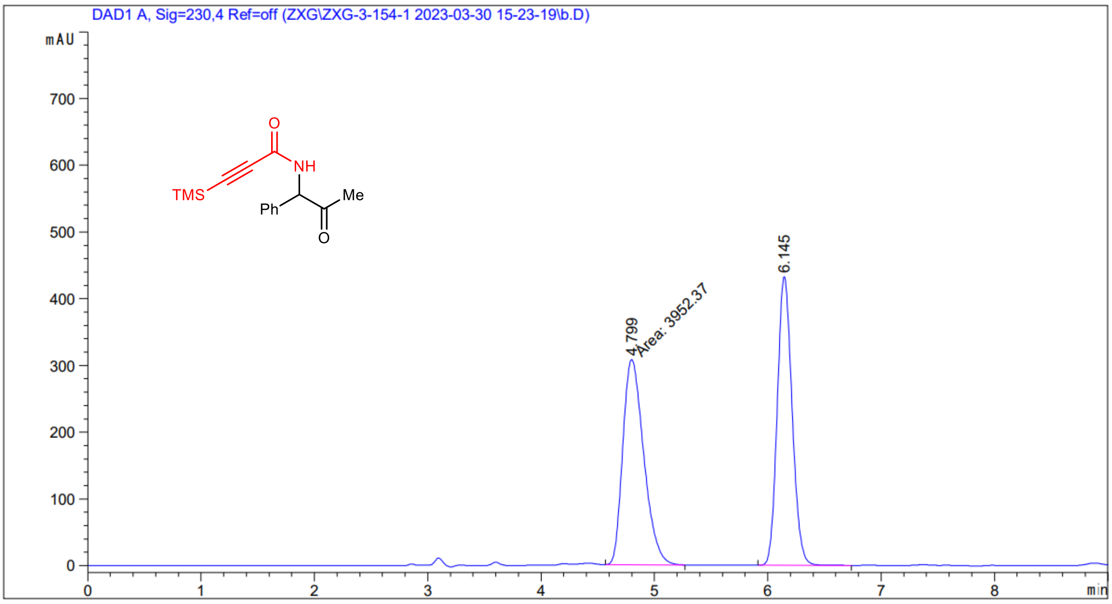

Peak #	RetTime [min]	Type	Width [min]	Area [mAU*s]	Height [mAU]	Area %
1	4.799	MM	0.2141	3952.37109	307.70825	50.1720
2	6.145	BB	0.1418	3925.26587	432.66833	49.8280

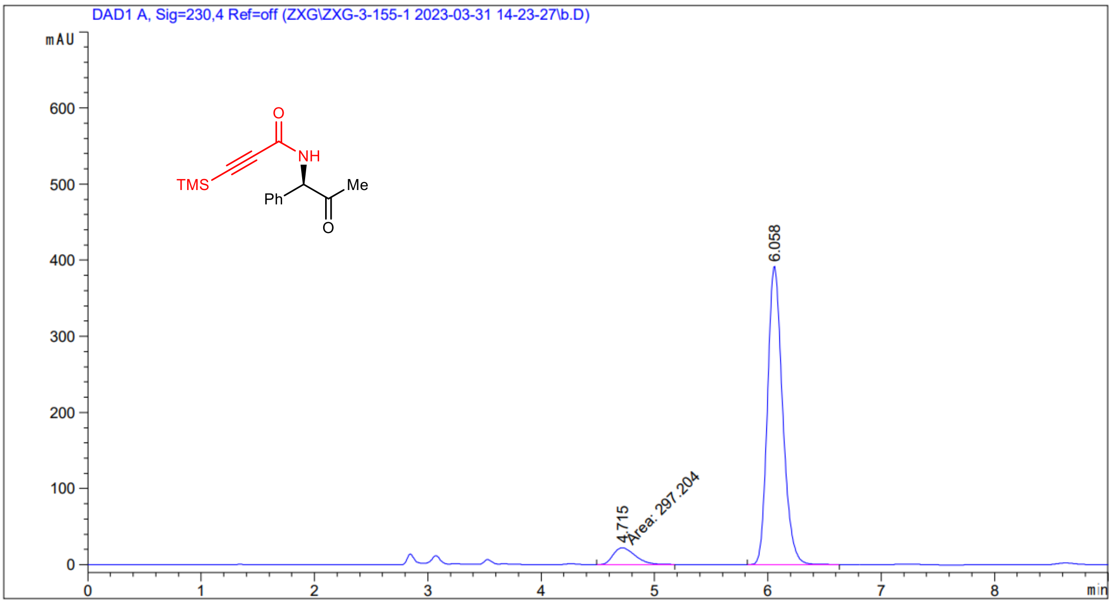

Peak #	RetTime [min]	Type	Width [min]	Area [mAU*s]	Height [mAU]	Area %
1	4.715	MM	0.2233	297.20422	22.18069	7.5963
2	6.058	BB	0.1436	3615.27808	391.80969	92.4037

Supplementary Figure 195. HPLC spectrum of (*R*)-N-(2-oxo-1-(*p*-tolyl)propyl)butyramide (4a)

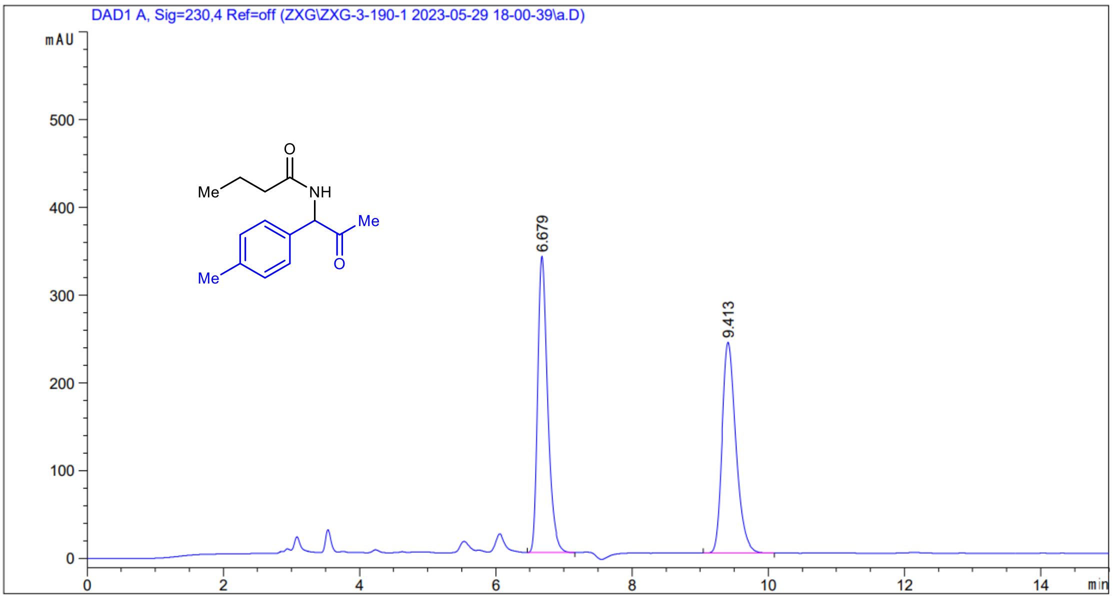

峰 #	保留时间 [min]	类型	峰宽 [min]	峰面积 [mAU*s]	峰高 [mAU]	峰面积 %
1	6.679	BB	0.1536	3400.16235	337.34381	50.0854
2	9.413	BB	0.2168	3388.56201	239.91452	49.9146

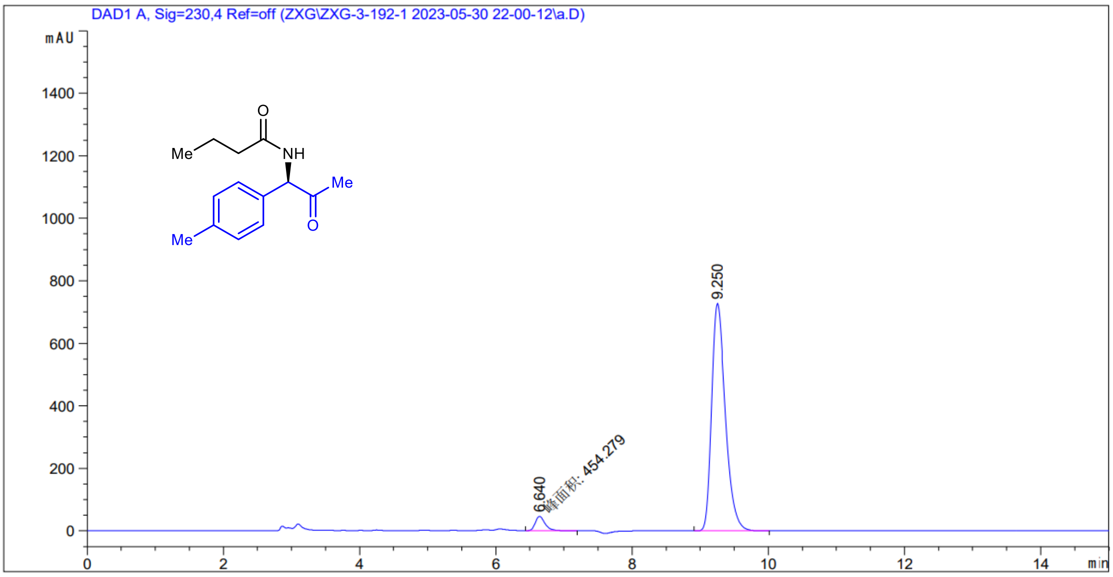

峰 #	保留时间 [min]	类型	峰宽 [min]	峰面积 [mAU*s]	峰高 [mAU]	峰面积 %
1	6.640	FM	0.1639	454.27917	46.19780	4.2559
2	9.250	BB	0.2160	1.02199e4	726.83948	95.7441

Supplementary Figure 196. HPLC spectrum of (*R*)-N-(2-oxo-1-(*m*-tolyl)propyl)butyramide (4b)

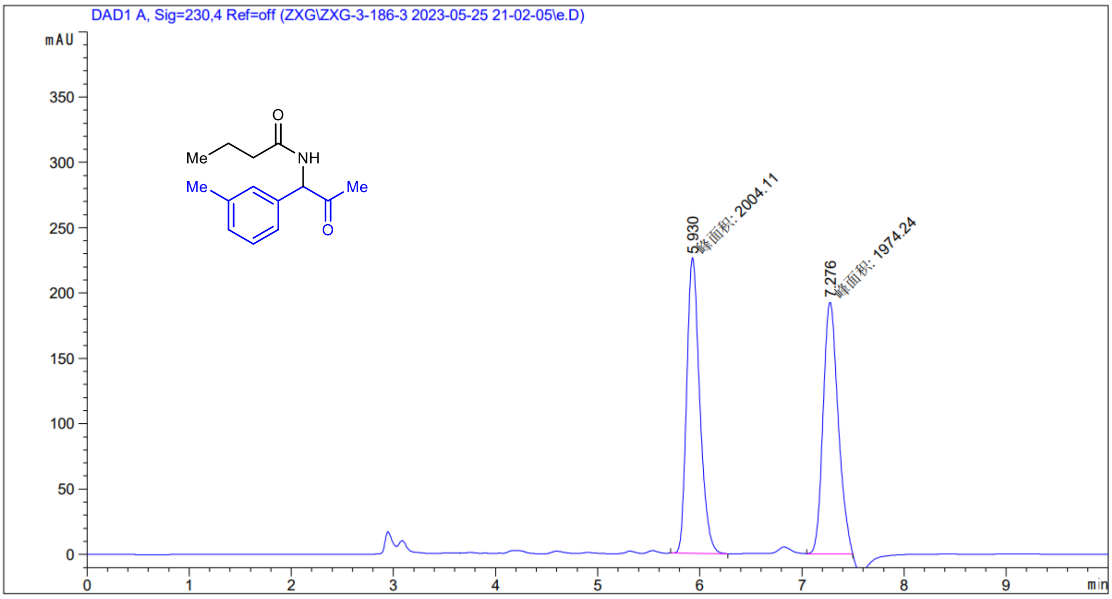

峰 #	保留时间 [min]	类型	峰宽 [min]	峰面积 [mAU*s]	峰高 [mAU]	峰面积 %
1	5.930	MM	0.1476	2004.11145	226.27333	50.3754
2	7.276	MF	0.1707	1974.24146	192.78877	49.6246

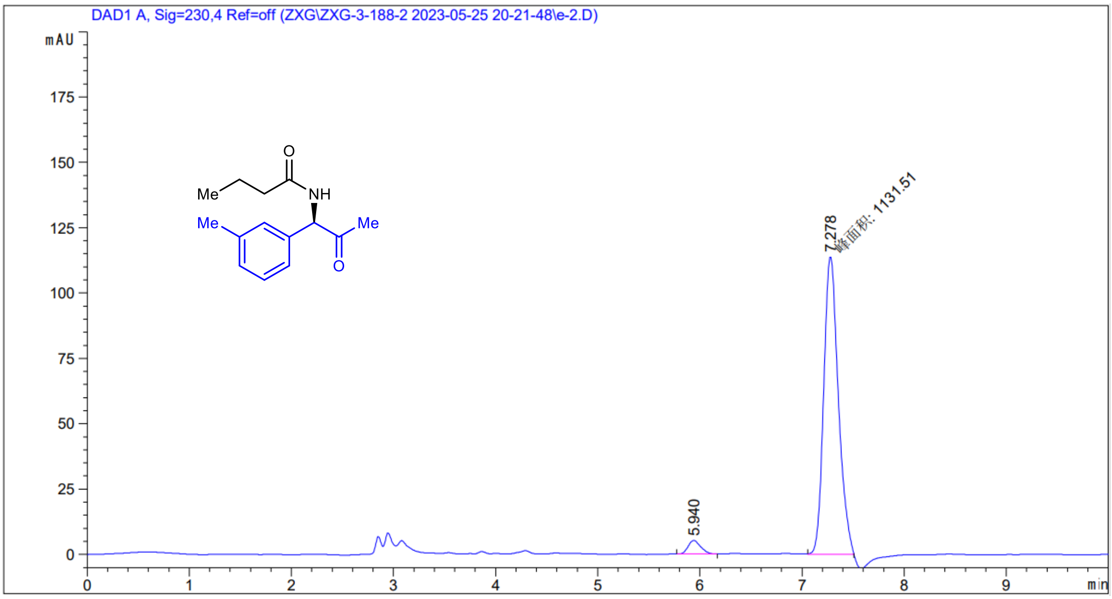

峰 #	保留时间 [min]	类型	峰宽 [min]	峰面积 [mAU*s]	峰高 [mAU]	峰面积 %
1	5.940	BB	0.1291	44.26665	5.21032	3.7649
2	7.278	MF	0.1654	1131.50769	114.03837	96.2351

Supplementary Figure 197. HPLC spectrum of (*R*)-N-(2-oxo-1-(4-(trifluoromethyl)phenyl)propyl)butyramide (4c)

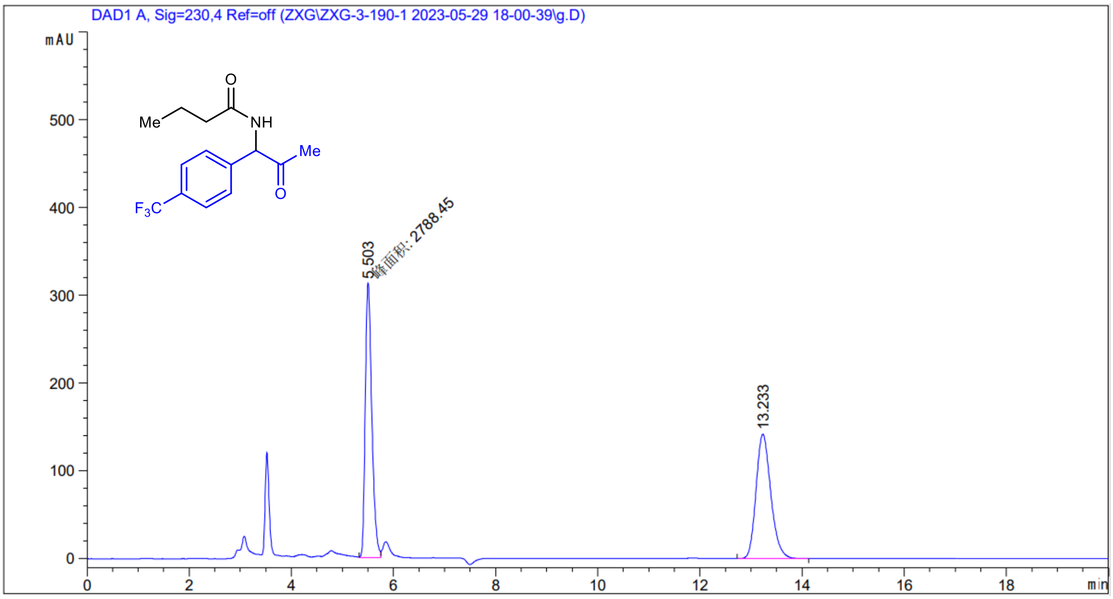

峰 #	保留时间 [min]	类型	峰宽 [min]	峰面积 [mAU*s]	峰高 [mAU]	峰面积 %
1	5.503	MF	0.1486	2788.44751	312.75696	49.5667
2	13.233	BB	0.3097	2837.19482	141.65559	50.4333

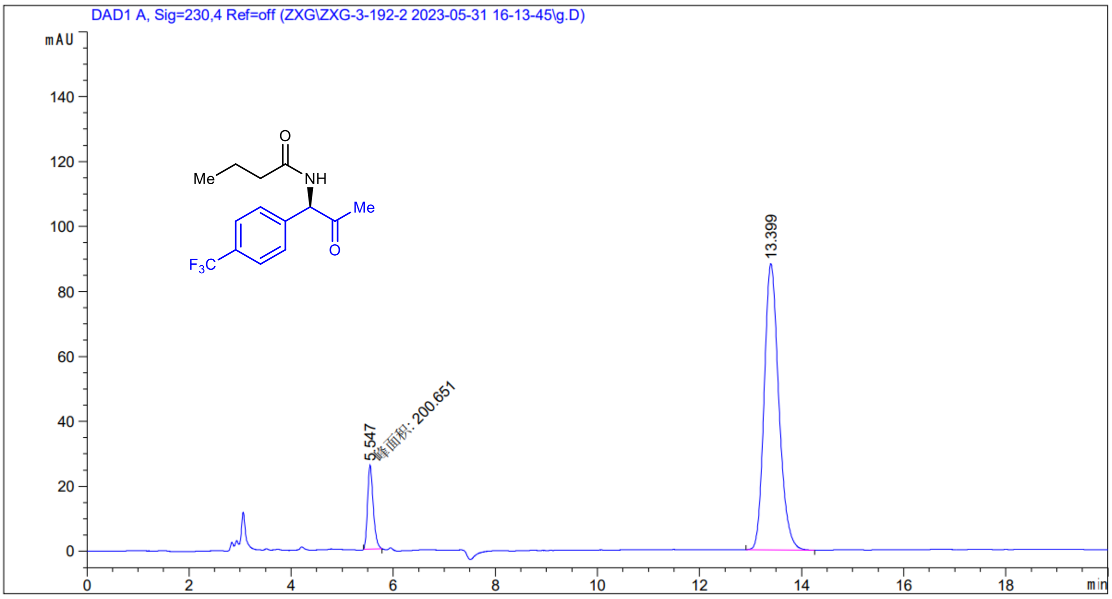

峰 #	保留时间 [min]	类型	峰宽 [min]	峰面积 [mAU*s]	峰高 [mAU]	峰面积 %
1	5.547	MM	0.1294	200.65062	25.84324	10.2630
2	13.399	BB	0.3063	1754.44287	88.11241	89.7370

Supplementary Figure 198. HPLC spectrum of (*R*)-N-(1-(3-methoxyphenyl)-2-oxopropyl)butyramide (4d)

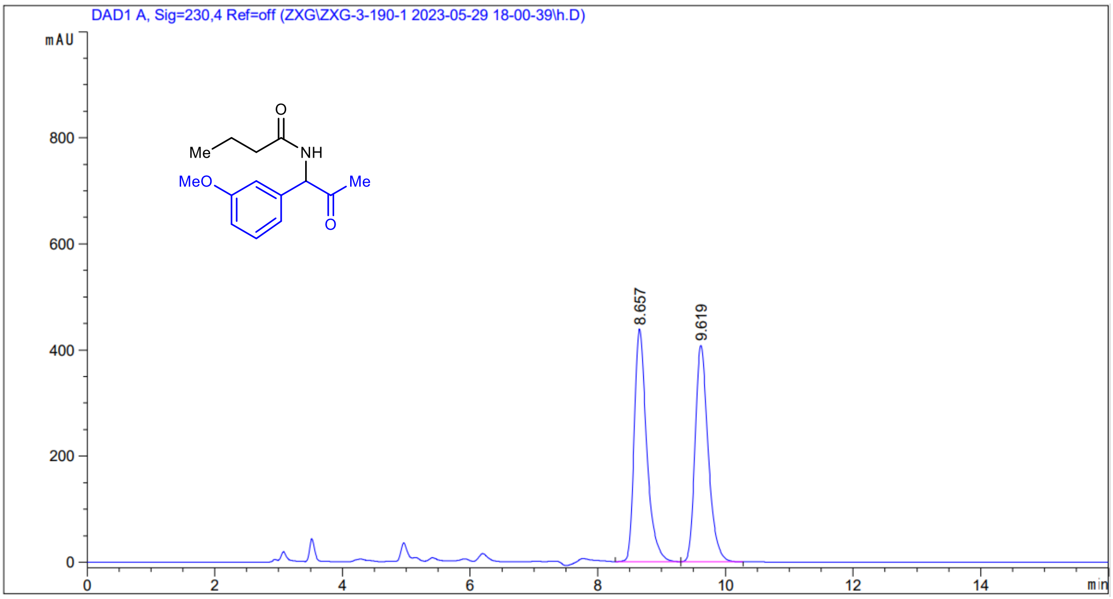

峰 #	保留时间 [min]	类型	峰宽 [min]	峰面积 [mAU*s]	峰高 [mAU]	峰面积 %
1	8.657	BB	0.2061	5948.57959	438.93573	50.6735
2	9.619	BB	0.2156	5790.45459	407.86102	49.3265

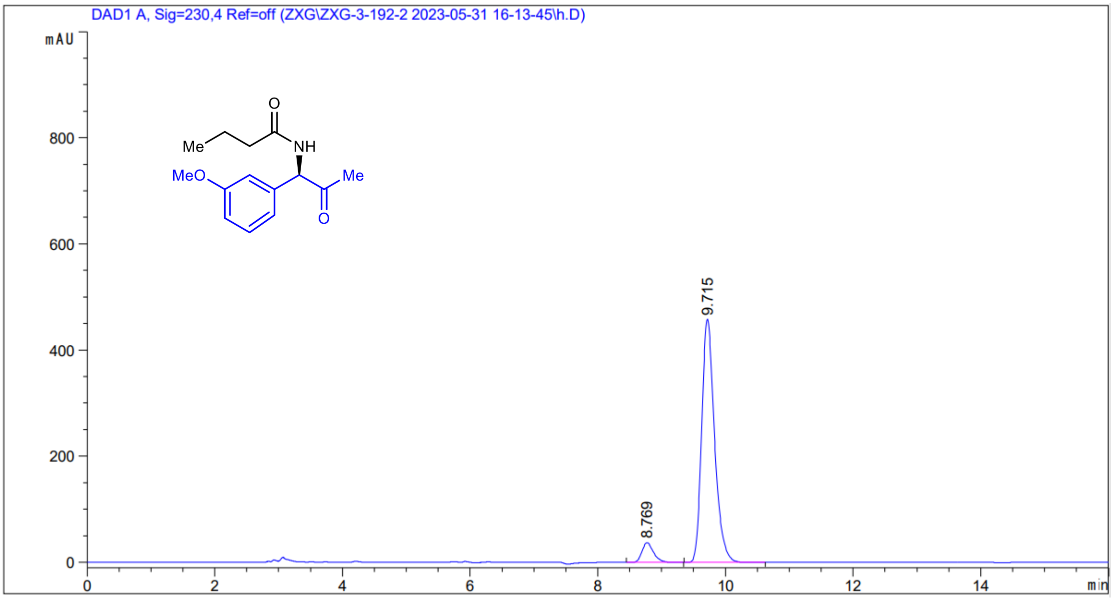

峰 #	保留时间 [min]	类型	峰宽 [min]	峰面积 [mAU*s]	峰高 [mAU]	峰面积 %
1	8.769	BB	0.1923	473.83728	37.28056	6.8565
2	9.715	BB	0.2139	6436.95850	458.29718	93.1435

Supplementary Figure 199. HPLC spectrum of (*R*)-N-(1-(2-fluorophenyl)-2-oxopropyl)butyramide (4e)

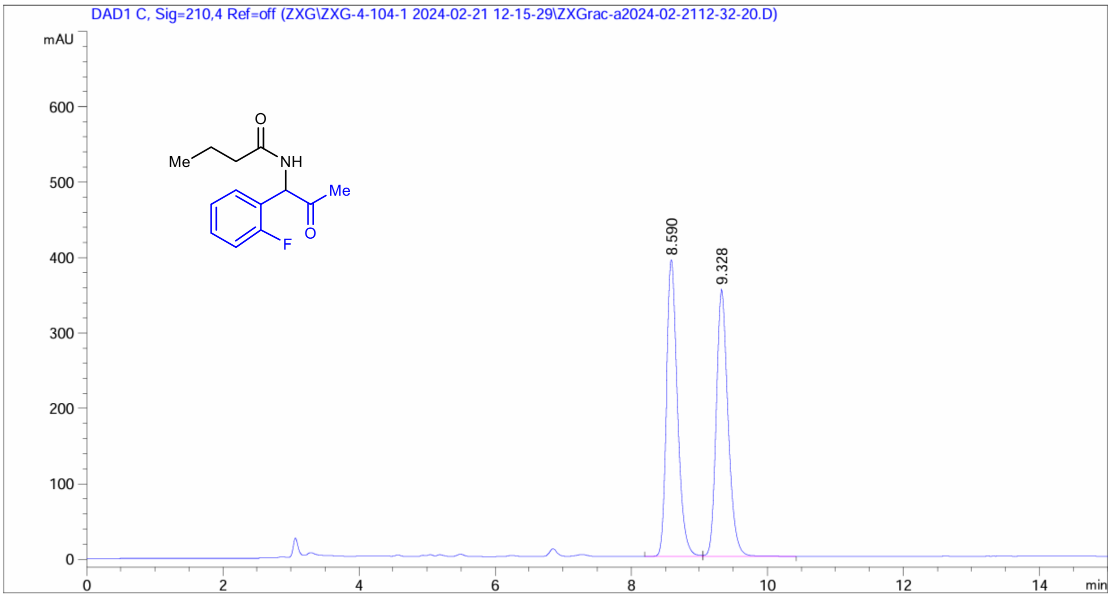

峰 #	保留时间 [min]	类型	峰宽 [min]	峰面积 [mAU*s]	峰高 [mAU]	峰面积 %
1	8.590	BV	0.1709	4414.60938	393.50937	50.4395
2	9.328	VB	0.1889	4337.68408	354.10028	49.5605

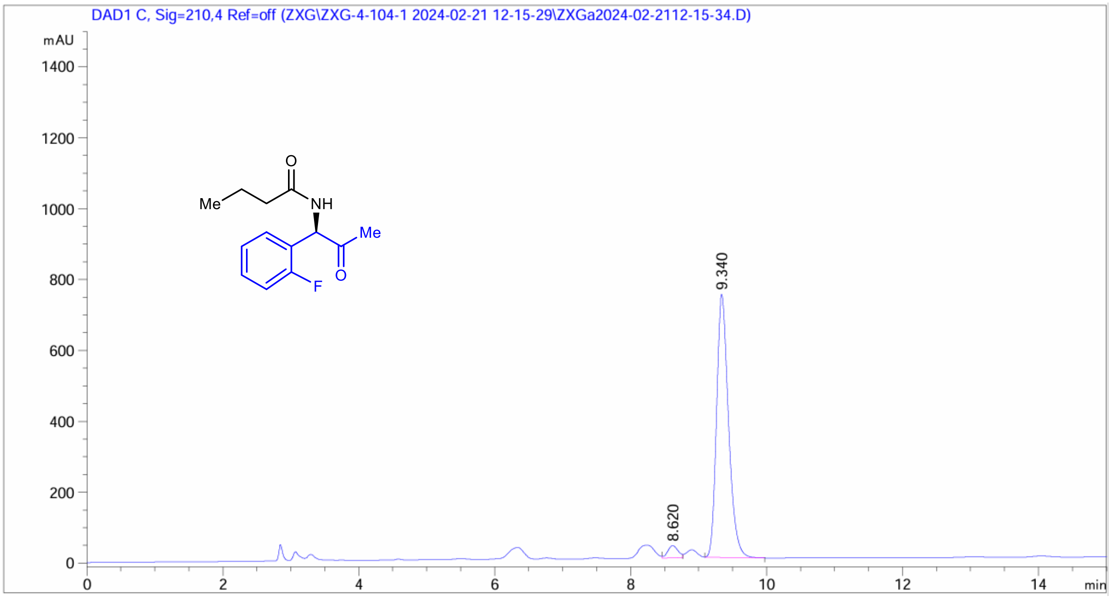

峰 #	保留时间 [min]	类型	峰宽 [min]	峰面积 [mAU*s]	峰高 [mAU]	峰面积 %
1	8.620	VV	0.1591	356.85468	34.36865	3.7440
2	9.340	BB	0.1902	9174.55078	742.31647	96.2560

Supplementary Figure 200. HPLC spectrum of (*R*)-N-(1-(4-fluorophenyl)-2-oxopropyl)butyramide (4f)

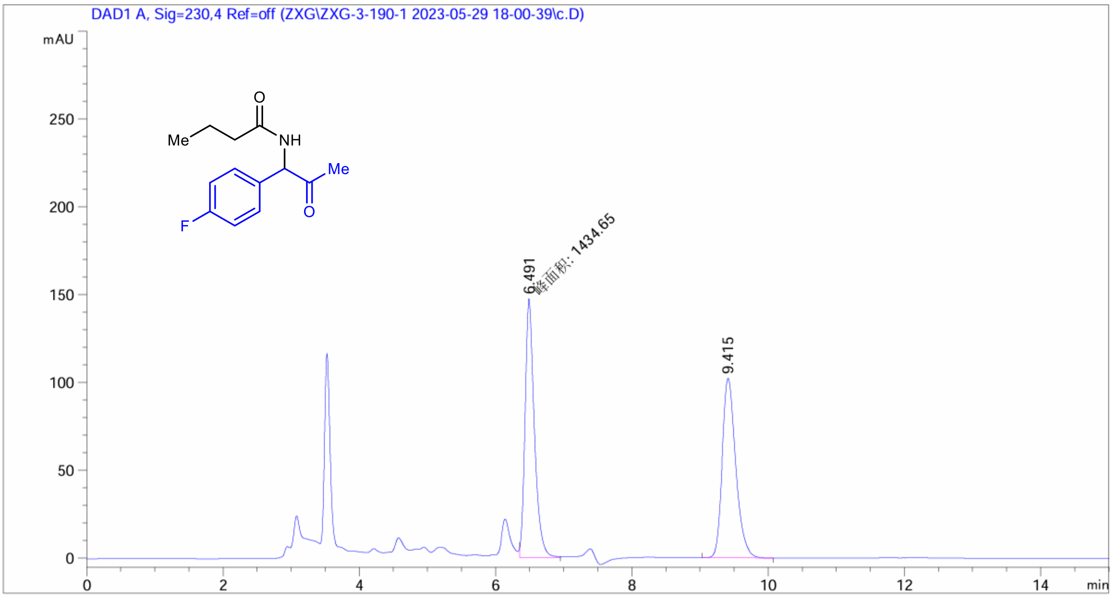

峰 #	保留时间 [min]	类型	峰宽 [min]	峰面积 [mAU*s]	峰高 [mAU]	峰面积 %
1	6.491	FM	0.1621	1434.64868	147.50188	50.4152
2	9.415	BB	0.2112	1411.02075	102.12785	49.5848

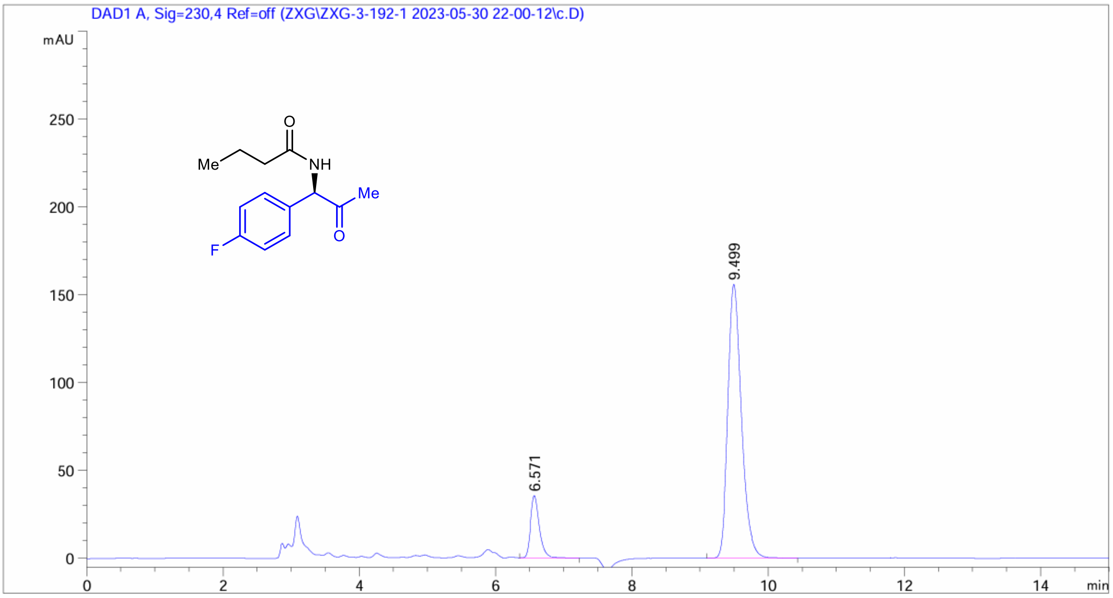

峰 #	保留时间 [min]	类型	峰宽 [min]	峰面积 [mAU*s]	峰高 [mAU]	峰面积 %
1	6.571	BB	0.1377	321.78268	35.51303	13.0265
2	9.499	BB	0.2110	2148.43945	155.70192	86.9735

Supplementary Figure 201. HPLC spectrum of (*R*)-N-(1-(4-chlorophenyl)-2-oxopropyl)butyramide (4g)

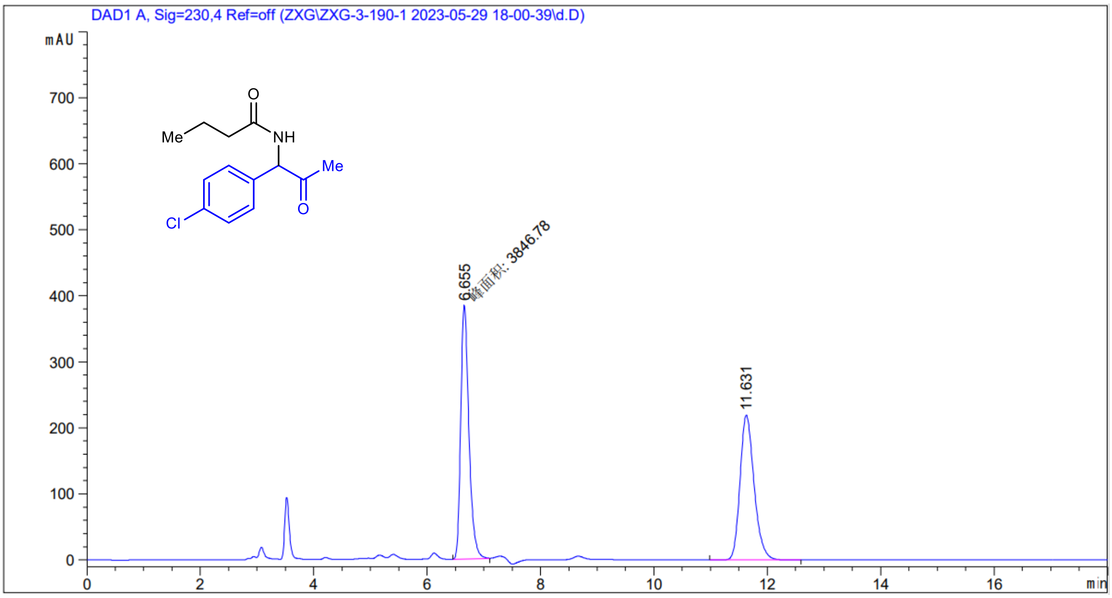

峰 #	保留时间 [min]	类型	峰宽 [min]	峰面积 [mAU*s]	峰高 [mAU]	峰面积 %
1	6.655	MM	0.1665	3846.78491	385.10263	50.4486
2	11.631	BB	0.2651	3778.37256	219.14998	49.5514

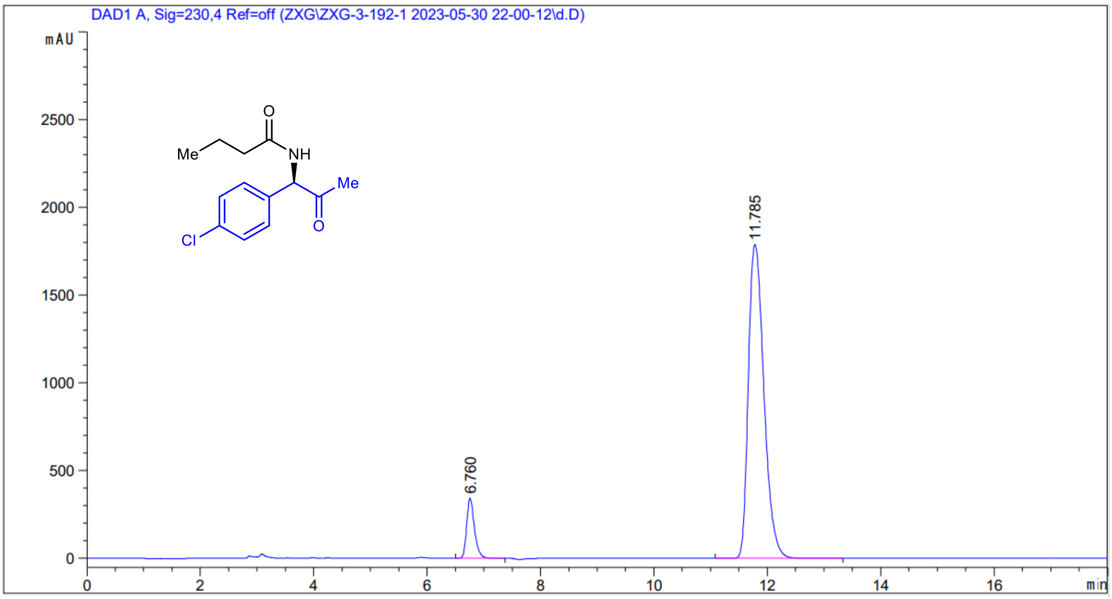

峰 #	保留时间 [min]	类型	峰宽 [min]	峰面积 [mAU*s]	峰高 [mAU]	峰面积 %
1	6.760	BB	0.1449	3257.15112	342.50375	8.7396
2	11.785	BB	0.2981	3.40115e4	1786.81641	91.2604

Supplementary Figure 202. HPLC spectrum of (*R*)-N-(1-(4-bromophenyl)-2-oxopropyl)butyramide (4h)

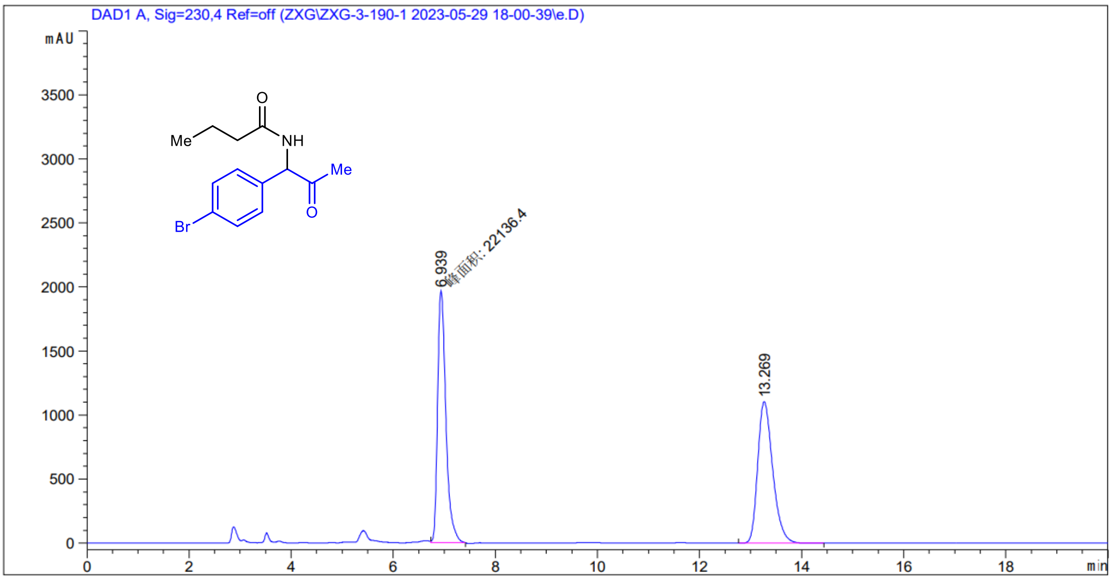

峰 #	保留时间 [min]	类型	峰宽 [min]	峰面积 [mAU*s]	峰高 [mAU]	峰面积 %
1	6.939	FM	0.1876	2.21364e4	1966.71216	49.5664
2	13.269	BB	0.3138	2.25237e4	1105.09106	50.4336

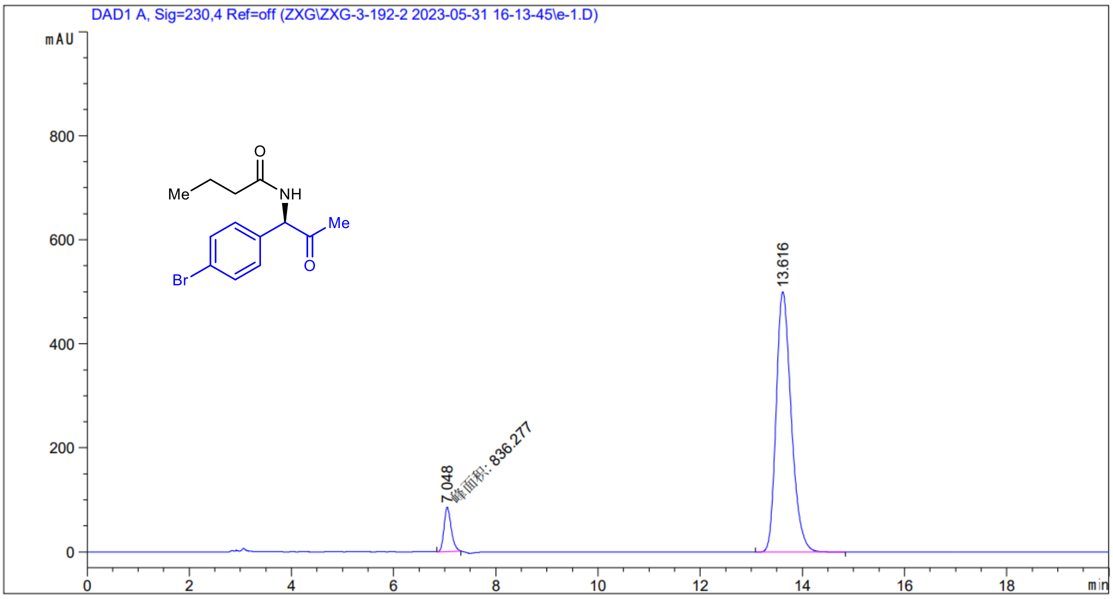

峰 #	保留时间 [min]	类型	峰宽 [min]	峰面积 [mAU*s]	峰高 [mAU]	峰面积 %
1	7.048	MM	0.1632	836.27655	85.39879	7.5451
2	13.616	BB	0.3150	1.02475e4	500.17133	92.4549

Supplementary Figure 203. HPLC spectrum of (*R*)-N-(1-(4-iodophenyl)-2-oxopropyl)butyramide (4i)

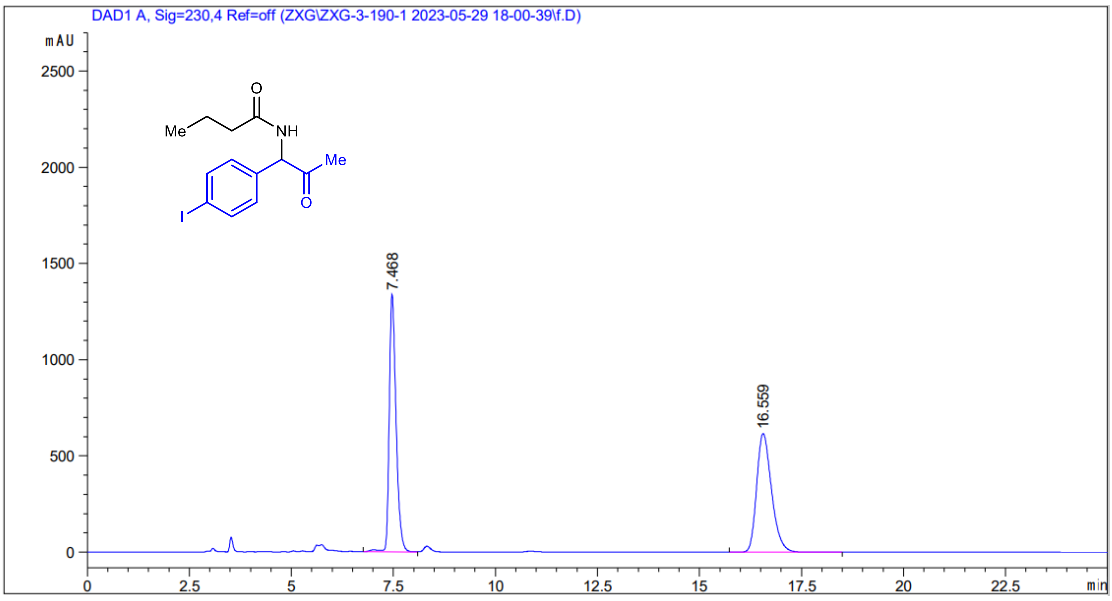

峰 #	保留时间 [min]	类型	峰宽 [min]	峰面积 [mAU*s]	峰高 [mAU]	峰面积 %
1	7.468	VV R	0.1762	1.55733e4	1337.78577	49.9281
2	16.559	BB	0.3896	1.56181e4	616.40295	50.0719

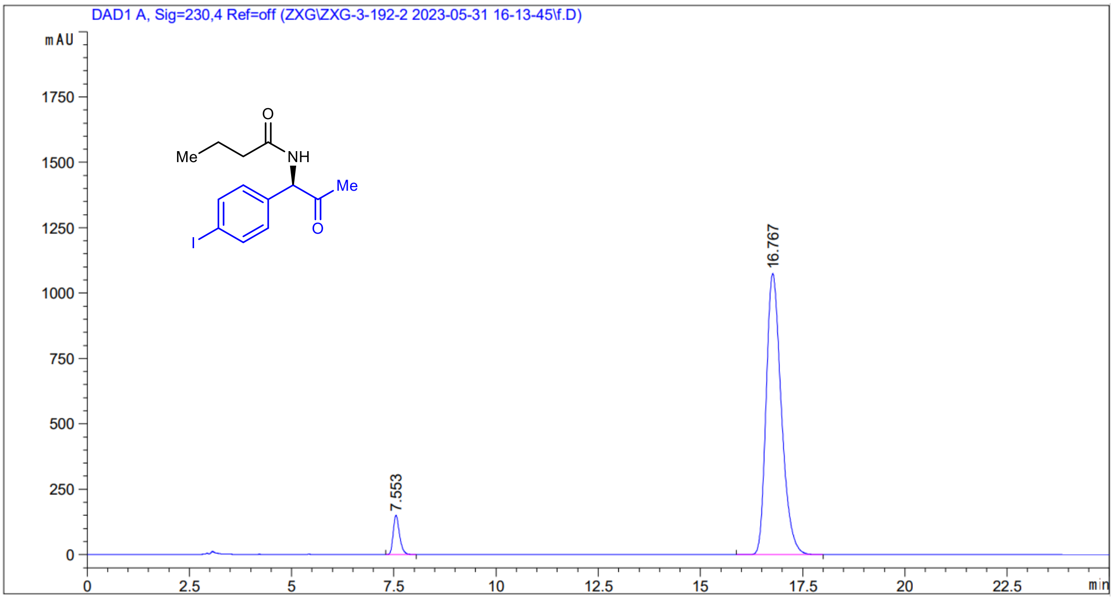

峰 #	保留时间 [min]	类型	峰宽 [min]	峰面积 [mAU*s]	峰高 [mAU]	峰面积 %
1	7.553	BB	0.1623	1601.96704	150.35869	5.4833
2	16.767	BB	0.3959	2.76132e4	1074.21362	94.5167

Supplementary Figure 204. HPLC spectrum of (*R*)-N-(1-(naphthalen-2-yl)-2-oxopropyl)butyramide (4j)

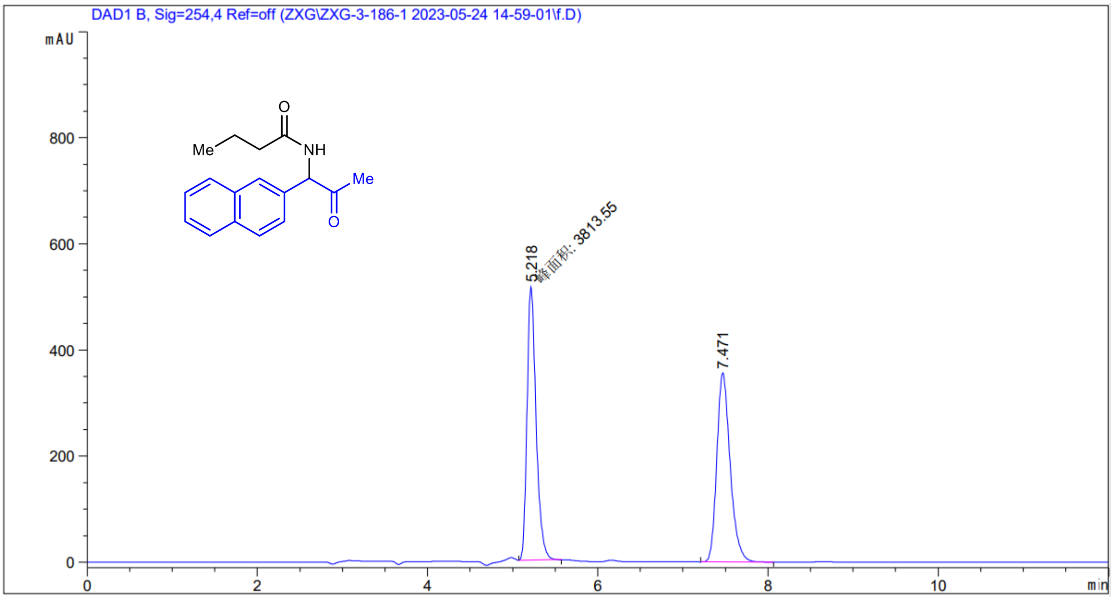

峰 #	保留时间 [min]	类型	峰宽 [min]	峰面积 [mAU*s]	峰高 [mAU]	峰面积 %
1	5.218	MM	0.1232	3813.55078	516.02673	49.6562
2	7.471	BB	0.1646	3866.36060	356.28616	50.3438

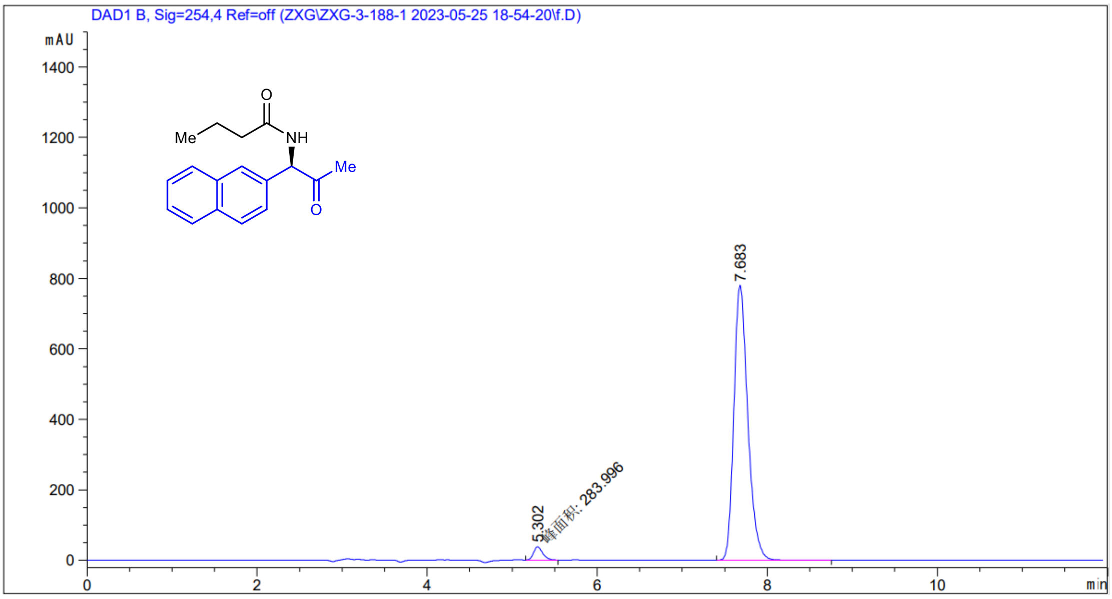

峰 #	保留时间 [min]	类型	峰宽 [min]	峰面积 [mAU*s]	峰高 [mAU]	峰面积 %
1	5.302	MM	0.1256	283.99622	37.69046	3.0984
2	7.683	BB	0.1749	8881.86328	779.59521	96.9016

Supplementary Figure 205. HPLC spectrum of (*R*)-N-(1-([1,1'-biphenyl]-4-yl)-2-oxopropyl)butyramide (4k)

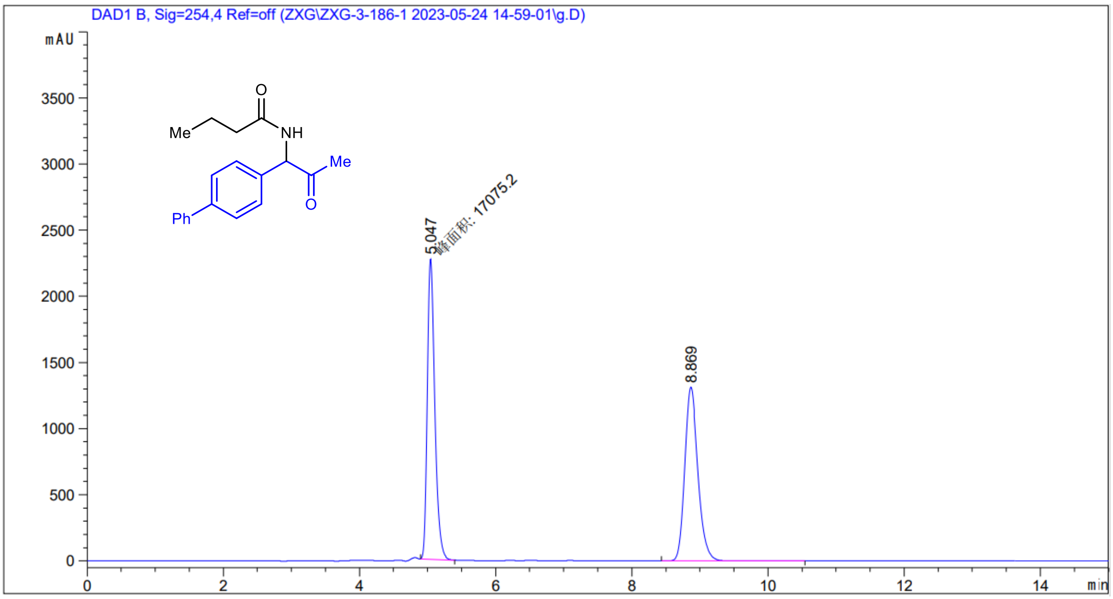

峰 #	保留时间 [min]	类型	峰宽 [min]	峰面积 [mAU*s]	峰高 [mAU]	峰面积 %
1	5.047	MM	0.1252	1.70752e4	2272.19189	49.1621
2	8.869	BB	0.2069	1.76573e4	1313.12573	50.8379

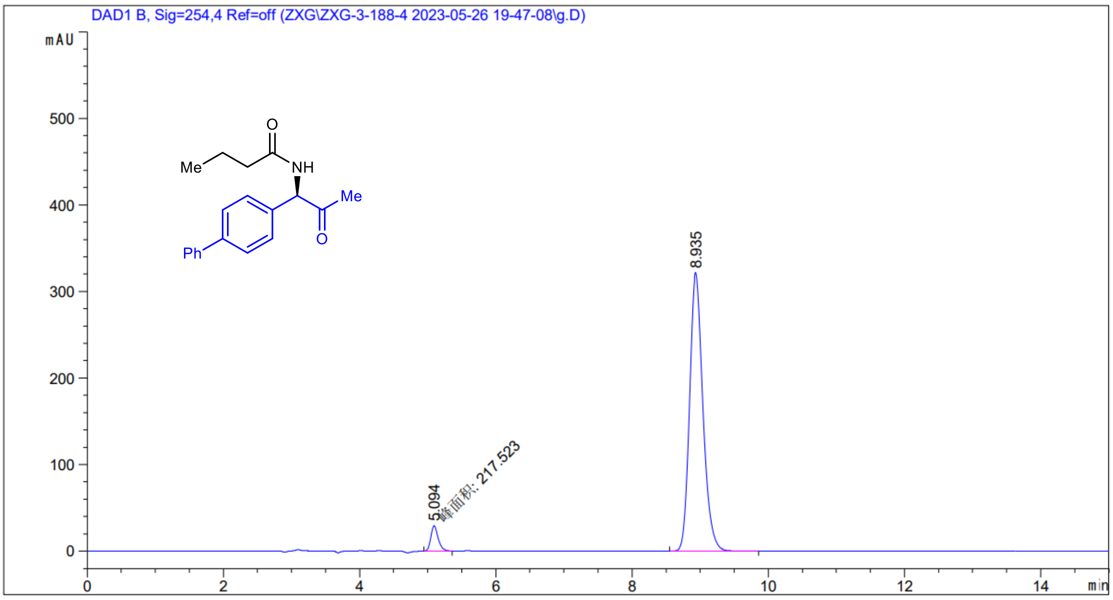

峰 #	保留时间 [min]	类型	峰宽 [min]	峰面积 [mAU*s]	峰高 [mAU]	峰面积 %
1	5.094	MM	0.1233	217.52321	29.40137	4.7116
2	8.935	BB	0.2095	4399.27197	321.72134	95.2884

Supplementary Figure 206. HPLC spectrum of (*R*)-N-(2-oxo-1-phenylbutyl)butyramide (4l)

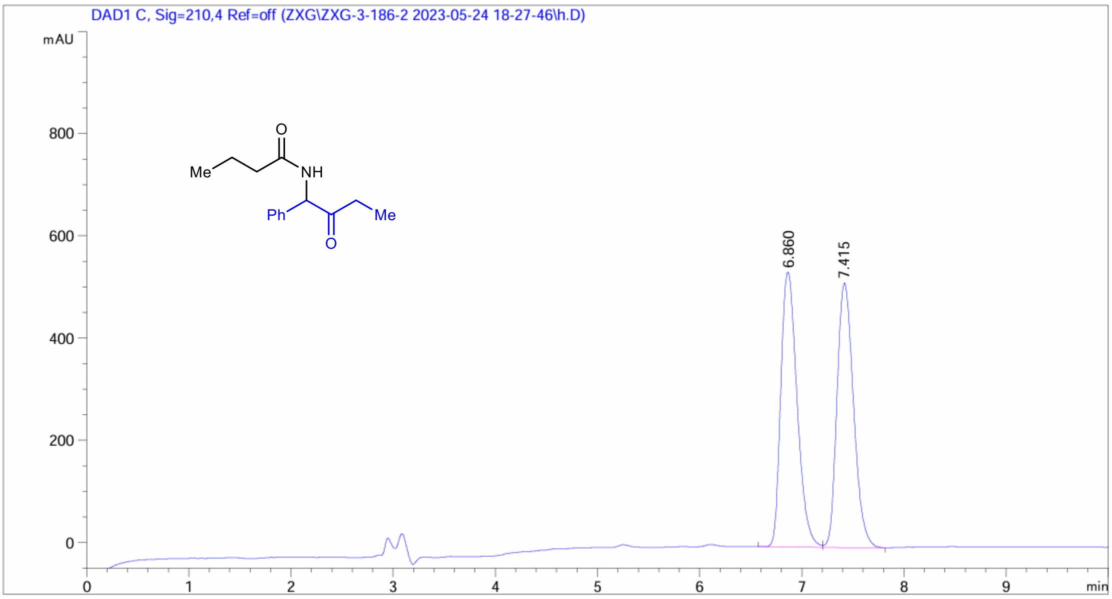

峰 #	保留时间 [min]	类型	峰宽 [min]	峰面积 [mAU*s]	峰高 [mAU]	峰面积 %
1	6.860	BV	0.1729	5939.40723	537.67950	50.5074
2	7.415	VB	0.1772	5820.07520	517.62543	49.4926

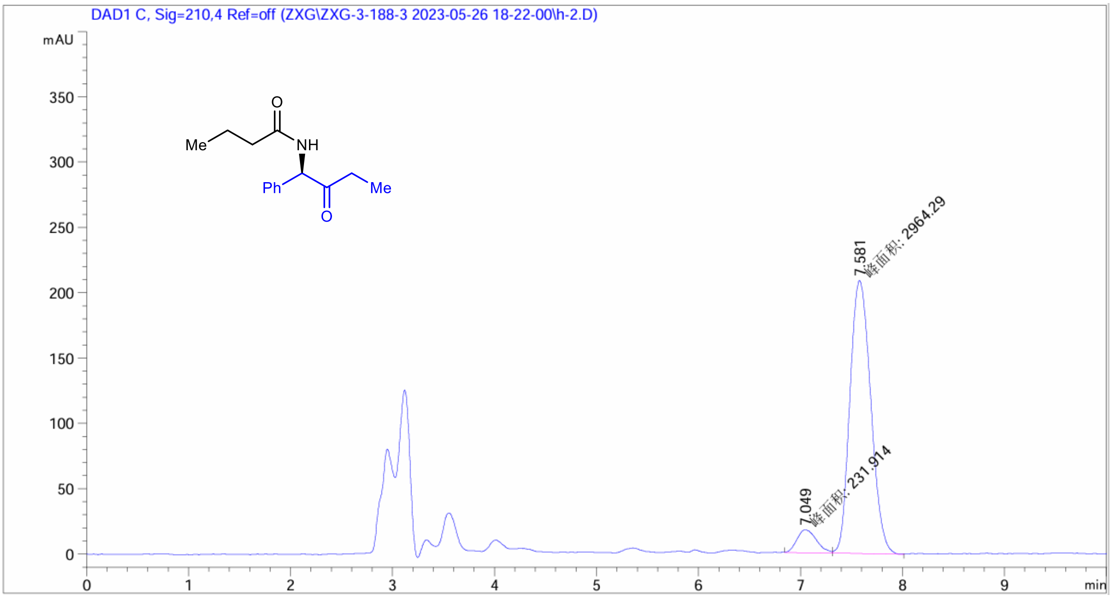

峰 #	保留时间 [min]	类型	峰宽 [min]	峰面积 [mAU*s]	峰高 [mAU]	峰面积 %
1	7.049	MF	0.2220	231.91438	17.41211	7.2559
2	7.581	FM	0.2364	2964.28564	208.98248	92.7441

Supplementary Figure 207. HPLC spectrum of (*R*)-N-(2-oxo-1-(p-tolyl)propyl)benzamide (4m)

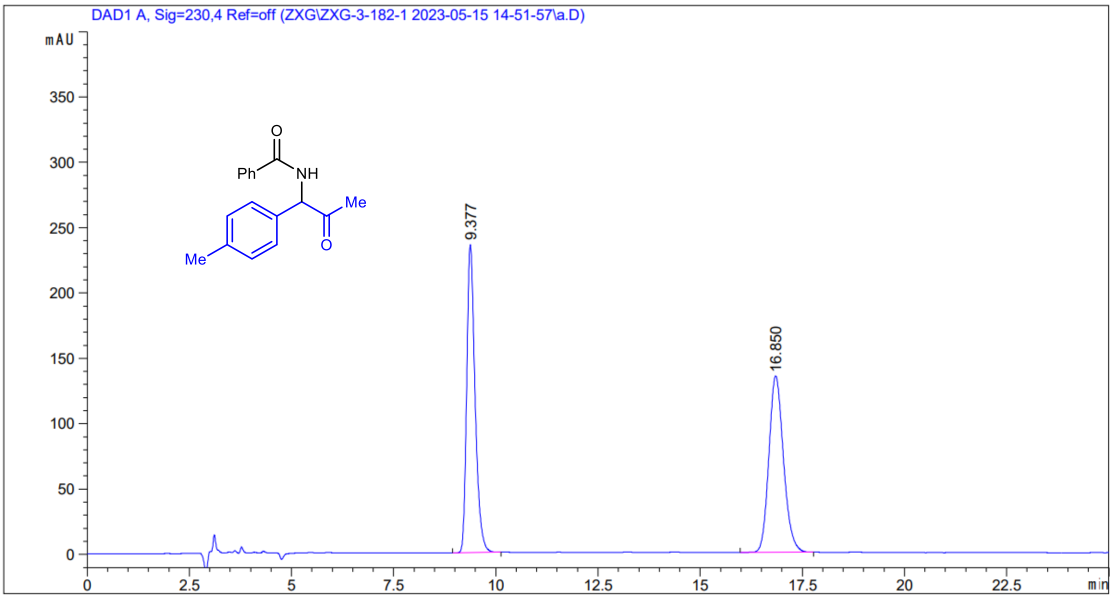

峰 #	保留时间 [min]	类型	峰宽 [min]	峰面积 [mAU*s]	峰高 [mAU]	峰面积 %
1	9.377	BB	0.2158	3350.54028	235.80510	49.9899
2	16.850	BB	0.3817	3351.88892	134.97870	50.0101

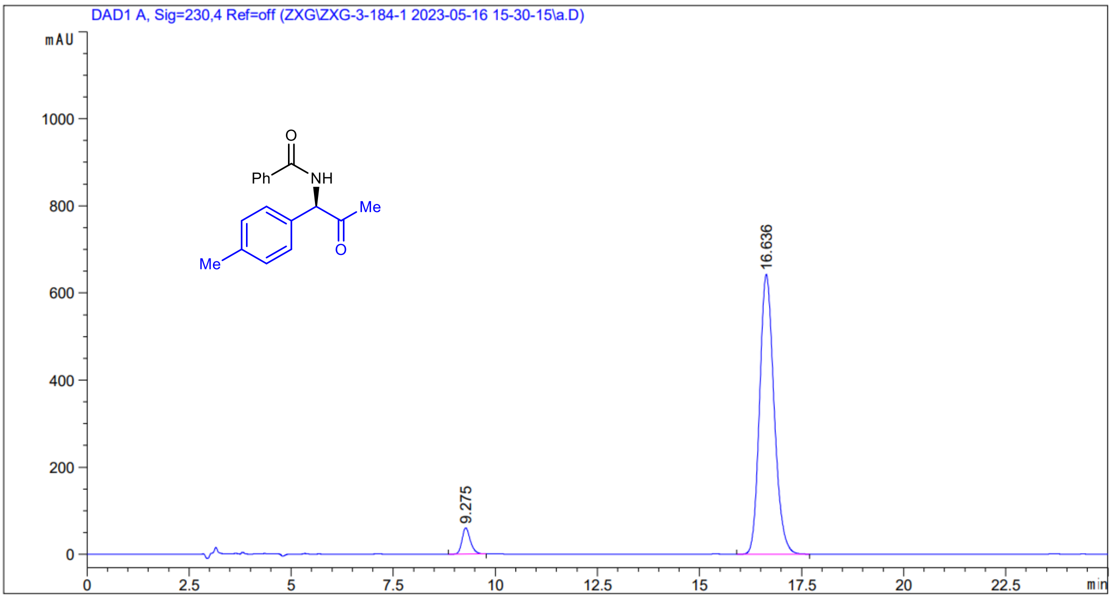

峰 #	保留时间 [min]	类型	峰宽 [min]	峰面积 [mAU*s]	峰高 [mAU]	峰面积 %
1	9.275	BB	0.2148	846.95300	59.94720	5.0745
2	16.636	BB	0.3799	1.58433e4	642.06970	94.9255

Supplementary Figure 208. HPLC spectrum of (*R*)-N-(1-(naphthalen-2-yl)-2-oxopropyl)benzamide (4n)

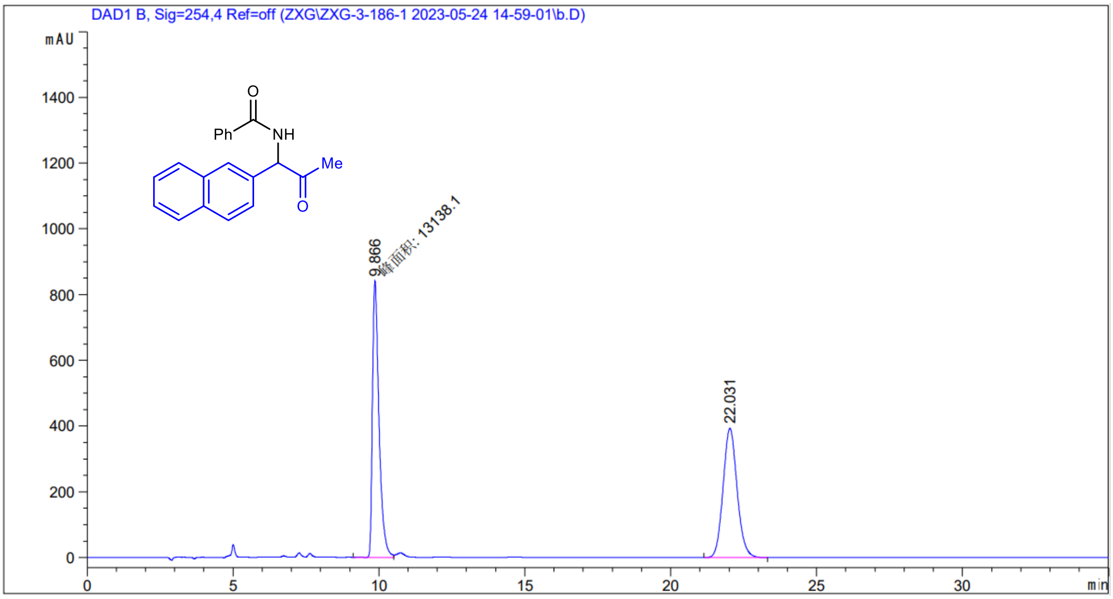

峰 #	保留时间 [min]	类型	峰宽 [min]	峰面积 [mAU*s]	峰高 [mAU]	峰面积 %
1	9.866	MF	0.2599	1.31381e4	842.39569	49.9798
2	22.031	BB	0.5139	1.31488e4	393.41153	50.0202

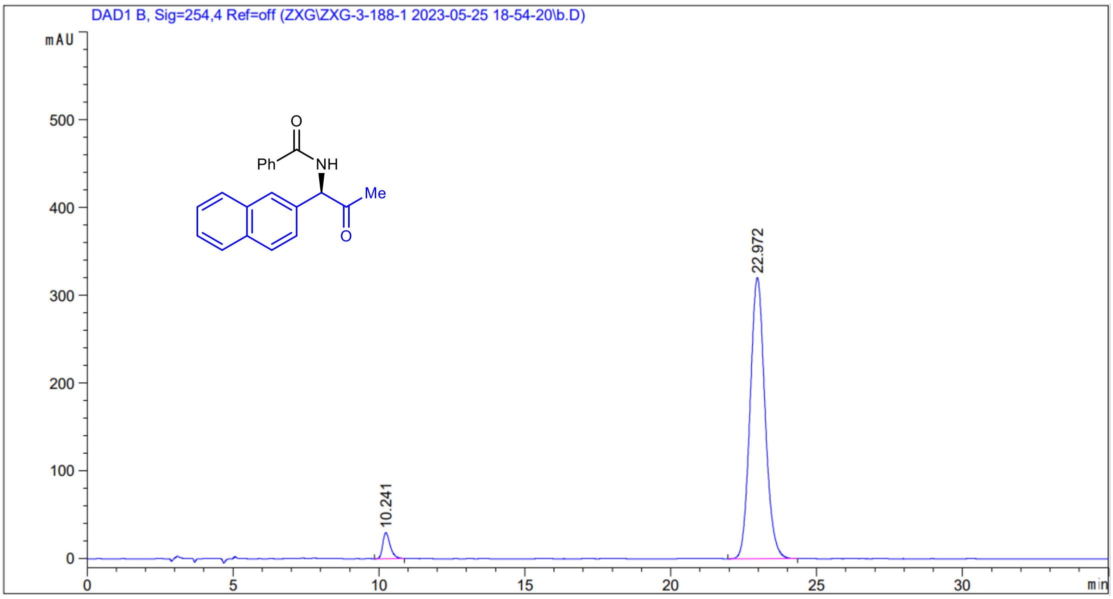

峰 #	保留时间 [min]	类型	峰宽 [min]	峰面积 [mAU*s]	峰高 [mAU]	峰面积 %
1	10.241	BB	0.2663	518.67413	29.61596	4.3660
2	22.972	BB	0.5460	1.13612e4	320.13669	95.6340

Supplementary Figure 209. HPLC spectrum of Methyl (*R*)-3-((1-(4-fluorophenyl)-2-oxopropyl)carbamoyl)bicyclo[1.1.1]pentane-1-carboxylate (4o)

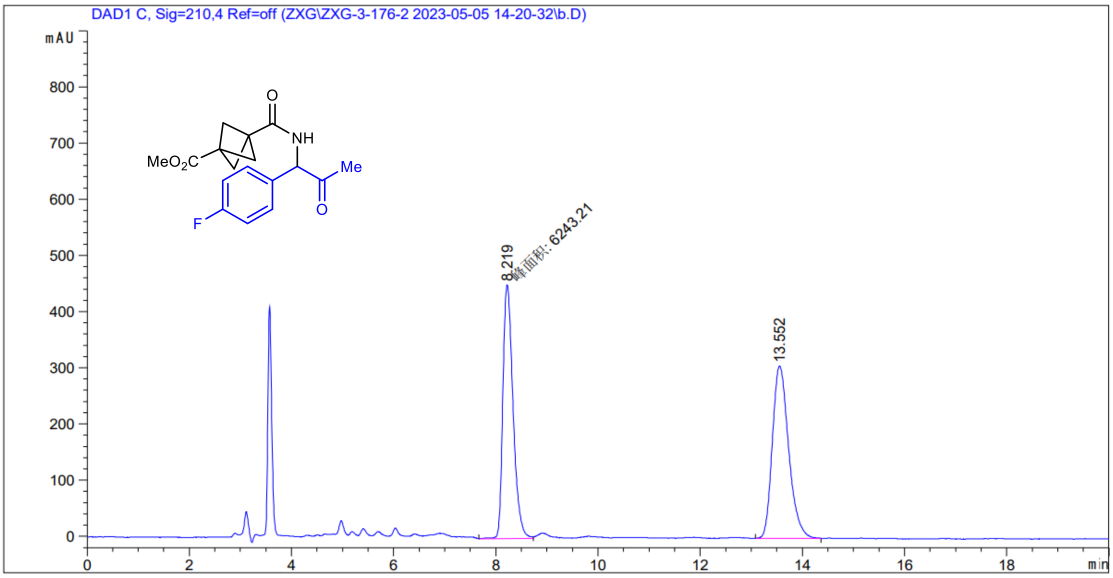

峰 #	保留时间 [min]	类型	峰宽 [min]	峰面积 [mAU*s]	峰高 [mAU]	峰面积 %
1	8.219	MF	0.2304	6243.21240	451.58194	47.9383
2	13.552	BB	0.3419	6780.22119	306.73477	52.0617

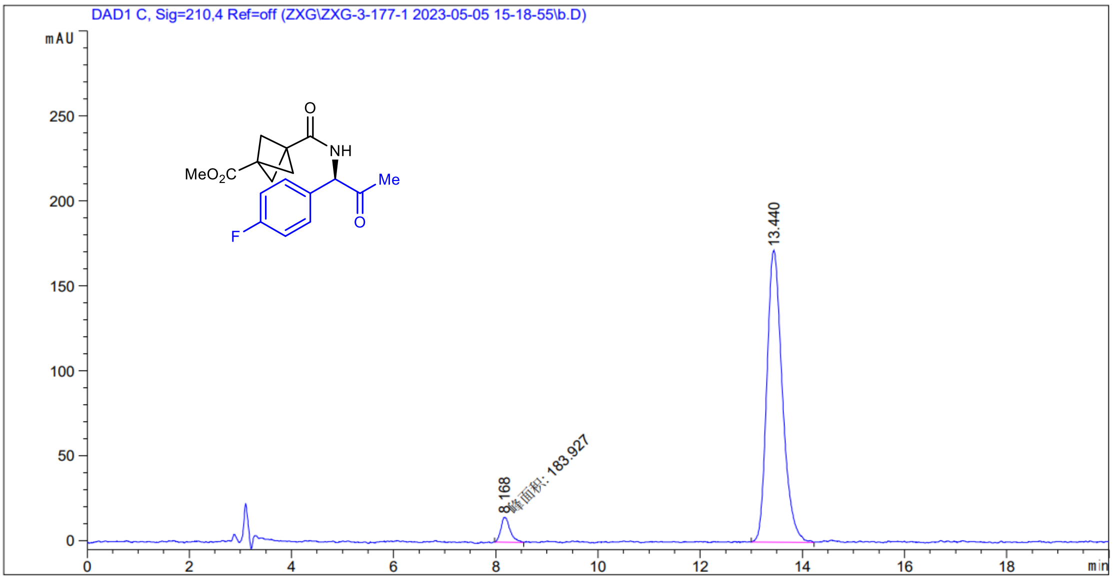

峰 #	保留时间 [min]	类型	峰宽 [min]	峰面积 [mAU*s]	峰高 [mAU]	峰面积 %
1	8.168	MM	0.2086	183.92659	14.69832	4.8453
2	13.440	BV R	0.3217	3612.04346	171.49251	95.1547

Supplementary Figure 210. HPLC spectrum of Methyl (*R*)-3-((1-(4-chlorophenyl)-2-oxopropyl)carbamoyl)bicyclo[1.1.1]pentane-1-carboxylate (4p)

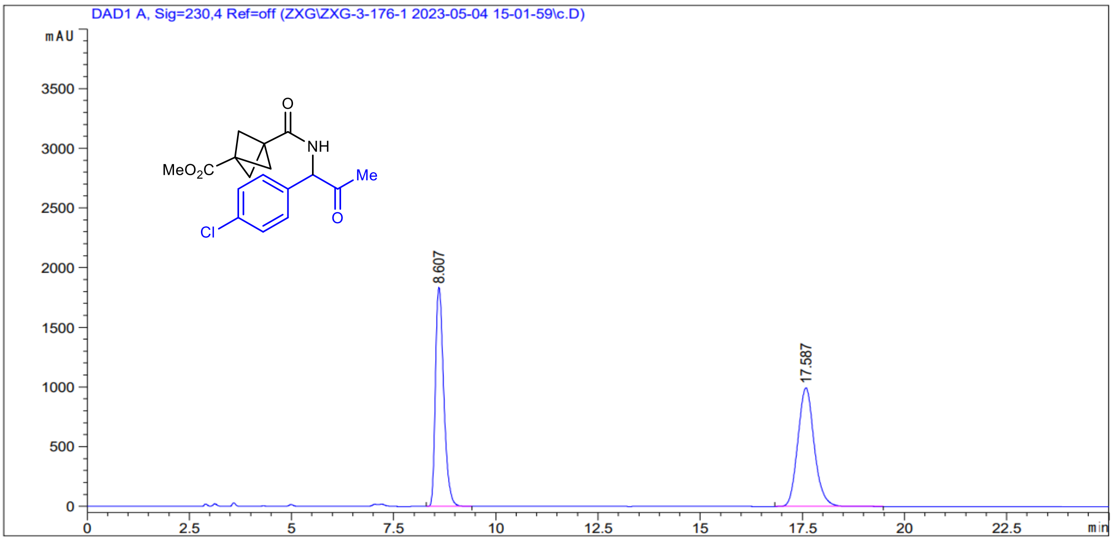

峰 #	保留时间 [min]	类型	峰宽 [min]	峰面积 [mAU*s]	峰高 [mAU]	峰面积 %
1	8.607	BB	0.2211	2.59520e4	1833.91565	48.3463
2	17.587	BB	0.4294	2.77274e4	994.13068	51.6537

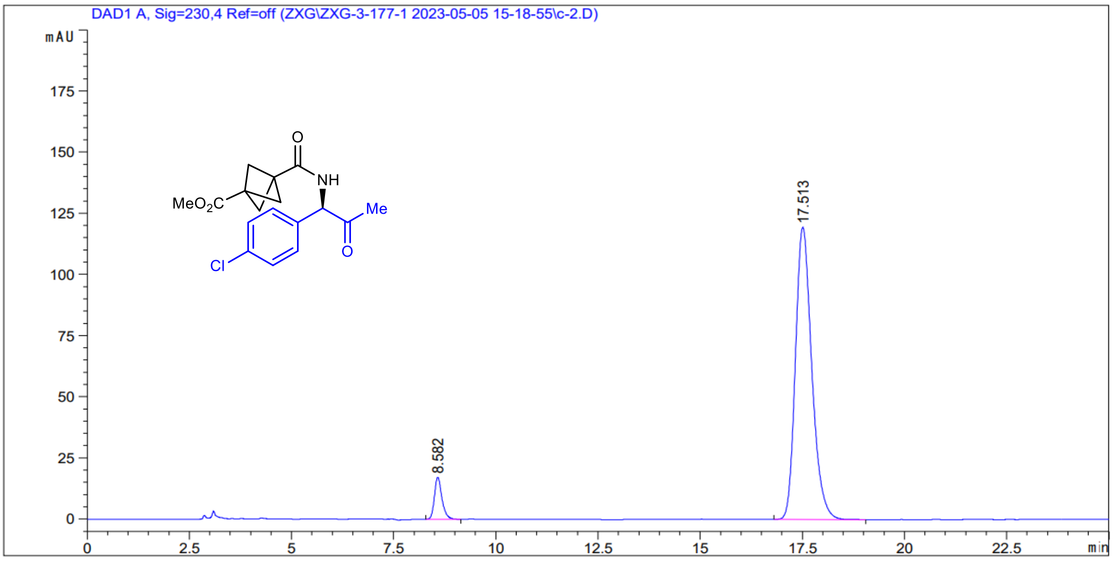

峰 #	保留时间 [min]	类型	峰宽 [min]	峰面积 [mAU*s]	峰高 [mAU]	峰面积 %
1	8.582	BB	0.1931	218.34508	17.07929	6.1752
2	17.513	BB	0.4261	3317.47363	119.41024	93.8248

Supplementary Figure 211. HPLC spectrum of Methyl (*R*)-3-((1-(4-bromophenyl)-2-oxopropyl)carbamoyl)bicyclo[1.1.1]pentane-1-carboxylate (4q)

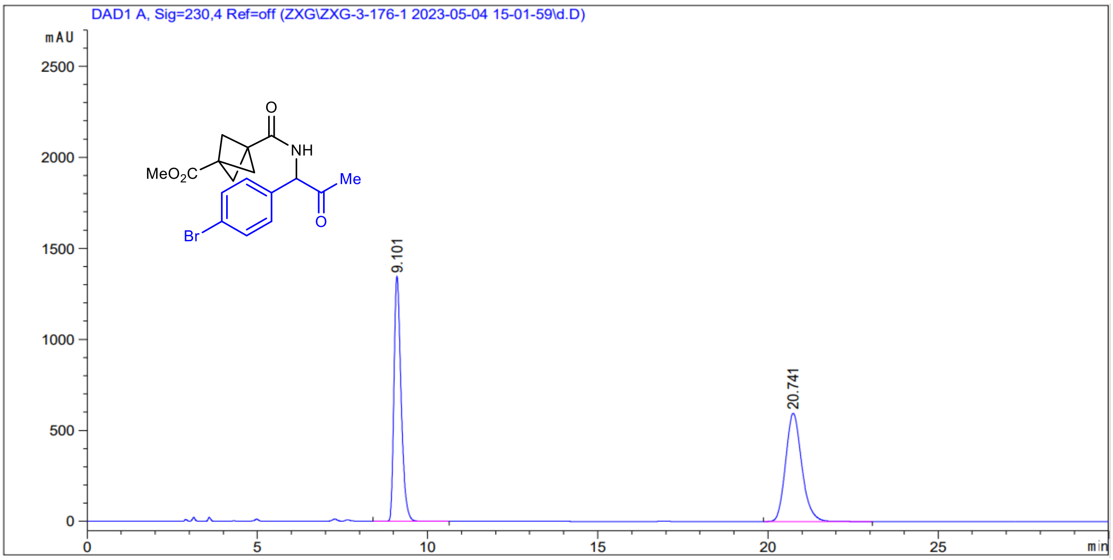

峰 #	保留时间 [min]	类型	峰宽 [min]	峰面积 [mAU*s]	峰高 [mAU]	峰面积 %
1	9.101	VB R	0.2195	1.93531e4	1346.07239	49.6381
2	20.741	BB	0.5053	1.96353e4	594.50201	50.3619

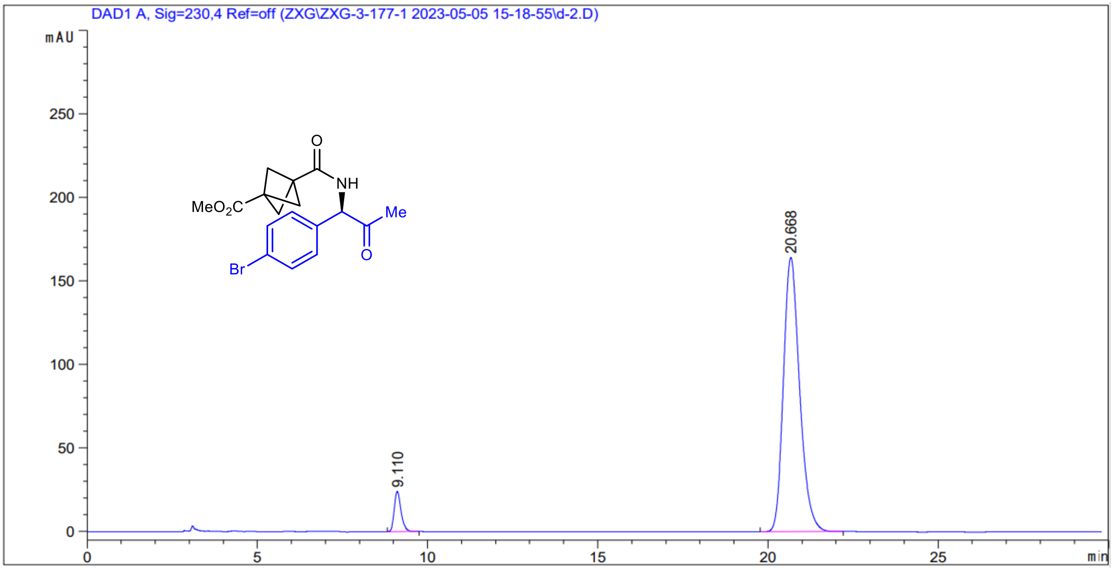

峰 #	保留时间 [min]	类型	峰宽 [min]	峰面积 [mAU*s]	峰高 [mAU]	峰面积 %
1	9.110	BB	0.2083	330.71130	24.07461	5.7306
2	20.668	BB	0.5089	5440.26416	164.03963	94.2694

Supplementary Figure 212. HPLC spectrum of (*R*)-2-(1-(4-chlorobenzoyl)-5-methoxy-2-methyl-1H-indol-3-yl)-N-(2-oxo-1-phenylpropyl)acetamide (5a)

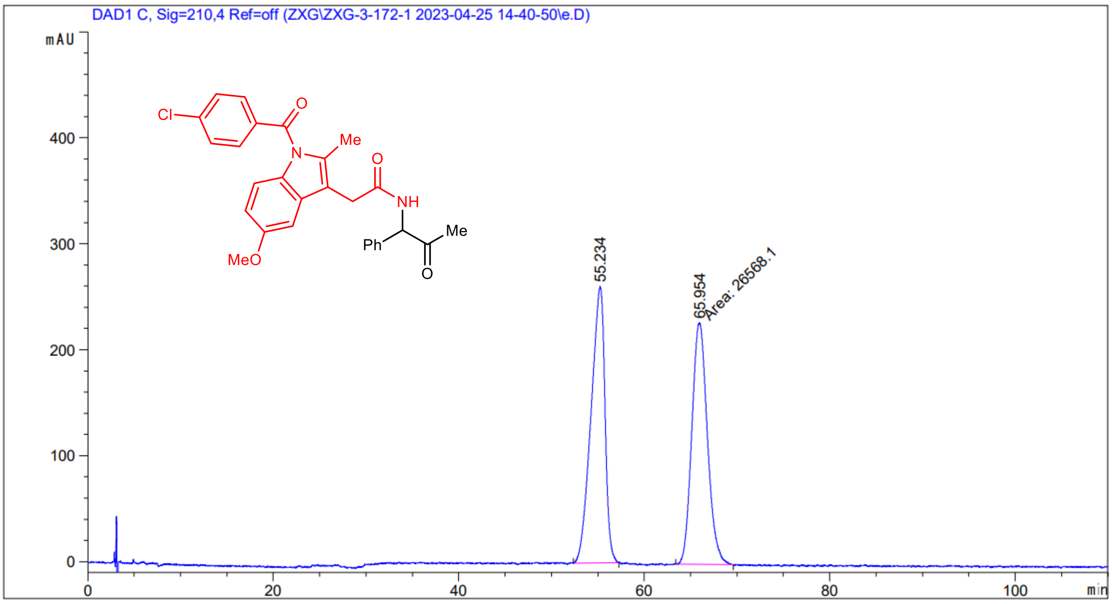

Peak #	RetTime [min]	Type	Width [min]	Area [mAU*s]	Height [mAU]	Area %
1	55.234	VV R	1.2229	2.72113e4	260.87671	50.5980
2	65.954	MM	1.9421	2.65681e4	228.00209	49.4020

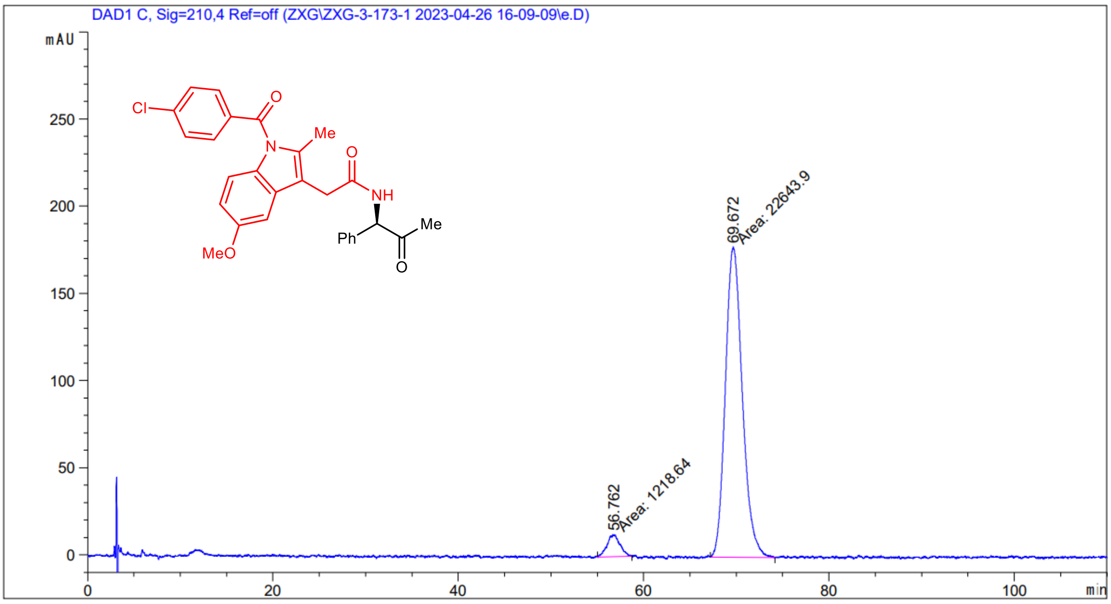

Peak #	RetTime [min]	Type	Width [min]	Area [mAU*s]	Height [mAU]	Area %
1	56.762	MM	1.6170	1218.63867	12.56105	5.1069
2	69.672	MM	2.1183	2.26439e4	178.15686	94.8931

Supplementary Figure 213. HPLC spectrum of (*R*)-1-(2,6-difluorobenzyl)-N-(2-oxo-1-phenylpropyl)-1H-1,2,3-triazole-4-carboxamide (5b)

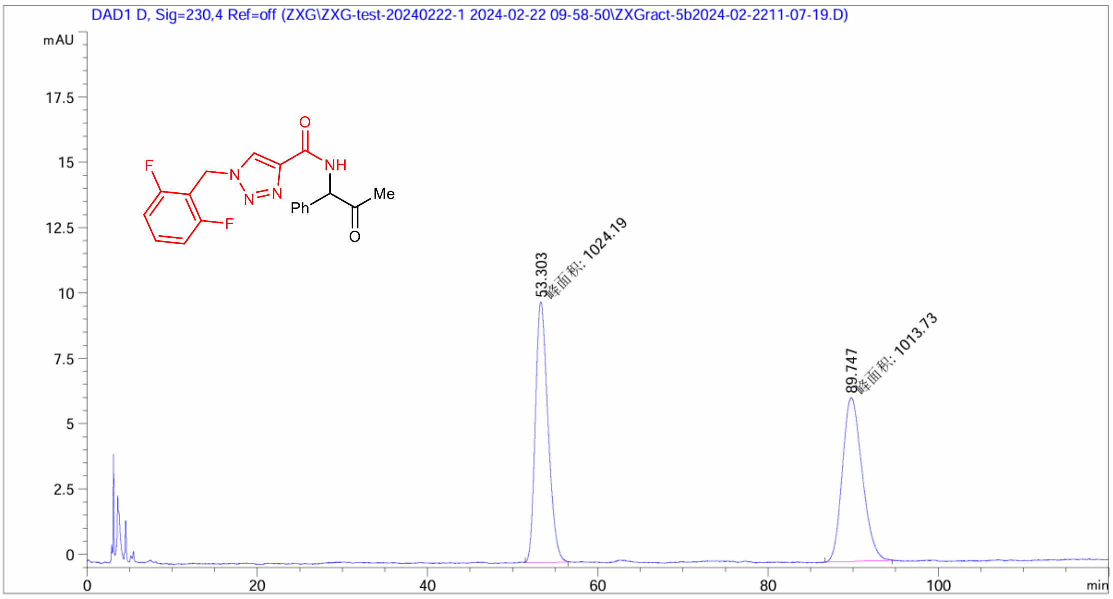

峰 #	保留时间 [min]	类型	峰宽 [min]	峰面积 [mAU*s]	峰高 [mAU]	峰面积 %
1	53.303	MM	1.7133	1024.19202	9.96288	50.2568
2	89.747	MM	2.6970	1013.72638	6.26457	49.7432

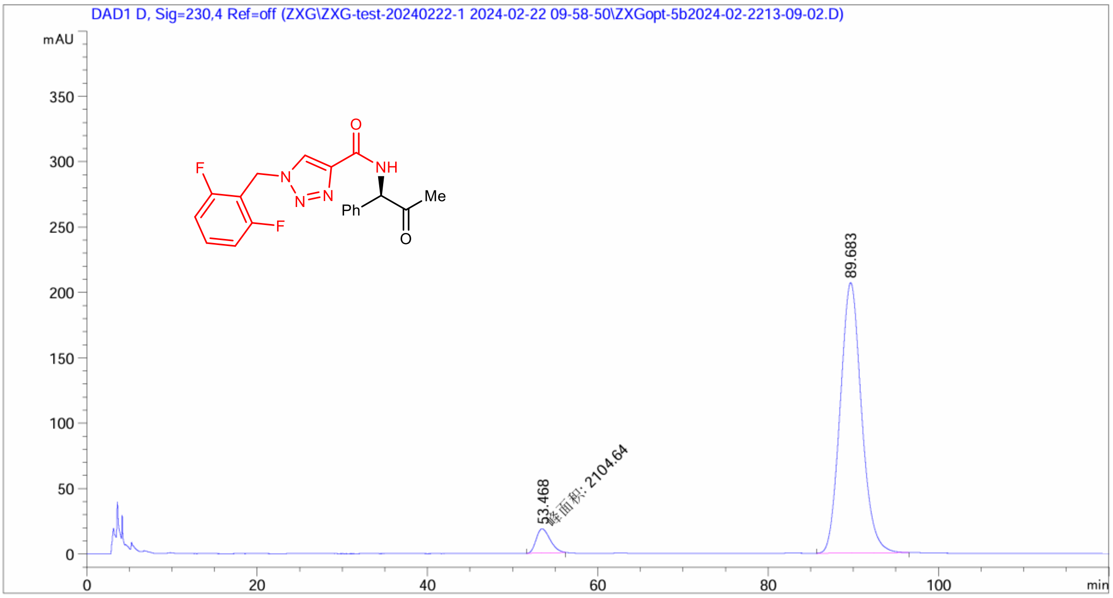

峰 #	保留时间 [min]	类型	峰宽 [min]	峰面积 [mAU*s]	峰高 [mAU]	峰面积 %
1	53.468	MM	1.8920	2104.63696	18.54004	5.5392
2	89.683	BB	2.4917	3.58907e4	206.70705	94.4608

Supplementary Figure 214. HPLC spectrum of (*R*)-3-(4,5-diphenyloxazol-2-yl)-N-(2-oxo-1-phenylpropyl)propanamide (5c)

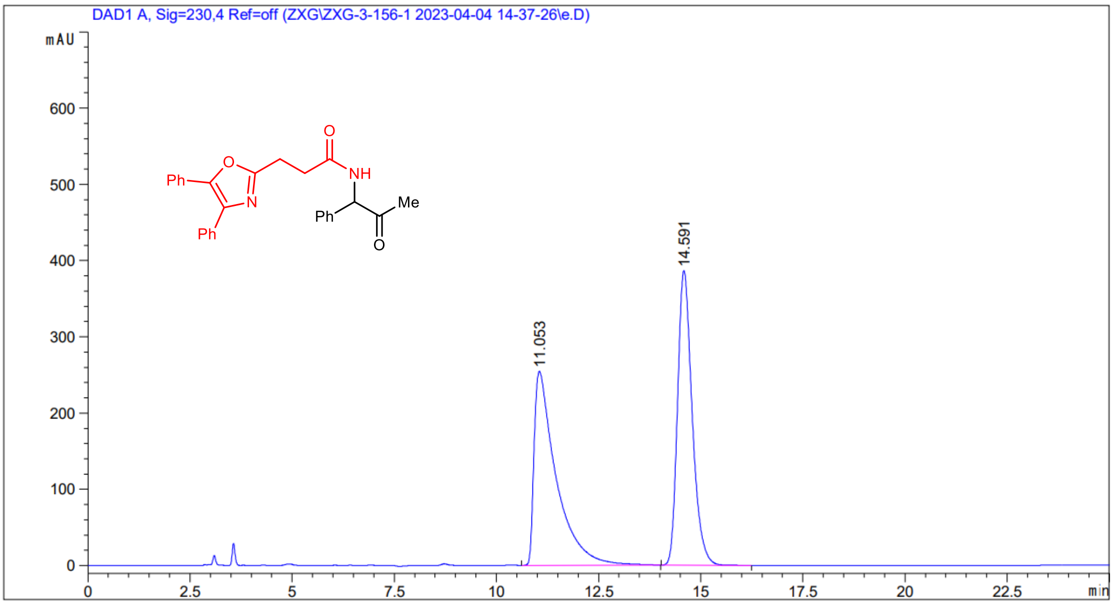

Peak #	RetTime [min]	Type	Width [min]	Area [mAU*s]	Height [mAU]	Area %
1	11.053	BB	0.5283	9558.24609	254.39586	49.9176
2	14.591	BB	0.3797	9589.78516	386.18750	50.0824

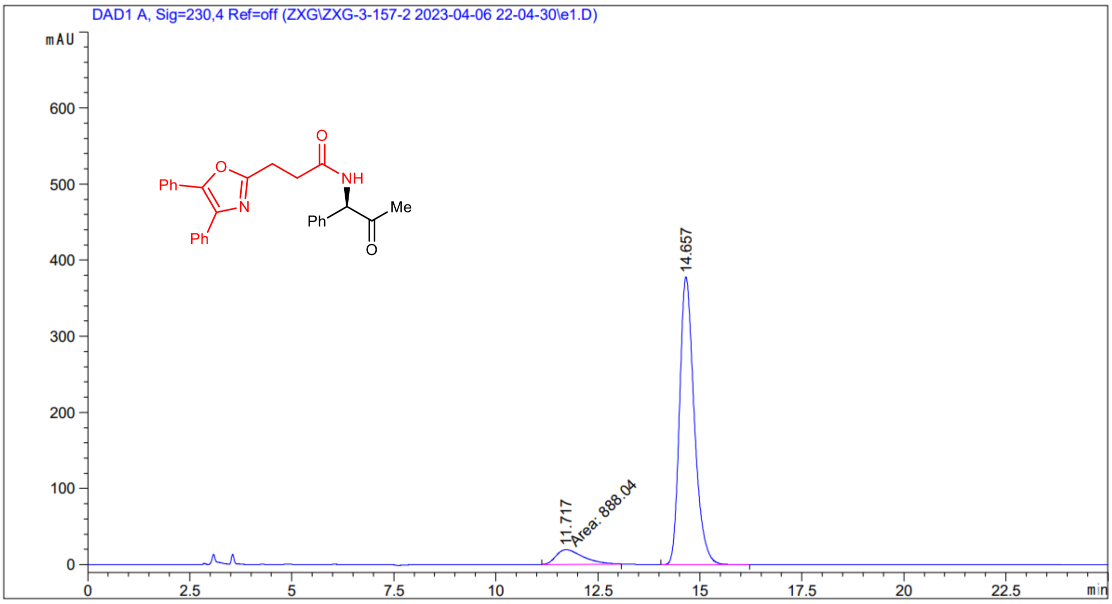

Peak #	RetTime [min]	Type	Width [min]	Area [mAU*s]	Height [mAU]	Area %
1	11.717	MM	0.7687	888.04028	19.25506	8.6089
2	14.657	BB	0.3811	9427.35547	377.79895	91.3911

Supplementary Figure 215. HPLC spectrum of (*S*)-2-(6-methoxynaphthalen-2-yl)-N-((*R*)-2-oxo-1-phenylpropyl)propanamide (5d)

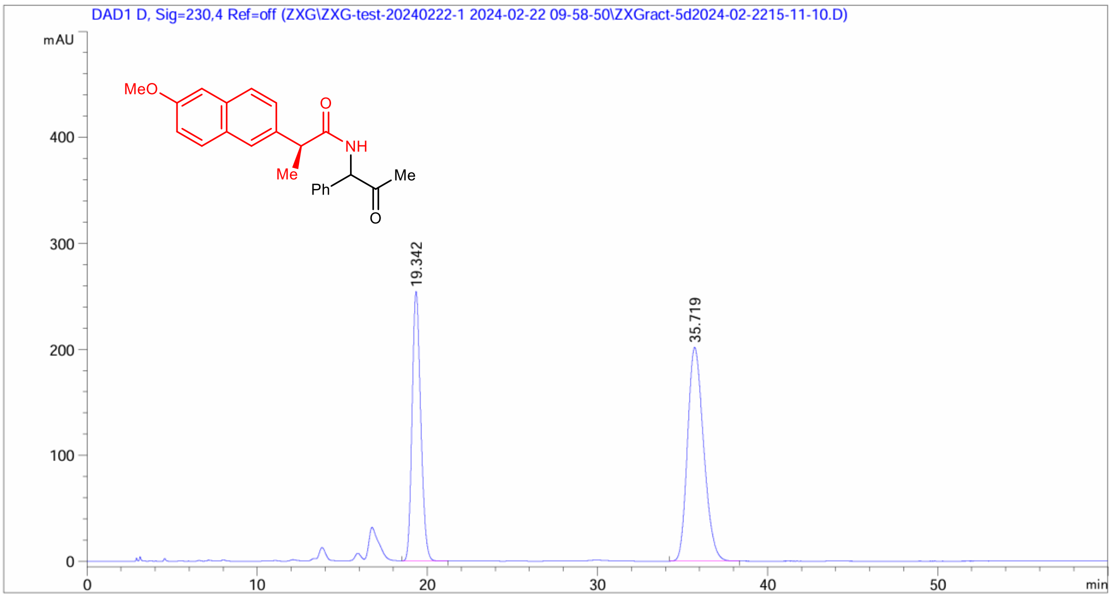

峰 #	保留时间 [min]	类型	峰宽 [min]	峰面积 [mAU*s]	峰高 [mAU]	峰面积 %
1	19.342	BB	0.5414	8969.59766	254.38710	40.3206
2	35.719	BB	1.0140	1.32761e4	201.67284	59.6794

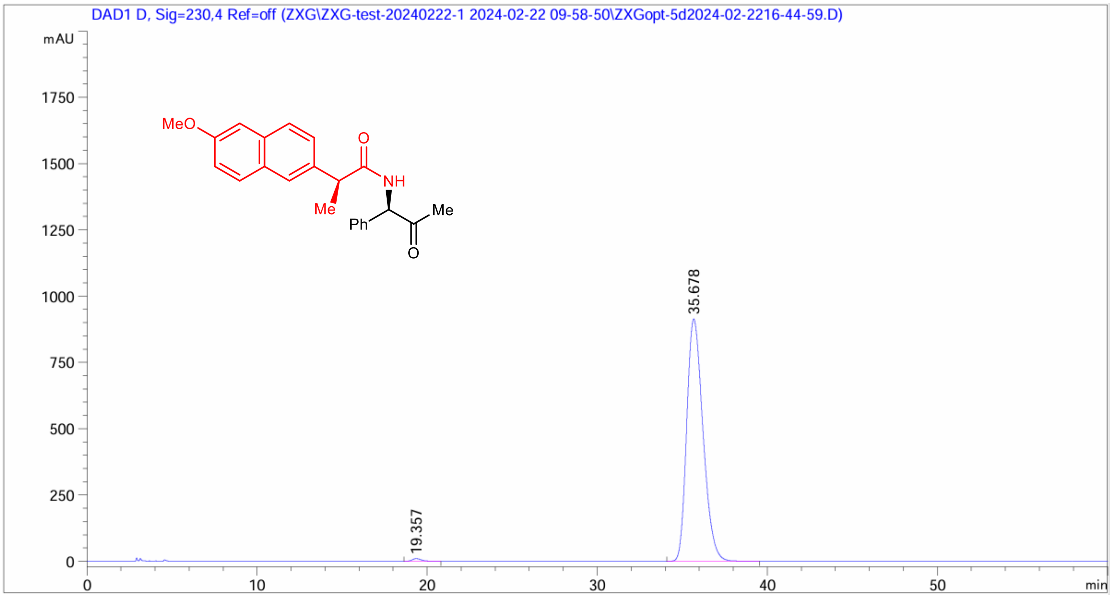

峰 #	保留时间 [min]	类型	峰宽 [min]	峰面积 [mAU*s]	峰高 [mAU]	峰面积 %
1	19.357	BB	0.5425	352.70520	9.83208	0.5776
2	35.678	BB	1.0312	6.07140e4	913.71631	99.4224

Supplementary Figure 216. HPLC spectrum of (*R*)-N-((*R*)-2-oxo-1-phenylpropyl)-2-(2-oxopyrrolidin-1-yl)butanamide (5e)

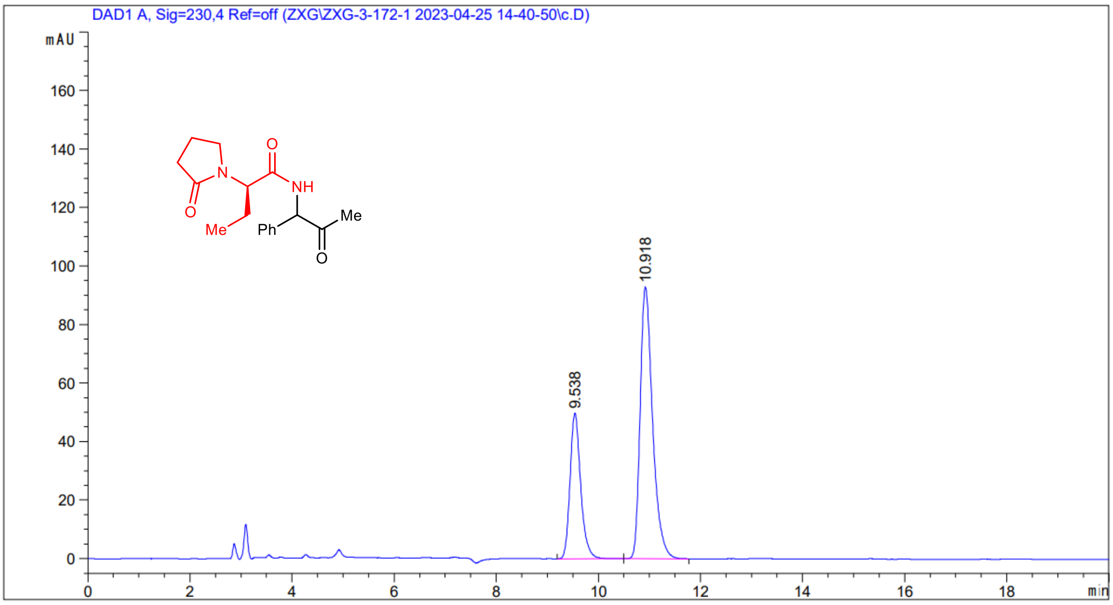

Peak #	RetTime [min]	Type	Width [min]	Area [mAU*s]	Height [mAU]	Area %
1	9.538	BB	0.2210	721.45691	49.80563	31.4801
2	10.918	BB	0.2552	1570.33057	92.90637	68.5199

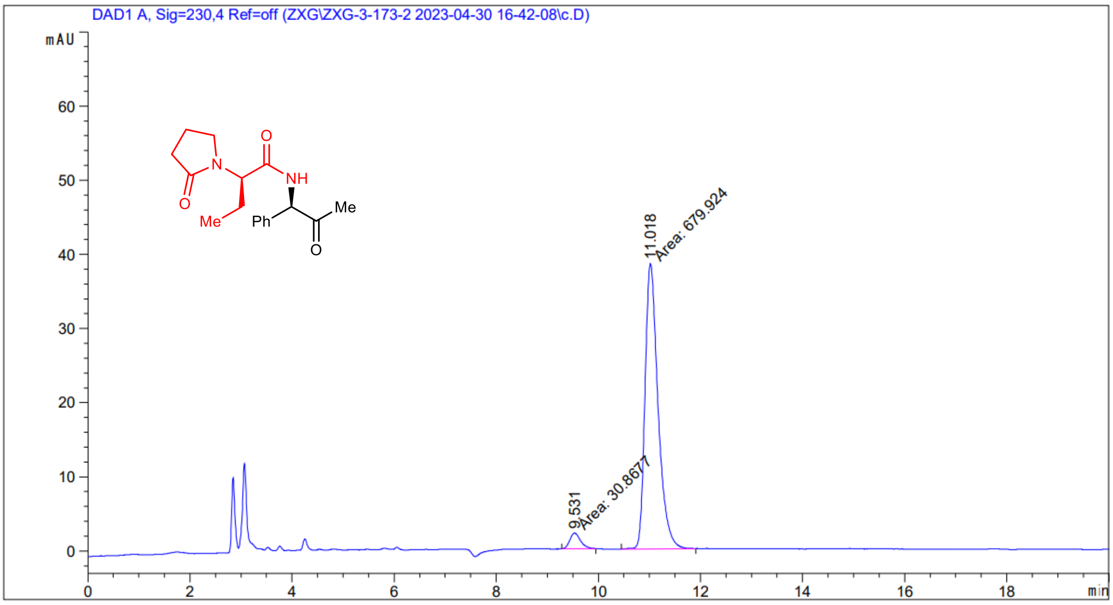

Peak #	RetTime [min]	Type	Width [min]	Area [mAU*s]	Height [mAU]	Area %
1	9.531	MM	0.2422	30.86773	2.12403	4.3427
2	11.018	MM	0.2948	679.92413	38.44207	95.6573

Supplementary Figure 217. HPLC spectrum of (2*S*,5*R*,6*R*)-3,3-dimethyl-7-oxo-N-((*R*)-2-oxo-1-phenylpropyl)-6-(2-phenylacetamido)-4-thia-1-azabicyclo[3.2.0]heptane-2-carboxamide (5f)

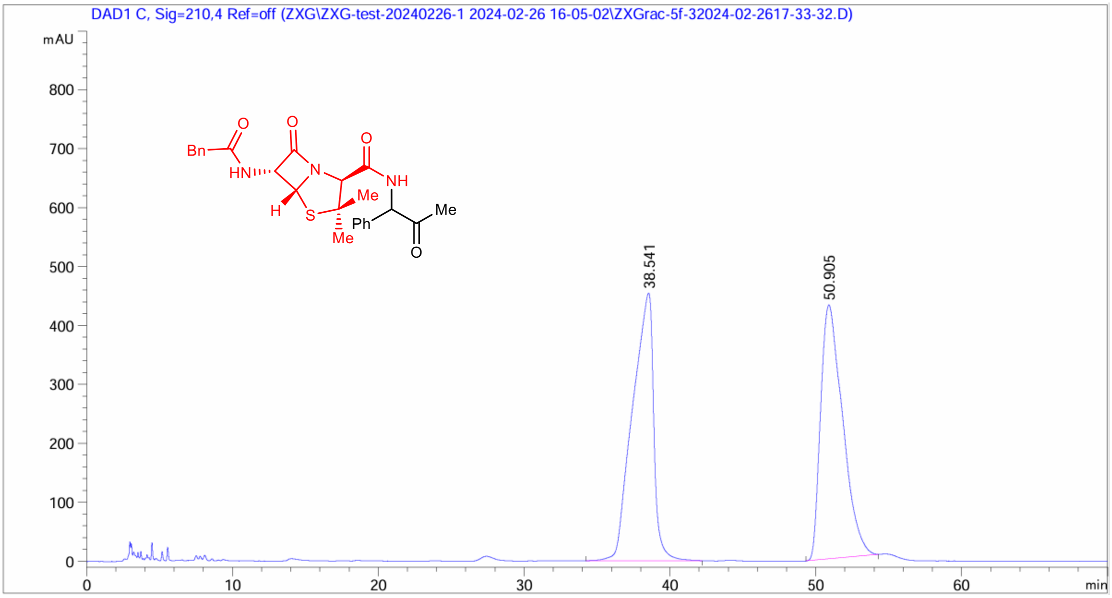

峰 #	保留时间 [min]	类型	峰宽 [min]	峰面积 [mAU*s]	峰高 [mAU]	峰面积 %
1	38.541	BB	1.3323	4.58205e4	454.43640	50.3139
2	50.905	BB	1.5244	4.52489e4	431.15436	49.6861

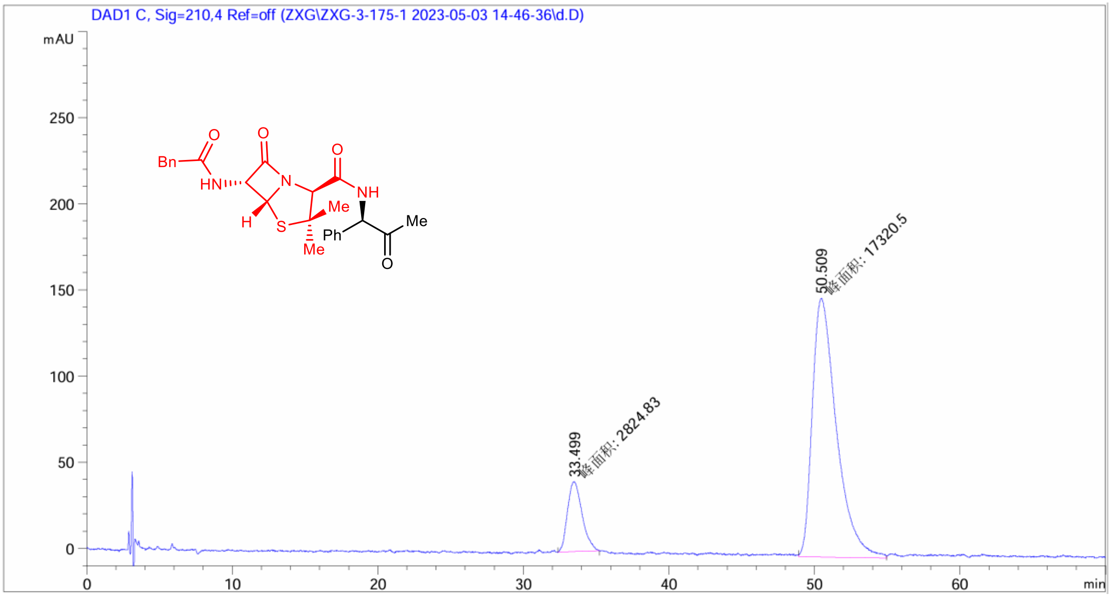

峰 #	保留时间 [min]	类型	峰宽 [min]	峰面积 [mAU*s]	峰高 [mAU]	峰面积 %
1	33.499	MM	1.1585	2824.82813	40.63830	14.0222
2	50.509	MM	1.9231	1.73205e4	150.10765	85.9778

Supplementary Figure 218. HPLC spectrum of Methyl N-(*tert*-butoxycarbonyl)-N-((*R*)-2-oxo-1-phenylpropyl)-L-glutaminate (5g)

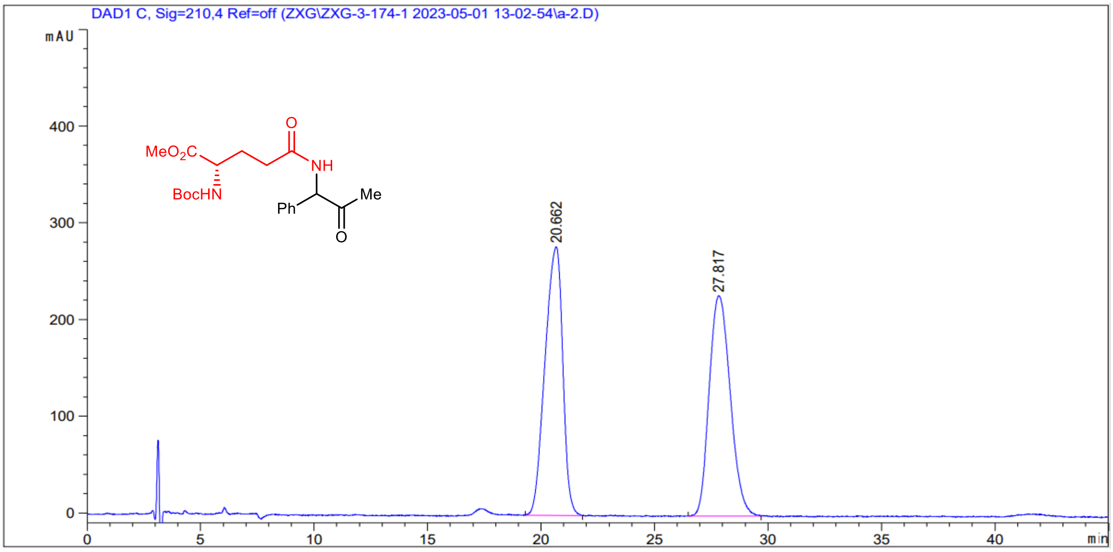

峰 #	保留时间 [min]	类型	峰宽 [min]	峰面积 [mAU*s]	峰高 [mAU]	峰面积 %
1	20.662	BV R	0.6468	1.50706e4	277.85965	50.6255
2	27.817	VV R	0.7586	1.46983e4	227.85933	49.3745

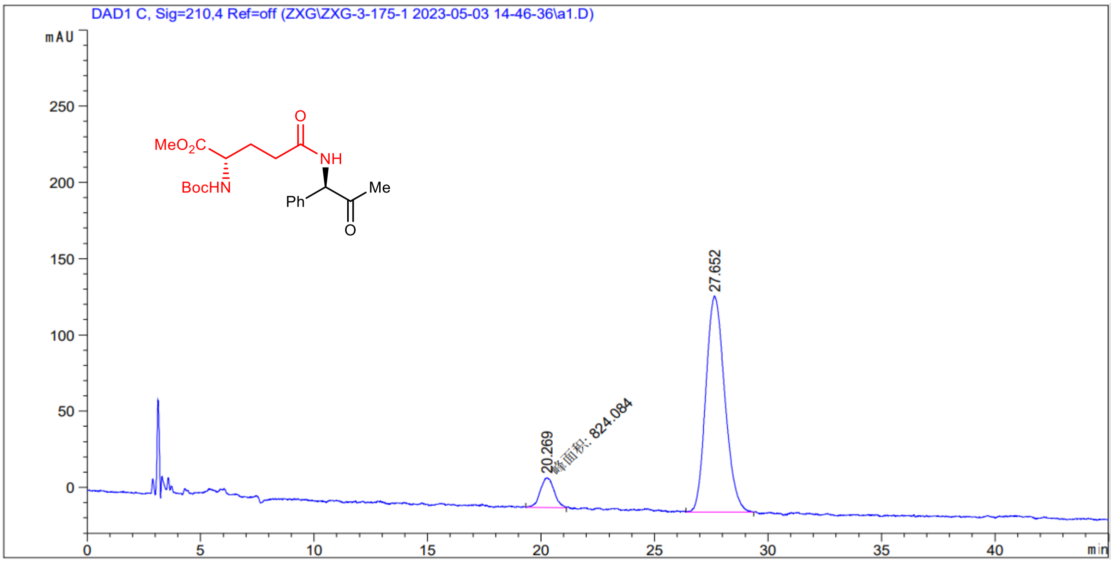

峰 #	保留时间 [min]	类型	峰宽 [min]	峰面积 [mAU*s]	峰高 [mAU]	峰面积 %
1	20.269	MM	0.7026	824.08392	19.54706	9.0947
2	27.652	VV R	0.7006	8237.03809	141.60367	90.9053

Supplementary Figure 219. HPLC spectrum of Methyl N-(*tert*-butoxycarbonyl)-N-((*R*)-2-oxo-1-phenylpropyl)-L-asparaginate (5h)

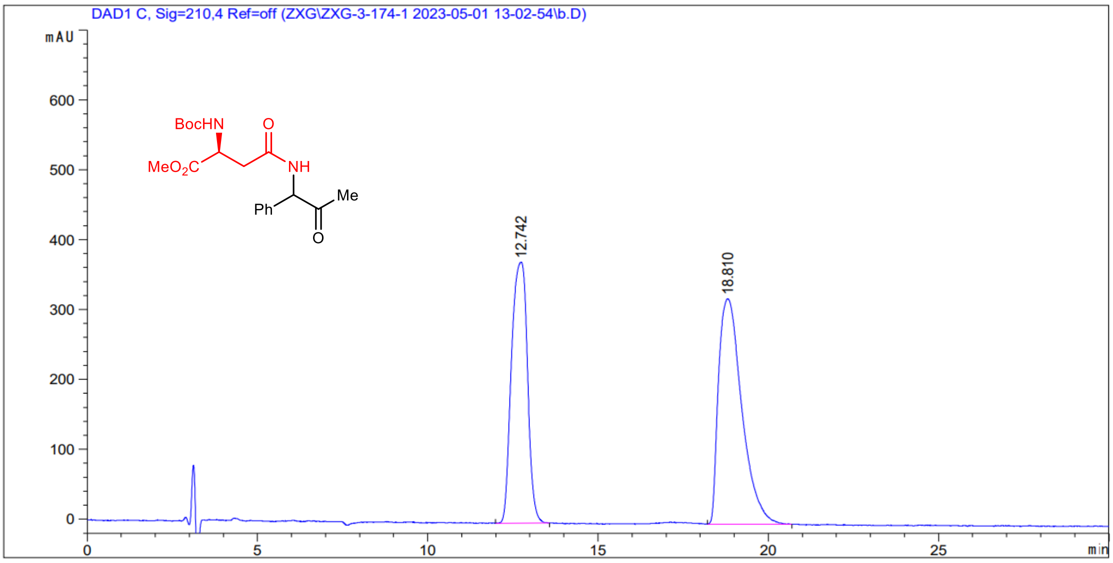

峰 #	保留时间 [min]	类型	峰宽 [min]	峰面积 [mAU*s]	峰高 [mAU]	峰面积 %
1	12.742	VV R	0.3872	1.22076e4	373.63910	44.3182
2	18.810	BV R	0.5667	1.53377e4	322.60587	55.6818

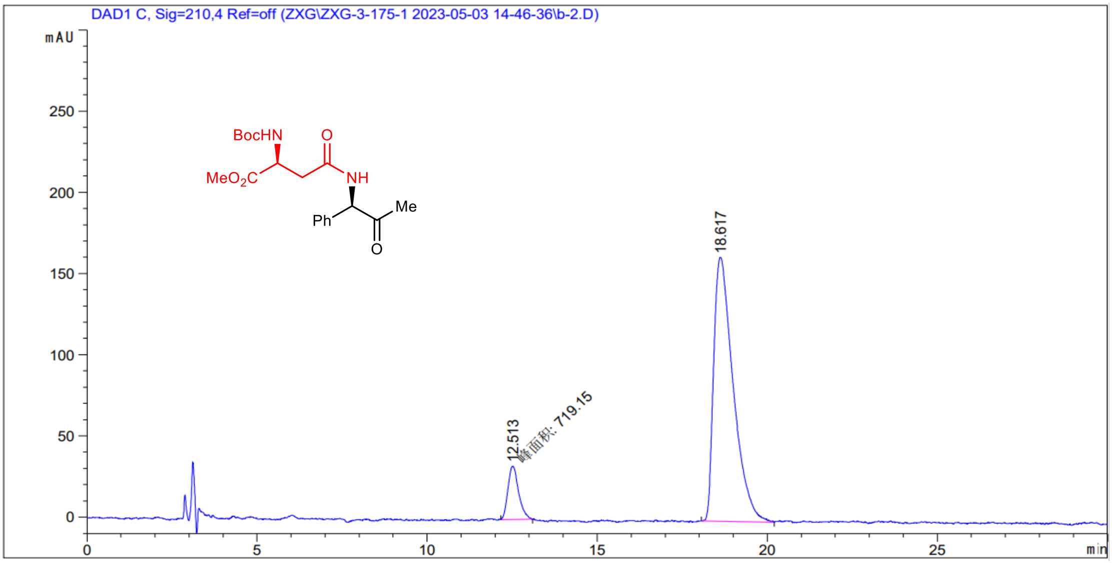

峰 #	保留时间 [min]	类型	峰宽 [min]	峰面积 [mAU*s]	峰高 [mAU]	峰面积 %
1	12.513	MM	0.3641	719.14984	32.92196	9.9939
2	18.617	VV R	0.5389	6476.77393	162.53999	90.0061

Supplementary Figure 220. HPLC spectrum of N-((1*R*, 2*R*)-2-hydroxy-1,2-diphenylpropyl)benzamide (7a)

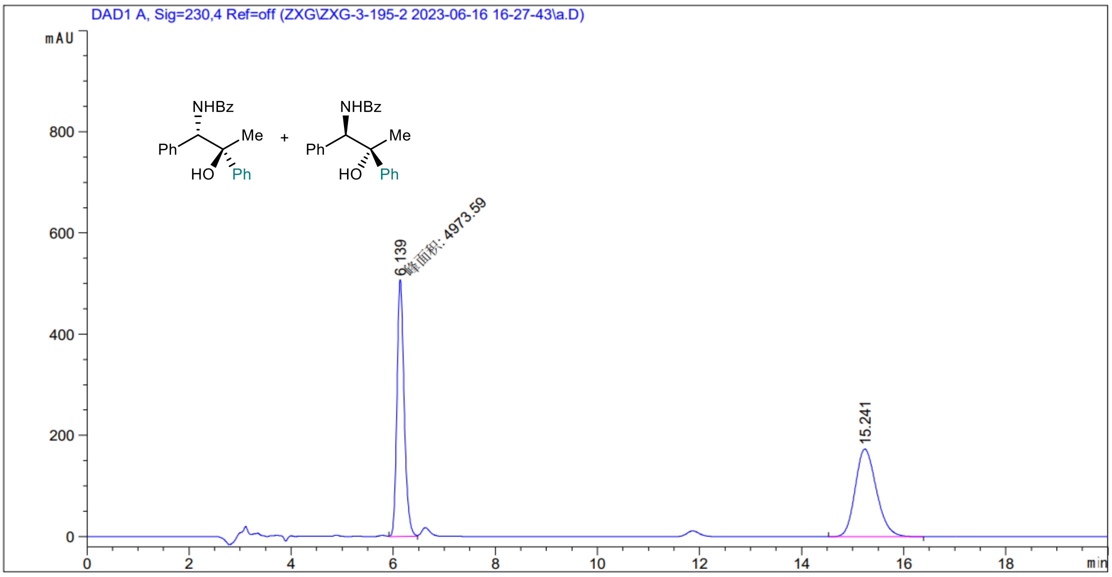

峰 #	保留时间 [min]	类型	峰宽 [min]	峰面积 [mAU*s]	峰高 [mAU]	峰面积 %
1	6.139	FM	0.1632	4973.59033	507.82654	49.8974
2	15.241	BB	0.4467	4994.04785	173.02542	50.1026

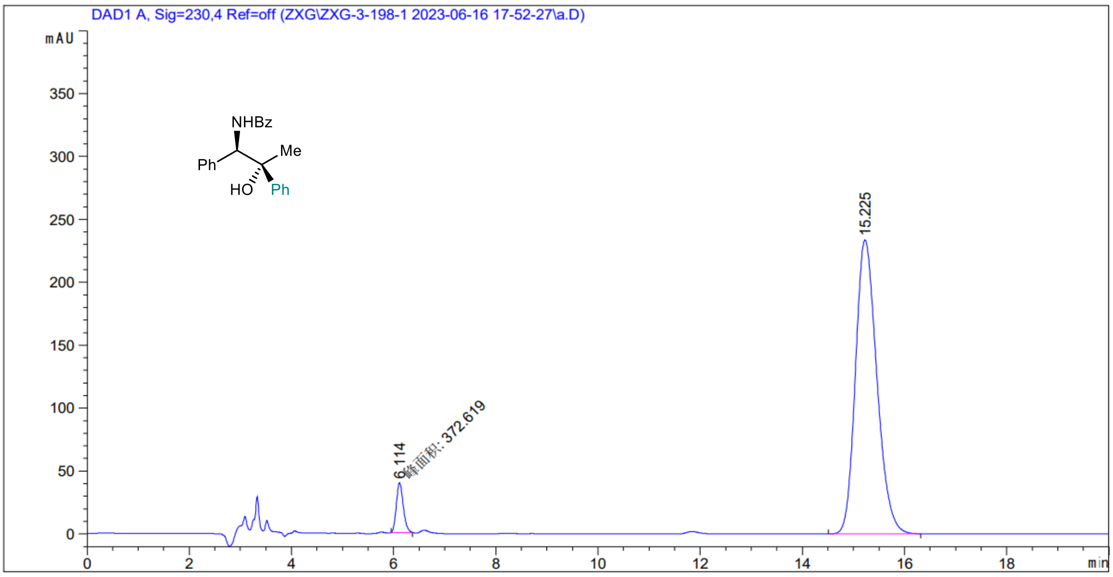

峰 #	保留时间 [min]	类型	峰宽 [min]	峰面积 [mAU*s]	峰高 [mAU]	峰面积 %
1	6.114	MM	0.1563	372.61865	39.72467	5.2081
2	15.225	BB	0.4509	6781.95313	233.45934	94.7919

Supplementary Figure 221. HPLC spectrum of N-((1*R*, 2*R*)-12-hydroxy-1-phenylpropyl-2-naphthalenyl)benzamide (7b)

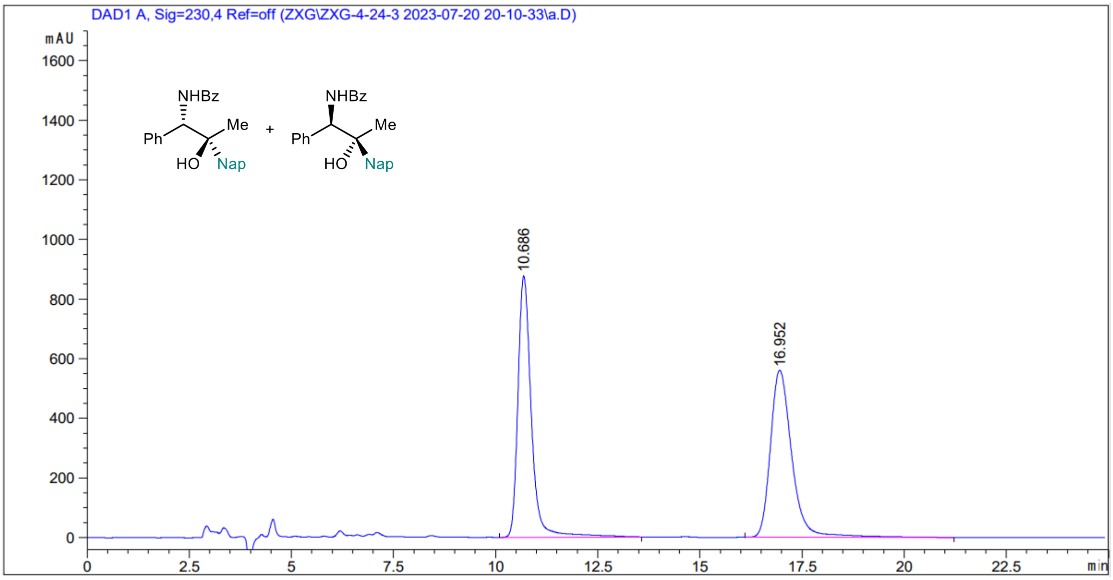

峰 #	保留时间 [min]	类型	峰宽 [min]	峰面积 [mAU*s]	峰高 [mAU]	峰面积 %
1	10.686	BB	0.3483	2.00004e4	876.35321	49.3225
2	16.952	BB	0.5579	2.05498e4	560.30396	50.6775

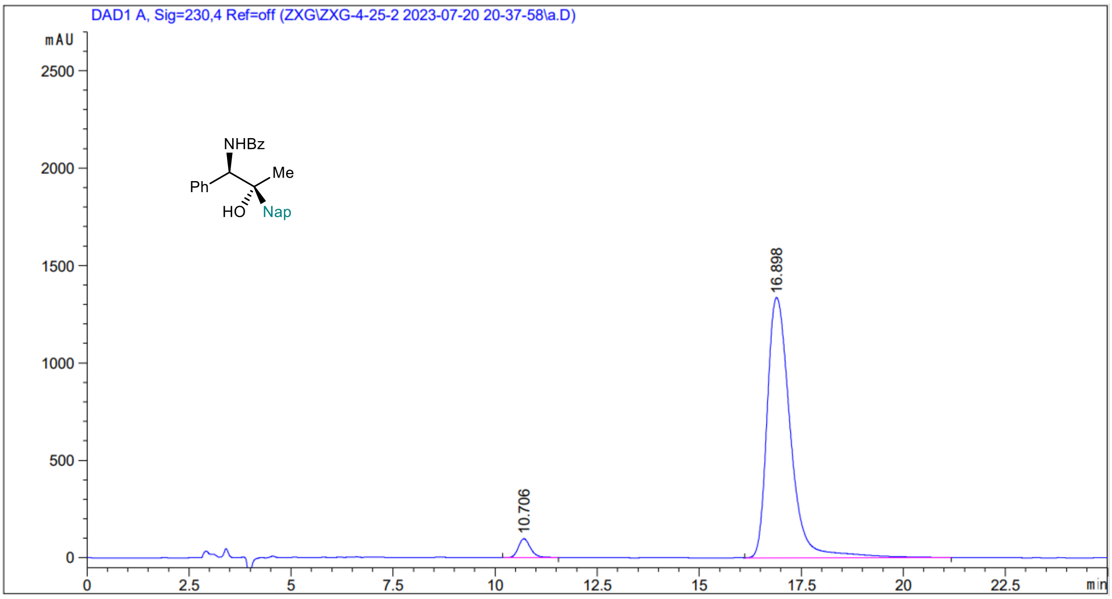

峰 #	保留时间 [min]	类型	峰宽 [min]	峰面积 [mAU*s]	峰高 [mAU]	峰面积 %
1	10.706	BB	0.3164	1999.49915	97.04222	3.6565
2	16.898	BB	0.6098	5.26839e4	1336.75342	96.3435

Supplementary Figure 222. HPLC spectrum of N-((1*R*, 2*R*)-12-hydroxy-1-phenylpropyl-2-vinyl)benzamide (7c)

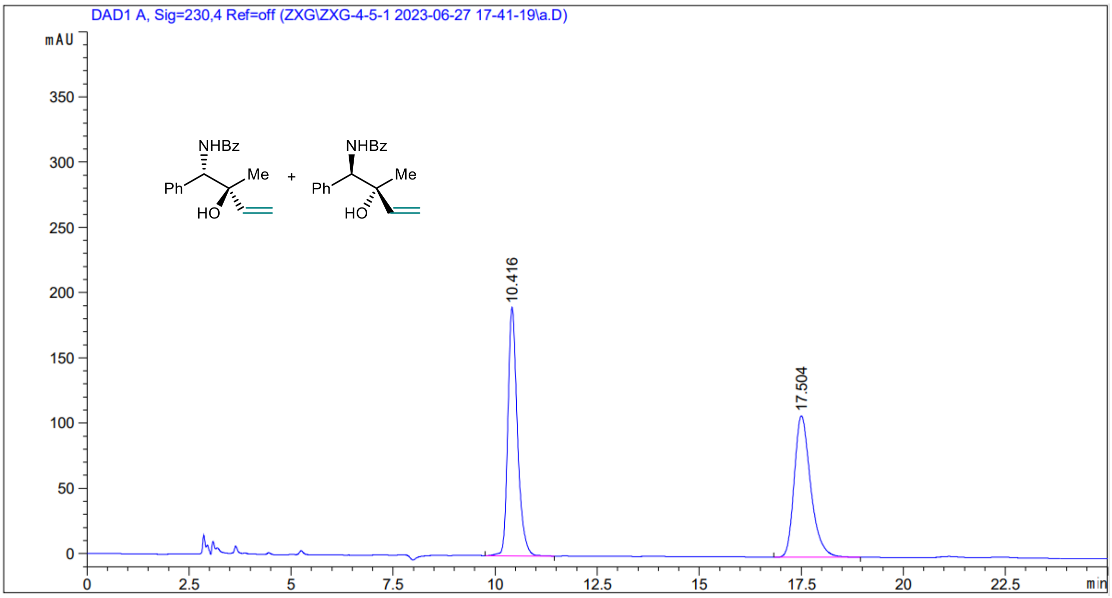

峰 #	保留时间 [min]	类型	峰宽 [min]	峰面积 [mAU*s]	峰高 [mAU]	峰面积 %
1	10.416	BB	0.2459	3102.21436	190.62340	50.5488
2	17.504	BB	0.4246	3034.85864	108.41052	49.4512

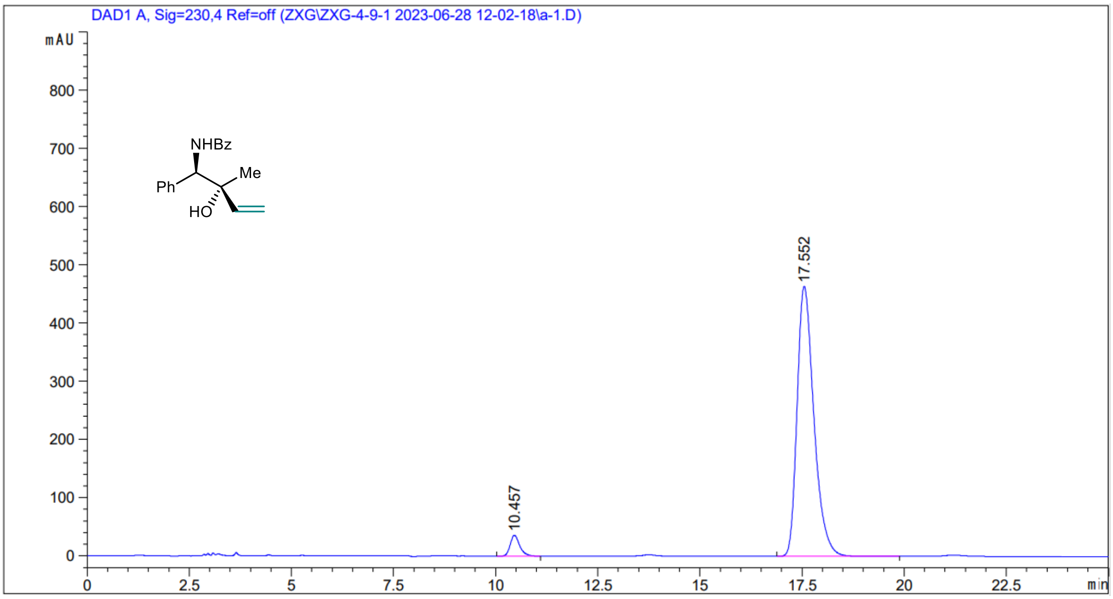

峰 #	保留时间 [min]	类型	峰宽 [min]	峰面积 [mAU*s]	峰高 [mAU]	峰面积 %
1	10.457	BB	0.2446	577.61078	35.72176	4.1751
2	17.552	BB	0.4375	1.32572e4	463.79486	95.8249

Supplementary Figure 223. HPLC spectrum of N-((1*R*,2*R*)-2-hydroxy-2-methyl-1-phenylbutyl)benzamide (7d)

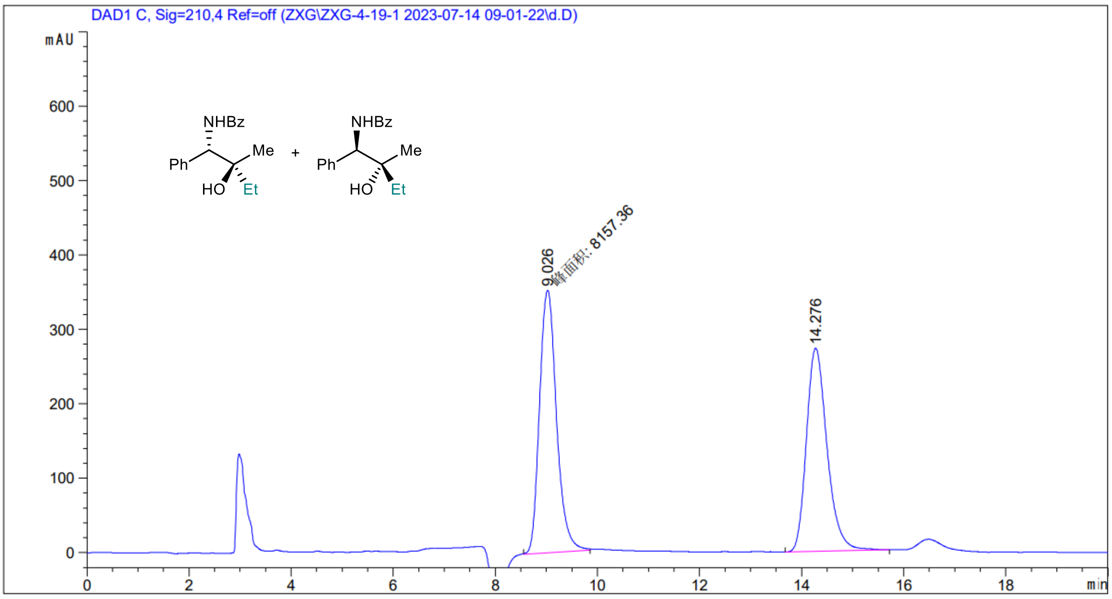

峰 #	保留时间 [min]	类型	峰宽 [min]	峰面积 [mAU*s]	峰高 [mAU]	峰面积 %
1	9.026	MM	0.3855	8157.35840	352.65695	51.5087
2	14.276	BB	0.4321	7679.48730	273.07758	48.4913

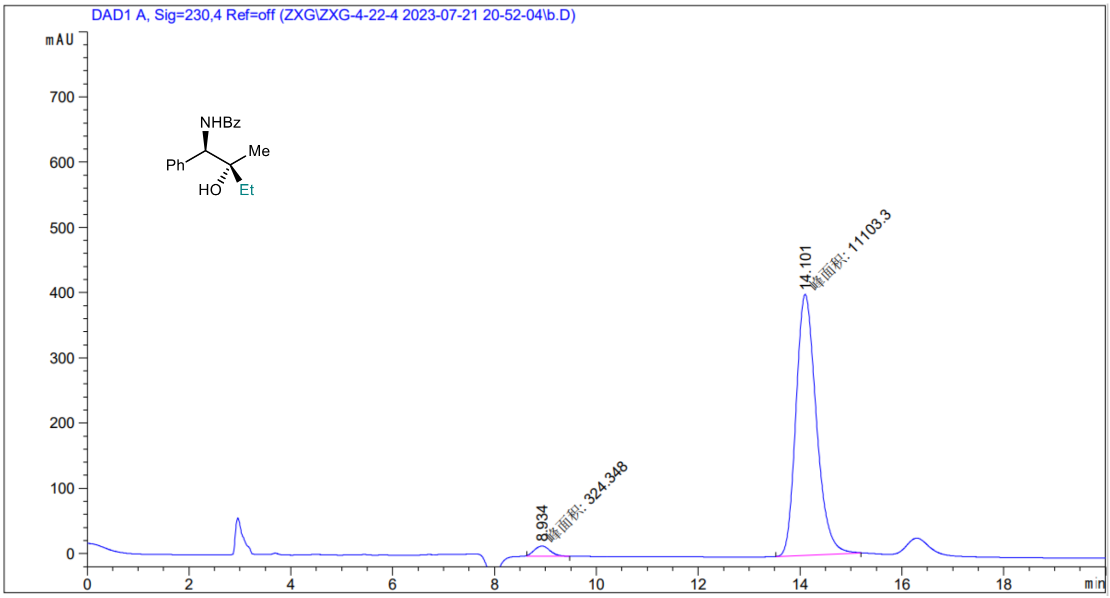

峰 #	保留时间 [min]	类型	峰宽 [min]	峰面积 [mAU*s]	峰高 [mAU]	峰面积 %
1	8.934	MM	0.3542	324.34790	15.26166	2.8383
2	14.101	MM	0.4625	1.11033e4	400.12106	97.1617

Supplementary Figure 224. HPLC spectrum of N-((1*R*,2*R*)-2-hydroxy-2-methyl-1-phenyldecyl)benzamide (7e)

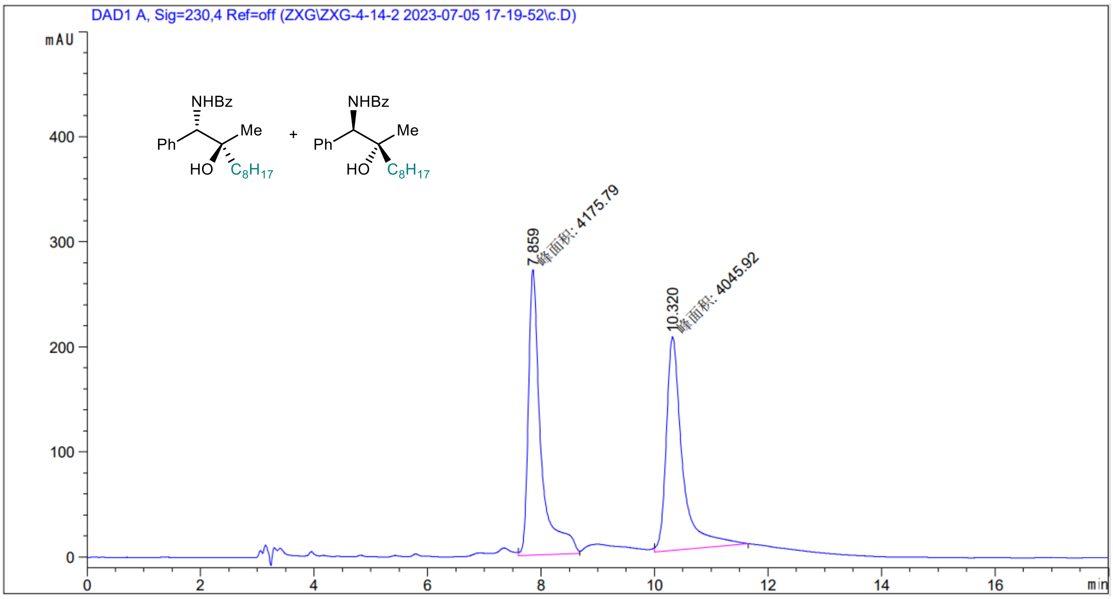

峰 #	保留时间 [min]	类型	峰宽 [min]	峰面积 [mAU*s]	峰高 [mAU]	峰面积 %
1	7.859	MM	0.2561	4175.78857	271.73682	50.7898
2	10.320	MM	0.3320	4045.91870	203.12959	49.2102

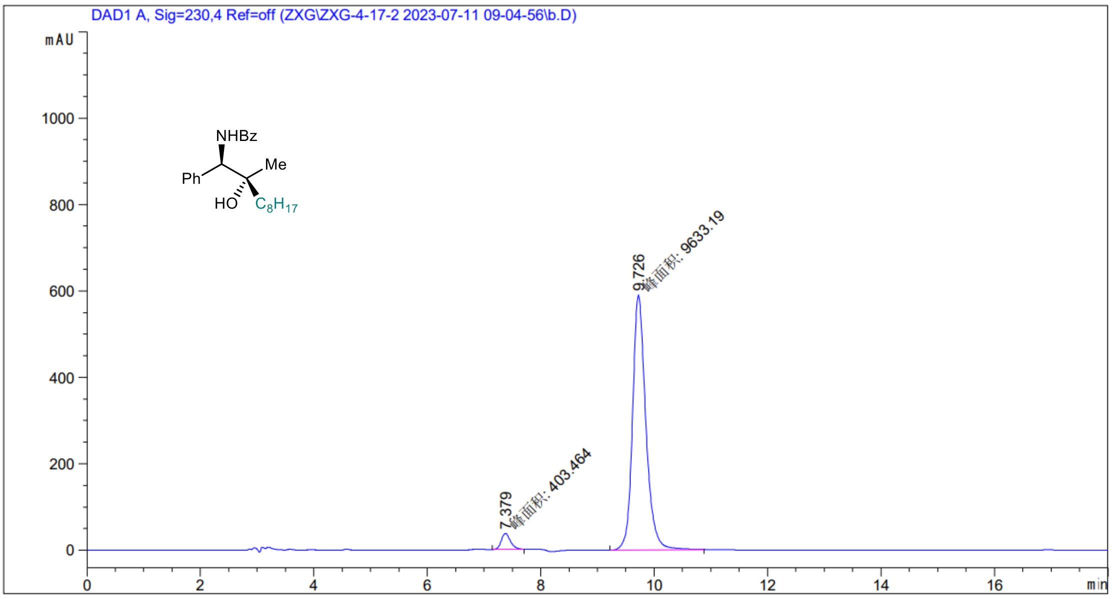

峰 #	保留时间 [min]	类型	峰宽 [min]	峰面积 [mAU*s]	峰高 [mAU]	峰面积 %
1	7.379	MM	0.1820	403.46365	36.94073	4.0199
2	9.726	MM	0.2721	9633.19336	590.10193	95.9801

Supplementary Figure 225. HPLC spectrum of N-((1*R*,2*R*)-2-hydroxy-2-methyl-1,3-diphenylpropyl)benzamide (7f)

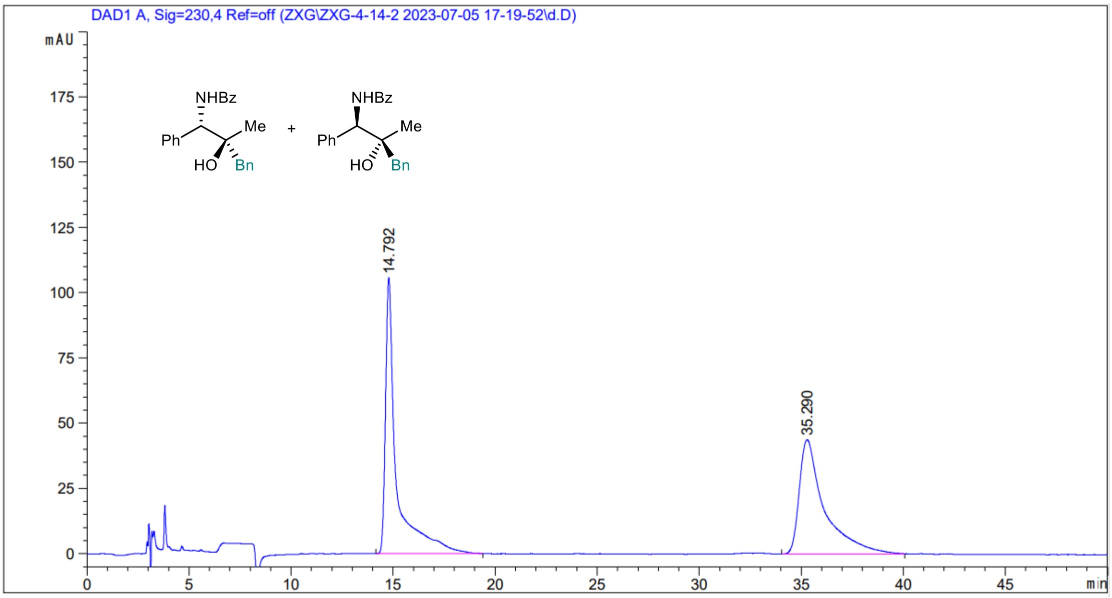

峰 #	保留时间 [min]	类型	峰宽 [min]	峰面积 [mAU*s]	峰高 [mAU]	峰面积 %
1	14.792	BB	0.5189	3940.09888	105.69219	51.3194
2	35.290	BB	1.1900	3737.50415	43.78151	48.6806

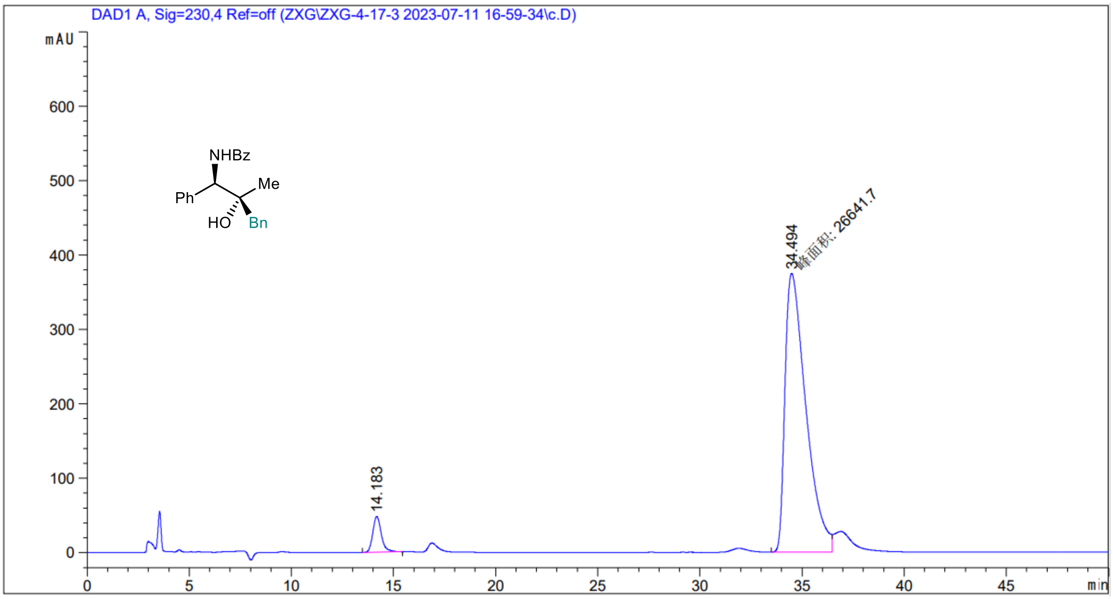

峰 #	保留时间 [min]	类型	峰宽 [min]	峰面积 [mAU*s]	峰高 [mAU]	峰面积 %
1	14.183	BB	0.4468	1394.99377	48.04272	4.9756
2	34.494	MF	1.1851	2.66417e4	374.67395	95.0244

Supplementary Figure 226. HPLC spectrum of N-((1*R*,2*R*)-2-cyclopropyl-2-hydroxy-1-phenylpropyl)benzamide (7g)

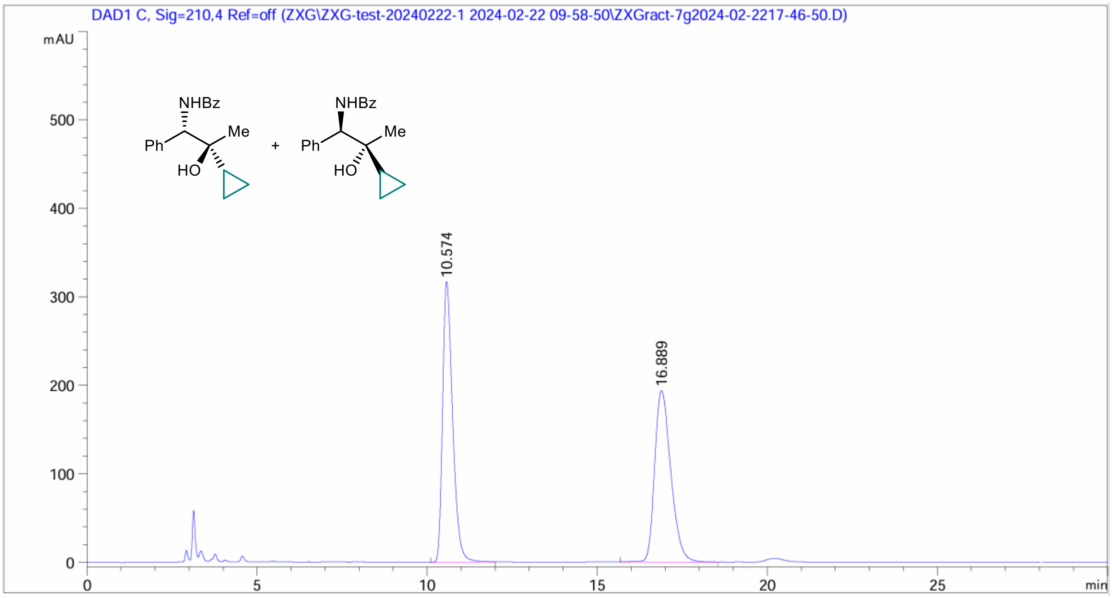

峰 #	保留时间 [min]	类型	峰宽 [min]	峰面积 [mAU*s]	峰高 [mAU]	峰面积 %
1	10.574	BB	0.3155	6507.17432	316.95526	50.3878
2	16.889	BB	0.5094	6407.02148	193.96448	49.6122

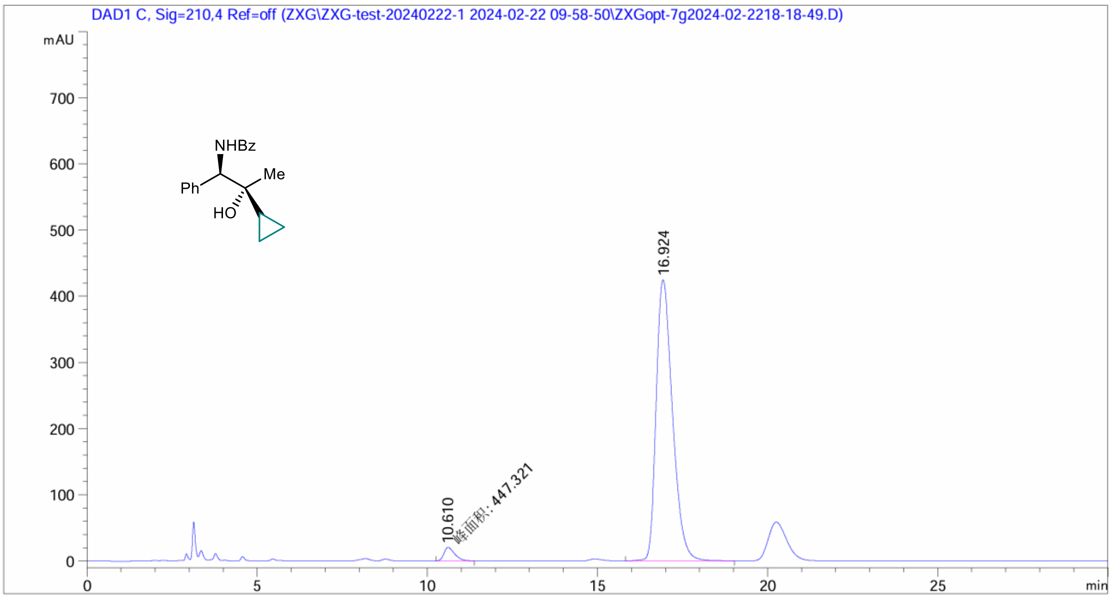

峰 #	保留时间 [min]	类型	峰宽 [min]	峰面积 [mAU*s]	峰高 [mAU]	峰面积 %
1	10.610	MM	0.3644	447.32120	20.45728	3.0616
2	16.924	BB	0.5170	1.41633e4	424.83139	96.9384

Supplementary Figure 227. HPLC spectrum of N-((1*R*,2*R*)-2-cyclohexyl-2-hydroxy-1-phenylpropyl)benzamide (7h)

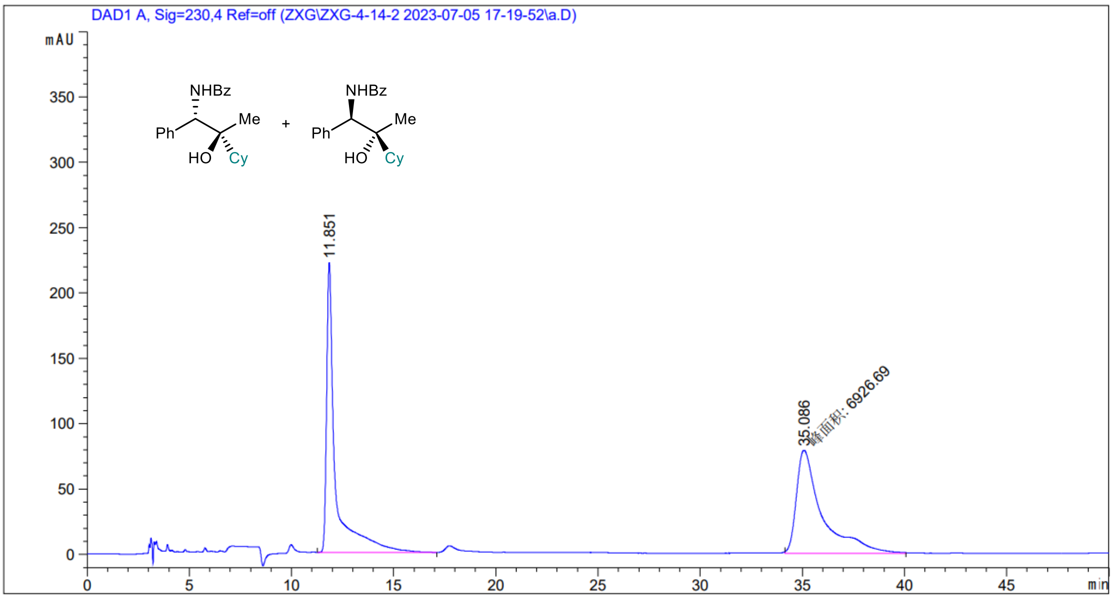

峰 #	保留时间 [min]	类型	峰宽 [min]	峰面积 [mAU*s]	峰高 [mAU]	峰面积 %
1	11.851	BB	0.4158	6617.50732	222.00711	48.8586
2	35.086	MM	1.4620	6926.68506	78.96198	51.1414

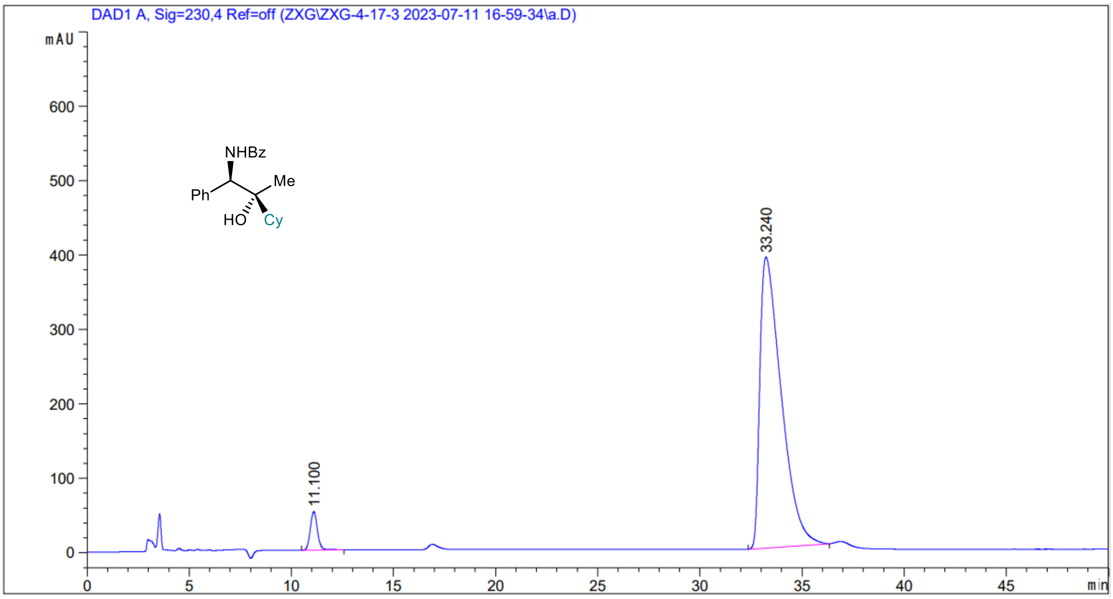

峰 #	保留时间 [min]	类型	峰宽 [min]	峰面积 [mAU*s]	峰高 [mAU]	峰面积 %
1	11.100	BB	0.3759	1244.65283	51.51672	4.1983
2	33.240	BB	1.0853	2.84017e4	391.08621	95.8017

Supplementary Figure 228. HPLC spectrum of (*S*)-N-(2-methyl-1-phenylallyl)benzamide (8)

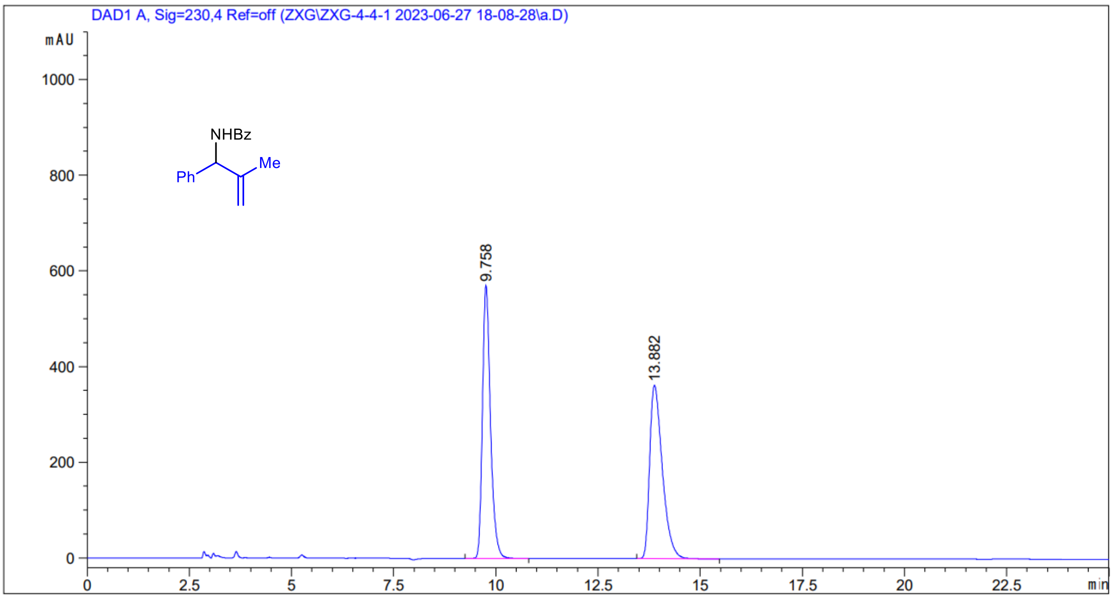

峰 #	保留时间 [min]	类型	峰宽 [min]	峰面积 [mAU*s]	峰高 [mAU]	峰面积 %
1	9.758	BB	0.2074	7798.13330	570.87585	49.9552
2	13.882	BB	0.3296	7812.10645	362.30804	50.0448

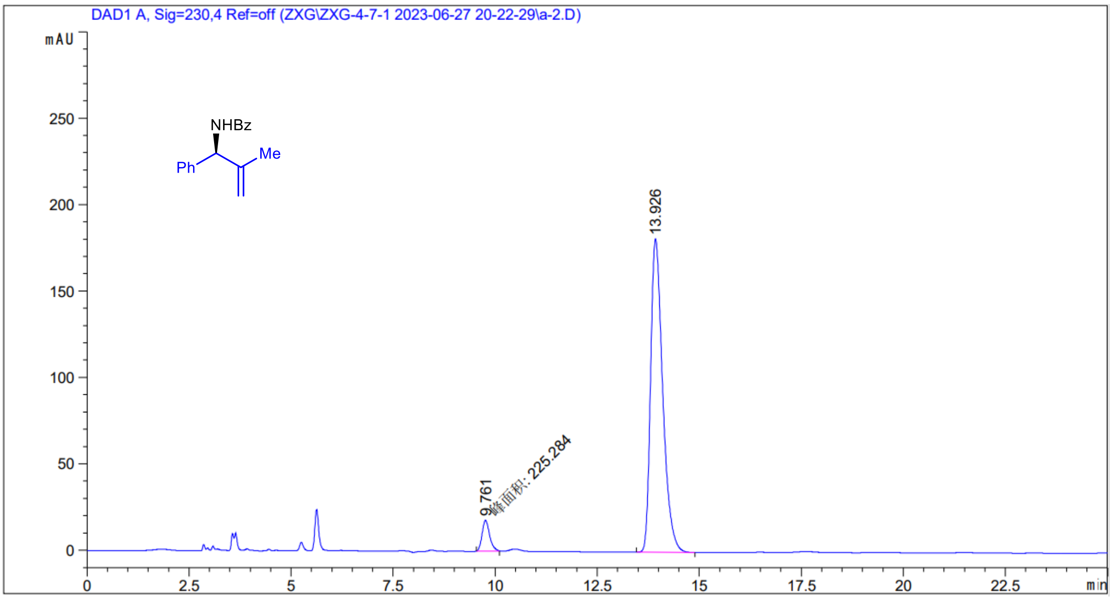

峰 #	保留时间 [min]	类型	峰宽 [min]	峰面积 [mAU*s]	峰高 [mAU]	峰面积 %
1	9.761	MM	0.2116	225.28360	17.74802	5.6920
2	13.926	BB	0.3141	3732.59131	181.37070	94.3080

Supplementary Figure 229. HPLC spectrum of N-((1*R*,2*S*)-2-hydroxy-1-phenylpropyl)benzamide (9)

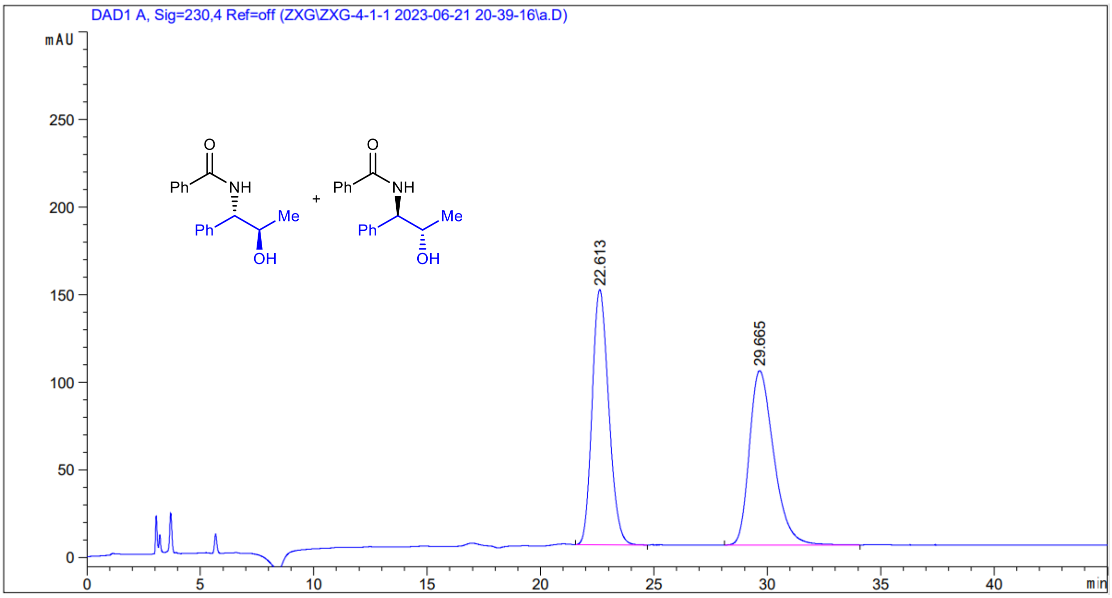

峰 #	保留时间 [min]	类型	峰宽 [min]	峰面积 [mAU*s]	峰高 [mAU]	峰面积 %
1	22.613	BB	0.7890	7390.08447	145.39743	49.8552
2	29.665	BB	1.1358	7433.00586	99.47038	50.1448

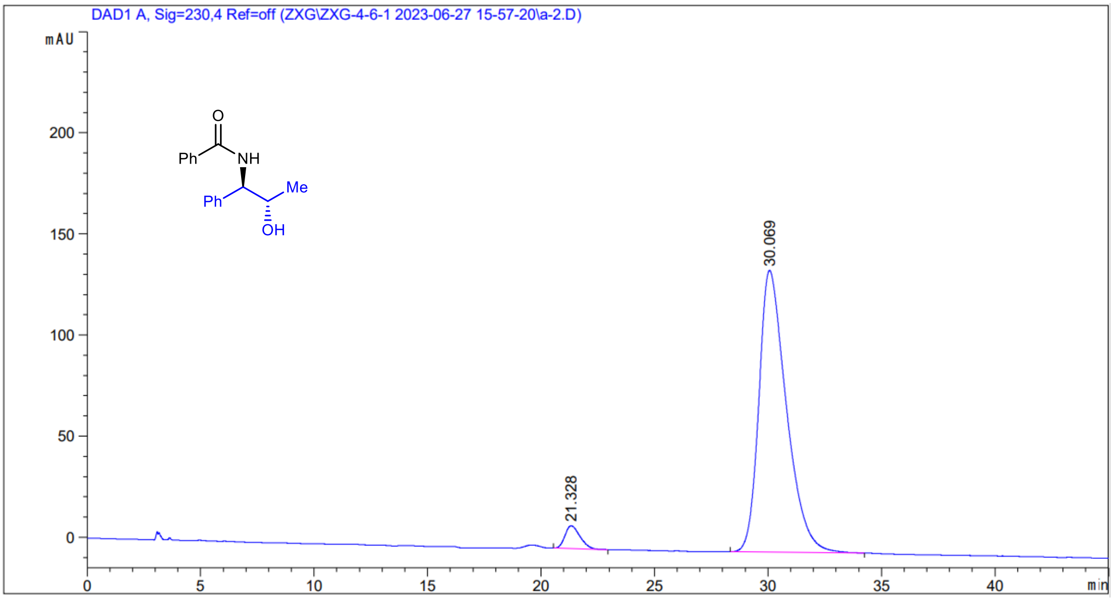

峰 #	保留时间 [min]	类型	峰宽 [min]	峰面积 [mAU*s]	峰高 [mAU]	峰面积 %
1	21.328	BB	0.6982	522.29999	11.27964	4.2567
2	30.069	BB	1.2452	1.17478e4	139.40140	95.7433

Supplementary Figure 230. HPLC spectrum of (4*R*,5*R*)-5-methyl-2,4-diphenyl-4,5-dihydrooxazole (10)

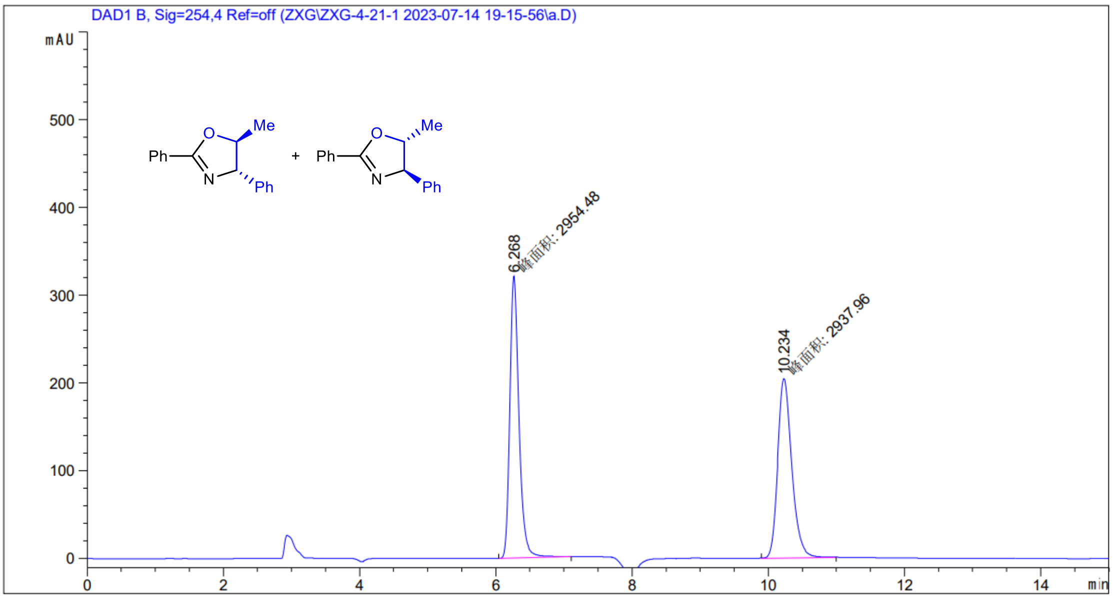

峰 #	保留时间 [min]	类型	峰宽 [min]	峰面积 [mAU*s]	峰高 [mAU]	峰面积 %
1	6.268	MM	0.1534	2954.47998	321.07089	50.1401
2	10.234	MM	0.2393	2937.96411	204.61395	49.8599

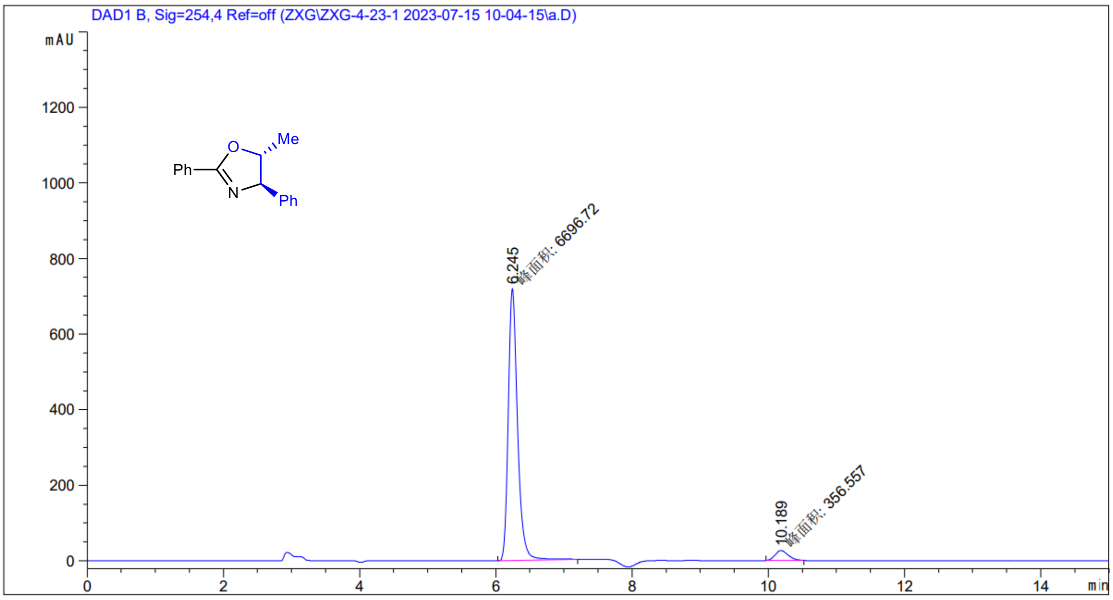

峰 #	保留时间 [min]	类型	峰宽 [min]	峰面积 [mAU*s]	峰高 [mAU]	峰面积 %
1	6.245	MM	0.1551	6696.71924	719.78809	94.9448
2	10.189	MM	0.2256	356.55722	26.33635	5.0552

Supplementary Figure 231. HPLC spectrum of (*R*)-5-chloro-1-methyl-N-(2-oxo-1-phenylpropyl)-1H-indole-2-carboxamide (11)

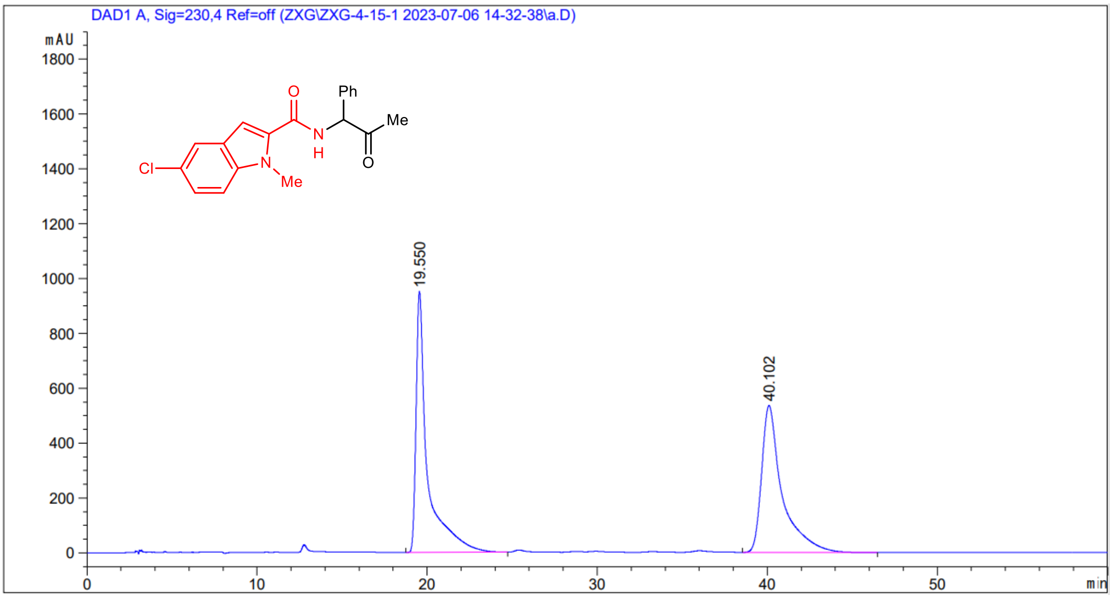

峰 #	保留时间 [min]	类型	峰宽 [min]	峰面积 [mAU*s]	峰高 [mAU]	峰面积 %
1	19.550	BB	0.6500	4.43550e4	951.17725	49.7611
2	40.102	BB	1.1976	4.47810e4	535.49988	50.2389

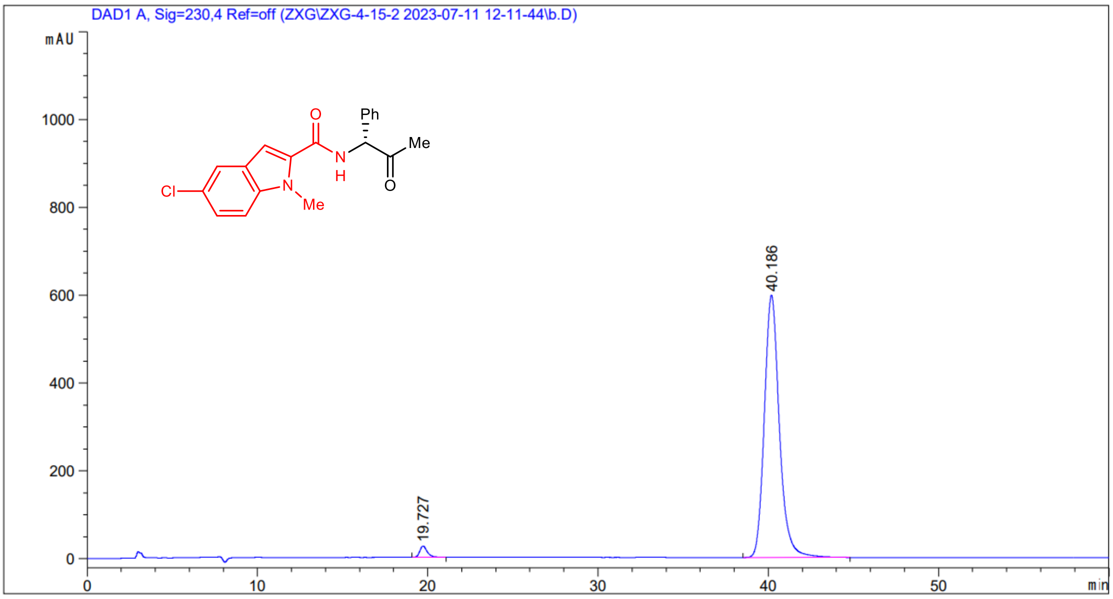

峰 #	保留时间 [min]	类型	峰宽 [min]	峰面积 [mAU*s]	峰高 [mAU]	峰面积 %
1	19.727	BB	0.4598	778.43835	25.66905	2.0810
2	40.186	BB	0.9339	3.66283e4	596.95422	97.9190

Supplementary Figure 232. HPLC spectrum of 5-chloro-N-((1*R*,2*R*)-2-hydroxy-1-phenylpropyl)-1-methyl-1H-indole-2-carboxamide (12)

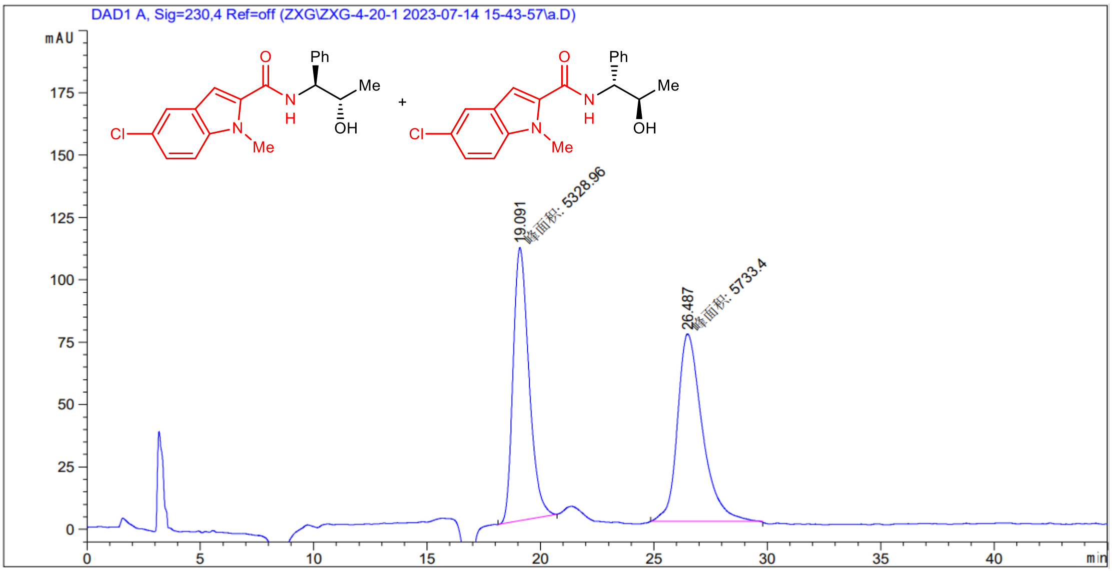

峰 #	保留时间 [min]	类型	峰宽 [min]	峰面积 [mAU*s]	峰高 [mAU]	峰面积 %
1	19.091	MM	0.8112	5328.96094	109.48487	48.1720
2	26.487	MM	1.2729	5733.39502	75.06999	51.8280

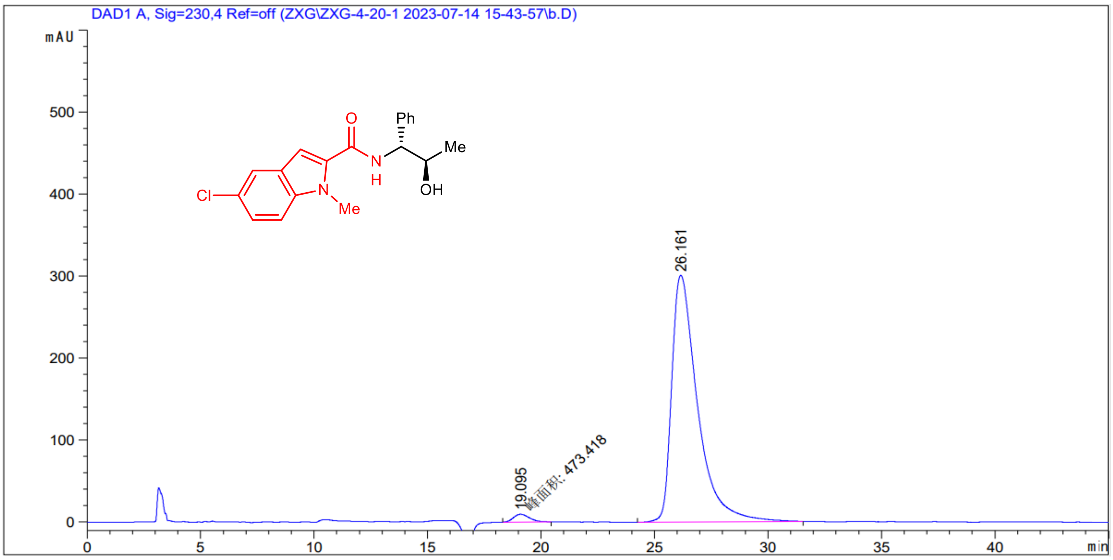

峰 #	保留时间 [min]	类型	峰宽 [min]	峰面积 [mAU*s]	峰高 [mAU]	峰面积 %
1	19.095	MM	0.8248	473.41754	9.56638	1.9514
2	26.161	BB	1.1647	2.37869e4	300.77417	98.0486

3 References

1. Green, S. P. et al. Thermal Stability and Explosive Hazard Assessment of Diazo Compounds and Diazo Transfer Reagents. *Org. Process Res. Dev.* **2020**, *24*, 67-84.
2. Nguyen, T. H. et al. Diaryldiazoketones as Effective Carbene Sources for Highly Selective Rh(II)-Catalyzed Intermolecular C–H Functionalization *J. Am. Chem. Soc.* **2024**, *146*, 8447–8455.
3. Armarego, W. L. F. & Chai, C. L. L., *Purification of Laboratory Chemicals-Six Edition*. Elsevier Inc., London, **2009**.
4. Xu, B., Zhu, S.-F., Dong, X.-Z., Zhang, Z.-C. & Zhou, Q.-L., Enantioselective N-H Insertion Reaction of α-Aryl α-Diazoketones: An Efficient Route to Chiral α-Aminoketones. *Angew. Chem. Int. Ed.* **2014**, *53*, 3913-3916.
5. Masui, M. & Shioiri, T., Stereoselective Synthesis of 1,2-Amino Alcohols by Asymmetric Borane Reduction of α-Oxoketoxime Ethers. *Tetrahedron Letters*, **1998**, *39*, 5195-5198.
6. Petrone, D. A., Yoon, H., Weinstabl, H. & Lautens, M., Additive Effects in the Palladium-Catalyzed Carboiodination of Chiral N-Allyl Carboxamides. *Angew. Chem. Int. Ed.* **2014**, *53*, 7908-7912.
7. Gaussian 16, Revision A.03, Frisch, M. J. et al. Gaussian, Inc., Wallingford CT, **2016**.
8. Becke, A. D., A New Mixing of Hartree–Fock and Local Density-Functional Theories. *J. Chem. Phys.* **1993**, *98*, 1372–1377.
9. Becke, A. D., Density-Functional Thermochemistry. III. The Role of Exact Exchange. *J. Chem. Phys*. **1993**, *98*, 5648–5652.
10. Grimme, S., Ehrlich, S. & Goerigk, L., Effect of the Damping Function in Dispersion Corrected Density Functional Theory. *J. Comput. Chem.* **2011**, *32*, 1456–1465.
11. Weigend, F. & Ahlrichs, R., Balanced Basis Sets of Split Valence, Triple Zeta Valence and Quadruple Zeta Valence Quality for H to Rn: Design and Assessment of Accuracy. *Phys. Chem. Chem. Phys.* **2005**, *7*, 3297–3305.
12. Weigend, F., Accurate Coulomb-Fitting Basis Sets for H to Rn. *Phys. Chem. Chem. Phys.* **2006**, *8*, 1057–1065.
13. Marenich, A. V., Cramer, C. J. & Truhlar, D. G., Universal Solvation Model Based on Solute Electron Density and on a Continuum Model of the Solvent Defined by the Bulk Dielectric Constant and Atomic Surface Tensions. *J. Phys. Chem. B.* **2009**, *113*, 6378–6396.
14. CYLview, 1.0b; Legault, C. Y., Université de Sherbrooke. (http://www.cylview.org), **2009**.
