# Supplementary material for: Enantioselective synthesis of chiral amides by carbene insertion into amide N–H bond
Source: Nat Commun. 2024 Jun 5;15:4793. doi: 10.1038/s41467-024-48266-5 (PMC11153641; doi:10.1038/s41467-024-48266-5)
Supplement: Supplementary file 3 — Description of Additional Supplementary Files [file 41467_2024_48266_MOESM3_ESM.pdf]

Description of Additional Supplementary Files

File name: Supplementary Data 1

Description: Cartesian Coordinates (Å) for Optimized Structures.
